# Posters Presentations

**DOI:** 10.1155/1996/35914

**Published:** 1996


					POSTER PRESENTATIONS
Topic: LIVER
POOl PO02
TREATMENT OF MALIGNANT LIVER TUMOURS BY
CRYOTHERAPY
Adam R, Akpinar E, Johann M, Kunstlinger F, Bismuth H.
Hepatobiliary Surgery and Liver Transplant Center, Paul Brousse Hospital,
Villejuif, France
The use of cryotherapy for the treatment of liver tumours has been established as a
therapeutic option when resection is not possible. The aim of this study was to determine
the real place of this treatment in the therapeutic stra.tegy of liver tumours.
Methods: From Oct 1993 to Nov 1995, 63 patients (pts) have been treated by cryotherapy
at our institution, either as a single treatment (Group 1-12 pts), as combined with partial
resection (Group 2-28 pts) or as complementary to a complete resection with no sufficient
matin of normal liver around the tumour (Group 3-23 pts). There were 13 hepatocellular
carc=nomas all with underlying cirrhosis, 25 metastases of colorectal cancer and 40
metastases of other malignant tumours. Mean number of tumours was 4.7 (Range 1-12) in
Group 1,4.3 (2-12)in group and 3.4 (1-12)in Group 3. Mean maximum tumour size was
63.5 mm (20-130), 40 mm (10-100), and 42 mm(16-100) respectively for the same groups.
We used the LCS 2000 device (Cryogenic Technology Ltd, Belper, UK) designed
specifically for hepatic cryotherapy to deliver liquid nitrogen to the t=p of a triple lumen
probe applied under ultrasound guidance to the lesion to be frozen.
Results: There were peroperative complications related to the procedure: one rupture of
the tumour during the freezing process and o,e perforation of the liver capsule. Both
complications were easily controlled by suture. Operative mortality within months was 1.5
% (1/63), unrelated to cryotherapy (cardiac infarct at day 3). Serum transaminases
increased post operatively in relation to the duration of cryotherapy and the number of
treated lesions They normalized within 5 days. In Group (Cryo alone), a reduction of
tumour size was observed in pts (33 %), with=disappearance of a treated lesion in one
case. Tumour markers were decreased in 4/5 pts with preoperative increased levels. Five
pts with huge multinodular lesions died of progression of the disease from to 11 months
after cryotherapy. Seven pts are alive of whom were subsequently submitted to hepatic
resection. In Group 2 (Cryo+Resection), a reduction in cryotreated tumoursize was
observed in 11 pts (40%). Decreased tumor markers,were demonstrated in 6 cases (22%).
Three pts died at 6-12 months of local (1) and extrahepatic recurrence (2). The remaining
23 pts are alive of whom 7 with local recurrence and 7 with recurrence outside the
cryotreated site .In Group 3 ('Adjuvant' cryotherapy), one patient died 2.5 months after the
procedure of sepsis unrelated to cryotherapy. The remaining 9 pts alive without disease
and 13 pts are alive with recurrence(1 local, 8 hepatic and extrahepatic). Overall, the
main determinants of recurrence following cryotherapywere maximum tumour size > 5 crn
and number of lesions >3. Of 12 pts with beth risk factors, 8 have currently recurred and 2
died of progressive disease.
Conclusion: Cryotherapy is a simple and safe procedure that may be useful in the
treatment of unresectable malignant liver tumours and as an adjuvant to liver resection.
Objective criteria of anti tumoral effects are demonstrated but need confirmation with a
longer follow-up. Selection of pts should exclude all those with large multinodulartumors.
REPEAT RESECTION OF LIVER METASTASIS FROM
COLORECTAL CANCER.
R Adam, H Bismuth, F Navarro, D Castaing, A Abascal.
Hepato-Biliary Surgery and Liver Transplant Research Center, Paul
Brousse, Hospital, Villejuif, France.
It is estimated that about 60% of patients submitted to hepatic
resection of metastasis from colorectal cancer will present a
recurrende. This recurrence is limited to the liver in about 30% of
cases. Repeat hepatectomy has been used increasingly in relation to
the low mortality and morbidity of hepatic resection. However, the
risk of these repeat hepatectomies, their long-term results as well as
the rationale for patient selection need to be clarified. For these
purpose, we have analysed over a period of 12 years (1983-1984) the
result of 57 rehepatectomies performed in 44 patients with hepatic
metastasis from colorecal cancer (2 hepatectomies 44, 3
hepatectomies 10; 4 heatectomies 3). These repeat hepatectomies
represented 19,5% of the 282 liver resections performed during the
same period for the same indication. The time interval between first
and second hepatectomies was over year in 23 patients (52%).
Extra hepatic disease was associated to hepatic recurrence in 11
patients (25%). Major hepatectomy (>3 segments) was performed in
50% of first resections, 36% of second resections, and only 15% of
third and forth resections. There was no post operative mortality
within two months. Per operative bleeding was not increased as
compared to that of first resections. Post operative morbidity was
11% (6/55) comparable to that of first resections. Overall survival
after repeat resection was 44% at 5 years with no difference related to
Dukes classification of initial colorectal tumor, to synchronous versus
metachronous metastasis or to local versus distant hepatic recurrence.
Conclusion: Repeat resection of liver metastasis from colorectal
cancer allows a long-term survival at least equal to that of first
resection with no mortality and comparable morbidity. This policy is
warranted when repeat hepatectomy is potentially curative.
P003 P004
CIRCADIAN VARIATIONS OF THE PREVALENCE OF ACUTE
VARICEAL BLEEDING IN CIRRHOSIS.
R Adam, P Ialongo, H Bismuth
Hepato-Biliary Surgery and Liver Transplant Research Center, Paul
Brousse, Hospital, Villejuif, France.
The pathogenesis of variceal rupture is not well understood. Risk
factors include increasing size of varices, red signs at endoscopy,
severity of liver disease and increase portal pressure. Circadian
variations of variceal haemorrage have been reported but are still
uncompletly documented. In this study, a total of 193 episods of
gastrointestinal bleeding in cirrhotic patients with portal hypertention
have been reprospectively analysed. Were excluded from the study all
the bleeding episodes unrelated to a variceal rupture, those with a
timing not clearly documented and those revealed by a maelena since
their diagnosis may be differed as compared to the real time of
bleeding. Were included in the study 33 variceal ruptures presenting
with hematemesis and diagnosed by emergency upper gastrointestinal
endoscopy. There were 25 males and females with alcoholic cirrhosis
(n=23, 70%), virus related cirrhosis (n=9, 27%), or biliary cirrhosiS
(n=l, 3%), Child B in 12 cases (36%) and Child C in 21 cases (64%).
The 24 hour-scale was divided in 6 periods of 4 hours each.
Results: The frequency of variceal bleeding was 33% (11/33) between
5.00 and 9.00, 9% (3/33) between 9.00 and 13.00 h, 0% between 13.00
and 17.00 h, 48% (16/33) between 17.00 and 21.00, 3% (1/33) between
21.00 and 1.00 and 6% (2/33) between 1.00 and 5.00. The'2 perio
of higher prevalence were compared to all the other periods in terms of
severity of bleeding with the following results
Gr (5-9h) Gr2 (17-21h) G3 (other) p
No. Bleeding 11 (33%) 16 (48%) 6(18%) <0.05
Blood units 9.1+8.9 6.2+6.5 3.5+3.2 NS
Rebledding 5 (45%) 4 (25%) (17%) NS
Hospital Death 4 (36%) 5 (31%) 1(17%) NS
Conclusion: There are circadian variations of the frequency of variceal
bleeding in the cirrhotic patient with 2 peaks at the beginning and the
end of the night, possibily related to known circadian variations of
portal pressure. These variations should be considered to optimize drug
therapy during the periods of higher prevalence of bleeding.
PREVALENCE OF CHOLELITHIASIS IN C].,HOSIS.
C.A88elopoulos,G.Simitzis,L.Floros,C.Papanastasopoulos,N.Falirees,
ICKounela,B.Papanastasopoulos
2nd Dept. Internal Medicine and Hepatologicel center of General
Hospital ofAthenes.Oreece.
The aim of this study is to investigate the prevalence of
cholelithiasis in cirrhotic patients () and fairs alTe this.This is
interesting, bec,a-use there is incz'eace prevalence of Hbs A8 in
Oreek popular (8-I0%). We studied 79(crp), 64 M and 15 F, mean
age 63,13 years (38-82). There were 43 patients with alcoholic,, 26
HBV(+), 7 HCV(+), 2 autoimmune and alcoholic and HBV(+)
cirrhosis. Cholecystomised patients weren't included in study. We
inves'figsted fears such as age, sex, setiology of cirrhosis, ascites,
hypersplenism, coexistence of disbctes melitous (DM), hesmolysis
and Child system and their correlation with prevalence of
cholelithiasis in (cp). We researched prevalence of lithiasis in
control group of' I00 hospitalized non-cirrhotic patients (mean age
61,8 yers,80M and 20F). Cirrhosis was confirmed with biopsy end
cholelithiasis was checked by ultrmound. We used X2 tes
and ANOVA test for stst/stical analysis.
There were 29, 26 and i4 (cp) as Child A,B,C, respectively.
Cholelithiasis was found 39% (31 pts, 24 Mend 7 F) in (cp) versus
16% in control group (p=0,0000). Prevalence of lithiasis in cirrhotic
females was 47% versus 37,5% in males (pNs)and in (cp) with age
<55 years was 63%, versus 8,3% in patients with same age in
control group (p=0,000). 44,5%, 16,6%, 64%, wm prevalence of
lithissis in alcoholic, HBV(+) and HCV(+) cirrhosis
respectively.Lithiasis was found in 68%, 87% and 84% in ascites,
hypersplenism mind hemnolysis respectively, versus 45% (p=0,03),
58% (p=0,01) and 70A (p--0,03) in. (cp) with no
ascites,hypecplenism and hemolysis respectively. Lithiasis
found 55% in child A, 16,6% in child B (p=0,000) and 64% in
child C.FinanlIy lithiasis was 70% in (cp) with (DM), versus 34% in
(c) with no (DM)(p=0,0000).
In conclusion prevalence of cholelithiasis is twice more frequency
in (cp) than non- cp and is correlated with coexistence of
ascites,hesmolysis, hypersplenism and (DM).It is more flquently in
atients <55 years ,while it has low rate in (cp) child B.
71
PO05 PO06
IN VITRO ASSESSMENT OF THE UPTAKE OF LIPIODOL BY
LIVER TUMOURS; IS IT A MECHANISM SPECIFIC TO LIPIODOL ?
ILA.M. A! Muf0, S. Bhattacharya, K.E.F. Hobbs, M.C. Winslet
University Department of Surgery, Royal Free Hospital & School of Medicine,
London, NW3 2QG, ENGLAND
Lipiodol, an iodinated ethyl ester derivative of poppy seed oil has
been shown to be taken up and retained selectively by primary and some
metastatic liver cancers, following intra-arterial injection into the hepatic
artery. The mechanism of uptake and retention of Lipiodol is unknown. The
uptake and retention ofLipiodol, constituent and non-constituent fatty acids by
primary and colorectal hepatic metastases in tissue cultures was assessed using
electron microscopy and computer assisted image analysis following silver
nitrate impregnation staining.
Cultures of Hep-G2 (human hepatocellular carcinoma cell line),
LOVe (colorectal cancer cell line) and SW620 (eel.rectal metastatic cancer
cell line) were studied. A control non-malignant cell line was used in the form
of HUVEC (human umbilical vein endothelial cells). The cultures were
exposed to Lipiodol (concentrations of 1%, 2% & 4%) for variable duration (3,
6, 12, 24, 48 & 72 hours). Electron microscopy revealed evidence ofuptake of
Lipiodol bypinocytosis, as early as three hours ofexposure. Membrane-bound
cytoplasmic vesicles oflipid particles were seen in all the cell lines (malignant
and non-malignant). However, at 48 & 72hours ofexposure, Lipiodol vesicles
were seen inside the nuclei of Hep-G2 cancer cells alone, suggestive of
incorporation during cell mitosis.
The uptake of Lipiodol by the these cell lines was compared to the
uptake of 2% solutions of Linoleic acid, Iodinated Linoleic acid, Oleic acid,
Palmitic acid, Stearic acid, Docosahexanoic acid and Eicosapentanoic acid.
All cell lines demonstrated uptake of all fatty acids, in a similar fashion as that
ofLipiodol. The uptake ofLinoleic acid and oleic acid in the Hop-G2 cell line
was much faster than that of Lipiodol, with intra-nuclear incorporation as
early as 12 hours.
This study indicates that the uptake and retention ofLipiodol is not a
phenomenon specific to Lipiodol. All cell lines showed cytoplasmic
incorporation ofLipiedol and other fatty acids in the form ofmembrane-bound
vesicles. However, only Hop-G2 showed intra-nuclear incorporation of
Lipiodol and all the other fatty acids, a specific phenomenon to human
hepatocellular carcinoma cells.
QUANTIFICATION OF UPTAKE AND RETENTION OF IODISED
OIL LIPIODOL IN PRIMARY AND SECONDARY HEPATIC
MALIGNANCY
ILA.M. A! Mufti, S. Bhattacharya, M.C. Winslet, K.E.F. Hobbs
University department of Surgery, Royal Free Hospital & School of Medicine,
Pond Street, London, NW3 2QG, ENGLAND.
Intra-arterial iodised oil, Lipiodol, which is differentially taken up
and retained by an unknown mechanism in primary and metastatic liver
tumours may be used as a therapeutic vehicle for adjuvant therapy. Uptake and
retention of Lipiodol by hepatic constituents was assessed in vitro. Human
hepatocytes, HUVEC (human umbilical vein endothelial cells), Hop-G2
(hepatoma), SW620 (cole-rectal metastatic cancer), LoVe (cole-rectal hepatic
cancer) and U-937 (histiocytic lymphoma) cell lines under standard tissue
culture conditions were evaluated over 48 hours. Intracellular uptake of
Lipiodol was assessed by computer-assisted image analysis of selective silver
nitrate (Ag2NO3) impregnation staining and electron microscopy.
Quantification ofLipiodol uptake by Image Analysis
(Integrated Optical Density in arbitrary units) n=12
2% Liplodol Hepatocyte HUVEC Hop-G2 SW620 LoVe U-937
6 hours 86.5 115.2 81.8 66 46.6 99.7
12 hours 92.3 121.7 89.5 76.2 59.4 145.2
24 hours 81.2 156.3 121.2 97.4 74.6 136.1
48 hours 65.3 119.7 139 128.7 75.2 114.4
Control 9.9 10.5 11.9 12.6 14.8 18.9
Hepatocytes, HUVEC and U-937 cells demonstrated rapid uptake
with subsequent decline suggesting metabolism or excretion. Hop-G2 showed
rapid and sustained uptake. Comparative uptake by SW620 and LoVe was
15% and 40% lower respectively. Electron microscopy demonstrated
cytoplasmic incorporation ofLipiodol in the form ofmembrane-bound vesicles
in all the cell lines, with intra-nuclear incorporation in Hop-G2 alone.
These results may explain the early clearance of iodised oil from the
normal liver and the preferential uptake and retention by hepatocellular
carcinoma compared to hepatic cole-rectal metastases. This suggests its
therapeutic use in metastatic disease may be limited by unsuitable tumour
specific pharmacokinetics.
PO07 PO08
THE EFFECT OF UROGRAFIN ON THE PARTICLE SIZE AND THE
UPTAKE OF LIPIODOL IN TARGETED THERAPY FOR HEPATO-
CELLULAR CARCINOMA (HCC)
R,A.M. A! Mufti, K.E.F. Hobbs, M.C. Winslet
University department of surgery, Royal Free Hospital School ofMedicine,
Pond Street, London, NAV3 2QG, ENGLAND.
Lipiodol, an iodinated ethyl ester derivative ofpoppy seed oil, has been used in
targeted therapy for primary and some metastatic hepatic cancers. Lipiodol is
insoluble in water, and Urografin (diatrizoate) is used clinically to emulsify
Lipiodol prior to its intra-arterial injection. The effect of Urogr'n in
combination with Lipiodol was assessed in tissue cultures of Hep-G2
(hepatoma) cell line. The cell cultures were exposed to 2% Lipiodol alone and
2% of Lipiodol with 2% Urografin for variable duration (3, 6, 12, 24, 48 and
72 hours).. The uptake and retention of Lipiodol was assessed using
computer-assisted image analysis. The size of the Lipiodol particles were
measured after 2 minutes agitation ofthe oil suspensions with culture media.
Mean Num
10 # 1025 # 25-50 # 50-100 # >100 # Total
2% Lipiodol 890 (101) 110 (38) 17 (3) 4 (1) 4 (1) 1,025
!2% Lipiodol/Urografln 575 (73) 220 (56) 21(4) 7 (2) 8 (2) 831
The mean size of the Lipiodol particles when combined with Urografin was
considerably larger than that of Lipiodol alone, with a reciprocal reduction in
the number ofParticles.
Quantification of Llpiodol uptake by image analysis
(O units) n=10
hours 6 hurs 12 hours 24 hours 48 hours 72 hours
2% Lipiodol 52.2 91.4 92.4 131.2 139 142
2% Lip.+ Urografln 49.4 59.8 dl.8 89.8 115.4 128
The addition of Urografin to Lipiodol resulted in a significant delay in the
uptake of Lipiodol by the cancer cells in vitro. A similar phenomenon in vivo
may interfere with the delivery ofLipiodol-targeted therapy for these tumours.
The continued use of Urografin to emulsify Lipiodol in clinical practice
requires further evaluation.
RESECTION AND CRYORESECTION OF LIVER
TUOUR
B.Alperovich
Siberlam, Medical Un.ivercity,Tomsk,Russia
Surgical treatment of liver tumeur of dife-
rsnt genesis attracts much ttention o .sur-
geons.It depends beth on, positive results of
the treatment area on. a number of advautages
in, comparison with the transplautation, o
the organ..
Ne operated 108 patient with different li-
ver turnouts.All of them were made liver re-
section, of different volume,icludig 33 ma-
lignam.t turnouts,75 innocent ones.56 liver res
ection of hemaugioma, 19 resection, of adenema.
33 liver resectiens o malignan,t turnouts of
differen.t volume were mad with 6 lethal
outcome.56 reections with hemngioma with-
out deats,19 resections with adenoma with
death.0riginal methods elaborated in the
clinic were eed in resections.
52 liver resections were made with cryotech-
nique(23 liver resections,29 cryodestructi-
on.s of liver stump after resection,s).Advanta-
ges of cryoeperations:decrement o haemorrhage,
ablastous operations, prophylaxis of the re-
cidivation of the illness.An original operati
on of hemangioma was elaborated ligation
of turnout nutrient vessels anA turnout cryo-
destruction..
Liver resection, operations of malignant and
innocen.t tumours work well at once and in.
the distam.t future.Cryetechnique decreases
haemorrhage in operations,in,creases ablasta-
tion and prevents from the recidivation of
the illness,
72
PO09 P010
PARTIAL SPLENIC EMBOLIZATION FOR PATIENTS WITH
BILIARY ATRESIA DEVELOPS A HYPERSPLENISM
H. Ando. T. Ito, T. Seo, F. Ito, K. Kaneko
Department of Surgery, Branch Hospital, University of Nagoya
School of Medicine, Nagoya, Japan
Partial splenic embolization (PSE) has been used as a
palliative treatment for hypersplenism in adults and children.
However, there has been no previous report of its use for treatment
ofjaundice. The ptlrpose of this study was to investigate the
utility of PSE forjaundice in patients with biliary atresia who
developed hypersplenism.
PSE was performed in eight patients with biliary atresia
who developed hypersplenism following Kasai procedure. Seven
of them had lost complete jaundice with Kasai procedure, but
became icteric thereafter. Jaundice remained unchanged in the
initial postoperative period in the last patient but subsequently
worsened. White blood cell, platelet, and red blood cell counts,
hematocrit, serum hemoglobin, and serum concentration of
glutamic oxaloacetic transaminase (GOT), glutamic pyruvic
transaminase (GPT), alkaline phosphatase, lactic dehydrogenase,
albumin, and total bilirubin were evaluated month prior to PSE
and 1, 2, 3, 6, 9, 12, 18, and 24 months following PSE.
Total bilirubin decreased in all patients following PSE from
8.6 + 3.6 mg/dL to 3.0 + 1.0 mg/dL. This change took place
within 3 months of PSE, and correlated with an increase in the red.
blood cell count.
PSE is a useful method for reducing serum bilirubin
concentrations in patients with hypersplenism following Kasai
procedure for biliary atresia.
STOMA IMPAIRS LIVER FUNCTION IN PATIENTS WITH
BILIARY ATRESIA
H. Ando. T. Ito, T. Seo, F. Ito, K. Kaneko
Department of Surgery, Branch Hospital, University of Nagoya
School ofMedicine, Nagoya, Japan
Thirty-two patients with biliary atresia who had had hepatic
portoenterostomy with stoma were studied for liver function
(SGOT, SGPT, g-GTP, ALP, and total bilirubin), cholangitis, and
stoma bleeding both before and after closure of the stoma.
Liver enzymes improved significantly within month after
closure of the stoma. These enzymes at month prior to stoma
closurversus month following closure were as follows (mean +
SE): SGOT, 144 + 13 to 99 + 9 IU/dL (p<0.01); SGPT, 147 + 14
to 88 + 8 IU/dL (p<0.01); g-GTP, 322 + 46 to 201 + 25 IU/dL
(p<0.01); ALP, 1272 + 124 to 1037 + 111 IU/dL (p<0.01).
They remained low thereafter. The total bilirubin concentration
was the only liver function test which did not change significantly
following closure. Cholangitis was observed in 20 patients (62.5
%), and major hemorrhage from the stoma site was seen in 14
patients (43.8 %) prior to closure.
We conclude that an external conduit in patients with biliary
atresia dose not prevent cholangitis, causes bleeding from the
stoma site and impair 5 liver function.
P011 P012
HEPATIC ARTERIAL INFUSION CHEMOTHERAPY FOR
RESECTED LIVER IN COLORECTAL CANCER
METASTASES
T. Aoki. Y. Koyanagi. T. Aoki. T. Ashizawa, A. Tsudhida. T. Ozawa,
O. Uda. T. Hashimoto. K. lnoue, H.Saito.l.Sonoda,S.Rai.A.Nakajima.
T.Majima
Department of Surgery. Tokyo Medical College. Tokyo, Japan.
INTRODUCTION; Recently hepatectomy of liver metastases in
colorectal cancer improved the survival for the patients of colorectal
cancer. However the rate of recurrence in remaining liver is still high
suggesting that potential metastases might exsist in the other parts of the
liver even after the apparent metastasis was resected. So the survival time
of the patients in colorectal cancer with liver metastases is not
sufficiently good. We report the results of hepatic infusion chemotherapy
after hepatectomy of liver metastases in colorectal cancer.
MATERIAL AND METHOD; Since January 1985 we have
performed 59 cases of hepatic resection for hepatic metastases from
colorectal cancer. The cases were divided into two groups: Hepatic Arterial
Infusion (HAl) group which included 32 cases regimen 5-FU
167mg/m2/day for 14 days I.A 5-FU 334-500mg/m2/week
intermittently.one shot or hours), and non-HAl group consisting of 27
cases regimen 5-FU 200mg orally. 5-FU 250mg /week I.V or non
therapy) .We analyzed the differences of two group in the back ground.the
the survival rate trod complications. Survival curve were established
using the Kaplan-Meier method.and these results were analyzed by
generalizd Wilcoxon ,and Logrank test. We also investigated DNA
histogram pattern and nucle,-u" DNA-protein contents.
RESULTS: The l-year survival rate was 90.1% in the HAl group and
51.2 % in the non-HAl group, the 3-year survival was 58.7% ,and 33.4%,
while the 5-year survival was 25.6% and 17.5% respectively. The results
in the HAl group were significantly better than in the non-HAl group.
Hepatic resection combined with arterial infusion chemotherapy can be
effective treatment for hepatic metastasis from colorectal cancer.
THE EXPERIENCES OF RECONSTRUCTION OF
HEPATIC VEIN IN THE LIVER RESECTION
T.Aoki, I.Sonoda, A.Masuhara, K.Inoue, H.Sait0, T.Hashimoto,
O.Uda, T.Aoki, A.Tsuchida, T.Ashizawa, Y.Koyanagi
Department of Surgery, Tokyo Medical College, Tokyo, Japan
In order to maintain liver function after the the liver resection it
is very important to preserve the remnant liver function by the
decrease of resection volume of liver. When the liver tumor
invades into main vessele such as hepatic vein, portal veini and
nferior vena cava, the resection and reconstruction of these
vessles must :be necessary to achive this purpose. We report two
cases which were performed the reconstruction of hepatic vein.
Case 1. 67 year-old-male was admitted to the hospital because
of hepatic recurrence after gastrectomy for gastric cancer. Liver
tumor was located in the $8 and about 7cm diameter in size and
it invaded into right hepatic vein. Tumorectomy with the
reconstruction of hepatic vein was performed. In the
reconstruction the autograft (right external iliac vein) was used.
In 5days after surgery liver function developed to normal
range.
Case 2. 55 year-old-male was admitted to the hospital because
of hepatic tumor. Tumor was located in the $2,--,4 with invasion
into middle hepatic vein. And extended left lobectomy with the
reconstruction (right external iliac vein) was performed. During
the reconstruction we used heparin coated tube for bypass from
middle hepatic vein (distal site) to the inferior vena cava.
73
P013 P014
A NEW TECHNIQUE OF ONE STAGE TOTAL HEPATECTOMY
IN THE RAT
I.Astarciolu_ H. Astarcioglu, D. Azoulay A. Lemoine,
H.Bismuth Hepatobiliary Surgery and Liver Transplantation Center,
Paul Brousse Hospital, 94800 Villejuif, France
This technique is described to perform a total (100%) hepatectomy in
the rat, in a single stage with the preservation of the venous return
from splanchnic and lower caval regions during the unhepatic
phase.A Y-shaped cavo-renal venous graft (the infrahepatic inferior
vena cava (IVC) till iliac bifurcation and the left renal vein (LRV) till
renal pelvis) was harvested from a donor rat.The polyethylene cuffs
were applied to the proximal ends of infrahepatic IVC and LRV of
the venous graft.In totally hepatectomised rat, porto-renal and lower
cavo-caval anastomoses were performed by cuff technique, and the
upper cavo-caval anastomose by running sutures.This one-stage
procedure, with a mean operating time of 40-2-_5 minutes was not
associated with operative mortality and the portal clamping time did
not exceed 15 minutes.Spontaneous mean survival of the unhepatic
rats was 360-230 minutes, and glucose supplemented animals had a
mean survival time of 20-t-5 hours.The anhepatic state was
associated with significant metabolic and biochemical alterations
(rapid decrease of glycemia and increases of SGOT, SGPT, LDH,
NH3).This practical and time-saving procedure does not require
considerable microsurgical expertize and utilizes a natural vein graft
with neither porto-caval shunt nor artificial vascular prothesis.The
model is particularly useful for metabolic studies in unhepatic rats.
TIPS AS A SALVAGE TREATMENT FOR UNCONTROLLED
VARICEAL BLEEDING IN CIRRHOTIC PATIENTS.
D. Azoulay, J. Raccuia, D. Castaing, H. Bismuth.
Hepatobiliary Surgery and Liver Transplantation Center, Paul
Brousse Hospital, 94800 Villejuif, France
Patients who continue to bleed despite standart treatment including
sclerotherapy have a poor prognosis with a mortality rate of up to
90%. TIPS has been used as salvage therapy ,for ruptured
oesophageal varices refractory to all conventional treatment. During
a period of 3 years, 65 cases of variceal rupture in cirrhotic patients
were treated at our center and a salvage TIPS was performed in 15
patients (23%) for active uncontrolled haemorrhage despite standart
medical and endoscopic treatment (Child A, 2; B, 1; C, 12). The
procedure was technically successful in all cases and haemorrhage
was controlled in 11/15 cases (73%). Three patients died of
persistant bleeding and liver failure; one case of moderate and
persistant haemorrhage was controlled by transfusions until bleeding
ceased. This patient was transplanted 3 months after TIPS and is
:alive 3.5 years later. Two patients had early recurrence of
haemorrhage due to TIPS thrombosis. These 2 cases of thrombosis
were desobstructed but both patients died of liver failure despite
bleeding control. Overall, 7 patients died within 60 days of TIPS by
haemorrhage and/or liver failure. One patient died of liver failure 7
months after TIPS following surgery for aortic aneuvrism. None of
the 8 survivors after 60 days had bleeding recurrence or
encephalopathy. Actuarial survival was 42.7 + 14% at and 2
years. TIPS is currently the alternative of choice for pesistant
bleeding refractory to standart management. However despite
control of haemorrhage, operative mortality remains high due to the
underlying severe cirrhosis.
P015 P016
VALUE OF THE TRANSJUGULAR ROUTE FOR EARLY LIVER GRAFT
BIOPSY (TJLB)
D. AZOULAy, J. RACCUlA, B. ROCHE,, H. BISMUTH
Hepato-Biliary Surgery and Liver Transplant Center, Paul Brousse
Hospital, Villejuif, France
Whereas liver graft biopsy remains the gold standart for the diagnosis of
acute rejection, conventional percutaneous liver graft biopsy in the early
period following liver transplantation (LT) may be impossible due to
coagulopathy and/or ascites. The feasibility of performing transjugular
liver graft biopsy (TJLB) in this setting has been evaluated. From 1991 to
1995, 124 TJLB were performed when biopsy was necessary within 30
days after LT (mean=10, range 4 to 27 days). Indications for this
technique included at least one of the following: thrombocytopenia
(<70.000), prothrombin time <60% of normal and clinical ascites. The 124
TJLB included 106 (85%) procedures without the native inferior vena
cava (IVC) and 18 (15%) with the native IVC intact after LT. There were
109 (88%) TJLB with sufficient biopsy material for definitive diagnosis, of
which 97/106 (92%) did not have preservation of the IVC versus those
11/18 (61%) with the native IVC intact (p<0.05). There was no associated
morbidity or mortality from the procedure. A definitive diagnosis of acute
rejection was obtained with 68 (62%) biopsies, diagnosis of functional
cholestasis with another 25 (23%).and 16 (15%) various other diagnoses.
TJLB was a contributing factor in ascertaining the diagnosis of acute
rejection with 46/68 (68%) patients with rejection, and with 2 of these
patients TJLB was used as the main criteria for emergency re.-
transplantation for hyperacute rejection. The treatment was unaffected
by biopsy results in 20/68 cases of mild. or moderate rejection. In the
remaining 41 patients, the biopsy aided in altering therapy for 4 (10%), of
which 2 had CMV hepatitis and 2 had emergency re-transplantation for
liver graft steatosis and necrosis. In conclusion, early TJLB is feasible and
safe in the early period after LT and it is easier to perform after the native
retrohepatic IVC has been removed. TJLB impacts both diagnosis and
management by alleviating inadequate and potentially deleterious anti-
rejection therapy and aids modification of therapy when rejection has not
been confirmed.
LIVER RESECTION IN THE ELDERLY PATIENT
JM Badia G Nawfal, G Zografos, G Dalla Serra, S Uemoto,
qA Habib.
Department of Surgery, Hammersmith Hospital, London, UK.
The outcome of liver resection in patients over 75 years, in a seres
of 76 consecutive elective resections for benign and malignant
hepatic tumours is analysed.
Aims. To .analyse the effectiveness of hepatic resection in the elderly
compared with the general population.
Methods. During a period of two years 76 consecutive elective
hepatic resections for tumours were performed at the Hammersmith
Hospital. The study included 11 patients the elderly group (EG;
age > 75 years) and 65 patients in the younger group (YG; age < 75
years). In all cases the liver resection was performed with the scalpel,
whereas 72 % of cases were done under total vascular exclusion.
Data was collected prospectively. The blood loss, amount of blood
products transfusion, morbidity and mortality were recorded. The
preoperative clinical features (cirrhosis, Child's classification and
liver function) were comparable in the two groups. The ehi-square
and Student-Fisher tests were used as appropriate.
Results. In 72 % a major surgical procedure was performed. Time of
total vascular exclusion of the liver was 29 +/- 2.8 minutes in the EG
vs 31.6 +/- 13.2 in the YG (n.s). Intraoperative blood loss was 438 +/-
507 mL in the EG vs 1770 +/- 199 mL in the YG (n.s.).. Total
operative time was 5.2 :e 0.5 h in the EG vs 6.3 +/- 2.1 h in the YG
(n.s.). There were no statistical differences in morbidity and
mortality between the two groups. Major complications developed in
4 elderly patients (36.4 %) and 22 younger patients (33.8 %). Three
patients died during the first 30 days after surgery (3.9 %), one in
the EG and two in the YG. There were 2 late deaths (2.6 %) during
the hospital stay, one in each group.
Conclusions. The outcome of liver resection in the elderly group was
satisfactory when compared with that in the younger group.
74
PO17 PO18
HEMOBILIA AFFER LIVER TRANSPLANTATION
Antonio Barrasa M.D. Javier Nulio M.D. Emih'o Vieente M.D. Rafad Barcena
M.D. Antonio L. Sanrronn MD. Adolfo L. Buenadicha M.D. Miguel Garcia
M.D.
Liv Transplantation Unit. Hospital Ramtn y Cajal. Madrid. Spain
Hemobilia is an uncommon but serious complication in patients undergoing
liver transplant. Its clasical form of presentation is jaundice, upper right abdominal
pain and gastrointestinal bleeding but in transplanted patients can show other
clinical presentations as recurrent cholangitis, masive haemorrhage or liver function
tests alteration.
Case 1: A 52 years old man transplanted for C virus chirrosis. On 8th
posttransplant month presented hepatic abscesses located in left hepatic lobe that
were treated with percutaneous drainage. A month later showed fever, jaundice,
liver function tests alteration, melena and progressive anemization. Ultrasound
doppler evidenced abscesses in left hepatic lobe and a cystic lesion with pulse flow.
CT scan and arteriography demonstrated a pseudoaneurism in left hepatic lobe. The
patient under,vent a left hepatectomy and is now asimptomatic 13 months after
transplantation.
Case 2: A 65 years old man that underwent liver transplantation for virus C
chirrosis. The day after transplantation presented elevated level of serum bilirrubin,
alkaline phos'phatase and gamma glutamyl transpeptidase. The prothrombine time
was normal. A Doppler flow study did not find any abnormality but
cholangiography through the T tube showed intrabiliary clotting. An attempt of
desobstruction with intrabiliary injection of urokinase failed. Then a biliary lavage
and hepaticoyeyunostomy was performed improving the liver function. The patient
had an uneventful postoperative course and continues to do well year after liver
transplantation, with slight abnormalities in liver function tests.
Hemobilia is often associated to hepatic trauma or liver biopsy in more
than a half of the cases. The most common pathologic alterations found are
pseudoaneurisms and arteriovenous or ..arteriobiliary fistula. Biliary hemorrhage can
be observed in transplanted patients with severe coagulopathy due to primary non
function or severe ischemic graft damage. The treatment of hemobilia in liver
allografts dds on the cause of the hemorrhage, location of the bleeding source
and time of diagnosis. Biliary lavage can be useful when hemobilia is discovered in
the postoperative period after liver transplantation. For peripheral and well
localized lesions liver resection is the prefered treatment. Finally, the arterial
embolization can be a valuable form of therapy for lesions with central location,
where resection is difficult or impossible. In these cases retransplantion should be
contempled.
CAROLI'S DISEASE. THE EVOLUTION OF THE SURGICAL
MANAGEMENT
Antonio Barrasa M.D. Emilio Vicente M.D. Javi Nufio M.D. Victor S.
Turritn M.D. Luis G. Alvira M.D. Manuel Devesa M.D. Adolfo L.
Buenadicha M.D. Fernando Pereira M.D.
Liv Transplantation Unit. Hospital Ramtn y Cajal. Ciinica Puetta de
Madrid. Spain
Caroli's disease in an uncommon malformation characterized by multifocal
cystic dilatation of intrahepatic biliary ducts. Since the first clinical description by
Caroli in 1958, fewer than 250 cases have been reported and of these 20% were
monolobar.
Between 1980 and 1995,. ten patients with Caroli's disease were treated.
The diagnosis was confirmed by ultrasound and CT scan. Endoscopic retrograde
cholangiography and percutaneous transhepatic cholangiography were used to
delineate the biliary affectation. Liver biopsy was available in five patients
Five patients of Caroli's disease were confined to the left lobe. Four
patients had associated hepatic fibrosis with portal hypertension. Other associated
lesions were; hepatolithiasis (5 patients), and choledochal cyst (1 patient).
The five patients with monolobar affectation underwent liver resection; left
hepatectomy (4) and left sectorectomy (1). Hepaticojejunostomy was carried out in
three patients with diffuse affectation, all performed before 1990. patient required
liver transplantation. Five of the six patients who required partial hepatectomy or
total liver resection with liver transplantation are alive. patient died of secondary
biliary cirrhosis two years after left hepatectomy. Two of the three patients who
underwent biliary derivation died of sepsis and renal amylodosis, 5 and 2 years
after surgery, respectively. patient died of biliary sepsis after endoscopic
retrograde cholangiography before the surgical treatment.
We conclude that partial liver resection shoul be considered as the
treatment of choice in patients with monolobar Caroli's disease. Liver
transplantation must be a safe surgical procedure that could be particularly indicated
in symptomatic patients with bilobar affectation, specially when hepatic fibrosis and
portal hypertension is present. Hepaticojejunal anastomosis is the therapeutic
solution every time hepatic resection cannot be performed
P019 P020
DE NOVO HBV AND HCV INFECTIONS AFTER LIVER
TRANSPLANTATION
R.Bellusci, E.De Raffele, R.Miniero*, E.Lucci, B.Santoni, S.Galli*, R.Lucchetti*, F.Fruet^,
R.Conte^, G.Sprovied*, A.Mazziot'd, A.Cavallad. Clinica Chirurgica II, *Laboratodo
Centralizzato and ^Servizio Trasfusionale, Policlinico S. Orsola, Bologna. ITALY.
INTRODUCTION, HBV and HCV infections often recur after liver transplantation (OLT).
However, the incidence, timing and the clinical behaviour of infections acquired with OLT
have not been widely investigated. Aim of the study was to evaluate the incidence of de
novo HBV and HCV infections in a group of liver transplant recipients with long-term
follow-up, .their biochemical and 61inial evolution, the usefulness of serological assays
for diagnosis. The virological and clinical course of patients with HBV-HCV coinfection
was also investigated. PATIENTS AND METHODS. One hundred twenty-one patients
transplanted at our institution between 1986 and 1994, with a follow-up _> 6 months,
entered the study. HBV and HDV serological patterns were determined using
commercial tests. For diagnosis of HCV infection, anti-HCV were evaluated with an
ELISA and a RIBA assay. HCV-RNA was detected by PCR. RESULTS. Three patients
became HBsAg positive after OLT. All of them showed signs of viral replication (HBeAg
and HBV-DNA reactive), but ALT levels raised only in one case. Twelve patients became
anti-HCV positive at different time intervals from OLT. Passively acquired anti-HCV
appeared in all cases within few days from OLT. After clearance of passive antibodies,
active anti-HCV seroconversion was usually delayed, with the new generation RIBA
assay showing better results than the RIBA assay. Three patients were repeatedly
HCV-RNA negative despite persistent anti-HCV reactivity: all of them were HBsAg
positive pre- and post-OLT. The viral genome was detected in 9 patients, even though
most of them were not persistently HCV-RNA positive dudng their follow-up. Four pre-
OLT HBsAg positive patients were found HBsAg positive after transplant. The remaining
8/12 patients, including 3 cases HBsAg positive pre-OLT who cleared after OLT,
experienced repeated ALT increases >2xN, after 138,8 +_ 82,0 p.o. days (range: 26-234):
ALT levels spontaneosly returned to normal values within 6 months in one case, while
remained >2xN longer than 6 months in the others. CONCLUSIONS. De novo infections
due to pdmary hepatotropic viruses were frequently observed after OLT. Eady diagnosis
of viral infection, in particular when the HCV is involved, may be problematic in these
cases and should be taken into account when persistent abnormalities of ALT levels are
observed. Monitoring of sedc viral markers and accurate evaluation of biopsy specimen
is manclatow. The interference between HBV and HCV might play a role in the
replicative cycle of one or both viruses in coinfected patients. Accurate anti-HCV
screening of blood and organ donors should reduce in the future the incidence of de
novo viral infections after OLT.
75
INVASIVE dETHODS OF EXAMINATION OF PATIENTS
WITH OPISTHORCHIASIS
E, I..Be1oborq..0xa,.A. Tun, E. V. Be1oborodora
Siberian Niedical University,Tomsk,Russia
The Ob-Irtishsky region of Opisthorchiasis
is the biggest in the world. The morphologi-
cal picture of the liver has been studied
only in experimental animals and the data of
pathologists, lOt patients with chronic opis-
thorchiasis accompanied by the liver enlarge-
ment aged between 18 and 66 years were exa-
mined.The duration of opisthorchiasis inva-
sion in half the cases was more than 10 years
Invasive methods of examination (laparoscopy
with targeted biopsy of the liver in 6 cases,
"blind" transcutaneous biopsy of the liver
in 30 pts) were performed following a thoro-
ugh clinical examination and biochemical,im-
munological, ultrasonic, radioisotopic me-
thods. At laparoscopy liver enlargement,red-
dish colouring o the organ and hardening of
the consistency were revealed in all cases.
Two patients had linear thickening of the li-
ver capsule and single cholangioectasis.Li-
ver-gallbladder adhesions were found in 2 ot-
her pts. orphological examination of the
liver was carried out in 36 cases.Most re-
quently we observed chronic persistent hepa-
titis-25 pts (69,4%),lobular hepatitis- 8
pts (22,2%),chronic active hepatitis-3pts
(8,4%).The character of the liver lesion de-
pended on the duration and intensity of in-
vasion,presence of additional hepatotropic
factors, patient s immunity. The peculiarity
of the liver lesion in the infection is mor-
phological igns of cholangitisaud c holestasis
P021 P022
COMPARISON OF A TISSUE OXYGEN ELECTRODE WITH LASER
DOPPLER FLOWMETER FOR ASSESSING ORGAN PERFUSION:
RESULTS IN AN ANIMALMODEL
E Bennett D Cox, V Chidambaram, A Seifalian, B R Davidson
Royal Free Hospital and School of Medicine London, UK
Accurate information on organ perfusion is essential for assessment of organ
function following transplantation. A parenchymal oxygen electrode (Clark
electrode) has not previously been compared with laser doppler flowmeter
(LDF).
Method: 4 anaesthetized ventilated pigs had a Clark electrode inserted into the
liver parenchyma and 2 surface LDF probes placed on the liver soon after
opening the abdomen. Ligatures were placed around the hepatic artery and
portal vein. Sequential readings of the Clark electrode (pO2), and LDF signal
were taken occlusion of hepatic artery (OHA), portal vein (OPV), and both
vessels (OALL). The data for LDF and the Clark electrode are expressed as a
% of the mean before clamping +/- SE, and analyzed using a paired Student's
T Test.
Results: The Clark electrode value on OHA, OPV, and OALL were
respectively: 75.9% +/- 16.9(P< 0.03),57.2% +/- 10.4(P< 0.001)and 55.6%
+/- 8.82 (P< 0.001). The LDF results on OHA, OPV, and OALL were
respectively: 73.2% +/- 41 (P< 0.15),65.5% +/- 27.4(P< 0.02),and 50.1%
+/-42 (P< 0.004).
Conclusion: Following OHA the pO fell significantly, and continued on OPV
and OALL. In contrast the LDF showed no significant fall on OHA, but
sighificantly fell on OPV and OALL. The Clark electrode may be a useful
method of assessing liver perfusion and reflects flow measurements on laser
doppler.
INTROFON AS A METHOD OF CAVITY MANAGEMF./CT IN
SURGERY FOR HYDATID DISEASE
O.Bilge, .Emre |.(zden, Y.Tekant, K.Acarh, A.Alper, O.Ano,ul
Hepatopancreatobiliary Surgery Unit, University oflstanbul, Istanbul
Faculty ofMedicine, lstanbnl, Turkey
Introflection is a method used for the residual cavity management in
surgery for hepatic hydatidosis. The experience of our unit .was
reviewed to evaluate the safety and efficacy ofthis method.
Introflection was used in 162 of 495 hepatic hydatidosis patients
operated in our unit between 1978 and 1995. Eighty-two patients
were female and 80 were male with an average age of38 years (7-71
years). Sixty-two percent of the patients had solitary cysts, while 18%
had two, and 20% three or more cysts: The cysts were located in the
right lobe of the liver in 59% of the patients, in the left lobe in 19%
and were bilobar in 22%. Lesion size ranged from 3 to 30 cm. Biliary
communications were found in 47 patients and common bile duct
exploration was performed in 27 of them. T-drainage was performed
in 10 patients, choledochoduodenostomy in 11, primary closure in
five and sphincteroplasty in one. In the remaining 20 patients, biliary
communications were sutured only.
One patient was reexplored for intmabdominal bleeding and died on
the second postoperative day (0.6 %). B""!Hazy fismlm developed in
fivepatients (3.7 %), of whom three dosed spontaneously. The other
two patients were successfully treated by endoscopic papillotomy.
Abcess formation in the cavity occurred in.two patients (1.2 %) and
was treated surgically. Six patients (3.7 %) had wotmd infectio and
six (3.7 % had pulmonary complications, Average hospital stay was
11 days (4-35). Four recurrences (10.2 %) were observed among the
39 patients whom were followed for a"median period of 36 months
(7-132 months).
In conclusion, introflection is an .efficient cavitymanagement method
in hepatic hydatidosis surgery.
P023 P024
INTRA-OPERATIVE ULTRASONOGRAPHY DURING SUR-
GERY FOR MALIGNANT TUMORS OF THE LIVER
Boldrini G., Giovannini I., De Gaetano A.M.,
Vellone M., Nuzzo G.
Depts of Surgery (Chirurgia Geriatrica) and
Radiology, Catholic University, Rome,Italy.
Intra-operative ultrasonography (IOUS) of
the liver was carried out in 68 pts under-
going surgery for malignant hepatic tumors
(11 hepatocellular carcinomas in cirrhotic
pts and 57 metastases from digestive tu-
mors), to implement the information obtained
in pre-operative investigations and to guide
the surgical procedure. The liver was scan-
ned at the beginning of the procedure, after
complete division of hepatic legaments, with
a 7,5 MHz probe. In 11 pts the examination
disclosed pre-operatively undetected liver
tumors. In 27 pts neoplastic lesions were
more accurately located in the hepatic seg-
ments,, modifying the evidences of pre-
operative imaging techniques. In 21 pts fur-
ther intra-parenchimal lesions of benign na-
ture (12 simple cysts, 5 hemangiomas, 3 li-
poid skip areas, necrotic nodule) were de-
tected and localized. Dimensions of lesions
recognized with IOUS ranged between 2 and 24
mm in diameter. In 9 pts IOUS determined re-
levant changes in the planned surgical pro-
cedure: 6 of these pts were submitted to mo-
re extensive resections. IOUS showed high
sensitivity in detection of liver neoplasms,
allowed accurate definition of the lesions
and of their relationships with vascolo-
ductal structures, thus contributing signi-
ficantly to the success of surgical resec-
tion.
76
COAGULATION DISORDERS AFTER ISOLATED HYPERTHERMIC LIVER
PERFUSlON WITH MITOMYCIN C
A. Bomscheuer1, K.-H. Mahr1, K. Oldhafer2, H. Lang2, S. Nadalin2, M. H61tje M.
Szabo ,R. Goldmann
1Zentrum An&sthesie, Abt.I, 2Klinik for Abdominal- und Transplantationschirurgie,
Medizinische Hochschule Hannover
Systemic toxicity is limiting cancer chemotherapy. The isolated hyperthermic liver
perfusion with Mitomycin C is therefore an alternative strategy in the treatment of li-
ver metastasis(I). From February 1995 to September 1995 6 patients were selected
who had irresectable hepatic metastases. Informed consent was obtained from all
patients. After isolation the liver was perfused for 60 minutes with a 41 C heated Mi-
tomycin C-Solution (20mg Mitomycin C/m2BS). A veno-venous bypass prevented
haemodynamic disturbances during anhepatic period. Use of heparine, anhepatic
phase and Mitomycic C therapy can be the cause of coagulation disorders during
this proceeding. We evaluated coagulation parameters at the beginning of sur-
gery(a),lO min before(b), 10 min after(c) starting mitomycin perfusion, 10 min be-
fore(d) end of perfusion, lO(e) and 60min(f) after perfusion and h after arriving on
ICU.
Quick FrI-T Fibrinogen Platelets AT III (%) F (%) F V (%)
(%) (sec) (g/l) (Tsd/ul)
79+7,9 41+5,4 3+1,4 175+56,8 82,8+7,3 94+/-9,9 93+/-14
72+/-15t3 39+517 2,7+/-11 169+/-54,3 6514+/-10,2 76+/-14 79+/-18
11+/-111 197+/-718 2+/-0,7 152+/-49,8 57,7+/-10,1 57+/-16 32+/-17
12+/-152 196+/-815 2,1+/-017 187+/-46,6 59,2+/-12,8 62+/-18 44+/-13
24+/-13,8 181+40 211+/-1,1 160+/-56,3 65+/-5,2 55+/-18 36+/-13
39+/-22,6 137+83 2,2+0,1 144+/-80,8 62+/-2,6 69+93 39+/-13
38+/-24,4 117+/-66 2,2+/-0,5 108+/-80,2 64,5+/-5,1 71+/-10 40+/-14
All patients got 4-6 units fresh frozen plasma. Protamine was given in one case to
neutralize heparine.ln all other cases PTT and Quick -test did not return to normal
during observation period. Signs of intravascular coagulation (Platelets:27Tsd/ul;
Fibrinogen: O,lg/I; Quick:4%) without severe bleeding was seen in one patient after
reperfusion. Substitution with fresh frozen plasma returned coagulation to normal in
this patient. The course of the other patients was not complicated by coagulation
disorders. Thus hyperthermia in combination with Mitomycin C seems not to induce
severe coagulation disorders.
Lit.: l.:Marinelli A.,et all. A comparative study of isolated liver perfusion versus
hepatic artery infusion with mitomycin C in rats. Br.J.Cancer (1990),62,891-896
P025 P026
EXPERIENCE OF 100 EMBOLIZATIONS OF SPLENIC
ARTERY IN LIVER CIRRHOSIS AND PORTAL
HYPERTENSION
K. Boulanov
Institute of Clinical and Experimental Surgery, Kiev, Ukraine
The hyperdynamic state of splenic flow is considered to be
responsible for the development of portal hypertension in liver
cirrhosis. The aim of the study was to evaluate results of 100
embolizations of splenic artery (ESA) in 92 cirrhotic patients
(Child-Pugh A-20, B-37, C-35). All of them had symptomatic
hypersplenism, 86 had esophageal varices, 32 had previous
hemorrhage, 67 had refractory ascites, 28 had episodes of
encephalopathy. ESA consisted in simultaneous insertion of
steel coil and 10-15 synthetic emboli. Hemodynamics was
measured using duplex system and direct portomanometry.
Patient follow-up ranged from 6 to 60 months. Remote results
were considered to be satisfactory in the absence of esophageal
rebleedings and encephalopathy, complete absorption or
improvement of ascites and correction of hypersplenism. Early
correction of hypersplenism was universal. Splenic infarction
developed in 45 patients but resolved within 2 weeks.
Splenectomy for subtotal abscess was performed twice. There
was no mortality. Within 3 months ESA was repeated in 8
cases for recurrence of hypersplenism due to arterial
recanalization. Early twofold reduction of splenic flow resulted
in the decrease of portal flow and portal pressure to
about 75% of pre-existing values, and increase of hepatic
arterial flow to 50%. Results of remote investigations were
similar to postoperative data. During the follow-up 12 patients
died of liver failure. On the whole, satisfactory results were
obtained in 65% of patients. Thus, ESA is effective mini-
invasive procedure, that shows favourable influence on the
course of liver cirrhosis and portal hypertension.
SURGICAL TREATMENT OF NON-COLORECTAL HEPATIC
METASTASES: LONG-TERM RESULTS
Bouzari H., Polastri R., Calgaro M., Capussotti L.
Department ofSurgery, Ospedale Mauriziano- Torino, Italy.
We have rewieved our experience with hepatic resecticm for non-
colorectal and non-nenroendocrine hepatic metastas in ordex to evaluate
short- and long-term survival. From January 1984 to December 1994 we
performed 27 hepatic resections in 24 patients. Sixteen were female and 8
males with a mean age of 57 years (range: 28-73). The sites of primary
tumor were: the breast (4), the stomach (3), the exoerine pancreas (4), the
leiomyosareoma of intestine (3), unknow primary (2), the kidney (2), the
papilla ofVater (2), the uterus (1), the adrenal (1), the ovary (1) and the
small bowel (1). There were 11 synchronous and 13 metachronous lesions.
Resection was curative in 71% ofeases. The operative procedure consisted
of 12 major hepatectomies, 8 mono- or bisegmentectomies and 7 non-
anatomical resections. Twelve patients (50%) were not uansfused. No
operative mortality (60 days) occurred. Morbidity was seen in 5 cases
(18.5%): pneumonia (3), subphrenic collection (1) and pancreatic fistula
(1). The mean postoperative hospital stay was 14 days (range:8-33).
Actuarial survival rate (Kaplan-Meier method) at 1,3 and 5 years was
65%, 28% and 18.7% respectively. Five patients are alive and tumor free
at 10,11,16,19 and 70 months from operation.
The mean of survival of patients with non-resected hepatic metastast
from non-eolorectal, non-neuroendoerine tumors is less than 8 months.
However, because most patients die from the disease with or without
surgical treatment, the goal ofresection should be clearly defined, whether
palliating specific symptoms or attempting to lenghtea the survival time. In
conclusion: an aggressive surgical approach in the treatment ofmetastatis
disease to the liver offers a chance for long-term survival and significant
palliation in selected patients on condition that hepatic resection can be
performed with a reasonable risk.
P027 P028
FACTORS AFFECTING LONG-TERIVl OUTCOME AFTER
HEPATIC RESECTION FOR HEPATOCELLULAR
CARCINOMA IN CIRRHOTIC PATIENTS.
H. Bouzafi, V. Vergara, M.M. Marueei, L. Capussotti.
Department ofSurgery, Ospedale Mauriziano- Torino, Italy.
The aim ofthis study was to identify the preoperative variables correlated
to postoperative long-term survival in 125 cirrhoties who underwent
hepatic resection for hepatocellular carcinoma (HCC) between January
1984 and December 1994. There were 20 women and 105 men. Mean age
was 63 years (range: 26-78). 90 patients belonged to Pngh's A class and
39 to classes B-C. 129 hepatic resections were carried out on 125 patients
(4 were reseeted twice). 22 were major hepatic resections, 86 mono- or
bisegmentectomies and 21 were non-anatomical resections. Survival was
calculated using the method of Kaplan-Meier. Survival estimates were
compared using the log-rank test. Survival estimates for the following
variables were calculated: sex, age, ethiology of the cirrhosis, symptoms,
esophageal varices, ascites, Pugh's class, macroscopic growth pattern
(solitary versus multiple), site ofHCC, red blood cells, white blood cells,
platelets, bilirubin serum level, AST, ALT, GGT, ALP, prothrombin time,
U and V coagulation factors, albumin, calcium, CEA and ct-fetoprotein
serum level, Hepatitis B surface antigen and antibody, Hepatitis C surface
antibody. Cox's regression model was used to determine the significance
ofthe variables in a multivariate analysis. Operative mortality (60 days)
was 6.9% (9 patients). Morbidity was 24% (32 patients). Actuarial
survival rate at 1, 3 and 5 years was 72.5/0, 47% and 34.2% respectively.
In this series, Pugh's class, age, ct-fetoprotein serum level, Hepatitis B
serum antigen and prothrombin time was found to be the single most
important predictor of survival. In a multivariate analysis model the
significant factor affecting survival were: Pugh's class, age, site of HCC,
AST, ALT, GGT, ct-fetoprotein serum level and HCV.
One third of patients reseeted for HCC can be expected to survive long-
tenn. Pugh's class and ct-fetoprotein serum level predicted long-term
outcome.
EXCISIONAL TREATMENT OF LIVER CAVERNOUS HEWANGIOMAS"
WHEN AND WHICH?.
R.BRACCO (F.A.C.S.), J.FRARACClO (F.A.C.S.)J.GRONDONA
"(F.A-[C-.-S.).SERVICE OF SURGERY,CLINICA PUEYRREDON,
MAR DEL PLATA,ARGENTINA.
While the role of surgical treatment of cavernous hem-
angiomas remains the subject of debate,current indica-
tions for surgical resection include rapid enlargement,
abdominal pain,potential for traumatic rupture and un-
certainty of diagnosis.ln a 6 year period we have trea
ted 21 patients carrying these tumors;the female/male-
ratio was 3"1 and the mean age 54 years old.The diagno
sis of cavernous hemangioma was retained if the tumor
demonstrated at least in one of the radiological exami
nations :ul trasonography(US) ,computed tomography(CT) ,a
giography and magnetic resonance imaging(MRl)and were-
not modified in terms of size,structure or number at
the 6 and 12 months US follow-up evaluation or at micro
scopic examination of the resected specimen.Fo urteen
patients were not operated on and had a mean diameter
of 4.7cm(2.5 to 6 cm).Seven patients with8 hemangiomas
were resected,the mean diameter was 12.9cm(7 a 22 cm)
and fullfilled criteria for surgical intervention. The
30-day mortality was O%.AII patients remain symptoms
free and without pathological findings at US yearly exa
mination.
Conclusions l)Most patients with liver cavernous heman-
iomas are asymptomatic and do not require surgical tre
atment.2)Excisional treatment is indicated when there-
is'persistent abdominal pain,exposure for potential tra
umatic rupture or uncetainty of diagnosis.3)Enucleati
when possible, is a safe alternative to resection for
treatment of these tumors.
77
P029 P030
SPECIAL TROCAR AND NEGATIVE PRESSURE
SUCTION APPARATUS IN THE SURGICAL
TREATMENT OF LIVER HYDATIC CYST.
T. Butr6n, S. Mallagray, MJ. Castillo, A. Garcia.
Department of Surgery. Cruz Roja Hospital, Doce de
Octubre University Hospital, Madrid, Spain.
One of the problems in the surgical treatment of huge
hydatic cysts is the possibiliW of spillage of hydatic
membranes and liquid to the abdominal cavity. We propose
the use of a great trocar attached to a negative pressure
suction apparatus to avoid this problem.
MATERIAL AND METHODS. Supported by the results of
a prospective study of 40 patients operated on by a
thoracophrenolaparotomy by superior edge of ten rib
(TPL10) and an open total or partial pericystectomy, we
show in the pictures a modified trocar ofDermileau attached
to a negative pressure suction apparatus that allowed a
sudden aspiration ofthe cyst.
RESULTS. A total of 65 cysts from 10 to 30 centimeters of
size were present in the 40 patients. All cysts were aspirated
with the negative pressure suction apparatus in.few seconds.
There were not spillage ofthe hydatic membranes and liquid
in any case. There were not morbidity due to the use of the
negative pressure suction apparatus. The six years follow-up
was normal in all cases.
CONCLUSIONS. The use of the trocar attached to the
negative pressure suction apparatus seem to be a effecaive
procedure to avoid spillage ofhydatie cyst
TOWARD SIMPLIFICATION OF SURGICAL MANAGEMENT
OF HYDATID DISEASE OF THE LIVER. RESULTS OF A
MONOCENTRIC COMPARATIVE STUDY.
P, Cam_Dan, V. Bellot, M. Chabaud, B. Pol, Y.P. Le Treut. Service
de Chirurgie Gn,rale, H6pital de La Conception- 147, Bd Baille
13385 Marseille Cedex 5 FRANCE.
The authors compared two series of patients operated for hydatid
cyst of the liver in the same surgical department: 48 patients treated
between 1980 and 1985 (group 1) and 38 treated between 1990 and
1995 (group 2). Main differences were pointed out as follows:Fifty
per cent of the patients of group were from northern africa, versus
31% in group 2 (p < 0,1). Arteriography was performed
preoperatively in 73 % of the patients of group 1, versus 13 % in
group 2 (p < 0,001). Thoraco abdominal incision was used in 31%
of the patients of group 1, whereas in none of group. 2 (p < 0,001).
lntra operative cholangiography was a routine investigation in group
1, and was only performed in 32% of the patients of group 2 (p <
0,001). Total cystopericystectomy was attempted in 33% of the
patients of group 1, versus 53% in group 2 (p < 0,1), including six
formal liver resections. External T-tube biliary drainage was placed
in 85% of the patients of group 1, versus 32% in group 2 (p <
0,001). Duration of post operative hospital stay was 29 +/- 12 days
in group 1, versus 17 +/- 11 days in group 2 (p < 0,001).These
results suggest that total cystopericystectomy through abdominal
approach, without routine drainage of the main biliary duct,
improves the outcome of surgery for hydatid disease of the liver.
P031 P032
THE SENSITIVITY TO ANOXIC INJURY IS INCREASED IN
HEPATOCYTES ISOLATED FROM RAT FATTY LIVER.
P. Caraceni, A. Gasbarrini, B. Nardo, S. De Notariis,F. Trevisani, G.
Gasbardni, A. Mazziotti, A. Cavallad, DH. Van Thiel, M. Bemardi.
Patologia Medica e Clinica Chirurgica II, Universita' di Bologna; Clinica
Medica II, Universita' Cattolica del Sacro Cuore, Roma; and Oklahoma
Medical Research Foundation, Oklahoma City (USA).
Livers with steatosis moderate to severe are usually refused as
donor organs for orthotopic liver transplantation (OLT) because their use
is a major cause of primary non-function (PNF). Clinical observations
suggest that the tolerance of steatotic livers to ischemia-reperfusion injury
is reduced. Thus, the aim of this study was to determine whether rat
hepatocytes isolated from fatty or non-fatty livers have a different
sensitivity to anoxia-reoxygenation injury.
Rats were divided into two groups and fed modified ethanol/liquid
and control/liquid Lieber-De Carli diets for 8 weeks. Light microscopy
showed that >85% of the hepatocytes from alcohol-fed rats contained
fatty vacuoles. Isolated hepatocytes were cast in agarose gel threads and
exposed to: a) continuous aerobic perfusion, or b) continuous anoxia, or
c) 2 h anoxia followed by h reoxygenation. Cell injury was assessed by
LDH release and cell viability by trypan blue (TB) exclusion. O2" formation
was detected by lucigenin-enhanced chemiluminescence (LCL).
No differences were observed during the control period: LDH
release was 25+2 and 27+4 mU/min, viability was 85+1 and 86+1%, LCL
was 4+1 and 5+1 nA in the fatty and non-fatty hepatocyte groups,
respectively. These parameters remained stable for 3 h of aerobic
perfusion in either group. In contrast, fatty hepatocytes died much faster
than control hepatocytes when exposed to anoxia: after 3 h of anaerobic
perfusion viability was 17_+11 vs 60-+4% (p<0.001) in the fatty and non-
fatty hepatocyte groups, respectively. After reoxygenation following 2 h of
anoxia, the increase in LCL was similar in the two groups (65+7 vs 78_+11
nA). Concomitantly, LDH release and TB exclusion did not differ during
reoxygenation between the two groups.
These results suggest that in isolated rat hepatocytes fatty
infiltration:l) does not affect cell injury under aerobic conditions; 2)
increases significantly anoxic injury; and 3) does not appear to influence
O2-generation and cell injury immediately after reoxygenation. The
reduced tolerance to anoxic injury observed in rat hepatocytes with fatty
infiltration can contribute to the high incidence of PNF occurring when
steatotic livers are used as donor organs for OLTx.
Biliary Complications in Liver Transplantation
D Casanova, M G Fleitas, E Martino, L Herrera, F Hernanz
Department of Surgery, University Hospital Valdecilla, University of
Cantabria, Santander, Spain
Although refinements in the surgical and anesthetic techniques ofliver trans-
plantation have helped to improve results, surgical complications remain
common. Complications of the biliary system have been reported to be as
high as 29%:These complications can be severe and lead to sepsis and death.
Aim: To evaluate the biliary complications in 130 consecutive liver trans-
plants. Patients and Methods: 130 OLT were performed in 118 patients (12
retransplantations) between Nov 90 and Nov 95. During OLT, the biliary
reconstruction most commonly used are choledococholedochostomy over a
T-tube in 125 cases, and cholodocoycyunostomy in the other 5 cases. The
cholangiographic control was performed two weeks after the transplant and
the T-tube is removed ten weeks later. Results: Eighteen patients (13.8%)
developed biliary complications: leak (13), stricture (3), and dehiscence and
necrosis ofthe duct in the othertwo cases. Surgical treatment was performed
for leak anastomosis in 7 of 13 cases, in 2 of 3 cases of stricture, and in both
cases ofischemic dehiscence while a retransplantand ahepaticoyeyunostomy.
No deaths were due to the development of biliary complications. Conclu-
sions: We come to the conclusion that biliary complications are the most
frequent surgical complications after OLT and cause of serious morbidity
and morality. The high number ofbile leak associated withT-tube removal is
related to the low inflammatory response around the T-tube as result of
inmunosuppresive agents and theT-tube of silastic. We propose to cover the
silastic T-tube with a conventional T-tube of rubber, in order to increase the
fibrous tract around it. For the stricture complications the endoscopic tech-
niques provide a safe and effective alternative to reoperative surgery for liver
transplant patients who develop biliary tract problems.
78
P033 P034
POLYCYSTIC DISEASE OF THE LIVER
G. Catania. F. Cardi, T. Salanitri, G. A. Petralia, D. Pluchino, V. Altadonna
Department of Surgery, Division of General and Oncological Surgery,
University of Catania, Catania, Italy
A clinical study was carried to evaluate the treatment of patients with
symptomatic polycystic liver disease by percutaneous drainage and
sclerotherapy with alcohol. Ten patients presented with polycystic liver
disease, of which six had polycystic kidneys too. Two patients drop out the
study: one died for renal failure and one did notpresentto the control. Eight
patients are the subject of the study, six female and three male (F:M 3:1),
mean age 50,7 years (range 28-60 years). Five patients of 8 had polycystic
kidneys and two of them had the liver micropolycytic variant. All patients
underwent echographic study.
Patients had bulging upper abdomen and pain in the right hypocondrium
originating perhaps from the stretching of the liver capsule. Only small
portions of healthy liver tissue could be detected by imaging methods. Two
femalepatientscomplainedofsymptomsofgastric compression(vomit) and
dyspnea. In order to ameliorate this symptoms and maintain the hepatic
functionby.reducing the compression of the functioning tissue, in these two
patientsUS-guidedmultiplecystpunctureswereperformedforpercutaneous
drainage and sclerotherapy with alcohol. Following the multi-stage
procedures the size of liver decreased and hepatic function gradually
improved. The other six patients are on echographic follow-up.
Inaccordancewiththeliteraturetheauthorsrecommendthemulti-stagecyst
puncture,and sclerotisatibn as beneficial therapy of symptomatic polycystic
liver disease without burden general anaesthesia, with minor risk and
minimal discomfort for the patients.
LONG-TERM FOLLOW-UP AFTER SURGERY FOR HEPATIC
HYDATID DISEASE
R.Chautems, B.Gold, L.Bhler, E.Giostra,
Ph.Morel, G.Mentha
Digestive Surgery, University..Hospital,
Geneva, Switzerland
On a long term, surgery is the only effective
therapy for the treatment of hepatic hydatid
disease. Between 1980 and 1995, 74 patients
were operated for this affection among 410
liver operations (18%) in our Digestive Sur-
gery Clinic. For these 74 patients (49 women,
29 men, mean age 43 years), 58 total pericyst-
ectomies, 28 subtotal pericystectomies, 28
hepatectomies and 8 unroofing operations of
the cysts were performed. The mean hospital
staywas 15 days (7-69), with a post-operative
morbidity of 22% and no mortality.
A 63 months (5,3 years) mean follow-up was
available for 66 patients (complete 49 (66%)
partial 17 (23%)). Eight patients were lost
for any follow-up. Late complications consis-
ted in two biliary-tract stenoses, one post-
transfusional hepatitis C, one incisional
hernia and a single hydatid recurrence, nessi-
tating a second operation 3 years after a
subtotal pericystectomy. Our follow-up policy
includes a single echography after one year,
if the operation was considered as radical
(total pericystectomy or hepatectomy), every
6 months if a subtotal pericystectomy or an
unroofing operation was performed.
In conclusion, we propose a radical surgery
(total pericystectomy or hepatectomy)when
technical facilities allow it. With this kind
of surgery recurrencies of hydatid disease are
exceptionnal and mortality close to zero.
P035 P036
Synergistic effect of complement activation and leukocyte
InCdtration in ischaemia-reperfusion injury of the liver.
Protective effect of the complement regulator sCRI.
It. Ch/vez-Cartaya, G. Pino-DeSola, S. Metealfe, DJ.G. White
and N. V. Jamieson.
University of Cambridge. Addenbrookes Hospital. Dept. of Surgery.
Cambridge, U.IC
With the purpose of studying the participation of complement and
neutrophils in tissue injury after ischaemia and reperfnsion the
complement cascade was inactivated and leukocytes were depleted in
model ofrat liver ischaemia. Soluble Human Complement Receptor Type
(sCR1XS0 mg/Kg IV) was given after vascular occlusion (n=8),
complement activity (CH50) was reduced to 10% in these rats.
Vinblastine (1.6 mg/K8 im) was administered five and two days before
the experiment to render the rats leukopaenic (<800 WBCYmm3). Non-
ischaemic rats (n=8), ischaemic non-treated rats (n=lS) and vinblastine
trted, non-ischaemic rats (n=3) were used m controls.
This experiment consisted ofthe temporary interruption ofarterial and
portal blood flow to the lef lateral and medial lobes ofthe liver during
45 minutes, followed by reperfnsion. Liver blood flow and haemoglobin
saturation measured by laser Doppler flowmetry and photometry were
recorded for one hour after declamping, with statistically significant
differences between the two experimental groups and the untreated
lschaemic control group (p<0.001).
Histology showed neutrophil infiltration and tissue injury in
the ischaemic control group, but not in the experimental rats. Marked
oedema was present in the leukopaenic rats. Intravascular neutrophils
were present in the sCR1 treated rats, without evidence of
transendothelial migration or tissue injury.
Immunohistochemistry showed dposits of C3 and C9 in the
endothelium ofthe ischaemic controls and of the leukopaenic rats but
not in the sCR1 treated group.
These results suggest that activation of complement alone is
responsible for changes in endothelial permeability in early reperfusion
injury but recruitment, activation and infiltration of neutrophils (due to
complement itself to endothelial activation) resulted in nmjor tissue
injury. The regulation of complement with sCRI offered strong
protection against ischaemia-erfusion injury.
DIAPHRAGMATIC SEEDING AFTER PERCUTANEOUS BIOPSIES OF
LIVER TUMORS
D. Cherqui, L. Salomon, J. Tran Van .lieu, P.L. Fagniez. Departments of
Surgery and Pathology. Universit4 Paris XII-H6pital Henri Mondor,
Cr4tefl, France
Percutaneous biopsies (PCB) of liver tumors are commonly used to obtain
pretreatment histologic diagnosis. Tumor seeding, in the peritoneum or
along the needle tract, is theoretically possible but considered exceptional
and unsignificant. However, the rate of tumor seeding after PCB is
probably underestimated.for several reasons: (a) only apparent lesions are
diagnosed (mainly cutaneous), (b) patients are not specifically screened for
this complication, (c) rapid cancer spread may lead to overlook local
seeding, and (d) a cause to effect relationship may be difficult to establish.
We report 6 cases of diaphragmatic seeding after PBC of a liver tumor. The
tumor was hepatocellular carcinoma in 4 cases and metastasis of colorectal
cancer in 2 cases. Tumors were all located in the right lobe, superficial, and
measured 2-8 cm. Direct fine needle PCB had been perfomed to 9 months
earlier. All patient underwent surgery (3 partial hepatectomies and 3 liver
transplantations). In 5 cases there was a narrow adhesion of the liver to the
diaphragm at the site of biopsy While in the other case there was a mm
granulation on the diaphragm facing the tumor. A patch of diaphragm was
resected in all cases and confirmed tumor involvment of the diaphragm. 3
patients died of recurrence and 3 are alive (2 with with recurrence). During
the same period (1989-1994), 117 patients underwent resection (88) or
transplantation (29) for primary or secondary liver cancer. Diaphragm
seeding was found in 6 of 29 patients (20%) who had undergone
preoperative PCB in contrast with 2 of 88 patients (2.2%) without
preoperative PCB (p<0.01).
These data suggest that diaphragm seeding is a potentially underestimated
complication of PCB of liver tumors. Superficial tumor location could be a
risk factor. Prospective intraoperative screening and resection of suspect
diaphragm lesions during surgery is required to evaluate the prevalence of
post PBC tumor seeding. Since this complication can compromise the
results of surgical treatment, we recommend that PBC should be avoided in
patients with liver tumors who are candidates for liver resection or
transplantation.
79
P037 P038
LIVER RETRIEVAL FOR TRANSPLANTATION:
OPERATIVE PROCEDURE AND THE HEPATIC
MICROCIRCULATION
V. Chidambaram A.M. Seifalian, D.P. Moore, K.K.
Changani, B.J. Fuller, B.R. Davidson.
University Department of Surgery, Royal Free Hospital &
School of Medicine, London U.K.
We studied the surface and parenchymal microcirculation of
porcine liver during the various stages of organ retrieval using
Laser Doppler flowmeter (LDF). Method: In 6 pigs, LDF
surface and parenchymal probes were applied to the left median
lobe of the liver soon after opening the abdomen. The hepatic
microcirculation was pecorded continuously for 90 secs as flux
units before (Premob) and after dissection (Postmob) of its blood
vessels as well as on sequential occlusion of hepatic artery (HA),
portal vein (PV) and both vessels. The data discussed as mean
+ SD was analysed using simple linear regression and paired
students test. Results: The Premob surface and parenchymal
perfusion .of the liver 226 +/- 73 and 254 +/- 82 flux units
respectively showed a significant correlation (r 0.8). Following
hilar dissection the surface perfusion dropped significantly (p
0.050) to 151 + 80 flux units, while the parenchymal flow 229
+/- 128 flux units showed no significant change (p >0.05).
Occlusion of HA, PV and both vessels resulted in significant fall
(p < 0.005) in surface flow to 120 +/- 46, 110 +/- 86, 77 +/- 53
flux units respectively. On the contrary the parenchymal flux
varied widely on occluding HA, PV & both vessels 402 +/-
451, 265 .263 and 249 +/- 169 units respectively and the
changes in flow values were not statistically significant (p
>0.05). Conclusion: LDF readings from the surface of the
liver reflect the parenchymal flow. Mobilisation lowers the
surface flux. Surface LDF measurements are sensitive indicators
of changes in vascular blood flow. LDF may be used for
evaluating hepatic microcirculation during organ retrieval and
studying of the effects of preservation on hepatic tissue
perfusion.
PATTERN OF GRAFT REPERFUSION IN HUMAN
LIVER TRANSPLANTATION
V. Chidambaram A.M. Seifalian, K. Rolles, B.R.
Davidson.
University Department of Surgery, Royal Free Hospital &
School of Medicine, London U.K.
We monitored the hepatic surface perfusion of human liver
grafts using Laser Doppler flowmeter (LDF) during
transplantation Method: Four histologically fatty and 4
normal grafts were studied at the time of graft implantation.
The mean preservation period of the two groups were
similiar. We measured the hepatic surface perfusion from 2
sites on each lobe using a Multimoor LDF as follows: 1)
venous reperfusion on completion of vena caval and portal
venous anastomosis; 2) arterial reperfusion on establishing
hepatic arterial inflow to the graft and 3) final perfusion
before closure of the abdomen. The results given as
perfusion units (PU) % of final perfusion values were
analysed using one sample test and unpaired students test.
Results: The surface perfusion following venous reperfusion
was 60 +/- 23 PU. Additional arterial inflow resulted in an
increase of surface flow values to 79 +/- 24 PU. The final
perfusion values were Significantly higher than post venous,
arterial reperfusion values (p < 0.0001). There was no
difference (p > 0.05) in the graft perfusion of histologically
fatty or normal grafts. Conclusion: Surface perfusion of the
graft improves with time. LDF can monitor vascular inflow
to the graft. There is no difference in the pattern of
reperfusion of fatty and normal grafts.
P039 P040
SURGICAL TREATMENT OF LIVER METASTASES
Ciferri E., Filauro M.., Cesaro S., Municin<5 O., Mori L., Gazzaniga
G.M.
Ist Surg. Dept. San Martino Hospital, Genoa, Italy
Nowadays surgical indication for liver metastases is based on
precise criteria that include their number, dimension, site,
continuity/contiguity with important vascular structures and
limphnodal involvement. Preoperative staging is obtained by US/CT
scan evaluation, CEA and Ca 19.9 dosage.
Between Jan. 1980 and Dec 1995 we treated 183 patients (median
age: 60, range: 35-82) for hepatic metastases: 139 from colorectal
cancer, 44 from non-colorectal cancer (stomach, gallbladder, biliary
tract, pancreas, carcinoid, adrenal gland, pelvic sarcoma, breast,
aesophagus).
We operated on 153 patients.
Mortality and morbidity rates were respectively 4,1% and 18,4%.
5 ys' actuarial survival of colorectal and non-colorectal metastases
were respectively 24% and 6%.
In the cases without surgical indication we performed a schedule-
oriented biochemical modulation of FUra bolus by MTX and Fura
continuous infusion by Leucovorin, to reduce neoplastic lesions and
infiltration.
At today we treated 17 patients obtaining 3 complete surgical
responses after 6 months ofthis chemotherapy.
11 patients were reoperated for colorectal repeated liver metastases
with 22% of 5 ys' global survival and a mortality rate of9%.
80
REPEATED LIVER RESECTIONS FROM COLORECTAL METASTASIS.
Ciferri E., Gazzaniga G.M.lst Surg. Dept.-S. Martino Hosp. Genoa
Italy
Patients affected by repeat hepatic metastasis from colorectal
cancer can be submitted to a re-resection after a careful screening
excluding, first of all, extrahepatie recurrences. Surgery is the
only therapy that offers these patients a 5 ys survival rate of 20-
30%. Personal experience includes 11 cases; average age 59 (range
45-71) (Tab I; Jan. 1980 Oct. 1995).
Tab.l: RE-RESECTIONS FOR REPEAT LIVER METS FROM COLO-RECTAL CANCER;
PERSONAL EXPERIENCE
e prim.turn, dX.L lat dX.L 2nd state
aE M CECUM-2 ILW 7 4RW 3S ANED
rE s9 M RECTUM.SZ 3,
7 R,ob 2S* DWD
BG 60 M SlGM-B2 36 R WR S" DWD
RM SS M RECTUM-CI L WR 11 R lob 14* DWD
NE S4 M LEFT COL-CI 19 segm 7+8 30 R WR 39" AWl)
ZE 64 M SlGM-C2 R lob 17 R WR 18 DWD
TG 69 F SIGM-CI L WR 19 R WR 2S* DWD
PL $9
l! SIGM-CI $5 R lob L WR 1, AWD
10 FE 65 M DESC-BI IL+IR sgm6/7 ANED
11 SL 45 M SlGM.CI R lob 14 gm ANED
ANED=alive not evidence disease; DWDIAWD, died/alive with disease; *=died
All patients were free of symptoms at the diagnosis, which was
always made during follow up, testing CEA and CA 19.9, chest CT,
abdominal US/CT/NMR. The preliminary results from a
multiinstitutional study (Registry of repeat resections of hepatic
metastases) directed by P.H. Sugarbaker, started in 1991, that
involved 170 patients from 20 institutions, included our
department, have emphasized, although not all the data were
statistically significant, the importance of some factors as the
numbers of mets (<4), the limited distribution in the liver and the
non infiltration of important vascular structures, the absence of
extrahCpatic diseases, intended as distant, local or lymphnodal.
Among these last it's very important to know the status of
hepatoduodenal lymphnodes, considered the road of metastatic
cells' escape ("mets from mets cascade phenomenon"). When it's
impossible to perform a radical operation, from the oncological
point of view (adequate extension of resection and respect of clear
margin), surgery loses every curative capacity. New studies on
chemotherapy, after obtaining the first encouraging results, are now
trying to extend the possibility of surgery.
P041 P042
HEPATIC HYDATIDOSIS: 690 PATIENTS SURGERY-TREATED.
E. C6rdoba Diaz de Laspra, L. Lahuerta Lorente, JL Garcia Calleja, V
Ferreira Montero.
Department of Surgery, Miguel Servet Hospital, Zaragoza, Spain.
Hydatid disease Echinococcosis is parasitic condition of worldwide
distribution. The organism involved, Echinococcus granulosus, belong to the
order of cestode flatworms of the family Taenia. Humans happen to be
accidental incidental intermediate host. The liver is the first filter in the
way of ingested eggs, being the most site of location. Arag6n is
regarded intensive endemic with incidence rate of 7,54 100.000
habitants/year.
We present retrospective study carried out 690 patients with hepatic
hydatidosis, who underwent surgery at department during the last 24
years. The incidences in regard to sex, age, symptomatology, cyst-related data
and postoperative mortality/morbility also reviewed. Emphasis is placed
the surgical treatment.
The average age of the patients 39 years with similar incidence for both
The most frequent signs and symptoms the following: dyspeptic
symptons (60%); hepatomegaly and/or palpable in right hypocondrium
(59%) and pain (46%). Radical surgery, which includes liver resection of any
magnitude ortotal cystectomy, represents 20% of all surgical treatments; the
remaining 79% covered by conservative surgery external tube
drainage, internal drainage, parcial cystectomy ). The most frequent
postoperative complication biliary fistula (72 cases). The reoperation rate
17,87% and the operative mortality rate 1,62%.
In opinion, liver resection of any magnitude either to avoid spillage of
cystic contents to circumvent altogether the problems of managing the
residual pericystic cavity, problems than it solves. It is for this
that think it must be the surgeon's good jugment in deciding how to
tecnically manage each patient depending the size, location, contents ofthe
cavity and rigidity of the cystic wall forgeting that hepatic hydatidosis
is benign disease.
P043
COMBINED DIAGNOSTIC IMAGING OF HEPATIC FOCAL
NODULAR HYPERPLASIA
De Gaetano A.M., De Franco A., Maresca G.,
Manfredi R., Monteforte M.G.
Dept of Radiology, Catholic University
School of Medicine, Rome, Italy
Twelve cases of hepatic focal nodular hyper-
plasia were evaluated with color Doppler ul-
trasound (CD), dynamic computed tomography
(CT) and magnetic resonance imaging (MR):
the aim was to verify the diagnostic accura-
cy of these imaging techniques. CD usually
showed homogeneous and isoechoic lesions; a
central scar was rarely apparent; high va-
scularity was evidenced, and in 25 % of ca-
ses a stellate distribution of the vessels
was present. Doppler spectra showed medium
to high flow velocities and high diastolic
flow. On unhenanced CT scans all the lesions
appeared homogeneous and isodense, and in 30
% of cases a central scar was evident. At
dynamic CT the lesions showed intense, tran-
sient hyperdensity. On MR scans the lesions
were almost isointense in T1w and T2w ima-
ges, while the central scar was hypointense
in TIw and hyperintense in T2w. Ultrasono-
graphy was poorly specific although some
patterns were suggestive for the diagnosis.
CD showed 100% specificity, but sensitivity
was lower than 25%. For dynamic CT specifi-
city was 100% and sensitivity 80 %. MR added
no useful information in cases doubtful at
CT; sensitivity was. 40 %. These results in-
dicate that ultrasonography usually disclo-
ses and localizes lesions; CD adds valuable
data; CT shows the highest specificity while
MR gives no increase in diagnostic accuracy.
81
TRANSPLANTATION OF AUTOLOGOUS HEPATOCYTES IN PIGS: A
COMPARISON OF SPLEEN, PANCREAS, MESENTERY AND SMALL
BOWEL-WALL AS IMPLANTATION SITES.
R.B.J. de Bondt LM.Flenddg*, T.M.van Gulik, A.Bosma**, E.A.van
Royen***, R.A.F.M.Chamuleau*, H.Obertop.
Depts.of Surgery, Exp.lntemal Medicine*, Pathology**, Nuclear
Medicine***, Academic Medical Center, University of Amsterdam,
Amsterdam, The Netherlands.
The aim ofthis study was to examine various implantation sites for hepatocyte
transplantation in a large animal model, i.e. in the pig. Hepatocyte
transplantation was undertaken in the spleen, pancreas, mesentery and small
bowel-wall. Methods: The left lateral liver segments of pigs (n=5) were
resected and immediately perfused in vitro with collagenase. After isolation of
hepatocytes, 1.5ml volumes ofthe hepatocyte suspension (40x 106cells/ml,
viability 78-87 %) were injected into the parenchyma ofthe spleen and 0.25ml
volumes into the pancreas, mesentery and into the subserosa ofthe proximal
jejunum (n=4). The implantation sites were marked with an inabsorbabie
suture. In one control animal, the respective implantation sites were injected
with NaCI 0.9 %. At 1-3 months postimplantation, 20 ml of HIDA-Tc (200 Bq
activity) was administered i.v., and total body scanning (TBSc) was performed
during 45 mins. The spleen, pancreas and injected regions of mesentery and
small bowel were subsequently excised for organ scanning (OSc). At the same
time, a piece ofthe remnant liverwas scanned. Finally, the implantation sites
were excised, immemed in formaldehyde.and placed in a gammacounterfor
direct tissue counting, afterwhich histopathological examination took place
(HE stained sections). Results: During TBSc of all animals, no radioactivity
could be detected otherthan in the remnant liver. OSc showed activity in 2/4
pigs at the implantation sites in mesentery and small bowel-wall, after and 3
mths, respectively. Microscopical examination after 1-3 months, showed
clusters of viable hepatocytes at the implantation sites in mesentery and
bowel-wall of 4/4 animals. At the border ofthese hepatocytes, ductular
differentiation was observed. Direct tissue counting ofthese regions showed
high activity in the mesentery (5362-84537 CPM/g) and bowel-wall (10702-
403343 CPM/g). Control values of mesentery and bowel-wall were 2212
CPM/g and 3503 CPM/g, respectively. Activity in the remnant liverwas 10-55
x104 CPM/g. Only occasionally, hepatocytes were found in the pancreas in 2/4
animals. No hepatocytes could be detected in any ofthe implantation sites in
the spleen. Conclusion: The mesentery and small bowel-wall provide more
favorable sites for hepatocyte transplantatioin pigs than pancreas or spleen.
Organ and tissue scintigraphy with HIDA-Tc=', but not total body scintigraphy,
is useful forthe detection of hepatocytes after transplantation.
P044
REPEATED HEPATIC RESECTION FOR COLO-RECTAL
METASTASES
P. De Nardi, P. Gini, M. Stella, G. Ferrari, V. DI Carlo
ChinJrgia Generale IRCCS H S. Raffaele- Universit degli Studi di
Milano
Surgical resection of colorectal liver metastases is currently
accepted as the standard therapeutic approach. However
hepatic recurrence after liver resection is still an uncommon
indication for surgenj.
Between Januan/ 1980 and November 1995, 81 patients
underwent hepatic resection for colorectal metastases;
among these, 11 patients (13.5%), ranging.in age between
28 to 74 years, subsequently underwent resection for
recurrence of colorectal hepatic metastases. Seven patients
had solitan/and 4 multiple metastases, ranging between 2
to 5. Three major hepatectomies and 8 wedge resections
were performed.
No mortality was observed and morbidity was 27% (3/11)
with major complication.
Mean follow-up was 40 months. After the second resection
1,3 and 5-year actuarial survival rates were 100%, 71% and
29% respectively. Seven patients are alive 5-103 months
(mean 41) after the second hepatic resection. Four patients
are currently free of disease after a mean of 59 months
(range 5-113). In the other patients sites of relapses
included the liver in 4 cases, the lung in 3 and a local
relapse in one case. No characteristics of primary or
metastatic disease predicted outcome.
Our results suggest that repeated resection for colorectal
liver metastases can be performed safely and can result in
long term survival in selected patients.
P045 P046
ENDOTOXEMIA AND LIVER RESECTION WITH TOTAL VASCULAR
EXCLUSION (TVE).
De Panla JA, Spinedi E, Argibay P, Pekolj J, Moscone C, Bonofiglio C,
Ciardullo M, de Santibaites E.
Departments of Surgery and Gastroenterology, Hospital ltaliano of Buenos
Aires, Buenos Aires, Argentina.
The circulatory changes induced by TVE may alter the intestinal barrier to
endotoxins and/or reduce the capacity of the liver to clear endotoxins of
intestinal origin. The frequent observation of unexplained fever, diarrhea,
encephalopaty and liver failure after TVE induce us to study the LPS plasma
levels and its relationship with the surgery outcome. In 13 consecutive liver
resections using TVE we determined plasma LPS levels using the
chromogeni limulus lysate test (QCL-1000, Withacker, MA) immedialdy
before, .during and 5 rain., h and 24 Hs after releasing the lam. The
patients were dived in Group A: with normal LPS plasma levels during or
after the TVE, and Group B: with abnormally high level of LPS >0.1
EU/ml plasma). None of the patients had abnormal LPS levels before the
TVE.
Results are summarized in the table as median and range or proportions:
Group A (n=6) B (n=7) p
Plasma LPS (EU/ml) 0.045 (0-0.07) 0.27 (0.14-14)
ICU stay (days) 1.5 (1-3) 3.0 (3-22) 0.07"
Total hospital stay (days) 8 (7-10) 13 (3-27) 0.04"
MVS* (hours) 7 (4-24) 29 (20-144) 0.02f
Red cells transfused (units) 1.5(0-4) 6(4-6) < 0.001"
Fresh plasma transf.(units) 2 (0-5) 8. (4-6) 0.05"
Diarrhea 0/6 6/7 < 0.001#
Fever 1/6 6/7 < 0.001#
Encephalopathy 0/6 3/7 0.03#
Positive bloodculture 0/6 2/7 0.07#
Deaths 0/6 1/7 0.17#
*Mechanical ventilatory suplmrt,
"U-test, # Z-test.
Conclusion: Elevated levels ofplasma endotoxins during or after liver
resections with .TVE were associated with more demand oftherapeutic
procedures, more incidence ofcomplications and a more prolonged ICU and
hospital stay.
USE OF INTERNAL JUGULAR VEIN FOR VEIN GRAFT IN THE
LIVING RELATED DONOR IN LIVER TRANSPLANTATION
E. de Santibafies, M. Ciardullo, J. Mattera, J. Pekolj, J. Grondona, J. Sivori,
HPB Surgery Section, General Surgery Service, Hospital Italiano,
Buenos Aires, ARGENTINA
The living related donor in pediatric liver transplantation has been
developed in the wodd as a consequence of the poor offer of
cadaveric organs, specially for the receptors under 10 kilograms of
weight. One of the main challenge is about the revasculadzation of
the donor hepatic Segment. In a lot of receptors the portal vein
thrombosis or the short length are present with high frecuence. For
avoid this problem we have used the internal jugular vein of the living
related donor. The vein was studied previously with the color
ecodoppler image. During the operation of the donor, further the
hepatic segmentectomy we add a transverse cervical incision in the
neck, for good exposure and whole resect of the intemal jugular vein.
At the back-table this vein is sutured in on end-to-end anastomosis
with the left portal branch of the donor.Then in the receptor operation,
the free segment of the intemal jugular vein is anastomosed with the
splenomesentedc confluence. Of eleven cases with living related
donor in liver transplantation in 8 of then, this technique was
performed. Only one of these patients developed a portal vein
thrombosis sixteen days after the operation and required another
transplant with a cadavedc donor. The eight patients still alive and
with an excellent quality of life. Some transplant centers use the lilac
cadavedc vein or the crio-preserved vein, however in an experience
with animals that we have performed, we noted a high incidence of
vein thrombosis. Therefore we conclude that the intemal jugular vain
is an excellent graft for anastomosis to the portal vein.
P047 P048
RETRO -HEPATIC CAVA VEIN RESECTION DURING THE
TREATMENT OF REGIONAL TUMORS
E..de .Santibacs, M. CiarduIlo, .Mattera, J. Pekolj, L Grondona, L Sivori,
A. Aldet.
HPB Surgery Section, General Surgery Service, Hospital Italiano,
Buenos Aires, ARGENTINA
We analyze the technique that we performed in 14 patients with
tumors that involved the retrohepatic cava vein, and were resected
including a partial of total resection of this vein.The 14 tumors treated
with this procedure were: 3 hepatocellular carcinoma, 2 colorectal
metastasis, 2 clear cells renal tumor, 2 dght renal vein
leiomyosarcoma, 2 suprarenal, 2 carcinoma, cava vein
leiomyosarcoma, hepatic hamartoma, hepatic sarcoma. We used
the total hepatic vascular exclusion in 7 patients and partial hepatic
vascular exclusion in 7 patients. 6 of the last groups with total control
of cava vein and with partial, control. The partial or total resection
of the retrohepatic cave vein was combined with 9 hepatic resection
of differents sizes, 6 nefrectomies, 5 suprarenalectomies an
orthotopic liver transplantation (in the patient with the hepatic
hamartoma that further involved the intra and suprahepatic cave
vein). The average time of surgery was 5.2 Hs, with a range between
3.6-8. The red cells average consumption was 2.2 units, with a range
between 0-5 and the plasma average consumption was 2.5 units, with
a range between 0-5.5. Operative complications occurred in 7
patients: 3 pleural effusion, 2 intrabdominal abscess, and 2 lower
extremity edema. No operative mortality was observed in either of the
approaches.
LIVER RESECTION WITH VASCULAR CLAMPING:
RESULTS ON NORMAL AND PATHOLOGICAL
LIVER
N.Demartines, A Gavelli, C.Huguet
Department of Surgery, Centre Hospitalier Princesse Grace
MC 98012 Principality of MONACO
The tolerance of normal human liver to normothermic ischemia
for up to 60 minutes is now well documented and routinely
carried out for major hepatectomy. But pathological livers may
not tolerate a long continuous ischemia and intermittent clamping
is preferred by most authors.
Among 107 consecutive partial hepatectomies performed with
use ofuninterrupted vascular damping, 73 were achieved on
normal, 20 on steatosie and 14 on cirrhotic livers (Child A).
A prospective study analysed the clinical and biological outcome
and compared the results in the 3 groups.
3,5 segments with an average isehemia time of 45 minutes were
reseeted in each group. Peroperative blood transfusion
requirement was significantly higher in the steatosie :1375 ml
versus 650 ml in normals( p < 0,025), and 250 ml in eirrhoties
(p < 0,01).
Biochemical post operative course was similar in all groups.
Operative mortality was nil. Postoperative complication rate was
32.9% in normals, 35% in steatosics and 28.6% in eirrhotics
(no statistic difference) but mortality was significantly higher in
cirrhoties: 14.3% versus 1.4% in normals (p<0.025), and 10% in
steatosic (n.s).
The results ofthis study suggest that liver isehemia is fairly
tolerated by. patientswith pathological livers and preserved
hepatic function. It may be recommended to decrease
peroperative blood loss during the course ofliver resection.
82
P049 P050
ADENOMA, HEPATIC HEMANGIOMA, FOCAL.NODULAR
HYPERPLASIA AND HYDATID CYST OF THE LIVER.
CASE REPORT.
I. Di Carlo, C. Candiano, B. Papillo, G. La Greca, S. Puleo
First Surgical Clinic, University of Catania, Catania, Italy
The association between hepatic hemangioma (HH) and
focal nodular hyperplasia (FNH) or between FNH and
adenoma has been reported. The authors reported a case
in wich simultaneously there were FHN, HH, adenoma and
hydatid cyst.. A 25-years-old woman was admitted at the
First Surgical Clinic of University of Catania, after
presenting pain in the right hypocondrium. No theraphy
with oral contraceptives and no prgnancy were found in
the disease hystory of the patient. No abnormalities of
laboratory tests were found. US and CT scans showed 4
masses with the caratheristics of HH, adenoma, FNH, and
hydatid cyst located respectively in segment II, III, IV and V.
The patient was submitted at the enucleation of hepatic
hemangioma, resection fo hydatid cyst and enucleation of
the adenoma and FNH. Post operative course was without
complications and patients was discharged from the
hospital after one week. Pathological examination
confirmed preoperative diagnosis. At our knowledge,.
association of hepatic hemangioma, FNH, and adenoma,
has never been reported. The simultaneously presence of
these three different kind of tumor suggest that it should be
the different expression of the same malformative anomaly.
CYSTIC ARTERY UTILISATION FOR PORT-A-CATH
IMPLANT IN PRESENCE OF RIGHT HEPATIC. ARTERY
ORIGINATING FROM SUPERIOR MESENTERIC ARTERY.
I.. Di Carlo, R. Frasca, R. Lombardo, G. Li Destri, S. Puleo
First Surgical Clinic, University of Catania, Catania, Italy
Hepatic arterial infusion chemotherapy is used to treat
either primary or metastatic liver cancer when patients are
not candidates for surgery. When the right hepatic artery
(RHA) origin from superior mesenteric artery (SMA) two
options are possible: ligation of the right hepatic artery and
insertion of the port-a-cath in gastro-duodenal artery
(GDA), or insertion of two catheter, one in GDA and a
special catheter in RHA. With the first technique shoul be
not perfused and with last technique arterial trombosis
frequentely occurs. The authors reported their experience
concerning utilisation of cystic artery to insert a port-a-cath
when RHA is present. From 1991 to 1995, 25 patients were
operated on at the First Surgical Clinic of University of
Catania, for port-a-cath placement to perform Ioco-
regional chemotherapy. In three patients (12%) affected
by non resectable colorectal metastasis preoperative
arteriograms showed RHA origin from SMA. In all these
cases the cystic artery was utilised to insert a catheter for
chemoinfusion of right liver, associated with insertion of
catheter in GDA for chemoinfusion of the left liver.
Intraoperative and postoperative control showed perfusion
of both of the lobes of the liver. Complications occurred in
none, and utilisation was performed from 6 to 24 months.
Utilisation of cystic artery preservs the native flow and is a
valid option to implant a port for chemoinfusion of the right
liver when RHA origin from SMA.
P051 P052
MAJOR LIVERRESECTIONFOR NON-COLORECTAL
HEPATIC METASTASES.
A Dfez Caballero, F Regueira, A.Sierra, A.Espf,
E.Nwose, J. Baixaulf F. Pardo, J.L. Hern(mdez, J A-
Cienfuegos.
Dpto of Surgery, University Clinic of Navarra,
Pamplona, Spain
Six patients with unilobular non-colorectal
metastases were studied for hepatic resection. The
metastases are from suprarenal carcinoma,
carcinoid tumor of small bowel, squamous cell
carcinoma of the lung, ampuloma in one patient
each respectively and leiomyosarcoma of the small
bowell in two patients. There were one synchronous
and five metachronous lesions. Three patients had
solitary metastatic lesions and three had multiple
lesions.
We realized six liver resections which
comprised 7,7% of the liver resection realized in our
center and 15% of liver resection done for
metastases. The surgical procedure was right
hepatectomy in five cases and right hepatectomy
with atypical resection of left lobe in one. There was
no surgical mortality. Two patients presented with
abscess and in one hematoma intrahepatic The
patients were followed up for 23,5 months (5'47), the
six all are alive, four are free of disease and two had
recurrence of the disease.
Major liver resection for metastases although
done mainly .for colorectal metastases is advocated in
patients with primary tumour other than colorectal
origin with equally long term survival.
PROGNOSTICEVALUATION OFSYNCHRONOUS AND
METACHRONOUS LIVER MEI'ASTASES IN COLORECTAL
CANCER
A Dfez Caballero. F Regueira, A.Sierra, A.Espf,
E.Nwose, J. Baixaulf F. Pardo, J.L. Hemtndez, J A-
Cienfuegos.
Dpto of Surgery, University Clinic of Navarra,
Pamplona, Spain
The controversy as to prognostic factor of the
appearence of liver metastases in colorectal cancer is not yet
settled.
We studied retrospectively the survival in patients
undergoing liver resection for colorectal metastases. The
series comprised 31 patients who were divided in three
groups. Group metastases synchronous (11 patients); group
II metastases metachronous in the first year of follow-up (7
pat); and group III metastases metachronous diagnosed more
than one year of follow-up 13 pat). The groups are
comparable for age, type of liver resection, number of
metastases, tumour size and resection margin.
In the group with a follow-up of 19 months (8-38).
10 patients (91%) had recurrence and 6 died(54%). In the
group II, with 17 month follow-up (6-24 months) 6 patients
(85%) had recurrence and 2 deaths (28%). In the group III
after 24 months of follow-up patient died(7%), 2 (14%)
presented with recurrence in the liver treated with re-
resection and 11 patients (78%) were free of tumour.
There was a significant difference in the survival
among synchronous and metachronous metastases; and in the
disease free interval among metachronous metastases which
appeared in the first year when compared with those with
more than one year of follow-up
83
P053 P054
REPEATED RESECTIONS FOR HEPATIC METASTASES
FROM COLORECTAL CANCER.
R. Doci, P. Bignami, P. Bagnoli, L. Gennari.
Istituto Nazionale Tumod, Milano, Italy.
Up to December 1994 hepatic resections for metastatic
colorectal cancer were performed with curative intent in 263
patients. Five of them died postoperatively from complications.
During follow-up 110 patients developed a recurrence confined
to the liver; 21 of these patients (19%) underwent hepatic re-
resection.
Interval between the two procedures ranged from 3 to 95
months (median 12 months). Surgery was non-anatomic
resection (NAR) in 18 cases, and formal hepatectomy in 3; most
ofthe patients had a single metastasis, but eight had 2 and one 5
metastases; in 2 patients a lung resection, in an intestinal and in
another a diaphragmatic resection were associated to hepatic
resection. Median blood loss was 500 ml (range 0-3000). One
patient (5%) died intraoperatively and 6 (30%) had
postoperative complications. Median survival from first and
second hepatic resection was 62 and 30 months respectively.
Actuarial 3- and 5-year survival from re-resection was 49% and
39% respectively. Three patients who developed a new
recurrence confined to the liver were submitted to the 3rd
hepatic resection; postoperative outcome was uneventful. The
results observed in this series support the indication to hepatic
re-resection in selected patients.
A NEW NEEDLE TO PREVENT TUMOR SEEDING FOLLOWING
PERCUTANEOUS NEEDLE BIOPSY
B.M.Doorschodt T.M. van Gulik, H.Obertop, D.J.Gouma.
Dept. of Surgery, Academic Medical Center, University of Amsterdam,
Amsterdam, The Netherlands
Percutaneous Needle Biopsy (PNB) is a useful tool in the diagnosis of
tumors. Although the procedure is considered safe by using fine needles, it
is, however, not free of complications including severe and even fatal
ones. Complications associated with PNB are post-biopsy hemorrhage and
dissemination of malignant cells in the needle tract resulting in
implantation metastases. To investigate the incidence of complications
due to PNB, we performed a literature analysis covedng the pedod of
1966 through 1995. 102 reports of needle tract seeding were identified
after PNB of a vadety of organs, describing a total of 145 cases. With
respect to liver and pancreas, tumor seeding was reported in 26 and 14
cases, respectively. Fine needles were used in 55 cases, core needles in
40 cases. The dsk of fatal hemorrhage after liver biopsy in patients with
malignancy is estimated to be 0,4%; for nonfatal hemorrhage 0,6%. This
analysis revealed that needle tract seeding and post-biopsy bleeding are
potential complications of PNB. To prevent the occurence of tumor
seeding following PNB and to decrease the risk of post-biopsy
hemorrhage, a concept was developed for a new biopsy needle. The
concept consists of a combination of a biopsy needle and a biodegradable
sheath. After the biopsy specimen is taken, the protective sheath remains
inside the body at the biopsy site. Detached malignant cells dudng
withdrawal of the biopsy needle are trapped inside the canal created by the
sheath and disintegrate isolated from the body. The resorbable material,
gelatin, has an active hemostatic effect on possible hemorrhage. A
prototype of the new needle has been developed. Conclusion: Analysis of
the literature showed that PNB causes a potential dsk of implantation
metastases and hemorrhage, particularly in liver and pancreas. By using a
needle with a protective biodegradable sheath, percutaneous needle
biopsy could become a safer procedure, with a decreased risk of post-
biopsy hemorrhage and without the dsk of causing needle tract seeding.
P055 P056
De!t of Surgery snd Radiology ',,
Spli Nepablic of (catia
In the rettive study e amlyzed atients with liver injuries
during the War in te seuth art of (latia from 1992. to 1994.treted
in t mr hospitals ad the ge hospital in Split. There ee
1268 ar casulties and 105 (8,/) of tham ad abdakrgl wounds and
16 (I,2fP/) ad liver injuries. Isolated liver injuries are in five
(4,7fff). CIF four (3,) atfents ee ured by velocity
ojectiles ar the others e injured by different types of mires,
qe most of liver ines ere the second and third grade acco
ta Scale of ( injury Scalirg Cttee-Aerican Association of
E[gery of ,.1988. Tve (11,4/) atients ere operated by
suture cr cmentop..]@sty ar feur (3,/) atients ere treated
tively ad nnitored clinicaly, by lab., ultrascund ad CT
Ce of the ccnservatively treated atients had liver ascess some
here s no mortality in the gr of atients with liver ires
treated by surgical cr ocnservative mete.
Ccusicn Patients with isolated ar injuries of liver grade I cr
II and with f can e treated ocservatively
HIIN AND ANDBIISON I-IPANY IN PAT I/SINg A
NICIIOSUIICAL TCHNIQIJg
H.J. Duran, G. Roddguez-Fabian, S. Alonso, L Lorente, M.A. Aller, JArias.
Department of Surgery. Universi Hospital "San Carlos". UCM. Madrid,
Spain.
Hepatic regeneration could be studied when different types of
hepatectomies are performed in the rat. The two superior lobes
hepatectomy in the rat, that consists in the resection of the 64,9% of the
hepatic parenchyma, was described by HIGGINS and ANDERSON in 1931,
using a macrosurgical technique.
A new experimental model of this hepatectomy in the Wistar rat (n=16),
but using a microsurgical technique (Olympus; 10x4x0,5) is described. At
first, the hepatic hilum of the middle (ML) and left lateral (LLL) lobes, that
is, the hepatic artery, the portal vein and the bile duct, were ligated
together. Next the falciform and posterior, right and left, ligaments of the
liverwere sectioned. The, the right hepatic vein, that drains the middle and
the right portions of the blL, was sectioned between ligatures. The drainage
of the middle hepatic vein, that drains into the SH-IVC the left portion of
the right blL, is close to that of the left hepatic vein, that drains the left
ML. This anatomic feature makes it possible the ligation of both veins
together without stenosis of the SH-IVC.
A body weight loss was experienced by the animals until the 5th day of the
postoperative period and at 21 days after the operation the body weightwas
similar to that of the control rats. On the 21th day after the operation the
size and the weight of the liver were similar to those of the control
animals, so regeneration of the non-resected hepatic lobes, right lateral
(RLL) and caudate (CL) lobes, have restored the overall hepatic mass.
The 70% hepatectomy using a micmsurgical technique is the mostsuitable
method to avoid the iatrogenic functional impairment of the remaining
hepatic parenchyma.
84
P057 P058
INTERMITINT VASCULAR EXCLUSION OF THE LIVER 0VEL)
(WITHOUT VENA CAVA CLAMPING), DURING MAJOR
HEPATECTOMY
D. Elias, Ph. Lasser, B. Debacne, S. Bonvalot
Department of Surgical Oncology, Institut Gustave-Roussy, Centre de Lutte
Contre le Cancer, Villejuif, France
Intermittent vascular exclusion of the liver is possible by association
clamping of the hepatic pedicle with clamping of the main hepatic veins
without interruption of caval flow. Like in the intermittent clamping of the
hepatic pedicle alone, it can be performed for more than 120 minutes
without postoperative liver failure, and avoids the detrimental
hemodynamic effects of vena caval clamping. It concerto the problem of
looping the terminal part of the three hepatic veins, and particularly the
common trunk of the middle and left hepatic veins or these two veins
separately, a manoeuvre which is usually considered as dangerous. In this
retrospective study, we analyse eight cases oftotal IVEL and eight cases of
partial IVEL (involving only the middle and lefl hepatic veins) during
major hepatectomy for malignant tumors. Liver parenchyma was
pathological in nine cases. IVEL was feasable in 89% of the 18 attempts,
and seemed unfeasable in one case with a huge tumor and in one case with
a very rigid cirrhotic liver. IVEL was efficient in reducing bleeding during
hepatectomy in 94% of the cases and failed in only one case with post-
chemotherapeutic fibrotic liver in a patient with a high venous pressure
(due to fluid overload). Mean duration ofIVEL was 60.2 minutes (range: 37
to 140), mean blood loss was 1230 mL (range 300 to 2800), and there
were no postoperative complications related to this procedure. For us, the
indications for IVEL are: a) hepatectomy for tumors close to the large
hepatic veins, b) conservative hepatectomy of pathological liver
parenchyma with high intraparenchymous venous pressure, c) poor
hemodynamic tolerance to vena cava clamping (which occurs in 18% ofthe
patients), and d) some cases necessiting reconstruction of a vital hepatic
vein. The major advantages of this technique of liver vascular exclusion
(good tolerance and possibility of long duration) me+tit its inclusion in the
list of different clamping techniques available for use during hepatectomy,
each of them having specific indications. We .conclude that IVEL is
feasable, efficient and indicated in numerous hepatectomies.
LIVER TRAUMA: RESULTS OF TREATMENT
.Endzlaas, A. Maleckas
1st Surgical Clinic of Kaunas Medical Academy, Lithuania.
Aim of the study: to analyze a group of patients after abdominal trauma and liver
injury, taking into consideration the character of trauma and treatment results.
In 1990-1994 415 patients with different abdominal trauma were treated. Closed
abdominal trauma was diagnosed in 58% cases, stabbed-cut injury -in 36%,
shotgun injury- in 6%. Liver trauma was diagnosed in 65 cases (16% of all
patients), males prevailed (75.4%). The age of patients in the group with liver
trauma ranged between 16 and 67 years, mean age 33.5+-10.2 years. Liver injury
was found in 28 cases (12%) of the blunt abdominal trauma group, in 33 cases
(22%) of stabbed-cut injury group, in 4 cases (15%) of shotgun injury group.
Diagnosis of liver injury was made on the basis of clinical findings, laboratory
analyses, ultrasound or laparoscopic investigations. In some cases we used
diagnostic laparocenthesis. All patients with liver trauma underwent laparotomy.
In the group of patients, operated because of blunt abdominal trauma with liver
injury (lst group), isolated liver trauma was found in 32% of cases, in the group
with stabbed-cut abdominal damage with liver injury (2nd group) in 64% of
cases, and in the group with shotgun abdominal damage with liver injury (3rd
group)- in 25% of cases. Multiple injuries were found in 68% of patients in the
1st group, the lien or bones being concomitantly injured in most cases. Mortality
rate in the 1st group was 32%. All fatal outcomes were in cases of multiple
abdominal or combined abdominal and brain thoracic trauma, accompanied by
severe shock and impairment ofvital functions. Multiple injuries in the 2nd group
were found in 36% of cases, 2 of them had fatal outcome. Multiple abdominal
organ injuries in the 3rd group were diagnosed in 75% of cases, however, without
any serious impairment of vitally important organs. We had no fatal outcomes in
the 3rd group. Liver suture was performed in all cases of the 2nd and the 3rd
group; in the 1st group it was performed in 26 of 28 cases, while marginal
resection was needed only in the rest 2.
Conclusions: The mortality rate in liver trauma often depends on concomitantly
affected organs and reaction of the organism to developing shock, ARDS, or
acute brain disorders. We suppose that liver injuries be successfully treated by
using primary sutures and avoiding performing of resections whenever possible.
P059 P060
THE LIDOCAINE (MEGX) TEST PREDICTION OF THE
POSTOPERATIVE COURSE AFTER HEPATIC RESECTION
G Ercolani A Mazziotti, GL Grazi, E Jovine, R Calliv/, M Morganti,
M Masetti, F Pierangeli, A Prindpe, A Cavallari
2 Dept Surgery, University ofBologna, S.Orsola Hospital, Italy
The postoperative course of pts. undergoing liver resection (LR) was
analyzed with respect ofthe performance ofthe lidocaine (MEGX) test, a
new approach to the evaluation of hepatic function. MATERIAL AND
METHODS From 2/93 to 2/95, 85 LRs were performed for HCC (11
pts.), HCC on cirrhosis (37 pts.), metastases (21 pts.) and others diseases
(16 pts.). There were 58 males and 27 females. According to the Child-
Pugh classification there were 83 A's and 2 B's or C's. There were 15
wedge LRs, 42 segmentectomies and 28 major hepatectomies. The MEGX
test was evaluated before LR and results compared with the postoperative
course. RESULTS The MEGX value was normal (i.e. > 50 tg/ml) in 46
(54.1%) pts; it was between 25 and 50 in 26 (30.6%) and it was < then 25
in 13 (15.3%). One cirrhotic patient died within 30 days from surgery (he
had a pre-operative MEGX value of 40 lag/ml). Atotal of 21 (24.7%) pts.
developed postop, liver-related complications (hepatic failure, ascites,
jaundice). The crosstabulation of the MEGX value with the presence of
postop, c )mplica.tion is as follows:
Compl. < 25 (%) 25-50 (%) > 50 (%) Total (%)
Yes 10 76.9 6 23.1 5 10.9 21 24.7
No 3 23.1 20 76.9 41 89.1 64 75.3
The tendency to observe a lower complication rate with better MEGX
values reached a significant difference (P<.0001). The same significant
trend was also observed when considering only the 37 cirrhotics (P<.002).
Furthermore, in these 37 pts., the postop, stay was 11 +/- 3.8 days when
MEGX was < 25 (13 pts.); 10 +/- 2.7 when MEGX was between 25 and 50
(21 pts.); 9.6 +/- 0.5 (3 pts.) when MEGX was > 50. CONCLUSIONS
The MEGX value appears to be a valuable predictive index for the
insurgence ofpostop, complications, mainly in cirrhotics.
HYDATOID DISEASE NO LONGER SEEN IN NORWAY
G.M. Ertvaag *, M. Hkonsen*, T.E. Gudmundsen*, H.
Ostensen*, E. Karlsen **
* Dept. ofRadiology, Central Hospital ofBuskerud,
Drammen, Norway
** The State Veterinary Laboratory, Harstad, Norway
Hydatide disease with liver and pulmonary affection was
regulary seen in Northern Norway until the 1970's.
Population at risk was mainly reindeer keeping laps. The
disease was transmitted to humans from reindeers via
domestic dogs specially used for keeping the animal herds
together.
In the year of 1977 the veterinary authorities ofNorthern
Norway started giving out a specific anthelminticum
(Dronocit (C), Bayer) free ofcharge to all actual families for
treatment oftheir dogs. This measurement combined with a
broad scale information campaigne was continued until the
mid 1980's. Thereafter, families in question continued
volontarily to treat their dogs for another four to six years.
Since 1982 no human hydatoid disease has been observed.
Furthermore, the occurrences ofhydatoid infection in reindeer
observed by the public meat control decreased dramatically
within a couple ofyears until disappearing completely.
In conclusion, in Norway, is has been possible to eliminate
hydatoid disease completely through an intense
combat/eradiction program.
85
P061 P062
A SIMPLIFIED APPROACH TO REGIONAL HEPATIC ARTERIAL
INFUSION FOR PRIMARY AND METASTATIC CANCERS OF THE
LIVER. S- Ettinghausen, R. Gray, M. Steves, R. Dalton, S. Givens-Crow,
P. Sugarbaker
Division of Surgical Oncology, Washington Hospital Center, Washington,
D.C., USA
Prior studies have established the efficacy ofhepatic arterial infusion (HAI) of
chemotherapeutic agents for the treatment of hepatic metastatic disease.
Techniques for HAI have included repeated transfemoral arterial
catheterization or direct catheter placement during laparotomy with an
implantable pump or port. Both methods are associated with well documented
risks, shortcoming and costs. In a joint effort between surgical oncology and
interventional radiology, we utilized a simplified technique originally reported
in the Japanese literature (Arai). In the procedure, which utilizes local
anesthesia, a 5ft. catheter is radiographically positioned into the hepatic artery
via the operatively-exposed left thoracoacromial artery (LTA), a branch of the
left subclavian artery. The catheter is then mated with a subcutaneous port
allowing subsequent easy-aocess for outpatient HAI therapy. In our initial
feasibility trial, 12 patients were considered'for placement of a LTA catheter
and port (CP). Two were excluded after angiography showed unfavorable
anatomy. Two had unsuccessful placement attempts due to inadequate size of
the LTA. Eight patients (6 male, 2 female; median age 64, range 34 73
years) had CPs inserted for treatment of cholangiocarcinoma (1) and liver
metastases from ampullary (1) and colorectal (6) cancers. Seven had prior
systemic chemotherapy. Insertion of the CPs took 4 hours (median) with a
range of2 6 hours and was not associated with any immediate complications.
A total of 38 cycles of chemotherapy was administered to the eight patients
using three different regimens. No patient developed symptomatic
gastroduedenal side effects or arterial thromboses. The CPs remained in situ
for II days, and 2+, 6, 8, 10, 13.5, 16 and 16+ months. In 3 patients the
CPs were removed: for infection (I days) and 2 for completion of therapy
(8 and I0 months). One of the 3 had a minor transient ischemic attack without
residual deficit following CP removal (8 months). While it is too early in this
trial to determine the efficacy of this approach, the LTA CP method appears
to be a simple, low-morbidity technique for delivering HAI chemotherapy and
avoids the cost and shortcomings of a laparotomy, repeated arteriotomies or
multiple inpatient admissions.
GALACTOSE SINGLE-POINT AS LIVER FUNCTION TEST
A. Fabbri, M. Brizi, G. Bianchi, G. Marchesini
Department of Internal Medicine, Univ. of Bologna, Bologna, Italy
Quantitative liver function tests are usually based on serial determi-
nation of hepatic substrates or products following oral or i.v. admin-
istration, but the need of rapid testing in donors and/or recipients of
liver transplant led to oversimplify procedures. Single-point tests have
been developed, such as single-point MEGX test, and also a galactose
single-point (GSP) test has been proposed. We aimed to determine
the error in liver function measurement introduced by simplified pro-
cedures. Galactose elimination capacity (GEC) was studied after i.v.
bolus injection in 905 subjects (Normal, 73 cases; Chronic hepatitis,
116; Cirrhosis, 716). Blood galactose were determined at 5-10 min
intervals, between 20 and 60 min after injection. Galactose levels at
time t=45 min (GSP45) or t=60 min (GSP60) were considered, and
their predictive value on final GEC, considered as gold standard in
liver function assessment,,was calculated by linear regression. There
was a good correlation between GSP and GEC, with differences in
relation to liver function (GSP45 vs GEC: Normal, R2=0.54; Chronic
hepatitis, R2=0.43; Cirrhosis, R2=0.31). In cirrhosis GSP60 was
better (R2=0.53, n=197), but still related to liver function (Child A,
Rz=0.64; B, R2=0.44; C, R2=0.32). The 95% confidence interval of
GEC predicted by GSP45 was as large as -41% to +47% of calcu-
lated GEC, and also in cirrhosis it varied within -30 to +18% in Child
A, -22 to +42% in Child B, and ,33 to +101% in Child C. GSP60
was slightly better (Child A, -12 to +26%; Child B, -23"to +21%;
Child C, -39 to +75%). The uncertainty of liver function measure-
ment based on GSP increased with poor fitting of the experimental
data on the regression of galactose concentration on time and low
galactose elimination. It was maintained within limits acceptable in the
decision-making process (5: 10%) only in nearly 50% of tests.
The measurement of liver function based on GSP, although sup-
ported by correlation with GEC, introduces a considerable error in
quantitative assessment, mainly in critical patients with more
advanced disease. The error is likely to be similar for any single-point
test, and must be considered whenever simplified procedures are used
for clinical purposes in the decision-making processes.
P063 P064
Effect of cold storage in University of Wisconsin
solution on the responses of porcine hepatic arteries to
S-hydroxytryptamine and bradykinin in vitro
S.. Flanders, K.J. Hardy, M.J. Lew
Departments of Pharmacology and Surgery, The
University of Melbourne, Australia
Responses to 5-hydroxytryptamine (5-HT), bradykinin
and sodium nitroprusside (SNP) were examined in
porcine hepatic arteries hour after dissection (fresh) and
following .24 hours storage in either Ca2+ free Krebs'
solution or the cryopreservative University of Wisconsin
(UW) solution.
In fresh arteries contracted to approximately 40% of the
maximum response to potassium with U46619, a
thromboxane A2 mimetic, concentration-response curves
to 5-HT were biphasic, with relaxation at low
concentrations (< 10-8M) and contraction at high
concentrations. Bradykinin produced
concentration-dependent relaxation of precontracted fresh
arteries with no apparent constrictor response.
Treatment of fresh arteries with NG-nitro-L-arginine
(L-NOARG, 10-4M) significantly attenuated the
relaxation response to 5-HT and displaced the bradykinin
concentration-response curve four-fold to the fight with
no affect on its maximum relaxation.
From these results it is concluded that cold
storage-induced damage to the endothelium is reduced by
UW solution compared to Ca2+ free Krebs' solution.
LIMITED RESECTION FOR HEPATOCELLU LAR
CARCINOMAS LESS THAN 5CM IN DIAMETER
Y.Fukuda, S.Yogit.a, S.Tashiro, T.Ohnishi, K.Mise, M.Ishikawa, H.Miyake,
M.Harada, D.Wada
The First Department of Surgery, The University of Tokushima, School of
Medicine, Tokushima, Japan
Hepatocellular carcinomas(HCCs) have higher equivalence of multicentric
synchronous and/0r metachronous occurrence. Extended or curative resection
HCCs is not always associa.tedwith prognosis. We determined the optimal surgical
procedures for primary HCCs by the evaluation of resected cases. Materials and
Methods]The therapeutic outcome after hepatic resection fi)r HCCs examined
in 89 patients followed up for more than 12 months postoperatively, absolute
non-curative cases were excluded. Categorization of predictive factors for recurrence
was performed by the general rules of primary liver cancer of the Japanese Liver
Cancer Study Group. [Results] The overall 3-year and 5-year disease free
survival rates were 39% and 38%, respectively. The overall recurrence rate was
39.7%(31cases). The significant factors affecting recurrence were age, aspartic
transaminase, eholinesterase, ICGR15, protein induced by vitamin K absence or
antagonist(PIVKAlI), severity of liver disease and involvement of portal vein.
Tumors more than 5cm in diameter had significantly high prevalence of portal
involvement and intrahepatic metastasis, and showed poor outcome in 5-year
disease-free survival rate. Resection less than subsegmentectomy(Hr0) was
associated with lower recurrence rate than resection 0f or more segments.
Five-year disease-free survival rates of bsolute-curative, relative-curative,
relative-non-curative resection were 50%, 22%,.48%, respectively. In the group of
relative-non-curative resection, 36 of 37cases were less than 5cm in diameter. I-IBV
related and HCV related cases were 21cases and 63cases 5-year disease-free survival
rates were 48%, 38%, respectively. In HCV related cases, multiple synchronous
occurrence was observed in 19cases(30.1%) at the operation, and 14 of the
19cases(74%) were considered of multicentric occurrence. Furthermore, in HCV
related cases, the duration from operation to recurrence was longer than that ofHBV
related cases, and the cases of metachronous multicentric recurrences were
!4cases(58.3%). [Conclusion] In conclusion, limited resection should be
indicated for HCCs less than 5can in .diameter without portal involvement nor
intrahepatic metastasis, especially in cases of hepatitis C related.
86
P065 P066
EVALUATION OF THE PRESSURE OF THE PERIHEPATIC
PACKING IN LIVER TRAUMA
E.M. Gadijev, D. Stanisavljevi6, M. Wahl, J. Butinar*, V. Pegan,
Department ofGastroenterologic Surgery, University Clinical center,
Ljubljana, Slovenia,
*Veterinary Faculty, University ofLjubljana, Slovenia
Liver trauma with injury of major hepatic veins is still a serious
surgical problem. Revival of perihepatic packing as a therapeutic
method brought also some problems. Shortcoming on one side and
overdoing on the other side may both compromise the method.
We made a study on six anestetised dogs in order to quantitatively
measure the pressure of appropriate perihepatic packing. Catheters
were introduced in the inferior vena cava and right atrium.
Tourniquet was placed over the liver to simulate the perihepatic
packing. While increasing the pressure over the liver we measured
the pressures in the inferior caval vein and in the right atrium.
The inci,ease of the pressure on the liver from 10 to 25 mm' Hg
increased the pressure in the inferior vena cava from mean cm
H20 up to mean 8 cm H20. At the same time the pressure in the
right atrium decreased from mean 0,5 cm H20 to mean -1,5 cm
H20 and also mean arterial pressure decreased from 60 mmHg to 35
mmHg. After the measurements we replaced the tourniquet with
packs. We provisionally closed the abdominal wall and at the same
time we tried to achieve the same pressures in the vena cava inferior
and in the right atrium as with the tourniquet.
The pressure on the liver between 20 and 25 mm Hg caused the
haemodinamical important fall ofthe pressure in the right atrium and
the arterial pressure. At the same pressure on the liver the pressure in
the inferior vena cava arose to 7-9 cm ofwater.
We concluded that with measuring the pressure in the inferior vena
cava by introducing a catheter, we can achieve the convenient
pressure for adequate perihepatic packing in serious liver injury.
INTERMITTENT VS CONTINUOUS ANOXIA/REOXYGENATION
HEPATOCYTES: EFFECT ON OXYGEN FREE RADICALS FORIVlATION AND
CYTOTOXICITY.
A. Gasbarrini*, A. Colantoni^, P.. Caraceni,, F. Trevisani^, E. Iovine', A.
Mazziotti', A. Cavallari ', E. Roda', M. Bemardi^.
Patologia Medioa ^, Cattedra di G-astroenterologia', Clinica Chrurgica It'.,
Universita' di Bologna; Patologia Medica*, Universita' Cattolica, Roma, Italy.
Temporary clamping of the hepatic pediole according to Pringle (Pringle
manouvre) is the most used technique to control intraoper atory bleeding durir;
hepatoreseetion. However, liver damage due to isehernia/reperfusion is a main
problem in prolonged damping. Intermittent clamping has been proposed as a
method to seduce organ injury. Aim of this study was to assess oxygen free radicals
formation (OFR) and cell injury during continuous or intermitent
anoxia/reoxygenation in rat hepatooytes.
METHODS: afar isolation, the cells were cast in agarose gel threads and
continuously perfused with oxigenated (95%O2-5/4202) Krebs-Henseleit
bicarbonate buffer. Hepatooytes were exposed to 2 h continuous or intennittent (4
periods of 30 m of anoxia followed by 10 m of reoxygenation) anoxia obtained by
95%N2-5%CO2 perfusion and to a h reoxygenation period. Cell injury was
evaluated by LDH release; OFR by enhanced-chemiluminescence: lucigenin and
luminol were utilized to enhance anion superoxide (02-) and hydrogen peroxidt,,
(H202) formation, respectively.
RESULTS: afar 2 h of anoxia, OFR formation was reduced to barely measurable
level in both groups. LDH release was significantely greater in hepatocytes exposext
to continuous compared to intermittent anoxia: 580-0 31040 % (13<0.01).
During reoxygenation O2- and H202 formation increased in both group, but it wa,,
significantly higher in cells exposed to continuous anoxia: 02:1004.11 vs 434-12 rha.
(p<0.01); H202:834-12 vs 384.10 nA (p<0.01). Parallely, during the early phase o:f
reoxygenation, LDH release increased significantly in both groups but it was highest
in cells exposed to continuous anoxia: 9004.40 vs 5504.65 (p<0.01). In both group,,
the lucigenin chemiluminescence peak, expression of 02 formation, occurred 10-15
before maximum LDH release.
CONCLUSIONS: These data suggest that: 1) since LDH release is temporally
delayed respect to 02- formation, OFR production seems to be the cause and not the
consequence of reoxygenation injury; 2) intermittent oxygen deprivation is able to
reduce liver eel1 injury and oxygen free radicals formation determined by
anoxia/reoxygenation; 3) when the Pringle manouvre is used to perform an
hepatoreseetion, it should be applied intermittently rather than continuously.
P067 P068
EFFECT OF ISCHEMIA/REPERFUSION ON HEAT-SHOCK PROTEIN 70 AND
90 GENE EXPRESSION IN RAT LIVER: RELATION TO NUTRITIONAL
STATUS.
A Gasbarrini. S Degli Esposti*, S De Notariis, P Caraeeni, S Loffredo*, M
Simoneini, A Colantoni, F Trevisani, A Mazziotti, A Cavallari, M Bemardi.
Universita' di Bologna and Universita' Cattolica di Roma', Italy; Brown University*,
Providence, USA.
Heat shock proteins (HSP) are intracellular proteins associated with
generalized response of the cell to stress conditions. Anion superoxide generation is
ofthe factor that determine HSP gene expression in post-isehemie condition. Otr
group recently showed that the production of anion superoxide induced by
reoxygenation in hepatooytes isolated from fasted rats is greater than in cells obtainext
from fed animals.
Aim ofthe study: to assess the levels of messanger RNA (mRNA) for HSP genes 70
and 90 in liver from fed or 24 h-fasWxi rats in baseline condition and during perkxt
of 60 min isehemia/120 rnin reperfusion. Reduced glutathione (GSI-1) was evaluated
to assess antioxidant status ofthe tissue.
Methods: liver isohemia was induced by placing miorovaseular damps around the
appropriate branches of the portal vein and hepatic artery of the lef lateral and
median lobe. Northern blot analysis of total RNA extracted from liver tissue was
performed. Specific P32 labelled eDNA probes for HSP 70, HSP 90, albumin and
reference Riboprobe were utilized. Densitometry analysis of autoradiograms was
performed to obtain semiquantitative data. GSH was assessed by spettrophotometric
methods.
Results: in baseline condition, liver from fasted animals presented significant
differences compared to organ from fed rats: mRNA for HSP 70 and 90 were
increased 2-3 fold and GSH was decreased by 40 % (0.724.0.1 vs 1.264.0.1 nM/mg;
p<0.01). After 60 rain isehemia, liver from starved rats presented 2-3 fold decrease
in HSP 70 and 90 mRNA, while HSP gene expression did not change significantely
in liver from fed animals; GSH decreased 55 % (to 0.320.1 nM/mg) in fasted liwr
and 25 % (to 0.944.0.1 nM/mg) in fed organ. Upon 120 rnin reperfusion, HSP 70 and
90 mRNA rose 4-5 folds only in fasted animals, while slight decrement in gene
expression was observed in the fed group; GSH concentration retttmed to 65 and 85
% ofbaseline value in liver from fasted and fed rats, respectively (to 0.474.0.1 and to
1.074.0.1 nM/mg).
Conclusion: prolonged period of fasting determines by itself significant reduction
in liver antioxidant status and an induction of HSP gene expression. Moreover, only
in the fasted group the reperfusion phase that follows a period of isehemia was
characterized by a marked rise in HSP mRNA. The reduced antioxidant status and
the greater generation of anion superoxide determined by fasting may be the
mechanisms underlying this phenomena.
CHEMILUMINESCENT REAL TIME IMAGING OF OXYGEN FREE
RADICALS FORMATION IN ISOLATED LIVER EXPOSED TO
SCHEMIA/REPERFUSION.
A. Gasbarrini B. Nardo, P. Pasini, S. De Notariis, A. Mazziotti, A. Cavallari, E.
Roda, P. Pola, M. Bemardi, A. Roda.
Angiology and Internal Medicine, Catholic Universty of Roma; Gastroenterology,
Medical PathololD' and Surgery, University ofBologna, Italy.
Tissue sensitivity to isohemia/reperfiion injury remains a main problem
afflicting liver preservation and tmnsphmtation. Oxygen radicals (OFR) are a
major cause of reperfusion liver injury. Studies on the mechanisms of OFR
production, however, are limited by the difficulty to measure in real time their
formation. Luminescence analysis has been recently proposed to measure OFR
generation in isolated cells or organs, but it allows only global tissue luminescence.
Using a special Saticon ultrasensitive videocamera with image intensifier able to
record the organ live image and to measure the tissue photons emission at a single
photon level, we aimed to visualize and localize oxygen free radical generation by
isolated perfused liver exposed to an oxydative stress.
METHODS: livers obtained by Whistar male fed rats (200-250 g) were exposed to 2
h warm isohemia. Successively, organs were placed in the luminograph apparatus
and perfused with an oxygenated Krebs Henseleit bicarbonate buffer containing 10
mM ofthe anion superoxide (02-) enhancer lucigenin for 60 min at 37o C and at rate
of 25 ml/min. Live image ofthe organ was recorded at the bei'nnin and at the end
of each experiment; chemiluminescence emission was recorded every minute.,
throught the entire experimental phase. The overlay ofthe tissue surface luminescent
emission with the live image of the organ was utilized to measure rate and spatial
distribution ofphoton emission (photons/see/organ surface)..
RESULTS: chemiluminescence was not detectable during isohemia, while it
observed aRer reperfusion. Photons emission started af few minutes ofreperthsion,
was maximal after 15-20 min and disappeared within 50-60 rain.
superimposition of chemiluminesoent and live image Immitted to determine the
regional production rate and distribution ofphotons. Chemiluminescence started from
the region surroundin8 the hepatic vasoulare pediele and diffused progressively to the
perifery. The addition of superoxide dismutase, specific inhibitor of02- formation, to
the perfusate reduced significantly the light emission observed reperfusion.
CONCLUSIONS: the luminescent imaging system here described, showing
possibility to evaluate in real time the rate and the spatial distribution ofoxygen free
radicals formation on the surface ofan intact organ, represents a novel and potent tool
for the study ofthe pathophysiology ofoxidative stress and the pharmaoodynamies o:f
drugs with antioxidant capacity.
87
P069 P070
IS FENESTRATION THE MOST ADEQUATE
OPERATION FOR LONG-TERM MANAGEMENT OF
ADULT POLYCYSTIC LIVER DISEASE (APLD) ?
J.F. Gigot, P. Jadoul, B. Van Beers, J. Etienne,
Y. Horsmans, A. Geubel, J. Pringot, P.J. Kestens
Department of Digestive Surgery, Louvain Medical School
The surgical management of APLD remains controversial
with partisans of fenestration technique or liver resection.
We reviewed our experience with ten patients operated by
extensive fenestration technique. APLD were classified in
3 types according to number and size of liver cysts and
remaining liver parenchyma. The mean preoperative liver
volume was 7761 cm3. There was no mortality.
Postoperative morbidity included intraoperative
hemorrhage (one patient) and biliary complications
(3 patients). The mean postoperative liver volume at
follow-up was 4450 cm with a mean reduction in liver
volume of 43 %. Type and II APLD are excellent
candidates for laparoscopic and open fenestration
procedure respectively. In type III APLD disease
progression was observed in 40 % of the patients, which
mean that fenestration procedure is not the best surgical
approach for this group of patients.
COLOR DOPPLER SONOGRAPHY IN DIAGNOSIS OF PORTAL
HYPERTENSION IN PATIENTS WITH LIVER CIRRHOSIS
E. Goncalvesovi, M. Szntovl1, V. Kupovl1, A. Kov, I'. Jurgo
1 st Department of Medicine Postgraduate Medical. Institute, 3 rd
Department of Medicine1, Medical School of Comenius University1,
Bratislava, Slovakia
Complications of portal hypertension, i.g. variceal bleeding, are the most
lequent causes of hospital morbidity and mortality in patients with liver
cirrhosis. Color Doppler Sonography (CDS) is the only fully nonlm,asive
tool for diagnosis and follow up ofportal hypertension.
Group of 94 patients with liver cirrhosis and endoseopically confirmed
esophageal varices Paquet II-IV, aged 25-74, median 60 years) and 50
healthy controls (aged 25-67, median 51 years) were examined. The
following data were evaluated: portal vein diameter, gall- bladder
thickness, common hepatic artery diameter, maximal spleen size, direction
ofportal blood flow, presence ofportosystemic collateral circulation, peak
flow velocity (PFV) and mean flow velocity (MFV) in the portal vein. The
portal vein was enlarged > 13 ram) in 40 patients 47 % ), gall- bladder
wall was more than 3 mm in 25 pts 29 % ), spleen enlargement > 13 cm
was present in 58 (69 %) patients. Portosystemic collateral circulation
was confirmed 67 times in 54 64 % patients. Hepatofugal flow was
diagnosed in 3 patients only. There was a statistically significant difference
p < 0.001 in PFV 10.22 +/- 3.73 vs. 16.47 +/- 3.37 cm/s and MFV
(5.99 +/- 2.71 vs. 10.4 +/- 2.5 cm/s between patients and controls.
Similarly, a significant difference in common hepatic artery diameter 5.65
+/- 0.95 vs. 4.4 +/- 0.46 mm was confirmed.
We consider the values ofPFV 7 cm/s and MFV 5 cm/s as specific for the
diagnosis of portal hypertension. Dilatation of the hepatic artery is the
result of increased arterial liver blood flow in cirrhosis. From diagnostic
point of view, the unfavorable feature of this sign is that the common
hepatic artery was measurable only in 65 % of cases .Spleen enlargement
exhibited the highest sensitivity. Collateral portosystemic circulation and
excessive deceleration or reversion of portal vein flow seems to be the
most specific ofan ultrasonographic diagnosis ofportal hypertension.
P071 P072
LIVER TRAUMA. MORBIDITY AND MORTALITY OF SURGERY
J.J. Gonzilez, L. Sanz, A. Miyar L., J.L. Grafia, G. Bermejo, A. Blanco,
E. Martfnez
Department of Surgery B. Hospital Central. University of Oviedo. SPAIN
Recent advances in prehospital care have increased our salvage rate but
paradoxically have increased our institutional mortality. We reviewed
our surgical experience with special emphasis on perioperative risk
factors. One hundred and eleven patients presenting hepatic injury were
detected during exploratory celiotomy in the sixteen year period from
January 1979 through December 1993. Their median age was 31 years
(5-84). Blunt tr,auma predominated, with 80 patients (72.1%). Injury
was due to penetrating wounds in 31 patients (27.9%). From a clinical
point of view it is noteworthy that, upon admission, 60 patients
(54.1%) had shock and 8 (7.2%) coma. A total of 124 associated
injuries ocurred among 77 of the 111 patients "Liver sutures" were
placed in 78 patients (70.3%), major resectional debridement was
performed in 15 patients (13.5%), and perihepatic drainage in 18
(16.2%). Secondary hepatic procedures (perihepatic packing, selective
hepatic artery ligation) were required in 16 patients. Uni- and
multivariate analysis was performed to discriminate variables in relation
to mortality and morbidity. 22 patients (19.8%) died and 56 of the 89
survivors (62.9%) had complications. Ten of the 22 patients who died
did not leave the operating room, nine experienced exsanguinating
haemorrhage. The other 12 died in the postoperative period, the main
causes of death being DIC:5, MOF:3, and sepsis: 2. The morbidity was
associated mainly with intraabdominal abscess (19), wound infection
(15) and pulmonary complications (53). Six variables showed influence
on mortality: age, sex (women), shock on arrival, orthopedic
involvement, degree of hepatic lesion and liver resection. Factors
correlating with morbidity were shock, gastrointestinal tract lesions and
hepatic grade. When the variables were introduced in the regression
model, the mortality was related only with sex (p-0.01) and shock
(p =0.007). The morbidity had correlation with shock (p =0.001) and
hepatic grade (p =0.01 ). The single most important factor influencing
final outcome is the presence of shock.
DIC: Disseminated intravascular coagulation
MOF: Multiple organ failure
ENDOVASCULAR EMBOLIZATION IN
HEPATIC TUMOURS.
E.Gouseynov,V.Koubishkin,V.Vishnevsky,D.Sarkisov
,R.Ikramov,A.Adamyan,
A.Tehjao,M.Titova.
Vishnevsky Institute of Surgery, Moscow,Russia.
The results ofretrospective analysis of the treatment of
215 patients with hepatic tumours are presented. 117
of patients had haemangiomes, 81- malignant lesions,
11- hydatid cysts, 6- simple cysts. Endovaseular
occlusion of the tumours were performed in all eases
by superseleetive catheterization of segmental arterial
branches with subsequent occlusion with hydrogel.
The main indication for selective embolization was
hyper vaseularity of lesion. Differents kinds of
hepatic resections were perfomed in 119 pts.
1- 30 days after embolization.The volume of
intraoperative bleeding was significantly reduced in
eomparision with pts.operated without embolization.
In 96 pts.operation after embolization were not
done. All this pts. had small benign lesions or
nonresee table tumours.
Preoperative superselective endovascular occlusion
of the. tumours signifieanly reduced intraoperative
complications, postoperative mortality and
morbidity.It may be treatment of choice in patients
with large nonreseetable hepatic lesions.
88
P073 P074
COMBINED TREATMENT OF LIVER CIRRHOSIS
A.M.Granov, T.L:Pirtz.khalava, D.A.Granov, P.G.Tarazov
Dept of Surgery, St Petersburg Research Institute of
Roentgenology and Radiation Therapy, St.Petersburg, Russia
The aim ofthis prospective study was to evaluate the feasibility
and success of combined treatment lymphosorbtion plus
medical therapy 'vs medical therapy alone for treatment of
advanced cirrhosis.
Group combined treatment consisted of 37 patients with
Child-Pugh Class B 9 or C 28 posthepatitis B liver
cirrhosis. Group II conventional medical therapy included 20
patients Class A- 5, B 12, C 3 ). In Group I, lymph
purification and reinfusion 500 ml to 1500 ml daily was
performed using chronic external surgical catheterization ofthe
thoracic duct. The carbon adsorbent with fibers of 8x10(-3)
mm to 12x10(-3) mm and 2m2/g external geometric surface
was used.
Control ofactivation was achieved in 33 89% vs 11 55%
patients, respectively. Hospital mortality rates were 3% and
15% in Groups and II. In two weeks after beginning of the
treatment, decrease of ascites was seen in 97% and 70% of
patients, respectively. Gastroesophageal varices decreased in
81% vs 10%, and eneephalopathy regressed in 89% vs 30% of
patients.
These data showed that combined treatment including
lymphosorbtion is effective in most patients with advanced liver
cirrhosis.
A DIFFERETIATED APPROACH TO SURGICAL
TREATMENT OF LIVER HEMANGIOMA
A.M.Granov, V.N.Polvsalov. K.V.Prozorovskij, P.G.Tarazov
Depts Surgery & Diagnostic Radiology, St.Petersburg Research
Institute of Roentgenology & Radiation Therapy, St.Petersburg,
Russia
We retrospectively evaluated treatment results in 140 patients
with hepatic hemangioma. Of them, 90 patients had solitary, 40
multiple and 10 diffuse tumor.
Only observation was done in 48 patients with small
asymptomatic hemangiomas. Surgical or interventional
treatment was performed in 92 patients. Hepatic resection was
made in 30 (32%) patients with postoperative death. Hepatic
artery ligation with catheterization and following peripheral
embolization was carried out in 11 cases. Transcatheter
embolization was perfomed in 35 patients including 6 repeated
procedures. Percutaneous injection therapy with sclerosing
solutions was done in 12 patients. Percutaneous or
intraoperative intratumoral injection of ferromagnetic was
performed in 10 cases including 8 after previous arterial ligation
or embolization.
The 3 to 10 year follow-up showed excellent results of hepatic
resection in 28129 of sundvors (97%). Tumor decrease with
fibrosis of hemangioma and symptomatic improvement were
seen in 54% of patients after arterial ligation and in 62% after
transcatheter embolization. Similar effects were seen in 85% of
cases treated by percutaneous injection therapy. The long-term
results of local ferromagnetic treatment are in the future.
These data showed that individual approach is preferrable for
patients with liver hemangioma depending on tumor size and
location, clinical symptoms and benefit-to-risk ratio.
P075 P076
ARTERIAL EMBOLIZATION (AE) IN LIVER
TUMORS COMPLICATED BY JAUNDICE
A.M.Granov, P.G.Tarazov, D.A.Granov
Department of Surgery and Division of
Angio/Interventional, St.Petersburg Research
Institute of Roentgenology & Radiation Therapy,
St.Petersburg, Russia
We retrospectively evaluated the results of AE in 14
pts with hepatocellular carcinoma (8) or liver
metastases (6) complicated by jaundice.
Jaundice of a mixed (obstructive plus hepatocellular)
origin with serum bilirubin (SB) 40 mol/L to 60
mol/L was in 8 patients. AE performed after 2-5 day
infusion therapy resulted in tumor size decrease in 5
and stabilization in 3 cases. The level of SB returned
to normal in these pts. The one patient with SB 95
mol/L and HCC occupying 70% of the liver, died of
hepatic failure 5 days post AE.
Obstructive jaundice with SB 140-400 mol/L was
diagnosed in 6 pts. The first step of treatment was
percutaneous transhepatic biliary drainage (PTBD).
After SB decrease < 60 mol/L, AE was done without
complications. Tumor decrease or stabilization was
seen in all 6 cases.
It is concluded that jaundice should not be
considered absolute contraindication to treatment of
liver malignancy. Level of SB < 60 mol/L is safe for
AE. Pre-AE decrease of SB can be achieved with
PTBD.
DEVELOPMENT OF ECHINOCOCCOSIS DIAGNOSIS AND
TREATMENT OVER A 20 YEAR-PERIOD
S. Gruttadauria, G. Marine, G. Gruttadauria
Department of Surgery, University ofCatania, Italy
The liver is the most frequent seat of echinocoecosis and its involvement during the
hydatid disease in about 70% of We looked experience in this
pathology over the last twenty years regarding the development of its diagnosis and
treatment. From November 1973 to October 1993 performed 89 operations of
liver oehinococus cysts in Unit. Patients divided into two groups: A and B.
Group A and Group B amounted to 65 patients who underwent surgery between
1973 and 1988, and 24 patients who underwent surgery between 1989 and 1993
respectively. We observed remarkable differences in diagnostic procedures between
the two groups of patients. In Group A always performed direct RX of the
abdomen, in 49% of carried out. hepatic seintigraphy, in 28% i.v.
eholangiography in of suspected relation between the cyst and the biliary tract.
In the most performed ultrasonography, CT cavography
with phlebography of suprahepatie veins. In Group B ultrasonography and CT
always carried out, while in performed arteriography only for
anatomic-surgical information and in of suspicious communication with the
biliary tract ERCP performed 4 times.
As far the serologic diagnosis is concerned, always carried out, for Group A
patients, test for hematie eosinophilia and the Casoni intradermal reaction test. On
the contrary, in Group B followed regularly the Elisa method for the antibody
assay and in the follow-up introduced of the ]LAST test. In Group B
introduced the of albendazole in pre-operative period. In Group A,
conservative procedures, performed 41 marsupializations, 20 partial
perieystectomies and radical procedures 4 total perieystectomies. In Group B,
performed 14 total perieystectomies and 10 liver resections. In all Group B patients,
performed the intraoperative ultrasonography during surgery. The post-operative
hospital stay was 30 days for Group A patients, with only death, and 18 for
Group B ones, without any deaths. Recurrences occurred only in Group A (11
equal to 17%), of them treated with further surgery of total perieystectomy
while in cases liver resections performed. The analysis of data shows that
if there are differences in terms of deaths between these two groups,
regards the average post-operative stay, the onset of post-operative complications
and recurrences, there is evidence ofimprovements related to radical surgical
approach. The trend to perform radical surgery is not general rule; in fact
each patient should be treated with different therapy and it is very important that
patients should not be exposed to excessive risks due to the benign nature of this
pathology.
89
P077 P078
CAPTOPRIL SIGNIFICANTLY ENHANCES
ENDOTHELIN AND EICOSANOID RESPONSE IN
HEPATIC ISCHEMIA-REPERFUSION INJURY
Bahadr M.GOLL00LU,A.0zdemir AKTANt,
Cumhur YEMEN1, Hzr KURTEL2, Rfat YALIN
Department ofSurgery and Physiology
Marmara University School ofMedicine, Istanbul, Turkey
The reactive oxygen metabolites (ROMs) and the vascular endothelial
factors such as endothelins (ETs) and thromboxane A2 (TxA2) were found to
be the mediators ofthe reperfusion component ofischemia-reperfusion (I/R)
injury. Captopril (CPT) which is a sulphydryl (-SH) group containing
angiotensin converting enzyme inhibitor has been shown to reverse the I/R
injury by its ROM scavenging effect. In this experimental s{udy, the effect of
CPT and BM 13.177 (a TxA2 receptor antagonist) were assesed on liver I/R
injury in rats.
The study.consisted offour groups ofsham-operated, control, CPT and
BM 13.177 treated Wistar-Albino rats. The middle and left lateral hepatic
arteries and portal veins were occluded in each group but the sham and the
corresponding agents were given to the animals prior to I/R injury. After I/R
injury, blood was drawn from the supra_hepatic vena cava inferior for ET-
like activity assay and the liver tissue samples were obtained for the
determination ofprostaglandin E2 (PGE2), leukotriene C4(LTC4) and
histopathologic examination.
PGE2 and ET-1 levels were increased significantly in the control group
when compared with the sham operated group. In COTgroup, LTC4, PGE2
and ET-1 levels were significantly increased when compared with the
control group while only ET-1 levels were not different than those ofthe
control group in BM 13.177 treated group.
It is concluded that ET-1 release increases in response to I/R injury in rat
liver and CPT further increases this release. It also appears that CPT has a
direct stimulatory effect on arachidonic acid metabolism in addition to its
free radical scavenging effect.
CLINICAL STUDY ON THE TREATMENT FOR SPONTANEOUS RUPTURED
HEPATOCELLULAR CARCINOMA
N.Hanashiro*, M.Furukawa, T.Sakai,
M.Sasaki, K.Miyashita, Y.Mine, Y.Sakamoto, S.Higa, E.Chosa, T.Kusano*,
*First Department of Surgery, Faculty of Medicine,University of the Ryukyus,
Okinawa
Department of Surgery, Nagasaki Chuo National Hospital, Nagasaki, Japan
INTRODUCTION
We analyzed experience with spontaneous ruptured hepatocellular
carcinoma(HCC) to evaluate the long-term benefits of the treatment with second-
stage hepatectomy and transcatheter arterial chemoembolization(TACE).
PATIENTS AND METHODS
From 1980 to 1995, 10 patients treated for spontaneous ruptured HCC
divided into two groups. After the clinical diagnosis of spontaneous ruptured
HCC, hepatic angiography performed, followed by TACE. After TACE, five
patients underwent second,stage hepatectomy( group A and the other five
treated with TACE alone(group B ). In group B, hepatic resection not
performed due to hepatic dysfunction and/or multiple liver metastasis.
RESULTS
Three in group A and three in group B in hemorrhagic shock state
admission. Emergencycomputed tomography and ultrasonography examination
carried out in all patients and demonstrated positive findings in 100%.
Hemorrhage stopped after emergencyTACE in all patients. In group A and
group B, the 1-year survival rate 53.3%, 40.0%, the longest-survival months
29 months, 15 months and the median duration of survival 8 months, 4
months respec ively.
1-year
hemorrhagic tumor
age(year)
shock size(cm)
survival
rate
GroupA (n=5)
65-1-10.6 S.D. 7.6+__1.5 S.D. 53.3%
M 4:F
62.80-8.14 S.D. 6.60-2.3 S.D. 40.4%
Group B (n=5)
M 4:F
*p<0.01
CONCLUSION
In the treatment of patients with spontaneous ruptured HCC, usually have
enough time to evaluate the liver function, because the disease itself demand
emergency treatment.Therefore, it is recommended first to perform emergency
TACE. Second-stage hepatectomy maycontribute to improvement of the
prognosis.
P079 P080
FAILURE OF THE PORTAL VEIN TO BIFURCATE
K.J. Hardy, R.McL. Jones
Department of Surgery and Liver Transplantation, Austin
Hospital, Melbourne, Australia
Failure of the portal vein to bifurcate is a most
uncommon event compared to absence of the right portal
vein which occurs in 20% of individuals. A "patient
undergoing right hemihepatectomy had the abnormality
of failure of the portal vein to divide into right and left
branches.
Materials and Results: The single trunk passed from the
porta deep into the liver, arched convexly to the right and
passed with no superficial left branch. After the arch the
deep section passed transversely to the left and then ran
forward in the umbilical fissure. The anomaly can be
diagnosed on contrast CT or Doppler ultrasound and be
conf'mned by intra-operative ultrasound or by lowering
the liver plate. Should the anomaly not be recognized,
ligation of a supposed right or left portal vein would in
fact be ligation of the entire portal flow. With an intact
hepatic artery the mistake may not be recognized with a
catastrophic post-operative outcome. An uneventful right
hemihepatectomy was performed for our patient.
Conclusion: Failure of the portal vein to bifurcate is a
rare anomaly that must be recognized for safe liver
surgery and can be diagnosed by imaging or by operative
dissection.
RADIOLOGICAL DIAGNOSIS OF HEPATIC
ADENOMA AND FOCAL NODULAR HYPERPLASIA.
P. Herman, V. Pugliese, A.L Montagnini, M.Z. Salem,
M.A.C. Machado, T. Bacchella, M.C.C. Machado, H.W.
Pinotti.
Department of Gastroenterology, Hospital das Clinicas-
University of So Paulo Medical School; Brazil.
Pre-operative diagnosis of benign liver tumors as
hepatic adenoma (HA) and focal nodular hyperplasia (FNH)
is often very difficult. This lesions are rare, generally affect
young women but their pathogeny and outcome are
completely different. Surgical resection of HA is advocated
based on the incidence of bleeding complications, in some
instances lifethreating. Moreover, ....oplastic degeneration
of HA have been reported. In contrast, FNH is often an
incidental finding and complications as hemorrhage or
malignant degeneration are extremely rare or absent, for
this reasons it must be conservatively treated.
The aim of this study was to evaluate 19 female patients
with benign liver tumors (10 FNH, 9 HA), trying to establish
pre-operative criteria for differential diagnosis. In the
present study, all patients were submitted to surgical biopsy
or to a hepatic resection.
Based on clinical and laboratorial data, distinction between
FNH and HA was not possible. With the development of
imaging methods used in combination (ultrasound,
computed tomographic scan, liver scintigrap.hy with HIDA
and sulfur-coloid, magnetic ressonance =maging and
angiography), according to a diagnostic algorithm, the
differentiation was possible in 79% of the cases.
90
P081 P082
Is hepatic resection the best treatment on
hepatolithiasis ?
S. W. Hong., S. M. Lee, H. Z. Joo.
Dept. of Surgery, Kyung Hee University, Seoul, KOREA
Intrahepatic stone (IHS) which mostly pigment stones is prevalent in East Asia,
but in U.S. and Europe.
It makes many complications and necessitates multiple operations interventional
procedures, and also sometimes couldn't be cured though lot of procedures
used.
The to make annoying problems recurrence because of bile stasis,
stricture of bile duct and recurrent infections.
We have studied retrospectively which is the best way to treat the intrahepatic
stones.
In Korea, 10-20% of gallstone disease IHS. We have IHS of 18.6%(465
of 2504 cases) that have preformed operation from sept. 1978 to Dec. 1994 at
Kyung-Hee University Hospital, Seoul, Korea. We did hepatic resection in of
definite intrahepatic bile duct stricture, multiple stones confined to resectable
segment lobe with without parenchymal atrophy, recurred IHS, suspected
malignancy with IHS. Between Jan. 1985 and Dec. 1994, 157 cases(56.9%) of
hepatectomy lateral segmentectomy 112 cases, lobectomy 28 cases, segmentectomy
15 cases, wedge resection case, trisegmentectomy have been performed
276 of hepatolithiasis. 28 cases(17.9%) had residual stones during and/or
after hepatectomy, 24 out of them had stones in the both lobe of the liver.
Balloon dilation and stone removal performed
Only 10 cases(6.4%) have of cholangitis and/or liver abscess within
years after operation but 38 cases(31.9%) in non-resection Postoperative
complications have had significant differences between resection and
non-resection We think aggressive hepatic resection may be the treatment
of choice hepatolithiasis.
COMPARISON IN SURVIVAL BETWEEN HEPATIC
METASTASES OF GASTRIC AND COLORECTALCANCERS
M.Hoshima H.Taniguchi, H.Koyama, H.Tanaka, M.Masuyama,
T.Mugitani, A.Takada, K.Kitamura, A.Hagiwara, T.Yamaguchi,
K.Sawai, and T.Takahashi
First Department of Surgery, Kyoto Prefectural University of
Medicine, Kyoto, Japan
Between April, 1988 and March, 1994, 176 patients with metastatic
liver cancer were treated at the First Department of Surgery, Kyoto
Prefectural University of Medicine Hospital. All patients received
multi-disciplinary treatment, while 51 also underwent hepatectomy.
The hepatic resection rate was 11.5% for gastric cancer and 39.4%
for colorectal cancer. The survival after hepatectomy for metastatic
liver cancer from a colorectal primary (39 patients) was better than
that for gastric cancer (6 patients). Median survivals of these
disease were 1317 and 566 days, respectively. The survival after
hepatic arterial infusion (HAI) therapy for metastases from gastric
cancer (13 patients) was better than that for colorectal cancer (25
patients). Median survivals of these disease were 881 and 648
days, respectively. Surgical resection was the most effective mode
among the treatments for liver metastases from colorectal cancer.
HAI was a better option for liver metastases from gastric cancer.
P083 P084
ROLE OF THE E-CADHERIN CELL-CELL ADHESION COMPLEX IN
METASTASIS OF PRIMARY COLORECTAL TUMOURS TO THE LIVER
,Poston GJ, Kinsella AR. Cellular Ontology Group, Department of Surgery,
University of Liverpool, Liverpool, United Kingdom.
The E-eadherin cell-adhesion complex is made up of E-eadherin, transmembrane
glycoprotein responsible for calcium dependent cell-cell adhesion, and several
cytoplasmic proteins alpha-catenin, beta-catenin, and the product of the tumour
suppressor gene APC. In coloreetal there is strong trend for advanced tumours
to have reduced E-cadherin expression but this relationship is not simply of loss of
expression of E-cadherin facilitating invasion and metastasis. It is probable that genetic
mutations i the cytoplasmic proteins associated with E-cadherin also play role in its
dysfunction. The purpose of this study to document patterns of expression of the
components of the E-eadherin complex in large series of liver metastases from
colorectal primaries. Frozen sections of liver metastases from 46 patients with eoloreetal
primaries were examined for expression of E-eadherin, alpha-eatenin, and beta-eatenin.
These antigens detected by monoclonal antibodies using standard
immunoperoxidase method. There 30 males and 16 females in this series with
median age of 65 yrs (37-83yrs). Complete down regulation of E-cadherin found in
43 patients (95 %) whilst there complete partial down regulation of beta-eatenin
and alpha-catenin in 39(85%) and 36(78%) patients respectively. Expression of all three
components of the E-cadherin complex was seen in only one liver metastasis and the
primary tumour from this patient reported moderately differentiated. In patients
expressing alpha-catenin membranous pattern of staining always seen and in this
group 80% pereent of patients (8/10) also expressed beta-catenin. No obvious correlation
found between the differentiation status of the primary tumours and expression of
any component of the E-eadherin complex.
These preliminary studies suggest there is simple relationship between the expression
of components of the E-eadherin complex and the presence of liver metastases. Down
regulation of cell adhesion molecules may be variable phenomenon which would
account for the variations in expression in study. Correlation with expression of
the E-cadherin complex in the corresponding primary tumours well as differentiation
status, Dukes' stage, and grade of the primary turn/ours is necessary in order to determine
the exact role the E-cadherin complex plays'in the development of liver metastases.
These studies on-going in laboratory in conjunction with molecular analysis of
the complex in order to identify possible mutations in the E-cadherin, alpha-catenin or
beta-catenin genes.
HEPATIC XENO-HEMODIAFILTRATION IN FULMINANT HEPATIC
FAILURE: AN ANIMAL MODEL.
S. Hyon, J. Vazquez, H. Ga'cia, F. Nftez, J. Pekolj, E. de
P. Argibey. Unid de Medicine Experiment. Hospi
Italiano de BuenosAires, Argenlina.
p is lhe ot aonors nnly in lhe a ldminnt
hepalic failure (FHF) because of the Iknin9 of this precedure. Now in
the laboratory our work is in the support of liver funclions
until lrans. We present here a model of liver xenoflerfusion
in an anknal ofF.H.F.
In 6 pigs we pedrned a poao-caval shuntand ligature ofthe hepatic
arlery. In these pigs cordJnuous h a
were cathelerized through the portal vein and perfused with
atologous red blood cells, albumina and in a closed
in FHF and the auxik'ary liver's circulalJon. The auxiliary liver worked
damage. LacEc acid (LA and arnnnia (NH3 rncg/dlt),
improved: LA8.2+-.6to 1.6..1, NH3 487+-11010 117+-13, p<.01 and
the MEGX test (nghnlt), remained at funclional levels, (> 90) at the
end of e .(direct xenotdusion) were
discontinued at 60.x 15 mintdes due Io hyperactde rejeclion. We
.conclude that this system is adequale to sup an animal in FHF
Now we are involved in a clinical lrial I probe this xeno-
91
P085 P086
EXAMINATION OF THE SINUSOIDAL CELL FUNCTION AFTER THE
LIVER RESECTION
S.ISHIHARA, S.MIYAKAWA, A.HORIGUCHI, M.HAYAKAWA,
N.NIWAMOTO, Y.IWASE, S.SATOH, H.YAMAMOTO, K.MIURA
Department of Surgery, Fujita Health University, Toyoake
Japan
We examined a difference of the sinusoidal cell function of
the survival cases and the death cases of hepatic failure.
We examined 20 cases of the liver resection for recentry 2
years. These patients were divided into two groups according
to the condition after operation, first was the survival group
(S group,17 cases), second was death of hepatic failure group
(DHF group,3 cases). We inserted the catheter in the hepatic
vein before operation. We mesured endotoxin (Et)and
hyaluronic acid (HA) as an index of sinusoidal cell function
fro.in the hepatic vein for 7 days after the operation.
Et varues before the operation didn't recognize significant
difference in each group. Highest value of Et after the
operation didn't recognaize significant diffrence in each
group. Et values after the operation gradually decreased in
each group, but didn't return to a value before the
operation. HA values before the operation didn't recognize
significant diffrence in each group. HA values after the
operation were significantly higher in DHF group than in
S group. HA values after the operation gradually increased
to DHF group.
Endocytosis was not the obstacle postoperative seventh day
from Et values chenge. We conclude that sinusoidal
endothelial cell obstacle participated as a couse of heptic
failure after the operation.
CLINICOPATHOLOGIC STUDY OF EARLY-STAGE HEPATOCELLULAR
CARCINOMA AND BORDERLINE LESIONS IN CIRRHOTIC PATIENTS
M. Ishikawa, S. Yogita, H." Miyake, Yo Fukuta, M. Harada, D. Wada, S.
Tashiro
The First Department of Surgery, The University of Tokushima, School of
Medicine, Tokushima, Japan
Objective: Recently,adenomatous hyperplasia "(AH) of the liver has
been considered a earlyneoplastic lesions since AH coexists with hepatocellular
carcinoma (HCC) and contains malignant foci. Object of this study is
to clarify clinical characteristics and surgical results of AH and
early-stage HCC. Patients and Methods: From 1994 to 1995, 36
patiens had surgery for HCC in our institute. Of these, 25 patients
with 41 small HCCs(<3cm)(sHCCs) and one patient with Atypical AH
(AAH) were selected for this study. Three lesions of AH, 5 lesions
of AAH and 2 lesions of AAH with focal malignancy in 7 of 25
patients with sHCCs were found by histological examinations. The
detectability of these lesions on imagings was evaluated. Cumulative
survival and disease-free survival rates were also calculated. Results:
Twenty one patients were diagnosed with Utrasonography( US
incidentally during the follow-up study for chronir disease. In two
patients, was first diagnosed on the basis of elevated serum
alpha-fetoprotein(AFP) and 14 (54%) of all patients had normal AFP
values. In the conventional studies, detection rates of US, CT and
Angiography for sHCCs and borderline lesions were 73% 80%, 34%
10% and 33% 20%, respectively. MRI, intraoperative US, Helical CT,
arterial angiographic CT and portal angiographic CT showed better results
of 70% 20%, 100% 86%, 78% 50%, 60% 50%, and 78% 56%, respectively,
except for CT-A. The six'- month and 1- year survival rates for all
patients were 83% and 66%, while disease-free survival rates at six-
month and 1-year were 83%. and 57%, respectively. A total of 7
patients (27%) had intrahepatic recurrence during a mean follow-up of 9.3
months. Conclusion: For tumors larger than cm in diameter, the
detection rates with various diagnostic modalities were rather high.
However, the differential diagnosis of borderline lesions from sHCCs
could be done only by pathologic studies. Most patients with HCC
and AH have associated liver cirrhosis with viral hepatitis, which
causes multicentric occurrence and high risk of residual cancerous
foci. Early detection of small hepatic lesions and treatment such as
resection or ethanol injection are of critical important in improving
the long-term survival.
P087 P088
BLOOD COAGULATION AND FIBRINOLYSIS AFTER
HEPATIC RESECTION AND EFFECT OF PROTEASE
INHIBITOR
M. Ishikawa, S. Yogita, H. Miyake, Y. Fukuta, M. Harada, D. Wada, S.
Tashiro
The First Department of Surgery, The University of Tokushima, School of
Medicine, Tokushima, Japan
Objective: An abnormality of the blood coagulation and
fibrinolysis sometimes occurs after hepatic resection and leads to a
fatal outcome. Serine protease inhibitors(PI)such as nafamostat
mesilate(FUT) or gabexate mesilate(FOY), have been widely used
in Japan for the prevention and treatment of disseminated
intravascular coagulation(DIC), while these PI have not been given
in clinical use in foreign countries. Therefore, a randomized
prospective control study was done to clarify the changes of
coagulation and fibrinolysis after hepatic resection and
prophylactic effectiveness of PI for DIC. Patients and Methods: Forty
five patients undergoing hepatic resection from 1994 to 1995
were evaluated and divided into the following three groups A:
Administration of FUT (0-5 POD of 120 mg/day n=16), B: Administration
of FOY (0-5 POD of 1200 mg/day, n =23), C: without Pl(n=6). Fifteen
patients who underwent gastric or intestinal resection were used as
controls. Several markers, namely fibrin degradation product(FDP),
thrombin-antithrombin [i] complex (TAT) and plasmin- plasmin
inhibitor complex(PIC) were examined and the relationship between
these markers and cytokines(IL-6, TNFa, HGF) was also examined.
Results Preoperative clinical data, operative time, weight of
specimen and blood loss during hepatectomy were not significant in
three groups. The TAT and HGF levels increased immediately after
operation in 6 DIC cases. FDP and TAT in group A, B and C
significantly increased from 3 postoperative days without an
equivalent increase of PIC. Each 3 patients in group A and B died of
aggravation of DIC, respectively. However, the FDP level of day 14 in
group C was higher than those in group A and B. There was a
tendency of high levels of cytokines in group C. Conclusion :These
results demonstrated a hypercoagulable state occurred after hepatic
resetion. The measurement of TAT was useful to detect early
stage of DIC. HGF was also potential to confirm differential diagnosis of
DIC from hepatic failure. The prophylactic use of PI for DIC
woued be advocated to carry out difficult liver surgery.
LOCALIZED FIBROUS NESOTHELIOHA OF THE LIVER
A.N.Isla,J.Rouco.
Dept.of Surgery Hosp. POVISA. Vigo SPAIN
Localized fibrous mesothelioma (LFH) is
also known as:solitary fibrous tumor,locali-
zed fibroma etc.
LFH frecuently involves the pleura. Only 4
cases affecting the liver have been reported
until 1991.
It seems to origin from a multipotncial
subserosal cell, capable of differenciating
along both mesothelial and fibroblastic lines
Clinical presentation generally is a large
epigastric mass,that swows up a low density
area enhanced by contrast medium on CT.Angio-
graphy shows hipervascularity.HRl appea-
rance has [lot been reported until now.
It can reach a diameteF of 30cm,
and the cut surface generally is grayish whi-
te and firm.Histologically is composed of fi-
bFoblast like cells and short spindle cells.
InmunofluoFescence, inmunochemistry and elec-
tron microscopy are useful in the diagnosis.
The treatment of choice is complete resection.
We report the case of a LFM of the liver in
a 44 y/o female who presented with a mass in
the rigth upper quadFant.CT showed a low den-
sity mass of 12 cm affecting the segments V
and VI.NRI ruled out haemangiona. Rigth hepa-
tectomy was carried out, and the diagnosis of
LFM was reported. Actually 5 years later
there is no evidence of recurrence.
92
P089 P090
PROTECTIVE EFFECT OF CALCIUM CHANNEL BLOCKER
(DILTIAZEM) AGAINST ISCHEMIA-REPERFUSION INJURY OF THE
LIVER
H. Isozaki, K. Okajima, K. Fujii, E. Nomura, H. Ham
Department of Surgery, Osaka Medical College, Osaka, Japan
Elevation of intracellular calcium concentration is thought to be associated
with the cell damage. In the present study, we evaluated the protective effect
of diltiazem, a calcium channel blocker, on isehemia-reperfusion injury of the
liver. (MATERIALS AND METHODS) The liver of male Wistar rat
was isolated and perfused by flow through method. The perfusion started by
the fluid consisted of Krebs-Henseleit bicarbonate solution regulated at 35"
C of temperature, saturated with 95% 02 and 5% CO2, during first 30 min.
Then, 120 min Ischemia was induced by stopping perfusion keeping the
temperature (35 C) of the liver. After the ischemia, the liver was
reperfused for 120 min. Rats were divided into two groups: Group A
(n=10),without adminstration of diltiazem Group B (n=10) with
administration of diltiazem (lmg/kg/hr) which was added intravenously
during 2 hours before operation and to the perfusion fluid. The following
items were examined at 120 min after reperfusion.. Hepatic enzyme and
hyaluronic acid in the perfused fluid, ATP and respiratory function of
mitochondria (RCR) in liver tissue, and level of intracellular calcium
concentration in hepatocytes by X-ray microanalyzer. (RESULTS) 1) The
level of RCR (2.15) and ATP 3.12 n mol/mg dry weight) in the liver
tissue of Group B were significantly higher than that of Group A
(1.41,P=0.01; 2.33n mol/mg dry weight P=0.02). 2) No significant
differences were demonstrated in the level of hepatic enzyme and hyaluronic
acid of the two groups. 3) The calcium concentrations in the mitochondria
(3.02 m mol/kg wet weight) and cytoplasm (2.79 m mol/kg wet weight) of
hepatocytes in Group B were significantly lower than that in Group A
(3.73,P=0.03; 4.08, P=0.03). (CONCLUSIONS) Diltiazem demonstrated
protective effect against ischemic liver injury. This phenomenon probably
caused by maintaining the low level of calcium concentration in hepatocytes
and the mitochonddal function.
ANTERIOR SEGMENTECTOMY WITH PRESERVATION OF
THE ANTERIOR-DORSAL BRANCH FOR MUCOUS
PRODUCING CARCINOMA OF THE RIGHT HEPATIC DUCT
Y. Iwas, S. Miyakawa, A. Horiguchi, M. Hayakawa,
S. Ishihara, N. Niwamoto, T. Satoh, H.Yamamoto,
K. Miura.
Dept. ofSurg, Fujita Health Univ., Toyoake, Japan
Intraductal papillary adenocarcinoma of the bile is a
minimally invasive carcinoma. When this tumor
involves the right hepatic duct without infiltration of
the left hepatic duct, the posterior hepatic branch and
the anterior-superior-dorsal branch, anterior
segmentectomy with preservation of the left hepatic
duct and the anterior-superior-dorsal branch is
indicated to the tumor. We describe our experience
with anterior segmentectomy with preservation of
the left hepatic duct and the anterior-superior-dorsal
branch for mucin-producing intraductal papillary
adenocarcinoma, involving the anterior-inferior bile
duct, and infiltrating to the right hepatic duct. A 58-
year-old female was admitted to our department for
suspicion of the intra hepatic stone Computed
tomography demonstrated dilatation of the right
hepatic duct and isodensity mass in the anterior
segment. ERC showed mucous in the bile duct. The
anterior bile duct was not detected. PTC demonstrated
tumor shadow in the right hepatic duct, development
of anterior-superior- dorsal branch. Hepatic
venography demonstrated the right hepatic vein, the
right inferior hepatic vein, and moreever branch
from the base of the middle hepatic vein. This hepatic
vein was regarded as drainage vein of the anterior-
superior-dorsal subsegment. When this operation is
indicated to the bile duct carcinoma of the right
hepatic duct, it is important to recognized the
condition of the ramification of the bile duct.
P091 P092
Correlation between mutant p53 gene and biological behavior of
hepatocellular carcinoma
K.S. Jeng, B.F. Chen
Department of Surgery and Pathology,
Mackay Memorial Hospital,
Taipei, Taiwan, R.O.C.
To investigate the biological behavior ofthe mutant p53 gene in hepatocellular
carcinoma and to elucidate the possible role ofhepatocarcinogenesis of
mutant p53 gene in the invasiveness and the prognosis ofhepatocellular
carcinoma.
Seventy-nine consecutive patients having received resection ofhepatocellular
carcinoma entered this study. Tissue sections ofreseeted HCC
(deparaffinized and rehydrated from formalin-fixed and paraffin embedded
sections) were incubated with Anti-Human p53 monoclonal antibody and
received immunostain. Scoring ofp53 result without knowing the patient
status was undertaken. Two percent immunoposifivity was regarded as the
threshold value.
The immunopositive rate was 81% (61 out of 79 patients). The result ofp53
immunopositivity, the clinical variables (associated liver cirrhosis,
alphafetoprotein, I-IBV infection, dead, disease free interval, dead, and
survival) and the histological variables (size, capsule, vascular permeation,
grade ofdifferentiation, multinodular, adequate margin, daughter nodule,
rupture, tumor necrosis and hemorrhage) were compared.
From univariate, vascular permeation, adequate surgical margin, grade I,
complete capsule and multi-focal lesions have statistically significant
difference between those with p53 positive and those with p53 negative.
From multivariate analysis, vascular permeation (78.1% vs 40.0% p=0.0088,
O.R. (odds ratio)=5.357), grade (1.6% vs 20.0%, p=0.0203, O.R.=15.750),
complete capsule (20.3% vs 46.7% p=0.0496, O.R.=0.316), multi-focal
lesions (60.9% vs 33.3%, p=0.0527, O.R.=3.120), dead (62.5% vs 33.3%,
p=0.0683, O.R.=2.923) and disease free interval (8.3M vs 39.1M, p=0.0107,
O.R.=4.900) have statistically significant difference.
Our results suggest that the biological behaviors ofmutant p53 gene are
strongly related to the invasiveness ofHCC. We recommend the
immunopositivity ofmutant p53 gene may predict the prognosis ofHCC in
selected patients.
PARASITIC DISEASE (Hydatid cyst and ascaridiasis)
AS A COUSE FOR OBSTRUCTIVE JAUNDICE
Joksimovic N, Serafimoski V, Neskovski M, Milosevski M.
Clinic ofGastroenterology Skopje, Macedonia
Examination was made on 25 pts.with sings of
obstructivejoundice, under suspection ofintrabiliary
rupture ofhydatid cyst were further investigated by another
US (ultra sound) examination and/or ERCP, and 3 pts.with
"strange bodies" like long tubes placed in common bile duet.
During dynamic watching slow movements ofthe parasites
had been seen.
US showed cystic liver lesion communicating with
biliary tree and enlarged gallbladder in 22 pts., while ERCP
was diagnostic in 14 pts. (19 pts. underwent surgery). All 3
pts.with corpora aliena in common bile duct were showed
by US and ERCP. From the parasite invastigation ofthe
feces ascaris lumbricoides was isolated. Aider that the
patients were put on the corresponding antihelmintic therapy
with Mebendasol. On the next echotomography check-up
after two.weeks the obstructive syndrome were solved,andthere
were no signs for obstruction.
These obstructions could be diagnosed in the easiest
and quickest way by means ofiltrasonography, and also to
foloow up the evaluation.
93
P093 P094
ESTROGEN- (ER) AND PROGESTERONE RECEPTORS (PgR) IN
HEPATOCELLULAR CARCINOMA (HCC)
S. JonLs, W.O. Bechstein, N. Kling, Th. Steinmueller,
O. Guckelberger, H. Lobeck, K. Hecker, P. Neuhaus
Departments of Surgery and Pathology, Vimhow Medical Center,
Humboldt University, D-13353 Berlin, Germany
A survival benefit of the antiestrogen tamoxifen was observed in non-
resectable HCC and in 60% of breast cancer patients with ER(+) tumors
and up to 75% if the tumor was also PgR(+). Data on the female sex
hormone receptor status in HCC are scarce, hardly checked for their
clinicopathological relevance, and refer almost exclusively to Japanese
patients. From 4/1992 to 12/1993, 34 hepatic resections for primary HCC
were performed. Cancerous tissue for cytoolic preparation and receptor
quantification in a monoclonal solid-phase enzyme immunoassay was
obtained from 28 patients undergoing resection, 3 additional patients of
the liver transplantation (LTX) program and 2 patients suffering from non-
resectable HCC. A receptor status was assessed positive for ER- and
PgR-levels exceeding 1.9 fmol/mg protein. ER and PgR could be detected
in the HCC of 13 (39.4%) and 6 patients (18.2%), resp. A lower age was
observed among the female patients, when their receptor status was
negative for ER or PgR or both when compared to the respective receptor
positive groups; a tendency that reached statistical significance in PgR(-)
women (PgR(-): 53.5+/-17.5 years vs. PgR(+): 72.7::2.6 years; p<0.05).
Liver cirrhosis was preexistent in 16 patients (48.5%) with an even
distribution among the different groups. No significant differences with
respect to tumor stage and grading could be observed. The most common
histopathological pattern was the trabecular type, while the fibrolamellar
variant occurred exclusively, though not statistically significant, in ER(-)/
PgR(-) HCC. There was one perioperative death (3.2%). Patients
undergoing LTX, explorative laparoto-my or non-radical resection were
omitted for survival analysis. 1-year-survival in the ER(+)-group was
significantly lower than in the ER(-)-group (40.0% vs. 78.6%, p<0.05).
Though not statistically significant, the 2-year-survival rates in the ER(+)-
and ER(-)-groups were 40.0% and 71.4%, resp. A comparable trend did
not become evident for PgR(+) and PgR(-) patients. The reduced survival
in the ER(+)-group may emphasize the putative potential of tamoxifen in
an adjuvant regimen after resection of HCC, all the more as the female
subgroup tended to be older than ER(-) patients.
Case reports 1) Anterior segnientectomy of the liver with re-
construction of middle hepatic venous tributary, and. 2) extended
hepatic resection of segment 8 associated with resection of a part
of the right hepatic vein.
u__.1), Makuuchi M.2), Takayama T.2), Kosuge T.3), Kawasaki S.4),
Miyagawa S.4), Nakazawa y.4), Kubota T.4), Kubota y.1), Maeshiro K.1),
Inoue T.l ), Ikeda S. 1)
First Department ot'Surgery, Fukuoka University, Fukuoka, Japan.
2) Second Department ofSurgeLv, ?bkyo University, Tokyo, Japan.
3) The Department ofgurgery, National Cancer Center Hospital, Tokyo, Japan.
4) Ftrst Department ofSurgery, Shtnshu University, Nagano, Japan.
Background When liver malignancy has invaded hepatic vein,
decisions regarding resection and reconstruction
necessarily concern.
Patients Case was 57-year-old male with two hepatocellular
carcinomas located in segments (S) 8 and 5. The plasma retention rate of
indocyanine green at 15 minutes (ICGR15') 18.2 %. We carried out
anterior segmentectomy with the thick hepato-venous tributary draining all
of $6. Following reperfusion, $6 was swollen and became dark purple in
color. In this case, reconstruction of the tributary performed because
considered resection of $6 tbllowing anterior segmentectomy might
produce liver/hilure.
Case two 64 -year-old female. A metastatic liver tumor
located in $8 and had invaded to the ventral of $7 over the right
hepatic vein (RHV). The right lobe 70.2 % CT volumetric
examination. However, draining of $6 and $5 by the inferior right hepatic
vein (IRHV) was confirmed by intraoperative ultrasonography, qherefore,
to prevent postoperative liver failure following right lobectomy,
carried out systematic resection of $8 and the ventral of $7
associated with resection of a part of the RHV.
Results In one, $6 liver remnant congestion disappeared
immediately following reconstruction of the middle hepatic
tributary. In two, although very slight congestion of liver remnant
found, the postoperative course uneventful and without jaundice.
Conclusion Obvious congestion may necessitate hepatic venous
reconstruction. However, if liver function and the volume of liver remnant
considered adequate to prevent postoperative liver dysfunction,
resection of part of the RHV is acceptable.
P095 P096
EFFECT OF LONG-TERM ENDOSCOPIC
SCLEROTHERAPY ON PORTAL HEMODYNAMICS IN
ADVANCED LIVER CIRRHOSIS
N.J.Kalita, K.I.Boulanov, A.N.Bouriy
Institute of Clinical and Experimental Surgery, Kiev, Ukraine
Endoscopic sclerotherapy (ES) of bleeding esophageal varices
proved to be the treatment of choice in patients with advanced
liver cirrhosis. Changes of portal hemodynamics caused by ES
were studied in 30 cirrhotic patients (Child-Pugh class B 14,
C 16), who had bled from large (> 5 mm) esophageal
varices. All of them were assigned to elective long-term ES,
which consisted of combined intra- and p.aravariceal injections
of 3% sodium tetradecylsulfate using free-hand technique.
Sessions of ES included 3-5 injections performed every other
day and were repeated every 6 month. Complications were of
minor importance. Portal venous flow (PVF) was measured
using duplex Doppler system before and after sessions.
Patients' .follow-up ranged from 12 to 36 months. Mean values
of PVF showed evidence of significant increase after ES from
437,0 to 580,2 ml/min (P<0,05), but demonstrated a tendency
to reduction before the next session. Complete obliteration of
esophageal varices was observed after 4 sessions of ES in 18
patients (60%). At the end of the follow-up, PVF in this group
was higher compared with that in patients with persistent
varices (591,0 versus 476,6 ml/min). Considerable decrease of
blood ammonia nitrogen level confirmed effective interruption
of gastroesophageal collateral pathways. Thus, long-term ES
in patients with advanced liver cirrhosis produces significant
stable rise in PVF, providing that complete eradication of
esophageal varices is achieved.
THERAPEUTIC INDICATION FOR SMALL HEPATOCELL
CARCINOMA
H. Kasugai, A Inoue, T. Matsunaga, I. Kaji, T. Otani,
Department of Gastroenterology, Osaka Medical Center for Cancer and
Cardiovascular Diseases, Osaka, Japan
The best way of therapy for small hepatocellular carcinoma (sHCC) equal to or
less than 2 can in diameter was investigated.We experienced 185 (17%) sHCC
cases among 1084 HCC cases from 1982 to 1993 in our institute. Therewere 142
male and 43 female patients. Age distributed 31 to 85 years old (mean 60 y.o.).
One hundred and eighteen cases (64%) had solitary and 67 (36%) had multiple
lesions. The first therapy were resection in 104 cases (56%), pereutaneous ethanol
injection (PEI) in 21 (11%), transcatheter arterial embolization (TAE) in 49
(26%) and no therapy in 11 because of liver dysfunction. Each of 3- and5-year
cumulative survival rates were 86% and 55% in resected eases 76% and 57% in
PEI eases, 52% and 35% in TAE cases, respectively. In solitary lesion group,
each of3- and5-year cumulative survival rates were 89% and58% in reseeted eases,
80% and 53% in PEI cases, 66% and 37% in TAE cases. Each of 3- and 5-year
cumulative disease flee survival rotes were 48% and 20% in reseeted cases 33% and
8% in PEI eases, respectively. These data indicate that survival rate in PEI group
is fairly well considering that PEI had been performed mainly for inoperable eases.
PEI is easy to be done, less risky, cheap, repeatable for recarrent lesions andneed
shorter admission. Indication of PEI can be widened to operable HCC cases in
addition to inoperable cases, especially for sHCC with some risk for surgery.
94
P097 P098
PORTAL ANGIOECHOGRAPHY USEFULNESS IN
LOCALIZING HEPATOCELLULAR CARCINOMA
S.Katagiri, K.Takasaki, M.Tsugita, M.Yamamoto, T.Ohtsubo, K.Akiyama,
A.Saito,
Department of Surgery,Institute of Gastroenterology, Tokyo Women's
Medical College,Tokyo,Japan
(Purpose) As for the pre-operated examination, it is very difficult to
determine the localization of Hepatocellular Carcinoma. Portal
Angioechography is a method of enhancing ultrasonographic images by the
injection of carbon dioxide gas into a branch of portal vein. We studied the
localization of Hepatoc611ular Carcinoma by Portal Angioechography.
(Material) We used in 83 cases,101 nodules of Hepatocellular Carcinoma to
determine the localization of tumor.
(Method) A 22-gauge needle for percutaneous transhepatic cholangiography
was inserted into an intrahepatic portal branch under ultrasonic guidance.
Ultrasonography was perfomed after the injection of 10-15 ml of CO2 gas
into the branch. Gas was injected into the anterior segmental branch in 70
cases,the posterior segmental branch in 10 cases and the left lobe branch in
cases. The accuracy of these determinations was confirmed at operation. We
studied the accuracy of Portal Angioechography, Ultrasonograhy, Computed
Tomography.and Angiography.
(Result) The accuracy of the localization was Portal Angioechography
89.1% (90/101), Ultrasonography 76.2% (77/101), Computed
Tomography 65.2% (58/89), Angiography 71.4% (55/77). The
misdiagnosis cases of Portal Angioechography were 11 cases (10.9%). The 8
cases were the localization of lower anterior and posterior segment borderline
(so-called $5/$6). The cases were the localization of upper internal and
anterior segment borderline (so-called $4/$8).
(Conclusion) Portal Angioechography is very useful for the localizing of
Hepatocellular Carcinoma. It is careful to determine the localization of lower
anterior and posterior segment borderline (so-called $5/$6).
SIGNIFICANCE OF HEPATIC RESECTION FOR
HEPATOCELLULAR CARCINOMA PRESENTING WITH BILE
DUCT TUMOR THROMBOSIS
Hong Jin Kim, Ki Whan Song, Sung Su Yun, Yong Sung Jeon,
Min Chul Shim and Koing Bo Kwun
Department of Surgery, Colleage of Medicine,
Youngnam University, Daegu, Korea
Hepatocellular carcinoma(HCC) is a common malignant tumor in
korea, as in the south-east asian country. HCC presenting with
obstructive jaundice(so-called icteric-type hepatoma) is
uncommon and it's causes are known to be tumor thrombi,
hemobilia, tumor compression, or tumor infiltration. Few
published reports on HCC with bile duct tumor thrombi exist
and its prognosis has been reported to be very poor. And so
most of treatment used to be palliative procedure such as
T-tube decompression, hepatic artery ligation or transartefial
embolization. But occasionally, curative resection can be offered
if the disease is still localized.
From June 1986 to July 1995, we managed 90 HCC patients by
hepatic resection. Of them six patients were presented with
obstructive jaundice due to bile .duct tumor thrombi. We
performed curative hepatic resection with Roux-en-Y
hepaticojejunostomy in two patients and curative hepatectomy
with tumor thrombectomy and T-tube drainage in two patients
and in the remaining two patients we performed only tumor
thmmbectomy with T-tube decompression. Only two patients
who underwent curative hepatectomy with Roux-en-Y
hepaticojejunostomy are alive more than two years after
operation until now. In conclusion, the aggressive surgical
approach including bile duct reconstruction for the HCC with bile
duct tumor thrombi should be considered for long term survival.
P099 P100
LIVER TRANSPLANTATION IN KOREA
S.T. KIM M.D., Ph.D., FACS
Department of Surgery, Hallym University, Seoul, Korea
Even though the liver transplantation(LTX) has been
accepted as the treatment modality of the end stage liver
disease in western countries, there are many problems to
perform LTX in Korea. Among them, most Koreans are
basically occupied with Confucianism as a religion.
Until now the brain death has not been officially accepted,
and the medical insurance does not cover the expenses for
the eenal organ transplantation presently. In 1988, the
first successful LTX was done by the author on 13 year
old girl with Wilson's disease. She is still alive. After
that, the favorable public response to transplantation from
the brain death patient has been induced. Fifty six cases
of LTX were done at 7 organ transplantation centers by
the end of .May 1995. Orthotopic LTX was 49, reduced
LTX was 5 and living related segmental LTX was 2.
During the early period, poor success rate was faced. It
may be due to high incidence of liver cirrhosis and
hepatoma, and high prevalence of hepatitis B viral
markers in recipients. Living related or reduced LTX has
gradually increased in number. In conclusion, legal
approval of brain death, careful recipient selection and
financial support by medical insurance will facilitate our
LTX outcome in the near future.
95
Study of EGF on Hepatic Tissue PGE2 and hepatic blood flow in Rats
using Acute Hepatic Failure Model
M.Komaba, S.Tanaka, H.Kamoshita, K.Kuga, K.Toshima, M.Uematsu,
F.Imai and G.Toda.
Dep of Internal Medicine I, The Jikei University School of Medicine
(AIMI We reported that the administration of EGF kept the hepatic
blood flow and protected hepatic cell necrosis in animal model of acute
hepatic failure. The volume of hepatic tissue PGE2 that participate
with hepatic cell regeneration and cytoprotection was measured.
IMETHOD EGF was administrated at 20/g/kg.Bw.iv, on male SD
rats about 250g in weight (injection group).After 30 minutes, D-
galactosamine (D-gal) was injected at lg/kg.Bw.ip. Insted of EGF, the
same volume of saline was administrated befor D-gal injection (non
injection group). The volume of hepatic tissue blood flow (HTBF) and
Hepatic tissue PGE2 were measured by semiconductor laser Doppler
flowmeter and PGE method (RIA) befor D-gal injection (n=5), after
30minutes (n=5), and 48hours (n=5). Blood sampling were collected
from infedor vena cava at same times. IRESULT) In non treatment
group, serum ALT and HTBF were 51.6+12.31U/I and 460+/-54.9mv.
In EGF non injection group, those were 43.0:1:5.9 and 473.6:1:49.6 at
0 minute, 45.6:1:5.5 and 456.4:1:16.7 at 30minutes, 7614:1:1536 and
220.0:i:23.7 at 48 houres. In EGF injection group, those were 39.4:1:
6.8 and 630.0:1:76.5 at 0 minute, 42.8:1:6.6 and 541.2+/-27.6 at 30
minutes, 5200:1:1812 and ?96.8+/-30.7 at 48 houres. Serum ALT at
48 houres in injection group was significantly (P<O.05) lower than
that corresponding value in non injection group. HTBF at 48 houres in
injecton group was signifi- cantly (P<O.01) higher than that in non
injection group. Hepatic tissue PGE2 was 0.26+/-O.06Pg/w.w.mg in
non treatment group. In EGF non injection group, those were O.19:!:
0.13 at 0 minute, and 0.24:1:0.18 at 30 minutes. In EGF injection
group, those were 0.94:1:0.20 at 0 minute, and 1.87+/- 1.06 at 30
minutes. That value at 0 minute and 30 minutes in injection group
were significantly (0 minute P<0.001, 30 minutes P<0.002)
higher than that in non injection and non treatment group.
ICONCULUSION] The results suggested that cytoprotection and
maintaining hepatic blood flow of EGF were induced by PGE2.
P101 P102
PROGRESSIVE DECREASE IN TISSUE GLYCOGEN CONTENT IN
RATS WITH LONG-TERM CHOLESTASIS
L.Kr/henb0hl*, J.Reiehen,C.Talos,S.Kr-henbii-hl+.
Departments ofVisceral Surgery* and Clinical Pharmacology,Inselspital,
Berne/Switzerland and Clinical Pharmacology and Toxicology, University
Hospital, Zurich/Switzerland
Aims: To investigate glycogen metabolism in liver and skeletal muscle from
rats with long-term cholestasis induced by bile duct ligation
Methods: Ligation ofthe common bile duct (BDL, n=6) or sham-operation
(CON, n=6) for or 4 weeks. CON pair-fed to BDL, rats studied in the fed
state. Glycogen as glucose (Anal Biochem 1968;25:486), glycogen synthase
(SYN) and phosphorylase (PHOS) by incorporation ofradioactive substrates
into glycogen (Hepatology 1991;14:1189), glucagon by RIA and glucose
enzymatically. Stereological analysis ofthe livers (Hepatology 1987;7:457).
Results: One week after surgery, the hepatic glycogen content was 16.9+8.1
vs. 26.2+11.0 mg/g liver wet weight or 28.8+13.8 vs. 38,6+16.4 mg/ml
hepatocytes in BDL and CON, respectively (both differences n.s.). Total
activities and active forms of SYN and PHOS expressed per ml hepatocytes
were both reduced in BDL vs. CON. Skeletal muscle glycogen was not
different between BDL and CON. Four weeks after surgery, the hepatic
glycogen content was 9.3+7.8 vs. 33.1+4.8 mg/g liver wet weight or
20.5+14.2 vs. 52.9+6.4mg/ml hepatocytes in BDL and CON, respectively
(both differences p<0.01 BDL vs. CON). Total activities and active forms of
SYN and PHOS were both reduced in BDL vs. CON. Skeletal muscle
glycogen content (soleus) was 0.40+0.21 vs. 1.11+0.34 mg/g wet weight in
BDL vs. CON (p<0.01). Plasma glucos.e concentrations were decreased and
glucagon concentrations increased in BDL rats at both time points. There
was a positive correlation between the volume fraction ofhepatocytes (a
measure ofliver damage) and the glycogen content per ml hepatocytes and a
negative between the plasma glucagon concentration and SYN activity.
Conclusions: Chronic cholestasis leads to a progressive decrease in the
hepatic and skeletal muscle glycogen content which is metabolically
significant as indicated by hypoglycemia. Both the decrease in SYN and the
correlation between glucagon and SYN suggest that the decrease in the
tissue glycogen content in BDL is due to impaired glycogen synthesis.
P103
FOCAL NODULAR HYPERPLASIA AND PORTAL VEIN
THROMBOSIS CASE REPORT
Kupovd V. Szntovi M., Goncalvesovi E., Belan V., Tureck L.,
Makaiovi L, Synak R.
3 rd Dept.of Medicine, Dept. of Nuclear Medicine, Dept. of
Biochemistry, Medical School of Comenius University, 1st Dept. of
Medicine, Postgraduate Medical Institute, Bratislava, Slovakia
Focal nodular hyperplasia (FNH) is a relatively rare benign focal liver
lesion. It is characterized by presentation ofthe central scar.
We describe a case of a 40- year- old woman with chronic persistent
hepatitis who had an episode ofvariceal bleeding four years after diagnosis
established. A round tumor ofthe lobus quadratus with a diameter of 6 cm
and splenomegaly were found by ultrasonography. Color doppler
ultrasound (CDU) revealed incomplete portal vein thrombosis, which
seemed to have been the cause of variceal bleeding. Due to signs of
hypersplenism splenectomy was performed and then she was submitted to
sclerotherapy with eradication of varices. Since liver tumor was not
evaluated during operation, CT and NMR were provided. Based on CT, the
diagnosis of adenoma or FNH was suggested. NMR could not exclude
hepatocellular carcinoma. However, normal alfa- fetoprotein values were
not indicative of a malignant origin. Dynamic liver scintigraphy with 99
To-colloid showed the typical image for FNH. Core needle biopsy from
the lesion confirmed this diagnosis.In the literature no case of simultaneous
portal vein thrombosis and FNH has been described as yet and the
association ofthe two conditions remains still vague.
Concluding, we would emphasize
1. the efficacy ofCDU for the diagnosis ofportal vein thrombosis, and
2. the importance of 99 Tc-eolloid seintigraphy for setting up the diagnosis
ofFNH when the typical central star does not occur; it can demonstrate the
oceurenee of Kupfer cells in the lesion and thus differentiate it from
adenoma, carcinoma or metastases.
GALACTOSE ELIMINATION CAPACITY AND PROTEOSYNTHETIC LIVER
FUNCTION IN LIVER CIRRHOSIS WITH PORTAL HYPERTENSION AND IN
PATIENTS AFTER LIVER RESECTION.
V. Kupovfi, L. Tureclo), M. Szfintovfi, J. Belfiek
III. Dept. of Internal medicine, Inst. Med. Chemistry., Biochemistry and Clinical
Biochemistry., Med. School of Comenius University, Department of Surgery,
D6rer's hospital, Bratislava, S o v a k a
Galactose in organism is being metabolized predominantly in the liver, in the
cytosol of hepatocyte. Galactose elimiriation determination expresses the total
liver capacity of galactose phosphorylation, which is the limiting step in
galactose metabolism and its elimination. We used examination of galactose
elimination capacity (GEC) after intravenous administration of a 20 % galactose
solution. After enzymatic determination of galactose concentration in six
samples of venous blood GEC was calculated. At the same time we evaluated
also proteosynthetic function of the liver, determining the albumin (Alb),
transferrin (Tf), prealbumin (PA).levels, cholinesterase (CHE) activity and
prothrombin time (Proth). Patients with histologically confirmed liver cirrhosis
with different severity of portal hypertension, patients after resection of the
liver and healthy persons were investigated. The group of patients with liver
cirrhosis was divided in subgroups according to the compensation of the
illness: Ci (compensated cirrhosis hepatis) and Ci d (decompensated
cirrhosis hepatis) and in groups according to Child-Pugh criteria (Ci A, Ci B
and Ci C). In patients with liver cirrhosis and patients after resection ofthe liver
mean values of GEC in comparison with healthy control subjects were
significantly lower. The mean levels of GEC betveen the subgroup Ci and Ci
d were statistically significantly different. Having divided the cirrhosis group
according to Child-Pugh criteria, the most marked decrease of GEC was found
in the group Ci C, the least marked one in the group Ci A. There were
significant differences between the groups Ci A and Ci B, but not between the
groups Ci B and Ci C. In liver cirrhosis the GEC assessment enabled to judge
appropriately the amount of hepatic tissue reduction which does not necessarily
correspond with the grade according to Child-Pugh classification. In the Ci
group, the evaluation of serum PA and CHE levels showed the best
differentiation of the Child-Pugh severity groups. Determination of GEC in
patients with liver cirrhosis makes the evaluation of the functional state of the
liver possible it is a quantitative test of liver function. This test could be
considered a quantitative index of functional mass of liver parenchyma. The
method of combined testing of GEC and proteosynthetic liver function, that we
propose, enables better differentiation of the severity and degree of liver
insufficiency. In patients with liver cirrhosis the most appropriate way is the
usage of the dynamic evaluation of GEC. Long term follow-up of GEC can be
used to advantage in patients with liver cirrhosis for prognostical criterion of
survival, as well as for the course of disease severity.
96
P104
ALPHA-FETOPROTEIN VARIANTS IN PATIENTS WITH
LIVER TUMORS AND CHRONIC LIVER DISEASES.
V. Kupovfi, L. Turecl, M. Szfintovfi, E. Uhlikovfi
III. Dept. of Internal medicine, Inst. Med. Chemistry, Biochemistry. and Clinical
Biochemistry, Med. School of Comenius University, Bratislava, S o v a k a
Alpha-fetoprotein (AFP) is a fetal protein which is being formed during the
fetal development period of the embryo but after birth the serum level falls
rapidly. Production of AFP is not confined to fetal and malignant cells, the oval
and intermediate cells formed in the course of liver tissue .regeneration also
posses this capacity.
The AFP levels were measured by radioimunoassay. The afinity
chromatography on Con A-Sepharose was used for separating variants of AFP.
The concentrations of Con-A reactive AFP and Con- A nonreactive AFP were
examined in the sera of patients with chronic liver diseases (steatofibrosis
hepatis, chronic active hepatitis, liver cirrhosis), in patients with liver tumors
(primary hepatocellular carcinoma, liver metastases) and .in control group. The
diagnosis of liver diseases was confirmed by histology. The mean values of
AFP in the patients with chronic active hepatitis, liver cirrhosis, liver
metastases and primary hepatocellular carcinoma were significantly higher than
in the control groupin patients with steatofibrosis hepatis. Patients in the active
period of the disease showed significantly higher serum AFP levels than
patients in the ifiactive period. The highest AFP concentration were found in the
patients with hepatocellular carcinoma, mean values in this group were
significantly higher than in the patients with liver metastases and patients in the
other groups. The mean values of the concanavalin A non-reactive fraction of
AFP of the examined groups did not differ significantly. This indicates that
estimation of AFP variants in patients with liver diseases may not provide useful
information additional to that provided by the values of total serum AFP. The
different elevation in the concentration of alpha-fetoprotein in the serum of
patients with primary and secondary tumors of the liver can be useful in
differential diagnosis of the type of tumorous affection Of the liver. Long-term
follow-up of alpha-fetoprotein serum concentrations can be used to advantage in
patients with cirrhosis of the liver for early diagnosis of primary hepatocellular
carcinoma developing in a cirrhotic tissue. The higher levels of alpha-
phetoprotein determined in the serum of patients with chronic active hepatitis
and liver cirrhosis presumably reflect regeneration of the liver parenchyma in
these pathological processes.
P105 P106
LONG TERM ASSESSMENT OF LIVER BIOTRANSFORMATION
FUNCTION AND ITS RELATION TO PROTEIN METABOLISM IN
LIVER CIRRHOSIS WITH PORTAL HYPERTENSION
V. Kup6ov, L. TureclQ, M. Szntov, J. Volmut
III. Dept. of Internal medicine, Inst. Med. Chemistry, Biochemistry and Clinical
Biochemistry, Med. School of Comenius University, Bratislava, S o v a k a
The liver plays a dominant role in disposition of the majority of drugs. There is
now clear evidence, that the pharmacokinetic disposition of a large number of
drugs is abnormal in patients with liver diseases. The most important place of
drug biotransformation, is the hepatic microsomal oxidative enzyme system
(m.e.s.) localised in the smooth endoplasmic reticulum of the hepatocyte.
Theophylline (TH) and antipyrine (AP) drugs metabolized by m.e.s, were
selected for this study. Since our model substances (TH and AP) are
metabolized in the liver by the m.e.s., their elimination rate is an indicator of
this system's functioning and estimation of their clearence and half-life (T 1/2)
could provide a clinically useful functional assessement ofthe degree ofhepatic
damage. TH levels were determined by HPLC method. AP levels were
determined by gas chromatography. Mean T 1/2 of TH and AP in patients with
histologically confirmed liver cirrhosis were significantly longer than in the
control group. Dividing the cirrhosis group according to Child-Pugh criteria (Ci
A, Ci B andCi C), the prolongation rate ofT 1/2 TH and T 1/2 AP depended on
the Child-Pugh groups severity. The most marked prolongation ofT 1/2 TH and
T 1/2 AP was present in the group with most severe portal hypertension and
liver damage (Ci C), the least marked prolongation ofT 1/2 TH and T 1/2 AP
was found in the group with the slightest liver damage (Ci A). The mean levels
of T/2 TH and AP between the groups Ci A, Ci B and Ci C were statistically
significantly different. Serum protein concentrations in the examined groups
were not influenced in the same way. By evaluating the proteosynthetic function
ofthe liver, we found out that prealbumin and cholinesterase are more sensitive
indicators of liver function than e. g. albumin prothrombin time and
transferrin The differences of the mean levels of prealbumin and
cholinesterase between the group Ci A, Ci B and Ci C were statistically
significant. In the liver cirrhosis group statistically significant correlation
between investigated biotransformation and proteosynthetic function was found.
We came to conclusion, that the evaluation of T 1/2 of TH, or T 1/2 of AP
with serum prealbumin level and cholinesterase activi.ty can be very good
markers ofliver function and the degree of liver insufficiency.
ULTRASOUND AND X-RAY GUIDED PERCUTANEOUS
INTERVENTIONS FOR TREATMENT OF HEPATIC
HYDATIDE CYb'WS
N.M. Kun, S.A. Dadvani, A:.N., Lotov, V.N. Andrianov, V.N.
Avakian, G.Ch. Musaev.
Surgical Clinic No 1, Moscow Medical Academy, MOSCOW,
RUSSIA.
Surgical treatment of liver hydatide cysts is still considered as a
highly risky operation due to its great mortality rate and serious postop-
erative complications such as parasites dissemination.
Between 1990. 1995 years 5 patients with hydatide cysts, ofthe liver
underwent percutaneous transhepatic drainage. Moreover, from 26
patients who were examined intraoperatively 3 cases were taken for
percutaneous draining because of the presence of multiple cysts or/and
their deep localisation in liver parenchyma.
A complex set of diagnostic measures including serologic tests and
ultrasonography in majority ofcases provided sufficient information to
diagnose hydatide cysts, their localisation, quantity, maturity levd and
to differ them from the other hepatic lesions. Ultrasound and X-ray-
guided percutaneous diagnostic punction and drainage were performed
simultaneously using multipurpose pigtail catheter /"COOK", Den-
mark/. Complete aspiration of cyst was followed by triple sanation of
its cavity with 20% saline solution. The detachment of chitinc mem-
brane and its partial discharge through the drainage tube were observed
everyday during routine ultrasonic control. The sanation of cyst cavity
with hypertensive saline solution was repeated in %8 days. Discharged
fragments of chitinic membrane were taken for pathomorphologic in-
vestigation. Complete discharge of chitinic membrane was achieved
after the substitution of drainage tube to a lager one. After receiving the
cytological confirmation about the absence ofliving parasites sclerosing
therapy with 96% ethanol was started. Diameter of cysts drained by us
was ranging from 56 to 98 mm and only in one case residue of cyst was
observed in subcutaneous tissue which was due to early substitution of
draining tube. Early postoperative complications were not observed
and long-term follow-up from 6-48 months revealed no residual cases.
However, this problem require further investigations.
P107 P108
CURRENT DIAGNOSIS AND SURGICAL MANAGEMENT
OF LIVER TUMORS
Ladny J.R., Snarska J., Sokolowski Z., Szymczuk J., Puchalski Z.
Department of General Surgery, Medical Univeristy of Bialystok, Poland
Every year increases number of diagnosed and operated on liver
tumors. It is related with technical improved diagnostic methods and
improved operating procedures.
In the authors Department between 1978 and 1995, 155 patients
underwent surgery for various liver tumors. In 96 cases they were
hepatic carcinomas, in 25 cysts (20 nonparasitic and 5 parasitic),
in 12 patients abscesses and in 6 hemangiomas. In 4 patients focal
nodular hyperplasia was established, in 2 inflammatory tumors and
in tuberculoma.
Multiple imaging modalities have been used in the diagnosis, including
ultrasound, dynamic computed tomography, angiography, scintigraphy,
and magnetic resonance imaging. Also Doppler (CDS) velocity
histogram analysis and percutaneous liver biopsy with CDS guidance
were performed.
Amongst 153 patients in 32 cases anatomical resection of the liver were
done. Six underwent left Iobectomy and 2 right Iobectomy. In 11 patients
extirpation of the cysts was carried out and 22 patients underwent
nonanatomical resection of the liver parenchyma with tumors. In 80
cases only laparotomy and biopsy was performed.
The postoperative complications included: hepato-renal insufficiency
5 cases, jaudance 4, stress ulcer -3, prolonged leak out of the fluid
6 patients, haemorrhage 2 cases. Overall mortality ratio was 3.7 %
On the basis of our experiences we state that radical resection of the
liver tumors are the best curative management.
IN SITU ISOLATED HYPERTHERMIC LIVER PERFUSION
WITH TUMOR NECROSIS FACTOR (TNF) IN PIGS
,S Nadalin, SR Shehata, L Moreno, A Thyen, P Flemming,
W Schtittler, M Martin, KJ Oldhafer, R Pichlmayr
Klinik f'tir Abdominal- und Transplantations-chirurgie, Medizinisch
Hochschule Hannover
Isolated hyperthermic limb perfusion with TNF is an effective
therapy ofirresectable soft tissue tumors. Only little data is available
about TNF in isolated hyperthermic liver perfusion (IH P). In this
study we analyzed the hepatic and systemic toxicity of IHLP.
Methods: 21 pigs (3 groups, 7 pigs each) underwent IHLP via the
hepatic artery and portal vein after total vascular isolation of the
liver. The livers were perfused with a blood-saline mixture at an
inflow temperature of 41.0"C for 45 minutes. 7 pigs (group A)
underwent oxygenated IHLP without TNF, while in group B and C
50l.tg TNF/kgbw were administered. Before reperfusion the livers
were washed with Ringer solution (group A and B) or with a
protein/dextrane solution (group C). The TNF concentration
(dttrfusate and systemic) was measured perioperatively. Laboratory
a were obtained within one week, liver biopsies were taken at the
d of the operation and at postop, day 7.
esults: In all pigs, a stable perfusion without major sytemic leakage
could be achieved. Mortality was 1/7 (portal vein thrombosis)in
group A, 5/7 (cardioresp. failure) in group B and 2/7 (hemorrhage,
cardioresp, failure) in group C. Initial median TNF concentration in
the perfusate were 2.31.tg/ml and decreased during perfusion to
1.SBg/ml. The systemic TNF concentration was below 0.1ng/ml
during perfusion but increased after reperfusion to 97.5ng/ml (group
A) and 12.5 ng/ml (group B). Transient liver toxicity (GOT, GPT)
was seen in all animals but normalized within one week. Bilirubin
and AP remained unchanged. Histology showed sinusoidal dilation
one hour and almost normal parenchyma one week after perfusion.
Conclusion: IHLP with high dose TNF is technically feasible
without significant systemic leakage. Hepatic outwash with
in/dextrane seems to be most important to avoid high systemic
concentration after reperfusion. IHLP causes only mild hepatic
toxicity which is almost reversible within one week. IHLP with high
dose TNF appears to be ready for clinical use in the treatment of
irresectable liver cancer.
97
P109 Pl10
APROTININ REDUCES BLOOD LOSS IN LIVER SURGERY
RESULTS OFA CONTROLLED STUDY
C..Lentschener, F. Mercier, C. Boyer-Neumann, D. Benhamou, C.
Smadja, C. Vons, D. Frartco.
Services d'Anesth6sie, de Chirurgie, et d'H6matologie, H6pital
Antoine B6clre, Universit6 Paris XI, France.
Although improvement in surgical management has allowed to
decrease blood loss during liver resection, liver surgery still remains
at risk of intraoperative bleeding and transfusion requirement. It has
been shown that administration of aprotinin a fibrinolytic inhibitor
and platelet protector decreased blood loss during open heart cardiac
surgery, orthotopic liver transplantation and hip or knee replacement.
The purpose of the present work was to determine if aprotinin could
reduce blood loss during liver resection. Ninety seven consecutive
patients undergoing liver resection for tumor were randomly
assigned at time of surgery to aprotinin (48 pts) or placebo (49 pts)
treatment. The aprotinin group received a loading dose of 2.106
kallikrein inactivator units (KIU) of aprotinin over a 20 minutes
period after induction of anesthesia followed by a c.ontinous infusion
of 5.105 KIU per hour until the end of surgery. The age, gender,
liver function, nature of the tumor, status of the liver parenchyma
and type of liver resection were similar in both groups.
Intraoperative blood loss was significantly (p<0.05) less in patients
receiving aprotinin (217 ml) than in those receiving placebo
(1655 ml). There were significantly less patients (p<0.02) requiring
blood transfusion in the aprotinin (17%) than in the placebo group
(39%). Mean blood transfusion (PRC) was significantly less
(p<0.01) in patients receiving aprotinin (0.6 U) than in placebo
(1.5 U). The same tendency was observed in patients with
malignancies with or without chronic liver disease and in those with
a benign tumor. No major adverse effect was observed in patients
receiving aprotinin. These results suggest that aprotinin reduces
blood loss and blood transfusion in patients undergoing liver
resection of a malignant or a benign tumor. Aprotinin is another
mean of decreasing blood loss during liver surgery.
ROLE OF LASER RAY IN TREATMENT OF LIVER
INSUFFICIENCY.
V.M.Lisienko
Department ofSmgical Disease, Urals Medical Academy,
Ekaterinburg, Rusda.
Mmifold positive effect on human organism, availability,
simplicity of laser using in any medicinal institutions give an
opportunity of the broad use of laser ray in complex
therapeutic and surgical treatment ofdisease ofliver and biliary
tracts. Ray of laser has especial significance in disease of
liver's instciency. Activation of fermental, oxidizing-
reductional reactions, and change of membrane's penetrability
are important. Excitation of bioenergetical, biostimulational
processes has analgetic, antioedema, anti-inflammatory effect.
It creates stimulating of regeneration of tissue, increasing of
protective stmasths of organism. Different kinds of low
intensive laser radiation are used for treatment after-effect of
the gall stone illness of 170 patients. The red helium-neon and
semi-conductor lasers for intravenous intracutaneous
endocholedohial irradiation have been applied. The
determination of individual sensibility of patient to laser
radiation was distinctive peculiarity of research. It was
permitted the nearest results to be 16.3 per cent more effec".Wve
and to speed up the date ofpatients recovery.
Plll Pl12
LIVER ASSOCIATED LYMPHOCYTES ISOLATED FROM
PERITUMORAL REGIONS OF COLON METASTASES ARE
HIGHLY RESPONSIVE TO MITOGENS
B.Lukomska, M.Winnock*, W.Gawron, Ch.Balabaud*, J.Polanski#,
W.L.Olszewski
Dep Surg Res Transpl, Med Res Ctr Inst, Pol Acad Sei, Warsaw,
Poland, * Groupe de Recherches pour L'Etude du Foie, Universite de
Bordeaux II, Bordeaux, France, # Surg Dep, Faculty of Medicine,
Medical School, Warsaw, Poland
Liver associated lymphocytes (LAL) have a well defined phenotypic
pattern, are low responders to mitogens, and suppress the PHA
responsiveness of peripheral blood lymphocytes (PBL). Their strategic
location in the sinusoid suggests that LAL may constitute a first line of
defence against gut derived bacterial, viral and food antigens. The
question arises whether LAL can also be involved in the recognition and
reaction to the metastatic cancer cells in the liver. Fifteen patients
undergoing partial hepatectomy forcolorectal adenocarcinoma metastases
were studied. In order to collect LAL population from normal liver
fragments (LAL-1) and peritumoral areas (LAL-2) the removed tissue
was perfused as described previously. The phenotype of isolated cells
were studied in FACScan, and responsiveness to PHA and ConA in
culture was measured. Results. There were no statistically significant
differences in phenotypes ofLAL-1 and LAL-2 population. Interestingly,
cultures of both populations with mitogens revealed evident differences.
The responsiveness to PHA (90 #g/ml) was 6304-1-634 cpm for LAL-1
and 18156:!:1147 cpm for LAL-2, (26547+2137 cpm for PBL), whereas
to ConA (5 #g/ml) the lymphoproliferative repense was 8177_+814 and
13556_+ 1310 cpm, respectively (13706_+ 1023 cpm for PBL), p<0.05.
Conclusions. Liver associated population isolated from liver tissue
adjacent to colonic adenocarcinoma metastases and remote normal areas
displayed similar phenotypes, whereas their responsiveness to mitogens
was different. Liver associated lymphocytes adjacent to tumor tissue
reacted stronger than those from normal distant areas. This indicates that
LAL could undergo subtreshhold stimulation by tumor antigens and this
effect was amplified by mitogens acting as additive factors.
98
OUTCOME RELATED FACTORS IN LIVER RESECTION FOR
HEPATOCELLULAR CARCINOMA IN CIRRHOTIC PATIENTS
L. Lupo, O.Pannarale, D. Altomare, L. Caputi, A. Volpi,
N. Palasciano, V. Memeo
Istituto di Clinica Chirurgica, Universit di Bari, Italy
Liver resection can offer a chance of cure for HCC. However, in
cirrhotic patients the outcome may be endagered by the onset of
postoperative liver failure leading to death.
The present study was aimed to identify factors correlated to the
unfavorable outcomein such patients.
Post-operative liver failure, defined a the presence of ascites,
jaundice andencephalopathy, major complications and deathwere
the end points. The following variables Ageand Sex, Child's score
(CH), serum Albumin (ALB), Bilirubin (BIL), Prothrombin
(PT), ASTandyGT, number and size of cancer nodules, extent of
resection (RES), Blood transfusion (BT) and Ischemic Time (IT)
were analysed by multiple in 33 cirrhotic: Child A: 20, B: 11, C:
2 who underwent liver resection of 42 lesions wedgeand non
anatomical resections 26, segmental 13, bisegmental 3) and
standard perioperative cares.
Results 16/33 48 % patients experienced different grades of
liver failure, 8 (24 %) developed major complications and 3 (9
%) died. Post-operative liver failure and complications were
related to BIL,PT, and BT: r2- 0.70, p<0.001 and 2--- 0.54,
p<O.O01 respectively, while Mortality was related to BIL, PT,
ALB andBT: 2-- 0.75, p<O.0001.
In the present series of cirrhotic patients, poor residual liver
function was the major factor determining liver failure,
complications and death following liver resection. BIL resulted,
by far, the most single relevant variable and BT the most
important surgery related.
Pl13 Pl14
COMBINED MINI-INVASIVE THERAPY FOR
COMPLICATIONS OF ADVANCED LIVER CIRRHOSIS
S.Lyalkin, K.Boulanov
Ukrainian State Medical University,
Institute of Clinical and Experimental Surgery, Kiev (Ukraine)
Mini-invasive approach seems to be most eligible in patients
with advanced liver cirrhosis. The aim of the study was to
evaluate results of elective combined therapy included splenic
artery occlusion (SAO) with subsequent endoscopic
sclerotherapy (ES) of esophageal varices, in 30 cirrhotic
patients (Child-Pugh class B- 18, C- 12). All of them had large
varices with previous bleeding, symptomatic hypersplenism
and ascites, 8 patients had clinical signs of encephalopathy.
SAO was performed in modified technique, that consisted of
simultaneous insertion of steel coil into the main trunk and 10-
15 synthetic emboli into the small branchesof the artery. ES
was carried out with intra- and paravariceal injections of 1%
sodium tetradecyl sulfate. ES began 14 days after SAO and
repeated every 6 month. Patient follow-up ranged from 12 to
36 months. Splenic infarction after SAO developed in 12
patients and resolved within 2 weeks. Complications of ES
were transient and of little consequence. There was no
mortality. During the follow-up no variceal rebleeding
occurred. Non-aggressive hemorrhage from gastric erosions
was observed in 3 patients. Four patients had episodes of mild
encephalopathy. Fifteen patients demonstrated complete
eradication of esophageal varices, accompanied by the
reduction in size of gastric varices. Correction of symptomatic
hypersplenism was universal. Twenty patients showed
complete absorption or considerable improvement of ascites.
Child-Pugh score significantly decreased in 15 patients.Thus,
subsequent application of SAO and ES proved to be effective
for the management of patients with complicated advanced
liver cirrhosis.
HEPATIC RESECTION FOR THE TREATMENT OF
WESTERN'S WORLD PRIMARY INTRAHEPATIC
LITHIASIS.
M.C.C. Machado, P. Herman, T. Bacchella, V. Pugliese, J.E.
Monteiro da Cunha, H.Wo Pinotti.
Department of Gastroenterology, Hospital das Clinic,as-
University of So Paulo Medical School; Brazil.
Thirty nine patients with intrahepatic lithiasis (IHL) were
treated between 1974 and 1993. There were 21 women and
18 men with a mean age of 38 years (range 11 to 75).
In 64.1% of the cases stones were bilateral in the liver
parenchima and in 23.1% were located only in the left lobe.
We adopted a systematic approach in the treatment of
these patients with an individualized surgery according to
the presentation of the disease. All patients were submitted
to surgical treatment, including biliary drainage procedures
and hepatic resections. Indications for hepatic resection
were the presence of liver atrophy or non-removable stones,
specialy in unilateral disease. Eleven patients were
submitted to liver resection, as an isolated procedure in 5
with unilateral disease and associated to a biliary drainage
procedure (hepatic-jejunal anastomosis) in 6 patients with
bilateral stones. There was no operative mortality.
In patients with unilateral disease where a resection was
good late results were found in all (100%) cases.
epatic resection associated to biliary drainage surgery for
bilateral disease leaded to 66.6% of good results.
In the treatment of IHL best late results occurred in patients
with unilobar disease, specially when a hepatic resection
was performed. In bilateral disease symptoms recurrence
occurred in 33.4% of the resected cases. Overall good
results in the treatment of 39 patients with IHL were
observed in 70.2% of the cases after a median follow up of
58 months.
Pl15 Pl16
TISSUE CHARACTERIZATION WITH MR IMAGING OF FOCAL
LIVER LESIONS: CAN THE BIOPSY BE ABANDONED?
R. Manfredi. A.M. De Gaetano, G. Maresca, GB Doglietto*, P. Marano, F.
Crucitti*.
Department of Radiology and Surgery*, Universit Cattolica del Sacro
Cuore, Policlinico "A. Gemelli", Rome, Italy.
PURPOSE: to evaluate the diagnostic accuracy of MR Imaging in
characterising focal liver lesions.
MATERIALS AND METHODS: We prospectively evaluated 51 patients
with focal liver lesions with different pulse sequences before and after the
e.v. administration of contrast media (Gd-DTPA). Morphologic data
(Signal intensity, margins, internal structure) and functional data were
recorded. All patients underwent percutaneous biopsy 1-7 days after the
MR examination; that was considered our gold standard. In case of
hemangioma, the gold standard was considered the 201Tl-labelled RBC
scintigraphy.
RESULTS: All hemangioma appeared hypointense on Tl-weighted images
and markedly hyperintense on T2-weighted images; all presented a
peripheral, globular enhancement during the arterial phase with centripetal
progression. Focal nodular hyperplasia (FNH) appeared isointense on TI-
and T2-weighted images in 71% of the cases; all lesions presented the
central scar. All FNH appeared hyperintense during the arterial phase ofthe
dynamic scan because ofthe hypertrophied arterial supply. On Tl-weighted
images hepatocellular carcinoma (HCC) presented a variegate appearance
(Hypo- Iso- and Hyper-intense). Conversely on T2-weighted images
appeared in 94% of the cases hyperintense. A pseudocapsule was observed
in 70% of the cases. In 74% of the HCCs presented hyperintensity during
the arterial phase. Metastases had a homogeneous appearance: hypointense
on T1-weighted images and hyperintense on T2-weighted images. MRI is
able to differentiate coagulative from colliquative necrosis.
CONCLUSION: MR Imaging is a powerful tool in characterising focal
liver lesions, reducing, in that manner, the number ofpercutaneous biopsies.
PREOPERATIVE DETECTION OF HEPATOCELLULAR CARCINOMA
NODULES: MR IMAGING WITH HEPATO-BILIARY CONTRAST MEDIA.
R. Manfredi G. Maresca, A.M. De Gaetano, G. Pirovano, P. Marano, F.
Crucitti*.
Department ofRadiology and Surgery*, Catholic University Rome, Italy
PURPOSE: To prospectively compare the diagnostic accuracy of Magnetic
Resonance Imaging (MRI) before and after the administration of a biliary
excreted contrast media (Gd-BOPTA) in the preoperative evaluation of
hepatocellular carcinoma (HCC) nodules.
MATERIALS AND METHODS: 14 consecutive patients with cirrhosis and, at
least, one HCC nodule, .underwent MR Imaging before and after the
administration of Gd-BOPTA, in order to determine the number and the site,
before surgical procedure. All patients underwent angiography with lipiodol
injection and lipiodol-CT weeks after. In patients undergoing surgical
procedure, an intraoperative ultrasound (IOUS) was performed. Lipiodol-CT and
IOUS were considered as gold standard of diagnosis. All patients had 6-months
follow up. 2 blinded readers reviewed the MR images for site, number, and
maximum diameter ofthe lesions. The results were recorded on evaluation sheets
and compared with gold standard tin'dings.
RESULTS: patients with a single nodule (diameter 1,5-7,2 cm) underwent
surgical procedure. The other 11 patients underwent chemioembolization because
8 had poor functiontal reserve and had satellite nodules. The sites of the
satellite nodules were: 6 in the VIII and IV segment, 4 in the II and VI segment,
2 in the III and VII segment, and in the sediment (diameter 0.2-13, mean 3.1).
Procedure Sensitivi
< cm 2 cm > 2 cm Total
(N=2) (N=14) (N=20) (N=36)
MR 0 10 (71%) 16 (80%) 26 (72%)
MR + (50 %) 14 19 (95%) 34 1,94%)
Gd- (100%)
BOPTA
The number and the site of the lesions were correct in all patients. The
conspicuity and the confidence in detecting/excluding lesions were superior in
post contrast images.
CONCLUSION: Gd-BOPTA-enhanced MR Imaging enables a better depiction of
satellite nodules and improving the selection of patient amenable of surgical
procedure.
99
PlI7 Pll8
PREOPERATIVE EVALUATION OF LIVER METASTASES: MR IMAGING
WITH HEPATOSPECIFIC CONTRAST MEDIA.
G. Maresca, R. Manfrcdi, A. De France, A. Spinazzi,S. Alfieri*, P. Marano, F.
Crucitti*.
Department of Radiology and Surgery*, Universit Cattolica del Sacro Cuore,
Policlinico "A. Gcmelli", Rome, Italy.
PURPOSE: To prospectively compare the diagnostic accuracy of MR imaging
with hepatobiliary excreted contrast media, Gd-BOPTA (Phase III clinical trial)
in detecting focal liver lesions.
MATERIALS AND METHODS: 20 consecutive patients with synchronous or
metachronous liver metastases were included in the study. All patients underwent
MR imaging before and after the e.v. administration of Gd-BOPTA. Gd-BOPTA
has a specific hepatocellular uptake. 2 radiologist independently evaluate the MR
images. The number, the site and the eventual coexistence of benign focal liver
lesions were recorded. All patients underwent surgical procedure with
intraoperative ultrasound (IOUS). The liver was scanned on its superior and
inferiorface, with high resolution probe.
RESULTS: the sensitivity of the lVIRI before and after the administration of
contrast media is reported in the table below:
Procedure
MRI
Gd-BOPTA-
enhanced MRI
< lcm
(1"=23)
13 (56%)
20 (86%)
Sensitivity,
2 cm > 2 cm Total
(N=28) N=33) (N=84)
23 (82%) 32 (96%) 68 (80%)
28 (100%) 33 (100%) 81 (96%)
There is an increase in the detection rate for small lesions (diameter < 2 cm) after
the administration of contrast media, specially for subcentimeter lesions. There
was a complete agreement on the site of the lesions between IOUS and MRI.
Benign lesions were characterized as such, even if they were very small (4-6
mm). The conspicuity of the lesions and the confidence in detecting/excluding
lesions were significantly superior after administration of contrast, because of the
higher contrast between the normal parenchyma that enhances and metastastic
lesions that no not.
CONCLUSION: Gd-BOPTA-enhanced MRI detects a higher number of
metastases and enables a more accurate selection of patients undergoing hepatic
resection.
DISTAL SPLENORENAL SHUNT. LONG TERM RESULTS.
CMarar/t J.L.Lzaro, J.Balsells, R.Charco,
E.Murlo, I.Bilbao, E.Hidalgo and J.Bonnin
Hospital General Universitario Vall d'Hebron,
Barcelona, Spain
MATERIAL A/D METHODS
From January 1987 to December 1995, 84 patients were
operated due to bleeding esophageal varices.
Forty-five patients underwent Warren operation with
a mean age of 55.8 +/- 13 years. Elective surgery was
performed in 32 cases (71.1%) and urgent in 13
(28.9%). Ethiology of portal hypertension was: liver
cirrhosis in 40 patients, liver fibrosis in i, portal
thrombosis in I, and rebleeding in patients with
previous portal hypertension surgery in 3. Child A
was in 32 patients (71.1%), B in 9 (20%) and C in
(8.9%).
RESULTS
Operative mortality was 6.7% (3 patients) and early
rebleedin.g occurred in four patients (8.9%) but shunt
occlusion was found in only one.
Follow-up: forty-two patients were discharged from
the hospital. Mean follow-up was 41.9 +/- 26.9 months
(range: 2.2 to 93.7). Ten patients (23.8%) died Jn
the follow-up (i from rebleeding, 3 from liver
failure, 2 from hepatoma, 1 from alcoholism, 1 from
post-transplant sipsis, 1 from respiratory infection,
and 1 from rectal adenocarcinoma).
Three patients presented encephalopathy requiring
medical treatment and three developed hepatoma. Three
underwent liver transplant.
Recurrent hemorrhage occurred in cases (9.5%) and
shunt occlusion was found in two by ultrasonography.
Several hemorrhage episodes were presented in other
patient despite open shunt and no evidence of
bleeding lesion was found by colonoscopy and upper
endoscopy.
Actuarial survival were: 83.5%, 80.8%, 77.7%, 70.1%,
and 60.8% at i, 2, 3, and 7 years, respectively.
CONCLUSION
Warren operation is a safe procedure with a low
rebleeding and encephalopathy rate, and low
mortality. We recommend this shunt as elective
operation in patients with bleeding esophageal
varices.
Pl19 P120
SURIGAL TREATMENT OF CYSTS HYDATID OF LIVER
I. Marinkovi6, M. Nikolic, R. krinkovic, V. Vuinic.
Department of Surgery, Medical centre of KralJevo,
Yuslavia In their retrspective study, the authors
the results of surgical treatment of liver's hydatid
cysts. The study covers 2 patients in the period
198o-1994. Regarding pathology, solitary and uncom-
plicated forms doeinated, as well as location at ri-
ght-liver's lobe. The dianosis was based on clini-
cal, laboratory, radiological, and seroloEical exa-
minations, as well as on echo-sonoraphg of abdomen,
and CT. Operative procedures consisted of cystecto-
my with Partial pericystectomy (32 pts), total peri-
cystectomy (2 pts), atypical and typical resection
of liver (6 pts>, marsupialisation (2 pts). Other
organs were surgically treated at 7 paents. Early
postoperative complications (bilious pertonitis, en-
ter bilious fistulas, liver's abscess, subphernic
abscess, dehiscence of operative wund, hemorrhage
of abdomen, etc). Were bored in o.9. Early postope-
rative mortality is 7.1. Bsed on our results, we
are for operative treatment in the form of cystecto-
my with partial percystectomy and omentum tamponade
of the cavity. We think this is most secure procedure
in majority of the cases.
PREVALENCE, CLASSIFICATION AND INCIDENCE OF
GASTRIC VARICES IN PORTAL HYPERTENSION OF
DIFERENT ETIOLOGIES.
_S.K. Mathur., L. Kakkar, S. Shah, M. Sanzgiri.
G.I. Surgical Services, Seth G.S. Medical College and K.E.M.
Hospital, Bombay, India.
A prospective study was done to document prevalence of gastdc
varices (GV) and Incidence of bleeding from vadous types of GV
in patients of portal hypertension (PHT) of different etiologies
and to propose a rational classification for GV. The study
includes 501 patients of PHT (cirrhosis 224, extrehepatic podal
venous obstruction [EHPVO] 195, non cirrhotic portal fibrosis
[NCPF] 80 and isolated splenic vein thrombosis 2 patients)
managed over a t0 year period; 349 males and 152 females with
age ranging from to 87 years. The GV were classified
(modified Hosking's classification as LCGV (Type 1), subcardlac
(Type 2a), diffuse fundal (Type 2b), diffuse fundal with splenic
vein thrombosis (Type 3a), diffuse fundal with generalised PHT
(Type 3b), LCGV with fundal varices (Type 4) and antral varices
(Type 5).The overall incidence of GV was 86%. (LCGV-65%,
Type 2-13%, Type 3-3%, Type 4-14% and Type 5-5%)
According to etiology, incidence of GV was 82% in cirrhosis,
95% in EHPVO and 75% in NCPF. However, the incidence of
bleed from GV was low, being 13%. (LCGV-13%, Fundal Type 2
& 4- 24%, Type 3- 100%) According to etiolgy, the incidence of
bleed was higher in patients of NCPF (22%) as compared to
cirrhotics and EHPVO (11%). in conclusion, though the
Incidence of gastdc vadces is high (86%), the incidence of bleed
is only 13%. Bleed is more common from fundal vadces (31%)
and in patients of NCPF.
100
P121 P122
LIVER MITOCHONDRIAL GLUTATHIONE METABOLISM
AND THE EFFECTS OF SOMATOSTATIN AND MANNITOL
ON GLUTATHIONE METABOLISM INAN EXPERIMENTAL
OBSTRUCTIVE JAUNDICEMODEL
A.Menteq, H.'evikel, Y. Yazer, A.(Yoker, F. Ytlmaz, M.Kth
Department ofHPB Surgery, Ege University, Izmir,Turkey.
Great efforts are made to determine the hepatic reserves and to
predict the prognosis and the severity of the negative effects caused
by obstructive jaundice besides treatment of obstruction. As we
know, in obstructive jaundice, respiratory functions in liver
mitochondria deteriorate, ketogenesis diminishes, bile duct
proliferation and hepatic fibrosis developes.
In this experimental tudy the hepatic metabolism of glutathione is
evaluated. Glutathione is found naturally in the body and in recent
years is popularised by its reducing effect on toxic metabolites and
preventing cell injury. Additionally the effects of mannitol, a
scavenger on free oxygen radicals, and somatostatin, a naturally
occuring cytoprotective hormon, on hepatic glutathione metabolism
are studied. After establishing experimental obstructive jaundice, 5
groups (Control-C n:7, sham operation-SO n:7, obstructive jaundice-
OJ n:8, obstructive jaundice treated with somatostatin-OJ+S n:7, and
obstructive jaundice treated with mannitol-OJ+M n:7) are formed to
measure the hepatic glutathione up-take labeled with Tc-99m. The
hepatic injury is also confirmed histopathologically.
Finally we observed that in obstructive jaundice liver glutathione
reserve is decreased and up-take is increased parallel to the severity
ofhistological injury. Mean liver glutathione up-take was 3.39 in C
group, 6.96-in SO group, 8.18 in OJ group, 11.74 in OJ+S group
and 9.22 in OJ+M group (p<0.05). It is also found that mannitol and
somatostatin did not improve glutathione reserves.
Clinically, glutathione scan can be helpful to evaluate the liver injury
caused by obstructive jaundice, to predict the prognosis of disease
and to assess the results of different kinds oftreatment modalities.
ORTHOTOPIC LIVER TRANSPLANTATION (OLT): AN ITALIAN
EXPERIENCE
IL Merenda, G.E. Gerunda, D. Neff, F. Barbazza, A. Bruttocao, G. Da
Giau, F. Zangrandi, R.M. Iemmolo, P. Angeli^, A. Bortoluzzi^, F.
Michielan*, P. Feltracco*, A. Maffei Faccioli
Istituto di Chirurgia Generale I, Istituto di Patologia Medica I, ^Istituto
di Clinica Medica II, *Istituto di Anestesia e Rianimazione, Padua
University
Between 1/7/1991 to 31/12/1995 we submitted 67 patients to 77
OLT. The indications to liver transplantation were: 41 chronic viral
cirrhosis (18 HCV, 11 H]3V all but 2 HBsAg+, and 12 mixed I-IBV and
HCV), 11 alcoholic chirrosis, 4 primary sclerosing cholangitis, 3 primary
biliary cirrhosis, secondary biliary cirrhosis, 2 HCC both in HBV and
HCV related cirrhosis, 3 fulminant hepatic failure (1 HBV related,
autoimmune and drug induced), Budd-Chiari syndrome and
cryptogenic cirrhosis. The patients mean age was 47 +/- 10 (range 22-
63). Accoprding to UNOS score at the time oftransplantation 9 patients
were status 1, 43 status 2, 12 status 3 and 3 status 4. Nine patients
needed a retransplantation because of PNF (5), chronic rejection (1),
fulminant hepatic failure (1), IVC thrombosis (1), arterial throbosis (1);
the last one required a third transplant because ofPNF. The incidence of
retransplantation was 12.9%. Seven (10,4%) early perioperative deaths
occurred (1 ventricular fibrillation, cerebral infarction, hepatic artery
mycotic aneurysm, fulminant hepatic failure and 3 MOF) and 5 late (1
intestinal volvolus, Pneumocistis Carini pneumonia, PTLD,
autoimmune hepatitis recurrence and HCV hepatitis recurrence with
tunsilla cancer). The patients and gratis year actuarial survivals are
respectively 83.5% and 72.6% with a mean follow-up of 486 and 421
days; chronic cholestatic liver diseases obtained the best results, with a
100% survival, compared to the patients opereted on for fulminant
hepatic failure with the worst, all dead at 1.5 year.
P123 P124
HEPATIC FIBROLAMELLAR CARCINOMA WITH
ENDOCRINE CHANGES
Morone G., Gramigna P., Abelli M., Cavallero M.,
Conti M., Ferro F., Maconi A.G., Picheo R., Zelaschi D., Fomi E.
University of Pavia (Italy), General Surgical Clinic, IRCCS San
Matteo Hospital, Hepato-biliary Surgical Unit.
A 18-year-old man was referred to our Institution with a 3-month
history of bilateral gynaecomastia. Also US findings of liver
haemangioma were reported. Phisical examination was normal,
as were haematologic and liver function test. Serum =-FP, HBV
and HCV markers were negative. CEA level was normal. CT
scan and MRI showed a 10-cm solid tumour in the right lobe of
the liver without evidence of extra-hepatic spreading. Des-7-
carboxy protrombin (DCP) level was elevated (162 ng/mg) and
by a needle biopsy a fibrolamellar carcinoma was diagnosed. A
right hemiepatectomy was performed and the postoperative
course was uneventful. The patient was discharged 12 days
after his laparotomy. He is currently alive without evidence of
recurrence 12 months after removal of the tumour. After surgery
DCP and oestradiol levels rapidly decreased and gynaecomasty
disappeared (DCP= 15 ng/ml; oestradiol= 69 pg/ml). Specimen's
histologic examination confirmed the preoperative diagnosis.
Intracellular (hepatocytes) oestrogens were found, but oestrogen
(ER) and androgen (AR) receptors were negative.
Conclusions 1) Surgical resection remains primary therapy for
fibrolamellar carcinoma with long-term survival. Liver
transplantation may be undertaken in patients with unresectable
tumour or hepatic tumoral recurrence. 2) Hormonal and
metabolic changes have been described in association with HCC
(erythrocytosis, hypoglicaemia, hypedipidaemia, hypercalcemia).
A fibrolamellar carcinoma producing oestrogens is rare and
intriguing observation. 3) According to HCC negative ERs and
ARs could be a better prognostic factor. 4) DCP positivity is an
interesting report and its investigation could allow an early
diagnosis.
101
LYMPHOBLASTOID IFN-ALPHA VERSUS RECOMBINANT IFN-
ALPHA.2a IN THE TREATMENT OF CHRONIC HCV HEPATITIS
E. Minola, R. Tambini, G.R Quinzan, A. Capelli, A.M. Cremaschi, M. Rizzi,
M.G. Finazzi, G. Gavazzeni, *A. $onzogni, *R. Ghislandi.
Department of Infectious Diseases and *Department of Pathology, Ospedali
Riuniti, Bergamo, Italy.
Background. Chronic hepatitis C is usually a mild progressive liver disease
d.ueto an infection with hepatitis C virus (HCV). Interferon-alpha is at present
the treatment of choice for chronic viral hepatitis. Available data show that
interferon (IFN) is more likely to be effective if administered for a prolonged
time (6-12 months).
Methods. To evaluate the efficacy of treatment with IFN, we retrospectively
studied 158 HCV-Ab positive, HIV-Ab negative patients (pts.) (89 men and 69
.women) aged between 16 and 63 (mean 43.1 years old) with chronic HCV
hepatitis (130 histologically proved, 82.3%) who received long-term therapy
with IFN-alpha (89 pts. with lymphoblastoid IFN-alpha and 69 pts. with
recombinant IFN-alpha.2a). The patients received IFN-alpha therapy for
year at the dosage of 3 million units (MU) thrice a week. In the lymphoblastoid
IFN-alpha group (54 men and 35 women; mean 43.2years old) 19 patients
had a history of intravenous drug use (IVDUs) with a drug free period > 12
months before therapy, 16 had been haemotransfused; 12 had histological
evidence of severe fibrosis and/o cirrhosis. In the recombinant IFN-alpha.2a
group (35 men and 34 women; mean 42.7 years old)9 patients had a history
of IVDUs, 22 had been haemotransfused; 8 had histological evidence of
severe fibrosis and/o cirrhosis. Treatment was completed in 101 pts. (63.9%;
in 53 pts. with lymphoblastoid IFN, 52.5%);.was stopped during therapy in 26
pts. (16.5%) because of absolute no response (18 with lymphoblastoid IFN,
69.2%) and in 16 pts. (10.1%) for intolerable adverse effects (11 with
lymphoblastoid IFN, 68.7%) and 15 pts. (9.5%) for other reasons.
Results:
Norm.ALTend therapy Norm.ALT at 12 mths FU Norm.ALTat24mths FU
1-1ymph.IFN.alpha 35/53(66.0%) 17/43(39.5%) 13/21 (61.9%)
2- IFN.alpha.2a 23/48 (47.9%) P=O.07 13/43 (30.2%) P=0.37 9/23 (39.1%) P=0.13
14 pts. included in the group and 11 pts. in the group 2, non responders
after 12 months of f-up, recieved a second course of IFN treatment with
higher doses and excluded at the 24 months of f-up evaluation.
Conclusions. In about half the patients with chronic hepatitis C treated with
IFN-alpha for a prolonged time, the serum ALT levels become normal, with
decreased activity in liver histology in 42.4% (14/33); a relapse is relatively
common after treatment has been stopped. There is no statistical difference
in biochemical response at the end of the therapy at 12 months and at 24
months of follow-up in patients who received lymphoblastoid IFN-alpha or
recombinant IFN-alpha.2a.
P125 P126
INTERFERON THERAPY IN LIVER CIRRHOSIS ASSOCIATED WITH
CHRONIC HEPATITIS .
E. Minola, ILTambini, G.P.Quinzan, G. Gavazzeni, A. Capelli, A.M. Cremaschi,
M. Rizzi, M.G. Finazzi, *A. Sonzogni, *R. Ghislandi.
Department of Infedious Diseases and *Department of Pathology, Ospedali
Riuniti, Bergamo, Italy.
Background: Chronic infection with Hepatitis C Virus (HCV) can lead to
cirrhosis and hepatocellular carcinoma. The prognosis of advanced cirrhosis
due to chronic hepatitis C is poor, and results of therapies, including liver
transplantation, have been unsatisfadory. Effediveness of interferon alfa (IFN-
alpha) in preventing hepatocellular carcinoma in HCV positive patients with
and without cirrhosis is unknown.
Methods: Thirty-five HCV Ab positive and HIV Ab negative patients (pts.),
with well compensated cirrhosis and chronic active hepatitis, treated with IF:N-
alpha in the period 1/1990-12/1993, were retrospedively evaluated. Thirty-
four pts. (97.1%) had an histologically proven diagnosis, whereas in one patient
liver cirrhosis was diagnosed based on unequivocal clinical signs and on
results of imaging procedures. These 35 pts. (16 men and 19 women, aged
between 26 and 62 [mean 51 years old]) were treated with recombinant or
lymphoblastoid IFN-alpha, 3 millions units (MU) thrice at week for year.
Twenty-four pts. (68.6%) completed the treatment, whereas IF:N-alpha was
discontinued in 4 pts. (11.4%) because of intolerable adverse effete, in 3 pts.
(8.6%) for thrombocytopenia, in 3 pts. (8.6%) because of no response and in
one patient (2.9%) because of marked exacerbation of liver cells necrosis.
Results:
ALT Norm. end therapy ALT Norm. 12 months FU
IFN-alpha 8/24 (33.3%) 3/24 (12.5%)
Treatment with IFN-alpha was not associated with an improvement of
histological features (periportal necrosis and portal and Iobular inflammation).
A liver biopsy was performed in 7 pts. after the treatment, and all patients did
not show any hystological changes.
Conclusions: Our findings showsthat interferon alfa may be useful in selected
patients with compensated cirrhosis and chronic adive hepatitis C, as 33,3 %
of patients normalized aminotransferase levels at the end of therapy and 3
patients are well and do not present any signs of readivation of liver disease
after one year of follow-up. We think that IF:N-alpha therapy should be
considered for seleded cirrhotic patients, also in order to prepare them for a
possible liver transplantation. IFN-alpha should be started at low doses and
the patients should be carefully monitored.
EVALUATION OF FUNCTIONAL CAPACITY
IN LIVER SURGERY
H.J. Mischinger, H. Bacher, H. Cerwenka, G. Werkgartner, F.
Quehenberger*, A. El Shabrawi
Department of Surgery, Department of Medical Statistics*
KarI-FranzensUniversityGraz, Austria
The high rate of postoperative mortality in extended liver
surgery is mainly;caused by liver dysfunction. In the recent study
we tried to evaluate liver function and liver biosynthetic capacity
for perioperative functional disorders.
Material and Methods: In a prospective study we analysed
41 patients 19 males 22 females with hepatic tumors. The
age ranged from 26 years up to 76 years, mean age was 59
years. Lver resection was performed under pringle manoeuvre
i.e. partial vascular exclusion ). Apartfrom quantitative liver
function test MEGX Mono-EthyI-Glycin-Xilidid labor
diagnostics, CT-Scan and Abdominal Ultrasonography were
performed preoperatively.
Results: Multivariate stepwise logistic regression analysis
revealed that parameters as CHE, Quick, PTT, bilirubin,
albumin, GGT, GPT, Child-Pugh score and MEGX were
significant predictors. In orderto determinethe relationship
between the preoperative liver chemist and the laboratory
data of the postoperative course, aprincpal components
analysiswascarned outonthecorrelation matrixofthe significant
variables. Preoperativelythere were no additional information for
the surgical risk using the MEGX Test in comparison with the
single parameters Bilirubin, albumin GGT GPT Quick and
P-rr).
The correlation matrix showed a decrease of single variables of
significant predictors postoperatively. In cirrhotic patients the
MEGX test showed an increase of significance.
Conclusion: The decrease of valence in single parameters
was based on operative and ischemic liver trauma
posttraumatic enzyme shedding and therefore the significance
of MEGX test increased postoperatively.
P127 P128
DIAGNOSIS OF ABSCESSES AT THE RESECTION MARGIN AP'TER
HEPATECTOMY
-EVALUATION OF COMPUTED TOMOGRAPHY APPEARANCE
OF PERIHEPATIC FLUID COLLECTION-
S.Miyzki,K.Takasaki,M.T ugita,M.Yamamoto ,T.Ohtsubo,
S.Katagiri,K.Akiyama
Department of Surgery, Institute of Gastroenterology,
'I'okyo Women's Medical College,Tokyo,Japan
Postoperative diagnosis based on computed tomography
(CT) was investigated for the early recognition of intra-
abdominal infectious disease following hepatectomy. This
study included 159 patients in whom CT was undertaken
during postoperative hospitalization,among 637 patients
who had undergone hepatectomy during the 7-year period
from 1987 to 1994. The CT findings of 75 patients with
infection and 84 patients without infection were
compared. Fluid collection at the resection margin was
observed in 66 and 63 of the patients with and without
infection, respectively. The significant CT findings
observed, in the patients with infection were (1) uneven
fluid collection indicated by a high attenuation value,(2)
gas in fluid collection, (3) irregularity of the fluid
collection margin, (4) marginal enhancement,and (5)
complications of pleural effusion. These findings Were
more significant in patients with hyperthermic symptoms
than in those without such symptoms. The investigation of
postoperative CT findings in post-hepatectomy patients
makes the diagnosis of intra-abdominal infection more
certain,and appears to be useful for applying treatment
with drainage.
INCIDENCE HBV INFECTION AND AUTOIMMUNE HEPATITIS BETWEEN
PATIENTS WITH CHRONIC HCV INFECTION
B. Mladenovi6, T. Tasi6, I. Stamenkovi6
Clinic for hepatology and gastroenterology, Ni, Yugoslavia
The study Was performed to establish the incidence of HBV infection or
autoimmune hepatitis in the patients with HCV positive, and chronic liver disease.
All patients were testing on HCV by a second generation ELISA tests, and were
tested on antinuclear and smooth muscle antibodies. We tested 39 patients,
18(46,15%) female. Theirs growth were from 1920-1975. All off them were with
chronic liver disease,, like hepatitis chronica activa were 17(43,59%), cirrhosis
13(33,33%), and HCC 9(23,08%). HBV and HCV positive were 15(38,46%), only
HCV positive were 23(58,97%), and 1(2,56%) was with autoimmune chronic
hepatiti,. We conclude that autoimmune chronic hepatitis is a rare condition, buth
possible, and that HBV infection combine with HCV were very often probably
because intravenous drug abuse and transfusion.
102
P129 P130
RUPTURE OF SPLENIC ARTERY ANEURYSM AFFER LIVER
TRANSPLANTATION
M Morganti, E Jovine, A Mazziotti, GL Grazi, E Scalzi, G Varotti, A Cavallari
2 Department of Surgery, University ofBologna, Italy
Aneurysm of the splenic artery (SAA) is a very rare disease in the general
population (0.7 %), but relatively frequent in patients with liver cirrhosis and
portal hypertension, with an incidence reaching 8-10%. In cirrhotic patients
this alteration is due to the hyperkinetic splancnic circulation. The spontaneous
rupture of the splenic artery aneurysm in cirrhotic patients with portal
hypertension is very rare and has only been described in patients who have
previously undergone liver transplantation (OLT).
The authors report their experience of two cases of rupture of splenic artery
aneurysm out of 230 liver transplants performed over the last 8 years. Both
patients, a male aged 38 transplanted for advanced stage cirrhosis HCV related
and a female aged 19 transplanted for an anti-LKM positive autoimmune liver
cirrhosis developed the rupture of the splenic artery aneurism within two
months after the transplant. Both were asymptomatic before the event and
underwent emergency splenectomy. At the time oftransplant, the splenic artery
did not appear to present any pathology in either of our cases. Neither of the
patients had undergone preoperative abdominal arteriography or MR/. Both
have survived and this is certainly not the rule, as shown by the data in the
literature. Attempts have been made to evaluate the risk ofrupture in relation to
the volume of the aneurysm and 15 mm is believed to be the threshold
diameter. Pain in the upper left quadrant of the abdomen may be a warning
symptom of rupture. Echo-Doppler alone is not sufficient to exclude an
aneurysm of the splenic artery. The high mortality reported after SAA rupture
suggests that arteriography or MRI should be carried out during the
preoperative work-up of the candidates for liver transplant. Once recognized,
especially if it is larger than 15 mm, the SAA must be treated by proximal and
distal ligation at the origin of the splenic artery and, when possible, the
aneurysm itself must be resected, since partial ligation alone may not be
sufficient for the formation of collaterals. When splenectomy is unavoidable,
some authors suggest delaying it until after the liver transplant.
ADVANTAGES OF AUTOLOGUS BLOOD TRANSFUSION FOR
HEPATECTOMY
,H.Taniguchi, A.Takadao M.Masuyama, H.Koyama, H.Tanaka,
M.Hoshima, K.Kitamura, A.Hagiwara, T.Yamaguchi, K.Sawai, and
T.Takahashi
First Department of Surgery, Kyoto Prefectural University of Medicine,
Kyoto, Japan
Seventy-one cases undergoing hepatectomy were investigated in regards to
advantages of autologous blood transfusion. Forty-four cases were
donated autologous blood before operation. Donated autologous, blood
volume significantly increased, 550g on an average in patients treated with
recombinant human erythropoietin (rh-epo). After donation, hematocrit
treated with rh-epo was as equal as without rh-epo. To investigate
postoperative changes in hematocrits and in liver function test, we classified
the subjects ipto 4 groups by intraoperative transfusion method, autologous
blood transfusion without rh-epo, autologous blood transfusion with rh-epo,
homolugous blood transfusion only, and no blood transfusion. Post-
operative hematocrits recovery was delayed in homologous transfusion
group, with hematocrit of 29.4% on 14 POD. In homologous transfusion
group,serum total bilirubin and transaminases were significantly higher
than in autologous and no blood transfusion group. In autologus with or
without rh-epo groups, liver function tests had similar courses in no blood
transfusion group, and the highest serum total bilirubin was 1.20 mg/dl on
POD. We consider that autologous blood transfusion was more beneficial
for hepatectomy than homolugous blood transfusion. And 800g of
preoperative autologous blood donation and less than 1500g intraoperative
blood loss will allow a safe hepatectomy without homologous blood
transfusion.
P131 P132
HEPATIC RESECTION FOR METASTATIC TUMORS FROM
COLORECTAL CANCER:ANALYSIS OF PROGNOSTIC
FACTORS AND PRESENT OF NEW PROGNOSTIC FACTORS
Ikuo Nagashima,Teruald Oka,Takuya Osada,Katsutoshi Naruse,
Tetsuichiro Muto
First Department of surgery,Universiy of Tokyo, Tokyo,Japan
Objective;
Determinants of prognosis after hepatic resection for metastatic tumors
from colorectal cancers were retrospectively studied in 64 patients.
Methods;
Clinicopathologic features of 64 patients were reviewed retrospectively
to determine which ones were strongly corelated to prognosis after
hepatic resection for metastatic tumors from colorectat cancers,using
univariate and multivariate analysis.
Results;
1,3,and 5 years survival rates after hepatic resection were
88.0%,56.2%,and 48.6% respectively. In a stepwise Co regression
analysis,infiltrative growth of hepatic metastases(hep-inf:p=0.0019),
cancer positive of hepatic resectional margin (rm;p=0.0022),lymphatic
invasion of primary colorectal tumors(ly;p=0.0048), adjuvant arterial
infusion chemotherapy (p=0.0200), and positive of other distant
metastases (p=0.0367) were significant prognostic factors for survival
after hepatic resection.
Conclutions;
Itis suggested,first that hepatic resection shuld be done without expose
of cancer at resectional margin,second that hepatic resection should be
attempted inpatients without infiltrative growth of hepatic
metastases,lymphatic invasion of primarytumors, or other distant
metastases, and third that adjubant arterial infusion chemotherapy might
be useful after hepatic resection.
103
THE FEASIBILITY OF PARTIAL HEPATIC GRAFT BY ARTIFICIAL
IVC: A PROPOSED MODEL FOR LIVING LIVER TRANSPLANT (LLT)
IN PIG
..13. Nardo, S. Cuccomarino, B. Santoni, P. Turi, A. Recordare, P. Caraceni*, R.
Bellusci, A. Mazziotti, A. Cavallari
Clinica Chirurgica II and Patologia Medica*, University ofBologna, Italy.
INTRODUCTION. Living related liver transplantation (LRLT) is the most
recent surgical innovation to supply at the availability of donor organs for
children. Today, the most common animal model of LLT utilizes the chimpan-
zee. Infact, it is very difficult to perform total hepatectomy in pigs while preser-
ving the intrahepatic IVC because of a large number of tiny short hepatic veins.
An anatomical study was performed to evaluate the feasibility of a LLT model in
pig. MATERIALS AND METHODS. Five large cadaveric white female pigs,
utilized for thoracic surgery, were used as liver donors and recipients. Donor
ration. The vascular and biliary elements to the left lobe were isolated. Two
small cannulae were inserted respectively in the left branch of the portal vein,
and in the left hepatic artery for the in-situ cold perfusion of the left graft. The
middle and left hepatic veins were clamped at the confluence into the vena cava,
and transected to permit the outflow of the perfusate. The left lobe was resected
along the demarcation line, due to the parenchimal cold perfusion. The liver graft
was removed. Finally, common venous orifice ofthe middle and left hepatic veins
was sutured before removing the clamp. Bench table surgery. An aortic graft with
iliac bifurcation was employed for the replacement of the IVC in the left graft.
The right iliac branch was cut 1.5 cm below the bifurcation and its orifice was
anastomised to the common duct of left and medial hepatic veins. Recipient
.The same animal was utilised as recipient, after removal of the right
lobe, as well as the intrahepatic IVC. The left graft was implanted ortho-topically
by suturing end-to-end the suprahepatic vena cava to the aortic prosthesis, the
portal vein, after clamping distally the prosthesis, .the iliac left branch of the
prosthesis to the inferior vena cava, and the hepatic artery. Finally, biliary
drainage was accomplished with a Roux-en-Y jejunal loop. RESULTS. The left
hepatectomy time ranged from 135 to 185 min (mean 150). The bench surgery
nean time was 25 min (range 20-33 min). The time of removal of the native
right lobe ranged from 8 to 14 min (mean 10 min). The mean anhepatic time was
93 min (82 to 104 min). The total recipient time was 183 min (range 161-216
min). The left lobe amounted to 31-42% (mean 35%) of the liver mass.
CONCLUSIONS. The present anatomical study demonstrates that it is possible
to use a vascular ostiaesis to re#ace the intrahepatic IVC in the recipient pig of
the left liver graft. This animal model could therefore be useful for the
experimental study ofLLT in pig.
P133 P134
INTRATUMORAL AND PERITUMORAL REGIONAL DIFFERENCES
IN DNA-PLOIDY OF RESECTED HEPATOCELLULAR CARCINOMA
B. Nardo, C. Melchiorri*, B. Stecca*, B. Santoni, A. D'Errico#, P. Chieco*, R.
Bellusci, A. Mazziotti, A. Cavallari. Clinica Chirurgica II, Universit di Bologna,
Istituto di Oncologia "F.Addarii", Bologna, # Istituto di Anatomia Patologica,
Universit di Bologna
INTRODUCTION: Human hepatocellular carcinoma (HCC) often reveals the
histopathological presence of a "tumor within a tumor", where the central part of
the nodule is composed of more malignant cells than the peripheral (Sugimura,
Science 258:603,1992). In this study we examined DNA ploidy and binuclearity
in the central and peripheral parts of the HCC nodule and in the surrounding
cirrhotic tissue.
MATERIALS AND METHODS: Fifteen cases of surgically resected solitary
HCC were selected. Central and peripheral parts of the neoplastic nodule and
surrounding cirrhotic tissue were available for 10 cases; in the remaining cases
the samples were taken from the central portion of the nodule and from the
surrounding cirrhosis. Control specimens were additionally ,obtained from normal
liver of 5 patients which underwent surgery for non-neoplastic pathology. Single
cell suspensions from formalin fixed specimens were Feulgen stained and the
cellular DNA content was quantified by image cytometry.
RESULTS: The main stem line was peridiploid all through the nodule in 4
HCCs, pefitetraploid in the central part and peridiploid at the periphery in 4
HCCs, peritetraploid in both parts in 2 HCCs. The main stem line in the samples
taken only from the central portion of the HCC was peridiploid in cases and
peritetraploid in 2. The fraction of mononucleate cells in the population of
polyploid (FMP) was generally higher in the central (78+/-17%) than in the
peripheral fragment (60+/-16%) of HCCs. Only two patients died of neoplasia
during the follow up (ranged from 80 to 1070 days); both presented a
peritetraploid main stemline of mononucleate cells in the central part of the
tumor. The main stem line of the surrounding cirrhotic tissue was always
peridiploid; however, in 4 cases, the FMP (44%, 45%, 56%, 66% respectively)
was much higher than in control liver (28+/-3%).
CONCLUSIONS: Our data indicate that peritetraploid stem line are more
frequent in the central portion of nodular HCC. Being a high ploidy of the main
stemline associated to a more aggressive clinical behaviour, the central part of
these HCCs might be in a more advanced stage of malignant progression then the
peripheral portion. In 4 cases the surrounding cirrhotic liver tissue presented an
abnormal amount of mononucleate polyploid cells; we will further evaluate if this
pattern may be associated to tumor recurrence after resection of a solitary HCC
nodule.
A 20-YEAR EXPERIENCE IN THE SURGICAL TREATMENT OF
RECURRENT HEPATIC HYDATID DISEASE
B. Nardo, M. Valeri, S. Cuccomarino, A. Recordare, E. De Raffele, B Santoni,
P. Turi, M.L. Lugaresi, R. Bellusci, A. Mazziotti, A. Principe, A. Cavallari
Istituto di Clinica Chirurgica II, University ofBologna, Italy
INTRODUCTION. Despite chemotherapy, surgery remains the treatment of
choice of hepatic hydatid disease (HHD). Although the literature on recurrence
rates of HI-ID is sparse, data on surgical treatment of recurrent disease seems to
be almost nonexistent. We report a 20-years experience in the surgical
management ofpost-operative recurrence ofHHD.
MATERIALS AND METHODS. From 1975 to june 1995, 23 patients (pts)
with recurrent HHD underwent surgery. Seven among 191 pts of our series,
treated for primary HHD, developed recurrence (3.6%). The remaining 16 pts
have had a surgical treatment in other Hospitals. The mean age of pts was 48 yrs
(range 22-74 yrs). A total of 44 cysts were found: 26 in the right lobe, 10 in the
left lobe, and 8 in both hepatic lobes. Diagnosis of post-operative recurrence of
HHD was based on US (91.3% of cases) and TC scan (100% of cases).
Angiography and endoscopic retrograde cholangiopancreatography (ERCP) were
used in the assessment of complex problems in 12 pts and pts, respectively.
Nineteen pts had undergone non-radical procedures (82.6%), and 4 pts radical
procedures (17.3%), for the primary HHD. The mean interval time from primary
to secondary HHD was 5.5 yrs. A total of 27 operations for recurrent HHD were
performed: 14 radical and 13 non-radical procedures. After surgery, albendazole
treatment in all pts was given. Eight non-radical and 4 radical procedures were
performed in the period 1975-85 vs 5 non-radical and 10 radical procedures in
the latest 10 yrs. In cases (21.7%) an US intraoperative was utilized.
RESULTS. There has been one death, due to sepsis after reintervention. Post-
operative complications, not requiring surgical reintervention, were seen in 6 pts
(26%): infections of the residual cavity, and external bile fistula. The
complications were higher after non-radical surgery (4 cases) than following
radical procedures (2 cases). Mean hospitalization period was also longer in pts
treated by non-radical surgery as compared to pt operated by radical procedures
(21.8 vs 17.7 days). No re-recurrence of hydatld cysts in the liver was observed
during a mean follow-up time of 3.5 yrs (range 6 months-5.8 yrs).
CONCLUSIONS. An higher recurrence rate appears after non-radical surgery of
primary HHD. Similary to primary HDD, the management of recurrent HHD is
radical surgery, whenever possible. In our experience, an improved operative
technique has allowed an increasing number of radical procedures in the last
decade.
P135 P136
EFFICACY AND MECHANISM OF PGE1 PROSTAGLANDIN E1
INTRAPORTAL INFUSION ON HEPATIC ISCHEMIA- REPERFUSION
INJURY
S. Natori, Y. Fujii, H. Kurosawa, A. Nakano, H. Shimada
Second Department of Surgery, Yokohama City University,
School of Medicine, YOKOHAMA, JAPAN
Isdemia-reperfusion induced liver injury is one of the major problems in surgical
liver dysfunction. Recently greater attention has been paied to the interaction of
neutrophils and endothelial cells through adhesion molecule. On the other hand,
PGE1 has been demonstrated to have protective effect on liver dysfunction.
However its precise mechanism has not been clarified. The AIM of our study is
to asses the efficacy of PGE1 intraportal infusion on hepatic ischemia-reperfusion
injury and clarify its mechanism. MATERIALS and METHODS: 70%
ischemic livers were established by clamping the left portal vein and hepatic artery
of the male SD rats for 60 min. After 3hrs of reperfusion samples were collected,
and measured the following items with or without intraportal infusion of PGE1
(1/z g/kg-min). 1) ALT,AST and the amount of bile 2) serum TNFx,IL-8 3)
the clearance of serum hyaluronic acid 4) histological analysis by HE staining
and immunohistochemical staining with ICAM-1 expression on endothelium 5)
the production of oxygen free radical of the liver under fluorescence microscope in
vivo RESULTS 1) PGE1 decreased the serum level of ALT and AST. 2)
PGE1 increased the bile production significantly. [0.97mg/g.liver-min(PGE1)vs
0.45mg/g-liver.min(CONTROL),p<0.05] 3) The serum level of IL-8 was
decreased in PGE1 group, although TNFa was not detected in both groups. 4)
The clearance of hyaluronic acid was kept higher in PGE1 group than in
CONTROL group. 5) Histological study revealed that the number of the cells of
focal necrosis of hepatocytes and infiltration of neutrophils were decreased in
PGE1 group. The expression of ICAM-1 on endothelium cells was also
suppressed by PGE1. 6) The production of oxygen free radical was decreased in
PGE1 group. CONCLUSION: These data indicate that PGE1 decreases
production of IL-8 and suppresses expression of ICAM-1. PGE1 intraportal
infusion may protect against the endothelial cell injury by reducing adhesion and
migration of activated neutrophils induced by ischemia-reperfusion of the rat liver.
PGE1 intraportal infusion could be an effective and practical strategy for hepatic
ischemia-reperfusion injury.
104
ANATOMICAL SEGMENTECTOMY FOR LIVER TUMOURS
TK.Neelamekam, K.Astbury, E.Carton, J.Mathias,
J.Geoghegan, O.Traynor. Liver Unit, St
Vincent,s Hospital, Dublin, Ireland.
The classic hepatic resections require removal
of a large volume of liver tissue. Resection of
individual liver segments allows preservation of
a greater mass of normal liver parenchyma
without reducing the curative potential of the
operation.
From 1989 to 1995 35 patients (20 female, 15
male; average age 56 years, range 30-70 years)
had liver segmentectomies. Fourteen patients
had resection of single hepatic segment and 21
patients had two or more segments resected
separately or in continuity. Indications for
operation included colorectal metastases (16),
gallbladder carcinoma (5), carcinoid (2),
hepatoma (1), benign lesions (11).
The limits of the anatomical segments were
defined by intraoperative ultrasonography and
the resections were performed by ultrasonic
dissection with portal inflow occlusion (mean
clamp time 34 mins). One patient died following
resection. One other patient died six weeks
later from cardiorespiratory failure. The only
major postoperative complication consisted of a
haematoma at the resection site which resolved
with conservative management. Eight patients
developed local recurrences and subsequently
died despite chemotherapy. The remaining 25
patients are alive and disease free with a mean
follow-up of 30 months.
Conclusions: Segmentectomy of the liver is a
safe procedure which minimizes loss of normal
parenchyma during hepatic resection. Segmental
resection is the logical procedure for isolated
or smaller hepatic lesions.
P137 P138
THE NEODJUVANT TRANSARTERIAL
CHEMOEMBOLIZATION IN HCC SUBMITTED TO LIVER
RESECTION. OUR EXPERIENCE
D. Neri G.E. Gerunda, A. Bruttocao, R. Merenda, F. Barbazza, F.
Zangrandi, F. Meduri, M. Bisello, A. Maffei Faccioli.
Department ofGeneral Surgery- University ofPadua- Padua- Italy
The transarterial chemoembolization (TACE) was introduced in mid '70
by Japanese authors as palliative therapy ofHCC. We tested this.method
also as neoadjuvant therapy in liver tumours followed by surgical
resection. We consider 28 patients affected by resectable HCC with a
mean age of 63.3 +10.3 years; they were divided in two group: 13
treated with a cycle of TACE before the surgery (TS) and 15 treated
only with surgery (S). All the patients were evaluated by US scan, CT
scan and clinically by Child-Pugh risk, Okuda stage, TNM stage,
Karnofsky index and AFP value at admission. The TACE was followed
by repetition ofCT scan for evaluation of gained LUF after fifteen days.
No patient was excluded to surgical resection after the CT scan control
for lesions previously not recognized. The therapeutic efficacy was
evaluated by percentage of necrosis resulted of 70% meanly. Both the
groups were evaluated by US scan, CT scan, clinically and with blood
samples after one, three, six months and later every six months. The
TACE was well tolerated with lesser complications as fever (38%), pain
in upper right abdominal quadrant (30.8%) and early nausea and
vomiting (85%). The relapses in TS group were less than S 0 vs 40%)
during the first year of follow up. The actuarial survival was 82.8% vs
86.6% in the first year, and 69% vs 65.1% at three years, respectively.
The overal mortality was 38.4% in TS and 60% in S. We concluded that
TACE as neoadjuvant therapy is well tolerated and "protect" from
tumor relapses favouring a better survival wthin two years fron surgical
treatment.
CLINICOPATHOIJ3GICALANALYSIS OF 42 INTRAHEPATIC
BILE DUCT CANCER (CHOI.ANGIOCARCINOMA) PATIENTS
WHO UNDERWENT LIVER RESF_L-q'ION. Y. Nozaki_', I. Ikai, Y.
Yamamoto, T. Kitai, Y. Sakai, N. Ozaki, M. Yamamoto, Y.
Yamaoka Y. lind Dept. of Surgery, Kyoto University Faculty of
Medicine, Kyoto, Japan, 606-01
In 10 years between 1985 and 1995, 42 cholangioeareinoma
patients underwent liver resection (resectability, 83.1%) in our
department. [1 in Stage I, 11 in Stage II, 5 in Stage III, 11 in Stage
IV-A and 14 in Stage IV-B (UICC)]. No patients of bile duct cancer
occurred at the porta hepatis were included. All had mild liver
dysfunction preoperatively; 6 with jaundice, with hepatolithiasis,
with liver cirrhosis. The total maximal diameters of tumors were;
more than 10 cm in 16, 5 to 10 cm in 15, 2 to 5 em in 9, and less
than 2 cm in 2. 27 patients had a single tumor and 15 had multiple
tumors. Vascular invasion was observed in 17 (to the PV trunk in 3,
to the first branch of PV in 9, to the main hepatic vein in 4 and to
IVC in 2). Biliary invasion was seen in 29 (to the extrahepatic bile
duct in 10). Liver resection was performed as follows;
trisegmentectomy in 11, bisegmentectomy in 27, segmentectomy in
2, less than segmentectomy in 2. Concomitant resection of the
extrahepatic bile duct was performed in 26, that of the h@atic artery
in 3, that of the PV in 8 and that of IVC in 2. Macroscopic
appearance of tumors were divided into 3 types: the intraductal
growth type in 2, the mass forming type in 37 and the pedductal
infiltration type in 3. Histologically lymphnode metastasis was
found in 14. As of Oct. 1995, 1-, 3- and 5-year cumulative survival
and recurrence-free survival rates of 42 patients were 63.6%, 21.2%
and 21.2% in the former and 54.7%, 18.3% and 14.6% in the latter,
respectively. Three year-survival rate of Stage II patients (52%) was
better than that of Stage III (20%), IV-A (26.4%) and IV-B (13.6%)
patients. Survival periods of patients with lymphnode metastasis,
either regional (n=5) or distant (n=9), were lower than those in
patients without metastasis. The analysis suggested that early
detection of the mass forming type tumors without bile duct invasion
or lymphnode metastasis is mandatory for longer survival of
patients.
P139 P140
PATTERNS OF RECOVERY AFTER LIVER RESECTION
Nuzzo G., Giovannini I., Boldrini G., Giu-
liante F., Chiarla C., Tebala G., Vellone M.
Geriatric Surgery Catholic Univ., Rome Italy
Data from 160 pts undergoing liver resection
for malignant or benign diseases were analy-
zed to characterize peculiar patterns of re-
covery after srgery, based on the postope-
rative monitoring of blood laboratory measu-
rements and other data. Three main postope-
rative trajectories were defined by a multi-
variable quantitative selection: I) uneven-
tful clinical course with early discharge;
2) complicated course (with hepatic insuffi-
ciency, sepsis or other complications) and
delayed discharge; 3) lethal outcome. Within
this framework, the possibility of identi-
fying each trajectory soon after surgery was
explored: among the relevant variables, the
fall in cholesterolemia (CHOL) in postop.
days I-3 correlated well in survivors with
the lenght of hospital stay (rZ= 0.30, p<
0.01). The fall in CHOL was larger in
trajectory 2 vs (p<O.01) and in trajectory
3 vs 2 (ns: too few cases in trajectory 3).
A delayed persistent increase in total bili-
rubin (BT) without cholestasis or main signs
of hepatic insufficiency was a peculiar pat-
tern in 3 pts: BT peaked after the 3rd week
and returned to normal after the 8th. with
the following evolution- BT= 2.6 + 2.0(POD)
O.052(POD) + O.O0034(POD) [r 0.74;
BT mg/ dl, 60% direct; POD Postop. Day].
These data may help to improve the characte-
rization of postoperative trajectories, with
the early prediction of complicated courses
and of the need for supportive therapy.
HEPATIC RESECTIONS IN NORMOTHERMIC ISCHEMIA
G. Nuzzo, F. Giuliante, M.Vellone, G.D.Tebala
Geriatric Surgery Unit, Catholic University, Rome, Italy
Temporary vascular inflow occlusion of the liver is an effective technique
to reduce the risk of bleeding and the number of blood transfusions during
hepatic resection. Aim of this study is to evaluate the influence of
normothermic hepatic ischemia (NHI) obtained by routine use of pedicle
clamping (PC) on postoperative outcome in 71 hepatic resections (2 re-
resections). Pts were 33 men and 36 women (mean age 56__.14 yrs; range
2-76). The most common indication for liver resection was malignant
tumors (80%). Liver cirrhosis was present in 12 pts and 19 pts had been
submitted to preoperative systemic chemotherapy. In cases with
contemporary resection of a portion of the digestive tract and with an
intestinal anastomosis, and in the case of deep jaundice, the use of PC was
limited for the risk of venous stasis on the intestinal anastomosis and for
the fear to worse the liver function already compromised. In 63 cases,
hepatic resection was performed with PC alone and in 8 cases hepatic
vascular exclusion (HVE) was used. Major resections were performed in
38 cases (53.5%). In 2 cases a tangential resection of the retrohepatic vena
cava under HVE was associated to right hepatectomy. The mean duration
of NHI was 42+_.20 min (7-107). Plasmatic values of ALT, AST, total and
conjugated bilirubin, prothr0mbin time, total cholesterol were evaluated
at I, III and VII p.o. days. Mortality was nil. Major complications
occurred in 12.7% of cases. Twenty-eight pts (39.4%) received
intraoperative (i.o.) blood transfusions: the mean of transfused blood units
was 2.5__.1.3 (1-6). Sixteen major resections (42.1%) did not require any
transfusion. Postoperative changes in liver function tests were moderate
and transient. This study confirms the benefit of PC in reducing i.o.
bleeding and postoperative complications. The routine use of PC during
hepatic resections is not associated with severe adverse effects on liver
function.
105
P141 P142
INTRAARTERIAL CHEMOTHERAPY 1N TREATMENT OF
COLORECTAL LIVERMETASTASES.
Pantoflicek J., Holeckova E.
Department of Surgery and Oncology,Hospital Sokolov,Czech rep.
From 1989 to 1994 a total of 217 patients with colorectal
carcinoma were admitted for surgery at our department.
We found in this groupofpatients during preoperative examination
and postoperative monitoring 78 cases ofliver metastases, 51 ofthem were
synchronous, 27 metachronous.
From this group of 78 patients 14/18%/underwent resectional
treatment, 35/45%/paliative treatment, 20/25%/patients were treated by
i.a.chemotherapy and 9/12%/patients by metamasectomy and
i.a.chemotherapy.
Hepatic artery acces was made by surgically implanted catheter
/lmplantofix Braun/through gastroduodenal artery.
In the group of 29 patients treated by i.a.chemotherapy monitoring
of,tumor response was made by tumor marker levels/CEA at 3m intervals/
and liver function tests. US imaging every 3m and abdominal CT scan
every 6 months was made.
2 different chemotherapy protocols were used during the years
1989-1994.
We observed- no change or progression in 11 cases/38%/
partial response in 6 cases/20%/
-objective response in 12 cases/42%/
Higher response rate 4/45%/we observed in the group ofpatients
treated by metastasectomy and i.a.chemotherapy compared with group
treated with simple i.a.chemotherapy 8/40%/.
We had no cases ofhepatic toxicity or duodenal ulceration, we
observed case ofport infection and 4 cases ofcatheter obstruction.
Median survival time was 11,2 months in the group of
i.a.chemotherapy patients.
Conclusion:Intmarterial chemotherapy has its place and benefit in
treatment ofliver metastases, we observed response to the treatment in 62%
ofour patients with minor toxicity.
ALVEOLAR LIVER HYDATID DISEASE: AUDIT OF SURGICAL
MANAGEMENT
J.A. Paraskevopoulos A.R.Dennison
University Department of Surgery, Royal Hallamshire
Hosp+/-tal, Sheffield, U.K.
Alveolar Liver Hydatid Disease (ALHD), a rare (5% of all
cases of liver hydatid disease) but often fatal disease,
can be efficiently cured only by complete surgical re-
section of the Echinococcus multilocularis(=alveolaris)
lesion.
We report our expeienee on 21 patients (10 male, 11 fe-
male) with a mean age 47.4 years (range 19 to 86) who
underwent surgery for symptomatic ALHD from 1972 to
1990. The main clinical manifestations were hepatomega-
ly in 15 (71.5%) patients, right upper quadrant pain in
4 (19%) and cholangitis in 2 (9.5%).The right liver lobe
was involved in 11 (52.5%) patients, the left in 2(9.5%)
and both lobes in 8 (38%) patients, respectively. Eight
patients (38%) underwent radical surgery in the form of
liver resection whilst in the remaining 13 (62%) pa-
tients various palliative procedures.were carried out:
liver resection (n=8), biliary-enteric bypass (n=2),
debridement and drainage (n=2), exploratory laparotomy
(n=1). The complication and recurrence rates were 28.5%
and 38%, respectively. There were 4 postoperative
deaths (19%) due to multiple organ failure (n=2) and
advanced local disease (n=2) leading to hepatic failure.
It is concluded that Echinococcus multilocularis is an
aggressive parasite resulting in extensive liver in-
vasion and requiring radical surgery which is associa-
ted with a high mortality rate.
P143 P144
THE POST CHEMOEMBOLIZATION SYNDROM IS NOT
RELATED TO TUMOUR NECROSIS.
F. Paye, M. Dahman, V. Vilgrain*, M. Zins*, Y. Menu*, P.
Jagot, J. Belghiti.
Department of Digestive Surgery & Radiology*, Hospital Beaujon,
University Paris VII, Clichy, France.
Neoadjuvant chenoembolization (CE) preceeding the resection of
hepatocellular carcinoma (HCC) prompted us to precise the
frequency and the causes of the post chemoembolization syndrom
(PCS), which associates pain, fever, raise of plasma transaminases
levels, and is generally considered as a result of turnout necrosis.
From 1989 to 1994, 29 patients with HCC including 8 with
cirrhosis and 15 with fibrosis had the whole procedure (CE and
resection) in our department and form the basis of this study. One
to 4 CE (Doxorubicin+Lipiodol+gelatin particles) have been
performed per patient. The PCS was defined by the occurrence,
during the first 3 days post CE, of a fever > 38.5C and/or a raise
of plasma transaminase levels (ASAT or ALAT) > 100 U/1 and > 2
fold the pre CE values.
After their first CE, PCS was present in 16 (55%) patients and
absent in 13. Among 8 patients who had several preoperative CE,
PCS recurred after a second CE in 3 (37%) but It never appeared
after iterative CE if it was absent after the first CE.
Histological examination of resection specimens revealed in
patients with PCS absence of tumour necrosis in 3 (19%), tumour
necrosis < 50% in 7 (44%), > 50% in 4 (25%), and a cotnplete
necrosis in 2 (12%), whereas in the group without PCS tumour
necrosis was always present including < 50% in 5 (38%), > 50%
in 5 (38%) and complete in 3 (23%). Liver cirrhosis involving the
non tumorous liver was present in 2 (12%) patients with PSC and
in 6 (46%) without PSC (p 0.04). Adhesions surrounding the
liver (perihepatitis) were present at surgery in 9 (56%) patients
with PCS versus in 3 (23%) without PCS (p 0.07).
In conclusion, the post chemoembolization syndrom was not
related to hepatocellular carcinoma necrosis but to the non-
tumorous liver status. It could be the result of a chemoembolization
toxicity on non-tumorous liver, which perihepatitis could be a
sequellae.
106
SHORT-TIME WARM ISCHEMIA INDUCES HEAT SHOCK PROTEIN
70 (HSP70) SYNTHESIS IN HUMAN LIVER.
R,Pfllicci*, F.Dondero*, A.Antonucci*, M.Barabino*,
M.Bertocchi*, C.Bottino*, G.Dardano*, F.Ermili*,
R.Mondello*, N.Morelli*, A.Pozzo*, U.Valente*,
L.Sampietro#, D.Storace#, M.Maiello#, D.Boeri#
*Transplantation Center of Genoa #Department of Internal
Medicine University of Genoa, Italy.
Primary failure is a poorly understood complication
possibly related to the ischemia-riperfusion injury of the
transplanted liver. Heath Shock Protein (HSP) system is one
of the most studied mechanisms of defense against cell
damage. The HSPs are a family of highly evolutionary
conserved proteins, constitutively evessed by the cell, but
rapidly induced by several types of acute stress causing
protein damage. HSPs contribute to the removal of altered
proteins and to the recovery of the protein machinery
synthesis. In a model of kidney transplant in pigs, heat
induction of HSPs correlates with enhanced survival upon
the further stress of ischemia-riperfusion. Aim of this work
is to evaluate whether a short period of warm ischemia is a
good inducer of HSP70 in human liver. In 10 subjects
undergoing partial hepatectomy for localized lesions, liver
biopsies were-obtained, from the unaffected portion of the
organ, before the clamp of the hepatic vessels and at the
end of the surgical procedure, after an average period of
recovery from ischemia of 76+39 rain. Total RNA was isolated
by the guanidine isothiocyanate method. The amount of
HSP70 and /-actin mRNAs was determined by the Norhtern
technique, with 32p-labeled cDNA probes. HSP70 mRNA
increases (150+__70%, 2p<0.05) after ischemia. The
enhancement of HSPT0 mRNA correlates with the length of
ischemia (r=0.658, 2p<O.O5). Our results show that a short-
time (30 min.) warm ischemia induces HSP70 synthesis in
human liver. The HSP induction preceding the harvesting
and processing of donor liver may enhance the organ
ability to recover from the ischemia-reperfusion injury,
possibly reducing the occurrence of primary failure.
P145 P146
LIVER TUMORS IN OUR DEPARTMENT
Piecuch J., Witkowski K., Orkisz W., Wylezol M., Slawski M.,
Pardela M.
II Department of General and Vascular Surgery Zabrze, Poland.
During last ten years (1985-19995) 129 patients with liver tumor
were treated in our department. 72 ofthem (56%) were qualified
for operation. This group consisted of 52 patients with primary
tumors and 20 patients with metastatic tumors. 29 of 52 primary
tumors were benign and 23 malignant. The benign tumors were:
hemangioma 13 (18,1%), cysts 10 (13,9%) and inflammatory
tumors 6 (8,3%). 72 partial liver resection were performed. In 6
cases metastatic tumor was synchronic with primarytumor (30/.o)
and in 14 cases metachronic (70%). The most common reason of
metastases was colorectal cancer (75%). The other reasons were:
gastric cancer (20%) and ovarian cancer (5%). The extension of
resection was determined according to Jorshy's classification.
There were no deaths in postoperative period in a group of29
patients with benign tumors. Several postoperative complications
occurred: biliary fistula (3,4%), subphrenic abscess 2 (6,8%),
pneumonia 5 (17,2%), wound infection 2 (6,8%). The mortality
rate in the group of43 patients with malignancy was 9% (4
patients). Postoperative complications rates were as follows:
biliary fistula 9%, acute liver insufficiency 7%, subphrenic
abscess 4,6%, pneumonia 14%, wound infection 7%. Besides liver
resection we performed liver transplantations in patients with
primary liver tumors. We consider the extended liver resection as
a difficult technically operation demanding perfect hemostasis
(possibly with the help ofargon coagulation or ultrasound knife)
and high quality intensive care during and after operation.
HEPATECTOMIES WITH TOTAL RESECTION OF
INFERIOR VENA CAVA
F. Pierangeli, A. Mazziotti, E. Jovine, M. Masetti, E.Scalzi,
G.Varotti, A.Cavallad
2 Departement of Surgery, University of Bologna, S.Orsola
Hospital, Bologna, Italy
Between January 1982 and December 1995 700 liver resections
were performed two of which with total resection of the inferior
vena cava (IVC) for tumor invasion. Patients and Methods: Two
patients affected by metastasis from colorectal cancer and
hepatocellular carcinoma respectively were submitted to
hepatectomies with total resection of the retrohepatic vent cava
under total vascular exclusion of the liver (TVE). No patient
required venovenous by pass. None had anticoagulant or
antiplatelet therapy after the operation. The median TVE time was
48 min. The first patient underwent a fight hepatectomy for a
colorectal metastasis: the retrohepatic vena cava involved by the
tumor was replaced with a 18 mm ring-enforced
polytetrafluoroethylene (PTFE) prosthesis. The second patient had
a fight hepatectomy with a total resection ofretrohepatic vent cava
for an hepatocellular carcinoma in non cirrhotic liver continuity was
established with a 16 mm PTFE prosthesis. Results: No patient
died in postoperative period. The first patient died 28 months after
the operation for tumor recurrence. The second patient is well and
free of desease 4 months after liver resection. Conclusions: tumor
involvement ofthe vena cava is not yet a controindication for liver
resection When is feasible direct suture is preferable. In case of
diffuse involvement of the retrohepatic vent cava a prosthesis is
required. Vent cava resection is easily performable with TVE
avoiding venovenous by pass.
P147 P148
RIGHT HEPATECTOMY IN ELDERLY PATIENTS
F.Pierangeli, S.Sommacale, L.Dugu6, A.Sauvanet, J.Belghiti.
Departement ofDigestive Surgery, H6pital Beaujon, University Paris VII
Clichy (France).
Less tolerance to liver resection in old patients has been suggested. The aim
of this retrospective study was to asses the recovery of old patients
submitted to right hepatectomy. Patients and methods: Forty non cirrhotic
patients with class or II of ASA were submitted for right hepatectomy (26
M; 15 F) from January 1990 to July 1994, Patients were classified
according to their age in two groups. In the first group were included
patients younger than 65 yrs (13 M, 11 F; average age: 46.1+/-12), while ha
the second were enrolled patients older than 70 yrs (12 M, 4 F; average age
72.7+3) Preoperative assessment of PT (86+/-13% vs 89+/-12%), factor V
(94+10 vs 102+13%), removed liver weight (1213+/-1348 vs 1080+/-1230 gr)
type ofvascular exclusion (2 TVE vs TVE) or Pringle maneuver and time
ofischemia (31+/-12 vs 36+/-8 rain) were comparable in both groups. Results:
blood transfusion rate (1.5+/-2 vs 2.3+/-2.4 units) and operative time
(318+/-110 vs 342+/-118 min) were comparable in the two groups.
Postoperatively (PO), ALAT rate on day PO (357+/-233 vs 358+/-218 UUL)
and day 7 PO (44+/-38 vs 58+/-25 UUL) were not significantly different.
Assessment of PT on day PO (50+/-10 vs 43+/-14%) and day 7 PO (71+/-17
vs 68+/-20) showed lower values in the age group >70 year, however the
difference was not statistically significant. On day PO, factor V (57+/-18
vs 43+/-20%) was significantly lower in the age group >70 years (13<0.05)
than in the < 65 years group, but there was not significative difference at
day 7 PO (74+/-26 vs 85+/-45%). A significant increase in bilirubine level was
noted on day PO (43+/-22 vs 92+75 mmoles/1) and day 7 PO (24+/-22 vs
47+/-50) in the >70 year group (p<0.01 and p<0.05 respectively). One
patient died in both groups (4% vs 6%). Postoperative course was
uncomplicated in 15/24 (62,5%) vs 11/16 (68.7%) patients. Hospital stay
was the same in both groups (16+/-6 vs 16,5+/-2 days). Conclusions: the
above described results indicate that age is not a major predictive factor of
recovery from right hepatectomy. However, our findings, indicate that older
patients show.a delayed postoperative improvement ofhepatic function.
A JOINT MANAGEMENT USING INTERVENTIONAL
RADIOLOGY FOLLOWED BY DERIVATIVE SURGERY IN THE
TREATMENT OF BUDD-CHIARI SYNDROME
A. Pisani Ceretti,* E. Opocher, R. Santambrogio, M. Castrucci, B.
Ongari,* M. Intra,* G.P. Spina*
2 Department of Surgery, Hospital Fatebenefratelli, Milan, Italy
6 Surgical Clinic, Hospital San Paolo, Milan, Italy
Department of Radiology, Hospital San Raffaele, Milan, Italy
Porto-systemic shunts are generally considered the most effective
therapy for primary Budd-Chiari Syndrome (BCS) in absence of
cirrhosis. However, when the BCS is complicated by the presence of
compression of inferior vena cava (IVC) with elevated pressure in its
subhepatic tract, an adequate decompression of the liver and portal
system is not possible. In these cases the complex surgical procedures
advocated in 1980's (meso-caval shunt or combined porto-caval and
cava-atrial shunts) present a high risk of failure. In 1990's
endoprotheses of various designs (Palmaz, Strecker, Wallstent and
Gianturco) have been used experimentally and clinically to
reestablish patency of occluded or stenotic tubular structures. In
particular, caval obstruction syndromes may be treated using
expandable metallic stents with a good efficacy. We present two
patients with a form of portal hypertension, characterized by
massive ascites and marked hepatomegaly, not responding to
aggressive medical treatment. In both cases the preoperative
investigations have revealed the presence of a BCS complicated by
compression of IVC with elevated pressure in its subhepatic tract.
Therefore, we decided, before performing a side-to-side porto-cava!
shunt, to clear the caval stenosis with several dilatations and the
placement of expandable metallic stents inserted by means of a
transfemoral venous approach. This stenting permitted to decrease
the subhepatic caval pressures respectively from 25 to 11 mmHg and
from 29 to 20 mmHg. After the subsequent surgical derivation, both
patients experienced an immediate relief of symptoms and a good
long-term result, respectively at 30 and 5 months of follow-up, We
conclude that the joint management using caval stents and porto-
systemic shunt can be useful in the treatment of the primary BCS
complicated by compression of IVC with elevated caval pressure.
107
P149 P150
CHANGES OF SYSTEMIC CIRCULATION SIX
MONTHS AFTER LIVER TRANSPLANTATION.
F.Piscaglia, G.Zironi, N.Venturoli, *GL.Grazi, *E.Jovine,
*M.Morganti, L.Masi, M.Valgimigli, C.Serra, *A.Mazziotti,
*A.Cavallari, L.Bolondi.
Istituto di Clinica Medica e Gastroenterologia, * Clinica Chirurgica
H, Universitt di Bologna, ITALY
Introduction" liver cirrhosis is characterized by a progressive
systemic hemodynamic derangement, the "hyperdynamic
circulation", characterized by increased heart rate and cardiac
output, with reduced mean arterial pressure. Aim: to assess the
effect of orthotopic liver transplantation (OLT) on systemic
hemodynamic changes of liver cirrhosis. Materials and methods: 20
patients submitted to transplant for end stage liver cirrhosis (m=14;
with ascites=12; 14 HCV+ and/or HBV, 3 alcohol, PSC, PBC,
Byler's disease), when first in the waiting list, underwent
impedance cardiography (by NCCOM-3, BoMed) in order to
measure cardiac index (CI), stroke volume index (SVI) and ejection
fraction (EF), mean arterial pressure (MAP) and heart rate (HR)
were also recorded. Cyclosporine and corticosteroids were
administered to all patients to prevent rejection. Hypertensive
patients after OLT were treated by Nifedipine. The same
measurements were repeated at six months in 17 patients (none
with ascites, two suffering from rejection, two retransplantated).
The 3 other patients had died (two in the first week after OLT and
one at fourth month for HBV reinfection). Student t-test for paired
data was used for statistical analysis. Results: at six months CI
dropped from 5.55 to 2.53 L/min/sqm (p<0.001)and SVI from
67.1 to 35.3 ml/sqm (p<0.001), MAP increased from 88.4 to
109.5 rnmHg (p<0.001) and HR decreased from 82.3 to 74.0 rpm
(<0.01). EF remained in the normal range but decreased from 61.4
to 52.9% (p<0.05). The three patients who suffered from primary
liver non function (two of which died) didn't show different
preOLT hemodynarnic parameters.
Conclusions: the typical features of systemic hyperdynamic
circulation are corrected six months after OLT.
A.]EEM[r QF LIVER ETION BY qUPt4HTATIVE RI/I.SIS
1A.PleskoviE, 2F.Danr, 1D.put and 1V.Pegan
Liversity of i.jubljana, School of HElicim, Ljubljana, SL0
2 Institute dosef Stefan, &mova 31, Ljubljana, SL0
Liver regeneration fter extensive hepatic resection is a serious
clinical probl. Presently there are no harmless and accuate
methods routinely used for the assessment of the progress of the
liver regeneration in patients. As NRI is a handless diagnostic
method for a,ssessment of soft tissues it seeed reasonable to
investigate the possibility of quantitative assessment of the liver
regeneration on animal mdels by use of NRI. Experiments were
perfoned on male rats weighing 150-200 g at the time of the major
surgical liver resection. 75% of th live was rmoved and NR images
were taken every day during the first w after the resection, and
late;" at 4 or 7 days intervals. The control group of animals was
sacrificed at th_ sane time intervals and the liver was weihed and
examined macro- and microscopically. Additionaly, the synthesis of
acetyl-cholinesterase by the regerating liver was measured on
liver hcmogenate by use of the constant pH-metric titration. The
results show that t[ liver regeneration reaches 60-75% during the
first 4 days, judged by the estimated size of the liver measured by
NRI. The results were in accordance with the increase of liver weight
measured in the control group of rats. Comparable although-smaller
progress in liver rege]eration was also revealed by use of the
methods, including the functional test of anabolic activity of the
regenerating liver by measurements of the cholinesterase activity in
a hmgeniz,ed liver specime]. NRI gave a further insight in the
process of the regeneration. The analysis of images revealed that the
reining part of the liver undergoes degeneration and simultaneously
the total liver mss is rapidly increased by the regenerating tissue.
We suggest that MRI is a reliable and hanless method for the
assessment of liver regeneration fter surgical resection.
P151 P152
MORBIDITY VARIATIONS AFTER LIVER RESECTION IN
CONSECUTIVE TEN-YEAR PERIODS 1975-84, 1985-95
G. Ramacciato, A.M. Balesh, W. Clazzer,
P. Aurello
I Department of Surgery, University of Rome
"La Sapienza," Rome, Italy
In order to determine complication types and
fluctuations in incidence, a retrospective
study was conducted on two groups of patients
undergoing curative hepatic resection for
neoplasm in consecutive ten-year periods:
1975-84 (group 1 52 patients) and 1985-95
(group 2 57 patients). Operative mortality
in groups 1 (6.1%) and 2 (3.2%) did not differ
significantly (p>0.05). The postoperative
complication rate decreased significantly in
group 2 (19%) vs. 1 (33%) (p<0.01). Abdominal
complications decreased significantly, from
25% to 10.5%, in group 2 (p=0.01);
postoperative hemorrhage occurred in two (4%)
group 1 patients, necessitating reoperation in
both cases with no mortality, vs. none in
group 2. While subphrenic abscesses decreased
significantly, from 9.1% to 1.9%, in group 2
(p=0.01), bile leak incidence in groups 1
(5.1%) and 2 (4.6%) showed no significant
difference (p=ns). Pulmonary complications
occurred in 15% group 1 and 7% group 2 cases
(p=0.01), and pleural effusion decreased from
8.9% in group 1 to 6.5% in group 2 (p=0.05).
Although morbidity after liver resection for
neoplasm has declined in the last ten years,
it still remains relatively high (27-47%) (i).
References
i. Sitzmann JV, Greene PS. Perioperative
predictors of morbidity following hepatic
resection for neoplasm. A multivariate
analysis of a single surgeon experience with
105 patients. Ann Surg 1994; 219: 13-7.
108
OCCURRENCE AND OUTCOME OF TUBERCULOSIS
AFTERLIVER TRANSPLANTATION
N. Rayes, W.O. Beehstein, O. Grauhan, R. Neuhaus, P. Lemmens, H. Keek, P. Neuhaus
Department ofSurgery, Virchow Clinic, Humboldt University, Berlin, Germany
Purpose: Patients with impaired T-cell function are at high risk for mycobacterial
infections. A particularproblem in liver transplant recipients is the hepatotoxicity
of the antimycobacterial drugs and their interaction with the metabolism of
immunosuppressants. In aretrospective studywe analysedtheincidence andoutcome
oftuberculo.sis after liver transplantation (LTX).
Material and methods: The charts of 570 patients who received a LTX between
9/88 and 12/94 were reviewed.
Results: The incidence oftuberculosis was 1,2% and the mortality 0%. All ofthe
infections were caused by Mycobacterium tuberculosis. There were two cases of
rejection and one drug induced hepatitis during treatment.
pat. pat. 2 pat. 3 pat. 4 pat. 5 pat. 6 pat.7
age in
55 22 58 61 52 41 57
years
previous Tb yes no no no yes yes yes
days after
OLT 189 350 375 920 21 60 120
immuno- CsA
CsA Fk 506 Fk 506 CsA Fk 506 CsA
suppression BT 563
rejection rejection
CMV
risk factors none none none none CMV CMV
involved
organs
lung
dissemi-
nated
lung kidney lung lung kidney
clinical menin- pneu- neph-
symptoms none
gitis monia ritis
MOFS fever fever
Conclusion: In order to reduce the incidence and mortality oftuberculosis a PPD-
test should be performedbefore OLT to define patients at high risk. Because ofthe
low mortality a routine INH-prophylaxis in PPD-positive patients does not seem to
be justified.
P153 P154
CMV-RETINITIS A RARE MANIFESTATION OF CMV-DISEASE
FOLLOWING LIVER TRANSPLANTATION
N. Rayes, R. Lohmann, C. Schmidt, H. Oetfle, W.O. Bechstein, R. Neuhaus,
P. Lemmens, H. Keck, G. Blumhardt, P. Neuhaus
Department ofSurgery, Virchow Clinic, Humboldt University, Berlin, Germany
Purpose: In immunosuppressed patients CMV-disease has a high morbidity and
mortality. Followingliver transplantation (LTX) clinical manifestations mostlyconsist
of pneumonia, hepatitis or unspecific symptoms like fever and arthralgia. In a
retrospective study we analysed the incidence and prognosis of CMV-retinitis.
Material and methods: Between 9/88 and 6/94 500 LTX were performed at our
centre. In the same period 83 CMV-infections occurred in 65 patients. Five ofthese
patients developed CMV-retinitis.
Results: patient patient 2 patient 3 patient 4
acute liver cryptogenic nutritivtoxic hepatitis C-
indication
for LTX
time after
LTX
symptoms
risk factors
therapy
clinical
course
failure cirrhosis cirrhosis cirrhosis
5 months 24 months 5 months 2 months
patient 5
HCC in
hepatitis C
3 months
impaired
pneumonia,
vision
impaired
impaired impaired
vision
none
vision
vision
overimmuno immun- pancytopenia renal failure tuberculosis
suppression deficiency rejection
cytotect/
foscavir
cytotect/ cytotect/ foscavir
cymevene cymevene cymevene
loss of
recovery vision
recovery recovery recovery
Conclusion: In contrast to liver transplant recipients CMV-retinitis is the most
common site ofinfection in AIDS patients and occurs in 15-20% ofthe patients. It
requires a continuous therapy with cymevene or foscavir.
Following liver transplantation CMV-retinitis is rare and can be treated sufficiently
with a short termtherapy. Because ofits location in peripheral areas ofthe retina and
the lack of additional symptoms CMV-retinitis is difficult to diagnose. Therefore a
fundoscopy should be performed in every patient with a presumed CMV-disease.
INFLUENCE OF POST-REPERFUSION BILIARY DAMAGE ON THE
DEVELOPMENT OF BILIARY STRICTURES (BS) AFTER LIVER
TRANSPLANTATION (OLT)
A. Recordare. B. Nardo, R. Bellnsci, A. Mazziotti, A. Principe, A. Cavallari.
Clinica Chirurgica II, University ofBologna, Italy.
BACKGROUND: Biliary strictures (BS) still represent an important cause of
morbidiB' after OLT. Other than surgical technique, other factors, like vascular
thrombosis, may predispose to BS. It was also suggested that prolonged cold
ischemia time (CIT) could be a risk factor for BS, and our previous report stated
that a significant post-reperfusion biliary damage occurs after a CIT > 10 hours.
Others reported a major incidence of BS after primary graft dysfunction (AST >
2000 in the first 72 hours after OLT). Aim of this stud)' is to verify if in our
experience the development of BS of unclear origin after OLT could be related to
a ischemia/reperfusion pathogenesis.
MATERIALS AND METIIODS: From Apr 1986 to Sep 1995, 220 liver
transplants were performed in 198 patients (pts) (123 males, 75 females, mean
age 42,48 _+ 12.06 years, range 7-60). Biliary reconstruction was choledocho-
choledochostomy on T-tube (C-C) in 208 cases, or choledocho-jejunostomy (C-J)
in 10 cases. In two pts biliary anastomosis was not performed because of
intraoperative death. Pts suriving less than month (n= 21) and pts with BS due
to arterial thrombosis (n 2) were not included in this stud),. In 22 pts the Euro-
Collins solution and in 153 pts the UW solution was used for organ preservation;
41 grafts in the UW group had CIT > 10 hours.
RESULTS: Eight pts developed a BS after OLT. All in the C-C group. All in the
UW group. One pt, that showed BS 13 months after OLT, had a Non-Hodgkin
limphoma involving the biliary tree. Another pt developed BS 40 months after
OLT becouse of alcoholic chronic pancreatitis. The remaining 6 pts developed BS
in the first month after surgeD'. One pt received a reduced-size and ABO-
incompatible graft, two known risk factors ofBS. Another pt had a stenosis at the
anastomotic level surgically corrected by re-doing C-C anastomosis. In the other
4 cases there were not evidences of gross technical defects; in 2 cases primary
graft dysfunction was observed in the immediate post-operative period; in the
other 2 cases CIT exceeded 10 hours. These cases were treated by percutaneous
balloon dilatation of multiple intra- and extrahepatic BS (n 1) or reconvertion
to C-J (n 3) with success.
DISCUSSION: BS were observed esclusively in the UW group, suggesting a
possible relation to the longer CIT permitted by the UW solution. Nevertheless,
only 5% of pts with CIT exceeding 10 hours developed a BS, supporting that the
damage could have a multifactorial etiology. BS were also seen after graft
dysfunction. A relative major sensitivi, of non-parenchimal cells (bile duct and
vascular endothelial cells) to reperfusion injury could be assumed.
P155 P156
RISK AND BENEFIT OF LIVER RESECTIONS FOR
MALIGNANCIES IN THE ELDERLY PATIENT
K.-P. Riesener R. Kasperk, P. Klever, V. Schumpelick
Dept. ofSurgery, University ofAachen, Aachen, Germany
Radical liver resections are the only chance of cure in patients
with hepatic malignancies. Nevertheless, liver resections still
bear the risk of complications and mortality, although the
procedure has become more safe in the recent years. The
study was. performed to rule out the risks ofliver resections in
patients of more than 70 years of age compared to younger
patients. From January 1986 to June 1995 we performed 330
livex resections in 288 patients with hepatic metastases (n
228) or primary liver malignoma (n 60). There wer 37
trisegrnentectomies, 46 hemihepatectomies, 34 left lateral
segmentectomies, 71 segmentectomies and 142
subsegmentectomies. 63 patients (71 resections) were older
than 70 years at the time ofoperation. The kind ofresection in
this group was comparable to the entire group. Overall 26
patients died subsequently to operation (hospital lethality
7,9%), 7 of them. were more than 70 years old. The
postoperative morbidity and long-term suryi.'val rates were
comparable in both groups. The mortality rate was influenced
by the kind of liver disease (primary vs. secondary tumour),
the extent of liver resection, and concomitant diseases rather
than by the patients' age. Liver resections for hepatic
malignancies are feasible even in the elderly patient and
provide considerably low mortality and morbidity rates. As a
conclusion the only chance of cure in malignant hepatic
disease should be offered to resectable patients regardless of
their age.
EXPRESSION OF TUMOR ASSOCIATED ANTIGENS BY
HEPATOCELLULAR CARCINOMA
V. Russo, *P. De Nardi, A. Ricci, *M. Braga, C. Traversad, *V. Di
Carlo.
Gene Therapy Program, DIBIT, * Chirurgia Generale IRCCS S.
Raffaele Universitt degli Studi di Milano
Many human tumours express antigens that are recognized by
cytolytic T lymphocytes (CTL). Recently several tumour specific
antigens, coded by MAGE, BAGE and GAGE genes, have been
identified in melanoma cells as well as non small cell lung
carcinoma, breast and ovarian tumours. These family antigens are
potential targets for an immunotherapeutical approach.
Liver tumours are a major cause of cancer death in cirrhotic
patients. Chemotherapy has failed to demonstrate a significant
therapeutical efficacy therefore a specific immunotherapy should
be an interesting goal. In order to examine the presence and
distribution of tumour associated antigens in hepatocellular
carcinoma (HCC) we used PCR analysis and ethidium bromide
staining to test the expression of the MAGE, BAGE and GAGE
family.genes in 8 patients with HCC.
According to TNM Classification there were one T1, four T2 and
three T3 tumour; three tumour were well differentiated (G1), two
moderately differentiated (G2) and three undifferentiated (G3).
Fifty percent of the lesions expressed at least one of the antigens;
MAGE-1, MAGE-2 and MAGE-3 were expressed in 37.5% of the
tumours; BAGE in 25% and GAGE in 12.5%. Antigen expression
seems related to tumour grading, in fact 2 out of 3 of the less
differentiated tumour expreessed 4/5 antigens while 2 out of 3 well
differentiated showed no antigen expression. However, due to the
small sample, no significant correlation, was observed between
antigen expression and tumour grading or staging.
Our results suggest that MAGE, BAGE and GAGE tumour
antigens could be targets for specific immunotherapy in patients
with HCC.
109
P157 P158
PLEURAL EFFUSION AFTER HEPATECTOMY: CAUSES AND
CONSEQUENCES
A, Sa Cunha, S. Thomas, L. Berthoux, A. Sauvanet, J. Belghiti.
Department of Digestive Surgery, Hospital Beaujon, University Paris VII,
Clichy, France.
The incidence of and risk factors for pleural effusion after liver resection are ill-
defined. To address this issue, prospective study was performed between
january and july 1995 in every patient undergoing elective hepatic resection by
an abdominal incision.
Patients and Methods 52 consecutive patients underwent hepatic resection of
whom, 6 were excluded because they had undergone a simultaneous
diaphragmatic resection. Ages of the 46 patients included for analysis ranged
between 21 and 77 years (51+14 years). Indication for resection was a primary
hepatocellular carcinomas in 22 patients (of whom 17 had an associated chronic
liver disease) a benign liver tumour in 15 patients, liver metastasis in
patients and a cholangiocarcinoma in 4 patients. The abdominal incision was
an upper midline incision (n=7), a right subcosml incision (n=7), a right and
left subcostal incision (n=20)or staped incision (n=12). Major hepatic
resection (resection of > 3 segments) was performed in 21 (46%) and 31 (67%)
underwent hepatic resection with a complete dissection of the fight coronary
ligament. All patients had pre and postoperative pulmonary function tests.
Pleural effusion was diagnosed by means of chest X ray examination who was
realised daily from day 2 onwards. Postoperative ascites developed in 13
patients.
Results 31 patients (67%) developed a pleural effusion requiring aspiration in
five and chest tube drainage in 4. The parameters significantly associated
(p<0.05) with pleural effusion were ascites (n=13 (1.00%), total dissection of
the fight coronary ligament (n=25 (80%) and chronic hepatopathy (n=13 (76%).
Postoperative function tests (EVMS) was significantly decreased in patients
with pleural effusion (58% vs 71%)
In onlu.sion these results indicate that after liver resection by an abdominal
incision, 2/3 of the patients developed a pleural effusion. This complication
required a specific treatment in 1/3 of the patients. The parameters associated
with pleural effusion were a postoperative ascites, the presence of chronic
hepatopathy and the total dissection of the right coronary ligament.
LAPAROSCOPIC TREATMENT OF LIVER HYDATID CYSTS
A. Sa_ala,m
Department of Surgery, Erciyes University School of Medicine,
Kayseri, Turkey
Laparoscopic use of a novel Perforator-Grinder-Aspirator (PGA)
apparatus which is specifically designed for the evacuation of' hydatid
cysts has been reported. Fifteen hepatic hydatid cysts in nine
patients were treated by laparoscopic technique using this tool which
mainly penetrates the cyst by opening a hole on the cyst wall, grinds
the particulate and sucks it all out; while the classical surgical
aspirators are almost always blocked by the daughter cysts and
laminated membranes.
If the cyst is small, the management of the cavity is achieved by
simple drainage, otherwise vacuum obliteration with the application of
-250 mbar negative pressure may be necessary. High vacuum
obliterates the cystic cavity by clinging to the opposing cyst walls, and
collapses the bile ducts which opens into the cavity.
In the postoperative period, none of the patients had bile drainage.
In the following 4-32 months period, Computerized Tomography (CT)
and ultrasound examinations revealed progressive decrease in the
size of six cysts and disappearance of the lesions in nine cysts.
It is therefore concluded that laparoscopic treatment of hepatic
hydatid cyst could be easily and effectively done by the use of this
novel instrument. Obliteration of the cyst cavity by high vacuum is a
time saving procedure, easy to perform and reduces or totally
eliminates bile drainage.
P159 P160
UNRESECTABLE COLORECTAL LIVER
METASTASES: RESULTS OF HEPATIC ARTERY
CHEMOTHERAPY USING A TOTALLY IMPLANTED
PUMP
G. Samori, C.Beati, A.Ferrandi, R.Dallatana, L.Gastaldo,
G.Mortara, G.Pancera*
Departments of Surgery and Ontology* S.Carlo Hospital,
Milan, Italy.
Intra-arterial chemotherapy in the treatment of colorectal liver
metastases without extrahepatic desease seems to increase the
response rates compared with systemic chemotherapy. From
January 1990 to September 1995, 54 patients (39 male, 15
female) with a mean age of 62 years (range 36-77 yrs) were
treated with floxuridine 0.25 mg kg over 14 days via a totally
implanted infusion pump (Synchromed Medtronic) with the
addition of systemic folinie acid (100 mg m-2 day- over 4 days)
and 5-fluoruraeil (5-FU) 400 mg m-2 day-1 over 4 days. There
was no operative death. Post-operative complications were: 3
haematomas, 4 wound infections, 2 catheter tromboses, 2 pump
failures, 5 catheter dislocations, 4 pseudo-aneurysms. In the
follow up 49 patients were evaluated. The median extent ofliver
envolvement was 30.6+9.9%. A complete response was achieved
in 14 patients while a partial response in 18 patients and no
change in 12 patients. The overall response rate was 65%;
48.9% ofpatients are alive at 2 years, with a median survival of
20 months. 22 patients developed chemically reversible hepatitis
and 15 diarrhoea; there was neither biliary sclerosis nor gastric
ulcer. Systemic and intra-arterial ehemotherapie infusion via an
implanted pump seem to reduce hepatic progressive disease with
an acceptable toxicity and postoperative complications. A
multieentrie randomized trial to evaluate the efficacy of intra-
arterial + systemic chemotherapy in the treatment of colorectal
liver metastases has been instituted.
HEPATIC TRAUMA. A 16 YEARS STUDY
L. Sanz, J.L. Grafia, J.J. Gonzdlez, G. Bermejo, A. Miyar L., A. Blanco,
E. Martfnez
Department of Surgery B. Hospital Central. University of Oviedo. SPAIN
Trauma is the principal cause of death in young people, and liver injuries
the most frequent abdominal lesions that lead to death. We review our
experience, with emphasis on clinical and therapeutic aspects. From
January 1979 to December 1994, 111 patients with liver trauma were
operated on in our unit. There were 91 men (81.9%) and 20 women
(18.1%), with median age 31 years (range 5-84). Etiological factors,
clinical aspects, method of management, complications and mortality
were assessed. 80 (72.1%) sustained blunt injuries (60 (54.1%) motor
vehicle lesions) and 31 (27.9%) penetrating, trauma (24 (21.6%) stab
wounds). There were 60 patients (54.1%) who arrived at the
emergency room with shock, 20 (18%) with open abdomen and 8
(7.2%) with coma. 74 (66.6%) had associated lesions. Abdominal
injuries included the spleen 28 (25.2%) the mesenteric vessels
12(10.8%), the pancreas, duodenum and vena cava 3, the
porta/mesenteric vein 2 and the hepatic veins as the most notable
lesions. According to Calne 1, the majority of lesions were grade II, 46
(41.4%) and III, 38 (34.2%). Surgical therapy included hepatorrhaphy
80 (72.1%), perihepatic drainage 16 (14.4%) and resectional
debridement 15 (13.5%). Secondary hepatic procedures were required
in 16 patients including perihepatic packing in 13 and selective hepatic
artery ligation in 4.22 patients (19.8%) died, 10 in the operating room
(9 due to exsanguination) and 12 in the postoperative period. From 89
patients who survived, 56 (62.9%) had complications: systemic 45
(50.6%) particularly pulmonary complications, and abdominal 39
(43.8%), intraabdominal abscess 19, wound infection 15.24 (21.6%)
required reoperation and 6 (5.4%) radiological drainage of hepatic
perihepatic collections. Hepatic trauma is, in our experience, a problem
most frequent among young adults, and is mainly related to motor
vehicle accidents. Mortality in these cases is moderate and morbidity
high. Haemorrhage control is the principal determining factor in reducing
mortality.
1. Calne et al. Br.J.Surg. 1982;69:365-8.
110
P161 P162
LIVER REECTION FOR METASTASES OF NON-COLORECTAL ORIGIN
M Schwa, S Emre, S Guy, P Sheiner, C Miller
The Mount Sinai Hospital, New York, New York, USA
Resection ofliver metastases ofeolorectal origin is ofproven benefit in
properly selectedeases; resection ofliver metastases ofnon-eoloreetal origin, on
the other hand, is less commonly performed mad less widely accepted. We report on
a series of29 such cases. Methods: The records ofall patients who underwent
resection ofnon-colorectal liver metastases between 3/89 and 11/95 were
reviewed. Primary site, length oftime from resection ofthe primary tumor,
indication for resection ofliver metastases, type ofresection, adjuvant therapies,
and patient survival were recorded. Results: Of221 liver resections performed at
our institution between 3/89 and 11/95, 29 were for tumors ofnon-colorectal
origin. Tumor types included: ovarian-9; leiomyosarcoma-3; lung (adeno)-2;
hemangiopericytoma-2; melanoma-2; adrenal-1; ampulla of Vater-2; anus
(squarnous)-1; breast (adeno)-1; breast (angiosarcoma)-1; salivary gland-1;
hypernephroma-1; thyroid (follicular)-1; malignant fibrous histiocytoma-1;
pancreas (neuroendocrine)-1. Indications for resection included: palliation of
symptorr due to large tumor mass-8; palliation ofsymptoms due to tumor
hormone cretion-2; asymptomatic liver-only tumors resected to alter prognosis-
13; debulking, along with resection ofdiaphragmatic/peritoneal metastases-6 (all
ovarian). Thirteen patients underwent anatomic lobectomy, and 16 underwent
nonanatomic resection. Mean time between resection ofthe primary and liver
resection ws27 months. Nineteen patients received adjuvant chemotherapy post-
liver resection. Atmean follow-up of26 months, 20/29 patients are alive; 13 of
these are clinically tumor-free, and 7 have or have had recurrent tumor. There was
one perioperative mortality, and there have been 8 tumor-related deaths.
Conclusion: These patients can be broadly classed into three groups: A) Patients
with ovarian Ca, in whom debulking is ofestablished value, B) Patients with
metastases apparently confined to the liver from a wide variety ofnon-colorectal
primary sites, and C) Patients with large, symptomatic liver metastases ofa variety
ofslow-growing types with or without less significant disease elsewhere. As part of
mulitdisciplinary approach, liver resection in these three groups ofpatients has a
role both in palliation ofsymptoms and in extending survival.
RADIOFREQUENCY ABLATION OF LIVER TUMOUR
C. Scudamore, A. Buczkowski, A. Poostizadeh, D. Owen, K. Qayumi
Tumoura of the liver remain major worldwide managementproblem. Primary tumoura
often in patients with very poor liver function making surgery imposeible complicated.
Metastatic lesions may betoo extensive for complete surgical removal. Multiple modalities for tumour
ablationhave beenstudied, including alcoholinjection, cryosurgeryand turnoutembolization- all have
disadvantages. Radiofrequency ablation oftumours has.recently shown promise. Our experience in
humans comprises 12 patients who have had their primary metastatic tumour ablated with
radiofrequency energy applied by insertion with 14 gauge probe followed by hepatic resection. This
probe generates spherical of tumour destruction up to in diameter with temperature
between 80-90* for minutes. All tumours stained with NADH stains demonstrating tumour
membrane destruction without protein coagulation. We have recently embarked upon ablation of
subtotally reseeted tumours (careinoid and colorectal metastases) using radiofrequeneyablation
adjunct to surgical resection. Our operative experience with this radiofrequeny probe has
demonstrated its usefulness potential treatment for small liver tumoura.
BT RRM 2.2 bi-polar hepatic vein
RS CRM 3.5 bi-polar portal hepatic
DH CRM 5.5 bi-polar nil
SM PM 2.7 mono-polar hepatic vein
VL HCC 5.0 mono-polar nil
FS HCC 1.0 mono-polar nil
NK CRM 2.7 mono-polar poflal hepatic
CC Cyst 4.0 mono-polar nil
RB CAR 1.5 mono-polar nil
CL CRM 3.8 mono-polar hepatic vein
TH HEM 1.5 mono-polar hepatic vein
ML HCC 3.0 mono-polar nil
bubble in vein
bubble in vein
CRM eolorectal metastases; PM pancreatic metastases; HCC hepatocellularmetastases; HEM
hemangioma; CAR Caroinoid
This preliminary study demonstrates that radiofmequency ablation shows promise for the
nonoperative management of liver tumours.
P163 P164
COMPARISON OF DIFFERENT APPARATUSES FOR FINAL
HEMOSTASIS ON THE RAW LIVER SURFACE (ARGON BEAM
COAGULATOR VS. PLASMA FLOWS AND ND:YAG LASER) IN
COMBINATION WITH TACHOCOMB AND TISSUCOL
Severtsev A., Bashilov V., Brekhov E., Tchudaev D.,
Uljanov V. Medical Centre for Russian Government
(MCRG); Moscow, Russia
Introduction: There a lot of different appa-
ratuses to control oozing from the raw liver surfa-
after liver resection. Argon beam coagulator,
plasma flows and laser irradiation (most frequently
Nd:YAG laser; usually used for these purposes,
Objective: To evaluate the local influence of dif-
ferent physical methods the liver tissue and
the whole body.
Patients and methods: We used argon beam coagulator
(Pfizer, USA), plasma flows (Fakel I, Russia) and
Nd:YAG laser irradiation (Dornier, FRG) to control
oozing from liver raw surface after resection du-
ring 21 operations. The final hemostasis was achi-
ved by Tissucol or TachoComb. The general influence
of diff.phys, methods was calculated by means of
APACHE system and clinical course. The local
damage was evaluated by the hystological exami-
nation of intraoperative biopsy. Every time, for
each clinical situation certain apparatus
selected.
Results: No significant differences were found in
APACHE system for different apparatuses. There were
no differences in hystology for argon beam coagu-
lator and plasma flows. The damage of liver tissue
froth laser irradiation was the deepist. The
post-operative course was much more favourable in
laser group. There were any differences between
groups with issucol and TachoComb. On the base of
empiric data, precise indications for each
apparatus were defined.
Conclusion: There are a lot of different apparatu-
to control oozing from liver raw surface. They
look very similar, but according to our data the
use of each apparatus is very specific and depends
the clinical situation and intraoperative
findings.
SURGICAL TREATMENT OF CIRRHOTIC ASCITES
(PARACENTESIS, PERITONEOVENOUS SHUNTING,
NARROW-LUMEN MESOCAVAL PTFE INTERPOSITION SHUNT
WITH FIBRIN SEALANT)
Severtsev A., Chegin V., Ivanova E., Bashilov
V. Med.Centre for Russian Government (MCRG);
Hosp. #51, Moscow, Russia
Ascites is common in patients with cirrhosis.
The most logical treatment of cirrhotic ascites
(CA) is hepatic transplantation. Because of the
obvious impracticability of that option and the
fact that the presense of CA usually means
terminal liver disease, a number of different
treatments had come to use. OBJECTIVE: To
estimate the clinical course of treatment of
patients with CA. PATIENTS & METHODS: In
1994-1995 we treated 34 patients with CA. The
treatment was begun from diuretics (180 mg/day
of furosemide and 400 mg/day of spironolactone)
during I0 days. On the basis of this test we
selected patients with diuretic-resistant
ascites (5 patients). Next stage was
paracentesis with albumin infusions. We
achieved success in 2 patients (positive
protein and amino-acids balance, rare admittion
to the hospital) during next 6 months. Failure
of treatment was in 3 patients. For these
patients used peritoneo-venous shunting
(Denver, USA) and after normalization of
nutritional status we used narrow-lumen.(up
to i0 mm) H-mesocaval interposition shunt with
PTFE vascular graft (Impra, USA). with the
support of vascular anastomoses by fibrin
sealant (Tissucol, Immuno, Austria). Long-term
successful results were obtained during next 5
months after the last surgery. CONCLUSION:
Aggressive approach for patients with CA could
prolong and normalize the life of this group.
111
P165 P166
FIRST EXPERIENCE OF SCLEROTHERAPY OF SMALL
LIVER LESIONS
Severtsev A., Pasternak N., Kornev A., Bashilov
V. Med. Centre for Russian Government (MCRG);
Hospital #51, Moscow, Russia
The question of treatment of the small liver
lesions (hemangiomas/NA with a diameter up to 4
cm, small non-parasitic cysts/NPC, non-
resectable metastases/MTS) is not cleared up
yet. OBJECTIVE: To develop an effective
treatment for small non-resectable liver
lesions. PATIENTS & METHODS: In 1995 we treated
2 patients with Polidocanol/PC/Aethoxysklerol
(intra-lesional injections) and 2 patients with
fibrin sealant/ Tissel/TS, Immuno (intra-
lesional injections) for small HA, 1 patient
with a single non- resectable gastric cancer
MTS in the liver (deep location, poor condition
of patient), 2 patients with NPC of the liver
(diameters were 2 and i0 cm). After
US-guided byopsy with frosen section
examination and cytology for NPC, we injected
up to 20 ml of 0.5% PC or up to 4 ml of rapid
TS. In case of single MTS (2.5 cm) we injected
(under US-guide) intraoperatively up to 5 ml of
1% PC into the lesion and apx. up to I0 ml of
0.5% PC para-lesionally. RESULTS: There a
reduction of HA sizes up to 50% on the 7th
post-procedure day (for PC and for TS). We
didn't find one NPC (2cm) during US-examination
on the 2nd month, and the 2nd NPC (10 cm) was
reduced to 3 cm. in diameter. In case of MTS
didn't discover new metastses and the size of
the treated MTS was the same on the 3rd month
after surgery. CONCLUSION: The use of PC and
fibrin sealant (TS) is an acceptable method for
the treatment of small lesions of liver.
EXPERIHENTAL STUDY ON HEPATIC CYTOPROTECTIVE FUNCTION OF NITRIC
OXIDE IN OBSTRUCTIVE JAUNDICE
H. Shimoda, H. gogure, g. Takagi
Second Department of Surgery, Dokkyo University School of edicine
Obstructive jaundice is closely associated with endotoxemia.
Bacterial endotoxin (LPS) and proinflammatory cytokines induce
nitric oxide(NO) synthase in various organs ia vivoas well as in
a variety of cells in qitraWe investigated whether NO production
is increased in obstructive jaundice by determining NOz-/NO3-
levels in portal plasma of common bile duct-ligated rats. While
the NOz-/N03- levels in portal plasma of control rats did not
change during the observation, a gradual increase up to day was
seen after the ligation. Although the NO synthase mRNA in the
liver was undetectable before the bile duct ligation, it was sub-
stantially induced by day following the ligation. A decreased
peripheral blood flow in the liver was observed in the bile duct-
ligated rats, which was further reduced by treatment of the rats
with the NO synthase inhibitor, L-NAME. We also investigated the
effect of bile acids on the induction of NO synthase in LPS-
stimulated J774 macrophages. Whole bile from control rats and, to
a lesser extent, from the bile duct ligated-rats inhibited NO syn-
thase induction in macrophages. Five different bile acids (cholic
acid, lithocholic acid, deoxycholic acid, chenodeoxycholic acid
and ursodeoxycholic acid), all inhibited NO synthase with
similar dose dependency.
In conclusion, obstructive jaundice is associated with increased
NO production which is derived from induced NO synthase. Elevated
NO may play an important role in obstructive jaundice, especially
in the peripheral blood flow in the liver.
P167 P168
CRYOTHERAPY FOR COLO-RECTAL HEPATIC METASTASES
M. SHROTRI, D. SHERLOCK, M. N. HARTLEY and G. d. POSTON
R'oyal'Liqerpool University Hospital, Liverpool/North
Manchester Hospital, Manchester.
Between October 1993 and October 1995 we have treated
45 patients with hepatic tumour using cryosurgery.
This paper presents our early results and attempts to
evaluate the role of this technique in the treatment
of patients with irresectable colo-rectal liver
metastases. 34 Patients (20 men and 14 women, with
mean age 60, range 40-75 years) treated had multiple
colo-rectal metastases (range ]-14). There were also
3 cases of carcinoid, renal cell carcinoma, neuro-
endocrine tumour and small bowel schwannoma
metastasis. There were 2 primary hepatomas. 17
patients underwent cryotherapy alone, 13 patients
underwent combined cryotherapy and resection. Of the
patients with metastases, the median number of lesions
treated was 5. Mean operative blood loss for cryo-
therapy, alone was 480 ml. (range 100-2.25 litre). 2
patients required re-exploration because of reactionary
haemorrhage. In this group patient died within 30
days of surgery related to ventricular arrhythmia
(2 others died of cardiac problems and.l died with
coagulopathy giving a total mortality of 4 in our
series of 45 patients). 6 months survival is 83%
(33% disease free), while the 12 month survival is
47% (8% disease free)." We conclude that cryotherapy
is still in its early stages of evaluation, but from
our preliminary data, it may offer survival benefit
for patients with previously irresectable liver
metastases of colo-recta tumours.
META-ANALYSIS OF EFFICACY OF BLOCKERS (/B) ON THE FIRST VARICEAL
BLEEDING (FVB) AND MORTALITY RATE IN CIRRHOTICS WITH HIGH RISK VARICES
(HRV) IN LONG TERM (24 mo) RCTS.
S. Slrin.qo, C. Carbone, R. Cogliandro, B. Misitano, G. Vitturini, L. Bolondi, R.
Corinaldesi.
Istituto di Clinica Medica Gastroenterologla Bologna. ITALY
In unselected cirrhotics the 2 yr. FVB rate is 25% and 40% of the episodes occur within the
first 6 (Hepatology 1994;20:66). Pts with HRV should have at least 10% higher FVB rate
respect the baseline risk. To minimise heterogeneity grouped RCTs with similar: a)FVB rate
in the placebo groups; b)length of f-u. RCTs whose placebo groups had 24 FVB rate
35% were defined RCTs. of HRV. We included RCTs: a)with an.average f-u of 24 too;
b)with 2 yr. FVB rate >35% in the placebo group; c)whose results were published in life tables
or data communicated by the Authors when requested. Only 3 studies had FVB rate in the
placebo group P_.25% but lesser than 35%. The overall number of pts included in the 3 RCTs
was 337:158 treated by I-B and 179 controls (CTR). The FVB rate in the pooled control
groups was 28.5% (range 22%-32%) and 35% of the episodes occurred within 6 too. The
overall sample size of 337 pts was that required to significantly decrease by 15% the r'mk of
FVB dudng 2 yr. ((x-.-5%; 1-1=90%) (Stat. Med. 1962;1:121). The Messori's meta-analysis
method for censored data was used (Comput. Progr.Meth.Biom.1993;40:261). The table show
the results: among treated patient there significant reduction of the FVB rate dudng and
after the first 6 too. However, surprisingly p-B did not reduce the mortality rate.
G Idaho et al. (1988)
I.MP.P. (1958)
T. Andreani et a1.(1990)..
%r,ed,uctlon,ofrisk,
CUM. BLEED. FREE RATE CUM .SURVIVAL RATE
<6mo >6mo mo >6too
-B-CTR /B-CTR ,p-B-CTR 14-CTR
100% 92% 95% 71% 93% 96% 88% 75%
95% 93% 73% 59% 94% 95% 54% 67%
95% 79% 95% 60% 80% 73% 67% 55%
13.3 24.2 -0.9 -1.2
Long rank OR 0.38 0.37 1.09 1.06
96% C.I). (0.16 088) (0.22.0.63) (0.44 2.39) (0.66 1.69)
Significance 0.01 <0.001 0A 0.4
CONCLUSIONS. Because the p-B efficacy has never been assessed in patients with HRV, I-B
long-term prophylaxys in pts with HRV should not be recommended. The long term effects of
I-B on FVB and mortality rate should be aseessei:l by appropriate RCTs in selected pts with
HRV
112
P169 P170
META-ANALYSlS OF EFFICACY OF ENDOSCOPIC SCLEROTHERAPY (ES) ON THE
FIRST VARICEAL BLEEDING (FVB) AND MORTALITY RATE IN CIRRHOTICS WITH
NON-HIGH RISK VARICES (NON-HRV).IN SHORT TERM (12 too) RCTS.
S.Sirinclo. C.Carbone, B. Misitano, L. Boiondi, R. Corinaldesi..
Istltuto di Clinica Medica Gastroenteroiogla. Bologna Italy
Studies of the natural course of oesophageal varices in unselected cirrhotics show that
among 1753 pts 14.9% bled during an average f-u of 12 mo and 63% of the episodes occurred
during the first 6 too. Pta with HRV should have at least 10% higher FVB rate respect the
baseline risk. To minimise heterogeneity we grouped RCTs with similar: a)FVB rate in the
placebo groups; b)length of f-u. RCTs whose placebo groups had 12 FVB rate <25%
defined as RCTs of non-HRV. We included RCTs: a)with an average f-u of 12 too; b)wlth
a FVB rate in the placebo group <_25% c)whose results were published in life tables
communicated by the Authors it' requested. Three of four RCTs mat all the inclusion criteria.
The overall number of pts included was 281:143 treated by ES and 138 controls (CTR). The
FVB rate in the pooled control groups was 16.6% and 56.5% of the episodes occurred within 6
too. The overall sample size of 281 pts exceeded that required to significantly decrease by
>15% the risk of FVB during 12 period (a-,.-5%; 1-190%). The Messofi's method for
censored data was used (Comput. Progr.Meth.Biom.1993;40:261). The table show the results:
ES did not reduce the FVB and mortality rate.
CUM. BLEED. FREE RATE CUM. SURVNAL. RATE
<6mo
ES CTR
W. Santangelo et al .(1988) 79 86
A.. Russo et al. (169). 100 95
PROVA (1,991) 89-90
% reduction of risk .3.0
Long rank OR 1.31
(95% CJ.| (0.6%2.38)
Significance 0.3
>6mo >6too >6too
ES CRT ES. CRT ES CRT
77-84 79-82 75-75
100-85 100- 95 100- 90
75- 76 74-83 72- 77
-1.2 -4.8 -1.7
1.07 1.39 1.11
(0.rd1-1.98) (0.74-2.64) (0.$3-1.96)
0.4 0.1 0.4
CONCt,USIONS. In short term studies ES do not have not effect in patients with non-HRV.
This might be due to the f-u length. Tdals of prophylactic ES with adequate sample size,
appropriate selection criteria and study design (long term f-u), should be undertaken in
cirrhotics with non-HRV
META-ANALISYS OF EFFICACY OF /3-BLOCKERS (/-B) ON THE FIRST VARICEAL
BLEEDING (FVB) AND MORTALITY RATE IN CIRRHOTICS WITH NON-HIGH RISK
VARICES (NON-HRV) IN LONG.TERM (12 mo) RCTS.
S. Sirinao. C. Carl)one, B. Misitano, R. Cogllandro, L. Bolondi, R. CorinaldeM.
Istituto di Clinica Medica GaMroenterologla Bologna. ITALY
Studies of the natural course of oesophageal varices in unselected cirrhotics show that among
1753 pts 14.9% bled dudng an average f-u of 12 and 63% of the episodes occurred dudng
the first 6 too. Pts with HRV should have at least 10% higher FVB rate respect the baseline
risk. To minimise heterogeneity we grouped RCTs with similar: a)FVB rate in the placebo
groups; b)length of f-u. RCTs whose placebo groups had 12 mo FVB rate 525% were
defined RCTs of non-HRV. This meta-analysis assessed the efficacy of ES in pts with non-
HRV in short term (12 too) RCTs. We included RCTs: a)with an average f-u of 12 too; b) with
FVB ratethe placebo group ;25%; c)whose results were published in life tables individual
patients data communicated by the Authors when requested. Two studies met the Inclusion
criteria. The overall number of patients included 246:121 treated with -B and 125
controls (CTR). The FVB rate in the pooled control groups was 18.4% and 39% of the episodes
occurred within 6 The overall sample size of 246 that required to significantly
decrease by >15% the risk of FVB during yr. ((x-,-5%; 1-190%). The Messori's meta-
analysis method for censored data was used (Comput. Progr.Math.Biom.1993;40:261). The
table show the results: -Bwere neither able to reduce the FVB and mortality rate.
CUM. BLEED. FREE RATE
-CTR
D. Lebrec et al. (1988) 00 94
PROVA (lg91) 92 91
% reduction of risk 5.3
Long rank OR 0.66
(96% C.I.) (0.19 1.66)
Significance.. 0.1
>0mo
#-CTR
85-80
3.7
0.81
(0.4 1.r)
0.3
<6mo
100-96
90-83
9.9
0.47
(0.18 1.23)
0.06
CUM .SURVIVAL RATE
>6mo
10.2.7 !.371
0.1
CONCLUSIONS. In patients with non-HRV I-B did not reduce both FVB and mortality rate in
the short term. This might be due to the shortness of f-u. Adequate sample size, eppropdate
selection cdteria and study design should be used for RCTs of prophylactic I-B treatment in
cirrhotics with non-HRV.
P171 P172
FIRST VARICEAL BLEEDING (FVB) IN UNSELECTED CIRRHOTICS: TIME-INTERVAL
EVENTS IN SHORT AND LONG TERM STUDIES,
S.Sirinqo, G. Vitturini, C. Carbone, B. Misitano, L.Bolondi, R.CorinaldesI.
Ist. dI Clin. Med. Gastroenterologia.-Bologna Italy.
To the FVB risk of cirrhotic patients enrolled in prophylactic RCTs the observed FVB in
the placebo groups should be compared with that reported in unselected cirrhotics. "[his report
is to asses the FVB rate in short (12 mo) and long term (24 mo) natural studies in
unselected cirrhotics with bleeding varices: through the whole f-u period and during the
first six months that is the highest risk period for FVB (Hepatology 1994;20;66). The data
reported obtained from the authors personal communications. The tables show the
FVB rate in 12 and 24 f-u studies.
TABLE 1-FVB rate in shod term (12 mo) studies.
Author
A.K. Burroughs et al. (1987)
NIEC (1988)
G. Minoli et al. (1990)
G. Piai et al. (1991)
G. K]eber at al. (1991)
G: Rig at al (1992)
s. siringo et al. (1994)
s. siringo et al. (1994)
Total
TABLE 2-FVB rate in long term (24 mo)
No
111
321
267
383
109
320
87
155
1753
F-U(mo)
17
12
12
12
12
14
12
12
studies.
Author No F-U(mo)
NIEC (1988) 321 23
G. Piai et al..(1991 383 25
G. Kleber et al. (1991) 109 21
S. Siringo et al. (1994) 87 24
S. $iringo et al. (1994) 155 25
Total 1055
Patients with bleeding
Total
24 (21.6%). 16(66.7%)
57 I17,.76) 35 (61.4%)
37 13.8,6 26 (70.0%)
51 (13.3%) 31 (60.8%)
26 (23.8%) 16 (61.5%)
3e(12.1%) 8 (.0%)
11 (12.6%) 9(81.8%)
16(10.3%) 14(87.5%)
261(14.9%) 165(63.0%)
Patients with bleeding
Total
85 (26.5%) 35 (41.2%)
90 (23.5%) 31 (34.4%)
32 (29.3%) 16 (50.0%)
22 (25.3%) 9 (40.9%)
37 (24.0%) 14(37.8%)
268(2.2%) 105(39.5%)
This review confirms that the first six months of f-u is the highest risk period for FVB. Risk
criteria for early and late FVB should be identified. The present survey gives figures of baseline
risk for the FVB in unselected cirrhotics that should be taken into account in evaluating the
selection criteda for prophylactic trials and pooling studies for meta-analysis.
HEPATIC ARTERY THROMBOSIS FOLLOWING LIVER
TRANSPLANTATION DEVELOPING A THERAPEUTIC
STRATEGY
G. Spiliopoulos, B. Meunier, M.A.C. Machado, C. Stasik, J. Roumeas, B.
Launois
Department ofDigestive Surgery and Transplant Unit, CHR Pontchaillou,
Rue Henri Le Guilloux, 35033 Rennes, France
Between 1978 and 1995, 236 liver transplantations were performed on
204 patients. 20 patients (8.5%) developed hepatic artery thrombosis
(HAT) post-operatiVely, all files were reviewed retrospectively in an
attempt to define a therapeutic strategy for this potentially lethal
complication.
Materials and methods there were 11 males and 9 females with a mean
age of 44.4 years (range 28-66 years). 14 patients (70%) had anomalous
arterial anatomy of their liver graft and or incongruity of the arterial
anastomosis. 4 patients had concomitant graft rejection. HAT occurred
within 30 days of transplant in 16 patients and after 30 days in 4 patients.
8 patients underwent revision procedures, ofwhich ultimately required a
retransplant. 4 patients underwent immediate retransplantation. patients
had radiologic or endoscopic interventional treatment and of these
patients later underwent bilio-enteric bypass.
Results 11 patients (55%) died, the primary cause of death being HAT
in 10 patients. 9 patients (45%) are alive with a follow-up ranging between
4 and 66 months. Liver function was normal in patients and poor in 4
patients.
Conclusions Early diagnosis of HAT is necessary for revisional surgery
to succeed. Immediate retransplantation is indicated in patients with a poor
general status who would not survive unsuccessful revisional surgery and
in patients with aberrant graft arterial anatomy or complex arterial
anastomoses because of the high risk of rethrombosis. Infected patients are
contraindications to retransplantation and can benefit from a
multidisciplinary approach (medical, endoscopy, interventional radiology).
Infections and late biliary complications following late HAT can be treated
by bilio-enteric anastomosis of liver resection after initial radiologic or
endoscopic management.
113
P173 P174
SURGICAL TREATMENT OF HEPATIC METASTASES FROM
COLORECTAL CANCER A COMPARATIVE STUDY BETWEEN
"CONVENTIONAL" AND POSTERIOR APPROACH TECHNIQUE
PRELIMINARY REPORT
G, SPILIOPOULOS, B. MEUNIER, N. PALASCIANO, M.A.C.
MACHADO, C. STASIK, F. GONZALEZ, B. LAUNOIS.
Department of Surgery and Transplantation Unit. University of Rennes
France.
Surgical resection still represents the best chance of improving survival
for some patients with hepatic metastases ofcolorectal origin.
The aim ofthis study is to compare the outcome ofhepatic resection for
metastases ofcolorectal cancer in two similar groups of patients using two
different techniques ofhepatectomy.
The group was constituted of 25 patients (15 men and 10 women,
with mean age of 63.1 years range, 32 to 80 years). The surgical
procedure was hepatic resection employing the posterior approach of the
hepatic hilum technique with intermittent clamping ofglissonian sheaths.
The group II was constituted of 23 patients (12 men and 11 women,
with mean age of 63.8 years range, 40 to 73 years). The surgical
procedure was hepatic resection employing the conventional technique
with in mass continuous clamping ofthe hepatic pedicule.
There was no statistical difference between the two groups concerning
sex, age and number of metastases. The duration of ischemia was superior
in the posterior approach patients (group I) with a mean of 84.2 minutes
against 37.5 minutes of the group II (p < 0.0001). There was no influence
of the ischemia time in the postoperative hepatic function, postoperative
course or recovering time.
The survival rate was superior in the group of the posterior approach
technique: 778.8 + 410.2 days (group I) Vs 572.5 +/- 349.7 days (group II).
Although this difference, it was not statistically significant (p 0.14).
We concluded that the posterior, approach procedure is a.feasible and
safe technique allowing to perform, segmentary and subsegmentary
anatomical resection. This new technique seems to improve the survival of
patients with hepatic metastases of colorectal cancer. This results still has
to be confirmed by subsequent series with greater number ofpatients.
ALTERED EXPRESSION OF DIPEPTIDYL PEPTIDASE IV IN
RESECTED HEPATOCELLULAR CARCINOMA
B. Stecca, B. Nardo*, I. Capri, B. Santoni*, A. Mazziotti*, P. Chieco, A.
Cavallari*. Istituto di Oncologia "F.Addarii", Bologna, *Clinica
Chirurgica II, Univcrsit di Bologna.
INTRODUCTION: Several malignant epithelial tumors often reveal
alterations in protease expression. In this study we investigated the
activity and the distribution of the exopeptidase dipeptidyl peptidase IV
(DPP IV) in human hepatocellular carcinoma (HCC).
MATERIALS AND METHODS: Twenty-one cases of surgically
resected Hoe were selected. Samples taken from the central and/or
peripheral part of the neoplastic nodule and from the surrounding
cirrhosis were analyzed. Control specimens were additionally obtained
from normal liver of 7 patients which underwent surgery for non-
neoplastic pathology. Frozen sections were incubated at 25C for 10 min
in a medium containing the specific substrate and Fast blue B salt and
DPP IV activity was quantified by image cytometry.
RESULTS: In control specimens DPP IV hepatocyte staining was
confined to the bile canalicular domain, with a centrolobular gradient.
The DPP IV expression was altered in all HCCs examined, both from
central and peripheral parts of the tumor; 2/21 cases showed loss of DPP
IV activity and 19/21 remodeling of DPP IV distribution on bile
canalicular domain. In 17/21 (81%) HCCs also staining of basolateral
hepatocyte membrane was found. The surrounding cirrhotic tissue of 20
patients revealed the same expression observed in control samples, one
case presented an altered distribution of DPP IV.
CONCLUSIONS: Our data indicate that alterations in DPP 1V
distribution and activity occur in HCC. Being the difference in DPP IV
pattern between neoplastic and non-neoplastic tissue clearly evident,
cytochemical determination of DPP IV could be useful support to the
diagnosis of Hoe, including intra-operative pathological diagnosis.
P175 P176
PARASITE CYSTS OF THE LIVER
G. Stoianov, N. Damlanov, G. Genehev, P. Purvanov, A.
Angelov, A. Dimitrov, P. lanovska
Clinic of Abdominal Surgery,University Hospital "Queen
Josnila" Sofia, Bulgaria
502 patients with liver eehinsis have been operated.
since 1971 up to 1994 and 125 of them were with a
recurrence of the diseases. The diagnosis was confirmed
by sonography (78.9%) and computer tomography
(90.5%). The immunological examinations were positive
as follows: Weinberg's reaction (72.1%), latex
agglutination (80.1%) and passive hemagglutination
(86.7%). In our opinion the hepatoseintigraphy has no
advantages for the diagnosis of the liver echinsis.
This examination was positive only in 63.2 % of the
patients. The most frequent applying method was
sonography combined with immnologieal examinations.
Thus the diagnosis was confirmed by 90% ofthe patients.
Different surgical methods were applied, but the "closed"
methods were of main importance. We prefer to use the
excision of the prominent part of the cysts and
omentoplastie with tissue glue. The intraoperative
sonography is an useful method for detecting small cysts
in eases of polyechinsis. The mortality rate was
3.98%. The operated patients are followed up and criteria
for prognosis and treatment are created.
HEPATIC CRYOTHERAPY FOLLOWED BY HEPATIC
ARTERIAL 5FU FOR COLORECTAL LIVER METASTASES.
Richard S Stubbs, Michael WC Booth and Majeed H Alwan. The
Wakefield Clinic for Gastrointestinal Diseases, Wakefield Hospital,
Wellington, New Zealand.
Methods: Patients were selected for treatment on the basis of 1)
metastatic colorectal cancer confined to the liver, 2) unsuitability
for resection, and 3) less than 50% liver involvement. Hepatic
cryotherapy was performed under general anaesthetic using a
Cryotech LCS 2000 system with intra-operative ultrasound
monitoring. An hepatic artery Infuse-a-port was inserted into the
gastroduodenal artery at the time of surgery for subsequent
administration of arterial 5FU which was given on an outpatient
basis by continuous infusion for 4 days in 4-weekly cycles using a
disposable infusion pump. Follow-up monitoring included regular
CEA estimations, 3-monthly CT scans of the liver and CXR.
Results: 24 patients had a median of 8 lesions treated (2-23) and
have been followed for a median of 30 months (20-47) from liver
tumour diagnosis. 15 patients were thought to have received
complete macroscopic tumour destruction at the time of surgery
and 9 had incomplete destruction. Post-operative complications
occurred in 10 patients but were generally not serious. There was no
30-day mortality and the median hospital stay was 9.5 days (6-23).
Arterial chemotherapy was well tolerated with severe side effects
occurring in only 12% of cycles. 12 of 22 patients showed
progression of tumour within the liver within 9 months of
cryotherapy. Of these 7 received less than the planned 6 cycles of
chemotherapy compared with only of the 10 who did not have
progression in the liver by that time (p 0.03, Fisher Exact).
Median survival from cryotherapy is 17 mo (7-33) and from liver
tumour diagnosis is 22 mo (10-39). Comparison with historical
untreated controls suggests a survival advantage of approximately
12 months for those receiving the treatment.
Conclusions: This initial experience indicates the safety of the
technique and the results suggest there' is an encouraging extension
of life for those so treated.
114
P177 P178
SURGICAL TREATMENT FOR MANY LIVER METASTASES FROM
COLON CANCERS
Y. Sugawara, K. Kubota, A. Maema, K. Inoue, Y. Bandai, M. Makuuchi
Second Department ofSurgery, University of Tokyo, Tokyo, Japan
A total of 12 patients with metastatic liver tumors from colorectal cancers were
treated surgically atourdepartmentfrom October 1994throughDecember 1995.
Two of the 12 patients had many (more than 10) liver metastases. The first
patient, whohad undergonesigmoidectomy forcolon cancer,was foundto have
synchronous liver metastatic lesions and referred to our department. In the
other patient, who had received hemicolectomy for the ascending colon cancer,
three liver metastatic lesions were found in the left lateral segment and the
anterosuperior area, 14 months after colectomy, they were resected. Two
months later, the patient was found to have other metatatic lesions andreferred
to our hospital. The clinical data for the patients were listed in the Table.
Preoperative careful examinations, not only oftheliver function and volumebut
also of the location and number of metastatic lesions suggested that all the
lesions couldbe resected safely. During surgery,all themetastaticlesions were
visualizedusingintraoperativeultrasonoaphy, whichcontributedto leaving no
lesions behind in theliver. The levels ofserum carcinoembryonic antigen fell
rapidly to below 5 ng/ml within month after surgery in both patients. We
believe that the presence ofmany metastatic lesions from colon cancers is not
contraindication for hepatic resection if theprimary lesion has been curatively
resected. 1
primary No. and location Time between colon ICG It and ype ofhepatic weight of
(age/gender) ofmetastatic and hepatic resection liver volume resection resect
location lesions specimen
(46/F) sigmoid 17 (III, IV, VI, synchronous 3.8/dl 152cm enucleation 155
colon VII, VIID
(49/M) ascending (I, IV, V, VII, months 4.6%/945cm segmentectomy IV 322
colon VIID & e_._n_uclea_._t.ion
The Roman number indicates that ofCouinauds segments. Indocianine green retention rain.
DIAGNOSIS OF PORTAL VEIN THROMBOSIS
SzAntov M., Goncalvesovl El., Kupovi V.,Makaiovl I2.,Synak IL2
3 rd Depart. ox Medicine, Dept. of Nuclear Medicine2, Medical School
of Comenius University, 1st Depart. of Medicine1, Postgraduate Me-
dical Institute, Bratislava, Slovakia
Portal vein thrombosis (PVT) is usually seen as a complication of
malignant or inflammatory abdominal disease, splenectomy, trauma or
liver cirrhosis. It may occur also as a complication of myeloproliferative
diseases and hypercoagulative states. During the last 2 years we had 9
.cases of PVT in our department diagnosed by color doppler
ultrasonography (CDU). Complete thrombosis was described in 2,
incomplete in 3 and findings of cavernomatous transformation of portal
vein was present in 3 patients. The following diseases were identified as
causes: hepatocellular carcinom (1), primary sclerotizing cholangitis (1),
myeloproliferative disease (2), status after cholecystectomy (1), chronic
active hepatitis (1), acute attack of pancreatitis (l),status after new-born
trauma with omphalitis (1). In one patient the etiology of PVT was
unknown. Six of the patients were referred to our department for bleeding
from esophageal varices, for ascites ofunclear etiology, and for chronic
pancreatitis with suspection of pseudocyst. In 8 patients coeliaco- and
mesentericography (AG) and computer tomography (CT) were performed.
Four of them were examined also by dynamic scintigraphy with 99 Tc-
colloid and the arterio -portal ratio was calculated. Arteriography was
conclusive in 4 patients, in whom it confirmed diagnosis while CT did so
only in 3 cases. The arterio portal ratio ofhepatic blood flow proved to be
inversed, indicating relative arterialization ofthe liver as a consequence of
PVT in all the patients examined.
In conclusion, CDU was found to be the most effective of the commonly
used non invasive and invasive imaging methods when performed by an
experienced examiner. CDU is indicated in patients with hemorrhage from
esophageal varices, particularly when liver disease does not explain the
presence of varices. Method of choice signalising changes in liver
hemodynamics appears to be dynamic scintigraphy with calculation of
arterioportal ratio.
P179 P180
EXPERIMENTAL STUDY ON WARM ISCHEMIC LIVER INJURY
COMPARISON OF CONTINUOUS VERSUS INTERMITTENT
ISCHEMIA-REPERFUSION
K. Takagi, H. Kognre
Second Department of Surgery, Dokkyo University School of Medicine,
Mibu, Tochigi, Japan
The tolerance of the liver to ischemia is controversial. The aim of this
study to compare its effects during continuous versus intermittent occlusion
of blood flow to the liver, measured by mean arterial pressure, hepatic tissue
blood flow, serum levels of GOT, GPT, arterial ketone body ratio, hepatic tissue
adenine nucleotides, energy charge (EC), hepatic tissue lipid peroxide (LPO),
hepatic tissue superoxide dismutase (SOD) and histological examination.
Male Wistar rats were allocated to have either continuous duration of
ischemia of 60 minutes (group A), intermittent inflow occlusion (four
15-minutes periods of ischemia separated by minutes reperfhsion, group B)
sham operation (group C). A temporary perioperative porta-caval shunt
used to exclude the effects of splanchnic stasis and allow independent
study of the effects of hepatic ischemia. Wilcoxon-test used for statistical
analysis.
In group A, the hepatic tissue blood flow significantly increased at 120
minutes after repeffusion and EC significantly increased at 30 and 120
minutes after reperfusion. There was significantly lower hepatic tissue LPO
level at 120 minutes after reperfusion and significantly higher hepatic tissue
SOD level at 30, 60 and 120 minutes in group A. Histological changes
milder in group A, which reveals ischemia-reperfusion injury less severe
and viability of the liver was well preserved.
It is suggested that for given duration of ischemia of the liver, continuous
inflow occlusion is more beneficial than intermittent occlusion during liver
surgery.
115
P181 P182
MORTALITY AND MORBIDITY OF 210 ELECTIVE HEPATIC
RESECTIONS
H.Taniguchi, A.Takada, T.Mugitani, M.Masuyama, H.Koyama, H.Tanaka,
M.Hoshima, K.Kitamura, A.Hagiwara, T.Yamaguchi, K.Sawai, and
T.Takahashi
First Department of Surgery, Kyoto Prefectural University of Medicine,
Kyoto, Japan
Between April, 1988 and October, 1995, we had 210 elective hepatec-
tomies. These were performed on 87 patients with hepatocellular
carcinomas, 74 with hepatic metastases, 26 with cancer ofthe biliary tree,
21 with benign disease, and 2 miscellenous. Thoracoraparotomy was
performed on 84 hepatectomies out of 210. Hepatecomies for cirrhotic
liver were performed for 71 cases. We had 5 operative death (2.4%) and 4
hospital death (1.9%). The morbidity of all patients was 54.4% including
minor complications. Major complications was occured in 47 patients
(22.4%) including 6 moderate (2.9%) and 15 severe (7.3%) pleural effusion,
12 ascites (5.8%), 9 pneumonia (4.4%), 4 bile fistula (1.9%), 3 abdominal
abscess (1.5%), 4 hepatic coma (1.9%), and 6 postoperative bleeding
(2.9%). There were no valuables which explained pneumonia, bile fistula,
abdominal abscess, and hepatic coma. Wound dehiscence and pleural
effusion were explicable by prothrombin (t=-2.41, p=0.017) and incision
(t=5.571, p<0.001), respectively. Ascites was explicable by operation
time (t=2.245, p=0.026), blood transfusion (t=2.232, p=0.027),
prothorombin time (t=2.158, p=0.032), and serum total bilirubin (t=-2.026,
p=0.044). Postoperative bleeding was explicable by blood loss (t=3.922,
p<0.001), blood transfusion (t=-2.732, p=0.007), and additional surgery
(t=2.151, p=0.033).
THE ROLE OF PROSTACYCLIN ON LIVER REGENERATION IN
RATS: ASSESSMENT OF DNA INCORPORATION IN DIFFERENT
LOBES FOLLOWING 40% PARTIALHEPATECTOMY.
R.Troisi;. U.J. Hesse and B. de Hemptinne. Department of Surgery, Universi-
ty ofGhent, Belgium.
Basic mechanisms involved in liver regeneration are still under debate. The
Araehinoid Acid Cascade AAC has been implicated in the early prereplicati-
ve hepatoeyte development that is activated by liver resection. Purpose of this
study was to analyze be regeneration of different liver lobes following 40%
hepatectomy and the contribution ofprostacyclin in this process.
Anlmab and methods: Twentyfour male Wistar rats, weighing 225 to 318 g
underwent 40% partial hepatectomy PH by resection ofthe Left Lateral Lobe
LLL ). [H3]Thyrnidine, 1.0 mCi/g body weight was injected intravenously 2
hours before sacrifice and animals were killed 24 hours after the PH. Regenera-
tion ofthe remaining liver lobes Left and Right Anterior RAL LAL versus
Left and Right Posterior RPL LPL was separately determined by
[H3]Thymidine nuclear DNA incorporation. Animals were randomly assigned
to two groups. Group I: control animals weighing 257 4- 26g treated with
saline injection at the time of PH n 12 ). Group II, 12 animals (weighing
2514- 26g were given 10/ag Iloprost, PGI2 analogue ILOMEDINE She-
ring AG Pharma at the time of PH.
Results: The [H3]Thymidine incorporation in the nuclear DNA for RAL vs
LAL and RPL vs LPL in both series was statistically not significant. However
in the control group, counting of 7237 4- 2254 mean 4- sd dpm x mg DNA
for anterior lobes RAL + LAL versus 9960 4- 2825 dpm x mg DNA for
posterior lobes RPL + LPL ), p .046 was recorded. In the Ilomedine
group the corresponding values of 16938 8629 dpm x mg DNA for the RAL
+ LAL versus 31809 4- 10072 dpm x mg DNA for the RPL + LPL p .045 ).
The difference between the two series was statistically significant p =. 045 ).
Conclusions: 1, ARer 40% partial hepatectomy in rats, the regeneration of
different remaining liver lobes RAL vs LAL and RPL vs LPL is quite similar.
2. The posterior liver lobes regenerate more than the anterior, and this for
unclear reasons. 3. Administration of PG2 analogue (Iloprost) is associated
with a significant increase of nuclear DNA incorporation compared to the
control animals, enhancing the liver regeneration in toto and of posterior liver
lobes, eonfirmin8 that the initiation ofDNA synthesis is mediated by metaboli-
tes ofthe Arachinoid Acid Cascade.
P183 P184
GAMMA-GLUTAMYLTRANSIi'ERASE ISOENZYME TYPES IN
SERUIVI AS A MEAN FOR DISCRIMINATING BETVCEEN
BENIGN LIVER DISEASES AND LIVER TUMORS
L.Tureck, ,V.KupEov, *M.Szntov, E.Uhlikov K.Lakti
Inst. Med.Chem., Biochem. & Clinical Biochemistry and *Dept.Medicine,
Med.Sch., Comenius University, Bratislava, Slovakia
The enzyme gamma-glutamyltransferase (GGT, EC 2.3.2.2) is a
glycoprotein present in various human tisuues and organs. GGT in serum
is generally thought to originate from the liver, because activities are
increased only in hepatobiliary disesaes. There are several reports of
binding of GGT to lipids or lipoproteins. The different isoforms of GGT in
serum, particularly those found in the course of hepatobiliary diseases, are
associated with various lipoproteins. Sacchetti et a1.(1988) studied the
association of GGT to lipoproteins in patients with hepatobiliary diseases.
In our work, we have also evaluated the percentage of GGT complexed
with low (LDL) and very low (VLDL) lipoproteins in chronic liver diseases
(cirrhosis, chronic hepatitis) and liver malignancies. The comparison of
the percentage of GGT activity after VLDL+LDL precipitation in sera
from cirrhotic and neoplastic patients showed, that the percentage of GGT
associated with LDL+VLDL is much greater in sera ofpatients affected by
neoplasia as compared to sera of cirrhotic patients. In the work of
Sacchetti et a1.(1988) a cut-off of 20 U/I of GGT complexed with
LDL+VLDL was used. Our results showed, that by this cut-off level the
diagnostic sensitivity for liver tumor patients was 80%. But the diagnostic
specificity towards cirrhosis and chronic active hepatitis was only 29% and
50% resp. The increase of cut-off level of GGT complexed with
lipoproteins to 40 U/I increased the diagnostic specificity tumors versus
chronic active hepatitis to 87% and tumors versus cirrhosis to 65%. A
comparison ofthe primary and metastatic liver tumors population with the
cirrhosis and chronic hepatitis groups gave a diagnostic efficiency of 73%
at a cut-off level of 40 U/I. The estimation of the percentage of GGT
complexed with LDL+VLDL is a useful addition to estimation of total
GGT and other laboratory tests that serve to discriminate chronic
hepatobiliary diseases from liver malignancies.
DYNAMIC THREE-DIMENSIONAL SPIRAL CT IMAGING OF THE LIVER.
AN AID TO PLANNING PARTIAL LIVER RESECTIONS.
T.M. van Gulik, M.S.van Leeuwen*, J.Noordzij*, H.Obertop, D.J.Gouma.
Dept. of Surgery, Academic Medical Center, University of Amsterdam and
Dept. of Radiology*, University Hospital Utrecht, The Netherlands.
Hepatic surgery is based on a precise knowledge of the segmental
anatomy of the liver. The segmental division of the liver is determined by
the intrahepatic ramifications of the portal vein and distribution of the
hepatic veins. A technique has been developed by which life-like three-
dimensional (3-D) models of the portal and venous hepatic systems are
created, using spiral CT. Video representation of this 3-D model allows
accurate assessment of the portal system and hepatic veins, in relation to
the liver lesion(s). Application of this technique in the work-up of 20
patients considered for partial liver resection, is reported. Methods: Spiral
CT scans (SR 7000, Philips Medical Systems) were obtained using 5mm
slices, reconstructed at 2mm intervals. 3-D renderings of the liver
parenchyma and the portal and hepatic veins were blended into a color
video representation. Patients and results: 20 patients (age 28-70 years)
underwent the 3-D imaging procedure, on which basis a strategy for
resection was planned. Anatomical vascular variations were noted; the
identification of accessory hepatic veins proved particularly valuable.
Dynamic (video) representation provided most information. 13 pats. had
1-6 metastases, 3 pats. had a hepatocellular carcinoma, 2 pats. had an
adenoma, pat. had FNH and pat. multiple carcinoid lesions. 3 pats.
were considered unresectable due to Iocalisation or extent of the lesions
(confirmed by laparotomy or laparoscopy). The following resections were
undertaken in the remaining 17 pats.: right hemihepatectomy (n=7),
extended right hemihepatectomy (n=4), left hemihepatectomy (n=2),
segmental resections (n=4). All resections were histologically radical,
postoperative mortality was 0. Samples of the 3-D video representations
are shown, displaying the liver at different angles, as a corrosion cast
specimen in which the lesion is projected inbetween the portal (red) and
hepatic venous (blue) branches.
Conclusion: Video representation of 3-D spiral CT images of the liver
improves accurate assessment of lesion(s) in relation with the portal and
hepatic veins, and proved a valuable tool in planning liver resections.
116
P185 P186
SURVIVAL AFTER LIVER RESECTION OF HEPATIC
METASTASES FROM COLORECTAL CANCER
E.Veloso, E. Cugat, R. Almenara, A. Navarro, E. Mufioz, P.
Collera, C. Marco.
Department of Surgery. Hospital Mutua de Terrassa. University of
Barcelona. Barcelona, Spain.
INTRODUCTION:There are many estudies, since Foster described
liver resection, that show a 5 years overall survival between 20 and
45% after hepatic resection for colorectal metastases (HM). This
survival is greater than in patients with metastases no resected.
MATERIAL AND METHODS:Between 1987 and January 1994,
548 patients underwent surgery for colorectal cancer at our
hospital. Of this group 23 patients underwent surgery for HM.
Number, size, localitation, presentation, surgical treatment, and
survival were analized in this study.
RESULTS:In 10patients HM were synchronous, and metachronous
in 13. Diagnosis was made by routine ultrasonography or CT scan
examination in 18 patients, increased CEA blood levels in 2, and
in 3 patients during operation. HM were single in 14 patients, and
multiple in 9. Sizes were between 0.5-5cm. Surgical treatment was
local resection in 11 patients, segmentectomy in 5, and
hepatectomy in 7 (6 right, and left). Morbidity was: subphrenic
abscess 2, hepatic abscess 1, pleural efusion 1, transitory liver
failure 1, urine infection 1. There was no operative mortality.
During follow up seven patients died after 5,10,11,12,25,30 and
37 months. Five year overall survival was 64%.
CONCLUSION: As is well known survival after resection depends
on number of HM (<4); colorectal tumor stage; disease-free
interval; positive hepatic nodes; free margen ofresection (> lcm).
When a radical resection of HM is possible without leaving
residual disease, survival is better than if no surgical resection is
performed, even if previous conditions are not present.
MAJOR HEPATIC SURGERY. EXPERIENCE WITH 100 CASES
Emilio Vieente M.D., Javier Nufio M.D., Manuel Devesa M.D., Antonio Barrasa
M.D., Adolfo L. Buenadieha M.D., Jesds Igea M.D., Yolanda Quijano M.D.
The improvement of surgical and anesthetic techniques have contributed to
progress in liver surgery. So, hepatic resection is now associated with low
morbidity and mortality rates.
Since 1986, 100 patients, who underwent major hepatic surgery, were
reviewed. The indication for liver resection was: Hydatid cysts (36), non-parasitic
cysts (3), cystadenomas (3), cystoadenocarcinomas (1), adenomas (2),
myoangiolipomas (2), primary malignant neoplasms (18), 14 of them in patients
with liver cirrhosis, secondary malignant neoplasms (25), carcinoma of the
gallbladder (4), carcinoma of bile duct (2), monolobar Caroli's disease (2),
adrenocortical carcinoma (1) and gastric cancer (1) with infiltration of right and left
hepatic lobe respectively.
The surgical technique used for echinococcal and non-parasitic cysts was:
Total excision of the cyst (23), non anotomical liver resection (11), left
hepatectomy (1) and left lateral sectorectomy (1). The anatomic resections used
were: Right lobectomy (11), right trisegmentectomy (5), left lobectomy (9), left
lateral sectorectomy (3), bisegmentectomy (13), segmentectomy (3) and
metastasectomy (6). In four patients, liver resection was associated to
duodenopancreatectomy (2), portal vein resection (1), adrenal gland resection (1),
total gastrectomy (1) and left colectomy (1).
Intraoperative ultrasound was used in 36 patients. Pringle occlusion prior
to parenchymal division was applied in 49 patients for 10 to 75 minutes. One
patient required total vascular exclusion. In 31 patients, the ultrasonic dissection for
fragmentation of tissue was used.
Four patients died of hepatic failure after liver resection. Two of the four
dead were of patients who had cirrhosis ond hepatocellular carcinoma and
underwent hepatic bisegmentectomy. Two other patients who died had a massive
liver metastasis from colorectal cancer and required right trisegmentectomy.
Moderate esteatosis were confirmed in the remanent liver parenchyma. We conclude
that major hepatic surgery is now performed with good results. Careful patients
selection remains crucial en order to decrese the incidence of postoperatory hepatic
failure.
P187 P188
HEPATIC NODULES IN PATIENTS WITH BUDD-CHIARI
SYNDROME.
C. Vons, J. Belghiti, V. Vilgrain, S. Hillaire, S. Erlinger, D. Franco, D.
Valla.
Service de Chirurgie, H6pital Antoine B6clre, Clamart, Services
d'H6patologie, de Chirurgie, de Radiologie, H6pital Beaujon, Clichy,
France.
The occurence of nodules in the liver has been described in a few
patients with a Budd-Chiari syndrome (BCS). However the incidence
of such nodules, their nature and their significance remain unknown.
The aim of this work was to determine the incidence, histological
features and evoluti.on of hepatic nodules in patients with BCS. From
1981 to 1995, 65 patients with BCS secondary to a hypercoagulable
state were treated and regularly followed-up. There were 41 women
and 24 men. Mean age at time of diagnosis was 27 years (range: 11-
54 years). Porto-systemic shunts (PSS) were performed in 39 patients
and medical treatment in 26. All but 15 patients had long-term
anticoagulant therapy. Hepatic nodules were found on liver US, scan
or MRI in 16 patients (25%, 10 women and 6 men) during follow-up.
Mean interval between diagnosis of BCS and discovery of liver
nodules was 5 years (range: 1-14 years). Hematologic disorders in
these 16 patients were primary myeloproliferative disease in 12,
paroxysmal, nocturnal hemoglobinuria in 3 and antiphospholipid
syndrome in 1. Nodules were present in 10 out of 39 patients after
PSS (26%) and in 6 out of 26 patients medically treated (23%). The
interval between diagnosis of BCS and occurence of nodules was
similar in patients with or without PSS. In 10 patients, hepatic nodules
were diffuse in both lobes of the liver. In 6 patients, there were to 3
nodules. In 9 patients, biopsy (5 during laparotomy) was performed
and led to the diagnosis of regenerating macronodule in 4 cases, an
area of sinusoidal congestion, hemorrhage, necrosis and neoductular
proliferation simulating a tumor in 2 cases, and hepatocellular
carcinoma in 3 cases. These last 3 patients had cirrhosis but the 13
others did not. One patient experienced rupture of a regenerating
macronodule as a result of excessive anticoagulation. One patient died
of multinodular hepatocellular carcinoma. In 2 patients hepatic
nodules were no more visible on liver imaging one year after
discovery. In conclusion, hepatic nodules are commonly found in
BCS. They are not enhanced by PSS. Nodules may be regenerating
macronodules but may also be hepatocellular carcinoma particularly
in patients with cirrhosis.
RESULTS OF LIVER RESECTION IN POLYCYSTIC KIDNEY
LIVER DISEASE.
C. Vons, D. Chauveau, J.P. Grtinfeld, D. Franco.
Service de Chirurgie, H6pital Antoine B6clire, Clamart, et Clinique
N6phrologique, H6pital Necker, Paris, France.
Partial hepatectomy and total hepatectomy followed by orthotopic
liver transplantation have been advocated in patients with polycystic
kidney liver disease (PKLD) and massive enlargement of the liver.
The purpose of this work was to evaluate the results of partial liver
resection in 9 consecutive patients. Between 1991 and june 1995, 9
patients (8 women, man; mean age: 55 years, 33-66) with
PKLD and symptoms related to massive liver enlargement (pain,
abdominal distension, gastroesophageal reflux, dysphagia, and
decrease of mobility) have had partial hepatectomy. All patients had
severe arterial hypertension, and chronic renal failure. Three patients
were on chronic hemodialysis. Four patients had a left lateral
lobectomy, 4 had a left hepatectomy. One patient had a right
hepatectomy extended to segment IV. In three patients anatomical
resection was associated with removal of cysts in another part of the
liver. In the three patients with end-stage renal failure a right
nephrectomy was also performed. Three patients required blood
transfusion (33%). There was no intraoperative or postoperative
death. All patients had postoperative ascites. This was easily treated
in patients with persistent diuresis. In hemodialysed patients ascites
persisted and was eventually treated by renal transplantation in one
patient. One patient (11%) died on day 45 postop of ascites
infection. Another patient had no improvement because of persistent
ascites and is presently awaiting for liver and kidney
transplantations. All other patients have had marked improvement in
their status. These results suggest that partial liver resection is
efficient in patients with PKLD and massive and symtomatic
enlargement of the liver without end-stage renal failure. In patients
with chronic hemodialysis combined liver and kidney
transplantations might offer better results.
117
P189 P190
A CASE OF HEPATOCELLUAR CARCINOh WITH LYMPH
NODE METASTASIS OF THE MEDIAgl]NUM
D. Wada. S. Yogita, M.Ishflmwa, I4Miyake, Y.Fukuc'la, 1VHm'ac'la, S.Tashim
1stDepm'tmentofSgey, UniversityofTokushinm, Tokushmm. Japan
We report suaessful smgical of hepatocellular cahnoma (HCC) ith
lymph node metastasisof the mediastinmn. A 56-yem,old man, vho under,vent
leit lateral segmentectomy for HCC with lymph, node llletastass of the
hepatoduodenal ligmnent (No 8a,12p2) in depm'tment 30 nmnths ago.
Histopathological finding of the minor Edmondson m of HCC. He
complained of fever unknown origin 26 moths after stages,. Chest. X-my fihn
showed mass shadow in the right superior mediastfiamn. The
considered to be svollen mediastinal lymph node 15' CT mad MRI. We
diagnosed solitmy metastasisto the mediasdnum fi'om HCC of thehver.
Right posterolateml thomcotomy de mad the mediasfinal tumor about. 5
cm,in dimneter resected. Histopathological findings mv(led that. the tumor
metas'taticlymph node of HCC. Ihepatientis st.ill alive withoutmctu'mnce
11 months after reoperation.
Lymphatic metastasis of HCC is uncommon, and mediasfinal metastasis
of HCCis exceechglymm, lh'om 1985 to 19.'-)5, 120 With HCCwereoperated
in depm'tmen I4,mph node metastasisof HCC observed in 5ca (4.A).
This considered that nediastinal lymph node would 1 metastasized via
b,mphatic pathway fi'om the liver to the, mediasmmn. Chaly 6 (rise reports have
been found (vnmming HCC with mlitmy metastasis to the mediastinum in
Japane hterature.
ADJUVANT CHEMOTHERAPY AFTER CURATIVE
RESECTION IN STAGE II (UICC) HEPATOCELLULAR
CARCINOMA PATIENTS. M. Yamamom, S. Arii, T. Tobe, *K.
Sugahara & The Adjuvant Chemotherapy Study Group for Liver
Cancer in Japan. Department of Surgery, Kyoto University Faculty
of Medicine, Kyoto, Japan 606-01, *Yamanashi Medical University,
Yamanashi, Japan, 409-38
Adjuvant oral chemotherapy relevant to frequent recurrence was
studied in 67 patients with Stage II hepatocellular carcinoma
between May 1988 and Dexember 1990. Patients underwent
complete excision of the tumor in 26 institutions, composing the
Adjuvant Chemotherapy Study Group for Liver Cancer in Japan.
They were stratified into 2 groups according to preoperative liver
dysfunction: 55 in Clinical Stage (CS) I, mild dysfunction, and 12
in CS II, moderate dysfunction. Then, a randomized controlled
study of postoperative oral administration of 1-Hexylearbamoyl-5-
Fluorouracil (HCFU) was conducted in each group. As of Oct.
1994, recurrence in CS patients was observed in 20 (17 of local
recurrence and 3 of distant metastasis) of 27 (74%) in the control
group and 15 (12 and 3, respectively) of 28 (54%) in the treatment
group and in CS II patients, 3 of 5 in the control group and 7 of 7 in
the treatment group. Twenty five patients (83%) died after
recurrence, while 5 patients died without recurrence. The cumulative
survival and recurrence-free survival rates of CS patients in the
treatment group were higher (P=0.076, P=0.040, respectively) than
those in the control group. However, in CS II patients, No
significant difference was observed (P=0.774, P=I.000,
respectively). HCFU intake was suspended due to side effects in 9
of 21 (40%) CS and 3 of 6 (50%) CS II patients. Main side effects
were neuropathy in 5 and liver dysfunction in 5, although these
symptoms resolved within 2 months of suspension of HCFU. The
present study suggests that the potential benefits of HCFU on tumor
recurrence, either metaehronous multicentric occurrence or
metastasis, should be weighed against the risks of adverse reactions
in patients with mild liver dysfunction.
P191 P192
SURGICAL TREATMENT FOR CHOLANGIOCELLULAR
CARCINOMA
M.Yamamoto, K.Takasaki, M.TSUGITA, T.OTSUBO
Tokyo Women's Medical College, Departmentof Gastrointestinal
Surgery, Tokyo, Japan
We divided cholangiocellular carcinoma (CCC) into three types,
nodular, periductal, intraductal type, according to the gross
appearance and relation to the major intrahepatic bile duct.
Seventy-four resected cases of CCC were classified into three types.
This study did not include the hilar type of CCC. Nodular-type tumor
(n=32) clinically showed no symptoms (44%), or were associated
with chronic hepatitis (41%). Concerning the tumor spread pattern,
intrahepatic metastasis (34%) and portal vein tumor thrombus (34%)
were characteristic. Periductal-type tumor (n=32) showed jaundice
(53%), hepa'tolithiasis(16%), and lymph node metastasis (50%).
Intraductal-type tumor (n=10) showed abdominal pain (50%) and
fever (60%). The histopathological feature of this type was papillary
adenocarcinoma (100%). The incidence of intrahepatic metastasis
(10%) and lymph node metastasis (20%) was lower than that of
other tumor types.
Intraductal type had a good outcome, with a 5-year survival rate of
68% in 10 cases. In contrast, the survival rate of the nodular type was
27% in 32 cases, and for the periductal type was 3% in 32 cases.
This classification is of considerable value in understanding the
clinicopathological features and in surgical decision planning in CCC.
CLINICOPATHOLOGICAL FEATURES AND TREATMENTS OF
INTRAHEPATIC CHOLANGIOCELLULAR CARCINOMA
Yamashita K.. Une Y., Shimamura T., Misawa K., Hata T., Namieno T.,
Tomioka N., Nakajima Y., Sato N., Matsushita M., and Uchino J.
First Department of Surgery, Hokkaido University School of Medicine,
Sapporo, Japan
The rdiable treatment of intrahepatic cholangiocellular carcinoma (CCC)
has not yet been established. The previous papers have reported that its
outcome is overwhelmingly poor. In this study, we reviewed the
clinicopathological features of the CCC patients, and disoJssed the surgical
procedures leading to more favorable outcome. Thirty-two CCC patients
were enrolled in this study, who had been treated at our institute over the
last 21 years. The patients were composed of 27 males and 5 females with a
mean age of 59.4 years. Fourteen of the 32 patients underwent hepatectomy
(group H), and the other 18 palliative therapies (group P). According to
Couinaud's classification, hepatectomy, 2 segments or more, was performed
in 10 patients and one-segment resection in the other 4 in group H; however,
4 of them did not receive complete removal of the lesion (group H-I). The
mortality rate rdated to surgery was 14.3 % (2/14) and the other 12
survived surgery. In group P, chemotherapy, irradiation, or non treatment
was sdeeted for the patients. The postoperative mean survival period
(MSP) was 22.3 months (mos) in the 8 patients with complete removal of
the lesion, and 4 of them are now still alive with the longest living period of
34.5 mos. In contrast, the MSP in groups H-I and P was 8.0 and 7.7 mos,
respectively. Histology of the resected specimens revealed that the tumor
frequently involved the portal and hepatic veins (vascular invasion) and to
the intra- and/or extra-hepatic bile ducts (biliary invasion) even in group H.
The incidence of these invasions was 38.5 % and 76.9 %, respectively.
Lymph node metastasis was also microscopically frequent: its rate was
28.6 % (nodal invasion). The above three histologic findings presumably
reflect a part of biological features of the disease. The present results also
indicate that complete resection of the tumor should result in better outcome.
The extended resection of intra- and/or extra-hepatic bile ducts with
combined dissection of lymph nodes is .also required for improving the
prognosis of selected CCC patients, since the cancer cells are likely to
involve these tissues.
118
P193 P194
HEPATOCEILULAR CARCINOMA WITH EXq'ENSION ll,,rro TIIF
RIGHT ATRIUM:
report of successful liver resection by hepatic vascular
excl ttsion using cardiopulmonary bypass
Shiro Yogita t, Seiki Tashiro t, Masamitu tlarada , Yoh [:ukuda ,
Daisuke Wada,Tetuya Kitagawa,and Ituo Katoh
First Department of Surgery , Cardiovascular Surgery 2, School Of
Medicine, Tile University of Tokushima
We report successful liver resection using cardiopulrnonary bypass
with total hepatic ,asctdar exclusion ('I'HVEI for hepatocellular
carcinoma (HCC) with extension into the right atrium. A 61-year-
old with cirrhotic liver referred to department with 11('("
in the medial segment of the left lobe of the liverand tumorthrombus
extending into the right atrium. Liver resection performed
follows: (1) left and caudate Iobectomy performed leaving the
left lobe of the liver connected by only the left and middle hepatic
trunks; (2) the intracaval tumor thrombus and the left lobe of the
liver removed bloc; (3) cardiac arrest not performed
during THVE. The patient ha'd uneventftfl postoperative
except for slight renal dysfunction and discharged from the
hospital months followi ng surgery, l-le continued to do well until
months postoperati vely when multiple pulmonary metastases
discovered.
In conclusion,by performing dissection of the hepatic parenchyma
to the hepatic vein prior to removal of tumor thrombtts, the period of
extracorporeal circulation,duration of ischemic time to the liver
and intraoperative blood loss all reduced and radical operation
could be performed without scattering tumor cells during extirpation
of the ttunor.
DIAGNOSIS AND TREA OF HEPATIE
CAVERNOUS HEMANGIOMA
Liu Yongxiong, Cai Shouwan, Fang Yuquan
Hepatic cavernous hemangioma is the most
fequent ly occurring benign tumur of liver.
Altogether, 6 cases of cavernous hemangicma
of liver corffirmed by surgical operation were
treated in our department from 1990 through
Febarary of 994. The average diabeter of thses
hemangiomas was 9.7cm, with the maximal one 40cm.
Nine cases located at or involved section VI,
and 2 cases at audate lobe. The surgical
resection rate was 98.5, There was nei ther
death due to operation nor severe complications.
The diagnos s of hepa c cavernous hemangioma
and. its differential diagnosis from hepatic
crIncma, operation indications, selection of
operat ion method, intervent ion therapy, and
other problems are discussed in the paper.
P195 P196
USEFULNESS OF Ki-67 AND P-53 PROTEINS AS FACTORS FOR
PREDICTING PROGNOSIS OF HEPATOCELLULAR CARCINOMA
J. Yoshimoto. A. Ogawa, N. Yoshida, M. Itaya, T. Kitabatake, T. Iwata, H.
Stgou, K. Namekata, S. Watanabe, R. Nakanishi, K. Kojima, M. Fukasawa, T.
Beppu and S. Futagawa
The 2nd Department of Surgery, Juntendo University, Tokyo, Japan
[Purpose] Possibility of assigning Ki-67 and P-53 proteins as factors for
predicting prognosis was investigated. [Test subjects and Methods] Fifty-
six cancer tissues and 56 non-cancer tissues obtained by hepatectomy, and 16
liver cirrhosis tissues taken by liver biopsy were fixed in formalin, embedded in
paraffin, and after heating with microwave, using MIB-1 (Immunotech S.A) and
DO-7(DAKO) as primary antibodies, immunohistochemical staining was
conducted by ABC method. [Results] The Ki-67 labeling rate was an
average of 8.1%, showing a significantly high value compared with that of the
non-cancer region (p< 0.05). The average of the liver biopsy tissue in the liver
cirrhosis cases was 0.2%. Regarding the comparison of the AFP value and the
rate ofKi-67, cases ofmore than 20ng/ml ofAFPvalue showed significantly high
values compared with cases less than 20ng/ml(p< 0.05). In histopathological
factors and Ki-67 labeling rate, the intrahepatic metastatic cases were
significantly higher than those of negative cases (p<0.05). Further, in cases of
involvement to portal vessels, infiltration to the fibrous capsule and aneuploid
cases and in cases of low differentiation, a trend of high value was observed.
When the Ki-67 labeling rate was compared between those of the presence and
absence of preoperative aortic injection therapy, the cases received aortic
injection therapy showed a significantly low value compared with those of non-
treated cases (p<0.05). In comparison of the period between hepatectomy and
recurrence and the Ki-67 labeling rate, those recurred within a short time less than
12 months showed a significantly high value compared with those recurred after
12 months and later (p<0.05). The P-53 protein positive cases were observed in
19(36.5%) of52 cases. The Ki-67 labeling rate in the P-53 protein positive cases
was significantly higher than that of the negative cases (p<0.05). In the
comparison of histological differentiation degree and P-53 protein staining
positivity, the positivity of the low differentiated type was high compared with
those of the high and middle differentiated types. [Conclusion] (1) it was
found that for the evaluation of malignancy of hepatocellular carcinoma, a
possibility that the Ki-67 labeling rate is a useful indicater. (2) In cases that
abnormal appearance of P-53 protein cancer region, the proliferating activity is
augmented and the mutation of P-53 gene is related to the dedifferentiation of
hepatocellular carcinoma was suggested.
119
ENDOSCOPIC TREATMENT OF BLEEDING OESOPHAGEAL
VARICES (BEV): BAND LIGATION (EBL) Vs SCLEROTHERAPY
(ES) IN RELATION TO THE TRAINING OF OPERATORS.
Zangrandi F.; Gerunda G.E.; Neri D.; Bisello M.; Merenda R.; Meduri F.;
Barbazza F.; Da Giau G.; Bruttocao A.; Ciardo L.; Girardi R.; Renon L.;
Maffei Faccioli A.
Istituto di Chirurgia Generale ^, Policlinico Universitario, via Giustiniani
2 Padova.
Since the end of 70' ES of BEV achieved large diffusion, due to
effectiveness in control of both acute bleeding and recurrence. EBL
produces varices obliteration with less tissue damage. A smaller number of
complications is reported, in comparison with ES, mostly concerning
recurrence of bleeding from mucosal sloughs; final results will be available
only at the end of several present studies in the world.MATERIALS: we
report our experience concerning the first 30 patients who underwent
respectively ES and EBL. Both groups of PTS were homogeneous for
aetiology and Child-Pugh's risk score.
SCLEROTHERAPY BAND LIGATION p
Prophylaxis 7(24%) 12 (41%)
Eradication 18(60%) 8 (27%) n.s.
Not Valuable 2(6%) 8 (27%) n.s.
Sessions for eradic. 3.4+/-1.5 2+/-1.2 n.s.
Recurr. bleed. 14 (47%) 13 (44%) n.s.
< 30 days 3 (21%) 6 (46%) n.s.
> 30 days 11(79%) 7 (54%) n.s.
Recurr. varices (in eradicated) 7(39%) 4 (50%) n.s.
Death for bleeding 3(10%) 5 (16%) n.s.
All rebleedings were treated by ES, in both groups.
P197 P198
UKRAIN THERAPY OF HEPATIC METASTASES
AGAINST A BACKGROUND OF
MAGNETOTHERMIA.
S.Zemskov, O.Kravchenko, Ya.Susak, M.Zemskova,
Department of General Surgery, Department of
Oncology of the Ukranian State Medical University,
Kiev, Ukraine
The method of combined therapy with preparation
Ukrain and magnetothermia was offered in order to
improve the poor condition of the patients with
colorectal cancer metastases into liver. Ukrain
(NSC 63 1570) a semisynthetic derivative of
thiophosphoric acid and alkaloids extracted from
Chelidonium majus L. is both immunomodulant
a.d cancerostatic, especially in case of colon
adenocarcinoma. Magnetothermia (USSR patent
N4874617/14- 02797.1990 year) a UHF magnetic
field (27- 40 MHz) with prolonged electric
component was conducted by means of
apparatus "Magniterm" (firm "Medilak",
Kiev,Ukraine). There were 30 patients with
dissiminated colorectal cancer treated with Ukrain
against a background of magnetothermia in main
group and 25 patients with the same diagnosis
treated with 5-FU (NSC 19893) against a
background of magnetothermia in control group.
Magnetothermia caused increase of sensitivity of
metastatic cells to antiblastic preparations,
especially Ukrain, proved by growth of patients'
survival rate. Also important is achievement of 1-
year social rehabilitation of almost 50% of main
group patients.
SURGICAL TREATMENT OF HEPATOCELLULAR CARCINOMA:
PERSONAL EXPERIENCE
V. Ziparo, G. Lucandri, G. Balducci, G. Di Giacomo,
P. Mercantini, F. Stipa, S. Stipa
Ist Department of Surgery, University of Rome "La Sapienza",
Rome, Italy.
Up to December 1995, 70 patients with hepatocellular
carcinoma (HCC) have been observed at Department: 31
of them (44.3%)- did not receive any treatment, because of
poor liver function (Child class C) wide extension of the
tumour (multicentric liver disease, portal hepatic vein
thrombosis, extra-hepatic metastasis). Among the 39 patients
treated (55.7%), received chemoembolization (15.4%),
had his lesions alcoolized (2.6%) and 32 surgically
treated (82%, operability 45.7%). This last group included
24 males and females, with age of 48 years (range
6-67); all patients carried liver cirrhosis: criptogenetic
in (28%), post-hepatitic in 23 (HBsAg+ in 9, 28%, HCV+ in
14, 44%). 25 patients belonged to Child class A (78%) and
to Child class B (22%). At surgical operation, patients
didn't satisfied the operability criteria, because of wider
extension of the tumour its multicentric behaviour. So
only 28 patients have been resected (resectability 40%). We
performed 17 major hepatic resections (60.7%) and ii
atypical resections (39.3%), with hospital mortality rate
of 17% and 9% respectively. At the follow up (mean 52
months, range 4-129), 8/24 patients (33.3%) presented with
recurrent HCC (mean disease free time 13 months,
range 7-62). One and year actuarial survival rate is 68%
and 60%. Only patient received liver transplantation
after the surgical resection. We believe that liver
resection (wedge atypical in particular) should
represent the treatment of choice in Child A and B patients,
when HCC doesn't show multicentric behaviour
extrahepatic involvment. Chemoembolization alcoolization
should be reserved for patients with poor liver function
for treating relapsing disease.
120
POSTER PRESENTATIONS
Topic: PANCREAS
P199 P200
ENDOSCOPIC CYTOTECHNIQUES IN DIAGNOSIS OF
HEPATOBILIARY AND PANCREATIC MALIGNANCY
Abdel MoetiA., E1-GoharyA.M., Rashed M.Y.T., El-Said Hassan,
Hamza M., Ramadan M
Hepatobiliary Unit, Faculty ofMedicine, Alexandria University,
Alexandria, Egypt.
Malignant strictures ofthe extrahepatic bile ducts are difficult to
distinguish from benign strictures, particularly in patients with
primary sclerosing oholangitis. Because attempts at diagnosing
small cancers with fmo-needle aspiration biopsy are not possible in
the absence of an associated mass lesion, endoscopic cyto-
techniques including the brush cytology and exfoliative bile/or
pancreaticjuice cytology, have been used as a potential means of
establishing a specific diagnosis ofhcpatiobioliary and pancreatic
malignancies. Her we report our experience with endoscopic
cytotechniques performed on 30 patients. Twelve patients were
diagnosed .as bile duct carcinoma, 9 patients as pancreatic
carcinoma, 4 patients as papillary carcinoma and 5 patients were
diagnosed as hepatocellular carcinoma. Fine-needle aspiration
biopsy was positive in eleven out ofthirty patients with no false
positive results; its sensitivity was 37.7% and specificity was
100%. Brush cytology was positive in 13 out ofthe thirty patients
with no false positive results; its sensitivity was 43.3% and
specificity was 100%. Exfoliative bile and/or pancreatic juice
cytologywas positive in six out ofthe thirty patients with no false
positive results; its sensitivity was 20% and specificity was 100%.
Endoscopic cytoteclmiques are complementary techniques ofhigh
specificity for diagnosis of biliary and pancreatic malignancies.
They are recommended to be done in any suspicious case since
they add to the diagnostic yield ofconventional ERCP.
APACHE III AND CRP AS EARLY PREDICTIVE INDEXES
INACUTE PANCREATITIS.
C.Aggdopoulos,SAloizos,A.Touliatou,C.Papanastasopoulos,
B.Tsaglis,B.Papanastasopoulos
2rid Dept. Internal Medicine. General Hospital ofAthenes.Greece.
The aim ofthis study is to investigate APACHE III system end c-
reactive protein (CRP) as early predictive indexes in acute
panzreatitis (AP) end to compaire them. APACHE III system
concludes many variables but it may be calculated at tim day of
patient's admission. In ontrast CRP is one biochemical exam but it
is not emzrgent and the result of this zxam is not ready at
admission.We studied 53 patients (29 males and 24 females) with
AP last four years. Patients with (AP) arc divided in two groups:
group A concluded mild panoreatitis and group B complicated and
/or lethal pacreatitis. As complicated (AP) were (AP) with
intrmbolomlnal collections, nmpimtory infections, ARDS, acute
renal foulure.In all patients we calculated APACHE III system and
we measured CRP in their scroum by mephelometry at admission.
We used Mann Whitney test,sensitivity (se),speoificity (sp) and
Azouracy (Ao) for statistical analysis and we compaired APACt
III, CRP and Ranson system as early prediotive indzxes. The mean
age ofpatients was 62+/-13 years (31-89). Acfiology of (AP) was: 33
lithiasiz, 4 alcoholio,14 idiopathic, lipidemic and vasulitic
pancmatitis. Thzre wzre 5 lethal and complicated (AP) and 37
mild (AP). Complications were 7 inlnmbdolominal coileotions, 6
respiratory infections, 2 ARDS and acute renal failure. The mean
value of APACHE III in group A was 2211 and 41,913,5 in
group B (10,0000) and mean value of CRP 6451 and 220
mgr/di respeotively(/0,0000).If out off sign ofAPACHE III was
31 then Se 87,5%, S181%, and A 83% and if cut off sign of
CRP was>t20 mgr/dl then Se=812%, S183,7% and Ao 83%. If
Ranson system_3 then S50%, S189% and A078%.
In conclussion APACHE III and CRP have the same Se, Sp and Ao
in early prognosis of (AP). Their results are bett than Ranson
system. APACHE III is more complzx than CRP ,but CRP is not
available at first day ofadmission in many hospitals.
P201 P202
ACUTE EMPYEMA AND GAGRENE GALL-BLADDER CHOLECYSTITIS.
THEIR TREATMENT BY THE OPEN METHOD OR LAPARASCOPIC
A. Agorogiannis
Surgical Unit District Hospital General of Larisa-Greece
The aim of this study is to show in cases of empyema and gagrenous
cholecystitis, which of the two is the best method, the laparoscopic
one or the open surgical method. During the last 17 years we
operated at our Institution 4.200 cases with benign deseases of
the biliary tract. The 900 cases were acute cholecystitis and
among these the 450 cases were empyema or gagreneus cholecystits,
rupture of the gall-bladder and choloperitoneum. With increasing
experience the successful treatment of patients with acute
cholecystitis by laparoscopic cholecystomy was reported in the
international bibliography, as also our very limited personal
experience is similar, in the international bibliography the con-
version rate of lapascopic cholecystectomyto the open method, for
the treatment of empyema or gagrenous cholecystitis was 81, 3%.
The minimal time for laparoscopic cholecystectomy in acute
cholecystitis is 105 minutes. If we add .the 60 to 90 minutes which
are required after the conversion for the open method and if we
think that this happens so often (81, 3%), then we can understand
the advantages of the open surgical method for the treatment of
empyema and gagrenous cholecystitis. Also as we can see from the
international bibliography, most of the iatrogenic injuries are
done during the laparoscopic treatment of these deseases of the
gall-bladder and bile ducts.
Conclusions: Today with the help of CT scan and ultrasound scan, we
are able to diagnose exactly preoperatively the degree of serious-
ness of acute cholecystits and it is empyematous, gagrenous, per-
forated and when there is free choloperitoneum. In these cases
mostly it is impossible to perform cholecystectomylaparoscopicaly
and the conversion to the open surgical method it can be avoided if
the open method is used from the beginning.
COMPLICATIONS OF BILIARY PANCREATITIS IN THE LATE
OPERATED PATIENTS
O. Alabaz H. Demiry0rek,, I. Sungur, A. Aklnolu, S. Ozkan
Department of Surgery, University ofCukurova, Adana, Turkey
It is well known that a biliary cause for the preceeding acute pancreatitis was
found in 50 %. Recurrences may occur at 30-60 % of late operated patients.
In this study, 27 complicated patients who had biliary pancreatitis were
retrospectively reviewed during the January 1988 May 1995. Although
planned elective operations, these patients had recurrences or complications
There.were 16 (59 %) female and 11(41%) male, median age was 47.3.
They had recurrences at 30-60th days after the first pancreatic attack.
Seventeen had less than 3 Ranson criteria, 10 had more. By using
ultrasonoghraphic and tomoghraphic analysis, 6 patients were accepted as
hemorrhaged pancreatitis, 3 had peripancreatic collection and abscess and
the other 3 had pancreatic pseudocyst. Twenty three patients underwent
surgery and 9 ofthem were lost. One ofthe 4 patients who medically treated
were lost. Wound infection and acute respiratory distress syndrome were the
most frequent complications. It is concluded that in the late operated group,
recurrences were high and complications could effect the mortality rate.
121
P203 P204
CELLULAR MODEL FOR PANCREATIC CARBONIC
ANHYDRASE ACTIVITY AND BICARBONATE SECRETION
M, (. Aldrich, M. Zhang, T.D. Nguyen*, S.P. Lee*, R.L. Schleicher,
S. Fink.
Depts. of Surgery and Gastroenterology*, Atlantt VA Medical
Center and Emory University, Atlanta, GA, and Seattle VAMC and
Washington University*, Seattle, WA
We are seeking to develop an in vitro model of pancreatic
bicarbonate secretion using nontransformed dog pancreatic ductal
epithelial (DPDE) cells. These cells are vimentin negative, keratin
positive, f.ail to grow in soft agar, and are nontumorigenic in athymic
mice. Cells have been grown using 3 different culture techniques:
(1) as monolayers in petri dishes with a 1:1 dilution of MEM and
human gallbladder myofibroblast (HGMF)-conditioned media, (2) in
TranswellsTM using HGMF cells as the nutrient layer, and (3) as
organotypic cell cultures (floating monolayers attached to collagen
rafts). Cells have been successfully cultured over 15 passages.
Electrical resistance was measured with an epithelial voltohmmeter
and reached 1900+357 ohms/cm in TranswellTM cell cultures.
Carbonic anhydrase acti,ity was measured using an Imidazole-tris
micromethod (Brion et al., 1988), expressing activity as enzyme units
per mg protein, as listed below:
Human RBCs 24.6+2.4 DPDE cells:
Dog pancreas, whole 4.3+0.5 nonpolarized (petri dish) 0.4+0.2
Transwell, polarized 5.0+1.2
organotypic, polarized 5.7+2.9
Cells were also placed in Ussing chambers to assess bicarbonate
secretion. Buffered and unbuffered (bicarbonate-free) chloride salt
solutions bathed the basal and apical surfaces, respectively (pH 7.4).
Apical pH was measured over time using a pH meter in the apical
solution; the average rate of apical pH change was +0.01 units/min.
In summary, polarized DPDE cells maintain growth, carbonic
anhydrase activity, and ability to secrete bicarbonate after numerous
passages. These cells will facilitate study of cellular mechanisms
involved in regulation of bicarbonate secretion.
LONG-TERM RESULTS AFTER SEVERE ACUTE
PANCREATITIS
R. Andersson, A. BJrjesson, P. Leveau, I. Ihse
Department of Surgery, Lund University Hospital, Lund, Sweden
Severe acute pancreatitis is still associated with a high incidence of septic
complications and mortality associated with multiple organ failure. The
experience oflong-term results after severe acute pancreatitis is, however,
limited. During the period 1981-1990, 519 patients were treated due to
pancreatitis at the department of Surgery in Lund. 94 patients (18%) had
severe pancreatitis (necrotising pancreatitis, organ failure, treatment at the
ICU). The mean age was 57 (19-101) years. 67 patients were men and 27
women. Etiological factors included alcohol in 29, biliary tract disease in
33, trauma in 3 and endoscopic interventions in 2 patients, while the cause
was unknown in 27 patients. 80 patients (85%) had no previous history of
acute pancreatitis. Hospital mortality was 15% (14/94) occurring in median
22 (2-72) days after admission and related to multiple organ failure. The
80 patients surviving their acute pancreatitis were followed up till
September 1995.
In 37%, in median 2 (1-8) new attacks ofpancreatitis occurred: 26 patients
died after in median 5 years, most frequently due to cardiovascular disease.
Median follow-up for the 54 long-term survivors was 6 years and 10
months. Both pain (56%) and signs of exocrine pancreatic dysfunction
(41%) were frequent during the first year after the severe acute pancreatitis,
though the patients gradually improved and only 20% experienced pain and
17% had symptoms of exocrine dysfunction at the latest follow-up. 17 %
developed chronic pancreatitis and 33% became diabetic. Despite the quite
frequent pain and exocrine dysfunction initially after the severe acute
pancreatitis, most patients considered their general condition as quite good,
gradually improving and their working capacity was not substantially
affected. Thus, in most cases long-term results after severe acute
pancreatitis seem quite encouraging.
P205 P206
INJURY IN PANCREATIC ACINAR CELLS AFTER UNILA-
TERAL PULMONARY ISCHEMIA REPERFUSION PRO-
TECTION WITH TRIMETAZIDINE.
S.Anghelakopou!os, Ch. Papakonstantinou*,l. Prou-
salidis, G.Kiriazis**, C.Koletsos***, K.Kai-
doglou****, C.Katsohis.
Ist Prop. Surgical Dep., Cardiac Surgery Dep.*,
Laboratory of Pneum.** I.C.U. "G.Papanikolau"
Hospital***, Laboratory of Histology-Embryolo-
gy****. Aristotle University of Thessaloniki
(Greece ).
The aim of the present study was to investigate
the beneficial effects of Trimetazidine (is
an O.F.Rs.) in the pancreatic acinar cells
injured after experimental unilateral pulmonary
ischemia with perfusion in dogs. ghteen dogs
were submited to the ischemia reperfusion
of the left lung. The dogs were divided into
following groups Group A (6 dogs control)
and Group B (12 dogs) treated with Trimetazidi-
ne IV. Superoxide dismutase (SOD) levels were
evaluated in the portal vein blood before,
during the ischemia and 10min, 30min, 60min
after the restoration of the lung perfusion.
The specimens of pancreatic tissue were taken
for electronic microscopy study at the end
of the experiment. In Trimetazidine treated
group, a decrease of SOD values has been obser-
ved in correlation to the initial values in
the different stages of the experiment (p<O,01)
Also, in group B in the ultrastructural appea-
rence of the pancreatic acinar cells no remar-
kable deviation from the normal pattern was
noticed. In conclusion Trimetazidine seems
to play beneficial role in the injured pancreas
after pulmonary ischemia with short-term reper-
fusion in dogs.
PANCREATIC COMPLICATIONS AFTER
PANCREATODUODENECTOMY FOR RADICAL TREATMENT OF
ADENOCARClNOMA OF THE PAPILLA OF VATER
T. Bacchella, M.C.C. Machado, J.E. Monteiro da Cunha, E.E. Abdo,
S. Penteado e H.W. Pinotti
Departamento de Gastroenterologia (Cirurgia do Aparelho
Digestivo) Hospital das Clinicas da Faculdade de Medicina da
Universidade de Sio Paulo, Brasil
Pancreatic .complications (PC) are the most important .determinants of
early morbidity and mortality after pancreatoduodenectomy (PDT) for
radical treatment of adenocarcinoma of the papilla of Vater (adenoca
pV).
CASE MATERIAL: Fifty-six consecutive patients with adenoca pV were
treated with PDT and digestive tract reconstruction with two isolated je-
junal loops. There were 64.3% males and 35.7% females with a mean
age of 55.9+_1.5 years (range 30 to 75 years).
RESULTS: Major surgical complications occurred in 58.9% of the pa-
tients and were dialnosed as PC in .53.6% of them
PANCREATIC COMPLICATIONS 'N %
ACUTE PANCREATITIS 22 39.3
PANCREATIC FISTULA 14 25.0
BILIARY AND PANCREATIC FISTULAS 5 8.9
Table1
PC provoked intra-abdominal bleeding in 10.7% of the patients, perito-
neal abscesses in 8.9%, and GI bleeding in 5.4%. These complications
caused reoperations in 12.5% of the patients during the early postopera-
tive period.
The operative mortality rate was 8.9% and all deaths were linked to PC
(p<0.05).
The median survival time was 27 months (range 20 days to 129 months).
Tumor metastases occurred in 43.1% of the patients and were the main
cause of late death in this series. Actuarial survival rates of patients who
survived PDT were: 1-year, 91.5%; 3-ye.ars, 61.8%; and 5-years, 49.9%.
CONCLUSIONS: PDT'is the preferred operation for radical treatment of
patients with adenoca pV however usual PC induce major morbidity and
mortality in the early postoperative period.
122
P207 P208
THREE CASES OF CYSTIC TUMOURS IN THE PANCREAS
DIAGNOSED BY CYSTIC LIQUID INVESTIGATION.
JM Badia, LA Hidalgo, R Muns*, A Heredia, C Admella*, T So16",
JM Gubem.
Department of Surgery and Department of Pathology*.
Consorci Sanitari de Matar6. Matar6, Barcelona, Spain.
Preoperative diagnoses of cystic pancreatic tumours can be difficult.
Clinical and radiologic symptoms are often inadequate to discriminate
reliably among the different possibilities. Cystic fluid can be obtained
preoperatively by fine needle aspiration (FNA) guided by US or CT
scan. The investigation of cystic liquid for tumour markers, amylase
content and cytology can be used as preoperative diagnosis criteria.
Three cases of cystic tumours of the pancreas surgically treated in
whom cyst- fluid analysis was performed are presonted.
Case 1: A 62 year-old woman presented with an asymptomatic cystic
mass in the tail of the pancreas detected by US performed for an
urn'elated disease. Fluid analysis (CEA, CA 125, CA 19.9, amylase
content and cytology) suggested serous cystadenoma, that was
confirmed at operation.
Case 2: A 54 year-old woman was admitted for left back pain. US
and CT scan showed a cystic tumour of 90 mm located in the tail of
the pancreas. Cystic fluid investigation showed high levels of CEA, CA
125 and CA 19.9, low amylase content and epithelial cells at cytology.
The diagnosis of mucinous cystic neoplasm was confirmed at surgery.
Case 3: A 59 year-old man presented with epigastric pain and
obstructive jaundice. US and CT scan showed a large multiloculated
mass in the head of the pancreas with obstruction of distal- common
bile duct. Plasma tumour markers were negative. Fluid analysis
showed very high levels of CEA, CA 125 and CA 19.9, low amylase
content and suspicious epithelial cells at cytology. A probable
diagnosis of adenocarcinoma of the pancreas was made. The biopsy
of the cystic wall at surgery confirmed a ductal carcinoma with cystic
degeneration that was considered unresectable.
Comments: The preoperative analysis of the cystic fluid, obtained by
FNA, can provide useful information for the diagnosis of pancreatic
cystic tumours. At the time of operation, biopsy of the cyst wall and
frozen section study are fundamental to confirm the diagnosis and to
a decision whether resection or drainage is the treatment of choice.
PANCREATIC CANCER RESECTION IN THE ELDERLY
G,Balzan0, A.Zcrbi, V.Di Carlo.
Dep. of Surgery, S.Raffaele Hospital, Unik,ersity of Milan, Italy.
The purpose of the study was to investigate the rationale of a
potentially curative resection for pancreatic cancer in the elderly.
From 1989 to March 1995, 97 patients had a pancreatic resection
for ductal adenocarcinoma at our Institution. Among them, 30
(31%) were 70 years or older (mean: 73.6, range 70-82;
male/female ratio: 0.9); the remaining 67 patients (69%) were
younger than 70 (mean 57.9, range 31-69, male/female ratio: 2.1).
We compared retrospectively the operative outcome and the
postoperative survival of the two groups of patients.
Mortality was 6.6% (2 patients) in patients aged more than 70 and
1.5% (1 patient) in the younger group (p=not significant (ns));
major complications (requiring relaparotomy) were 20% and 8.9%
respectively (ns); minor complications were 16.6% and 26.8%
respectively (ns). The distribution acccording to UICC stages of
patients aged more than 70 and of younger patients was: stage I:
16.6% and 31.3% (ns); stage II: 0% and 3% (ns); stage III: 80%
and 58.2% (p<0.1); stage IV: 3.3% and 6%.(ns), respectively. The
rate of non radical resections was similar in the two groups (40%
and 43.2%). The incidence of poorly differentiated tumours (G3)
was slightly higher in the patients aged more than 70 (40% vs
26.8%; ns). Median postoperative survival was 13 months for
patients aged 70 years or older and 16 months for the younger
group; the difference between the survival curves was close to
significance (p=0.08). A multivariate analysis (Cox method) with
tumour stage, radicality and grading did not show an independent
effect of age on postoperative survival.
In conclusion, an accurate selection is needed to perform a
potentially curative resection in the patients with pancreatic cancer
aged more than 70. The mortality and morbidity rates are
acceptable; however, they are higher than in younger patients.
Furthermore, postoperative survival is slightly shorter in patients
who are 70 years of age or older: a possible cause could be a more
aggressive tumour behaviour in the elderly, as showed by tumour
stage and grading.
P209 P210
EPIDERMAL GROWTH FACTOR RECEPTOR CONTENT IN HUMAN
DIGESTIVE SYSTEM AND HEPATO-BILIARY PANCREATIC
NEOPLASIAS
G. Belli, V. D'Alessandro, A. D'Agostino, P. Ceccarelli,
Department of Surgery University Federico II, Naples, Italy
A. Di Carlo, A. Bloise, A. Mariano, V. Macchia
Department of Biology and Pathology Cellular and Molecular,
University Federico II, Naples, Italy
Epidermal growth factor receptors (EGF-R) have been identified in
many tumor cell lines and in human primary tumors and it has been
suggested that increased level of this receptor may represent an
important event in neoplastic transformation. However, so far there
are only a few reports concerning EGF-R content in digestive
system neoplasias and whether the measurement of EGF-R may
provide useful information on the malignancy of these tumors still
has not been established. In the present paper the binding of 125-1-
EGF to the plasma membranes of 100 samples of human digestive
system tumors was determined. These included 65 large bowel
adenocarcinomas, duodenum adenocarcinoma, 2 anus spino-
cellular carcinomas, 13 gastric carcinomas, 2 pancreatic
carcinomas, 4 gallbladder cancer, 4 primary hepatomas and 9 liver
metastases. Ninety eight samples of histologically normal tissue
excised surgically along with the tumors were used as controls. All
plasma membranes studied showed specific EGF binding.
However, only in a few cases tumor plasma membranes showed an
EGF receptor level higher than that of normal. The binding of 125
I-EGF was compared with clinical stages and grades of
differentiation. No correlation between tumoral stage and 125 I-
EGF binding was observed. The results obtained suggest that the
level of EGF-R may not be used as a biological indicator for a bad
prognosis or for anticipating a relapse of the tumor in human
digestive system neoplasias.
CLINICAL APPROACH TO ACUTEPANCREATITIS
R. Bianch i, M. Bergant no, I. Urban, R. Un o
Department of surgery,H.Varallo Sesia, Italy
From 1990 to 1995, was arrived our obsrvation 58 cases
of Acute Pancreatitis, with average of 63 years old
(20 DS). The 58% suffering from gallstone disease, in
terestng in 13% of Datients the main biliary tract'the
se every to was operated a distance of 10 days (7 DS)
from acute episode, whether with laDarotomy or videola
paroscopy associated with ERCP.Left patients, the 27%
interesting of alcolic and the 15% mixed disease (viral
infectious and malformative).
In according to Ranson" 13 patients was on the first
stage,12 on second, 14 on the third, 9 on fourth, 4 on
the fifth and sixth, at last, 2 on the seventh.
The surgery indication, with necrosectomy, draining
and washing the peritoneal cavity, was putting only in
2.5% of patients with mortality altoghether of 8% for
MOF and morbility of 16%" this, in our opinion, could
suggest that surgery is little necessary and the sur-
vivel and to be dependent on standardization of the
intensive care, with a commom index of reference that
furthermore permitting an histories case very homogene
ous between several centres.
123
P211 P212
LONG-TERM RESULTS OF SURGICAL DRAINAGE OF PANCREATIC
PSEUDOCYSTS
D. Boerma. T.M. van Gulik, L.T. de Wit, H. Obertop, D.J. Gouma
Dept. of Surgery, Academic Medical Center, Amsterdam, The Netherlands.
In the past few years, invasive but non-operative methods have challenged
surgical internal drainage as the standard therapy of chronic pancreatic
pseudocysts. Techniques as percutaneous drainage under radiological
guidance, endoscopic cystoenterostomy and endoscopic transpapillarystenting
have gained ground. In this report we evaluate the long-term results of
surgical, internal drainage of pancreatic pseudocysts in 43 patients with chronic
pancreatitis (CP) (m:f =, 32:11, mean age 45 yrs, range 24-76 yrs), operated
upon between 1984 and 1994. Prev/ous history: 15 pats (35%) had previously
undergone invasive therapy for a pseudocyst (7x cystoenterostomy, 3x
transpapillary stenting, 3x percutaneous drainage and 2 endoscopic drainage).
25 pats (81%) had a single pseudocyst and 8 pats had two or more. Median
size ofthe pseudocysts was 6.7 cm. (range 2-30 cm.). Surgery:
cystogastrostomy was performed in 28 pats, cystoduodenostomy in 8 pats and
cystojejunostomy in 7 pats. The indication for operation was abdominal pain in
32 pats, biliary obstruction in 2 pats, infection ofthe pseudocyst in 2 pats and
suspidon of malignancy, hemorrhage in the pseudocyst and intestinal
obstruction, each in pat. Postoperative complications occurred in 6 pats
(14%): ARDS, ileus, fever, wound-infection, 2 sepsis. Mortality was
zero. Follow-up was available in 38 patswith a mean follow-up time of 7.3 yrs.
3 pats (8%) developed a recurrent pseudocyst (one was drained
endoscopically, the othertwo were observed). 4 pats developed a pseudocyst
located elsewhere in the pancreas (1 underwent cystoenterostomy, no
intervention was required in the remaining three). 15 pats presented with
recurrent pain after a mean pedod of 4.4 yrs due to progression of CP. Other
complications of CP were: 3x CBD stricture, pancreatic abscess and
pancreatic cardnoma. 19 pats (50%) were pain-free after a mean follow-up
time of 7.0 yrs (range 1.7-10.7 yrs). Conclusion: Surgical intemal drainage is a
safe and effident procedure with in this study, a success rate of 92%.
Recurrent complaints in this group were believed to be due to progression of
CP, orto other complications of CP. Until the results of non-surgical methods
have been conclusively evaluated, classical surgical intemal drainage remains
standard therapy for chronic pancreatic pseudocysts.
DOES SEPSIS INFLUENCE THE OUTCOME OF PATIENTS
WlTlt SEVERE NECROTIZING PANCREATITIS?
PC B0rnman, K Michalowski, L Mitchell, JEJ Krige, M van Wyk,
Terblanche.
Department of Surgery, University of Cape Town Surgical,
Gastroenterology, Groote Schuur Hospital.
It is generally believed that sepsis is the most important factor causing
deterioration in necrotizing pancreatitis. To test this view this study was
undertaken to determine the incidence of sepsis, and to compare the
outcome between sterile and infected necrosis in deteriorating patients
undergoing surgery. Forty two consecutive patients admitted to ICU with
severe pancreatitis over a 3 year period (1992-95) were reviewed (33
males, 9 females; age 20-76 mean 44 years). Causes included: alcohol
27, gallstones 4, hyperlipidaemia, idiopathic 5, ERCP induced 1,
organophosphate 1. The severity of the disease was classified according
to the APACHE II score and extent of the pancreatic necrosis was
assessed by CT scan or during laparotomy. Surgery was pertbrmed only
in patients who deteriorated in spite of intensive supportive therapy. The
diagnosis.of sepsis was established by US or CT guided aspiration or at
laparotomy. Twenty one patients (50%) underwent laparotomy
(necrosectomy 19, decompression 2). Overall 18 patients (42%) died; 4
of 21 (19%) who were treated conservatively and 14 of 21(66%) who had
surgery. Seventeen of the 19 positive cultures grew gram negative
aerobes. In the conservative group (21 patients) sepsis could not be
documented in the 4 deaths while all 7 patients in whom sepsis was
confirmed, survived. Three of the 9 patients operated on within the 1st
week and 8 of 12 patients operated after week had documented sepsis.
The surgical mortality was 6 of 11 (APACHE score: mean 15; range 8-
27) and 8 out of 10 (APACHE score: mean 16; range 11-25) with and
without sepsis respectively. Sepsis does not appear to be the principal
factor causing deterioration in necrotizing pancreatitis nor is it associated
with a worse prognosis than sterile necrosis when surgery is performed
in this situation.
P213 P214
OCCLUSIVE NETHOD IN PANCREATIC SURGERY.
I.Buriev,V.Koubyshkin,H.Zaidenberg,T.Savvina
A.V.Vishnevsky Institute of Surgery, Hoscow
Russia.
An experience of 207 occlusions of pancreatic
duct,cysts,fistulae and parapancreatic collec
tions in 116 patients with inflammatory and
91 with tumorouse diseases of pancreas is pre
sented. For occlusion, in 47 patients a non-
absorbabel silicon material "Elastocil" was
used and in 160 absorbabel "RABRON". Both are
radiocontrast media with antibacterial additi
ons. For 95 patients with pancreatic fistulae
and collections occlusion was perfomed through
the drainage tube without laparotomy,no com-
plications or deaths were observed.ll patient
had recurrence and cure wos aghieved in 77,9%
of cases.For 11 patients with pancreatic cyst
percutaneous needle aspiration and occlusion
were performed without mortality: cure in
81,1 of cases,recurrence in patient.During
proximal pancreatectomy occlusion was used at
the reconstructive stage in 101 patients to
occlude the ducts of the pancreatic stump(52)
or "occlusive pancreatojojenostomy" (49).This
way,the incidence of complications reduced to
27,7.ortality rate was 11,8.Postoperative-
ly "Sandostatin" was used. Analysis of long-
term results on follow-up for up to 5 years
showed that,following occlusion patients did
not develop functional disorders.Also progres
sion or development of diabetis melitus were
not observed.
Occlusive method is effective for the treat-
ment of fistulae, cysts (<5 cm) with formed
walls. The use of occlusion during partial
pancreatectomy reduces the risk of leakage.
PYLORUS PRESERVING PANCREATODUODENECTOMY FOR
MALIGNANT DISEASE OF THE PANCREAS
G. Capriata, R. Annibali, V. Basilico, R. Bellotti, P.Ceriani, D. Clerici, F.
Giacd, B. Griffa, F. Sacchi
Department of Surgery I, Valduce Hospital Corn0
According to recent surveys pancreatic cardnoma (P.C.) is the 4th. leading
cause of death for malignant tumours. Whipple procedure includes a gastric
resection, more to avoid anastomotic ulcer than to obtain a more complete
radicality. However it is responsible for some digestive problems. Pylorus
preserving pancreato-duodenectomy (P.P.P.D.) has been originally
introduced for chronic pancreatitis. (1) Preservation of approximately cm
of the duodenum allows a physiologic regulation of gastric acidity. (2)
Preservation of pyloric funcion grants: normal gastric acid secretion, normal
gastric reservoir function, regular gastric emptying, reduction of alkaline
biliary reflux, reduction of operative time. Since January 1993 we have
performed 8 P.P.P.D. (7 for adenocarcinoma and for cysto-adenoma
borderline). We have reported no mortality; a pancreatic fistula has
resolved spontaneously on the 20th. P.O. day. Nsogastric tube has been
left for a mean time of 7,8 days (range: 4-12). In our experience, gastric
emptying has proven to be effective, without the need to associate a
pyloroplasty to standard P.P.P.D. Four patients have subsequently died for
progressive disease (mean survival time 8 months). Follow-up in the
remaining4 patients rangesbetween6-17 months. Endoscopy performed at 6
months has revealed no anastomotic recurrences and benign jejunal ulcer.
Accordingly, we believe that P.P.P.D. is an effective procedure to lower
the sequelae of gastric resection in the surgical treatment of P.C. It
improves the quality of life, which is essential for pattens with such a
poor expectancy.
REFERENCES
(1) Traverso L. W., Longmire W.P. Preservation of the pylorus in
pancreaticoduodenectomy. Ann. Surg. 1980; 192: 306-310.
(2) Graces P.A., Pitt H.A. and Longmire W.P. Pancreato duodenectomy
with pylorus preservation for adenocarcinoma of the head of the pancreas.
Br. J Surg. 1986; 7
124
P215 P216
EXTRINSIC COAGULATION PATttWAY ACTIVATION
AND EXCESS THROMBIN GENERATION
IN PANCREATIC CANCER
V Chinswangwatanakul, A.K. Kakkar, N. DeRuvo, S. Tebbutt,
R.C.N. Williamson
Department of Surgery, Royal Postgraduate Medical School,
London, U.K.
Ductal carcinoma of the pancreas is one of the most aggressive
solid tumours and is associated with a strong tendency to
thromboembolic complicati6ns. We have studied circulating levels
of five coagulation factors in 14 patients with pancreatic carcinoma
and 14 age matched control subjects without cancer. Plasma levels
of tissue factor (TF), factor Vlla (FVIIa), factor Xlla (FXIIa),
thrombin-antithrombin complex (TAT) and prothrombin fragment
1+2 (PFI+2) were measured using ELISA techniques. TF was
three times higher in pancreatic cancer patients (median 788 vs 204
pg/ml, p= 0.04), FVIIa was 56% higher (97.5 vs 62.5 mu/ml, p=
0.01), TAT was seven times higher (14.1 vs 1.7 ,g/l, p- 0.0001)
and PFI+2 was 68% higher (3.7 vs 2.2 nmol/i, p= 0.01). This
indicates an activation of the extrinsic pathway and excess thrombin
generation in pancreatic cancer and suggests that this procoagulant
may play a pivotal role in the thromboembolic complications
associated with this disease.
SYNTHESIS AND SECRETION OF ATRIAL NATRIURETIC PEPTIDE
IN HUMAN PANCREAS
Baik-Hwan Cho*, Jong Hun Kim*, Suhn Hee Kim,
Sung Zoo Kim and Kyung Woo Cho
Department of Surgery* and Physiology ,Chonbuk National University
Medical School, Chonju 560-180, Republic of Korea
The natriuretic peptides system is involved in control of body fluid volume
and smooth muscle contraction as endocine and paracrine manner. We
have reported the presence of atrial natriuretic peptide (ANP) in gall
bladder and bile in several species animal including human. To elucidate
whether human pancreatic juice has ANP, the juice samples were collected
from the pancreaticoduodenectomized patients via polyethylene tube. The
tubes were inserted into the main pancreatic duct through percutaneous
transjejunal route during reconstruction procedure. The presence and
characterization of ANP in human pancreatic juice were screened using
radioimmuno-assay with ANP specific polyclonal antibidy and high
performance liquid chromatography. Serial dilution curves of extracts of
pancreatic juice were paralleled to the standard curve of atriopeptin II1.
The concentration of ANP was 4.46+1.48 pg/ml in pancreatic juice (n=6)
(20-40 pg/ml in human blood). The total amount of ANP in pancreatic
juice for 24 hrs was 604.5+192.7 pg/24 hrs (n=6). The gel filtration and
HPLC profile of ANP showed major peak corresponding to low molecular
weight of ANP that indicate the ANP is active form. Reverse transcriptase-
polymerase chain reaction (RT-PCR) was employed for detection of ANP
mRNA from pancreatic tissue. Predicted size and sequence of RT-PCR
products revealed that there was a ANP synthesis in pancrease. To
localize which cell synthesize ANP, immunohistochemistry for the detection
of ANP granules is under study. These results suggest that human
pancrease synthesize and secrete the ANP via exocrine system and this
ANP may act on di0estive system as a paracrine manner.
P217 P218
IS PROPHYLACTIC GASTROENTEROSTOMY A SAFE AND
EFFECTIVE PROCEDURE IN ADDITION TO BILIARY BYPASS IN
UNRESECTABLE PANCREATIC HEAD CARCINOMA?
P.P.L.O. Coene B.A. van Wagensveld, T.M. van Gulik, D.J. Gouma
Dept of Surgery, Academic Medical Center, Amsterdam, The Netherlands
Recent studies suggest that a prophylactic gastroenterostomy in addition
to palliative biliary bypass for unresectable pancreatic carcinoma
increases morbidity and mortality without offering any long term benefit. In
this study the efficacy of an additional gastroenterostomy was evaluated in
120 consecutive patients with incurable pancreatic head carcinoma
(male/female ratio 1.07, mean age 63 years, range 39-90 years),
performed in combination with a biliary bypass in 118 patients. Indication
for surgical palliation was local unresectabilty in 83, liver metastases in 30,
and peritonitis carcinomatosa in 7 patients. Gastroenterostomy was
performed prophylactically in 92 patients (PGE,) or for symptoms in 28
patients (SGE). There wasno difference in tumor size or dissemination in
patients with PGE and SGE.
Results: Overall postoperative morbidity was 28% (operation related
complications 18% and general complications 10%). Delayed gastdc
emptying (DGE, defined as nasogastdc intubation >7days) occurred in 18
patients (15%). Incidence of DGE was comparable among patients after
PGE and SGE (12% vs 18%, resp., ns). 30-day mortality was 0.8%,
hospital mortality was 2.5%. Median hospital stay was 17.5 days. Overall
postoperative median survival was 28 weeks (32 weeks for locally
unresectable tumors vs 14 weeks for metastasized tumors, p<0.05).
Survival was significantly longer in patients after PGE (28.5 weeks)
compared to SGE (19 weeks, p<0.05).
Late gastric outlet obstruction (GO0) developed in 12 patients (10%) after
a median interval of 28 weeks. 7 patients had a previous PGE (8% of all
PGE) and 5 patients previous SGE (18% of all SGE). Onset of late GOO
was 27weeks (median) and 11weeks in the patients with previous PGE
and SGE, resp. There was no relation with tumor size or tumor
dissemination. Of the 12 patients with late GOO, 5 patients had diffuse
intra-abdominal tumor spread with segmental bowel obstruction found at
re-operation (postoperative survival mdn 3 weeks), whereas 7 patients
were considered preterminal and were not operated.
Conclusion: Prophylactic gastroenterostomy in addition to biliary bypass is
associated with low morbidity and mortality, and proved effective in
preventing gastdc outlet obstruction. The occurrence of late obstructive
symptoms after gastroenterostomy was found to be a terminal event.
125
PANCREATODUODENAL RESECTION AS A SECONDARY RECONSTRUCTIVE
OPERATION
M.V.Danilov, I.M.Buriev, _V.P.Glabay, A.G.Mylnikov
A.V.Vishnevsky Institute of Surgery, Moscow, Russia
Between 1975 and 1995, 232 patients subjected to pancreatoduodenal
section (PDR) for pancreatic and periampullary tumors (204) and chronic
pancreatitis (28). Previously 131 of these patients have had surgical interventions
including percutaneous (57) and transpapillary (8) biliary decompression,
biliodigestive bypass external drainage of bile duct (53); operations
have been previously performed stomach and pancreas. During PDR the
most favorable conditions for bile tract management after transhepatic
cholangiostomy, in papillary tumors also after endoscopic papillosphincterotomy.
The optimal method of biliodigestive bypass before PDR considered the
terminolateral hepaticojejunostomy. If PDR performed after
gastroenterostomy Bilroth-II distal gastrectomy, earlier created anastomoses
preserved usually with supplement of truncal vagotomy. In patients with
chronic pancreatitis who have been previously undergone the longitudinal
pancreatojejunostomy its preservation the best solution during the PDR.
P219 P220
"VENTRAL OPEN PACKING" IN TREATMENT OF INFECTED
PANCREATIC NECROSIS
M.V.Danilov, V.P.Glabay, A.G.Mylnikov, R.a.Temirsultanov
Department of Surgery, Moscow Medical Academy, Moscow,
Russia
In period from 1991 to 1995 166 patients with acute necrotising pancreatitis
(ANP) observed. Infected pancreatic necrosis in 55 patients in
terms of 3-20 days from the onset of the disease. The general signs of this
complication palpable in the upper abdomen (in 69,1% pts), fever (in
all) and presence of intra- and/or peripancreatic cavities and fluid collections in
US and CT examinations (in all). The diagnosis of infectious complications of
pancreatitis considered the strict indication to surgery. In 24 patients with
acute infected pancreatic pseudocysts and abscesses transcutaneous drainage
applicated in (failured in 2); open external drainage of suppurative cavity with
subsequent lavage performed in 16 (4 died). In 31 patients with the wide-
spread infected pancreatic and peripancreatic tissues necrosis ventral open pac-
king used. Abdominal cavity opened by wide bilateral subcostal incision,
the gastrocolicum ligament cut and right and left colonic flexures fallen
down, and the posterior surface of the pancreas mobilised. Thereby removing
of pus and debridement of non-fixing devitalised tissues easy achieved. The
closely fixed removed because of bleeding hazard. The
peripancreatic and retrocolic spaces packed with wads of gauze, abdominal
wall closed by temporary sutures. The wads replacements performed
every two days amounting from to 18 times. The overall mortality rate
35,8% (11 patients) including with alcohol-induced ANP and with biliary ANP,
the of death in all due to sepsis. In conclusion, believe that
the ventral open packing appears to be the operation of choice in treatment of
wide-spread infected pancreatic and peripancreatic necrosis.
MANAGEMENT OF PANCREATIC TRAUMA
K. Daskalakis, T. Mavromatis, G. Diamantopoulos..
3rd Surgical Department, "EVANGELISMOS" Hospital, Athens,
Hellas.
The aim of this study is to present the problems in diagnosis
and management of pancreatic trauma. During the last decade
(1985-1995), 7 patients with pancreatic trauma (4 males, 3
females) were treated in the 3rd Surgical Department of
"EVANGELISMOS" Hospital. The causes of pancreatic trauma
were: traffic accident in 4, gun shot in 2 and stub wound in
patient.
All patients with pancreatic trauma, had multiple injuries in other
intraabdominal organs, or/and in the chest, or/and in the bones.
All patients submitted surgery.
During operation, all the injuries of intraabdominal organs were
managed at the same time. Especially, pancreatic trauma was
managed as follow: suture of the wound of the pancreas and
drainage in 1, distal pancreatectomy in 1, anastomosis of the
distal pancreas using Roux-en-Y jejunal loop in 2 and drainage
of the pancreatic area in 3 patients.
Three patients reoperated on 3, 2 and times each after the
initial operation because of complications. Early postoperative
complications were presented in 6 patients: one intraabdominal
abscess, 3 pancreatic abscesses, 2 post-traumatic necrotizing
pancreatitis and 4 pancreatic fistulas. Pancreatic fistulas were
managed conservatively and the other complications by surgery.
There were no perioperative deaths in this series.
In conclusion, the pancreatic trauma has a high gravity especially
if associated with multiple intraabdominal injuries. The final
diagnosis is established intraoperatively. The surgical interventions
must be adapted to the associated visceral injuries. The best
results in treatment are obtained when the correct and definitive
treatment is carried out at the first possible opportunity.
P221 P222
ACUTE PANCREATITIS. TREATMENT WITH 5 FLOURACIL.
G. de la Llera, M. Larrea, M. Garcia.
Department of Surgery. Faculty Hosp. "Gral
Calixto Garcia", La Habana, Cuba.
Acute pancreatitis (AP) sometimes turns to
severe forms with a high mortality rate.
It has been reported that 5 flouracil (5 FL)
blocks the synthesis of the pancreatic enzymes
avoiding the progression of the illness. This
was the reason of this study.
In our Hospital were admited 27 patients
during 9 months with the diagnosis of .PA that
was confirmed with a compatible history and
physical examination data, indicators of a
nonspecific inflammatory response, laboratory
finding of elevated serum amylase or serum
lipase level and an enlarged pancreas with or
without peripancreatic fluid collection
documented by computed tomography (CT) or
ultrasonography (US).
The patients were classified in two groups
according to the Ranson's prognostic indicator
and Balthazar's CT grades: "High risk" group
and "Low risk" group.
The treatment was 5 FL in an IV bolus of 250
mgs a day during 1 to 3 days.
In a].l patients with 5 FL treatment the
abdominal pain was noticed to disappear
sudenly and the time of recovery was the same
for the high risk group and the low risk group.
Only one patient died and was a non 5 FL
treated because of an early surgical operation.
In conclusion the 5 one more weapon in the
AP treatment.
DELAYED GASTRIC EMPTYING OF SOLID FOOD IN PANCREATIC
CANCER
A.F. El-Attar, H.W. Merrick, J.M. Howard
Department of Surgery, Medical College of Ohio, Toledo,
Ohio, USA
The study was designed to monitor gastric emptying as
related to operation on patients with pancreatic cancer,
with comparison of delayed gastric emptying (DGE} before
and after operation to define its incidence and whether it
was caused by the tumor or by operation. Gastric emptying
studies (GES), usually on the fifth postoperative day,
were performed in 32 patients with caer of the exocrlne
pancreas (group A) using technetium Tc sulphur collold
mixed with scrambled eggs. Postoperative gastric emptying
was prolonged in 64%, compared to 33% before operation.
Eleven patients underwent resection and 17 were explored
but not resected. Resection of the pancreatic head was
performed in 10 patients. In the unresected group gastric
bypass, with or without billary bypass, was performed in
11. Postoperative DGE occurred in 40% after resection in
contrast to 73% undergoing gastric bypass. Patients with
advanced disease had an overall incidence of postoperative
DGE of 77% compared to 40% with resection. Intraoperative
radiotherapy did not contribute to DGE; postoperative DGE
resultlng in 40% compared to 38% without therapy. Control
groups Included (B} patients with other malignancies in
the area of the pancreatic head, (C} patients with benign
abdominal diseases, (D} a normal population (literature).
In group B postoperative DGE occurred in 46%;
preoperatively 38%. The overall incidence of DGE in Group
B was 25% with resectable cancers in contrast to 60% in
advanced disease. In group C postoperative DGE was 20%;
10% preoperatively. We conclude that DGE is a frequent
complicatlon in patients with pancreatic cancer; more
frequent with advanced disease includlng patients
undergoing gastric bypass. DGE was more frequent
following procedures that left the cancer unresected, even
though the operative time and trauma were reduced. This
suggests that DGE may be, , a blologlcal result of
the pancreatic cancer, per se. Prophylactic gastric bypass
may be inadvisable.
126
P223 P224
PANCREATIC DISEASES AND SPan-1 SERUM TUMOR
MARKER
A. Frena
2nd
Department of General Surgery, Regional Hospital of Bolzano,
Bolzano, Italy
The antigenic determinant recognized by monoclonal antibody SPan-1 is
greatly elevated in sera of pati+nts with exocrine pancreatic cancer but not
in sera of normal individuals. A soluble form of SPan-1 is shed into the
blood of inflammatory disorders of the pancreas. Experimental studies
demonstrate that in malignant tumors of stomach and colon the blood level
of SPan-1 correlates closely with disease progression. This suggest that
SPan, could be an important tumor marker clinically. As preliminary
studies showed soluble SPan-1 to be increased in the blood of patients
with pancreatic cancer, we tried to answer the question whether SPan-1
could be a useful tumor marker in exocrine pancreatic carcinoma.
Between October 1994 and September 1995 we determined the levels of
SPan-1 in sera of 30 patients with ductal carcinoma of the pancreas, 15
patients with chronic pancreatitis and 20 healthy controls. The test for
SPan consisted of a SPan-l-Riabead research kit (Dainabot Co., Tokyo,
Japan) with a cutoff level of 30 U/ml.
The results were as follows:
Mean Value (U/ml) Standard
Deviation
HEALTHY CONTROL GROUP 8.5
CHRONIC PANCREATITIS 22.0
PANCREATIC CANCER 216.9
2.7
7.9
298.1
There is a significant difference in SPan-1 blood plasma level between
patients with chronic pancreatitis and patients with pancreatic carcinoma
in comparison with the control group (p=0.001). Sensitivity and specificity
of SPan-1 for pancreatic cancer in this population are 60.2% and 65.0%
respectively (64.8% and 52.5% for an established marker as CA 19.9).
SPan-1 can be another useful serum marker in detecting pancreatic cancer.
EARLY SURGICAL TREATMENT OF PANCREATIC TRAUMA
A. Frena, A. Mazziotti ,G. Gozzetti
2nd
Department ofGeneral Surgery, Regional Hospital of Bolzano, Bolzano, Italy
2nd
Department of General Surgery, University of Bologna, Bologna, Italy
The frequency of pancreatic traumas is increasing costantly: they account for 4-9%
of abdominal traumas undergoing surgery. These traumas are frequently very
difficult to early diagnose.
Our experience is based on 21 pancreatic traumas, observed between 1984-1994
(4,7% of the abdominal traumas undergoing surgery). Pancreatic organ injury is
divided into five grades on the basis of the AAST 1990 Classification.
D8 IX Ill IV V
IN. ofpts. 1013 4
% 147,6114,3 119,01 14,314,8
The diagnostic accuracy of our clinical, biochemical and radiological
investigations in )ancreatic traumas are summarised below:
ERCP CTscan AMYLASE X-rays US
Pts. 2 12 21 7 11
Pts.POSITIVE 2 9 12 4
% 100 75 57 43 36
The results of o ement were as follows:
Grade Pts Associat. Died Surgical treatment Complications due
Injuries to late treatment
10 7 7 hemostasis, drainage pseudocyst
II hemost., drain., debridem. acute pancreatitis
III 4 2 distal pancreatectomy
IV 3 2 pancreatoduodenectomy
V pancreatoduodenectomy
The main problem with posttraumatic pancreatic lesions is linked to early
diagnosis. The consequences of intraoperative diagnostic errors may lead to
serious complications (chemical peritonitis from pancreatic juices, external
pancreatic fistula, acute necrotizing pancreatitis), almost always with an
unfavorable prognosis. The difficulties involved in diagnosing these lesions can be
attributed to three eszsenzial factors: 1. the frequent occurrence of multiple
lesions; 2. the need for a second operation, due to lesions that were missed during
the first operation or to surgical measures that were inadequate for the lesions
encountered; 3. the considerable time that in many cases elapses between the first
observation and diagnosis.
P225 P226
LAPAROSCOPIC AND RETROPERITONEOSCOPIC
NECROSECTOMY FOR INFECTED NECROTIZING
PANCREATITIS. M. Gagner, MD, A. Pomp, MD, Department
of General Surgery, The Cleveland Clinic Foundation,
Cleveland; Ohio and HoteI-Dieu de Montreal, Montreal,
Quebec.
Over a 2 year period, 11 patients underwent laparoscopic or
retroperitoneoscopic pancreatic necrosectomy for infected
necrotizing pancreatitis. Three techniques have been
employed: a retrogastric/retrocolic approach in 50% of the
patients, a transgastric approach in 37% and a
retroperitoneoscopic in 15%. Using 3 to 5 trocars of 10 mm,
a window is created along the right pericolic gutter and a
right angle instrument perforates the gutter to aspirate the
pus and perform a debridement. Similarly, the gastrocolic
gutter ligament is entered, debrieded and large axiom sump
drains are positioned in the necrotic areas under laparoscopic
guidance. The transgastric route is used with endoluminal
trocars to perform a linear posterior gastrotomy. Through the
gastrotomy the necrotic material is debrided, cleaned and
aspirated in the body/tail of the pancreas. The
retroperitoneoscopy is used in the flank, extraperitoneally
under direct vision for trocar insertion. The debridement and
necrosectomy are performed from the anterior surface of the
psoas up to the body and tail of the pancreas. A drain is
positioned also under laparoscopic guidance. The mean age
was 54 (41-69) with a mean hospital stay of 51 days. Six
patients had respiratory failure, 6 had gram negative
septicemia and 2 had an open reintervention. There was 2
mortalities from multiple organ failure. These techniques
achieve the same objective as an open procedure but may be
less invasive and may result in a decreased stress response
post operatively.
PANCREATIC AND PERIPANCREATIC SUPPURATIONS
G.Ghidirim. E.Gutu, I.Mahovici, I.Gagauz, D.Ciurea
Department ofSurgery, Medical University ofChisinau, Republic ofMoldova
Sixty-seventy percent of the severe forms of pancreatic necrosis have
various complications. Suppuration is one of the most severe complica-
tion, due to its high incidence and mortality. The vast majority of the
purulent complications occur in the third phase ofthe pancreatic necrosis
(liquefaction and sequestration) and only a few of them occur in the
second phase (necrosis). Suppurative complications of pancreatic
necrosis can be classified into three different categories: focal, diffuse
and remote suppurations. This classification is for medical decision's
(diagnosis, treatement) great benefit. There have been 727 patients with
complicated pancreatitis kept under our observation. Amongthem, 270
had purulent complications: 13 with intraparenchymal abscesses, 163
with purulent necrotic peripancreatitis, 45 with purulent diffuse peri-
tonitis, 30 with purulent necrotic omentitis and 19 with purulent lesser
omental bursitis. Although only 39% ofthepatients were over 60 years of
age, half(48.9%) ofthe purulent complications have been found among
them, which emphasizes the negative influence of immune and vascular
factors in the pathogenesis of the suppurative complications of
pancreatic necrosis. Besides, the necropsy revealed a lot of remote
purulent complications: purulentpylephlebitis with multiple abscesses
ofthe liver (8 cases), abscesses ofthelungs (8 cases), purulentpleuritis (7
cases), purulent focal miocarditis (4 cases) and purulent nephritis and
perinephritis (6 cases). Ten patients had lethal septicopyemia. In order to
reduce the incidence of such complications, the surgical tratement ofpan-
creatic andperipancreatic suppurations needs to be carefully selected,
based on specific criteria, ifidividualisated and back up by a complex
drug terapy, including direct blood transfusions.
127
P227 P228
LYMPH NODE METASTASES IN PANCREATIC DUCTAL
ADENOCARCINOMAS: CORRELATION WITH TUMOUR
SUPPRESSOR GENE P53 ALTERATIONS
PC. Giulian0tti, D. Campani*, D. Cecchetti*, F. Ceccarelli*, G. Fomaclari*,
T. Balestracci, A. Costa, D. Giardino, E. Rossetti, F. Mosca
Institute of (3eneral and Experimental Surgery, *Department of Pathology
University of Pisa, Pisa Italy
No definitive data between p53 over-expression and tumour grade, stage or patient
survival status has been demonstrated so far in pancreatic adenocarcinoma.
Nodal metastases are correlated with a worst prognosis. Micrometastatic nodes
may be sometimes overlooked and the real tumour stage underscored.
Aim of this study was to investigated the possible correlation between nodal
involvement and p53 over-expression in pancreatic cancer.
We determined cellular expression of the p53 tumour suppressor gene and
proliferating activity in 48 pancreatic adenocarcinomas collected from 1971 to
1993.
The study was carried out by immunocytochemistry after microwave irradiation of
formalin-fixed, paraffin embedded tissue. Immunocytochemical analysis was done
using D07 (Dako) and MIB1 (Immunotech) monoclonal antibodies with the biotin-
avidin (ABC) method to asses p53 protein and proliferative activity, respectively.
The results were expressed in % value of labelled cells and correlated with pTN
status and Kltppel grade of the tumours.
Twenty-five tumours (52%) had p53 over-expression; 14 (29%) showed high
growth fraction; 9 (23%) were T1, 27 (69%) were T2, 3 (8%) were T3, 20 cases
(48%) had positive nodes; 16 (33%) tumours were (31, 20 (42%) were (32 and 12
(25%) were G3.
The statistical analysis showed 16/22 (73%) NO p53- tumours versus 14/20 (70%)
N1 p53+ tumours demonstrating a.positive correlation between the presence of
lymph node metastases and p53 over expression (p=0.005).
In addition, according to the histological grade p53 immunoreactivity was present
in 7/16 (44%) G1, 10/20 (50%) G2 and 8/12 (67%) (33 tumours. The statistical
analysis did not demonstrate a significant correlation between tumour grade and
p53 over expression although the p53 expression increases from E;1 to (33.
No relation was present between clinical-pathological parameters, p53 expression
and proliferating activity assessed by MIB antibody.
In conclusion our preliminary results suggest that p53 over-expression in
pancreatic cancer may have a prognostic value indicating the probability of nodal
involvement.
PANCREATIC CANCER RECURRENCE
PC. Giulianotti, G. Caravaglios, T. Balestracci, C. Marini*, E. Federiconi*,
F. Falaschi*, S. Cecconi, F. Sbrana, U. Boggi, F. Mosca
Institute of General and Experimental Surgery, *Department of Radiology,
University of Pisa, Pisa Italy
The aim of the study to analyse the kind of pancreatic cancer recurrence in relation to the
accuracy of diagnostic procedures (CT-scan, Tumour Markers [CA 19.9, CEA, CAR-3 and CA
195] ), and to the clinical rrelapse of specific symptoms. We analysed 30 patients (19 male 11
female; mean age 64,2 8,8 yr., range 50-86) with ductal adenocarcinoma of the pancreas which
underwent duodenopancreasectomy between 1988 and 1993. cases were T1NOM0 (6,6%), 24
(80%) T2 (11 (36,6%) NO and 13 (43,3%) N1), 4 (13,3%.) T3N0. 13 pt. were Stage (43,3%), 4
Stage (13,3%), 13 Stage III (43,3%). The complete follow-up related to clinical evidence of the
disease reviewed.
during the year US, CT-scan, EGDS and Tumour Markers performed every three
months;
from to I!1 year US, CT-scan, EGDS and Tumour Markers were performed every six
months;
from IV year US, CT-scan, EGDS and Tumour Markers were performed every year.
Seven patients excluded from the study for incomplete follow-up. CT were reviewed
blindly by two different radiologists. Two patients died, disease free, for not neoplastic cause
(survival 62,9 and 26,6 months) while patients is still alive 86,4 month after the surgical
resection without signs of The real survival rate calculated with the Kaplan-Meier was
20,67 2,7 months (range 3,9-86,4) with median of 16,6 months. The free disease time
11,38 2,85 months with median of 5,5. The type of diffuse (peritoneal
carcinomatosis in 4 pt. (20%), local (near the pancreatic stump) in 4 (20%), pure liver
metastases in (35%) and in 5 pt. (25%) nodal recurrence found. In 3 pt. (15%) the
recurrence detected by CT scan, in 7 cases (35%) observed increase of the tumour
markers title, in 4 cases (20%) the patients showed specific symptoms (back-pain) with low
tumour markers title without any evidence of at CT-scan. These symptoms preceded
the morphologic evidence of 3 months (2 CT-scan, CT-scan tumour markers,
laparotomy). In the other six positive: tumour markers and specific symptoms (3
15%), CT and tumour markers (1 pt. 5%) and CT and specific symptoms (2 pt. 10%).
The tumour rarker CA 19.9 positive in 10 pt.(50%) with recurrence, CA 195 in 6(30%),
CAR-3 in 5 (25%) and CEA in 3 (15%). The association tumour markers specific symptoms
positive in 17120 pt. (85%), tumour markers CT in 16/20 (80%) and CT
symptoms in 13/20 (65%). The statistical analysis by X2 did not demonstrate any significance
analysing the different associations (P=0,9). An aggressive follow-up not justifiable in
pancreatic cancer owing to the lackness of effective adjuvant therapies. An early detection of
recurrence however may allow differentiated therapeutic strategies. The association of clinical
symptoms and serum markers assay seem to have good sensibility and the most favourable
cost benefit ratio. CT-scan should be reserved to patients with high suspicion of recurrence,
with the aim of better define it.
P229 P230
ASSESSMENT OF DIFFERENT TYPES OF RECONSTRUCTION
FOLLOWING PANCREATIC HEAD SURGERY
K. Hanasawa H. Hoshi, Y. Kurumi, A. Nishimura, Y. Endo, T. Tani, J. Shibata,
M. Kodama
First Department ofSurgery, Shiga University of Medical Science, Shiga, JAPAN
VariOus reconstructions following pancreatic head surgery were evaluated for
anastomotic complication, remnant pancreatic function and nutritional status. The
pancreatic head surgery was performed for 10 lower billiary tract carcinomas, 6
duodenal carcinomas, 11 papilla Vater carcinomas, 18 pancreatic head tumors, 2
gastric carcinomas, 2 gall hddercarcinoma and 2 chronic pancreatitis. These include
40 pancreatoduodenectomies (PD), 8 pylorus-preserving pancreatoduodenectomies
(PpPD) and 3 duodenum-preserving pancreas head resections (DpPHR). PD was
performed for pancreatic head carcinomas, lower billiary tract carcinomas, gall
bhdder carcinomas, papilla Vater carcinomas and gastric carcinomas. PpPD was for
part of papilla Vater carcinomas and mucin producing tumors of pancreas head. All
cases of chronic pancreatitis were treated with DpPHR. In PD cases the
reconstruction was done in conventional "Child reconstruction" with duct-mucosal
pancreatojejunostomy (type IIA, 17 cases), with invaginated pancreatojejunostomy
(type KB, 19 cases) and end to end gastrojejunostomy with pancreatogastrostomy
(type IVC, 4 cases). In PpPD cases end to end gastrojejunostomy with duct-mucosal
pancreatojejunostomy (type IIIA, 3 eases) and IVC (5 cases) was performed. All
DpPHR cases were reconstructed in type IVC. In 40 PD cases, 8 cases ofleakage in
panereatojejunostomy was observed, 5 in IIA type (including 4 major leakage)and
3 in IIB type. All 4 major leakage and 3 of 4 minor leakage cases had non-dilated
pancreatic duet. In 8 PpPD eases, no panereatojejunostomy leakage was seen but one
hepaticojejunostomy leakage. In DpPHR cases, minor leakage from the preserved
duodenum was seen but no panereatogastrostomy leakage was observed.
The leakage from panctreatojejunostomy was seem in high incidence with duct-
mueosal anastomosis especially non-dilated pancreatic duet eases. On the contrary,
the leakages were minor in invaginated panereatojejunostomy or gastrostomy and all
those complications were conservatively manage& No leakage from
panereatogastrostomy was seem and those finding suggested pancreatogastrostomy
is safe and reasonable anastomosis even in non-dilated duct pancreas.
STUDY ON CANCER METASTASIS TO THE LYMPH NODES
AROUND THE PANCREATIC HEAD AND INDICATIONS FOR
PYLORUS-PRESERVING PANCREATODUODENECTOMY
H_Hara,K. Okajima,H. Isozaki,S. Morita,T. Ishibashi,S. Sako,K. Fujii
Department of Surgery,Osaka Medica College,JAPAN
Purpose The present study was designed to evaluate the lymph node
metastasis, especially paragastric lymph node, in patients with cancer of the
pancreatic head region and to clarify the indications for pylorus-preserving
pancreatoduodenectomy (PpPD).
Materials and methods: Postoperative his,rathological examination
were adequately examined on the fifty-six patients (23 patients of pancreatic
cancer, 21 patients of cancer in the papilla of Vater,12 patients of distal bile
duct cancer), who were surgically treated during the past 16 years in the
department of surgery, Osaka Medical College.
Results: Lymph node metastasis was frequently observed in: 1) pancreatic
head cancer showing the infiltrative type or duodenal infiltration, 2) cancer of
the papilla of Vater showing the ulcerative tumor type or pancreatic
infiltration, 3) distal bile duct cancer showing the infiltrative type or
pancreatic infiltration, 4) moderately or poorly differentiated cancer.
Paragastric lymph node metastasis was observed in 3 patients with pancreatic
head cancer,one with distal bile duct cancer,but none with cancer of the
papilla of Vater. All the three patients of pancreatic head cancer with
paragastric lymph node metastasis showed the infiltrative type and the
infiltration to the second part of duodenum. The one of distal bile duct cancer
with paragastdc lymph node metastasis showed the nodular infiltrative type
and was diagnosed as poorly differentiated adenocarcinoma. No. direct invasion
of cancer to the first part of duodenum or the stomach was observed in this
series.
Conclusion: Indications of PpPD are pancreatic head cancer without
duodenal infiltration, cancer of the papilla Vater, and distal bile duct cancer
without pancreatic invasion.
128
P231 P232
THE USEFULNESS OF HELICAL COMPUTED TOMOGRAPHY FOR
THE DIAGNOSIS OF THE CYSTIC NEOPLASM OF THE PANCREAS.
A.Horiguchi,S.Miyakawa,K.Miura
Dept.of Surgery,Fujita Health University,Toyoake Japan.
Helical (spiral) CT scanning, a volumetrical CT scan, has enable
us to scan the entire pancreas during a single breath hold, so that
even a small lesion of cystic neoplasm has been able to be
demonstrated. Additionally, high quality images of multiple
planner reconstruction (MPR) or three-dimentional
reconstruction (3-D) were able to be obtained due to continuous
data acquisition, and early phase imagings of dynamic CT could
be acquired because of high-speed rotation of the scanner.
Helical CT was performed on 13 cases with cystic neoplasm of the
pancreas. In the mucin hypersecreting tumors of the pancreas,
septal formation papillary rising in the cystic lesions showed
distinctly. In the serous cyst adenoma of the pancreas, septal
formation ,small caltifications, honey comb lesions were showed
distinctly. In the solid cystic tumors of the pancreas, we could
discriminated between solid lesion and cystic lesion by helical
CT.
On the basis of our data ,it was conclueded that helical CT is
superior to conventional CT in detecting cystic neoplasm of the
pancreas.
CARCINOID TUMOKSOF THE PANCREAS. CRITICAL
REVIEW BASED ON TWO PATIENTS AND 37 REPORTED
CASES.
qohn M. Howard, M.D.
Medical College of Ohio, Toledo.
The literature on carcinoid tumors of the
pancreas is confusing as the term has
erroneously included a broad spectrum of
islet cell tumors.
The following criteria are proposed as a
basis for diagnosis:
1. A demonstrable pancreatic neuro-
endocrine tumor
2. The finding of at least one of the
following without another dominant hormone
being demonstrated:
a) elevation of 5-Hydroxytryptamine
(serotonin) in the serum or in tumor tissue
b) elevation of 5-Hydroxy indole acetic
acid (5-HIAA) in the urine
c) positive argyrophilic or argen-
taffin tumor stains.
Based on these criteria only 39 "true"
carcinoids have been identified. The
diagnosis has usually been missed, often
confused with chronic pancreatitis. The
tumors are therefore usually large, mean
diameter 4.4 cm (0.5-12 cm), and have
metastasized. Thirty-two (82%) have been
malignant; thus often associated with a
carcinoid syndrome. The prognosis for long
term cure is currently not good; long term
survival being low. Radiologically the
tumor is usually oval, hypodense with
hyperdense capsule on CT; often revealing
increased vascularity and focal areas of
calcification. When a diagnosis of chronic
pancreatitis is made, measurement of urinary
5-HIAA should be considered.
P233 P234
SAFE PANCREATOJEJUNOSTOMY AFTER WHIPPLE'S PROCEDURE
BY DUCT-MUCOSA ANASTOMOSIS WITH TOTAL PANCREATIC
DRAINAGE THE PRELIMINARY EXPERIENCE
Chih-Jen Huang, Cheng-Chung Wu, Dah-Cherug Yeh, Hurng-Sheng Wu, Tse-Jia Liu,
Fang-Kang P'eng
Department ofSurge, TMchung Veterans General Hospital, Taichung, Taiwaaa
In spite ofimproving surgical techniques, panereaticojejunostomy (PJ) remains
decisive factor ofthe ofWhipple's pancreaticoduodenectomy. In the past,
there three categories ofPJ: (1)invagination ofpancreatic stump into the lumen
ofintestine (2)direct suture ofpancreatic duet to jejunal mucosa (due-to-mucosa
technique) (3)direct contact ofpancreatic duct to jejunal mucosa with total pancreatic
juice drainage and without suture. Based the experience ofpast authors,
speculate that (1)healng ofPJ is best facilitated by primary healing ofpancreatic duct
to jejunal mucosa. (2)contact ofthe pancreatic juice to intestinal content should be
delayed until complet,', healing ofPJ in order to prevent activation ofpancreatic
enzymes. Therefore, devised technique ofPJ by simultaneous combination of
duct-to-mucosa technique and total pancreatic juice drainage. After resection of
pancreatic head and d,aodenum, two purse-string sutures using fine polyglycan
material applied to the margin ofmain pancreatic duct mad jejunal hole which
prepared for PJ, respectively. Both purse-string sutures fastened until
stenting tube was inserted into the main pancreatic duet and the duct-to-mueosa
sutures (non-absorbable Ticron material) were tied. The stenting tube was kept forl
month. Within an 18-aaonth period, we used this technique 31 patients who
underwent Whipple's procedures for periampullary lesions. Atnong them, five
patients pyloric-preserving resections and three patients received combined
resection ofportal vein. The mean(_+SD) diameter ofmain pancreatic duct
3.03(+_2.01) and in 18 patients it 2rain. The pancreatic parench)qna
estimated soft in 20 patients. The daily amount oftotal pancreatic drainage was
greater in patients with soft pancreatic parenchyma than had parenchyma (205.9+89.6
89.2+95.9 ml, p<0.02). Seven patients had complications but died of
operation. There tvo patients had dirty discharges from peripancreatic drain
tubes xvhich were removed the 24th and 28th day after operation. Nevertheless, the
amylase level ofthe discharge is low. The mean(+SD) postoperative hospital stay
16.5(+7.6) days, range 10 to 34 days. Although aral pancreatic enzyme should be
prescribed before oTing ofthe stenting tube, had long term pancreatic
insufficiency. Based preliminary results, may speculate that duct-to-
mucosa anastomosis with total pancreatic drainage may be thc most suitable method
ofPJ after Whipple's r.'section.
EXTENDED PANCREATICODUODENECTOMY: A NON JAPANESE
EXPERIENCE
C. lacono*, E. Facci*, L. Bortolasi*, G. Mangiante*, G. Prati*, G. Zamboni,A.
Scarpa,G. Serio*
Departments of Surgery* and Pathology University of Verona, Verona, Italy
Extended pancreatico-duodenectomy (EPD) has been proposed by Japanese
Authors in the attempt to improve the survival of pancreatic carcinoma (PC), in
spite of this has not aquired yet great diffusion in the Western countries.
The aim of this work is to report our experience in 24 EPDs performed from
April 1993 to October 1995. Eighteen pts were male and 6 were female; mean
age was 60 years (range 41-74). Sixteen pts had PCs, had intraductal
mucine producing tumor (IMPT) and 7 periampullary carcinomas (PAC). The
operative risk was evaluated according to ASA Classification: 7 pts were ASA
14 pts were ASA and 3 pts were ASA II1. Seventeen EPDs were carried
out with gastric resection and 7 with pylorus preserving techinique. Median
operative time was 360 min. (Interquartile Range: IQR 80). The median
volumes of transfused blood and plasma were 800 ml (IQR 750) and 800 ml
(IQR 700) respectively. Operative mortality was nihil. Complications were
present in 11 pts (45.8%); 9 pts (37.5%) had surgical complications. Median
recovery time was 16 days (IQR 11.5). The mean number of removed lymph
nodes of first and second level was 34 (range 16-61); the mean number of
lymph nodes of first level was 18 (range 16-61) and of second level was 16
(range 8-31). Three pts out of 16 with PC had first and second level negative
nodes, 5 pts had first level positive nodes and second level negative nodes 2
pts had first level negative nodes and second level positive nodes and
eventually 6 pts had first and second level positive nodes. The pt with IMPT
had negative nodes. Two out of 7 pts with PAC had first level positive nodes.
Eleven pts with PC, the pt with IMPT and the 7 pts with PAC are alive and
without evidence of disease at mean follow up of 15 months (range 2-32); pt
with PC is alive at 9 months with local recurrence and another pt with PC at 6
months with local recurrence and liver metastases. Three pts with PC passed
away at 15,16 and 28 months for metastases without local recurrence.
In conclusion EPD is a surgical procedure that is feasible even in Western pts,
and has a low operative risk similar to standard PD's one; allows moreover
a more correct staging and reduces local recurrence. We cannot assume
any conclusion about the survival because follow up time is too short however
we think that adiuvant therapies are necessary to prevent and reduce distant
metastases that this operation does not seem to decrease.
129
P235 P236
ACCUTE NECROTIZING PANCREATITIS
STRATEGY AND TREATMENT
N.Jaramov, I.Viachky, E.Kolarov, V.Muikov,
M.Miteva
Emergency Surgical Clinic, Medical University
of Sofia, University Hospital "Queen Joanna"
(Bulgaria)
Accute necrotizing pancreatitis is one
of the principal problems of the acute abdo-
men. Nowadays this form reaches 35% of all
cases of acute pancreatitis. Method of necrec-
tomy followed by postoperative prolonged lo-
cal lavage in the area of pancreatic couch
was performed in a prospective study in cohort
of i00 patients with acute necrotizing pan
creatitis. The average number of Ranson's
Pognostic Criteria was 4,5.
Of the patients, in 80% we observed
subtotal to total necrosis of pancreatic pa-
renchma, in 75% necrotizing parapancreati-
tis or retroperitoneal destruction with
retroperitoneal phlegmon. The used method
significantly reduced mortality (. 19%).
Necrectomy with prolonged postoperative
lavage lead to increased postoperative survi-
val in patients with necrotizing pancreatitis.
By means of postoperative local lavage it is
possible a prolonged evacuation of biologi-
cally active substances and devitalized tis-
sues to be performed, evading destruction of
vital exocrine and endocrine zones.
EXOCRINE PANCREATIC ENZYMES-SUBSTITUTIONAL THERAPY
IN PRACTICE
A. Jelenkovie*, I. Stamenkovie**, R. Arsie**
*Department of Pharmacology and Toxicology, **Ciinic of
Gastroenterohepatology. School of Medicine, Nia, Serbia,
Yugoslavia
Pharmacological effects of drugs can be expected only if
certain conditions are met. One of them is taking
recommendable doses. The research of outpatient
utilization of all drugs which also includes pancreatic
enzymes, in municipality Ni {241800 inhabitants) in the
1985-1989 period was based on all presribed drugs handed
out in the Associated Pharmacies Nia (annualy on the
average 3232002 prescriptions). The expenditures for all
the drugs were covered by health insurance. Utilization
is expressed as defined daily doses (DDD)/1000
inhabitants /day. The DDD is a chosen average daily
quantity of the drug most frequently used in most
frequent indications in adults. The utilization of
pancreatic enzymes amounts to 0.93 DDD/i000 inhabitants
/day in comparison to 309 DDD/1000 inhabitants/day for
all drugs. It means that about 0.01% of the population
of Ni take pancreatic enzymes every day. But, in
'eality, a far greater number of people take those drugs
as shown by a study on the prescribed daily doses (PDD)
of pancreatic enzymes in which 41 physicians were
interviewed gastroenterologists-7, internists-4,
specialists in occupational medicine-5 and eneral
practitioners-25}. On average the PDDs are 36-75% of the
DDDs. The smallest changes of the DDDs are made by
gastroenterologists (the PDDs are 62.5-75% of the DDDs).
The biggest differences are found ira primary health Care
i.e. with general practitioners and specialists in
occupational medicine {the PDDs ar 36-48% of the DDDs).
The expected optimal therapeutic effects of pancreatic
enzymes cannot be gained by such dosage; in fact it
leads to insufficient or, even to placebo effects.
Besides that, it is evident that therapeutically
educational influence of gastroenterologists on other
physicians is unacceptably weak.
P237 P238
PROBLEMS IN SURGICAL TREATMENT OF
PANCRFTOJEIUNOSTOMY DEItISCENCE
M.Jeremic, M.Stojiljkovic, M.Visnjic, A.Bogicevic, V.Pejoic, G.Stanojevic,
M.Stojanovic, P.Stojanovic, SZeremic
SURGICAL CLINIC, CLINICAL CENTER NIS, YUGOSLAVIA00
Pancreatojejunostomy (PJA) is very delicate operative procedure, with high
percent ofanastomotic leakage and high mortality rate. The autors shows a serie
of 55 PJA perfoxmed in the ten years period. Thirty-seven anastomoses were
performed after cephalic duodenopancreateotomies, 14 after left
panoreateotomies and 4 inside by-pass operations. Indications were as follows
malignant tumour of periampullary regions (37), malisnancy of body and taft of
pancreas (14), chronic pancreatitis (3) and avulsion of papilla Vateri (1). Two-
layers anastomotio tedmique was done, with telescopic anastomosis in selected
oases. Anastomotic leakage oocured in 12,46% (8 cases). Rate of this
complications has been much higher at elderly patients with malignancy.
Dehiscence of PJA was treated medicaly in 62,2% (5 oases), with parenteral
hyperalimentation, and correction of fluid and acidobases balance). In 3 cases
suppression of pancreas secretion with Stylamine were done. Reoperation was
performed in 3 cases with one reanastonmsis and 2 drainages and later
reimphntation of fistulous chanel in the small bowel The authors work out in
detail criterias about the decision for reopoemtion and due time for
reintervention. Reanastomotic leakage ocoured in case (early intervention).
Mortality rate was 37,5% (40% conservative and 33,3% surgicaly treated). The
best results have been achieved with the combination of carefully selection of
patients and high surgical tedmique and experience. Estimate of gold moment
for reintervention is essential in treatment of PJA dehiscence.
THE CHOICE OF OPERATION METHOD IN CHRONIC PANCREATITIS
COMPLICATED BY PSEUDOCYST LESIONS
M.Jesipowicz, S.Rudzki, S.Stettner, Z.Kosut,
J.Jesipowicz
The First Department of General Surgery
Medical Academy in Lublin, Poland
Pseudocyst lesions are complications arising from
chronic pancreatitis or as the result of chronic chan-
ges following acute pancreatitis. The occurance of
pseudocyst lesons.increases and varies from 0,004% to
0,01% in hospitalised patients. The aim of the present
study is to choose the method of treatment in accor-
dance with: the location of pseudocyst lesions in pan-
creas, their quantity and spreading, their relation to
pancreatic duct, and with the picture of pancreatic
duct. Diagnoses were achieved by using USG, CT, ERCP
examinations and intraoperative pancreatography. There
were 148 patients operated on in the years 1975-1995.
In i06 different kinds of pancreatic and pseudocyst
lesions resections were applied. Various types of
internal anastomoses were performed in 28 patients,and
18 patients underwent external drainage. Pseudocyst
lesions situated at caput of pancreas pose special
difficulties which require individual approach in each
case. Internal drainage by endoscopy was not applied.
No recurrence of pseudocysts appeared after resective
operations. We observed: 3 recurrences of pseudocysts
and chronic pancreatitls remaining in 2 cases after
internal drainage, as well as one pancreatic fistula
following external drainage. After resection operation
diabetes was found out in 2 cases in distant postopera
rive period. In our experience decision as to the
operation method ought to be taken on the basis of the
general condition of the patient and local technical
possibilities.
130
P239 P240
PALIATIVE TREATMENT FOR PANCREATIC MALIGNANCIES ROLE OF THE
OPERATIVE BY-PASS PROCEDURES
Jovanovi6 M., Jeki6 IM, Bilanovi D, (olovi6 R, Mili6evi6 MN.
The First Surgical Clinic, Clinical Center of Serbia, Belgrade, Yugoslavia
INTRODUCTION Aim of the study was to analyze the effects of biliodigestive
(BD) by-pass procedures in management of inoperable pancreatic head
cancer.
MATERIAL AND METHODS A retrospective study was carried out for a
thirteen year period ranging from 1982. to 1994. There were 371 pts. Sex ratio
was M/F 2/1 248/123 ). Age ranged from 23 to 90 years, mean M-59,3 and
F-62,8. Fifteen pts.(4%) were below forty years old, 158 pts. (42,6%) 40-60,
while 198 pts. (53,4%) were over sixty. Mean symptom at the time of
admission was jaundice in 326 pts.(87,9%) and pain in 304 pts.(81,9%).
Cholangitis was present in 37pts.(10%). The onset of jaundice was painfree in
almost three fourth of pts.-269 (72,5%). Duration of jaundice varied from 3 to
180 days prior to operation. Almost half of the patients, 147(45,1%) were
jaundiced over three weeks at the time of admission. Bilirubin level varied
from 16 to 939, mean 247,4. Twenty five patients already had a BD procedure,
15 of the pts. without an GEA (gastroenterostomy). Operative procedure was
HJA (hepaticojejunostomy) in 284 pts.(76,5%) without GEA 28 pts., HDA
(hepaticoduodenostomy) in 36 pts. (9,7%), without GEA in 24 pts., and other
forms of by-pass procedures in the rest of 45 pts. (12,1%). In six patients,
(1,6%) no operative by-pass procedure was possible. Specific morbidity was
low, biliary fistula occurred in 15 pts.(4,0%), followed with diffuse peritonitis in
6 pts., while ascending cholangitis was present in 7 pts.(1,8%). Upper GI
hemorrhage occurred in 22 pts. (5,9%) and wound infection in 19 pts. (5,1%).
Early postoperative mortality was 33 pts. (8,9%). In thirty patients (8,1%)
hepatorenal syndrome had developed. All patients that died were jaundiced
more than three weeks.
DISSCUSION Most patients were treated in an advanced stage of the
disease with increased intraoperative risk. Combined BD and digestive by-
pass procedures are feasible in most patients. The preferred BD procedure is
Roux-en-Y HJA with a jejunal loop no shorter than 60 cm. Gastric outlet
obstruction was prevented by retrocolic GEA. Average life expectations are 12
months.
CONCLUSION Properly done combined BD and digestive by-pass
procedure could provide good palliation for the duration of life. Although the
life expectancy of such patients is relatively short the quality of life is
acceptable and these patients do not die due to bile duct obstruction.
EFFICACY OF SOMATOSTATIN IN THE PREVENTION OF
POSTOPERATIVE COMPLICATIONS FOLLOWING
PANCREATIC SURGERY.
J.M.Jover; J.C. Adana; J. Lopez Herrero; J.L.
Ramos; C. Maillo; P. Artufiedo; M. Moreno
Azcoita.
Department of surgery. Hospital de Getafe.
Madrid, Spain.
Group A
Group B
* p =0.03
The aim of this study was to determine
whether prophylactic somatostatin infusion
can prevent postoperative complications after
pancreatic surgery.
PATIENTS: A randomized prospective study was
undertaken using two groups of patients
Group A (n=20) received prophylactic
somatostatin (250g/h in continous perfusion
for 5 days after surgery). Group B (n= 22)
which did not receive somatostatin. These
patients had undergone pancreatic surgery
(resections or drainages) between January
1993 and October 1995.
RESULTS: both groups were homogeneous
regarding sex, age, and diameter of the duct.
Type of surgery:
Drainage Distal Res. Pancreatoduo.
Group A 1 14 7
Group B 3 6 Ii
Pancr. Morb*. Mort. Reope. Hospital
Fistula stay
10% 35% 5% 10% 27d
22.7% 68.2% 13.6% 22.7% 24d
CONCLUSION. In sumary the results of this
trial show that somatostatin after
pancreatic surgery reduces the morbidity,
mortality, reoperations and incidence of
pancreatic fistulas.
P241 P242
SURGICAL TREATMENT OF CHRONIC PANCREATITIS.
D.Karcz, B.Ke_.dZ H. Labza, T.Popiela.
81st Department of General and GI Surgery, Jagiellonian
University, Krak6w, Poland.
The aim of study is the analysis of 419 patients treated
for chronic pancreatitis. The authors present diagnostic
procedures and indications to surgical treatment of
patients with chronic pancreatitis.
Material Analyzed group included 99 females and 320
males. Diagnosis was on the clinical examinations and
diagnostic procedure (ERCP, USG, CT, FNAB). Out of
this group 93 were .qualified to surgery. In 56 patients
we performed resective procedure and in the remaining
37 surgical decompression. Non-surgical decompression
was performed in other 152 cases (endoscopic
papillotomy 56, percutaneous draining 96).
Results Resective procedures are more effective than
the decompression in the treatment of chronic
pancreattis. Alternative non-surgical decompression
resulted in obtaining temporary effects which are
however less satisfactory than after surgery.
The Effect Of Nesidioblastosis In The
Streptozotocin Induced Diabetes In Rats
A. Kartal, C. Vatansev, A. Kaymakfl, S. Yol, M.
Belviranh, S. Duman, A.Aslan
Department of Surgery, University of Selcuk, Konya,
Turkey
Forty rats with streptozotocin-induced diabetes
were divided into four groups of ten animals. The
first group had sham operation. In the second, third
and fourth groups of animals, the head of pancreas
were wrapped with polypropylen, chromic cat-gut,
and polysulphon, respectively.
At the end of the third week 14 percent of
first, 43 percent of second, 40 percent of third and
33 percent of fourth groups of animals recovered
from diabetes both clinically and biochemically.
Microscopic examination of rat pancreas was partly
compatible with biochemical results because of light
microscopic studies.
We concluded that, experimental
nesidioblastosis was not specific to either animal
pancreas or wrapping material; as well as hamster,
rat and as well as celophan as material
polypropylen or chromic cat-gut will cause
nesidioblastosis.
131
P243 P244
WIRSUNGO DUODENOSTOMIA AFTER PANCREATODUODENECTOMY FOR
CANCER OF THE PAPILLA OF VATER.
G,Keleti, N.De Negri,G.Hollts
Dept.of Surgery.St.Lszl6 Hospital.Budapest.Hungary
Between 1989 and 1993 cancer of the papilla of rater
was surgically treated in 20 out of 26 cases.18 pa-
tients underwent duodenopancreatectomy and two had a
papillectomy. In the cases of early cancer we have de-
velopped a new procedure including a pylorus preser-
ving pancreatectomy with the resection the only middle
part of duodenum and an anastomosis between the Wit-
sung duct and the distal part of duodenum.There were
no surgical or hospital deaths in these 20 patients.
The most common postoperative problem was the delayed
gastric emptying and four of them required reoperation
There were only 1 leakage of Wirsung anastomosis.
Three years after pancreatoduodenectomy fourteen pa-
tients have been alive with a satisfactory quality of
life.Two patients with sever pancreatic invasion to
the pancreatic parenchyma have survived only 18 and
22 months after pancreatoduodenectomy.
We believe that the minimal pancreatoduodenectomy
seems to be preffered in the treatment of the early
caner of the papilla of Vater ,while in patients
with advanced cancer an extended pancreatoduodenectomy
neds to be done.
A STUDY OF SURGICALANATOMY FOR DUODENOM,PRKSERVING
RESECTION OF THE HEAD OF THE PANCREAS AND A PROPOSITION
OFA NEW PROCEDURE
W. Kimura, N. Futakawa, H. Shinkai, I. Hart, T. Inoue, Y. Takei, K. Morikane,
T. Muto, A. Kuroda, H. Nagai'.
Department of Surgery, the University ofTokyo, and "Jichi Medical University,
Japan
[Aim] To precisely examine the topography of the duodenum, pancreas, bile
duct and supplying vessels, for duodenum-preserving resection of the head of
the pancreas.
[Matefia and Methods] Forty autopsied cases were studied, in.which the head
of the pancreas was well preserved and no pathological lesion was found. The
local anatomy of this region was grossly examined.
[Results] 1. The connective tissue membrane of the posterior surface of the
pancreas was found in all of the cases, and most of the pancreaticoduodenal
arteries and veins are situated on this membrane. 2. The arcade formation
between the ASPD and the AIPD was found in all of the cases. In 88% of the
cases was the arcade demonstrated between the PSPD and PIPD. This arcade
was always located dorsal to the common bile duct. Arterial branches to the
common bile duct and the papilla of Vater were divided from PSPD. 3. After
departing from the gastroduodenal artery, ASPD ran toward the point, 1.5 crn
below the papilla of Vater, then turning around to the posterior aspect ofthe
pancreas to join AIPD. Thus, to the contrary to the prevailing notion, AIPD was
found on the "posterior" surface of the pancreas. 4. The artery toward the papilla
of Vater ran along the fight side of the common bile duct. No such big artery
toward the papilla was found. 5. No case was found, in which ASPD, AIPD,
PSPD, PIPD, and their branches to the duodenum, the bile duct and the papilla
of Vater were completely buried in the pancreatic parenhyma.
[Conclusion] It may be possible to remove the head of the pancreas, while
preserving the vascular arcades and their branches.
[A Proposition of ANew Procedure] We proposed a new procedure of duode-
num-preserving subtotal pancreatectomy of the pancreas. A reason why we
leave the part of the pancreas between the duodenum and the ASPD and the
common bile duct is that the artery toward the papilla of Vater ran along the
right side of the common bile duct and would be difficult to be preserved with
the removal of this part of the pancreas. The most important technique of this
procedure is to keep the connective tissue membrane of the posterior surface of
the pancreas intact, so as to preserve pancreaticoduodenal arteries, because all
the pancreaticoduodenal arteries and veins are situated on this membrane.
P245 P246
CYSTIC TUMORS OF THE PANCREAS
Takashi Kodama ", Takashi Yokoyama2', Masaki Ito3', and Yuichiro Matsuura
First Department of Surgery' General Medicine First Department of Internal
Medicine3', Hiroshima University School of Medicine, Hiroshima Japan
Despite the recent increased attention to pancreatic cystic neoplasms, there have
been inadequate appreciations of the different types of cystic tumors and of the
diagnostic and therapeutic approaches. In evaluating our experience of cystic
tumors, we will emphasize the difficulties in achieving a definite diagnosis
without resection. From 1987 to 1995 we treated 26 patients (14 women, 12
men; mean age, 62 years with cystic neoplasms of the pancreas including
serous cystic adenoma, 6 mutinous cystadenomas, mucinous cyst
adenocarcinoma, 6 mutinous ductal ectasias, 5 papillary adenocarcinomas, 2
solid and cystic tumors, 2 adenocarcinomas with cyst formation, and
malignant tumors with cystic pattern (1 sarcoma, combined carcinoma, and
non-functioning endocrine tumor). Mean tumor size was 4 cm 1.5 to 11.5 ).
Eight patients(30%) had no symptoms. Computed tomography was useful for
detection of cystic lesion and for showing rim calcification, but was not reliable
for distinguishing neoplasm. Arteriography showed hypervascularity in serous
adenoma, solid and cystic tumor, and malignant tumors with cystic pattern.
Endoscopic pancreatography showed no communication with the cyst cavity in
14 of 26 cases of cystic neoplasmas but presented the ectatic ducts in 10 cases
of mutinous ductal ectasia and papillary adenocarcinoma. Stenosis or
obstruction of the pancreatic duct suggested to be cancer. Cytological
examinations including p53 staining was useful to distinguish benign from
malignant tumor in some cases. The tumor was resected in 9 cases by distal
panceateetomy, in 13 by proximal resection, in one by total pancreatectomy, in
3 by enucleation with no operative deaths. Twelve tumors(46%) were
malignant. Seven cases (60%) resected for cure are alive without evident
recurrence.Cystic tumors of the pancreas represent a spectrum of disease ranging
from benign cystadenoma to adenocarcinoma. We recommend resection
whenever possible, even if preoperative evaluation suggests benign disease.
Stomach-Preserving Gastric Bypass for
Unresectable Pancreatic Cancer
M. Konishi, M. Ryu, T. Kinoshita, N. Kawano, H. Tanizaki, A. Cho
Dept. of Surgery, National Cancer Center Hospital East, Kashiwa,
Japan
We attempted stomach preserving gastric bypass (SPBG) for
patients with unresectable pancreatic cancer. A stomach preserved
as much as possible was bypassed to jejunum by end-to-side
anastomosi.s. Twenty five patients were underwent SPG.B from 1992
to 1995. In five patients, another bypass was carried out and 44
patients had no gastric bypass in same period. The mean operative
time for SPGB was significantly longer than that for another bypass.
However the mean intraoperative blood loss was almost same. The
operative morbidity was 28% and there was no operative death.
Although the high incidence of delayed gastric emptying was found
(24%), the comfort index (ratio of duration of good palliation to
duration of survival) of patients with SPGB exceeded 50% when
cancer extension limited to regional or small amount of systemic
metastases. That of patients with another bypass or without gastric
bypass was less than 40%. Our results show that SPGB is a safe and
effective gastric bypass in patients with regional or small amount of
systemic metastases. In contrast, when large amount of systemic
metastases presented, mean survival was less than 100 days and the
comfort index was less than 30% in all groups. In such patients, non-
surgical palliation should be performed if they did not have symptoms
of gastric outlet obstruction.
132
P247 P248
SURGICAL TACTICS IN CHRONIC PANCREATITIS
COMPLICATED OBSTRUCTIVE JAUNDICE
V.Koubyshkin, T.Savvina, A.Vihorew
A.Vishnevsky Institute ofSurgery, Moscow,Russia
The results of various surgical teeniques for chronic
pancreatitis(CP),complieated by obstructive jaundice,is
presented based on the experience of the treatment of 53 pts.
over 18 years.On investigation 20 of them had
calculi(parenchymatous or duetal),19 had single or multiple
cysts of the pancreatic head and 14 had extensive fibrosis of
the pancreas. In all patients the diagnosis was verified
intraoperatively by morphologie investigation.In the previous
years, for surgical treatment,we most frequuently used
combinations of internal drainage procedures(n=21). This
operations provided quick disappearence of jaundice and
pain,were associated with a minimal number of postoperative
complications and operative mortality of 4.8% (1 pts.).On
long term follow-up progression of the complications of CP
was observed and this led to the death of 6 pts.For this
reason,in recent years for CP we perform PPPDE (n=8) or
less frequently the classic Whipple proeedure(n=24).The early
and long-term results of this operation are
muchbetter.Postoperative complications are minimal and
mortality of 6,3% (n=2) was observed.Factors in support of the
use of the various forms of PDE for the treatment of CP
include deep degeneration of the pancreas and frequent
congenital anomalies, which are determinants of chronic
inflammatory processes or tumorous metaplasia,and which we
observed on morphologic examination of operative specimens
following Whipple procedures.
ACUTE NECROTIZING PANCREATITIS
Ko.elj M, Borovak Z, Flis V, Potr S, Horvat M, University
HospitalMaribor, Dept.forAbdominal and Vascular Surgery,Intensive care
unit, Ljubljanska 5, 62000Maribor
The purpose of the present study was to compare the outcome of the
acute neerotizing pancreatitis (ANP) and the number and the nature of
the operations. It was a prospective trial which lasted from 1992 to
october 1995. Acute neerotizing panereatitis was classified according
to Atlanta consensus. 33 patients were included and were treated
surgically and in intensive care unit (ICU) (20 male, 13 female,
average 52.8, range 27-74). First operation was performed (average) 5
days after the onset of symptoms (range 3-11 days). 14 patients died
(42.4%). Average hospital stay in survivals was 62 days (range 33-
113) and average stay in ICU in survivals was 19 days (range 3-72).
Average hospital stay in patients who died was 36 days and average
stay in ICU was 32 days (range 1-80). The average number of
reoperations in the survival group was 3 (range I-5), the average
number of reoperation in the second group 1.5 (range 1-3). The
average APACHE-II score in the survival group was (atr 48 hours)
was 8 and in the second group 19. In the survival group the first
operation performed was external drainage in two cases, neerosectomie
with continuos local lavage in 9 cases and neeroseetomie with .open
packing in 8 eases. In the group with fatal outcome the first operation
performed was external drainage in 7 cases, necrosectomie with
continuos local lavage in 3 cases and necrosectomie with open packing
in 4 cases. The reasons for death was massive hemorrhage in two
patients, in.the rest of group multiorgan failure developed. In the
conclusion we may state that the severe ANP is-from the onset on very
malignant disease, the disease with high mortality and in our study the
mortality could not be decreased by different surgical approaches
neither by any other form ofcontemporary medical treatment.
P249 P250
INFLUENCE OF INTRAOPERATIVE ULTRASOUND ON SURGERY FOR
CYSTIC LESIONS OF THE PANCREAS
K. Kubota, T. Noie, H. Abe, K. Sano, Y. Bandai, M. Makuuchi
Second Department of Surgery, University of Tokyo, Tokyo, Japan.
A retrospective study was carried out to investigate the influence
of intraoperative ultrsound (IUS) on surgery for cystic lesions of
the pancreas (CLPs). Twenty-one patients with CLPs, including
five pseudocysts and 16 true cysts, were scanned using 5.0- or
7.5-MHz transducer intraoperatively. In 4 patients with
pseudocysts, two with retention cysts and one with a serous
cystadenoma, the operative procedures were performed under IUS
guidance. In 2 with pseudocysts, the site and size of cyst-
jejunostomy was decided using IUS, in one, IUS determined the
puncture point for the external drainage and in the remaining
one, distal pancreatectomy was performed under IUS guidance
without damaging the major vessels. In 2 patients with retention
cysts, IUS enabled us to leave no unpalpable of invisible small
cysts behind. One patient with a serous cystadenoma underwent a
laparoscopic-assisted procedure and IUS was useful for
confirming the location of the pancreatic duct. In one with a
pseudocyst, IUS showed that the cyst did not involve tha pancreas
and subsequently, cystectomy without pancreatic resection was
necessary. In one of the two with mucin-producing
cystadenocarcinomas, IUS demonstrated a skip lesion in the duct
of the pancreatic tail, which contributed to changing the operative
procedure from pancreatoduodenectomy to total pancreatectomy
and in the other, IUS showed that the lesion was localized in the
body of the pancreas and subsequently, segmental pancreatectomy
was carried out instead of scheduled pancreatoduodenectomy or
distal pancreatectomy. Thus in the three patients, the operative
procedures were changed in the light of the IUS f'mdings. In the
remaining 11 patients, although IUS visualized CLPs clearly, it
added no new information relevant to the operations. In
conclusion, IUS contributes to performing surgery for selected
CLPs, such as pseudocysts with severe inflammation, retention
cysts with unpalpable or invisible small cysts and mucin-
producing cystadenocarcinomas with possible other lesions.
THE SURGICAL TREATMENT OF CHRONIC PANCREATITIS
G. La Guardia, A, Frena, A. Imperiale, F. Martin, W. Thaler, P. Catalano,
M. Schellner, I. Valorzi, G.P. Marzoli
2nd
Department of General Surgery, Regional Hospital of Bolzano,
Bolzano, Italy
The methods of treatment of chronic pancreatitis have been controversial
for many years. Current trends in Europe are to perform more anastomoses
and less resections that some years ago. Neither has been proved to be
more efficient than the other.
Methods: from 1984 to 1995 we performed 119 operations in 105 patients
(85 men and 20 women; mean age 50.5 yrs.) who had chronic pancreatitis.
Of these operations, 48 (40,3%) were resections (31 distal
pancreatectomies, 15 duodenopancreatectomies and 2 total
pancreatectomies), 65 (54,6%) were diversion procedures (27 Partington-
Rochelle operations and 38 internal drainage of cysts) and 6 (5,0%) were
combined procedures (distal pancreatectomy and cystojejunostomy). The
indication for surgery was abdominal pain in 74% of patients,
biliary/duodenal obstruction in 14%, suspect of cancer in 12%. All
patients had severe pancreatic disease: 42% had pseudocysts, 44% had
severe duct dilatation with parenchymal atrophia and calcifications, 14%
had inflammatory mass of the head of the pancreas; furthermore 9 patients
had splenic vein thrombosis with left portal hypertension, 3 had
hemosuccus pancreaticus, 3 had pancreatic ascites, 3 had pancreatic
fistula.
Results: the postoperative mortality rate was 2.8% and morbidity occurred
in 16%. The incidence of diabetes after surgery was high (28.5%) after
resection, but is also occurred after diversion procedures (18.5%). The
quality of life (pain relief and exocrine insufficiency)' and lenght of
survival was similar after resections and after diversions, althaught
patients with alcoholic pancreatitis (35%) had the worst quality of life and
long-term prognosis.
Conclusions: there no single ideal operation for chronic pancreatitis.
Lasting pain relief occurred in 58.5% of our patients and was similar in
resective or in drainage procedures. Long-term survival and functional
results depend mainly on alcohol withdrawal. The outcome of surgery for
chronic pancreatitis is not poor, but is poor the outcome of this disease.
133
P251 P252
PANCREATICODUODENAL NECROSIS DUE TO CAUSTIC BURNS
S. Landen,H.H. Wu,L.B.B. Jeng,B. Launois
Departments of Surgery Rennes-France,Tainan-Taiwan
and Taipei-Taiwan
Fourteen patients having undergone extensive foregut
excisions for chemical burns extending beyond the pylo-
rus were included in a multicenter retrospective study.
Injuries always resulted from massive ingestion
of liquid caustics during attempted suicide. All
patients underwent duodenectomy or pancreaticoduodenal
resection and oesophagogastrectomy or total gastrectomy.
Immediate biliopancreatic reconnection was performed in
10 patients,with no added morbidity. Operative mortality
(50%) was due to uncontrollable septicemia and only
patient died as a result of surgical complications. A
trend towards a more favorable outcome was noted in the
group of patients operated within 12 hours of ingestion
ohcaustics,those undergoing a formal pancreaticoduo-
denal resection (versus duodenectomy),and those
having immediate biliopancreatic reconnection (versus
delayed econnection). A satisfactory quality of
life as defined by the ability to feed orally and the
preservation of voice function was obtained in 2 of 5
long term survivors. Functionnal recovery was unpredic-
table during the acute phase and depended on late
scarring of the airway-alimentary tract junction.
Chemical burns extending beyond the pylorus should
not be considered as being beyond surgical recourse.
Early radical debridement appears necessary to save
these patients who otherwise face inevitable death.
Duodenal necrosis should be managed by pancreatico-
duodenectomy followed by immediate biliopancreatic
reconnection.
A satisfactory quality of life can sometimes
be achieved but depends on late scarring of the
airway-alimentary tract junction.
IMPACT OF CONTINUING ALCOHOL ADDICTION ON THE
RESULTS OF RESECTIVE SURGERY FOR CHRONIC
PANCREATITIS
B. Launois, B. Chareton M. Foglia, O. Grard, C. Stasik, G.
Spiliopoulos, J.P. Campion.
Department of Digestive Surgery and Transplant Unit, CHR
Pontchaillou, Rue Henri Le Guilloux, 35033 Rennes, France
Materials and methods "Between 1972 and 1991, 149 patients
underwent resective surgery for chronic pancreatitis. Surgical
procedures included 87 pancreaticoduodenectomies and 62 distal
splenopancreatectomies. Excluded from the study were 5 patients lost
to follow-up and 10 patients having died in the post-operative period.
Rsults 46 patients had continuing alcohol addiction (OH+) and 88
patients had been weaned for alcohol (OH-). Post-operative comfort
was poor in 3.5% of OH- versus 30.4% of OH + patients, medium in
26.7% of OH patients versus 30.4% of OH + patients and good in
69.8% of OH versus 39.2% of OH + patients. Pain was present in 8%
of OH versus 57% of OH + patients. Body weight was stable or
increased in 100% of OH versus 47% of OH + patients. 53% of OH+
patients experienced weight loss. Return to normal activity was 85% in
OH versus 50% in OH + patients. 5, 10 and 20 year survival for OH
and OH + patients was 90% vs 75%, 85% vs 60% and 70% vs 30%
respectively. Following pancreaticoduodenectomy, 5, 10 and 20 year
survival for OH and OH + patients was 95% vs 70%, 90% vs 50% and
60% vs 50% respectively. Following distal splenopancreatectomy 5, 10
and 20 year survival for OH and OH + patients was 90% vs 90%, 70%
vs 70% and 70% vs 15% respectively. Late deaths occurred in 21 of 46
patients in the OH + group 5 oropharyngeal oesophageal tumors, 2
cardiovascular conditions, 9 acute alcohol realted complications, 5
"other" causes. Late deaths occurred in 17 OH patients, 5
oropharyngeal oesophageal tumors, 6 cardiovascular conditions, 9
acute alcohol related complications, 6 "other" causes.
,Conclusions The best results of pancreatic resection for chronic
pancreatitis are obtained in those patients who can be weaned from
alcohol. Comfort, return to normal activities and survival are improved.
P253 P254
RESULTS AFTER 5 AND 10 YEARS FOLLOWING SURGERY FOR
CHRONIC PANCREATITIS LONG TERM SURVIVAL IS
UNRELATED TO SURGICAL TECHNIQUE
B. Launois, G. Spiliopoulos, B. Chareton, C. Stasik, M.A.C. Machado,
J.P. Campion, B. Meunier.
Department of Digestive Surgery and Transplant Unit, CHR
Pontchaillou, Rue Henri Le Guilloux, 35033 Rennes, France
In papers published by LEGER (1)and MOREAUX (2) in 1974 and
1984 it was suggested that pancreaticoduodenectomy should be
banned from the surgical arsenal of chronic panereatitis because of the
disappointing long-term results. The aim of the present study was to
review long-term outcome of patients having undergone surgery in our
Department.
Patients and methods between 1972 and 1994, 381 patients
underwent surgery for chronic pancreatitis at a referral centre. There
were 322 males and 59 females with a mean age of 45 +/- 12.3 years.
Alcohol abuse was the prevailing etiological factor (89.7%). Surgical
procedures included 153 resections (87 Whipple procedures, 62 distal
pancreatectomies, 4 total pancreatectomies), 113 bypasses (40
pancreatic + biliary and or digestive bypasses, 73 biliary and or
digestive bypasses) 89 cystoenteric anastomoses, 4 splenectomies and
22 exploratory laparotomies.
Rtmdts Operative mortality was 7.8% for resections, 5% for pancreatic
es, cystoenteric anastomoses and exploratory laparotomies.
Morbidity was 17% for resections and cystoenteric anastomoses and
7.5% for pancreatic bypasses, 5 and 10 years survival was 92% and
76% for resections, 79.7% and 53.8% for cystoenteric anastomoses,
85% and 66% for pancreatic bypasses. (n N.S.). Diabetes mellitus
developed in 39% of patients who underwent resections and 37.5% of
patients having bypasses. Persistent pain was present in 24% of patients
following resection and bypass.
Conclusion the long-term outcome of patients with chronic
pancreatitis is unrelated to the type of surgery.
(1) LEGER L. Ann.Surg. 1974 180: 185-191.
(2) MOREAUX J. World J.Surg. 1984 8 346-350
134
SURGICAL TREATMENT OF PANCREATIC ISLET CELL TUMOURS.
Leautko J.. Popiela T., Hartwich A., Turczynowski W.
/st Department of Genera/and GI Surgery, Jagiellonian University, Krakdw,
Poland.
The aim of the study is the analysis of surgical treatment results in patients
with pancreatic islet cell tumours. Material: Between 1975-1995, 26
patients with pancreatic islet cell tumours were undergoing surgical
treatment. There were 16 patients with insulinoma, 3 females with
glucagonoma and 7 patients with Zollinger-Ellison syndrome (ZE). All
insulinoma patients had clinical symptoms of Whipple's triad and diagnosis
was based on the endocrinological criteria. Three patients with
glucagonoma had previous diagnosis of pancreatic canber and were
qualified to surgery. All patients with ZE were undergoing previous surgical
treatment and the final diagnosis in 6 of them was established in our
Department basing on the gastrin level in fasting state, after meal and
upon secretine test. Results: In 11 patients insulinoma was confirmed
intraoperatively and excised The results were satisfying in 9 patients
with benign insulinoma followed-up for about 10 years. One patient died
month after operation for metabolic complications and another one with
malignant insulinoma died 3 years after surgery. In the remaining 5 patients
insulinoma was not confirmed intraoperatively and histopathological
findings showed nesidioblastosis or #-cells hyperplasia. In the follow-up
period in one case profound hypoglycaemia developed soon after the
surgery, in other 4 patients mild temporary hypoglycaemia was corrected
by diet only. In 3 patients with glucagonoma undergoing surgery
immunohistological examination revealed e-cell pancreatic tumour. Re-
examination of these 3 cases showed mild diabetes mellitus in 2 patients,
and skin rash in one case. Out of 7 patients with ZE, gastrinoma was
confirmed intraoperatively in 4 cases and excised. Four patients received 5-
FU and streptozotocin. One patient with multiple liver metastases died one
year after surgery, and 5 patients were followed-up for 4 to 13 years.
Conclusions: 1. Surgical treatment is the method recommended in case of
histopathologically confirmed insulinoma. Completeness of excision should
be verified by monitoring blood glucose level or by using the Biostator
Controller. In cases of nesidioblastosis or #-cells hyperplasia partial or total
pancreatic resection is the only possible method of choice. 2.
Glucagonoma is the rare pancreatic islet-cells tumour. Blood glucagon
estimations is the only method enabling preoperative diagnosis. 3. Early
diagnosis and appropriate treatment is essential in patients with Zollinger-
Ellison syndrome. We recommend performing pentagastrin test in all
patients with short history of disease and severe symptoms of gastric and
duodenal ulcers.
P255 P256
OUR EXPERIENCE IN THE MANAGEMENT OF HEPATIC ANGIOMAS.
REPORT OF 45 CASES IN THE LAST 7 YEARS.
C. Loinaz, E. Moreno Gonz/dez, I. Garcia Garcia, R. Gomez, M. Manzanera, C.
Castellon, A. Calle.
The hepaticangiomas(HA) are the most frequentbening hepatictumours. The surgical
indication and evolution of non resected cases still controversial.
We present a retrospective trial of 45 patients with HA since 1986. Thirty-three
patients were females and 12 males. The median age was: 50.3 (Range 32-82 years).
Clinical picture: pain (23 patients), hepatomegaly (5 patients), other digestive
symptoms (18 patients) and fortuitous finding (8 patients). Nien patients had hepatic
biochemostry disturbances. There were thrombocytopenya. The diagnosis was
established by: ultrasonography in 33 cases (all informed as hepatic mass), CT 36
cases (9 mass effect,and 27 angioma), gammagraphy 1,9 cases (16 angioma diagnosed
and 3 mass effect), arteriography in 19 cases (14 diagnosed and 5 not diagnosed) and
RM 2 cases with HA posttive diagnosis. In 37/45 (82.2%) the diagnosis of HA was
achieved preoperatively. Four patients were operated on: 2 difffuse HA, 2 <3 cm and
medical contraindication. Within the 41 patients operated on, in 39 the incision was
subcostal and SUML in 1. HA localization: 21 cases was located in the right hepatic
lobe, I0 cases in left hepatic lobe and 14 cases in both lobes. The surgical treatment
was: tumorectomy (T) of HA 10, typicai hepatic resection (THR) 9, T+THR 4 and
atypical hepatic resection (AHR) 12. In6 cases the HA were not treated: 2
angiomatosis, 2 associated to carcinoma, for observation and in case the left
hepatic artery was embolized due to the large size ofthe HA. The median loss of
blood was 463,6 cc (0-3.000 c). There was no mortality and the morbidity consisted
of3 right subphrenic colections and pleural effusion.ARer a median follow-up of2,6
years (6 months-7 years) recurrence have been not detected in the resected cases.
Conclusions:
Except in small size angiomas and diffused angiomas, the remaining cases of
symptomatology appearsmust be operated on. At the presentthe HA could be resected
without loss of blood. We recommend T as first choice procedure, if possible, or
failing that the economical AHR.
NECROHEMORRHAGIC ACUTE PANCREATITIS (NHAP)
A SUNJECT TO BE RETAKEN
A. Lopez Labrador, R. Gomez, P. Rico, A. Gonzalez Chamorro, M. AbradelO, M.
Manzanera, E. Moreno Gonzalez.
The treatment of NHAP is still being in controversy, since the mortality was not
improved in the last few years. We present 39 patients with this disease treated in our
Institution.
Between 1973 and 1994, 39 patients with NHAP vere operated on. Twenty-one
patients were males and 18 females, with a median age of 54 years (range 20-84). Of
these, 26 patients (66.6%) were lithiasic, 12 (30.7%) alcoholic and traumatic.
Twenty-three patients had infection and abscess formation. Six patients underwent
partial pancreatic resection. In the remaining: debridgement and abdominal cavity
lavage, leaving in 28 patients a tube for abdominal lavage. In patients laparostomy
was left; 7 patients required reoperation.
The overall mortality was 41% (16 patients) with a median age of 60 years.
Of 16 death patients, 9 patients died because of persistent sepsis; 3 died due to
multiorganie failure, 2 because of respiratory distress, sudden death and ACVA
(Strok).
Pancreatectomy: death, 4 pancreatic fistula of a long evolution.
Laparostomy: death. Prolonged postoperative Hospital stay.
Abdominal lavage and closure: 28 patients, 14 patients died.
There has been found no ideal surgical treatment for the management of NHAP.
The pancreatic resections and laparostomies present a very high mortality; although
in our serie the mortality is lower. The surgical treatment must be individualized
depending on intraoperative findings and must be directedto the control ofthe sepsis,
which is the main cause of death in patients with NHAP.
P257 P258
TIMING O1 SURGICAL TREATMENT FOR ACUTE
BILIARY PANCREATITIS
Y_fSI].LK, V.ANDRIUSHCHENKO, A.BARWINSKA
DEPARTMENT of SURGERY, LVIV MEDICAL
INSTITUTE, LVIV, UKRAINE
The intraoperative morphological changes and immediate
postoperative outcome in 47 patients were retrospectively
studied, Patients age ranged from 22 to 89 yers, most
were females (80,9%). Surgical treatment included
cholecystectomy, corrective biliary surgery and
debridement of pancreatic or retroperi toneal
necrosisTabscesses, The eveluation of morphological
changes and mortality rate ws performed in correlation
with duration of onset of pancreatitis. Patients were
assigned toone of 4 groups: I (n-10/21,3%) underwent
immediate surgery within 48 hours of disease; II
(21/44,7%) early operations within 3-10 days; III
(12/25,5%) delayed surgery on 11-15 days after partly
recovery from the acute attact; IV (4/8,5%) elective
operations about 3-4 weeks after pancreatitis had
subsided. The most aggressive lesions were in the patients
of Group-I: local/diffuse fat necrosis of pancreatic tissue
in 70% cases, oedema of retroperitoneal area (50%),
destructive cholecystitis (50%), while only one patient had
impacted ampullary stone. Furthermore, the decreasing
morbidity of necrotizing pancreatitis with no stones
impaction in Group II-III was noted. The highest
mortality rates were in Group (40%) due to MOF in
patients aged 6'/-89 years and in Group III (16,7%) in
consequence of complications of necrotizing pancreatitis
(duodenal fistula- 1, arrosive intraabdominal bleedin.g-
1). Overall postoperative mortality was 14,9%. We
concluded that the optimal surgical strategy acute biliary
pancreatitis must include initial intensive medical therapy,
exposure treatment and elective biliary surgery after the
pancreatitis has subsided.
CYSTIC AND PAPILLARY EPITHELIAL NEOPLASM OF THE
PANCREAS: A STUDY OF 5 CASES
M.C.C. Machado, J.E.M. Cunha, T. Bacchella, J. Jukemura, S. Penteado,
M.A.C. Machado.
Department of Surgery University of Slo Paulo, Brazil.
Since the first description by Frantz in 1959, there are few reports
about this pancreatic tumor. They constitute rare tumors with prevalence
in young women and, although malignant, they have good prognosis with
benign evolution despite the fact that they present histologically features of
aggressive tumor. This is an important fact due to the possibility of cure if
completely resected.
We present our findings in five patients with Frantz tumor. Four
patients were women ad one was man (range, 10 to 39 years). All patients
underwent complete resection of the tumor. In three patients, invasion of
the portal vein was present and segmentary resection of this vessel was
performed. The outcome was favorable in four cases. One patient is alive
five years after the pancreatic resection. One patient was operated on 15
years before with the initial diagnosis of unresectable cancer underwent
resection but died two years later with hepatic metastases.
The majority of patients described in the international literature' comes
from Japan. There are only seven cases of Frantz tumor occurring in
males, including one of this series. The jaundice is rare even with large
tumors. Ultrasonography and computerized tomography demonstrate
encapsulated, spherical, hypervascularized and solid-cystic tumors.
The importance ofthis rare tumor stays in the fact that it can be cured,
if correctly recognized, with total resection even in the presence of
invasion ofvascular structures.
135
P259 P260
DIAGNOSIS AND SURGICAL TREATMENT OF
INSULINOMA STUDY OF 57 CASES.
M. C.(. Machado, J.E. Monteiro da Cunha, J. Jukernura, T.
Bacchella, S. Penteado, E.E. Abdo, A.L. Montagnini.
Department of Gastroenterology. Sio Paulo, Brasil.
BACKROUNDS: Despite.its low incidence insulinoma is the most
frequent endocdnal tumors of the pancreas. Once the diagnosis
has been stablished by demonstrating hypoglycaemia with
inappropriate hypednsulinemia dudng prolonged fasting the
problem is correct localization of the lesion. The aim of this study is
to present our experience with the treatment of 57 consecutive
cases of insulinoma.
PATIENT; AND METHODS: 57 patients were referred with clinical
diagnosis of insulinoma. There were 36 women and 21 men
(average age 36,7 years). All the patients had neurological
symptoms.The percentage of tumors preoperatively identified was
27% by CT, 30% by U.S. 54,1% by artedography and 94% by
portography and venous sampling.
RE;U.LT;: All patients were succesfully treated by operations. In
only one patient there was a negative pdmary exploration following
by positive reexploration with intraoperative US. In 9 cases there
were multiple tumors (2-10). We found 53 benign lesions and 4
malignant tumors. MEN were found in 5 cases. The operative
procedures were enucleation in 29 tumors, distal pancreatectomy
with splenectomy in 28 tumors, 4' distal pancreatectomies with
spleen preservation; pylorus preserving pancreactectomy in 3
cases benign). There was no operative death.Two patients with
malignant diseases died before 2 years with recurrence.
C0NCLUSlONS: Preoperative localization of insulinoma is not
essential for the surgical treatment. Intraoperative US is useful in
dificult cases. Enucleation is the treatment of choice when
possible. Distal resections should be performed with spleen
preservation.
CYSTIC TUMORS OF THE PANCREAS MISTAKEN FOR
PANCREATIC PSEUDOCYSTS: STUDY OF SEVEN CASES.
M.A.C. Machado*, G. Spiliopoulos*, P. Volpe**, A.L. Montagnini**, C.
Stasik*, T. Bacchella**, J.E.M. Cunha**, J.P. Campion*, M.C.C.
Machado**, B. Launois*.
Department of Surgery and Transplantation Unit.
*-University ofRennes, France. **- University ofSlo Paulo, Brazil.
The majority ofcystic lesions ofthe pancreas are pseudocysts. A small
fraction of pancreatic cysts are neoplastic rather than inflammatory in
origin. Nine to thirteen per cent of pancreatic cysts are neoplastic, benign
or malignant. Failure to recognize the neoplastic origin ofa neoplastic cyst
will lead to an improper management.
The authors present 7 cases of cystic tumors mistaken for pancreatic
pseudocysts, including mucinous cystadenoma and 4 mucinous
cystadenocarcinoma. There were 5 women and 2 men with mean age of 46
years (range 21-71). Four were drained by cystjejunostomy and three by
cystgastrostomy. Two patients with no metastases at first operation, had
metastatic spread at reoperation. One patient underwent resection but.
presented peritoneal recurrence few months after surgery. In the other four
cases, subsequent resection was possible and probably curative. Four
patients, with mucinous cystadenoma and with cystadenocarcinoma,
are still alive without evidence ofdisease with follow- up from to 6 years.
Pancreatic pseudocysts present clinical, radiological and surgical
characteristics that may help differentiate them from. cystic neoplasms.
Beware of intraoperative biopsy that shows no epithelial lining and
attention to recurrent pseudocyst. All pancreatic cyst that do not disappear
after intervention should be considered as a cystic tumor. They should be
treated by << en bloc >> resection, including cystoenteric drainage if present.
Confusion of these entities should not occur, but errors can often be
corrected.
P261 P262
SURGICAL TREATMENT OF PANCREATIC TRAUMA
M.A.C. Machado, P. Volpe, A.L. Souza, R.S. Poggetti, P.D. Branco, D.
Birolini.
Department of Surgery University of Slo Paulo Brazil.
With the aim of aiding the accurate diagnosis and treatment ofpatients
with pancreatic injuries, we reviewed the medical records of sixty-five
patients, treated for traumatic pancreatic lesions in the 5-year period from
1989 through 1993.
Records, including operative and pathology reports, were reviewed to
study the location of the pancreatic injury, associated intra-abdominal
injuries, type of injury, trauma scores, treatment, complications and
mortality rates.
There were 58 male and seven female patients witha mean age of 28.3
years (range, 2-77 years). Of the 65 pancreatic injuries, 45 (69.2%)were
caused by .penetrating wounds and twenty by blunt trauma. The most
frequent site of lesion was the head of the pancreas (38.5%). Associated
injuries were found in all but five of the patients. In the 65 patients, 170
intra-abdominal injuries were found or 2.6 per patient. Twenty-eight ofthe
65 patients (43.1%) had liver lacerations. Lacerations of major abdominal
vessels (27 patients), gastric lacerations (25 patients) and colorectal
lacerations (17 patients) were the next most commonly seen injuries.
Fifteen of the twenty deceased patients died within two days after the
accident of severe concomitant injuries. Simple drainage were performed
in 33 patients, distal pancreatectomy in 17 and duodeno-pancreatectomy in
six patients. Pancreas-related complications occurred in 20 (30.7%) of 57
patients who survived the initial operation.
We concluded that the type ofrepair employed in our series was related
to the class of injury and clinical conditions (based on trauma scores).
Therefore, whenever possible, conservative management (no pancreatic
resection) was employed in patients sustained class and II injuries and
pancreatic resection in class III and IV injuries.
SEQUENTIAL ENDOLAPAROSCOPIC APPROACH OR
TRADITIONAL CHOLECYSTECTOMY FOR ACUTE
PANCREATITIS
Manjanaro T., Cogliandolo A., Pidoto R.R., Gioffr M.A., Micali B.
General Surgery, University ofMessina, ITALY
Only few studies show the efficacy of sequential endolaparoscopic (SEL)
approach in the treatment ofmild/moderate acute biliary pancreatitis (ABP').
The aim of this study was to compare the results with SEL or laparotomic
approach ofmild/moderate ABP.
METHODS
The last 24 consecutive patients hospitalized for mild/moderate ABP and
treated with endoscopic sphyncterotomy (E?" within 48 hours from the
admission, were considered in the study. Twelve of them, 5 males and 7
females (mean age of 52.7-4.1 years) were operated on laparoscopic
cholecystectomy (LC) and 12 (5 males and 7 females, mean age of 42.8.2.4
years) underwent traditional cholecystectomy (TC). The incidence of
complications and the mean postoperative hospitalization time were compared
with the chi-square and the t-test respectively.
RESULTS
No mortality was observe. ES was successfully completed in all patients of
each Group. without complications. LC or TC were performl 6.5=-0.6 and
7.2.7 days respectively after ES. In the TC Group there were 2/12 (16.6%)
gastrointestinal blecdings, who requires blood transfusion. No major
complications were observed in the LC Group (p=NS). The incidence of
parietal complications was 2/12 (16.6%) in the LC Group and 3/12 (24.9%)
patients in the TC Group (p=NS). Postoperative stay was of4.1-0.3 days in LC
and 8.9--0.7 days in the TC Group (p<0.001).
CONCLUSIONS
SEL approach is safe, effective and less expensive than traditional approach to
remove gallbladder lithiasis after mild/moderate ABP which requires a lesser
hospitalization time.
136
P263 P264
FACTORS INFLUENCING SURVIVAL AFTER PANCREATODUOD
FOR PANCREATIC CANCER. OUR EXPERIENCE WITH 50 CASES.
Marrano D., Casadei R., Greco V.M., Minni F., Okoro H.U.
ist Surgical Clinic, University of Bologna, BolognItaly
According to literature review index of resecability of
pancreatic cancer is very low (5-37%); five-year survi-
val after pancreatic resection of pancreatic carcinoma
is 5 to 20%. In our experience five-year survival is 16%
with a median survival of 22 months. A retrospective stu-
dy of 50 cases of pancreatic carcinoma underwent to ra-
dical pancreatoduodenectomy (R0)(II with pylorus preser-
vation) was performed to verify the existence of factors
influencing the prognosis. Following factors were consi-
dered: age (>65, <65 yrs), sex, type of resection (with
or without pylorus preservation), tumour size (>3 cm. o
<3 cm.), grade of differentiation (poor, moderate, well),
invasion of peripancreatic structures (nerve, duodenum,
choledocus) and, finally, lymph node involvement. Patient
survival (in day)was considered and all parameters were
studied with statistical tests (chi square). Age, sex,
type of resection aren't factors influencing survival in
anyway; tumor size isn't statistically significant but
for T < 3 cm. median survival is higher than T > 3 cm.
(636 vs 359 days); grade of differentiation shows a me-
dian survival higher for well and moderate differentia-
ted cancer with respect on poor (514 e 436 vs 394 days);
also invasion of peripancreatic structures, particularly
duodenum, isn't statistically significant but if there
isn't invasion median survival is better (605 vs 365days)
Finally the only parameters that is able to improve pro-
gnosis is lymph nodes involvement: infact if there isn't
lymph nodes involvement (NO) median survival is 603 days
and five-year survival is 22.5% while with lymph nodes
involvement (NI) median survival is 278 days and five-
year is 0% (P < 0.05). In conclusion the only factor
that could improve survival after pancreatoduodenectomy
for pancreatic cancer is lymph nodes involvement (P<0.05).
Tumor size, differentiation and no invasion of peripan-
creatic structures could determine a better median sur-
vival but aren't statistically significant.
WHEN THE COLECTOMY IS NECESSARY IN THE SEVERE ACUTE
PANCREATITIS ?
Martins Jr, E. Crema, J.J.R. da Rocha, O. Ftres, J.I. de Andrade
Deparlment of Surgery, Faculty of Medicine of Tri,qngulo Mineiro, Uberaba
(MG) and Deparent of Surgery, Faculty of Medicine of Ribeiro Preto,
Ribeir Preto (SP) Brazil
It has been estimated that percent of patients with acute pancreatitis develop
some kind of colonic complication. The colonic involvement includes
mechanical obstruction from either extrinsic compression, fistula formation,
colonic necrosis, colonic perfuration and fisuflization of the pseudocyst into the
colon. This paper reports 5 cases ofthe severe acute pancreatitis in which cases
the colcctomy was done because of the involvement of the colon in the
inflammatory mass.
PATIENTS AND METHODS
High alcohol intake was thought to be the ause ofpanatitis in patients (all
men) and the gallstones were the aetiological factor in two patients (all women).
The overall median age was 40 years. All patients were treated in the intensive
mre unit for 9,5 days in median. The oleomy was perforned due to a
ishaemic perfuration the colon (2 cases), blockaded perfuration the olon (1
ke), fistulization to the wound (l ce) and the neoity ofthe debridment (1
case). The subtotal colectomy with primary ileotransversostomy was performed
in and in 4 patients the ileostomy and the mucosal fistula of the sigmoid
were done. The median length ofhospital was 48,5 days. One patient died in the
26 postoperative day due a cerebro accident. Four patients survives, one
with endon and exocin pancreatic insufficiency.
DISCUSSION
Colonic involvement in the acute panereatitis aggravate the abdominal and
sistemie situation. The colonic disorder seems to be due a direct action of the
pancreatic enzymes in the serosa of the colon and fat mesocolon. Secondly,
thrombosis of mesenteric and submucosal vessels may lead to infarction of the
mucosa and deeper layers and the possibility of associated fat infarction and
necrosis. The surgical management of patients with colonic involvement in
severe acute panereatitis remains difficult. The perfuration and the necrosis,
obviously, demand the colonic ressection. However, sometimes the decision
about the colectomy is subjective, and based on surgical experience, mainly in
cases where there is no evidence of necrosis, but with severe inflammatory
disorder ofthe colon and mesocolon. In conclusion, the surgeon may be prepared
for resection colonic in severe acute pancreatitis.
P265 P266
CLINICAL ROLE OF COLOR DOPPLER ULTRASOUND IN
DIAGNOSING PANCREATIC ENDOCRINE TUMORS
Y. Maruyama, W. Kimura, T. Muto
First Department of Surgery, University of Tokyo, Tokyo, Japan
We evaluated the diagnostic significance of blood flow pattern in
pancreatic endocrine tumors detected by color Doppler
ultrasonography.
Four patient with pancreatic endocrine tumors were studied with
ultrasonographic apparatus equipped with color Doppler system
(Toshiba SSA-270A, 3.75MHz sector or convex scanner). One
patient with gastrinoma, patient with non-functioning islet cell
tumor, and 2 patients with insulinoma metastasis to the liver were
studied. We obtained color expression in the tumors and pulsatile
or continuous waves in the FFF analysis. These results were
compared with those on conventional x-ray angiograms and CT
scans. Operation were performed in two patients with gastrinoma
and non-functioning islet cell tumor. Two
patients with metastatic insulinoma were studied repeatedly after
chemotherapy
Ultrasonography was able to detect all cases, but could not
sufficiently evaluate the internal echo. Color Doppler ultra-
sonography was able to reveal a significant amount of the internal
blood flow in all cases ,and to reveal anatomical relations between
the major vessels and tumors. Color Doppler ultrasonography
was reflected in hypervascular findings on angiogram and CT
scan, and proliferating vascular findings on histology.
In conclusion, color Doppler ultrasonography is a useful for not
only diagnosing the location of the pancreatic endocrine tumors,
but also evaluating the blood flow within the tumors.
Therefore, we expect that color Doppler will be useful in clinical
application for diagnosing pancreatic tumors.
INDICATION TO SURGERY IN ACUTE PANCREATITIS
F. Meduri, L. Losacco, F. Diana, F. Zangrandi, L. Pulzato, G.E. Gerunda,
A.Maffei Faccioli
Department of Surgery, Padua University
During the last fifteen years we observed 73 cases of acute pancreatitis; in
accordance with the classification of Atlanta (1992) 31 were mild acute
pancreatitis (MAP) and 42 were severe acute pancreatitis (SAP). We
observed the following complications of SAP: 14 fluid pancreatic
collection (FPC), 5 pancreatic necrosis (PN), 12 pseudocysts, 6 pancreatic
abscesses (PA) and 5 different complications haemorrhage, MOF, etc.
The overall mortality of SAP was 12% and in particular it was: 7% in FPC,
60% in PN, 0% in PC, 40% in PA. Concerning therapy, the approach was
the subsequent: among the 14 FPC, 7 were submitted to surgical drainage
1 pt died: p.o. mortality 14% ), 2 to percutaneous CT-guided aspiration
and 5 to pharmacologic therapy; all 5 PN underwent surgery, with 60%
of p.o. mortality; among 12 Pc, 6 were drained by surgery, was
submitted to a percutaneous aspiration and 5 spontaneously regressed;-
finally, among 6 PA, 5 underwent surgery (p.o. mortality 40% and was
submitted to percutaneous aspiration. SAP complications require a rational
therapeutic approach: aseptic complications need only a careful
observation and the indications for surgery are represented by the risk of
infection, increasing size and haemodinamic failure; pseudocysts need
surgery only if voluminous, progressively growing, filled up by Wirsung
duct, or if complicated, while percutaneous aspiration is suitable only for
unloculated, stable and easily accessible Pc or in low compliance pts;
finally, small and stable PC need no treatment; infective complications
always require urgent surgical management, only selected pts with a high
operative risk may be submitted to a temporary percutaneous CT-guided
aspiration.
137
P267 P268
FAT MALABSORPTION FOLLOWING PYLORUS-PRESERVING
PANCREATODUODENECTOMY
Shuichi Miyakawa, Makoto Hayakawa, Akihiko
Hodguchi, Shin Ishihara, Tunekazu Hanai, Naotatu
Niwamoto, Tadashi Satoh, Yuji Iwase, Kaoru Miura.
Dept. of Surgery, Fujita Health University, Toyoake,
Japan.
The pylorus-preserving pancreatoduodenectomy (PPPD)
affords excellent nutritional health in comparison with
the standard Whipple procedure (SPD). But, maldigestion
and malnutrition are observed in some of patients who
underwent PPPD. The superiority of PPPD should be
demonstrated between the patients with the same
pancreatic exocrine function. We suposed that the
pancreatic exocrine function following operation is
provided for the fibrosis of the pancreatic remnant, and
studied the fat malabsorption following operation using
13C- trioctanoin breath test between the groups classified
by the fibrosis of the pancreatic remnant. We presumed
the area of fibrosis in the pancreatic remnant by
measuring fibrosis of the resected caudate wege
histologically. We tested to 11 SPD cases and 25 PPPD
cases. The 13C excretion rates and the cumulative value
following PPPD were significantly better than those
following SPD. The 13C excretion rates and the
cumulative value in the patient.s with more than 30 96
pancreatic fibrosis were significantly lower than those
in the patients with less than 30 96 pancreatic fibrosis,
regardless of the surgical procedures. But, the
cumulative values following SPD group was significantly
lower than that following PPPD or DPPHR in the patients
with less than 30% pancreatic fibrosis. The results
suggested that fat absorption following PPPD is superior
to that following SPD in the patients with the same
fibrotic area of the pancreatic remnant, and is provided
for the fibrotic area of the pancreatic remnant.
OMEPRAZOLE PRE ADMINISTRATION TO RATS
DECREASES HISTOPATHOLOGYCAL ALTERATIONS
OF CERULEIN INDUCED PANCREATITIS
AL Monta_mfini, KRM Leite, MS Kubrusly, P Herman, AMM
Coelho, NAT Molan, MCC Machado, HW Pinotti
Dept. OfGastroenterology Sto Paulo University Medical School
BRAZIL
Pancreatic content of digestive, enzymes play an importantwhole on
the intensity of acute panereatitis (AP). We have recently shown
that omeprazole (OMPZ) administration to rats lowers the amount
of trypsin on pancreatic tissue and decreases tissue water content
and serum amylase after induction of AP. This study was carded
out to determine if OMPZ administration have any influence on
eerulein induced AP hystologieal findings. Twenty five male Wistar
rats were divided in four groups: G Control- received saline
solution and no cerulein, G II OMPZ Control received OMPZ 5
mol/Kg for 3 days) and no. cerulein, G III Acute Panereatitis.
saline solution pre treatment and eerulein 40 g/Kg), and G IV
OMPZ/AP OMPZ pre treatment and cerulein. Four hours after
AP induction all animals were killed and pancreas removed for
histologycal examination. Results:
EDEMA ACINAR ]N'FLAMMAT. HEMORRHAGE
NECROSIS INFILTRATE
G 0,8 0 1,5 0
GII 1,1 0 0,4 0
G ltl 2,8* 1,0" 2,8* 0,1
G IV 2,0* 0,2* 1,2" 0,1
p< 0,05
Conclusion: Omeprazole treated group presented less intense
pancreatic histologycal findings. We believe that this effect is
mediated by changes in pancreatic enzyme contents induced by
omeprazole administration.
P269 P270
INFLUENCE OF CIRCULATING PHOSPHOLIPASE A2 ON
ARACHIDONIC ACID CASCADE
M. Motovoshi. W. Kimura, T. Muto
Department of Surgery, University of Tokyo, Tokyo, Japan
In order to clarify the role ofphospholipase A2 in the pathogenesis of
multiple organ failure in severe aftlte pancreatitis, we investigated about the
influence of circulating phospholipase A2 on arachidonic acid cascade. Guinea
pigs weighing 600-800 g were anesthetized by pentobarbita135mg/kg i.p.,
andjugular vein and carotid artery were cannulated. After 12 hours for
recovery from preparation, guinea pigs were divided into 3 groups, and
phospholipase A2 extracted and purified from porcine pancreas was injected at
0, 20, and 50U/kg/min respectively with normal saline 0.lml/kg/min for
30min. Then blood was sampled and serum phospholipase A2 activity, serum
phospholipid concentration and composition, plasma arachidonie acid
concentration and distribution, and plasma eicosanoids concentration were
determined. As a result, intravenous infusiou ofphospholipase A2 decreased
serum phosphatidylcholine and phosphatidylethanolamine concentration,
increased serum lysophosphatidylcholine and non-esterized fatty acid
concentration, decreased plasmaphospholipid-bound arachidonic acid
concentration, increased plasmafree arachidonic acid concentrafon, decreased
total plasma.arachidonicacid concentration, and iucreased plasma
concentration of prostaglandin E2, prostaglandin Fltx, thromboxane B2, and
leukotriene B4. The result suggests that phospholipase A2 in circulating
blood degradates phospholipid in plasmalipoprotein to liberate arachidonic
acid into plasma, which is uptaken intracellulary and converted to eicosanoids.
Excessively produced eicosanoids may cause systemic microcirculation
disorder, which may contribute to development ofmultiple organ failure.
ALTERNATIVE APPROACH TO MANAGEMENT OF THE PANCREATIC
STUMP AFTER PANCREATODUODENAL RESECTION
A.G.Mylnik0v
A.V.Vishnevsky Institute of Surgery, Moscow, Russia
Our experience in pancreatoduodenal rsection (PDR) comprises 232 operations.
The main of postoperative morbidity and mortality has been the
complicated of the pancreatic stump. We believe that the to
in PDR is alternative approach to pancreatic stump treatment based
following criteria: morphological changes in the remaining pancreatic parenchima
and diameter of the main pancreatic duct(MPD). For this aim have
distinguished types of pancreatic stump. Type is characterised by hard,
fibrous-changing pancreatic parenchima 'and dilatation of the MPD of
than in diameter type is defined in of rather firm-parenchima and
moderate MPD extention (3-6 mm}. In patients with these two types of
pancreatic remnant the most favourable results obtained after longitudinal
pancreatojejunostomy (PJS) used in 21 anastomotic leakage not seen;
the but somewhat noted after terminolateral PJS (used in
59): (10,3%) anastomotic leakages without mortality. Finally, type
is determinated in patients with smooth, non-altering parenchima and with
diameter of the MPD less than mm; in 38 patients with such adversible
pancreatic remnant the content attained when the "occlusive" PJS, wich
combined pancreatic duct occlusion with terminolateral PJS, performed:
morbidity rate 19%, mortality rate 6,1%. The considerably results
received when other methods of pancreatic stump management have been used
(such simple occlusion, external drainage ligation of the MPD) these
three above-mentioned ways but without concordance with pancreatic stump
morphology.
138
P271 P272
SURGICAL MANAGEMENT OF MUCIN-PRODUCING PANCREAS TUMORS AND A
NEW APPROACH: RESECTION OF THE INFERIOR HEAD OF THE PANCREAS
T.Nakagohri, T.Asano, W.Takayama, T.Uematsu, T.Kenmochi, K.Isono
Department of Surgery, Chiba University, School of Medicine
Surgical results of 24 cosecutive cases of mucin-producing pancreas tumors were
studied. And we present a new approach for the partial resection of the inferior
head of the pancreas.
Patients
24 cases of mucin-producing pancreas tumors have been surgically treated in
Chiba University hospital since 1979. They were classified into three groups,
mucinous cystic tumors ductectatic type), mucinous cystic tumors (megacystic
type), and intraductal papillary tumors, according to the continuity between the
cyst and the pancreatic duct and the location of the papillary tumors. There were
16 patients with ductectatic type, 4 patients with megacystic type, and 4 patients
with intraductal type.
Results
The mean age of patients with ductectatic type, megacystic type,intraductal type
were 61,54, 62 respectively. 15 lesions (62.5%) were located in the head of the
pancreas, 9 lesions (37.5%) were in the body and tail of the pancreas. 13 lesions
(81.3%) of the ductectatic type were located in the head of the pancreas,
meanwhile 4 iesions (100%) of the intrductal type were located in the body of the
pancreas. The mean size of ductectatic tumors, megacystic tumors and
intraductal tumors were 2.6 cm, 8.9 cm, 1.4 cm respectively. As for 15 patients
with tumors in the head of the pancreas, 7 patients underwent Whipples PD, four
patients underwent PPPD, one patient received duodenum-preserving resection of
the head of the pancreas. For the rest three patients with ductectatic cystic
tumors, we performed resections of the inferior head of the pancreas. This
procedure preserved the duodenum, the common bile duct and the upper part of the
head of the pancreas around the duct of Santorini. Distal pancreatectomies were
performed in 7 cases with the lesions in the body and tail of the pancreas. Two
patients underwent segmental resections of the body of the pancreas. Histological
examination revealed that 5 tumors well differentiated papillary adenocarcinomas)
invaded into the pancreas parenchyma, and only one of them invaded into the
adjacent organs. Lymph node in volvement was not observed in our 24 cases.
Only one patient died of recurrent disease ten months after the operation.
Conclusion
Muin-producing pancreas tumors have good prognosis after surgical treatment.
We believe resection of the inferior head of the pancreas has a significant role to
play in the management of patients with mucin- producing pancreas tumors
Near-infrared spectrometry for fecal fat analysis in
patients with pancreatic steatorrhea
T. Nakamura, A. Terada, Y. Arai, N. Yamada, Y.
Tandoh, K. Imamura, H. Kikuchi, T. Suda
3rd Department of internal Medicine, Hirosaki University
School of Medicine, Aomori, Japan
This study was aimed at corhparing a new method for measuring
fecal fat excretion, assayed by near-infrared spectrometry (NIRS),
with Van de Kamer and gas-chromatographic (GLC) method.
We used an Infra Alyzer450 (Bran-Luebbe, K.K. Japan). Near-
infrared analysis of fecal fat was assayed on the three-day stool
collection from 70 patients (40 chronic pancreatitis, 20
gastrointestinal disorders, and 10 diabetes mellitus).
Hepatadecanoic acid and 23-nordeoxycholic acid were added to
freeze-dried feces as an internal standard, and the sample was
hydrolysed in an autoclave. The mixture was then butyrated,
acetylated and analized by gas liquid chromatography. The fatty
acid and neutral sterols were eluted and bile acids were then
separated.
A close linear correlation was found between NIPS and both Van
de Kamer (p<0.01) and GLC (P<0.01) methods on the fecal fat.
But A weak correlation was found between NIRS and GLC method
on the fecal bile acid.
The measurement of fecal fat excretion by the near-infrared
spectrometry is a rapid, simple and useful method for measuring
and monitoring steatrrhea (especially pancreatic steatorrhea).
P273 P274
A SCORING SYSTEM BASED ON NEW HISTOLOGIC
PARAMETERS FOR PREDICTING OUTCOME IN CASES OF
DUCTAL ADENOCARCINOMA OF THE PANCREATIC HEAD
T.Nakatsura,* T.Hasebe,** M.Ryu,* T.Kinoshita,* N.kawano,*
M.Konishi,* Y.Tsubono, T.Kosuge,**** K.Mukai**
*Surgery Division, National Cancer Center Hospital East, Kashiwa,
**Pathology Di vision, and***Epidemiology and Biostati stics Division,
National Cancer Center Research Institute East, Kashiwa, and
****Surgery Division, National Cancer Center Hospital, Tokyo, Japan
Although many reports have described prognostic factors in pancreatic
ductal adenocarcinoma, the best parameters for predicting treatment
results have not been established. Many surgically resected cases are
UICC Stage III, a poor prognostic category, but some patients survive
for a long time. To define caractedstic of cases with good clinical
outcome, we investigated which histologic parameters are best for
predicting survival in patients with ductal adenocarcinoma of the
pancreatic head. Materials and Methods: Twenty five cases of curatively
resected Stage III carcinoma of the pancreatic head were examined. In
addition to parameters previously recognized for ductal adenocarcinoma
of the pancreas, three additional histologic parameters were investigate(
1) fibrotic focus (FF), 2) direct invasion of the tumor into the resional
lymph nodes (DILNT), and 3) tumor necrosis (TN). Results: Tumor
recurrence and tumor death were more fi:equent in patients having
adenocarcinoma with FF, DILNT, TN, or lymphatic permeation (ly).
Of the three parameters investigated in this study, FF was significantly
associated with tumor recurrence and tumor death. A scoring system
was devised on the basis of the three factors and ly. The absence or
presence of FF, DILNT, TN or ly was given a score of 0 or 1, and a
total score was calculated. The total score was linearly correlated with
survival. Patients with a score of 4 survived for a significantly shorter
time than other patients. Conclusion: The results show a clear
prognostic significance of the presence of FF, DILNT, and TN in ductal
adenocarcinoma of the pancreatic head and suggest the scoring system is
usoful for classification of Stage III ductal adenocarcinoma of the
pancreatic head.
ULTRASOUND GUIDED TREATMENT OF HEPATIC
AND PERIHEPATIC ABSCESSES
Zs. N6meth, T. Winternitz, P. Kupcsulik, L. Flautner
1st Dept. Surgery, Semmelweis Med. Univ. Budapest,
Hungary
The ultrasound guided puncture and drainage ofhepatic and perihepatic
abscesses became to be a routine procedure at the last decade.. Since
1989 we performed 49 such interventions. The risk of that type of
intervention is significantly lower hence the lethality is much lower and
the effectiveness is the same compared to the surgical intervention. This
treatment should be especially chosen if the patients are at high risk.
Part of our patients had biliary obstruction caused by pancreatic
malignancy, others had postoperative liver or perihepatic abscess.
Criteria for ultrasound guided puncture were: good visibility of the
abscess, no intersection of gut or other organ, good cooperation of the
patients.
A Picker LS 5000 and a HITACHI EUB 565A ultrasound apparatus
was used with 3,5 MHz biopsy transducer type. The puncture was
performed with a 20-22 Gauge disposable Chiba needle. Ifthe puncture
was successful a bacteriologic sample was withdrawn. And a 7-12 Fr
trocar drain was introduced. The controlled suction was applied using a
vacuum bottle. The drain was irrigated twice a day. The clinical status
and the ultrasound picture were controlled every day.
RESULTS: 39 of 49 patients were free of any symptoms after an
average drainage time of days. 16 patients needed repeated drainage as
the drain slide out incidentally. 2 patients died because of the
underlying malignant disease but without symptoms of the hepatic
abscess. 3 patients needed 2 simultaneous drains for a single abscess, at
patients the treatment was unsuccessful, and the patients underwent
an operation. According to our experience the percutaneous ultrasound
guided drainage of the intra-abdominal abscesses is a useful, low risk
method in the treatment ofpatients with no other indication for surgical
intervention.
139
P275 P276
THE LONGTERM BENEFIT OF LAPAROSCOPIC STAGING FOR
PATIENTS WITHHEPATOPANCREATOBILIARY MALIGNANCIES.
E.J.M.Nieveen van Di_ikurn, L.Th.de Wit, H.Obertop, D.J.Gouma. Department
of Surgery, Academic Medical Center, Amsterdam, The Netherlands.
Diagnostic laparoseopy combined with laparoscopic ultrasonography is increasingly
used for staging hepatopancreatobiliary (HPB)-malignancies.
The effect oflaparoscopic staging can be expressed in change oftumorstage and
number ofdirectly prevented laparotomies. However, not only early prevention of
laparotomies but in particular adequate longterm prevention oflaparotomies must be
an important effect oflaparoscopic staging. Therefore the number oflate
laparotomies was assessed in this retrospective study.
Patients and Methods: Between January 1992 and July 1995, 171 patients with
assumed resectable HPB-tumors after conventional preoperative staging, underwent
diagnostic laparoscopy and laparoscopie ultrasonography. Included were patients
with distal (pancreatic head n--100/papillary n 14)and proximal bile duct
tumors(n=26), livertumors(n=24) and pancreatic body/tail tumors(n=7). The records
ofall patients were analysed for surgical/non-surgical treatment.
Results: Six patients (1 proximal tumor/3 distal tumors/2 livertumors) were lost
from follow up, 165 patients couldbe evaluated. Explorative laparotomy was
performed in 122 patients (76%) and laparotomy was prevented in 43 patients
(26%); 17 patients with distal and 13 patients with proximal bile duct tumors,
10 patients with livertumors and patients with pancreatic corpus/tail tumors.
During follow up 5/17 patients (29%) with distal and 2/13 patients (15%) with
proximal bile duct tumors needed late laparotomy because ofduodenal obstruction,
after median time interval of7 months (range 3-12 months).
The effect ofstaging GI-tumors with diagnostic laparoscopy can be expressed by the
number ofdirectly prevented laparotornies, 43/165 (26%), however late laparotomies
were necessary in 7/43 patients (16%). Laparotomies are avoided, as shown by
longterm follow up, in 11/25 patients (44%) with proximal bile duct tumors, 10/22
patients (45%) with livertumors and 3/7 patients (43%) with pancreatic corpus/tail
tumors. In only 12/111 patients (11%) with distal bile duct tumors a laparotomy was
really avoided compaired to the initial 17/111 patients (15%); 5 patients developed
duodenal obstruction, which is as yet not treatable without surgery.
Conclusion: The benefit oflaparoscopic staging was obvious for patients with
proximal bile duct tumors, liver tumors and pancreatic corpus/tail tumors, but for
patients with distal bile duct tumors,late laparotomies have to be taken into account.
ACUTE PANCREATITIS (AP) TREATMENT & SEPTIC
COMPLICATION (SC).
V. Ohonovsky, M. Podilchak, A. Yavorsky
Department of General Surgery, Medical Institute, Lviv, Ukraine.
The development of septic complications (SC) in patients
with acute pancreatitis (AP) essentially worsens the prediction of the
sickness. Among 127 patients we found a severe form of AP,
according to 3.H.C.Ranson, parameters, in 27 (21.2%) patients. The
treatment tactics involved intensive conservative therapy:
antibiotics, intravenous fluid, fresh frozen plasma transfusion,
diuretics, anticholinergics, H2-receptor blockers, gastric intubation
suction, low-fat elemental diet. Patients with high enzymenia
underwent treatment by intravenous injection of 5-fluoracyl. In case
of peritonits; laparoscopy with effusion evacution and drainage or
laparotomy was done. Surgical procedures were performed on 14
(11.0%) patients. They involved billary pathology removal,
necrectomy, omentopancreatoplexy, omentum sac and abdominal
cavity drainage. Antibiotics were injected into the liver round
ligament, the abdominal cavity was irrigated by 0.02% solution of
chlorhexidine bigluconate. UV autoblood irradiation was
administered. Parapancreatitis epigastrium mass was irradiated by
local antiflammatory X-ray therapy with an accumated absorbed
dose of 1.2-1.5 Gr. SC which required furher operations developed in
3 (11.1%) patients with severe AP form. One patient died. Besides
two more patients, who were delivered from other hospitals with
already developed SC, also died. In 19 patients with severe form of
AP dynamics of natural killer cells (NKC) and lymphocyte adherence
inhibition test (LAIT) was studied. The decrease in NKC level
(4.1+/-1.1% against control indicator 7.4+/-0.7% p<0.01) and increase in
LAIT level (39.4+/-5.1% against the control value 23.0+/-3.2% p<0.05)
was found at the beginning of the illness. If the AP course was
favourable the above mentioned indicators tended to stabilize:
6.7+/-0.9% and 27.3+/-4.7% respectively, p>0.05. In patients with SC
they were on the previous levels. Thus, NKC level and LAIT level
definition may be helpful in SC prediction in patients with AP.
P277 P278
PERCUTANEOUS PANCREATIC CYSTOGASTROSTOMY
GUIDED BY ULTRASOUND SCANNING AND GASTROSCOPY
A.K.Olsen*, R.Svihus**
Department of Surgery* and Department of
Radiology**, Central Hospital of Rogaland,
Stavanger, Norway
During the years 1985-1995, 16 patients of
mean age 45 (range 9-68) years with pancreatic
pseudocyst were treated by percutaneous
cystogastrostomy, guided by ultrasound
scanning and gastroscopy. They comprised 8
men and 8 women. In 2 of the patients a
recurrent cyst occurred and 4 years after
primary intervention.
A diagnosis of chronic pancreatitis was
verified in 12 patients (75 per cent).
A double pigtail catheter (Ultrasound
Pancreatic Cyst Drainage Set Cook) was
successfully placed in 17 cysts (94 per cent).
No complications or death has occurred.
The median time to radiological resolution
was 5 days (range 1-60 days), and the median
follow up after successful treatment was 36
months (range 1-120 months).
The method described is less traumatic than
operation, and mortality and complication
rates compare favourably with those seen
after surgery and the results are at least as
good.
IS A FINE-NEEDLE ASPIRATION BIOPSY NECESSARY IN
DECISION-MAKING PROCESS BEFORE PANCREATODUODENECTOMY FOR
PANCREATIC HEAD TUMOR
Paczkowski PM, Zieniewicz K, Krawczyk M, Najnigier B,
Nyckowski P, Paluszkiewicz R, Patkowski W, Plaszczyk D.
Dept of General Surgery Liver Diseases, Medical
Academy of Warsaw, Warsaw, Poland
A retrospeive study was carried out to the value
of fine needle aspiration biopsy (FNA) in preoperative
qualification or pancreatoduodenectomy in patients with
pancreatic head tumor, diagnosed by imaging techniques
(sonography, CT-scan, ERCP, endosonography). Among 411
patients treated in our department for pancreatic
pathology between 1992 and 1995, almost half presented
with pancreatic head tumor (137- neoplasmatic, 60-
chronic pancreatitis). SiXty pancreatoduodenectomies
performed based preoperative diagnosis of cancer
in 53 patients and of chronic pancreatitis in the
remaining 7. In the former group FNA performed in 23
'cases, giving cytological diagnosis of in 14 of
them. Histological examination of the optative specimen
confirmed the diagnosis of cancer in all but patients.
in whom FNA revvealed malignancy. In the remaining
patients all operative specimens confirmed cancerous
process. In patients with preoperative diagnosis of
non-neoplasmatic tumor FNA was performed in 2, and was
negative. In all of them final histological examination
did not demonstrate cancer in the excised specimen.
Our results, well other pancreatic surgery
centers', drew to the conclusion that FNA should be
reserved for patients in whom the operative treatment is
not planned for various reasons. In those who
candidates for surgery (of any kind), the decision
should rather be based intraoperative histological
examination.
140
P279 P280
ROLE OF MAGNETIC RESONANCE IMAGING IN ACUTE
PANCREATITIS DIAGNOSIS AND STAGING
D. Parolini, .L, Giangreco, M. Carlucci, A. Zerbi, A.
Vanzulli*, A. Del Maschio*, C. Staudacher
Dept. of Emergency Surgery, Dept. of General Surgery,
* Dept. of Radiology, S. Raffaele Hospital, University of
Milan, Milan, Italy
Aim of this study was to compare Computed
Tomography (CT) and Magnetic Resonance Imaging
(MRI) diagnostic reliability in acute pancreatitis (AP).
During a 44 months period 21 patients with AP
underwent both CT (5 scans without contrast
medium) and MRI (T1 sequences) at the beginning of
the symptoms. The scans were evaluated according to:
pancteatitis degree and presence and rate of necrosis.
Pancreatitis degree was assessed using Balthazar's
grading for CT and a similar classification for MRI.
Thirteen patients (61.9%) had oedematous
pancreatitis and 8 (38.1%) necrotizing. pancreatitis.
Necrosis was diagnosed intraoperatively or with CT
scan. MRI staging was identic to CT ones in all but 2
patients (90.5% diagnostic accuracy) who resulted to
be grade E at CT and grade D at MRI. Pancreatic
necrosis was properly identified on MRI in all the 8
patients (100%) with necrotizing AP, whereas CT
resulted diagnostic only in 5 patients (62.5%), since 3
scans were performed without contrast medium
infusion bacause of acute renal failure. MRI was
proved to .be an effective alternative in AP diagnosis,
since it provides the same diagnostic and prognostic
information as CT and it does not need contrast
infusion, which makes it preferable to CT in the follow
up of severe AP evolution.
OCTREOTIDE SCINTIGRAPHY. ROLE IN DETECTION AND
STAGING OF PANCREATIC AND LIVER APUDOMAS.
C. Pasquali, C. Sperti, # N. Borsato, G. Liessi, P. Gasparoni, #
G. Ferlin, S. Pedrazzoli
Semeiotica Chirurgica, University of Padua, # Nuclear Medicine,
Radiology, Medicine, Castelfranco V. Hospital, Italy.
From October 1992 to November 1995, 27 patients with proven
diagnosis of pancreatic apudoma and/or hepatic or nodes
metastases from abdominal apudoma, and 8 patients suspected to
have an abdominal endocrine tumor, underwent Octreotide
scintigraphy. Among the 27 patients, 11 had a ZES (4 with MEN 1),
6 insulinomas, 8 Carcinoids (3 foregut and hindgut), 2 "non-
functioning"tumors (1 PPoma, Somatostatinoma). Fourteen/27
had liver metastases at time of investigation; in 3 cases were not
previously suspected. Liver metastases were detected in 13/14
cases and in 5 cases the hepatic mass was single. In case a
multiple liver mass (octreoscan negative) was found to be a HCC in
a ZES patient. Unexpected distant sites of uptake outside the
abdomen were detected in 3 patients with liver metastases and in
patient with localized disease. The Octreotide scintigraphy was
negative in 6/27 patients (5/6 insulinomas and carcinoid). In 3
cases without metastases, the pdmary was detected because of a
positive octreoscan. Seven cases had the octreoscan before and
after treatment; in 5 a consistent uptake reduction was shown after
palliative treatment and was completely negative after excision of
a metastatic node. A single false positive occurred in the 8
suspected cases of apudoma.
Octreotide scintigraphy is able both to detect the primary tumor and
unsuspected metastases in patients with abdominal apudomas,
excluding those with insulin secretion.
P281 P282
TREATMENT OF ZOLLINGER-ELLISON SYNDROME.
C. Pasquali, C. Sperti, G. Liessi, S. Pedrazzoli.
Semeiotica Chirurgica University of Padua, Radiology, Castelfranco V.
Hospital, Italy.
From 1970 to 1995, 53 patients with Zollinger-Ellison syndrome (ZES)
were observed. MEN type syndrome was found in 24.5 % of the
patients. Only 28% of the patients had no gastric surgery before the
diagnosis of ZES. Seven cases (13 %) had distant metastases at time of
diagnosis and 3 more cases developed liver metastases during the follow
up (9,15 and 21 years later). Thirtytwo patients (60%) underwent surgery;
11 had only a "palliative" gastrectomy and 21 had a laparotomy with
resective purposes. A total of 11/32 surgically treated patients (34%) had
a gastrin fall to normal after surgery (9 of them with a follow up of 5 -17
years), including one MEN case and one with liver tumor. Two of these
"cured" patients had a late recurrence 5 and 14 years later. Out of 32
patients who underwent surgery 22 % had an emergency gastrectomy 2
associated with tumor resection) for complication due to ulcer disease
and 9121 of the patients who had resective surgery had also a
gastrectomy associated. Morbidity was 34% and mortality 18% (3/6 in
emergency 5 before 1979). Twenty-seven patients initially had
antisecretory drugs therapy, but 3 escaped to H-2 blockers and needed
emergency sUrgery (after 3 mo.-3 yrs.) while 9 required omeprazole for
long term-failure 2-14 yrs.) of H-2 antagonist. Four medically treated
patients had their primary tumor detected 3, 7, 9 and 14 year after the
diagnosis; only one had than the tumor resected and became
normogastrinemic. Five out of ten patients with liver metastases survived
more of 5 years after detection of the secondary.
Long-term palliation or "cure" may be achieved by tumor resection in ZES
(34 % in our series) however life-long follow-up is mandatory for late
recurrencies (> 5 yrs.). Re-evaluation of patients without positive imagiog
is mandatory for possible reversion from medical therapy to resective
surgery.
MANAGEMENT TACTICS OF SEVERE NECROTIZING
PANCREA'ITI3S
A.Perejadov, S.Chooklin, M.Pavlovsky
Department ofSurgery, Medical Institute, Lviv, Ukraine
146 patients with necro"tin8pancreatitis were treated, 48 (32,9%) of
them were operated. Biochendal and inununologial investigations
hyperglycenda, which accompanied by the decrease ofinsulin and C-
peptide blood level, hypoproteinemia, amynotranspheras and alkaline
phosphatase ativity increasing were noted in most patients. In the
62,3% of patients increase of T-lymphocytes-supressors activity and
the separation of cells in the immune response were marked. The
anu"oactefial, des/mtoxicative, including the methods of extracorporal
desintoxicafion, immtmoregulatory therapy were performed in all
patients. Operations, which were performed in the early stages of
necro"ttzin8pancreatitis development 02 patients), were accompanied
try the big quantity of complications (41,7%) and high (50%)
mortality. The decreme of IgA, 18G and IgM concentration, cortil
blood level and T-lymphocytes in the postoperative period were
marked. If the changes were taking place on the backsund of
immuuorcgulatory cells' disbalance, in 29,5% ofpatients the different
pund.ent-septic complications were noted. Complex evaluation of
clim'cal-laboratory and ultrasonographic results allowed to approach
more strictly to term detumination and surgery indicatiom and avoid
groundless .operations. Abscesses, retr0peritoneal phlegmon signs and
free located sequester, presence were indications to postponed
operation performance. Operations performed 5-9 days after
admission, occurred in the period when relative stabilization oforgan
and system functions ensued. Performance of operations in fixed
terms allowed to decrease complications' number to 8,3% and
postative mortality to 16,7%.
141
P283 P284
ANASTOMOSIS PANCRETOJEJUNAL WITH PANCREATIC DUCTUS TO THE
JEJUNAL MUCOSA'S SUTURE VERSUS OPEN JEJUNAL DUCTUS
EXPERIMENTAL RESEARCH.
..V.,Perez Auladell, M.Morales, J.Medrano, E.Garcia, J.Agull6
Departament of Surgery,University of Alicante,Alicante,Spain
Since 1898 Alesandro de Codeville carried out in Bolognia the first pancreatic
resection,suturing the pancreatic duct,to the pancreatic surgery's modern age that
began with Whipple(1935),one of the main problems today still unsolved has been
how to trat the pancreatic remanent.
The common denominator to all these tecniques is the high incidence of pancreatic
fistulas,that reach a high mortality rate.
We present the experimental survey carried out in 12 adult dogs weighing between 15
and 25 kg.,realizing the following surgical tecnique:
Group A Pancreatic ductus to jejunal mucosa's suture (6dogs)
Group B :Open ductus pancreatic (6 dogs).
Factors of tissue harm through blood test
of:glucose,insulin,amylase,lipase,elastase,c.r.protein were analysed.
Research of chemotripsine in the stools.
Moreover,a pancreatic morphologic research is made through pathologic anatomy of
the necropsy's pieces.
Existing in the final analysis clear advantage of the group A over the B.
As conclusion,we think that this tecnique of pacreatic ductus to jejunal mucosa's
suture reduces the incidence of pancreatic fistulas,and keeps the pancreatic
endochrine and exochrine functions.
NECROTIC PANCREATITIS: CHOICE OF SURGERY
R.Petrauslden6. N.ileikien6, K.Katilius, J.Stanaitis
Vilnius univexsity emergency hospital, Lithuania
The purpose of work: to comparc mtrvival of patients ill with
pancrconeerosis (after Atlanta's classification) due to different methods
ofsurgery.
Results: 57 patients 20 female and 37 male) ill with pancrconccrosis
were operated durin8 the period of 1992-1995. The ave.rae abe was 37
for male patients and 53 for female patients. Biliary ethiology was
observed in 15 patients (26,3%), other causes in 43 (75,4%) eases.
Mortality_was 36,8%. The averase stay in hospital in case of marvival was
58 days, in case o1 death 19 days. All patients were iveu antibiotics,
intravenous infussions, spasmolythics, local cold application, nasosastrie
tube prior to operation. In 3 eases (5,2%) only lapaxoscopic dralnas of
abdominal cavity was needed. In 6 cases (10,5%) only lapaxoscopie
drainase and pereutaneous tramhepatie eholecystostomy was
performed. In 13 patients (22,8%) laparoseopy was not sufficient and
was followed with laparotomy in several days. In ease of biliary
ethiology 15 patients(26,3%) underwent laparotomy, eholecysteetomy,
drainase of common bile duct, drainage and sctonase of bursa
omentalis 7 (46,6%) of them died. In non-biliary panereoneerosis
laparotomy, eholecystostomy, drainase and setonage of bursa omentalis
was performed in 27 eases in 14 eases (51,8%) patients have died, in 6
eases was performed only laparotomy, draina8e and setonase of bursa
omentalis. Relaparotomy and neereetomy of pancreas was performed
in 14 eases (24,6%). Due to septic complications died 18 patients: 11
patients due to pancreas abscess, 7 due to the progrein8
retroperitoneal phle8mone. 3 patients died due to bleedin8.
Conclusion: there is no sisnlfieant difference in survival of patients ill
with panereoneerosis due to different tactics of sursety.
P285 P286
LONG-TERM RESULTS OF PANCREATIC AND
PERIAMPULLARY CANCER SURGERY
T.Popiela, B.K, D.Karcz, H.Labza
Ist Department of General and GI Surgery, Jagiellonian
'Jniversity, Krak6w, Poland.
The aim of the study is the analysis of long-term results
of pancreatic and periampullary cancer surgery.
Material The authors analyze 450 cases treated for
pancreatic and periampullary cancer using own
diagnostic model based on USG, ERCP, and FNAB. In
this group of patients there were performed 144
resection procedures (Whipple 79, Traverso 29, total
resections 17 subtotal 17, segmental 2). The
presentation compares own modified techniques of
duodenopancreatectomy (Whipple vs. Traverso)
assessing long-term survival following the procedures
and complication rates.
Results The rate of perioperative mortality was 8.2%
and long-term survival in pancreatic cancer was 3.4%
while in the periampullary cancer 65.2%. Obtained
results confirmed high efficiency of ERCP and FNAB in
pancreatic cancer diagnostics. It was also observed that
life comfort in patients undergoing
duodenopancreatectomy modo Traverso was better as
compared to Whipple procedures at the similar
percentage of perioperative complications.
CARCINOMA OF THE PANCREAS.
MODERN DIAGNOSIS AND SURGICAL TREATMENT
.Puchalski Z., Ladny J.R., Polak6w J.,
Rog M., Trochimowicz L., Barczyk J.
Department of General Surgery, Medical Univeristy of Bialystok, Poland
The aim of the study was the presentation of our experiences in modern
diagnosis and surgical treatment of pancreatic carcinoma.
Between 1984 and 1995 in our Department. was treated 226 patients
with carcinoma of the exocrine portion of the pancreas. There were
146 males and 80 females with a mean age of 57.4 years. In 157 cases
(69.5%) the primary seat of tumor was located in the head and in
69 cases (30.5%) in the body or tail of pancreas. The diagnosis was
determined on the basis of clinical manifestations and laboratory studies.
Very useful in the diagnosis were tumor markers (CEA, CA 19-9).
Imaging studies included an upper gastro-intestinal series,
ultrasonography (US), color Doppler sonography, CT, enhanced CT,
MRI, and US and CT guided biopsy.
Of 226 patients, 215 underwent surgery. Pancreaticoduodenectomy
(PD) i.e. Whipple,s, Child,s etc with N2 resection ofthe lymph nodes and
pylorus-preserving PD were standard operations for pancreatic head
carcinoma. Total pancreatectomy (TP) was, performed in advanced
cases of pancreatic head carcinoma, those with intrapancreatic
metastasis, and in cases in which the function of the remnant pancreas
was severely impaired. Five-year survival rates after resection of
pancreatic cancer was about 10%.
The study shows that although there has been improvement in
diagnostic methods survival rates after resection of pancreatic
carcinoma are still lowest among gastrointestinal tumors. Of relevance is
the non-characteristic picture of disease in its early stages.
142
P287 P288
ROLE OF URGENT ENDOSCOPIC PANCREATIC DUCT
DECOMPRESSION IN ACUTE BILIARY PANCREATITIS
Rashed M.Y.T., ZEI-Khishen M.
Dept. of Medicine (HPB), ZDept. of Surgery,
Alexandria University, Egypt.
Urgent endoscopic sphinctrotomy (UES) was perf-
ormed in 38 patients(22F,16M) with a mean age
of 48 years presented with acute biliary pancr-
eatitis. Using Atlanta classification 2Z were
mild(GI) and 16 were severe(GII). GI patients
were refered within 48 hours after the onset of
severe abdominal pain, while GII patients were
delayed beyond 72 hours. UES was done as early
as possible within 8 hours. All patients in GI
recovered with no complications. In GII there
were 3 deaths(pulmonary embolism), one case of
recurrent gastrointestinal bleeding, pancreatic
pseudocyst in 2 and pancreatic abscess in one.
Retrograde cholangiography(ERCP) showed sizea-
ble stone(s) in 10, gravel(seen endoscopically)
in 13, sludge in 5, and only inflammed papilla
with no stones or gravel in 7. In 3 patients
biliary parasites(fasciola in 2, ascaris in i)
were retreived. Gallstones were found in 22, sus-
pected gravel in 5 while ii were cholecystecto-
m+/-sea.n+/-+/- paulenss but 2 were 3aundlced. Fistul-
ospnlncroComy was resorted to in 7. In the 2
patients without jaundice small stone was found
lodged in the pancreatic duct orifice rather
than the biliary orifice. This study shows that
UES halts the process of pancreatitis in mild
and severe cases. It allows safe and effective
decompression of both biliary and pancreatic
ducts. It is a safe alternative to surgery.
IMMUNOHISTOCHEMICAL ANALYSIS OF CATHEPSIN B
AND LAMININ IN INVASIVE PANCREATIC DUCTAL
ADENOCARCINOMA
..!-l. Saito, Y. Koyanagi, T. Aoki, T. Ashizawa, A. Tsuchida, T. Aoki, O. Uda,
T. Hashimoto, K. Inoue, S. Masuhara, I. Sonoda, Y. Nagakawa
Deptartment of Surgery, Tokyo Medical College, Tokyo, Japan
Purpose: Cathepsin B is a cysteine lysosomal proteinase. It is known that it
degrades the component of extracellular basement membrane as laminin. High
levels of cathepsin B have been reported to be associated with tumor
malignancy in many human tumors, but only few report can be seen in the
field of pancreatic carcinoma. So we evaluated the correlation between the
expressioti of cathepsin B, laminin and prognostic significance in invasive
pancreatic ductal adenocarcinoma.
Material and Method: Tumor specimens from 42 patients of invasive
pancreatic ductal adenocarcinoma resected surgically from 1986 to 1994 at
Tokyo Medical College Hospital. Ages ranged from 44 to 80 years (mean,
61.0 years). And they were 31 male and 11 female (=2.8:1). Formalin-fixed
and paraffin-embedded sections were stained with hematoxylin and eosin, and
were used to classify the tumors according to the classification of the Japan
Pancreas Society. Adjacent sections were stained for cathepsin B and laminin
using the streptavidin-biotin technique. Cathepsin B immunoreactivity was
evaluated as "positive" when more than 50% of the carcinoma cells were
positive, as "negative" when less than 50% of the carcinoma cells were
positive. Laminin immunoreactivity was evaluated as "positive" when there
was unequivocal immunostaining of the extracellular basement membrane, and
which was more than 50% of carcinoma nests were positive, as "negative"
when less than 50% of carcinoma nests were positive. Statistical analysis was
performed usinig the chi-square test with Yates correction.
Results: 30 (71.4%) of the 42 cases were "positive" for cathepsin B and 17
(40.5%) of the 42 cases were "positive" for laminin. Correlation was found
between cathepsin B expression and laminin expression (p<0.001). And
correlation was found between cathepsin B expression and some prognostic
significance (pathological stage, tumor size, nodal metastasis, venous
invasion, perineural invasion, etc.). In invasive pancreatic ductal
adenocarcinoma, these results suggest that cathepsin B have an important role
in tumor invasion and metastasis.
P289 P290
ACUTE PANCREATITIS DURING PREGNANCY
A. Saroukos, M. Velegrakis, E. Gaganis, M. Sawidou,
S. Kandylakis.
First Department of Surgery, Venizelion General Hospital,
Iraklion Crete Greece.
Women suffering from hypertriglyceridemia are prone to
develop acute pancreatitis during pregnancy. Pancreatitis
during pregnancy may be due to any other known reasons as
it happens to non pregnant women.
The relation between hypertriglyceridemia and pancreatitis
is well documented. Pregnancy as well as the use of
contraceptive pills which contain estrogenes increase the
triglyceridia levels rapidly. This increase is higher in familial
hyperlipidemia which raises the risk of acute pancreatitis.
The mortality of acute pancreatitis during pregnancy
indipendently on the reason is higher compared to non
pregnant women and it is estimated to 30% approximately.
We present three cases of pregnant women with
pancreatitis who were treated in our department. The first
patient suffered from familial hyperlipidaemia, the second from
cholelithiasis and the third one had no apparent reason. We
emphasize the meaning of the early diagnosis in the succesful
treatment of the disease and we make a brief review of the
whole issue.
UPPER GASTO-INTESTINAL TRACT BLEEDING DUE TO
ARTERIO-VENOUS MALFORMATION (AVM) OF THE HEAD
OF THE PANCREAS.
MD Shahrudin & SM Noori
Department of Surgery, Faculty of Medicine
University of Malaya, 59100 Kuala Lumpur, MAL.
Pancreatic AVM is a rare condition that may
cause gastro-intestinal bleeding. A 45-year
old man with an AVM of the head of the
pancreas is described. He presented with
frequent haematemesis. He had been extensively
investigated and angiogram revealed an AVM.
An exploratory laparotomy was performed after
his latest haematemesis and Whipple procedure
carried out to resect the AVM. He had a Bil-
roth ii gastrectomy i0 years earlier for a
bleeding duodenal ulcer. AVM pancreas is a
very rare condition and few had been reported
in world-wide literature. Most are congenital
but post-operative or traumatic causes should
be considered.
143
P291 P292
EFFECTS OF PANCREATIC DUCT LIGATION AND AGING ON
ACUTE TAUROCHOLATE-INDUCED PANCREATITIS IN RATS
H.Shinkail), W.Kimural), I.Hanl), K.Morikanel), T.Inouel),
N.Futakawal), T.Mutol), K. Okubo2), K. Miyasaka3)
1)First Dept. ofSurgery, University ofTokyo
2)Third Dept. of Internal Medicine, Yokohama City university
3_)Dept. of Clinical Physiology, Tokyo Metropolitan "Institute of
Gerontorogy
[Mm.] We soughtto determine the effects ofligafion ofthe pancreatic duct
and aging on acute pa.ncreatitis cause_..by taurocholate.
.[Methods]Youngadultand old malewlster rats were used. Six hours alter
ligation ofthe common bile duct in both the duodenum and liver hilus, rats
were harvested and the pancreata were perfused. Taurocholate or normal
saline was injected retrogradely into the common bile duct. The levels of
amylase and lipase in the portal venous effluence were determined as
maikers of damage to the pancreas. The pancreas was also histologically
examined afterthe perfusion experiments using an Image Analysis System.
[Rersults] 1. A nonsignificant elevation ofpancreatic enzymes in was
found in .l..rtalveno_s effluence _bythe retrograde injection ofsaline intothe.
common bile duct. lnjectioin ot taurocholate causbt amarked elevation ot
enzymes in the effluence for the first 30 min. after injection, which then
graiually decreased. 2. Basal levels o.f pancreatic enzymes were
significai.tly higher in the ligation group than in the non-ligationgroup.
Injection of sah'ne into the common bile duct had no apparent effect on
enzymes in the effluence. In contrast, taurocholate inJection.into the.
common bile duct produced a marked increase in enzymes in the portal
venous effluence. However, no significant difference was found be[ween
e ligation group and the non-likation group. 3. Similar findings were
obtained when old rats were used. 4. Alihough basal levels of enzymes
were almost the.samein non-liKatedold and young adult rats, taurocholate
injection into the pancreatic duct i.n old rats resulted in_a significant
depression ofenzymes compared t.othat inyoung adult rats. In the ligation
group, pancreatic enzymes in the portalvenous effluence following
taurocholate i_njection .te.nded to be lower in old ra.ts than in young adult
rats. The results were histologically suplgrted in that various degrees of
fibrosis were found in the pancreata ofold rats.
[Conclusion] When taurocholate was injected into the common bile duct,
1. highductal pressuredue.to ligation ofthe pancreatic ductdid not produce
any additional-damage to the pancreas in either young adult or old rats, and
2. levels.ofp.ancreauc enzymes in portal venous effluence were lower in
old rats than in younger rats.
DOWN REGULATION OF PANCREATIC
INFLAMMATION WITH A POTENT PLATELET
ACTIVATING FACTOR ANTAGONIST REDUCES THE
INCIDENCE OF PANCREATIC INFECTION.
Skaife P, Walker N*, Van Saene R*, Smith G*, Kingsnorth AN.
Depts of Surgery & Microbiology*
Royal Liverpool University Hospital.
Bacterial infection ofthe pancreas, occurring as a late sequel of
severe acute pancreatitis on a background of necrosis, is a serious
state claiming a high mortality. Previous attempts with antibiotic
prophylaxis have proven disappointing, while antibiotic treatment of
established pancreatic infection has made no impact on survival.
Translocation ofbacteria from the gut to an area ofintra-abdominal
inflammation is the proposed route ofinfection, and is proportional
to the level ofthe inflammatory process.
Using a microvascular ischaemia model of acute pancreatitis in the
rat, known to induce panceatic sepsis in 100% of subjects, a potent
platelet activating factor (PAF) antagonist, BB882, shown to
ameliorate local inflammatory changes in acute pancreatitis was
administered as a single intraperitoneal dose 30 minutes after
induction. Control animals had an equivalent dose of 0.9% saline
injected. Sham-operated animals always
exhibited sterile pancreata;treated animals demonstrated a reduction
in the incidence ofpancreatic infection (8/12) compared with the
control group (14/14), while quantitatively showing a reduction in
bacterial count compared with controls. All bacteria isolated from
the pancreas were enterobacteria present in the gut ofthat animal.
Histological analysis revealed a marked improvement in the treated
group median score 5.0, range 3-10) compared with the control
group (median score 17, range 8-18) (p=<0.005)
In conclusion, amelioration ofthe inflammatory process has been
shown to reduce the incidence and microbial pancreatic
concentration in acute pancreatitis.
P293 P294
BACTERIAL TRANSLOCATIONS IN ACUTE
PANCREATITIS IN RATS.
L.J. Souza, S.N. Sampietre, D.R. de Andrade, M.C.C.
Machado.
Department of Gastroenterology Univ. of Sio Paulo Brasil
BACKGROUND: Infection of necrotic tissue and abscess
formations are the most serious complications in acute
pancreatitis (AP). the present study was undertaken to clarify
the role of the intestinal tract as a source of infections in AP.
MATERIAL AND METHOD,: Ninety Wistar rats were divided
in eight groups. AP was induced by infusions of 2.5% Na+
taurocholate into the pancreatic duct. The control groups had
sham operation. Culture of blood, pancreas, mesenteric
lymphonodes and peritoneal cavity and cecum were
performed at 6,24,48 and 96h after AP and at the same
periods in the control groups.
RESULTS: Bacterial growing was present in mesenteric
lymphonods in AP in 60% (6h), 90% (24h), 70% (48h) and
40% (96h) (p< 0.05 when compared to control groups)
Bacteria were recovered from pancreatic tissue in 67% (6h),
90% (24h), 50% (48h) and 40% (96h) of the rats with AP.
(p<0.05). No overgrowth of cecal gram negative bacterias was
observed in AP rats. Pancreatic inflammation was more
intense at 24h after AP.
CONCLU$10N,: Bacterial translocation is an early
phenomenun already present at 6h after AP., being higher at
24h and decreasing after this time iniatilly caused by gram
positive and lately by gram negative bacteria and associated
with the severity of pancreatic lesion.
RARE NEOPLASMS OF THE PANCREAS.
C. Sperti, C. Pasquali, G. Liessi, P. Gasparoni, A. Canton,
S. Pedrazzoli.
Semeiotica Chirurgica University of Padua, ^ Radiology and
Dept. of Medicine- Castelfranco V. Hospital, Italy.
From 1970 to 1995 we observed in our Department 613 patients
with solid exocrine neoplasms and 94 pancreatic apudomas.
Excluding ductal adenocarcinomas, insulinomas and gastrinomas,
38 (5.4%) patients had a rare type of pancreatic malignancy,
commonly referred or suspected to be of ductal origin. These
included 13 acinar cell carcinomas 14 clinically silent endocrine
tumors, 4 secondary neoplasms, 3 pleiomorphic (giant cell)
carcinomas, sarcoma, leiomyosarc...,a, schwannoma,
lymphoma. Among 13 acinar cell carcinomas, 4 had liver spread
and 7 local invasion; 3 underwent resection. Eight 114 apudomas
had liver metastases and 9/14 underwent resection of gross tumor.
Two/3 pleiomorphic carcinomas underwent resection. The patient
with sarcoma had surgical exploration and radiotherapy. The
patient with leiomyosarcoma underwent gastric and biliary by-pass,
followed by radio- and chemotherapy. The malignant schwannoma
was resected despite extensive local involvement to surrounding
organs. The patient with lymphoma had radio- and chemotherapy.
All 4 secondary tumors were resected. Median survival time in
these 38 rare pancreatic neoplasms was 18 months (range
120).
In 5.4% of cases with solid pancreatic tumor, supposed to be of
ductal origin at admission, was shown a different histology and
behaviour. In 51% of these cases a resection was possible and
long-term survival was not uncommon despite extensive disease
was found at surgery.
Study supported by Italian National Research Council (CNR), project nr.
94.01179.PF39
144
P295 P296
RECURRENCE AFTER RESECTION FOR DUCTAL
ADENOCARCINOMA OF THE PANCREAS.
C. Sperti, C. Pasquali, *A. Piccoli, A. Canton, S. Pedrazzoli.
Semeiotica Chirurgica, and Medicine, University of Padova, Italy
Long-term survival for patients with carcinoma of the pancreas is
poor, even after resection. Most patients who undergo curative
resection develop recurrence usually at the same site of resection
or in the liver, but there are only a few reports on the incidence and
pattern of tumor relapse Detailed knowledge of the sites of
recurrence of carcinoma of the pancreas and the study of the
factors influencing disease-free survival, is important in developing
surgical and adjuvant treatment.We analyzed the pattern of failure
and clinico-pathologic factors influencing the disease-free survival
of 78 patients who died after macroscopic curative resection for
pancreatic cancer performed in our Department from 1970 to 1992.
Local recurrence was a component of failure in 56 patients
(71.8%), hepatic recurrence in 48 (61.5%), both accounting for 97
% of total recurrence rate. Ninety-five per cent of recurrences
occurred by 24 months after operation. Median disease-free
survival time was 8 months, and cumulative 1, 3 and 5-year
actuarial disease-free survival rates were 66 %, 7% and 3%
respectively. Multivariate analysis showed that tumor grade
(P=0.04), microscopic radicality of resection (P= 0.04), lymph node
status (P=0.01) and size of the tumor (P=0.005) were indipendent
predictors of disease-free survival. Patterns of failure and disease-
free survival were not statistically influenced by the type of surgical
procedure performed. Median survival time from the detection of
recurrence until death was 7 months for local recurrence vs. 3
months for hepatic or local plus hepatic recurrence (P<0.05).
From our experience it appears that surgery alone is an inadequate
treatment for cure in patients with pancreatic carcinoma. Effective
adjuvant therapies are needed to improve Ioco-regional control of
pancreatic cancer after surgical resection.
Study supported by Italian National Research Council (CNR), project nr.
94.01179.PF39
SURVIVAL AFTER RESECTION FOR DUCTAL ADENOCARCINOMA OF
THE PANCREAS.
C. Sperti, C; Pasquali, A. Piccoli, V. Costantino, S. Pedrazzoli.
Semeiotica Chirurgica, and Medicine, University of Padova, Italy
Resection is the only chance of cure for pancreatic cancer, but the
problems related to resectional surgery of pancreatic carcinoma still
remain the following: 1) the high operative risk; 2) the choice of surgical
procedure; and 3) the poor 5-year survival rate. However, a significant
decrease in mortality and morbidity has been reported recently by several
specialised centers together with an encouraging 5-year actuarial survival
rate: 21-24%. Patients with no residual tumour after resection had 5-year
survival rate of 36% and patients without lymph node metastases had a
57% five-year survival rate. A retrospective study of 113 patients (20% out
of 549 patients with pancreatic adenocarcinoma who underwent
pancreatic resection in our Department'from 1970 to 1992, was performed
to evaluate early and late results and the eventual progress of this surgery
in the recent years. We also analyzed the clinico-pathologic factors related
to prognosis. Surgical resection was performed whenever it was
technically possible and when no distant metastases were detect. Limited
invasion of portal and/or mesenteric vein were not considered a
contraindication for pancreatic resection. The post-operative hospital
mortality rate was 15% (4.7% in the last 11 years). Actuarial 5-year
survival rate was 12%. Survival was significantly influenced by age (P
0.03), vascular resection (P 0.02), radicality of operation (P 0.01),
number of transfused blood units (P 0.01), tumoCs grade (P 0.002),
lymph node status (P 0.001), perineural invasion (P 0.01), tumor's
size (P 0.008), preoperative diabetes (P 0.001), and pTNM stage (P
0.0001). Multivariate analysis showed that stage, diabetes, age, and grade
were independent predictors of long-term survival. The type of pancreatic
resection (Whipple, subtotal, total, or distal pancreatectomy) did not
influence prognosis. Five-year survival was 14% in the period 1970-1981,
and 11% in the period 1982-1992, without statistical difference. These
results suggest that patients' characteristics and tumor's findings rather
than operative procedures affect long-term survival after resection for
pancreatic carcinoma.
Study supported by Italian National Research Council (CNR), project nr.
94.01179.PF39
P297 P298
LONG-TERM RESULTS OF SURGICAL TREATMENT OF
PANCREATIC PSEUDOCYSTS: STUDY OF 103 PATIENTS
G. Spiliopoulos, M.A.C. Machado, M. Lakehal, C. Stasik, B. Chareton, N.
Georgieu, B. Meunier, J.P. Campion, B. Launois
Department of Surgery, University ofRennes France.
During the period May 1972-December 1994, 111 patients underwent
surgical treatment of pancreatic pseudocysts, following acute (14 cases) or
chronic pancreatitis (97). Ofthese, 103 patients underwent operation other
than resection. There were 82 men and 21 women. The mean age of
patients was 44.4 12.4 years. Alcohol abuse account for 81.5% (84
patients) ofcases ofpancreatic pseudocysts.
The main indication was persistent pain (62%), pain and compression
(13%), palpable mass (6%) and pancreatic fistula (6%).
Nineteen patients underwent percutaneous drainage before the surgical
procedure. Cystgastrostomy was performed in 39 patients, cystejunostomy
in 34 patients, cystduodenostomy in 6 patients. External drainage of
pseudocyst was performed in 25 patients. The operative mortality was 5%
with postoperative morbidity of 17%. The mean size ofthe cysts was 7.6 +/-
3.4 cm. Fifty-eight pseudocysts were located in the body and tail (48%),
and 45 in the head of the pancreas. The diagnosis was made in the
preoperative period in 97 patients (94%).
The mean. hospital stay was 15 +/- 9 days. The five-year survival rate
was 80% and ten-year survival rate was 50%. Sixty two patients are still
alive. Among these patients, 19 (30.6%) present insulin-dependent
diabetes and 13 (21%) presents persistent pain. Alcohol abstinence was
obtained in 34.6% of alcohol dependent patients. A good quality of life
was obtained in 80% of living patients in a long term follow-up (up to 20
years).
Long term results are correlated to the natural course of chronic
pancreatitis. The surgical management of pancreatic psei]docysts still
represents the best therapeutical option, with low mortality and morbidity
rates.
SURGICAL TREATMENT OF ACUTE PANCREATITIS
ANALYSIS OF 137 PATIENTS
C. Stasik, M.A.C. Machado. M. Lakehal, G. Spiliopoulos, B. Chareton, B.
Meunier, JP. Campion, B. Launois
Department of Surgery, University ofRennes France.
Controversy still surrounds the management of severe acute
pancreatitis. Between January 1973 and December 1993, 137 patients with
severe acute pancreatitis were operated on in our Surgical Department.
The patients' age range from 22 to 80 years with a mean of 49.6. There
were 92 men and 45 women. Alcohol abuse account for 28.5% (39
patients) of cases of acute pancreatitis. Biliary stones was the cause in 36
patients (26.3%). The surgical management was decided in pl;esence of
necrotic and/or septic complications of acute pancreatitis. There was no
systematic or scheduled operations. The surgical approach was tailored to
the operative findings and clinical course.
The majority of patients underwent necrosectomy and drainage with
re-explorations only when necessary. Ten patients underwent pancreatic
resection, with high mortality rate (60%). The overall mortality rate was
32.8%. The median hospital stay was 60 days. The morbidity rate was
52.8% and consisted on intra-abdominal collections, pancreatic fistula and
intestinal complications. Thirty-four percent of the patients (46) required
reoperation, usually because of persistent intra-abdominal sepsis. There
was a significant (p < 0.05) lower survival rate in this group ofpatients.
There was correlation between the extension of necrosis and mortality
rate (p < 0.05). Patients with infected pancreatic necrosis at the time of
operation presented an increased mortality (p<0.05). However, in patients
with sterile pancreatic necrosis that presented postoperative infection,
there was no significant increase in mortality rate.
Two main factors should influence the outcome of severe acute
pancreatitis, namely the extension pancreatic necrosis and the presence or
absence of infection. Therefore, the management should be more
aggressive in this group ofpatients.
145
P299 P300
EARLY GRAFT LOSS AFTER PANCREAS TRANSPLANTATION: AN
ANALYSIS OF RISK FACTORS
RJ Stratta, RJ Taylor, R Sindhi, D Sudan, P Castaldo, Gill, J Jerius
Department of Surgery, University of Nebraska Medical Center, Omaha,
Nebraska, USA
Although the results of PTX continue to improve, early graft loss remains
a problem. Over a 79 month period, we performed 195 PTXs in 185
recipients, including 133 combined pancreas-kidney (PKT), 17 sequential
pancreas after kidney (PAK), 3 combined liver-pancreas (LP), and 42
solitary PTXs. We retrospectively analyzed causes and risk factors for
pancreas allograft loss occurring in the first 4 months after PTX. All
patients underwentwhole organ PTXwith bladderdrainage and received
triple or quadruple immunosuppression. Results: A total of 29 pancreas
grafts (15%) were lost. Seven patients (4%) died with functioning grafts
(2 myocardial infarction, arrhythmia, 3 infection, liver failure). Of the
remaining 22 grafts lost, 11 were due to thrombosis, 3 rejection with
thrombosis, 5 poancreatitis, 2 infection, and hemorrhage. The incidence
of early graft loss varied according to type of transplant: PKT (10%);
PAK (41%); LP (67%); and solitary PTX (17%); p<0.001. In patients
undergoing pancreas without kidney transplant, the risk of vascular
thrombosis with graft loss was higher (8/62=13% vs 6/133=4.5% PKT,
p<0.05). The 3 cases of early graft loss due to rejection were also in
PTX recipients not receiving a PKT. An effect of donor age was noted
in the PKT recipient group. In PKT recipients receiving organs from
donors 48 years of age or older, the incidence of vascular thrombosis
was higher (25% vs 4%, p=0.03) when compared to donors below 48
years. An effect of donor weight was noted in all PTX recipients. When
a PTX recipient received an organ from a donor weighing 88 kg or
greater, the risk of early graft loss was higher (50% vs 9%, p<0.001). In
patients without early graft loss, patient survival is 96% and pancreas
graft survival is 91% after a mean follow-up of 32 months. Conclusions:
Early pancreas grat loss is an important source of morbidity after PTX
and is most commonly due to vascular thrombosis. Specific donor (age
and weight) and recipient (type of transplant) risk factors for early graft
loss can be identified. The use of selective anti-coagulation or more
stringent donorselection may minimize the risk of early graft loss, leading
to reduced morbidity and improved long-term results.
THE DUODENAL SEGMENT (DS) IN VASCULARIZED PANCREAS
TRANSPLANTATION (PTX)
R.J. Stratta, R.J. Taylor, R. Sindhi, D. Sudan, P. Castaldo, J.A. Lowell,
I. Gill, J. Jerius, S.J. Radio
Department of Surgery, University of Nebraska Medical Center, Omaha,
Nebraska, USA
Bladder drainage by the DS technique is currently the preferred method
of PTx but is associated with unique complications. Over a 79 month
period, we performed 195 PTxs (133 combined kidney-PTxs, 59 solitary
PTxs, 3 combined liver-PTxs) in 185 diabetic patients. All patients
underwent whole organ PTxwith bladder drainage using a 6-8 cm length
of DS as an exocrine conduit. Results: DS pathology or problems
occurred in 47 cases (24%). Fourteen DS leaks were documented in 10
patients and required operative repair; 7 developed peri-pancreatic
sepsis, with 6 requiring 2 re-operations. Thirteen DS leaks occurred
early, at a mean of 64 days after PTx. Six DS leaks occurred in 4 pa-
tients with cytomegalovirus (CMV) duodenitis. Twenty-four patients
(13%) underwent enteric conversion foreither refractorydehydration with
metabolic acidosis (n=17), DS leak (n=2), severe dysuria with urethral
disruption (n=2), or recurrent duodenitis with hematuria (n=3). Cystos-
copy was performed in 16 patients for significant hematuria originating
from the DS (3 CMV duodenitis, 5 anastomotic bleeding, 3 rejection, 5
nonspecific duodenitis). One patient developed stone formation from the
DS staple line. Five patients experienced pancreatic ductal obstruction
early after PTx (3 pancreatitis, 2 pancreatic fistula), and 3 were suc-
cessfully managed non-operatively. DS histopathologyaftercystoscopic
biopsy included acute rejection (n=8), focal erosion with villous atrophy
(n=8), and chronic rejection (n=6). In patients with DS complications,
patient survival is 95%, and pancreas graft survival is 84% after a mean
follow-up of 32 months. Conclusions: Complications related to the DS
remain an important source of morbidity but rare cause of mortality after
PTx. In spite of unique side effects, transplantation of the DS as an
exocrine conduit remains the method of choice for either bladder or
enteric drainage after PTx.
P301 P302
PROGNOSIS OF THE ADVANCED DUODENAL
CANCER AFTER RESECTION WITH A CASE REPORT
M.Sugita. M.Ryu, T.Kinoshita,M.Konishi,N.kawano,H.Tanizaki,A.Cho
Dept. of Surgery, National Cancer Center Hospital East,Kashiwa,
Japan
We report a case of duodenal cancer located in a supraampullary
part in a 48-years-old male whom pylorus preserving pancreato-
duodenectomy (PPPD) was performed with lymph nodal dissection.
The resected specimen had Borrmann 2 type cancer which was
4.5cm in diameter. Histopathological examination revealed
moderately differentiated adenocarcinoma with subserosal layer
invasion. Lymph nodal metastasis was observed in node out of 48
dissected, nodes.The postoperative course was uneventful. The
patient has been healthy without evidence of recurrence and half
year after operation.
We also report Japanese review of 28 cases of advanced duodenal
cancer reported between 1990 and 1995. The location of the lesion
was bulbar in 7 cases (25%), supraampullary in 14 cases (50%), and
infraampullary in 7cases (25%). Distal gastrectomy including the bulb
was performed in 3 cases, pancreatoduodenectomy in 23 cases,
and PPPD in 2 cases including our patient. Over all 5-year survival
rate after resection was 25.1%. Lymph nodal metastasis was
revealed in 16 cases. 5-year survival rate of the cases without lymph
nodal metastasis (68.6%) was significantly better than those with
metastasis (8.5%). Hepatic recurrence was occurred in 8 cases and
lymph nodal recurrence especially in hepatoduodenal ligament was
occurred in 4 cases.
We concluded that to perform curative resection with lymph nodal
dissection especially in hepatoduodenal ligament and adjuvant
chemotherapy are important to improve the outcome of advanced
duodenal cancer.
Adult onset of Nesidioblastoses:
A surgical dilemma and the effect of a somatos-
tatin analogue.
Joung Wo Suh,M.D.,
Chong Seob Cho,M.D.
Department of GqERALSurgery
Dongguk University Kyongju Hospital
The author experienced 2 cases of adult onset of nes-
dioblastoses,one in 38 year old male and another in
58 female.
For the first case, pancreaticoduodenectomywas done
removing about 60% of the pancreatic tissue. But the
result was not satisfactory with relapsing hypoglycem-
ic bouts in the occasion of severe diarrhea or overex-
ertion suggesting inadequet resection.
For the second case, 85% distal pancreatecton!f was
done with complete relieving of symptoms for.the foll-
owing 3 months but with recurring hypoglycemic episod-
es thereafter especially in fasting period, suggesting
pancreatic islet cell regeneration or proliferation.
We treated these two cases of persisting and recurred
hperinsulinism with Somatostatin analogue 201-995 for
four months for the first case and three months for
the second case and acquired long term cures for two
years and one and a half years up to now respectively
The blood insuline, C-peptide and PP remain in normal
range and fasting blood glucose remain around 80 mg/dl
and fasting I-G ratio below 0.4.
From these results, we inferred that Somatostatin an-
alogue 201-995 not only has the suppressing effect on
the islet cell's secreting function but also has supp-
ressing and deterring effect on the cellular growth it
self, suggesting son kind of antineoplastic activity.
We strongly think that further clinical and experir-
ntal study is necissary.
146
P303 P304
IMPROVEMENTS AND PERSPECTIVES IN THE DIAGNOSIS
AND TREATMENT OF PANCREATIC CARCINOMA
U. Sulkowski, P. Dinse, V. Lange1, J. Brockmann, J. Kocik
Departments of General Surgery and 1Anaesthesiology,
Westflische Wilhelms-University, MOnster, Germany
A retrospective study was carried out to evaluate
whether any improvements have been made for
pancreatic cancer, the most fatal gastrointestinal
malignancy, during the last two decades. From 1973 to
1994 1004 patients suffering from pancreatic
adenocarcinoma were treated at the Department of
Surgery of MOnster University Hospitals. 578 were male
and 426 female (sex-ratio: 1.4 1). 717 tumors were
located in the pancreatic head. Over the years the rate of
curative resections increased from 15.9% to 25.2%.
Operative mortality for curative resections could be
reduced from 12 to 2.4%. The crude 5-year-survival rate
for patients treated until 1989 was 2.0% showing a minor
improvement. With a palliative radiochemotherapy in 45
patients with irresectable tumors we observed a median
survival of 15 months. Two patients are alive and free of
tumor for more than 4 years, now. Overall, despite a
significant reduction of perioperative mortality the
general prognosis continues to be very poor. New
treatment regimens have to be developed, taking into
account that in tumors without distant spread a
combination of postoperative radiation and
chemotherapy was succesful in improving the
prognosis.
EVALUATION OF FUNCTIONAL ACTIVITY
AND VIABILITY OF PANC,REATIC TISSUE
DURING COOL PRESERVATION
KUSTADIOL SOLUTION.
Tc,hao A.V., Zakharova O.A., Sawina T.V.,
Buriev I.M., Ionkin D.A.
A.V.Vishnevsky Institute of Surgery,Moscow,Russia
The viability of pancreatic tissue during preservatio.n
in Kustadiol solution /Dr.F.Kohler Chemic GMBH/
was studied in experiments on dogL RNA synthesis
in pancreatic cells was stadied in 2,6,8 and 12 houJrs
after cool preservation by means of light and
electron microscopy radioau.tography.D.esintegratio.n
of cell's organelles began from 8 hours of
preservation. In 12 hours only 60% of cells
incorporated 3H-Uridine to the nucleus i.n
comparision with the first hour of pr,servation.
Qualitative evaluation of tissu vi.ability and
functional activity is important for prognosis of the
results oforgan transpantation.
P305 P306
EFFECTS OF PROTON PUMP INHIBITOR ON EARLY
GASTRIC STAGNATION AFTER PYLORUS-PRESERVING
PANCREATICODUODENECTOMY; RESULTS OF A
RANDOMIZED STUDY
N. Toyota, T. Takada, H. Yasuda, T. Uchida, T. Isaka
First Department of Surgery, Teikyo University
School of Medicine, Tokyo, JAPAN
The suppressive effects of a Proton Pump Inhibitor in improving
gastric stasis during early postoperative period after pylorus-
preserving-pancreaticoduodenectomy (PPPD) was assessed.
Thirteen PPPD patients were divided into two groups. Group
(n=7) served as controls and were given no medication. Group 2
(n=6) received Proton Pump Inhibitor through jejunal tube.
The daily volume and total acidity of the gastric juice, aspirated
via nasogastric tube, were measured on post-PPPD days 1-7.
Two patients in group were withdrawn from this study after 3
days due to a large amount of excreted gastric juice (exceeded
2,000 ml). The mean daily aspirated volume of gastric juice in
group was 1,200 ml on the 4th postoperative day, and it
gradually fell after the 6th postoperative day. In contrast, in
group 2, the gastric juice secretion was significantly lower
(p<0..05), being 175 ml on the first postoperative day, than in
group 1. The total acidity of the gastric juice in group 2 was
significantly lower after the 3rd postoperative day than in group
1. These results indicate that the postoperative administration
of Proton Pump Inhibitor via jejunal tube suppresses the volume
and acidity of the gastric juice after a PPPD.
LAPAROSCOPIC SPLENECTOMY. CLINICAL RESULTS IN A
SERIES OF 34 CASES
M Tr|as, EM Targarona, C Balagu, Ardid, JJ Espert.
Serv. of Surgery. Hosp. Clinic. Univ. of Barcelona. Barcelona. SPAIN.
During last years it have been shown that laparoscopic splenectomy is a feasible
and safe alternative to open surgery. Controversies exist about the preferred
technique (anterior or lateral approach), or the eficacy on long term control of
haematological diseases. AIM: To report the immediate results and early follow
up of a consecutive series of 34 LS. MATERIAL AND METHOD. Between Jan
93- Dec 95 we have performed 34 LS. Diagnosis was thrombo.cytopenic purpura in
21 cases, spherocytosis in 6 cases, HIV-thrombocytopenia in 4 and haemolityc
anemia in 4 cases. RESULTS: There were 12 women and 22 men. Mean age was
34 + 12 y. Operative time was 180-J:60 min. 3 patients were converted (9%) and 9
patients required transfusion (26%). One patient was reoperated for
hemmorrhage, other developed a postoperative pneumotorax and 4 patients had a
febrile syndrom (Morbidity 7/34 (21%). In this series, 3 (9%) accessory spleens
were identified. There were no postoperative deaths The length stay was 4.5 + 3 d
(3-14). Ten patients were operated with an anterior approach and 24 with lateral.
There was a significative reduction of operative time, number of trocars used,
transfusion rate and stay in the group operated with a lateral approach vs anterior.
Mean follow was 13:1:9 m. Five patients (14%) failed to LS (4 ITP and HIV-
thrombopenia). One patient needed to be reoperated for a trocar hole hernia. In the
subset of patients with ITP, 4 of21 (19%) failed to respond to splenectomy.
CONCLUSION: These results suggest that LS is a safe alternative approach to
open surgery. Lateral approach facilitates LS, with a substantial diminution of the
operative time and blood requirements. Although a shorter follow up, LS seems to
offer a satisfactory long term results for treatment of autoinmunne or hemolytic
diseases requiring splenectomy.
147
P307 P308
THE CLINICOPATHOLOGICAL ASPECTS OF
PANCREATIC CANCER WITH PORTAL VEIN
RESECTION
A.Tsuchida, H.Saito, K.Inoue, T.Hashimoto, O.Uda,
T.Aoki, T.Aoki, S.Masuhara, I.Sonoda, T.Ashizawa,
T.Aoki, Y.Koyanagi
Department of Surgery, Tokyo Medical College,Tokyo, Japan
For the past 6 years we experienced 37 surgery of pancreas
cancer where 16 cases were resected associated with portal vein
and clinicopathologically evaluated. For the pathological stage
(based on the Japanese Society of Biliary Surgery) there were
case of stage 11 of stage aand 4of stage b. For the
resection of portal vein there were 3 of weadge resection and
13 of coronary resection. In 9 cases portal-IVC bypass was
used. There were significant differences between portal vein
invasion and tumor size, lymphatic infiltration, choledochal
invasion, duodenal invasion and retropancreatic invasion. For
the prognosis 2 of 16 cases are alive (within year) and 14
were died (the longest case:550 days). The cause of death were
12 case of metastasis or recurrence and 2 cases died within
one month after surgery. Although the resectability has been
improved, their prognosis has not been improved. The
multidisciplenary treatment included surgical therapy should
be necessary for the improvement of QOL.
SURGICALTRF.TMENT OF PATIENTS
WITH INSUUNOMAS.
V.Tsvirkoun, M.Danilov, V.Pomelov, V.Vishnevsky.
A.V.Vishnevsky Institute of Surgery, Moscow, Russia.
This study is about the surgical treatment of 15 pts. with insulino-
mas. The age range was 21-71 years (mean 41 yrs.). Women=7,
men=8. Location of insulinomas: pancreatic head-5, body-4, tail-5,
diffuse-1. Preoperative diagnosis involved US, CT, angiography. Ef-
ficiency- 35.6%. Intraoperative US (IOUS) was performed in 12 pts.
Efficiency 83.3%. Nine pts. were operated for the first time, 6 had
repeated operations. Organ preserving operations were performed
in 8 pts. 53.3% (enucleation-6, minimal pancreatic resections-2);
pancreatectomies 7 pts. 46.7% (distal resection with splenec-
tomy- 6, total- 1). No postoperative mortality was observed. Eight
pts. (53.3%) required additional surgical procedures including 3 re-
laparotomies. Success of surgical treatment of patients with insuli-
nomas depends on the following conditions: determination of tumour
location US, CT, angiography, palpation, IOUS; stabilization of glu-
caemic levels pre-, intra- & post- operatively- with the help of
,,Biostator,,; verificati6n of the fullness of tumour excision IOUS,
,,Biostator,,; prophylaxis of postoperative pancreatitis Sandostatin,
cytostatics.
P309 P310
ANATOMICAL LOCATION AND HISTOLOGICAL
DIFFERENCES IN VENTRAL AND DORSAL PANCREAS
T. Uchida(1,2,3), K. Suda(2), T. Takahashi(3), T. Takada(1)
(1) First Department of Surgery, Teikyo University, School of
Medicine, Tokyo, Japan (2) Department of Pathology,
Juntendo University, School of Medicine, Tokyo, Japan (3)
Department of Pathology, Institute of Development, Aging and
Cancer, Tohoku University, Sendal, Japan
Based on the histological evidence that Langerhans islet is
positively stained by anti-pancreatic polypeptide staining in the
ventral pancreas and negatively in dorsal pancreas, we
investigate anatomical location and histological differences of
both of pancreatic anlagen. Material and Method 35
autopsy specimens with normal pancreas were subjected to this
study. "Makrozerien" (Journal ofMicroscopy, Vol. 149, 175-183,
1988) method was used for three dimensional reconstruction.
Further the sections were stained with hematoxylin-eosin,
grimelius, aldehyde-fuchsin, lead-hematoxylin, anti-pancreatic
polypeptide, anti-glucagon, anti-insulin, anti-somatostatin and
anti-serotonin staining. [Results] (1) Ventral pancreas was
found to be existed only in the backside of the head of pancreas.
Dorsal pancreas was being anterior area ofthe head ofpancreas.
Body and tail of the pancreas were all dorsal pancreas. (2) In
the ventral pancreas, many cells containing brown granules in
the irregularly shaped islets were noted by the Grimelius
method. These granules were positive for anti-pancreatic
polypeptide and negative for anti-glucagon and anti-insulin.
On the other hand in the dorsal pancreas, black granules in the
uniformed islets were positive for anti-glucagon (A-cell) and
anti-insulin (B-cell). [Conclusion] (1) The ventral pancreas
located posterior area of the head of pancreas and the dorsal
pancreas covered in front of it. (2) Differentiation of both
anlagen was macroscopically and immunohistochemically clear.
DELAYED GASTRIC EMPTYING AFTER PYLORUS PRESER-
VING PANCREATODUODENECTOMY AND STANDARD
WHIPPLE PROCEDURE.
MI vn Berge Henegouwen, TM van Gulik, TM Moojen, LTh de Wit,
EAJ Rauws, H. Obertop, DJ Gouma. Department of Surgery, Academic
Medical Center, Amsterdam, The Netherlands.
The pylorus preserving pancreatoduodenectomy (PPPD) has been accep-
ted as an alternative to the classic Whipple procedure (PD). It has been
suggested however that this procedure is associated with delayed gastric
emptying which is probably leading to a prolonged hospital stay.
Therefore the aim ofthis study was to evaluate whether the PPPD is asso-
ciated with more complications, delayed gastric emptying and prolonged
hospital stay compared with a classic PD.
During a 3 year period (1993-1995) 106 consecutive patients underwent a
pancreatoduodenectomy. Seventy-five patients (mean age 63 y, range 37-
78) underwent a PPPD and 31 patients (mean age 60 y, range 38-79) a
PD. The overall hospital mortality was 1.9%.
Postoperative complications were not different between the PPPD and PD
respectively 33% versus 29%. Delayed gastric emptying (defined as > 10
days gastric suction) occured after PPPD and PD in respectively 27% and
26% (NS).
Patients after PPPD had gastric suction for a median period of7 days, sig-
nificant longer compared with 2 days gastric suction after PD (p < 0.05).
There was however no difference in the median period ofenteral feeding
by feedingjejunostomy respectively 11 and 8 days and median hospital
stay respectively 20 and 22 days.
Patients with postoperative complications (n 33) had a longer period of
gastric suction: compared with patients without complications respective-
ly 8 and 5 days (p < 0.05) and a prolonged hospital stay respectively 30
versus 18 days (p < 0.05).
It is concluded that PPPD is associated with prolonged p.o. gastric suction
time but without prolonged enteral feeding and hospital stay. A prolonged
gastric suction time and hospital stay seems to be associated with postope-
rative complications.
148
P311 P312
LAPAROSCOFY IN ACUTE PANCREATITIS
D.Venskutonis. J.Babrav/ius.
2rid entofSurgery, Kamms Medi(C)al Academy, Lithuania
We have treated 815 patients with acute pantav.tis in 1980-1994.
Seventy two (8.8%) were reader surgery, 49 (6.0%) underwent
dosed treatment, 24 patients died. The total lethality made up 2.9
%; after mngery 29.2%; after closed treatment 6.1%.
For the diagaosing mui treatment of acute paucreatitis we have
used urgent and dynamic lapy w/.'th the routine clinical
examination. Two steps of laparoscopy were div/ded: diagnostic
and curative. In the diagnostic step the form of pancreatitis, the
grade and spreading of peritonitis, following sad parallel
pathologies were established.
When the diagnosis of enzymatic sseptio peritonitis was formed
with the exclusion of parallel pathology, whioh needs urgent
mrge, we passed to the endosoopic trealment procedures, lrom
the 116 urgent laparosoopies 49(42.2%) were made due to the
acute pmuaeatitis, lor 38 patients with diagnosed asepti(C)
enzymatio peaitonitis beside the routine conservative treatment we
have used lapasopi drainage of peritoneal avity (sub hepatio
and fight lateral spaces) and novooain blockades of round hepati(C)
ligament. In the first days the common state of patients improved:
the pain deoreased, the nausea and vomiting stopped, intoxistioa
dezreased. We have observed no ootnptioatios after the
endosoopie diagnostiz sad ourative procedures. Three patients
The method permits to form the exact diagnosis and in most cases
exolude the non obligatory surgery, to decrease the number of
oomplioations sad shorten the presenoe ofpat/ent in hospital.
ENDOSCOPIC TREATMENT OF PANCREATIC AND BILIARY FISTULA
Z.Wajda, M.Dobosz, A.Babicki
II Department of Surgery, Medical University of Gdask
Poland
Authors present 16patlents with biliary or pancreatic
fistula. In ii cases, ERCP examination revealed billary
fistula, in 5 patients pancreatic fistula was diagnosed
The causes of billary fistula were cholecystectomy
in 7 patients /4 laparoscopic and 3 classical/, T-tube
drainage failure in 2 patients, stab abdominal wound
and blunt abdominal trauma in one patient each.
Pancreatic fistula was a consequence of necrotizing
pancreatitls in 2 patients, and in patients undergone
insulinoma enucleation, distal pancreatic resection
and after blunt abdominal trauma. In all the patients,
endoscopic papillotomy was performed within 9-18 days
after the fistula was diagnosed.. Eight billarM fistu-
las and three pancreatic fistulas healed spontaneously,
following endoscopic papillotomy only. The bile and
pancreatic Juice outputof the fistulas was reduced
for about 75% within 2 dams after the papillotomy.
Three patients with biliary and two with pancreatic
fistula required additionally an endoprosthesls
insertion. In these patients, the fistulas were also
healed within 2 weeks after the prosthesis implacement.
Authors conclude, that endoscopic papillotomy is an
effective method in billary and pancreatic fistula
treatment. Consecutive endoprosthesls implacement is
necessary in cases when papillotomy is uneffectlve
in billary and pancreatic fistula healing.
P313
THE ROLE OF ENDOSCOPIC AND
INTRAOPEARTIVE ULTRASOUND IN THE
LOCALIZATION OF THE PANCREATIC INSULOMAS
T. Winternitz. L.Flautner, T.Tihanyi, J. Horhnyi
1st Department of Surgery, Semmelweis Medical University
Budapest, Hungary
Adequate localization is remains a difficult problem associated
with endocrine pancreatic tumors. There are several reasons for
this: tumors are small, there is no constant site within the pan-
creas, multiple tumors are possible, etc.
From 1967 to 1995 we performed in our department 56
operations for supposed insular pancreatic tumors.
We studied the predictive value of CT, Ultrasonography,
Angiography, Scintigraphy, Endoscopic US; Intraoperative
Sonography and the Intraoperative Palpation in order to
compare the localization ofthese tumors.
The localization based on our patients material was correct
with sonography, angiography and CT in 12 (43%) of 28, in 20
(83,3 %) of 24 and in 8 (42,1%) of 19 examinations
respectively. The intraoperative US was true positive in 14
(93,3 %) of 15 patients and at two operations this examination
was true negative. The endoscopic US was correct at 2 out of2
examinations. Up to the 88,2 % of the tumors was palpable
intraoperatively.
According to our experiences it seems that preoperative
localization is difficult. Angiography and endoscopic
ultrasound proved to be most helpful. Surgical exploration was
indicated in most of the cases based upon the laboratory
findings and clinical symptoms of hormonal activity of the
endocrine tumors. In these cases the localization can be
accurately done by Intraoperative Echography.
149
POSTER PRESENTATIONS
Topic: BILIARY
P314 P315
LAPAROSCOPY VERSUS MINILAPAROTOMY
FOR CHOLECYSTECTOMY
T. Abe, N. Sakashita, T. Suto, S. Ogasawara,
T. Kikuchi, T. Ishikawa, H. Yamada, Y. Kusaka
Demof Surge, lwate Prefectural Fukuoka Hospital,
Ninohe, lwate, Japan
Laparoscopic Cholecystectomy (LC) for gallbladder stones has
quite popular for these years in japan. Wehave experienced
80 cases of LC for four years. At the same time, we began
minilapcolc cholcyaomy/[O in the ,tht upper
quadrant We had 30 minilaparotomy cases. Mean wound
length is 4.3cm. We introduce both operative methods and
report the comparative study between LC and MC about mean
operating t/me, blood loss, complications, the use of analgesics
and hospital stay.
Results
OPE. BLOOD COMPLI- ANAL- HOSPITAL
TIME LOSS CATION GESIC STAY
(rain) (ml) (case) (%) (day)
LC 105 23 25.0 8.2
MC 85 75 2 56.7 12.4
Conclusion
Our comparve study suggested that ittakes longer operating
time in LC than MC, but LC is superiorin otherfactors to MC.
In wound healing, LC is more excellent than MC. LC is
desirable method for cholecystecmmy.
SPCTANEJS ENTEROBILIARY FISTULA
A'.' Agorogiannis
Surgical Unit.District Hospital Of Iarisa-Greece.
The aim of this study is to see how we treat today in
the era of laparoscopic billary surgery,the internal en-
terobiiary fistula.During the last 17 years we operate
d upon 4.200 cases of benign deseases of the biliary sy
sten.Among them we treated also127 spontaneous enterobi
liary fistula.ln 70 cases the fistula was cholecystoduo
deanal.In 25 cases the fistula was cholocystocolic.in
21 cases the was between the chocbus syst and the CBD,
(all types of Mirrizi syndrcme).In 10 cases the fistula
was cholosystogastric and chodochogastric.The etiology
of the last type was gastric ulcer adhered to the ceru-
men bile duct.Although the experien in the laparosco-
pic treatment of cholelithiasis is continuously grouing,
the internal biliary fistula are treated mostly by the
open method.This operation needsseparation of the adhe-
red organs,closure of the opening to the intestinal wall
exloration of the ccmon bile duct for stones and other
gine surgical actions that are impossible to be done la-
parscpcaly at the present time.In the internationall
bibliography there are scme cases treated laparoscopica
ly,but are rare exeptions.Another problem is the ileus
due to obstruction by gall-stones that passed through
the fistulous tract into the small intestine (gall-sto-
ne ileous ].
Conclusions:According to our experience in the treatment
of internal enteroliary fistula,these must be treated
by the open surgical technic.As time passes surgeons ga-
te more experience and will be able to do and operation
s laparascopicaly.At the present time to avoid iatroge-
nic damages to the extrahepatic bile ducts and to the
intestine we reccment the classic open method for the
treatment of spontaneous enterobiliary fistula.
P316 P317
INFLUENCE OF ENDOTOXIN ON THE LIVER
MICROCIRCULATION AT CHOLESTASIS
O.Akhaladze
Department of Hepatic Surgery,
I.M.Secenov Moscow Medical Academy, Russia
Investigations by means of Limulus Amebocyt Lysate (LAL)
test proved, that at mechanical obstruction ofbile duets
inereses portal vein endotoxemia.Using hydrogen clearance
polarography method we studied the liver tissue blood flow
in 7 Sham-operated and 7 bile duet ligated rats. The
significant decrease of flow was noted in jaundiced animals
(54.47+4.02 ml/min/100g remus 93.91+7.68ml/min/100g,
p<0.05 in Sham'operated rats) on the 6th day after
operation. The influence of. E.Coli endotoxin LPS B4 on the
liver tissue flow was studied by means of its injection into the
portal vein or into the lumen of upper parts ofgut. Injection of
0.Sxl0g LPS into the portal vein of Sham-operated rats
led to the decrease of a liver tiue tlow to 37.66+1.96
ml/min/100g on the 60th minute from injection (n=7). In the
bile duct ligated group (n=7) the same procedure lowered
tissue flow to 27.18+1.41 ml/min/100g and 4of7 animals
died. We noticed no significant change in a fiver tissue flow
after an injection of 2xlO g LPS into the lumen ofthe gut
during 3 hours study. On the 3d day after a bile duct ligation
(fiver tissue flow was 67.12+1.01 ml/min/lOOg) an injection
of the same quantity of LPS into the lumen of the gut
decreased the fiver tissue flow to 28.98+2.85 ml//min/lOOg
and 3 animals of 7 died during 3 hours. Our investigation
demonstrated that at obstructive jaundice a situation appears
when absorbtion of endotoxin in gut significantly
increases and leads to the considerable disorder of liver
microcirculation.
MANAGEMENT OF INTRABILIARY RUPTURE OF LIVER HYDATIDOSIS
A. Alanolu, S. Ozkan, O, Alabaz, FC Ozkan
Department of Surgery, University of eukurova, Adana, Turkey
Rupture into the biliary 'stem is a major complication of multivesicular
hydatid cysts in the liver. Groxvth of e echinococcal cysts causes
displacement, distortion and stenosis ofthe hepatic ductules qth impaired bile
drainage. The hydatid cyst can rupture into the biliaD' tract due to long term..
compression causing biliary colic, obstructive jaundice and possibly liver
abscess. During the last 11 3'ears period, 131 cases fith liver hydatidosis were
treated at Department of urgery, School of Medicine, eukurova University,
14(11%) of them had biliary fistulae. Six(4,5 %) of these fistulae had
choledochal communications. There were 10 female and male, median age
was 40 at admission. Secondary bacterial infection xvas the most common
complication in our cases( 60 %). Median hospital stay 18 days. After removal
of the ecchinococcus cyst, obliterating the biliary openings within the cax4ty
combined xfith(6 cases) or xqthout T-tube drainage xas performed all of the
cases. According to the thickness of the ectocyst xall, size of the cavity
severity of the infection and degree of bile leakage, one of the folloxng
procedures of obliteration can be performed 1. Closure by im,ersion suture of
ectocyst, 2.Omental flap obliteration, 3. Closed catheter drainage.
151
P3!8 P319
HUMAN BILE DUCT CONTRACTILE ACTIVITY
Alexander B*., Stamford I.F*., Steger AC*., Bishai P*., Barr
A.,+ +Heaton N+., Howard E.R*., Benjamin I.S*.
Departments of Surgery*,Transplantation+and Histopathology+ +,
King's College School of Medicine and Dentistry, The Rayne
Institute, 123 Coldharbour lane, London SE5 9NU.
Responses ofhuman common bile ducts to pharmacological agents,
active on the gastrointestinal tract, were studied to determine the
possible relevance to biliary dyskinesia. Seventeen common bile
duets (donor 8, explanted 9) from liver..s of transplant patients; 4
abnormal common bile ducts (3 biliary dyskinesia, obstruction)
were also studied. Common bile duet segments were mounted in
organ baths to measure isotonic (12) and isometric (5) contraction
and exposed to a range of pharmacological agonists. Spontaneous
bursts of myoelectrical activity (1-4 min") were observed in 18/21
common bale duct specimens independent of the pharmacological
agents tested. No common bile duct responses were .obtained to
any pharmacological agonists usedin 20/21 specimens. Relaxations
to sodium nitroprusside and aeetylcholine and contractions to nor-
adrenaline were obtained in one specimen 0.75cm away from the
Sphincter ofOddi. Common bile duct morphology from explanted
and donor livers appeared normal with scanty intermixed muscle
fibres and dense connective tissue located within the walls.
Mucosal inflammation was observed in the 4 abnormal common
bile ducts. No other reports of spontaneous myoelectdcal activity
in the common bile duct have been reported in man.
BILIO-ENTERC ANASTOMOSIS WZTH THE BLUMGART'S
TECHNIQUE: THE NAPOLI EXPERIENCE
L- Aloe, H. Ansalone, G. Aragiusto, M. Montanaro,
I. Damiano, L. Vicenzo. Dipartimento di Emergeh-
za. Settore Chirurgia Epatobiliare in Urgenza.
Ospedale Cardarelli. Napoli. Italy.
Surgical relief of biliary obstruction whether
benign malignant still poses considerable
technical problems and this may favour the choice
of non-surgical options.
Since 1990 have performed bilio-enteric
anastomosis usingthe Blumgart's technique (S.G.&
O. 1982, 154:885-887; Br.3.Surg. 1984, 71: 257-
261) in 49 cases. Thertyone patients male
and 18 female, age range 41-85 years. General
status was assessed for all patients Excellent
(3), Good (13), Fair (27) and Poor (6). Fifteen
patients were affected by CBD stones, 7 by benign
stricture and 27 by malignant obstruction. Chole-
dochoduodenostomy performed in 16 cases and
Roux loop hepaticojejunostomy in 33: (16 at
common hepatic dbct; 7 in the confluence
after focalresection; at tight'hepatic duct; 4
at left hepatic duct; 5 at segment III duct). In
case the anastomosis associated with Whip-
pie operation and in 2 cases with major liver
resections. Five patients (10.2) died postoper-
atively. However 3/6 were in the "poor" general
status group while 2/43 in the other groups
(P=0.036). Five patients (I0.2) developed post-
operabive complications such cholangitis (I),
bliary fistula (2), wound infection (2). We
conclude that the reported technique has demon-
strated to be safe and cuick-to-perform and
has been associated with acceptable morbidity-
mortality rates in series. Its availability
should be taken into a:=ount when selecting
patients for surgical not-surgical biliary
decompression.
P320 P321
INTRAHEPATIC CALCULI FORMATION FOLLOWING
EXCISION OF A CHOLEDOCHAL CYST
...H. Ando, K. Kaneko, F. Ito, T. Seo, T. Ito
Department of Surgery, Branch Hospital, University of Nagoya
School of Medicine, Nagoya, Japan
Formation of intrahepatic calculi is one of the major late
complications following excision of a choledochal cyst. There are
only few studies, however, dealing this complication. It is
general belief that the main cause of intrahepatic calculi is an
anastomotic stricture. We report our experience with eight
patients who had intrahepatic calculi following excision of
choledochal cyst.
In order to clarify the cause of intrahepatic calculi, seven
patients underwent cholangioscopy and direct visual inspection
during surgery, and one patient underwent percutaneous
transhepatic cholangioscopy. Intrahepatic bile was cultured, and
calculi were analyzed.
Stenoses of intrahepatic bile duct (membranous and septal)
were demonstrated near the hepatic hilum in all patients. Calculi
were always located at the hepatic side of the stenoses. No
anastomotic strictures were noted at hepaticojejunostomy. The
calculi contained mainly calcium bilirubinate. Escherichia coli,
Klebsiellapneumoniae were cultured from the bile.
Stenoses of the intrahepatic bile ducts were demonstrated in
all eight patients, which were considered to be the primary cause of
the intrahepatic calculi following excision of the choledochal cyst.
152
MECHANICAL SUTURE BY THE APPARATUS
SPP-2 0 IN BILIARY SURGERY
U.A. Aripov, N.U.Aripova
Department of Faeultative Surgery of the 1-Tashkent Medical Institute,
Tashkent, Uzbekistan.
Our evidence ofthe mechanical suture's performing by the apparatus SPP-20 on
the biliary system with 408 patients displayed additional to above mentioned its
advantages. 1. Performance ofthe transduodeni papillesphincteroplasty (TDPSP)
by the traditional method of Archibald did not prove to be applicable due to the
traumatic property and the complication of the intervention's tehnic. We
modificated the apparatus SPP-20 and created the fixative clamp to lead out the
big duodenal nipple to the duodenotomic injury; the using of this'fixative clamp
significantly simplifies the technic of TDPSP and makes it unnecessary to be
additionally operated on choledochotomy.2. After the supraduodenic (SD)
choledochoduodenoanastomosis (ChDA) the developed underanastomosis "blind
bag"ofeholedoch sometimes is reason ofcholangitis and pancreatitis; as well as
presence ofthe rotary deformation ofthe duodenum and the stretch in the area of
anastomosis (especially, during performance ofthe wide anastomosis) are the risk
factors in the development of its failure and dyseenezia of the duodenum. To
remove the above montioned shortcomings ofthe SD ChDA we created more
effective method of forming the retroduodenic RD ChDA with main
requirements being preseved releavant traumatic property and simplificate of
making process. Upon the using ofmodificated apparatus SPP 20 we performed
TDPSP 257, RDChDA 96, double internal drain of the eholedoch 48,
transduodenic cutting ofthe contractive ChDA and papillectomy 2. Specific
post operative complications were detected with 17 4,2 % patients acute
pancreatitis with 5 patients, bile expiration ones, cholangitis with 4 ones,
acute hepatic renal insufficiency with ones, bleeding with 2 ones. Of the
total number patients died 1,2% ). Distant results up to 10 years had been
studied with 282 patients. Good results were detected with 263 patients,
satisfactory with 31 ones, poor results with 8 patients. Residual stone of the
choledoeh resulted in poor results with patients, as well as restenosis of big
nipple of the duodenum. This group of 7 patients had to undergo the operation
again 2 patients with restenosis ofthe big nipple ofduodenum and 4 patients with
residual choledocholithiasis had been made ChDA by method, with we created.
Retrograde endoscopic lithoextraction was made with one patient with residual
choledocholithiasis. One patient with restenosis ofthe big nipple ofthe duodenum
refused to undergo reoperation. So, the low rate of lethality, as well as different
specific complications, typical for traditional operational technic on the biliary
system by mannual suture both in immediate post-operative period and in distant
P322 P323
INTRADUCTAL ULTRASOUND IN THE PREOPERATIVE
STAGING OF PANCREATOBILIARY CARCINOMA
S.Asahara J.Ariyama, M.Suyama and K.Sato
Department of Gastroenterology, Juntendo University. Tokyo 11"3, Japan
Objective To determine the feasibility of using intraductal
ultrasound for preoperative staging of pancreatobiliary carcinoma.
Subjects and methods Intraductal ultrasound (IDUS) was
performed in 14 patients using FUJINON SP501 with 20MHz. These
included 9 bile duct carcinomas, 3 pancreatic carcinomas and 2
carcinomas of the papilla of Vater. IDUS was performed using
trasnhepatic biliary drainage route. In all patients tumor was resected
and histology and ultrasound findings were compared.
Results:In IDUS normal bile duct was visualized as having two layer
structures. The first hypoechoic layer from the lumen corresponded to
the mucosa and fibromuscle layer, and second hyperechoic layer
corresponded to the subserosa and pedcholedochal fat tissue. Bile
duct carcinomas appeared as hypoehoic mass rel.ative to adjacent
structures. Advanced bile duct carcinomas were readily diagnosed due
to destruction of external hyperechoiclayer. However, was difficult to
differentiate fibrosis of the duct wall from carcinoma. Submucosal
tumor extension was clearly depicted as hypoechoic mass. Accuracy of
depth invasion of bile duct carcinomas was 88.9%, of invasion to the
pancreas-and duodenum was 100%. Diagnosis of common bile duct
invasion of carcinomas of the pancreas and papilla of Vater was
achieved in 100%.
Conclusion IDUS may be a clinically useful method for preoperative
staging of pancreatobiliary carcinomas.
PERCUTANEOUS BILIARY DESOBSTRUCTION
D. Azoulay, D. Castaing, H. Bismuth
Hepatobiliary Surgery and Liver Transplantation Center, Paul
Brousse Hospital, 94800 Villejuif, France
Biliarylithiasis (particularely retained stones in the main bile duct and
intra hepatic stones) may be treated percutaneously obviating
reoperation. We report our experience with 100 cases of biliary
stones treated percutaneously between 1980 and 1995. The 100
patients were classified into 3 groups: Group A: gallbadder stones
(12 cases), Group B" main bile duct stones (35 cases), Group C:
intrahepatic stones (53 cases). The means to clear the bile ducts
included percutaneous endoscopy and contact lithotripsy. After
3.3:L-0.6 courses, desobstruction was complete in 75 cases (75%):
group A 10/12, group B 26/35, group C 39/53 and partial in 13
cases (13%) allowing to optimize the patient for curative surgery.
Desobstruction failed in 12 cases (12%) successfully treated by
surgery. Morbidity included 5 severe complications (5%) and one
patient (1%) died from angiocholitis.
Percutaneous desobstruction of the bile ducts may be proposed as a
priority in patients with a biliary drain in place and when
sphincterotomy is impossible or contraindicated. These manoeuvers
have a definitive place in the armamentarium of hepato-biliary
surgery.
P324 P325
GALLBLADDER CONTRACTION AFTER PARTIAL GASTRIC
RESECTION
M.R. Bandini, A.Pezzolla, M.A. Filograna, M.Buonfantino, I. Ugenti.
G. Fabiuno.
Clinica Chirurgica Universit di Bad Dir Prof. M. Fersini
Are increased incidence ofgallstones hasthere repartered in patients who
haveundergonepartial gastrectomy,but little isknowereaboutthepatho-
phisiologic mecehanism. In general, variants factors are involved in the
pathogenesis ofgallstones. Recentlymuchattentionhasbeendrawntothe
role of gallbbladder motility and cholecystokinin (CCK) secretionin the
formations ofgallstones. Gallbladderfunction is suggestedto etange aler
gastric surgery Baxter, Fagerber ). In several studies the plasma CCK
response aler oral ingestion of meal was found to be significantly
increased in .patients who have undergone partial gastrectomy when
comparedwith normal subjects Hofinan, Inoue). Itisnotknownwhether
increasedpostprandial CCKsecretiongallbladderemptyingcontributeto
the formationofgallstone inpatiens partial gastrectomyFewstudies have
beencarriedouttoinvestigateCCKsecretion,gallbladdercontraction,and
theirrelationshipinpatientswhohaveundergonepartialgastrectomyafter
excluding the effectofgasticemptying. We have investigate the intestinal
phase ofCCK.secretion and gallbladder contraction in these patients by
administration ofthe stimulus directly in to intestine In the last years 70
patientswhohadundergonepartialgastrectomywerestudied. Allhadbeen
operatedonbecauseofpepticulcer, to23yearsbefore.Elevenpatientshad
Billroth anastomosis, 44 ofthem uderwent surgical therapy for gastric
ulcer and 16 for duodenal ulcer. Gallbladder emptying was measured by
ultrasonography. Corn oil, a powerful stimulus for CCK release and
gallbladdercontraction, was administered throughthe small tube into the
duodenum or the efferent limb in a dose of 60 ml. within 2,3 minutes.
Gallbladderemptying,ultrasonographystudied,wassignificantly reduced
at 10 minutes in Billroth patients and from 15 trough 30 minutes in
Billroth II patients, compared with normal subjects. In the stimulation of
digestive functions after feeding cephalic, gastric, intestinal and post-
absorptivephasescanbedefined. Allthoughthereisatemporal relationship,
between these phases due to the aboral progression of food, trough the
digestivetract, thesephasesdoinfactoverlap intimeandprobablyinteract
with one another,
SURGICAL TREATMENT OF THE GALLBLADDER CARCINOMA.
Pawel Blalek, Zbignlew Biejat, Halina Makowska, Pawel Konrad, Jerzy A. Polafisld
3 rd Dept. of Surg. 2 nd Faculty of Mad. Warsaw Mad. School, Czemlakowski Hospital,
Stpifiska 19/25, Warsaw, Poland
Between 1989-1995 28 patients with gallbladder carcinoma were admitted to our
Department. Surgical procedures were limited to laparctomy in 5 cases. 12 patients had
palatlv6 operations lavra urd'va tlm e, month,'. 11 ca wr6 trcat by xtndd
cholecystectomy Icholecystectomy plus bisegmentectomyn ofthe liver and lymphadenectomy/.
All patients had invoivment of serosal layer, and only two ofthem were with no regional
invasion. Average survival time 15 months but 5 patients are still alive.
The major route of spread ofthe gallbladder carcinoma is local or regional seldom distant.
The main role in regional spread play lymphnodee metastases.
We performed common bile duct, ratroportal, retropencreatic, celiac.and interaortocaval
nodes excisions with pathological exams. Survival ofthe patients following extended
cholecystectomy was correlated with the presence or absence of lymphatic invasion. In
expedenca nodal metastases to celiac or interaortocaval nodes are contraindication to
curative resection.
4 patients had local invasion to the colon/3/and pancreas111. They underwent curative
resection with hemicolectomy or pencreatoduodenectomy. All ofthem died in postoperative
pedod.
The optimal surgical treatment for carcinoma ofthe gallbladder remains unclear. We think
that operative procedure should depend on lymph nodes invasion. Cases with local Invasion
to adjacent organs should be qualified to peliative non curative procedures.
153
P326 P327
rtly, PANCR].TITIS
K.BICKI, KOZ[CKI
Deprtment of C.era]. , Mdica]. Cn:'.: fbr Pcxtaduate
k4ucat:.:ion,
Acu necrot:.fzi..., pancre..atitis /NPt' .is iz. e of fac.
in etiolo of [i bi!.i. si.c:::u /BV. ver, ]y
st.. ee r' in the a.i.!able ]..i.et.
i.stitution, in 0f 1( mtihs with E, tb eti.olic factor
tatic is /I/. tihs fe
f 4istFict i.ta].s, e 4i d ta si-
ly."tic of di]. t of I bile t"
//, latem t by exte bi].i di.e,
ri ti. th t;i it
it of i. y i inti. opi d.
tri.ti. srt. th of th t si t of
by of s i.. ir / zir ti./. atkt
of ic ie [Dth ti, with ..y e
i s]; o i e bili .
tmtive e ].it. t t].]N .ti
si d. di i%al %r 3 .initi
tti. of t , t of t litis,
tit tijej]. R Y tis
te ults: to , t tive foll ri.
3,5 d. 2,5 , ti].y, wi t ti sis
d. st of ].itis vi the liar ft.i
u].. ...( of tl.ts eibits
of" i atic ici iit dit
lli.t.
lis tial risk of s. bile t
d sict. Fic bili tt ..e y ve a le
in lioti t , of .Si.
r ].ete v f
LIVER RESECTION: A TREATMENT OF CHOICE IN
PATIENTS WITH PRIMARY LOCALIZED INTRAHEPATIC
GALLSTONES.
G. Borgonovo, C. Vons, C. Smadja, D. Grange, D. Franco.
Services de Chirurgie, H6pital Antoine B6cl6re, Clamart, et H6pital
Louise Michel, Evry, Universite Paris XI, France.
Liver resection or percutaneous dilatation of bile ducts and stones
extraction have been diversely advocated in the treatment of
intrahepatic gallstones. The purpose of this work was to analyze the
results of 21 consecutive patients with primary localized intrahepatic
stones treated by liver resection. There were 12 females and 9 males
with a mean age of 44 years (range 20 to 71 years). All patients
had relentless cholangitis. Sixteen patients had had, prior to liver
resection, an average number of 2.25 therapeutic procedures
(range to 5) followed by recurrent cholangitis. There were 7 left
lateral lobectomies, 7 left hepatectomies, and 7 right hepatectomies.
There were no operative death. Four patients (20%) experienced
postoperative complications: 2 subphrenic abcesses and 2
subphrenic hematomas. The rate of subphrenic abcesses was
significantly lower in 15 patients receiving prophylactic
antibiotherapy (0%) than in the 6 other patients (33%). The mean
follow-up was 55 months. All patients but two were free of biliary
symptoms and postoperative imaging confirmed the absence of
stones in the remaining liver. Two patients had recurrent cholangitis
resulting from complications of biliary procedures performed before
the diagnosis of intrahepatic stones: stenosis of a hepatico-
jejunostomy treated by re-anastomosis and postsphincterotomy
stenosis of the papilla treated by repeated endoscopic
sphincterotomy. Cholangitis disappeared thereafter in both patients.
These results suggest that liver resection is a very efficient treatment
in patients with primary localized intrahepatic gallstones.
P328 P329
LAPAROSCOPIC SUBTOTAL CHOLECYSTECTOMY FOR THE
"DIFFICULT" GALLBLADDER.
PC Bornman K Michelowski, JEJ Krige, PJ Gallagher, Terblanche.
Department of Surgery, University of Cape Town, South Africa.
Dissection of Calot's triangle can be hazardous in the presence of severe
inflammation of the gallbladder (GB) or cirrhosis with portal
hypertension. Open subtotal cholecystectomy has proven to be a safe,
simple and definitive procedure in this setting (Ref). This study reviews
the outcome of laparoscopic subtotal cholecystectomy (LSC) performed
in 30 patients (mean age: 53 yrs; 22 females, 8 males) over a 23. month
period (January 1994 November 1995). These patients constitute 8.8%
of the total number of laparoscopic cholecystectomy (N 340) and 16%
of patients with acute cholecystitis (N 186). Eighteen in the later group
were converted to open cholecystectomy. Indications for LSC was acute
cholecystitis/empyema (N=23) severe fibrosis (N=6) and cirrhosis with
portal hypertension (N= 1). The cystic duct and artery were isolated by
blunt dissection and clipped before division when possible. When this
was not feasible the GB was divided at the junction of the cystic duct
with Hartman's pouch and then tied with an Endoloop (Ethicon), or
suturing (2 cases). Initial standard dissection of the GB was attempted but
converted to the subtotal modification if no dissection plane or bleeding
difficulties were encountered. The GB was entered and the wall was
divided along the junction with the liver using diathermy and clips,
leaving a disc of posterior wall in situ. Gallstone recovery and irrigation
were meticulously performed. A portovac drain was inserted in 14 cases.
Median operation time was 73 minutes (range: 45-130). There was one
death due to a myocardial infarction. There were 5 local complications
(1 self-limiting bile leak, 4 subhepatic collections of which 2 required
percutaneous drainage) and 9 respiratory infections. Median hospital
stay was 5 days (range 2-28 days). LSC is a safe, relatively simple and
definitive operation for removal of the difficult gallbladder. In most
situations this technique avoids the need for open conversion or
cholecystectomy particularly in the high risk patient and avoids potential
injury to the CBD in complex cases.
Ref. Bornman PC, Terblanche Surgery 1985; 98: 1-6.
154
Return to work after cholecystectomy:
results of laparoscopic versus open surgery
D. Borzomati. G.Costamagna, G.B. Doglietto, M. Buononato, A. Tringali,
M. Mrciano, F. Crucitti.
lstituto di Clinica Chirurgica Universita' Cattolica de] S. Cuore,
L.go A. Gemelli 8, 00168 Roma.
A significant decrease of postoperative pain and an early return to normal
alimentary habits make up some of the main advantages provided by
Laparoscopic Cholecystectomy (LC). Some authors pointed out an earlier
retum to work for these patients compared to patients undergoing Open
Cholecystectomy (OC).We evaluated, by a self-administered postal
questionnaire, the time passed between surgery and the return to work of
200 LC patients (59 M, 141 F, median age 49 years) and 200 OC patients
(83 M, 117 F; median age 53 years) who underwent surgery from July
1990 through May 1994. Three hundreds and sixteen patients (79%)
retumed the completed questionnaire (173 LC (86.5%) and 140 OC
(70%)). 36.4% (n=63) and 7.1% (n=10) of the LC and OC patients
referred a return to work within 10 days from surgery (p< 0.0001). Return
to work beyond 45 days from surgery occurred respectively in 4% (n=7)
and 25.7% (n=36) of the two groups of patients (p< 0.0001). The data-
distribution statistical analysis showed that half of LC and OC patients
resumed their professional activity within 15 and 30 days from surgery
respectively. The results of our study warrant that LC shows in comparison
with OC a shorter convalescence time-and an earlier retum to full
activities. These figures may imply a significant decrease of social secunty
and insurance costs.
Rrn to wor, after cholecystectomy: patients'dmtribufio.;
30.6%
20
3. -:"
10
1L%
li'6 i" 3.
10;8%:'='-
'"'--"
wi wimin within witn witn wimin beyond
days 10 days 15 days 20 days 30 days 45 days 45 days
B Laparoscopic Cholecystectomy en clecystectomy
P330 P331
MANAGEMENT OF COMMOM BILE DUCT STONES'EVOLVE IN THE
LAPAROSCOPIC SURGERY ERA
R.BRACCO (F.A.CoS.),J.FRARACCIO(F.A.C.S.),R.JURY, A.
MICHET?'I. SERVICE OF SURGERY,CLINICA PUEYRREDON,
MAR DEL PLATA, ARGENTINA.
A retrospective study was carried out on 386 laparosco-
pic cholecystectomies(LC)concerning the management of
common bile duct stones(CBDS).Initially while expertise
in LC was achieved we didn't perform routine intraopera__
tire cho]angiography (IOC) and preoperative endoscopic
retrograde cho]angiopancreatography(ERCP) was indicated
in selected patients with predictive factors suggesting
CBDS'(Jaundice,high serum level of bilirrubin and alkali
ne p.hosphatase and ultrasonographic bile ducts dilata-
tion).Later,when skills in handling the cystic duct we
re developed IOC became a routine procedure as we used
to in the preaparoscopic .surgery era.Actually we have
started doing laparoscopic transcystic exploration of
the.CBD when unexpected stones are found in the IOC.
Summar of results
I) Patientssuspected of carrying CBDS'I5 on 386 LC
3,88%. (By ERCP).
2) Patients not suspected of carrying CBDS'4 on iii
IOC 3,63% (treated by transcystic drain+postopera_
tive endoscopic sphinterotomy(ES).
3) Patients with unknown retained stones'5 on 386 LC
1,29% (treated by ERCP and open CBDS removal.
Conclusions l)Preoperative ERCP is,in our group,the
standard procedure for higlhly suspected carriers of
CBDS.2)Routine IOC allows the recognition of unsuspec-
ted CBDS and acquiring skills for handling the cystic
duct for further laparoscopic exploration.3)The manage__
ment of CBDS remains controversial according to:a)expe_
rtise in LC and use of routine lOC.b)availability of
experienced, bil iary endoscopist.
EXTRACORPOREAL LITHOTRIPSY AND SPHINCTEROTOMY IN
TREATMENT IN PATIENTS WITH CHOLEDOLITHIASlS.
B.S.Briskin, A.E.Ivanov, V.P.Ivlev
Moscow Medical Institute, Department of Surgery, Russia
27 patients with choledocholithiasis xvere managed in our department
aged 50-90 years [11 patients older than 80 years of age]. We designed a
technique combining extracorporeal lithotripsy with endoscopic operations
on the major duodenal papilla. All the patients had had jaundice/bilirrubin
90,5-196,9 mmol/l. 14 patients was underwent cholecystectomy earlier. 7
patients had parapapillary diverticulums.The number of gallbladder stones
were from to 15. Stone diameter in choledochus was 17-45 mm. As a
first stage of treatment, was performedsuccessfull endoscopic
sphincterotomy 2-7 days after the admission. 2 patients was performed
microcholecystostomy under ultrasound guide for decompression of biliary
tract. Nasobiliary drain was used in 5 patients after the endoscopic
sphincterotomy to prevent the .blockade offhe terminal part of choledochus
and create the optimal conditions forevacuation of fragments of stones. We
utilized the Dornier Lithotripter Compact. Choice the position and
efficiency control we carry out under ultrasound and X-ray guide. Good
results shoxved a maximal stone size of 6 mm in diameter. Therapy was
continued when the fragments of stone were less than 6 mm in diameter [7
patients with numerous and big stones]. After successful treatment almost
all patients became stone free. Only one patient was underwent
choledocholithotomy.
After extracorporeal lithotripsy we observed a transient diastasuria in
5 patients, microhematuria in 5 patients, acute pancreatitis in 4 patients.
Complete clearance was obtained in all patients after one or more
procedures before discharge.
No mortality was reported. Mean hospital-stay was 22.
In conclusion, combined extracorporeal lithotripsy and endoscopic
sanation of choledochus seems to be an effective method of treatment in
elderly patients with choledocholithiasis.
P332 P333
ROKI'FANSKY-ASCHOFF SINUSES OF THE GAI.,I.,BLAI)I)liR CAN BE TI-IE
INITIAL SITE OF FORMATION OF SOME TYPE OF BLACK PIGMENF
GALLSTONES. A STUDY BY SCANNING ELECTRON MICROSCOPY.
A. Cariati, *F. Cetta, **P. Romano, A. Costao, A. Casano, **D. Zaccheo. Institute of
Surgical Clinics, University of Genoa and '*Siena. **institute of Human Anatomy,
University ofGenoa, Italy.
Introduction. Black pigment stones (BS) ma,inly consist of glassy of bilirubin
polymers and frequently found in patients with hemolysis, with cirrhosis, chronic
hepatitis in patients with previous gastrectomy. We have recently documented that
black microstones both in the hunen and in the Rokitanskv-Aschoff (R-A)
sinuses of patients with adenomyomatosis (ADM) of the gallbladder (GB) (!). Material
and Method. During the prospective stndy of 168 consecutive patients who had
systematic stone and bile analysis and histologic examination of GB wall at the
University ofGenoa, ADM, was found in 54 patients (32 %). The 54 ADM patients had
gallstones, which black (alone or in association with other stone) in 32 patients (59
%). 21 ofthese patients kad BS unique stones; the other patients had BS associated
with single cholesterol (n= 7) multiple mixed (n= 4) stones. While 12 patients BS as
nnique stones associated with the typical risk lhctor lbr BS, there patients
who unrelated to the typical risk factors for 13S. 7 of these patients, with age
of 51 years (range 43-72) had black intraparietal and intrahuuinal microstones. The
residual 2, with mean age of 35.5 years (range 34-41) had spicular I3S in the main
Inmen ofGB but not within evident R-A sinuses. In patients with ADM and BS (4 with
intraparietal and intraluminal BS and with spicular BS in the main GB hunch)
scanning electron microscopy (SEM) analysis of stones and gallbladder specimens
performed. Results. SEM analysis ofGB specimens has demonstrated: 1) the presence of
black microstones within the R-A ginuses of GB; 2) the microstructure of these
microstones (prevalence. of granules of calcium bilirubinate). SEM analysis of black
pigment gallstones showed the prevalence of glassy of bilintbinate at the
periphery and of granules of bilirubnate in the center of non spicular BS and the
presence of calcium bilirubinate, phosphate atfd carbonate in spicular and coraMike BS.
Conluiom. It is suggested that: (i) R-A sinuses .of the GB can be the initial site of
lbnnation of BS, in partichlar of non spicular subtype; (ii) in black pigment microstones
found within the R-A sinuses ealcimn bilirubinate is fonnd mainly in clustered state. This
treud is reversed in the periphery of BS lbund in GB main lumen, where calcium
bilirubinate is found in glassy masses; (iii) non spicular BS start from core of
bilirubinate granules, extruded in the main GB hunen, while becoming surrounded by
black pigment material in later stage.
!) F Cetta, A Cariati et al Gastroenterol. 1993; 104: A 353.
155
COMPLICATIONS OF DIAGNOSTIC AND THERAPEUTIC
ERCP A PROSPECTIVE STUDY. D.L.Carr-Locke J.Vandervoort,
T.C.K.Tham, R.C.K.Wong, A.D.Roston, A.Slivka, A.P.Ferrari Jr.,
M.Hughes*, D.R.Liehtenstein, J.Van Dam. Division of Gastroenterology,
Harvard Medical School and School ofPublic Health*, Boston, MA.
The incidence ofpost-ERCP complications has been derived mainly from
retrospective studies which underestimates its incidence.
AIM To determine the incidence of complications following diagnostic
and therapeutic ERCP in a single center. METHODS Data on ERCP-
complications in 817 i3atients were prospectively entered ha a database.
Definition of complications panereatitis abdominal pain and fourfold
elevation in amylase and/or lipase after 24 hours resulting in prolongation
of admission; bleeding Hgb drop of >2g/dl and the need for endoscopic
hemostasis; cholangitis fever, chills, elevated liverenzymes and positive
blood culture within 48 hours of the procedure; hypoxia O2-saturation
<90% for 2 minutes; hypotension systolic blood pressure <90mmHg for
2 minutes; bradycardia heart rate <50bpm for 2 minutes.
RESULTS Ofthe 817 ERCPs, 47.6% were diagnostic and 52.4% were
therapeutic. The overall complication rate was 80/817 (9.8%).
Complication Diagnostic (389) Therapeutic (428) Total (817)
Panereatitis 27 (7.0%) 36 (8.5%) 63 (7.7%)
Bleeding 0 (0%) 6 (1.4%) 6 (0.7%)
Cholangitis 0 (0%) 4 (0.9%) 4 (0.48%)
nypoxia (0.25%) 2 (0.47%) 3 (0.36%)
Hypotension &/or (0.25%) 3 (0.71%) 4 (0.48%)
bradycardia
TOTALS 29 (7.4%) 51 (12%) 80 (9.8%)
CONCLUSION The higher risk ofpost-ERCP complications after
therapeutic ERCP is mainly caused by eholangitis and post-
sphincterotomy bleeding. The incidence ofpanereatitis is the same for
therapeutic and diagnostic ERCP.
P334 P335
BILE TRACT STRICTURE PRECEDES NOT FOLLOWS THE OCCURRENCE OF
PRIMARYHEPATOLITHIASIS.
CettaF, *Cariati A, Lombardo F, Baldi F, GiubboliniM, Bardlini L.
InstofSurgClinics, Univ. ofSiena. *InstofSueg. AnatomyUniv. ofGenog
Dudng the study of 1740 consectaive patients, who underwent surgery for bile
truer disease and who had syslematic stone (GS) md bile analysis and
comtmmon of the content (bile and GS) to the container (gallbladder and bile
duct wall we observed 18 patients with primary lilhiasis (HL) in a 16-
choledocholithia-6s, 7 had HL utter previous operation on the Iract. Two
patients, both female, (aged 57 and 61) had HL limited to some liver segments,
by sectorial Caroli's diseases. Bothhadcholesterol (Ch) GS. In the other,
13 patients GS were brown in 8 cases, Ch or mixed in 4, and black pigment in
case. The .last 3 patients had exnrgsite stones, i.e., different stone populations in
the same patient), always including some brown GS. Therefore, brown GS were
found in 11 patients (61.1%), but were foundas unique stones in only 44.4
% of patients with HL. Present findings have 2 peedian'ties: (i) they were
collected chaing a large prospective study; (ii) eight of the 18 patients were.
included in the study at least twice. Therefore, they had repeaed X-ray
examinations together with bile arat GS analysis. This group of lients was
analyzed to detect the precta'soy role of stricture and bile stasis in the
pathogenesis ofHL. In this subgroup ofpatients it was shown that slicture and
bile stasiswere aprerequisite, not a consequence ofintrahqatic GS. In particular,
the following sequency was observed: 1) normal bile duets at the thne of
choleey; 2) postve stricture without stones; 3) presence of GS
wilhin the intrahapatic duets. In 3 ofthe patien with Ch, with mixed and
with black GS, stones also contained suture material. Therefor strkre at the
confluence of the bile ducts, aml bile duct dilatation certainly preceded, not
followed stone formation. In addition, it was shown, that: 1. Bile infectionwas (i)
conslant in patients with. brown intrahqatie GS, (ii) present, but not always, in
patients with composite GS, and (hi) absent in patients with Ch GS as unique
stones. Themean agewas42.2 years(range29-63)inpatientswith Ch HL, while
it was always grea than 50 in patients with brown GS as unique snes..It is
suggested that congenital or acquired abnormalities ofthe bile tract determine the
occurrence ofsevere ormoderate stricaa causing adelayedclearing ofthe bile.
Aoeording to the degree ofthe stricture, if GS form in the tirst decades in the
absenee of bacterial infection, they can be Ch or mixed; ff they form in the
elderly in the ce of bile infection by E.coli, they are almost invariably
brown pigmem stones. On the contrary, factors due to diet, or supersaturation of
thebilewith Ch seem oflittle importanceinthepathogenesis ofprimmyHL.
DIFFERENT HISTOTYPES OF GALLSTONES CARCINOMA ARE ASSOCIATED WITH
DIFFERENT RISK FACTORS AND ARE LIKELY TO BE DUE TO DIFFERENT GENETIC
ALTERATIONS.
CetmF, *Cwiati A, Lombardo F, Baldi F, Oiubbolini M, Bardlini L.
Inst ofSurgClinies, Univ. ofSiena. *InstofSurg. Anatomy Univ. ofGenoa.
Seventy-lh'ee consectaive patients with gallbladder carcinoma (GBC), 17 men
(M) and fi women (W), mean age 69,4 years, were found during the study of
2850 pattents, who underwent surgery for biliary tract diseases (GBC=2.59% of
all patients). Sixty-nine had gallstones (GS). Patients were collected in 2 differen!
centers (49 outof1690 in the former mid 24 out of 1160 in the latter). Thirty-one
ofthesepatients (7 M, 26 W, mean age 68,7), who had complete removal ofthe
gallbladder (either by simple cholecystoeton or more radical operations) had
systematic_ bile and stone analysis. In partiadm', in addition to bile pH and bile
culture,, the bilia trypsin conlt was also determined, as a reliable marker of
pancte,afico-biliary reflux (PBR). Large stones were more frequmt in patients
with Naamous cell carcinoma (SQC) (n=8; all with GS>I5ram) than in patieats
with adeno (ADC) or poorly differentiated carcinoma (n=22) (15:GS>15 ram;
7: GS<lSmm). Time lapse ofGS in patients in whom this finding was available
also resulted significantly different: 16.3 years in patients with A1X2 (n=13) vs.
31.6 years in pients with SQC (n=6) (p=0.002) (1). In paraedar, 3 paents had
papillary tumors: 2 had papillary carcinoma (PAP) and papillary adenoma
with severe dysplasia. None ofthese patients had associat GS. Two ofthe 3
had increasedb'tliary trypsin values suggesting PBR. On thebasis ofpresent data,
itis suggestedthat PAP and SQC seem to be associated with different factors and
conditions. PAP is frequently associated with PBR, but seldom with GS. On. the
contrmy, SQC is more closely related to long-standing cholesterol or
combination stones and to risk factors affecting their formation (female sex,
parity, obesity orhigh fatdiet). Instead ofclassifying GBC as a unique entity, it is
suggeaed to separate carcinomas associated with. or related to GS from tho
without GS. Distinction between these 2 groups as well as between PAP and
SQC could be ofimportance for both epidemiologic and clinical purposes, i.e.,
for a proper recognition of the risk fairs, a knowledge of the natural
history of the illness and a con evaluation of the therapeutic options, in
particular, since eancer is due to biochemical alterations affecting cell genes, it is
likely that PAP and SQC are also due to different genetic mutations, as it has
recently been suggested for diffemn histotypes of esophageal, gastric or
pancreaticcancer. (1)CettaF, et al. Gastroenterology 104: A353; 1993.
P336 P337
BACTERIAL OVERGROWTH IS THE BASIC FACTOR FOR THE FORMATION OP
CLOGS OBSTRUCTING BILIARY ENDOPROSTHESES USED FOR ENDOSCOPIC
STENT[NGOFPATIENTSWITHCARCINOMASOFTHEPANCREASANDBILETRACT.
CettaF, Lombardo F, Baldi F, Chubbolini M, Barellini L, *Andreoli F.
Inst ofSurgClinics, Univ. ofSiena. *InstofSurg. Pathology, Univ. ofFlorence.
Data are reported concerning 15 patients, who had treatment ofnon resectable
tumours ofpancreas and bile tractby biliary endoprostheses. In all ofthe patients
bile samples were obtained for bile culture before the pl of the
endoprostheses. Bile culture of these samples resulted positive in 9 of the 15
patients (60%) (E.coli in 4 cases, Proteus in 3, Enterobact and Klebsiella in
case, respectively).
Prostheses were removed within an average interval of251 days (range 55-416
days) alter implantation. Deposits obstructing the endoprostheses were examined
by stereomicroscopy and scanning electron microscopy and analyzed
quantitatively by Fourier transform infrared spectroscopy. All the deposits
contained, in addition to small amounts of cholesterol, calehan palmitate and
bflirubina whichare the typical components ofbrown stone External deposits
(i.e., out ofthe prostheses) showed a higher bilirubinate palmitate ratio when
cowithinternal deposits, whichusuallyhad agreaterpalmitate content. In
particular, calcium palmitate was found uniformly in hundreds of light layers
altmmting with tan b'dirubinate layers throughout the entire cross section ofthe
clog Bile culture at removal of was positive in 100 % of cases,
usually showing apolymicrobicassociatiorL
It is suggeaed that material resulting in stent clogging has the same composition
as brown pigment stones and then also has the same pathogeaesis. In partiodar,
bile infection, as in brown stone pathogenesis, precedes, not follows the dogging
of the prostheses, and is the basic responsible for the formation of the brown
deposits, mainly by the action of beta glucuronidase and phospholipases, the
latter causing diffme 'tation ofpalmitate. Sphineterotomy1)
and placement
of a pmnment foreign body within the bile trad2)
are basic factors for bile
con.lmfinm'on and growth of in patients with previous sterile bile
However, in addition to the diameter of the prosthesis and other technical
pamnetets, factors related to the patient as age and types ofbacteria(3)
present in
the duodenum, also play a role in stent clogging, which has to be considered a
(1) Cetta F Amh Surg 128: 329-336; 1993. (2) Cetta F, Lombardo F, Rossi S
HPBSurgery6: 235-242; 1993. (3)CettaFArmSurg.213: 315-326; 1991.
156
SURGICAL MANAGEMENT OF TUMORS OF THE AMPULLA OF
VATER
B. Chareton, J. Coiffic, E. Bardaxoglou, S. Landen, J.P. Campion, 13
Launois
Department of Digestive Surgery and Transplant Unit, CHR
Pontchaillou, Rue Henri Le Guilloux, 35033 Rennes, France
M.aterials and methods Between, 1970 and 1992, 63 patients
underwent surgery for ampullary .tumors. The group comprised 33
males and 30 females with a mean age of 64.8 + 9.8 years. Surgical
procedures included subtotal duodenopancreatectomies (n 40), total
pancreatectomies (n 3), ampullectomies (n 8) and surgical bypass
or exploratory laparotomies (n 12). Resectability was 68%.
Pathology included 53 adenocarcinomas, undifferentiated lesion and
.9 benign lesions. According to the MARTIN staging criteria tumors
were classed as follows stage 7, stage II 11, stage III 14, stage
IV 21. All patients with stage I, II or III tumors underwent resection.
Among the stage IV patients, 11 were resected and 10 had bypass
procedures.
Results Mean hospital stay was 20.6 days. For the patients having
undergone subtotal duodenopancreatectomies, mean time of stay was
24.8 days (16.5 days when the postoperative course was
uncomplicated). Overall operative mortality was 12.7%, and 7.5% after
subtotal duodenopancreatectomy. Five-year survival for the entire
group was 40%. Five-year survival for stage through IV tumors was
85%, 65%, 44% and 8% respectively. For stage I, II, and III lesions,
survival was significantly better following subtotal
duodenopancreatectomy than after ampullectomy. For stage IVlesions,
and 2-year survival following subtotal duodenopancreatectomy and
surgical bypass was 70% and 25%, 20% and 0% respectively. We now
consider subtotal duodenopancreatectomy rather than ampullectomy as
the treatment of choice for benign ampullary lesions, having
reoperated, 2 patients with stage IV tumors who had undergone
ampullectomy for apparently benign lesions, 4 and 22 years
previously.
Conclusions Subtotal duodenopancreatectomy is the treatment of
Choice'for ampullary tumors, even when these are benign.
P338 P339
CHEMICAL COMPOSTION OF THE BILIARY STONES
STUDIES WITH INFRARED ABSORPTION SPECTROPHOTOMETRY
MC.Chen, YL. Chen, SJ. Kuo. ST. Chen HC. Chang
epartment of General Surger,Changhua Christian
Hospital Taiwan.
To make a comparison between the chemical
composition of the common bile duct stones and
intrahepatic stones, we analyzed every surgical
specimen of the biliary stones(135 from CBD
92 from IHD) between Jan 1993 and Aug 1995.
By use of the infrared absorption spectrophoto-
metric method, they were classified according
to their major chemical composition into 5
categories: 1.cholesterol stone 2.calcium
bilirubinate stone 3.calcium stearate stone
4.calci1m carbonate stone 5.calcium phosphate
stone. There was no gemder difference in the
mean age of each group, but the mean age of
IHD group (53. years) was younger than that of
the CBD group(64 years).It may be due to the
different mechanism of stone formation.
The relative incidence of hepatolithiasis in
this series was around 20%, it made no difference
with the average figure(3486/17182=20.3%,Su.1992)
reported in Taiwan.Calcium stearate stones were
mainly found in the IHD group, most of them were
muddy and fragile.
USE OF INFRARED ABSORPTION SPECTROPHOTOMETRY
IN GALLSTONES ANALYSIS
YL.Che, SJ. Kuo, ST.Chen, HC.Chang
Department of General surgery, Changhua
Christian Hospital, Taiwan.
The aim of this study was to investigate the
epidemiological characteristics of gallstone
disease in Changhua county, located in central
Taiwan. From Jan 1993 to Aug 1995, we analyzed
314 GB stones for its chemical composition
and classification by infrared absorption
spectrophotometric method. The were classified
according to their major chemical composition
into 5 categories: 1.cholesterol stone 2.
calcium bilirubinate stone 3.calcium stearate
stone 4.calcium carbonate stone 5.calcium
phosphate stone. In this study, we found that
the incidence of the cholesterol stone was 32%
it was lower than the 43 to 56% reported by
other hospitals in Taiwan. The reason is that
Changhua is primarily an agricultural county.
The pure cholesterol (> 98%)stones only
comprised 27 % of the GB stones in this series.
Becom+/-ng Westernished in our dietary patterns,
the occurrence of the pure cholesterol GB stones
will increase. The relative incidence of the
pure choesterol stone to other kinds of GB stones
was opposite to the age distribution in this
study.
P340 P341
RESULTS OF RECONSTRUCTION OF BENIGN BILE DUCT
STRICTURES
R. (olovir, D. Bilanovir, V. Kalezir, S. Matir, M. ukalo
Surgical Clinic of the Institute for Digestive Diseases, Clinical Center of
Serbia, Belgrade, Yugoslavia
Over period of 21 yearn(1974.-1994.) 103 patients were operated for benign bile duet
strictures. There were 66 (64.1%) women and 37 (35.9%) men. The average age was 50.2
years (ranging from 16 to 84 years). Two patients had congenital stricture, 6 stricture due
to chronic pancreatitis, posttranmatic and 94 postoperative bile duct stricture (82 after
cholecystectomy, 8 after distal gastrectomy for duodenal ulcer, and 4 after hydatide cyst
operations). According to Bismuth's classification, there were 23 (22.3 %) strictures of
type I, 28 (27.2 %) oftype II, 33 (32 %) oftype III and 19 (18.5 %) oftype IV. A number
ofcomplications were registered in our patients; intrahepatic lythiasis in 38, liver fibrosis
cirrhosis in 15, athrophy/hypertrophy complex in 7, liver abscesses in 10, external
biliary fistula in 12, biliodigestive fistulas in 5, colonic fistula in 2, gastrojejunal fistula in
1, incisional hernia in 9, supurative pericarditis in 1, retroperitoneal biloma in 1,
perihepatic, subphrenic absceses and billiary peritonitis in 1, respectively, portal vein
thrombosis in 1, and the number ofminor complications, like inadequate goux-en-Y in 35
patients and so on. The majority of patients had dense intraabdominal adhesions. In 69
patients to 6 previous attempts of reconstruction were performed elsewhere. In two
patients reconstruction was technically impossible due to portal vein thrombosis in and
to inaccessible bile ducts in patient. In 101 patients 102 reconstructions were carded out
(one patient was reoperated one year after previous reconstruction). In 96 patients
hepaticojejunostomy with 75 cm long Roux-en-Y, in choledochoduodenostomy, and
choledochoplasty (stricturoplasty) in were carded out. Three patients died in the early
postoperative period (two in whom the reconstruction was impossible, and one due to
worsening of previous renal insufficiency). Four patients died later, two due to variceal
bleeding, one due to myocardial infarction and one due to pancreatic carcinoma. Six
patents were lost from follow-up. The rest of 90 patients were followed up for 5.5 years
(ranging from 6 months to 21 years).
Good result of the reconstruction was achieved in 67 out of 90 reconstructed patients
(74.4 %), satisfactory in 21 (20.4 %), and unsatisfactory in 2 (1.94 %). We conclude that
satisfactory results ofreconstructions ofbenign bile duct strictures could be acheived in
majority ofpatients providing that the operation is not performed too late, and that it is
properly done.
INJURIES TO THE SEGMENTAL BILE DUCTS
R. (olovir, D. Bilanovir, S. Matir, V. Kalezir, M. gukalo
Surgical Clinic of the Institute for Digestive Diseases, Clinical Center of
Serbia, Belgrade, Yugoslavia
Surgically important variations ofthe segmental bile ducts ofthe right lobe of
the liver appear in about 15-35%. FrequenCy ofthe injury to these bile ducts is
not known, but it is believed to be more frequent then it is usually thought, as
the ligature of small bile duets may pass without serious consequences. The
treatment ofthese injuries is controversial.
We treated 10 patients; eight women and two men aged from 21 to 65 years
(average 42.5 years). In nine patients there was an injury of the seetoral or
segmental duct, while in one there was an injury ofthe Lusehka's bile duets. In
patients injury took place in our Institution, other patients were operated
elsewhere and later sent to us. Injury was recognized immediately in 4 patients
only, so an immediate repair was performed in patients, while a ligation of
the injured duet was done in patient. Four patients were operated for biliary
peritonitis with lavage and external drainage, three ofwhich developed exter-
nal biliary fistula that ceased spontaneously, while only one had to be reoper-
ated and an anastomosis between the injured duct with the Roux-en-Y jejunal
limb was performed. In two patients who developed an external biliary fistula
through the subhepatic drain reoperation was not necessary. A patient in whom
an injured duct was ligated, developed liver abscess and wound disruption and
subsequentlydied. Other 9 patients were followed up from months to 15.5
years, average 3.5 years, and all are symptom-free.
We conclude that the management ofthese injuries depends upon the size ofan
injured duct, time ofrecognition ofthe lesion and eventual complications.
157
P342 P343
BENING STENOSIS OF BILIO-DIGESTIVE ANASTOMOSES:
PERCUTANEOUS TREATMENT WITH BILIOPLASTY
A.R. Cotroneo, C. Di Stasi, P. Bertolino, R. Manfredi, G.
Costamagna*, P. Marano, F. Crucitti*.
Department of Radiology and Surgery*, Universith Cattolica del
Sacro Cuore, Policlinico "A. Gemelli", Rome, Italy.
PURPOSE: 2'./ of the patients undergoing cholecistectomy present
iatrogenic complications to the biliary tree, that are treated with bilio-
digestive anastomosis. Many ofthese anastomoses evolve in a benign
stenosis. The aim of our study is to evaluate the percentage of
success and the complication rate of percutaneous treatment of
anastomotic stenoses with bilioplasty.
MATERIALS AND METHODS: 29 patients with recurrent
cholangitis and subsequently treated with bilio-digestive anastomosis
were included in the study. At time of presentation they hd an
in6rease in ,Gt and alcaline phosphatase; 4 had jaundice.
Percutaneous cholangiography showed a stenosis ofthe anastomosis
in all patients.
24 patients underwent percutaneous bilioplasty with one year follow
up. In patient the treatment was not feasible because of complete
obstruction ofthe anastomosis, in patient we observed a re-stenosis
after year. Complications occurred in 4 patients (2 major and 2
minor): successfully treated with interventional procedure.
CONCLUSION: Clinical follow up of patients undergoing bilio-
digestive anastomosis is mandatory, because of the high risk of
stenosis. We think that percutaneous bilioplasty is the treatment of
choice because is able to obtain the same results of the surgical
procedure with inferior complication rate.
ANALYSIS OF OPERATIVE AND IMMEDIATE POSTOPERATIVE
COMPLICATIONS DURING LAPAROSCOPIC CHOLECYSTE-
CTOMY IN A SERIES OF 402 CASES.
K. Daskalakis, B. Christidis, E. Anagnostou, G. Diamantopoulos.
3rd Surgical Department, "EVANGELISMOS" Hospital, Athens,
Hellas.
The aim of this study is to analyse the intraoperative and
immediate postoperative complications of laparoscopic
cholecystectomy and their management.
In the 3rd Surgical Department of "EVANGELISMOS" Hospital,
409 patients were programmatised for laparoscopic
cholecystectomy. Finally, 502 laparoscopic cholecystectomies
were performed (98.3%) and 7 open cholecystectomies (1.7%) for
different reasons. There were 37 intraoperative complications in
34 patients (8.3%). The management of intraoperative
complications was: conservative in 7 patients, laparoscopical in
23 and by open operation in 4 patients. In these 4 patients
(0.9%), laparoscopic cholecystectomy was converted in open
cholecystectomy because of haemorrhage in 3 patients and
common bile duct injury in patient.
Immediate postoperative complications were presented in 20.
patients (4.8%). Two of them were managed by open operations
(1 haemorrhage, bile leakage) and by laparoscopic operation
(extraction of drainage tube from peritoneal cavity). The other
complications were managed conservatively. There was one
postoperative death in this series (0.25%) because of acute
cardiopulmonary insufficiency.
In conclusion, the small number, of intraoperative and
postoperative complications, suggest that laparoscopic
cholecystectomy should replace the oper cholecystectomy in the
majority of cases.
P344 P345
INFLUENCE OF PNEUMOPERITONEUM PRESSURE ON LUNG
COMPLIANCE AND RESISTANCE DURING LAPARASCOPIC
CHOLECYSTECTOMY
V. Dinevska, T. Boskovskl, D. Jovanovsld, S. Janevsid
Department of Anesthesiology and Intensive Care, Mtary Hospital, SkopJe,
Republic of Macedonia
The aim of this study is numeric and visual presentation of the positive
pressure ventilation and lung level changes during pneumoperltoneum (positive
abdominal pressure).
I00 patients in general anesthesia were evaluated, with ASA
classification female, and the average age was 40. Compliance, resistance, peak
peep, plato, tidal volume, minute volume, explratory CO2, 0'2 saturation with
pulse oxymetrin during surgery, metabolic and gas status before and at the end
of pueumopeHtoneum (P.P.)
We .used Drager apparatus, Spirolog2, PM$050 monitor Drager
overbuilt on Drager anesthesia apparatus.
The results are presented graphicaliy and in table showing m|ddle
values in percentages. The value in horizontal position is the initial and control
value. In Trendelenburg position with / 40 the compliance decreased from
100% (Initial value) to 74,3%. The effect of compression from the abdominal
organs on the lungs was 26%. With Increasing abdominal pressure, compliance
decreased on 46,5%. The effect of the compression was 54%. Anti-
Trendelenburg position with Z 40,compliance increased on 54%. The effect of
compression decreased on 35%.
In P.P. with 5 mm Hg pressure, the compliance was 64% and the
effect of compression was 36%. In P.P. with 10 mm Hg pressure, the
compliance was 47%. At 15 mm HE, the compliance was 44% and the effect of
compression was 56%. At 20 mm Hg the compliance was 35% and the effeoCt of
compression was 62%. At 25 mm Hg, the compliance was 36% and the effect
of compression was 64%. The largest effect of compression was on 15 mm Hg.
From 15 to 25 mm Hg the effect was Iflgher for only 8%. In obesity patients the
largest effect was on 10 mm Hg and between 15 and 25 mm Hg the effect was
higher for only 3%. Higher pressure than this value cannot make inter-.
abdonflnal vlsuality better.
During this surgery the patient must be continuously monitored. The
problem with adequate ventilation exists in patients with chronic obstructive
lung disease and significant compliance reduction before surgery, high value o!
blood CO2 or bad 02 saturation due to any reason In the respiratory
physiological circle.
LUNG COMPLIANCE .ND RESIST.JICE DURING I,APARATOMY
AND LAPARASCOPIC CHOLECISTECTOMY
V. Dlnevsk_a, D. Jovanovsld, S. Janevskl, T. Boskovskl
Department of Anesthesiology and Intensive Care. Military Hospital, SkopJe,
Republic of Macedonia
The most obvious difference between Iradlttonal surgery and
laparascoplc surgery is the need to establish pneumoperitoneum.
In this stud.." we present the results of compliance and resistance
during two different surgical melhods, In general NLA anesthesia.
Ventilation was with constant tidal volume and frequency.
In this study we used Drager apparatus, Spirolog 2 and M.P.8050
monitor Draget, vtlch were overbuild on Drager anesthesia apparatus.
Compliance, resistance, explrato "O2, peak, plato, peep ol
airway gas, 02 saturation with pulsoxhnetry were continuously monitored.
Metabolic mtd gas status were measured at the beginning and
after pneumoperltoneum (P.P.). The Initial value of compliance and
resistance, in horizontal position represented a control value for the other
positions in the measurement.
In Trendelenburg position, compliance, vts 73% and resistance
was 24% higher. In Trendelenburg
"40 wilh P.P., compliance decreases
on 46% and resistance was 65% higher than at the beginning. The
compliance value In the Trendelenburg position s 53% and resistance
34%. At the end position (horizontal) compliance ws 89% from the initial
value. The compliance value In lraditlonal (laparatomy) choleclstectomy
represented a control value for the other positions. In cholecistectomy
position compliance was higher for 10,5% and resistance was 11%. During
surgery compliance decreased for 24% and resistance decreased for 8%.
At the end position (horizontal) compliance and resistance are nearly the
Initial value. These changes can easily be established in lung
uncompromlsed patients. The problem vdth adequate ventilation exists in
chronic obstructive lung diseases with significant compliance reduction
before surgery, high value of CO2 or 02 saturation of .any reason In
respiratory Physiological circle.
In laparascopic surgery conant monitoring is necessary.
Increased Intraabdomlnai pressure after insufllating may cause
respiratory problems, especially in patients with diminished pulmonary
reserves.
158
P346 P347
MANAGEMENT AND OUTCOME OF LAPAROSCOPIC BILE DUCT
INJURIES
N Doctor, Dooley, R Dick, K Rolles, B Davidson.
University Dept. Of Surgery, Royal Free Hospital School of Medicine.
The incidence of bile duct injuries is reported to have increased following the
introduction of laparoscopic cholecystectomy(LC). We report the results of
management of 15 patients referred to the Hepatobiliary Unit following bile
duct injuries sustained at LC over the last years. 15 were female and was
male. The age ranged from 22 to 74 years.
Four patients had conversion and repair following recognition of injury at the
time of LC. They presented 4-8 months later (mean 6.5 months) with
cholangitis. In the other 11, injury was suspected in the postoperative period (2-
42 days, mean 11 days), because of abdominal pain, fever, and ileus. Diagnosis
was by ERCP and when necessary by percutaneous transhepatic
cholangiography. Angiography was performed when major injurywas diagnosed.
US and CT were performed for the diagnosis and drainage of intra-abdominal
collections.
Two patients had a cystic duct stump leak, a gallbladder fossa leak,
common bile duct (CBD) injury, 9 a common hepatic duct (CHD) injury, and
an associated vascular injury.
The 5 patients with cystic duct or fossa leak had successful nasobiliary and
percutaneous drainage of bile with no sequelae at a median of 18 months.The
patient with CBD injury was successfully managed by primary repair. Three of
four CHD injuries repaired at the time of LC needed revision and responded
to percutaneous dilatation. revision reconstructions were successful (mean 11.2
months). Five Roux loop reconstructions for injuries diagnosed later were
successful (20 months mean follow up).
Patients without main duct damage were successfully treated with percutaneous
drainage and stents. Roux loop reconstruction was successful both as a primary
procedure and for failed repairs done elsewhere. Patients with major duct
injuries should have angiography.
COMPLICATIONS OF PERCUTANEOUS STENTING FOR BILIARY
OBSTRUCTION:A FIVE YEARREVIEW
Doctor N Millson C, Dafnios N, Salamat A, Whiteway H, Dooley J, Dick R,
B R Davidson
Hepatobiliary Unit, Royal Free Hospital Medical School, London NW3
Introduction: Percutaneous placement of biliary stents is usually performed
when the endoscopic method has failed or the ampulla is inaccessible due to
previous surgery. The complications of the percutaneous route have not been
properly evaluated.
.Method: Using the Radiology department computers all patients who had
undergone percutaneous stent placement (PSP) over a five year period, were
identified. The casenotes were analysed for patient characteristics, indication for
t.he procedure, diagnostic criteria, complications and outcome.
Results: 94 patients (50 men and 44 women) underwent PSP over a five year'
period. Mean age 68.5years (range 37 to 90 years). The main indications were
carcinoma of the pancreas or liver/hilar metastasis (59%). Prior to the
Procedure, all patients had biliary obstruction, associated with cholangitis in 10
(11%), or impaired renal function in 9 (10%) sixty-five(69%) had previous
unsuccessful ERCP, whilst 23 (24%)had previous gastric surgery.
Eighty-five patients had successful PSP. Early complications were seen in 23
(27%) patients: acute cholangitis 10 (11%), haemobilia 4 (5%), renal
impairment 4 (5%), bile leak (3%), perforation 4 (5%), pleural effusion
(4%). There were eleven (13%) in hospital deaths. 60 (64%) patients were
discharged home, 10 (11%) transferred back to their referring hospitals whilst
patients required surgery or radiotherapy. The late complications were as
follows: Stent blockage in 10 (12%), cholangitis 13 (15%), stent migration 5
(6%) and a broken stent in (1%). 71 (84%) of the 85 undergoing PSP had
their symptoms relieved. Of the 9 patients who failed stenting, there were 5
deaths, three required palliative surgery and one was discharged for palliative
care.
Conclusions: Percutaneous stent placement is an effective method ofpalliating
obstructive jaundice, but should be limited to those patients who have failed an
endoscopic approach.
P348 P349
SENS1TWITY AND SPECIFICITY OF CHOLECYSTOKININ
CHOLESCINTIGRAPHY.
G.W. Dyke, A. Wycherley*, L. Kow, R.T.A. Padbury, T.G.
Wilson and J. Toouli
Departments of Surgery and Radiology*, Flinders Medical
Centre, Adelaide, Australia.
The aim of this study was to evaluate the sensitivity and
specificity of cholecystokinin cholescintigraphy by reviewing
our use of this test over the last ten years. A total of 185
patients underwent cholecystokinin cholescintigraphy for
acalculous biliary symptoms between 1985 and 1994.
Outcomes were available for 148 (80%) of these patients. Forty
six patients had an abnormal gallbladder ejection fraction
(GBEF < 40%) of whom 34 underwent cholecystectomy.
Symptoms relief was obtained in 28 patients with a median
follow up of 6.5 years and over 80% had a histologically
abnormal gallbladder removed. Of the 139 patients with a
normal GBEF, 32 were lost to follow up, 17 had an alternative
diagnosis made, 23 lost their symptoms with no diagnosis
being made and 30 have persistent symptoms. Thirty seven
patients with a normal GBEF have undergone cholecystectomy
for ongoing symptoms. The range of GBEF in this sub-group
was from 43% to 99% with a median GBEF of 66%. Symptom
relief was obtained in 30 of these patients (81%) and
histologically abnormal gallbladders were removed in 34 cases.
These results are no different to the outcomes in patients with
abnormal GBEFs. This study confirms that cholecystokinin
cholescintigraphy will predict patients who will benefit from
cholecystectomy with a sensitivity of over 80%. The specificity
of the test is 45% at best and may be less than 30%. This
suggests that cholecystokinin cholescintigraphy requires
further evaluation before it is used as a routine test.
159
COMPLICATIONS DURING LAPAROSCOPIC CHOLECYSTECTOMY
..Endzinas, A. Bogueviius
1st Surgical Clinic of Kaunas Medical Academy, Lithuania.
Preoperative, intraoperative and postoperative data were documented systematic-
ally and prospectively in 593 patients, who underwent laparoscopic cholecystec-
tomy (LC) from January 1993 to November 1995. Results: In 18 cases initially ap-
plied LC was converted to open procedures (3%) because of unclear anatomy (5),
technical difficulties (8)or when intraoperative complications were diagnosed
(5 cases). Intraoperative complications occurred in 13 cases. We had 4 serious bile
duct injuries (0.67%). Two of them were unrecognized at the primary interven-
tion, other two were immediately detected and conversion to open procedure was
done. In one case of acute cholecystitis common bile duct was transected because
of its misidentification as cystic duct, in another case it was laceratecL Suture of
common bile duct with its drainage were performed in both cases followed by
good results 6 month later. In one case injury of ductus hepaticus communis oc.
cured, but it was misdiagnosed as bile leakage after operation. The patient was
reoperated month later because of persistent bile fistula. The site of injury was
not found at laparotomy and just drainage of infrahepatic space and port of liver
was made. Two months later stricture of bile duct formed and relaparotomy was
done. Signs of mechanical jaundice disappeared after hepaticojejunostomy was
pe.rformed. Another patient readmitted to the clinic 10 days a,qer LC because of
peritonitis. Thermal necrosis of ductus hepaticus communis was found on laparo-
tomy. Primary suture of bile duct with its drainage with Kehr's tube led to stric-
ture forming. Four moths later hepaticojejunostomy was performed with good re-
suits. Two diaphragm injuries without opening of the thoracic cavity occurred.
Five patients had significant intraoperative bleeding: from cystic artery (2) or
liver bed (3). In three of them conversions to laparotomy were necessary, In one
case bleeding from liver bed was solved by applying, the drainage tube placed in
infrahepatic space. One fatal outcome occurred because of unrecognized electro-
thermal perforation of small bowel and diffuse peritonitis in 78 years old patient.
We suspected that insulation of instrument was damaged and perforation of bo-
wel occurred as a result of direct contact of metal instrument port and bowel
wall. More than 50% ofall complications occurred when the surgeon was perfor-
ming his first 50 operations, or when he started with acute cases. All of our sur-
geons were mastered themselves. Conclusion: Good learning programs with
obligatory hands-on training should be introduced for the beginners; experienced
surgeons should accompany the less experienced ones until the threshold num-
ber of interventions is performed.
P350 P351
BILE DUCTSTRICTURE AFTER BLUNTTRAUMA
T. En0i, D. Hayashi, K. Okamura, T. Takahashi, H. Shinagawa, S.
Noshima, N. Morita, K. Esato
First Department ofSurgery, Yamaguchi Univ., Ube, Japan
Bile duct injury caused by blunt abdominal trauma is not common,
probably because bile duct is protected by surrounding tissues,
including the liver, the pancreas, the duodenum and the vertebral
column. Two cases of obstructive jaundice after blunt abdominal
trauma in vehicle accident have been treated in our institute. In this
study these two cases are to be reviewed extensively. Case 1: Forty-
two-year-old female was admitted to the nearer hospital immediately
after vehicle accident because of severe abdominal pain, which
spontaneously reduced by the next morning. Eighteen days later after
the accident, apparent jaundice and liver dysfunction were pointed.
out. She was referred to our institute for further examination and
treatment. Ultrasonography (US) and endoscopic retrograde
pancreato-cholangiography ERCP revealed dilatation of biliary
tract and circular stenosis of mid-portion of extrahepatic bile duct, at
which no malignant cells were detected through the cholangioscopic
biopsy. Although jaundice successfully disappeared by biliary
drainage, stenosis of the biliary tract persisted. Eight weeks after the
initial trauma, she underwent choledochojejunostomy. Surgical
specimen showed fibrosis of the common bile duct without any
evidences of malignancy. Case 2: Forty-year-old male was admitted
due to further examination for jaundice 2 weeks after the vehicle
accident. Naso-biliary tube was placed in the proximal portion above
the stenotic lesion of lower bile duct following ERCP. The biopsy
finding through the cholangioscope showed no malignancy. Stenotic
lesion of lower bile duct had no tendency for improvement despite 6
weeks' biliary drainage. Biliary reconstruction was performed and his
postoperative course was uneventful.
Hepatobiliary Cystadenoma A Case
Report and Literature Review
Huo Feng, Luo Mingyi, Ma Oi, Zhu Yong.
Dept.of Hepatobiliary Surgery Naval General Hospital.
Beijing, China 100037
Hepatobiliary Cystadenoma (HBC) was very arely
diagnosed in the past.But it has been reported more
frequently in China in last two decades. After treating a
case of HBC recently in our hospital, a review of literatures
on this subject was done, and 9 cases of ttBCfromdomestic
documents were analysed together with our case.
The occurenco of ttBC may be related with the
abnormal dovelopment of the biliary duet epithelium during
embryogenesis. Owing to high recurrence rate after resection,
it is now regarded as kind of premallgnant tumors. The main
clinical symptoms include abdominal pain. tumor mass.
jaundice, and gastrointestinal complains.. Be well aware of the
disease and with advanced image diagnostic techniques, it is
possible to establish an accurate diagnosis before operation.
As for it's treatment, radical hepatolobectomy is
advocated in order to prevent recurrence following surgery.
Pathological confirmation by frozen section and
choledochography should be instituted during operation.
KEY WORDS hepatobiliary Cystadenoma,
diagnosis, operation
P352 P353
Mini-incision, Primary Chloleystorrhaphy
in the Management of Cholecystolithlasls
and Cholecystopolyps
Huo Feng, Luo Mingyi, Ma Yusngui
The Naval Genera/Hospital of PLA, 100037
Wang Qi, The 302 Army Hospital of PLA, 100039. Beijing, PRC
From November 1990 to November 1994, Olympus A5207
laparoscope was used to remove gall stones and
polypoid lesions, through mini-incision of the abdominal
wall and fundus of the gallbladder. The POIyI were sent for
frozn section immediately and intraoperative eholanglography
was performed. After complete hemostasis, the mucous
membrane and serous coat were separately closed by continuous
suture using 4/0 nontramatic suture needls. A totle of 244
cases were treated with this technique. Among them, 132 had
cholecystolithiasis, 112 had polyps. One case developed bile
leakage which was cured by catheter drainage. One
hundred and three patients with gall stones and 88 with polys
four cases of gall stons (3. 88%) and five polys (5. 68%)
respectively. The techniqune of mini-incislon and primary
cholecystorrhaphy is less tramatic and capable of preseving
the function of gallbladder. Generally the patients could get
up and take liquid diet in the evening after operation. The
hosptal stay is shorter than those undergoing cholecystectomy.
Key words cholecystolithiasis, cholecystopolyp,
laparoscope primary suture
FREQUENCY AND CHARACTERISTICS OF ABDOMINAL SYMPTOMS IN GALLSTONE
DISEASE: RESULTS OF AN EPIDEMILOGICAL STUDY.
D. Festi. A. Sangermano*, S. Sottili*, A. Colecchla*, M. Orsini*, E. Roda and the M.I.CCL Group.
Physiopathoiogy Department, University G. D'Annunzio, Chleti and *Gastroenterqlogy
Department, Universita' di Bologna, Italy.
Different surgical and non surgical therapeutic options are presently available for the
management of gallstone patients, One of the key factors in clinical deci.ion making is the
recognition of symptoms caused by the gallstones; however, there is still no definitive agreement
regarding the abdominal symptoms specifically attributable to this condition. The aim of the
present study was to evaluate the frequency and characteristics of abdominal symptoms in
gallstone disease (GD) patients, during an epidemiclogical study, Multicentrica Itallana Colelitiasi
(M.I.COL), a community-based Investigation of GD prevalence in Italy, involving 18 cohorts in
10 Italian regions. Components of the study protocol pertinent to this work include
ultrasonographic (US) examination of the upper abdomen and direct interview of subjects using
precoded questionnaire regarding medical history and abdominal symptoms during the years
prior to the Interview in gallstone-free (GF) and in gallstone patients (GS), and in the yrs prior
to surgery in cholecystectomized patients (Q. The pepulation studied comprised 29,584 subjects
15,910 males and 13,674 females; aged 30-69 yrs). On the basis of the clinical and US results,
this population was divided into the following groups: GF= 25,479, GS=2,480 and C=1,634. The
frequency of abdominal symptoms (belching, heartburn, nausea, vomiting, bloated feeling alter
meals, intolerance to fatty or fried foods, heavy feeling the right side epigastrlum, bitter
taste in the morning and abdominal pain) and the characteristics of pain (localization, radiation,
intensity, frequency, relation to meal, rate of complications) when present, were evaluated on the
basis of the questionnaire. Multivariate analysis was performed with the multiple logistic
regression model to estimate the Odds Ratio and the 95% C.L. adjusted for the other variables in
the model. Multivariate analysis demonstrated that, among the evaluated symptoms, three
closely linked to GD abdominal pain (OR 2.2 GS va GF; OR 4.4 C vs GF), heavy feeling on the
right side (OR 3 GS GF: OR 1.7 C vs C al jntcE,mlc.e to flty fri ftd.o=/,t'::t GI
vs GF; OR 1.3 C vs GF). Pain was present in 17.9% of GS, 72;5% of C prior to surgery and
8.3% of GF. With respect to GF, the pain in GS tends to radiate (OR 1.3), is variable in duration,
forces the patient to lie down (OR 1.3), is not relieved by bowel movements (OR 1.2), is meal-
related and triggered by abundant meals (OR 1.2), and can be associated with complications
(OR 1.4). In C before surgery, with respect to GF, the pain forces the patient to lie down (OR
2.2), needs drugs to be relieved (OR 1.3), has a longer duration (OR 1.3), occurs more
frequently (OR 1.5), is meal-related (OR 1.2), is not relieved by bowel movements (OR 1.6), and
is more frequently aasodated with complications (OR 2.1). This study confirms that abdominal
pain is closely associated with GD, and also shows that other abdominal symptoms are
significantly linked to this disorder. If confirmed by the longitudinal study, these findings could be
usefJI in the therapeutic management of gallstone patients.
160
P354 P355
LAPAROSCOPIC SPLENECTOMY FOR MASSIVE
SPLENOMEGALY
GA Fielding, IS Bailey, J Lumley, NA O'Rourke, LK
Nathanson.
Royal Brisbane and Wesley Hospitals, Brisbane,
Australia.
Laparoscopic splenectomy for haematological disorders
and staging of Iymphoma has been widely reported.
However little experience of laparoscopic splenectomy
for massive splenomegaly has been reported. We
report 16 (12M, 4F) laparoscopic splenectomies for
massive splenomegaly (> 1300g). Median splenic
weight 1800g (range 1300g 3500g).
Pneumoperitoneum was by open cannulation, vascular
control was achieved with Endo GIA. No pre-operative
embolisation was utilised. Operation was completed
laparoscopically in 13/16 cases. Three patients were
converted due to haemorrhage. Seven of 16 required
pre-operative blood transfusion. Operative duration
was 150 minutes (105 -270) in patients completed
laparoscopically. Post-operative stay was 4 days (2
13). There were no deaths. Three patients had
significant post-operative morbidity (MI, post-operative
bleed, left pleural effusion). Post discharge recovery
was rapid and comparable to laparoscopic splenectomy
for smaller spleen. Laparoscopic splenectomy for
massive splenomegaly is achievable and desirable.
Operative bleeding is a significant problem and
experience is required to complete the procedure.
BILIARY CYSTADENOCARCINOMA RESECqD BY
SEGMENT 3 AND 4 HEPATECTOMY
M. Frameglia, Y. Nimura, N. Hayakawa, J. Kamiya, S. Kondo, M.
Nagino, M. Kanai, M. Miyachi
The First Department of Surgery, Nagoya University School of
Medicine, 65 Tsurumaicho Showaku Nagoya, 466, Japan
We report a ease of biliary cystadenocarcinoma of the liver in a 72-
year-old woman presented to our hospital with abdominal fullness.
Laboratory data showed an elevation of alkaline phosphatase and a
decreased excretion of Indocyanine green (ICG). CT revealed a
cystic tumor with papillary projections ,measuring 13-15 cm, in the
left medial segment of the liver ($4). Percutaneous transhepatic
cholangioscophy (PTCS) disclosed the tumor in the dorsal
subsegmental duct of $4 and the cholangioscopic biopsy from the
tumor revealed papillary adenocarcinoma. PTCS showed the left
lateral posterior segmental bile duct (B2) joined the common tract of
the left medial (B4) and left lateral anterior (B3) segmental bile duct,
and the tumor involved B4 and B3 but not the common tract of B4
and B3. A radical surgery, which included segment 4 and 3
resection with preservation of the left hepatic duct and the segment 2
was performed. The histopathological examination revealed that the
tumor did not involve the liver parenchyma and had no lymph node
metastasis. Postoperative course was unremarkable and the patient
at present time, 4 years and 6 months after the operation, is doing
well without recurrence of the tumor.
We present an unusual surgical procedure of segment 4 and 3
resection with preservation of the left hepatic duct and segment 2 in a
patient with poor liver function and emphasize the diagnostic role of
percutaneous transhepatic cholangioscophy (PTCS) to make a
precise information ofthe segmental anatomy of the intrahepatic bile
ducts and cancer extension.
P356 P357
METHYLPREDNISOLONE: IS THERE A ROLE FOR PBC
TREATMENT?
P. Fusaroli, A. Pezzoli, F. Azzaroli, C. Mazzeo, A. M. Gioacchini, G.
Mazzella C. Cerr, E. Roda. Dept. of Internal Medicine and
Gastroenterology; Dept. of Pharmacological Science, University of Bologna,
Bologna, Italy.
Methylprednisolone (MP) treatment has been considered to be an effective
treatment for primary biliary cirrhosis (PBC). Since bone loss is the major
side effect, extensive cliniial trials have been limited.
Up to now, biliary lipid metabolism in PBC has not been investigated during
MP therapy. We studied six patients with histologically confirmed PBC
(stages I-III) administering 24 mg ofmethylprednisolone per day for a month.
All patients underwent a 30-day washout period of all previous treatments
before entering the trial. The following parameters were tested before and
aider the treatment period: serum biochemistry, serum bile acids,
immunoglobulins and autoantibodies; cholic acid pool size, kinetics and
synthesis; biliary lipid secretion; biliary bile acid molar percentage;
cholesterol saturation index (Carey).
MP induced a statistically significant (p<0.03; Wilcoxon rank test and
Bonferroni correction) increase in total and HDL-cholesterol serum levels
(from 228+22 mg/dl to 248+/-23 and from 52+/-4 to 67+/-2, respectively;
mean+/-SE) and a decrease in ALP (p<0.03) and 3,-GT (p=ns) levels (from
546+/-176 U/L to 283+/-92 and from 220+/-95 to 125+/-146 respectively;
median+/-SE).
We didn't find any statistically significant variation in the following
parameters: biliary lipid molar percentage, biliary lipid output, cholesterol
saturation index, cholic acid pool size, kinetics and synthesis. On the contrary,
we observed a significant increase (p<0.03) ".m bile deoxycholic acid (DCA)
molar percentage (from 17.7+/-1.5 % to 39.1+/-2.5; mean+/-SE).
Summarizing our findings, we can conclude that a short term administration
of MP has no effect on biliary lipid secretion, cholic acid pool size and
kinetics; on the other hand, it modifies endogenous bile acid pool size by
increasing deoxycholic acid levels.
Before corticosteroids can be considered for the reatment of PBC, further
studies are needed to evaluate whether the DCA accumulation is long-lasting
thereby affecting the beneficial immunosuppressive effects.
161
ERCP ASSOCIATED WITH LAPAROSCOPIC CHOLECISTECTOMY IN
THE TREATMENT OF CHOLELITHIASIS
P. Giamundo, M. Valente, L. Esercizio, G. Ansaldi, L. Tibaldi, L. Ghezzo*
Department General Surgery -Osp "S. Spirito" Bra (CN) Italy
*Department Gastroenterology- Osp. "S. Croce" Cuneo (CN) Italy
Laparoscopic cholecistectomy (LP) has been almost universally accepted
as the treatment of choice in the management of gallstones However,
gallstones are associated with choledocolythiasis in 5-15% of cases. The
management ofpatients with suspectect common bile duct stones remains still
controversial. It remains technically difficult to remove bile duct calculi
laparoscopically and feww centres currently have the expertise or equipement
to do this. Possible options are: 1) Endoscopic cholangiography and stone
removal followed by laparoscopic .cholecistectomy or 2) cholecistectomy,
intraoperative cholangiography and common duct exploration. Fromjanuary
'94 to july '95 142 cholecistectomy have been performed in our Department:
76 laparoscopic, 66 laparotomic. In 42 (30%) patients bile duct calculi were
suspected on the presence of one or more of the following, criteria: at least a
two-fold increase in total serum bilirubin, serum alkaline phosphatase, serum
amylase; an ultrasound common bile duct greater than 8 mm or showing a
possible stone in duct; a recent history of pancreatitis and jaundice. A
preoperative ERCP was performed in all ofthese cases. Bile duct calculi were
demonstrated in 26 cases (62%) and a successfull endoscopic removal of
calculi was achieved in 23 cases. In patients calculi could not be cleared
from the bile duct endoscopically in these cases an open cholecistectomy was
performed. In the remaining 39 patients, a LC was successfully performed in
38 cases (1 failing having refused the operation). The overall mortality rate
was 0. The morbidity of preoperative ERCP was 19 % due to bleeding (3
patients, not requiring blood transfusion), cholangitis (2 patients) and
pancreatitis (3 patients). These complications caused an overlenght in the
hospitalization but in no cases were determinant in the choice of surgical
treatment. In conclusion, altought the choice of treahnent in patients with
suspected common bile duct stone is still .controversial, we believe that a
precholecistectomy ERCP with stone removal should be performed in
selectioned cases according to low mortality and morbidity rates.
P358 P359
THE LAPAROSCOPIC TREATMENT OF COMMON BILE
DUCT STONES (CBDS) A PLEA FOR A PRECISE
SURGICAL STRATEGY.
J.F. Gigot, B. Navez
Department of Digestive Surgery, Louvain Medical School
Ninety-two patients with CBDS have been prospectively
treated by laparoscopic common bile duct exploration
(CBDE). Age over 75 years-old and high risk patients
were found in 17 and 16 % respectively. Seventy-six
patients underwent initial transcystic approach while 30
patients finally underwent choledochotomy. Mortality was
0 versus 13 % according to age > 75 years and high risk
patient. Transcystic approach carried out the lower stone
clearance rate (63 %), the higher biliary complications, the
shorter operative time and postoperative hospital stay
(POS). Choledochotomy carried out the greater stone
clearance rate (93 %), the lower complications rate and
the longer operative time and POS.
In conclusion, laparoscopic CBDE required a precise
peroperative strategy during laparoscopy and should be
avoided in old and high risk patients.
LAPAROSCOPIC CHOLECYSTECTOMY IN ACUTE
CHOLECYSTITIS
A Gradauskas, A. Eidikis
Vilnius City's University Hospital, Vilnius, Lithuania
Few years ago acute cholecystitis was considered to be a
contraindication to laparoscopic cholecystectomy (LC), but
now attempts are made to treat it with this procedure.
Among our 468 patients to whom LC was performed.- 87
(18.6%) were operated on because of acute cholecystitis. To
all of them'diagnosis was confirmed after
pathologoanatomical examination of the specimen. 76
(87.4%) patients were ill with acute phlegmonous, 11 (12.6%)
with acute gangrenous cholecystitis. 72 (82.8%) were
women, 15 (17.2%) were men. The patients ages ranged.
between 23 and 82 years (mean range 53.3 years). The
operating time ranged from 62 to 143 minutes (average- 82
min.). The average duration of postoperative hospitalization
was 7.3 days (from 3 to 17 days). There was no mortality and
few complications: wound infection in four patients,
postoperative pneumonia in one, and one patient turned
yellow 2 days after LC (choledocholithiasis was successfully
treated with EPST). In 12 (13.6%) cases converting to an
"open" operation was necessary because of: dense adhaesions
and subhepatic infiltrate 9 cases, adhaesions in abdominal
cavity after previous operations 2 cases, technical disorders
of equipment 1 case. In our experience LC in acute
cholecystitis is possible to perform in most cases. It benefits
the patient because of more rapid recovery and less pain
compared to open operation. To our .opinion the significant
skill and experience in performing LC is required before
attempting to treat acute cholecystitis laparoscopically.
P360 P361
SAPHENOUS VEIN GRAFT FOR IATROGENIC LESION OF THE
COMMON BILE DUCT. REPORT OF A CASE.
B. Griffa, V. Basilico, D. Clerici, P. Ceriani, G. Capriata
Department of Surgery I, Valduce Hospital, Como, Italy
Iatrogenic injury of the common bile duct occurring during elective
cholecystectomy is a rare but very serious complication for the patient and
a real problem for the surgeon. If it's immediately recognized and repaired
at the operation, the lesion has a better prognosis than if it's treated later
on. In case of tissue loss the customary treatment has been a Roux-en-Y
hepatico-jejunostomy. The Authors describe a case of 2 cm. long lesion,
localized at the distal common hepatic duct, caused by a too large T-tube
inserted forcefully into the biliary tract after a successful cholecystectomy
and choledocholithototmy. To repair the tissue loss, a graft of saphenous
vein was emploied, with a smaller T-tube inserted through the
anastomosis. The patient had an uneventful post-operative course. The
follow up for six years has been regular and no late complications have
occurred. The Authors emphasize the possibility to use more. liberally the
saphenous vein graft to treat iatrogenic injuries of the common bile duct
with tissue loss.
REFERENCES
1) Bismuth H., Lazorthes E. Les traumatismes operatoires de la voie
biliaire principale. J. CHIR., 118 10 601-09,1981.
2 Salim A.S. Choledochoplasty by vein grafts in iatrogenic bile duet
injuries. HPB. SURG., 5 (3) 195-201, Apr. '92.
3 Ristic D., Brzakovic I., Marusic G. Substitution of the ureter with a
segment of the major saphenous vein in dogs. ACTA CHIR. JUGOSL. ,-37
SUPPL. 95-7,1990.
4 Wittrin G., Clemens M., Arndt M., Ruhland D. Replacement of the
common bile duct by an autologous vein Get ). RESEARCH IN
EXPERIMENTAL MEDICINE, 173 95-103,1978.
LIVER RFECTION AND INTRAOPERATIVE CHOLANGIOSCOPIC
EHL AS A TREATMENT MODALITY FOR IHD STONE
SH. Hart, MS. Lee, HJ Kim, YD Kim, HY Kim
Devartment of Surgery, lnje University, Seoul, Korea
Hevatolithiasis is vrevalent disease in east Asian countries. However,
there are still controversies about which one is the appropriate
treatment modality. Retrospective study was carried out on 66
patients with intrahepatic duct(IHD) stone that were treated
surgically. Clinical profiles of patients were reviewed and modalities
of surgical treatment were compared in respect of outcomes and
complications. There were 40 females(60.6%) and 26 males(39.4A;).
The 5th and 6th decades exhibit peak incidence(54.5/6). Pain was the
most common symptom(66.7) and history of recurrent cholangitis
was confirmed in 38 patients(57.6A;). Stone location was mainly in
left Iobe(51.596), and bilobar distribution occuvied 27.396(18 cases).
Elevation of liver enzyme levels was observed in significant
proportion of the vatients; ALT in 39(59.1%), AST in 42(63.6),
alkaline phosphatase in 38(57.6%). Elevation of plasma -GT was the
most evident laboratory l'mding(78.8/6) while elevation of bilirubin
level(46.9) was not remalkable. Partial liver resection was done in
42 cases(63.6/6); left lateral segmentectomy in 30(71.4/6),
subsegmentectomy of right lobe in 8(19.1%), left Iobectomy in
3(7.1%), right Iobectomy in I(2.49). Hepaticojejunostomy with
subcutaneous jejunostomy was carried out on llcases(16.7%) which
had severe ductal stricture and dilated common bile duct(CBD).
Intraoperative cholangioscopic examination with electrohydraulic
lithotripsy(EHL) was carried out on 44 cases(66.7%). Mean operation
time was longer in the case with EHL(199.3 rain) than other
group(156.2 min). However, the incidence of postoperative residual
stone was decreased with intraoperative cholangioscopic EHL unless
increase of comilications. Recurrent cholangitis was absent in the
patients who received partial hepatectomy, and adequate biliary
drainage procedure. In conclusion, resection of cHseased part of liver
and adequate biliary drainage are the treatment of choice for IHD
stone disease. Intraoperative cholangioscopic examination with EHL
could reduce the incidence of residual stone and provide the precise
information about biliary trees such as combined malignancy or
ductal stricture.
162
P362 P363
HISTOLOGIC FACTORS OF PROGNOSTIC VALUE IN
GALLBLADDER CARCINOMA
LA Hidtlao, IM'Badia, $ Sufio-l, T Soler*, C Admella*, :I Feliu,
JM Gubern.
Department of Surgery and Department of Pathology*.
Consorei Sanitad de Matar6. Matar6, Barcelona. Spain
Gallbladder carcinoma (GC) is fifth in frequency in developed
countries. Its prognosis is usually bad, mostly due to the advanced
state of the disease at diagnosis.
Aims: To analyse the prognostic histologic factors according to the
TNM classification and the cell differentiation degree in our series
of GC.
Methods: Twenty-five GC were_ included in the series (4 men, 21
women, mean age 75+/-13 years). All patients had gallstones. Six
eases were preoperatively diagnosed of GC by US scan (24 %), in 3
cases the diagnosis was made by the surgeon at operation, and the
rest were unsuspected histologic diagnosis. Patients were divided into
.group A (complete resection, n=15) and group B (no resection or
neomplete resection, n=10).
Results: Only in one patient of gr.oup A the diagnosis was made
preoperatively. In this group, sx tumours were T1N0, well
differentiated in 5 eases. Four eases were; 3 of them NO (well
differentiated and N1 (undifferentiated). There were 5 T3
tumours, one of them NO (undifferentiated) and 4 N1 (2 well
differentiated). In group B there were 10 GC, 8 of them
preoperatively diagnosed by US. Seven tumours were T3 (2 well
diffcrent-;ated, 2 moderately differentiated, 3 undifferentiated).
Three tumours were T4 (all of them undifferentiated). All patients in
group A survived more than 2 years, except for one case T3N and
one T2N1, who succumbed due to local relapse. Patients in group B
did not survived more than 6 months.
Conclusions: 1) Preoperative diagnosis of GC is not frequent,
especially in non locally advanced tumours (TI T2). 2) The level
of infiltration in gallbladder wall (T) is related to node metastases. 3)
Well differentiated turnouts have a better prognosis,
A CLINICAL STUDY OF FREE RADICAL IN THE PATIENTS WITH
OBSTRUCTIVE JAUNDICE
A. Horiguchi. S. Miyakawa, K. Miura.
Dept. of Surgery, Fujita Health University,Toyoake Japan.
In some patients with severe obstructive jaundice OJ on whom
PTBD was performed, the bilirubin decreasing rate is poor. In
these patients, the tissue injury of the liver has become
irreversible due to OJ. Recently free-radical have attracted the
attention of researchers as the cause of the tissue injury.
Therefore the generation of free radicals, wa studied in
comparison with the bilirubin decreasing rate in patients with OJ.
(MATERIALS AND METHODS) Blood samples were collected 1, 3, 7,
14, and 21 days after PTBD from 40 patients with and from some
controls. In these samples, serum total bilirubin levels, serum
lipoperoxide (LPO) serum superoxide dismutase (SOD) activity
and luminol dependent chemiluminescence (LDCL) were measured.
(RESULTS) LPO LDCL and SOD activity levels of blood samples
collected the first day after PTBD in patients with OJ were
significantly higher than those of the controls. The patients were
divided into two groups; one group (A) had a good bilirubin
decreasing rate (b<-0.05), the other group (B) had a poor bilirubin
decreasing rate (b>-0.05]t In group B, LDCL and LPO levels were
significantly higher each day in comparison with those of group A.
SOD activity of group A was consistently higher than that of group
B. From results it can be concluded that 1) Free-radical are also
generated in the patients with OJ, 2) In patients with poor
bilirubin decreasing rate, the generation of free-radical is higher
but scavenger levels are lower, therefore tissue injury ensues
more seriously.
P364 P365
FUNCTION CAIGSS CF LIVER CELL IiEiBRANE FOR
BILIBUBIN EJ4CRETION IN OBSTRUCTION JAUNDICE
YT Huang, WH Wu
Department of Surgery, The First Hospital,
Beijing Medical University, Beijing, China
The aim of this animal study was to investiga-
te dynamically the relationship between excre-
tory function o f bilirubin, and ultramicrostru-
cture of the liwer. The obstructive jaundice
(OJ) model was made in 322 Wistar rats with
ligation of common bile duct and the sham for
control. The saples of bile juice, peripheral
blood and liver tissue were collected on the I,
3, 5, 7,14,28 days postoperatively and the 1,7;
days after relief of obstruction. The results
showed that Na+K+ATPase existed on the sinu-
s0id, intercellular and bile canacular memb-
rane facies had irregular distribution. The
Na+K+ATPase activity was increased in the ear-
ly stage of 0J and decreased some extent later.
The changes became evident with CJ prolonged,
expecially on the facies of bile canalicular
membrane. The activity of sucinate dehydrogen-
ase(SDH) within liver cell decrease gradually
with duration of OJ prolonged. The activities
of Na+K+ATi:ase and SDH could recover followed
the duration prolonged after relief of 0J. it
also showed that ,erum ff-bilirubin maintained
higher level after relif of OJ. it. ight ex-
plain that clinical jaundice often disappeared
slowly because of delayed excretion of -ili-
rubin through the aidaey, despite the better
recovery of liver function.. Clinical observa-
tion revealed that the course of disappearen-
ce of OJ was hot regular after relif of obs-
truction and amelioration of liver function
was not compatible with. extent of improvement
of OJ.
163
THE PATHOLOGICAL INVESTIGATION OF GALLBLADDER
MUCOSA BY STEREOSCOPE
K. Inoue, Y. Koyanagi, T. Aoki, T. Ashizawa, A. Tsuchida, T.
Aoki,
T. Ozawa, O. Uda, T. Hashimoto, H. Saito,I.Sonoda,A.Masuhara
Dept. of Surgery, Tokyo Medical College, Tokyo,Japan.
Materials and Method;We evaluated 34 cases of gallbladder
which were resected from February 1995 to April 1995. we
observed the structure of mucosa and blood vessel wlich could be
seen through by stereoscope(10-60X). Then the specimens were
fixed for 24 hours by 10% buffer hormalin, they were stained by
Hematoxylin-Eosin and observed pathological findings in order to
be distinguished with the findings by stereoscope.Results;(1)The
normal mucosa of gallbladder showed regular and latticed pattern of
mucous membrane .(2)In the inflammatory mucosa the irregularity
of latticed pattern and the product of small blood vessel could be
seen .Their blood vessel ran along the peak oflattice.(3)In the
hyperplastic mucosa tall papillary mucous pattern could be observed
and the product of blood vessel was quite little.(4)The mucosa with
cholesterosis showed the irregularity of latticed pattern and the
settlement ofcholesterol in the peak of lattice.(5) In the cancer of
gallbladder the latticed pattern disappeared and large blood vessels
with encasement which ran disorderly could be
observed.Discussion;The purpose of this study is the trial of the
intraoperative observation by stereoscope inorder to improve the
diagnosis ofminute abnormality such as surp.erfiial type ofearly
gallbladder cancerbecause in our past experience 32.9% (25/76) of
resected gallbladder cancer were pathologically diagnosed as.
gallbladder cancer initially after surgery. As there were differences
ofthe mucous patternbetween normal; inflammatory mucosa and
malignant lesions in our series, it is suggested that the observation
by stereoscope is useful for the differential diagnosis.
P366 P367
TREATMENT OF PATIENTS WITH ADVANCED TUMORS OF THE BILE
DUCTS WITH CONTINUOUS INFUSION 5-FLUOROURACIL IN
CONJUNCTION WITH CALCIUM LEUCOVORIN,
MITOMYCIN-C AND DIPYRIDAMOLE:
A PHASE II TRIAL
William H. Isacoff, M.D., Mandy S. Greenberg, Ronald K. Tompkins, M.D.,
Howard A. Reber, M.D., Ronald W. Busuttil, M.D., David McFadden, M.D.,
Priya Jamidar, M.D., Olivia Taylor.
Department of Medicine, Department of Surgery, UCLA, California, USA.
From February 1988 until January 1995, we treated 31 patients with
advanced tumors of the extrahepafic bile ducts with continuous infusion 5-
Fluorouracil (5FU) (200mg/m2/day), Calcium Leucovofin (LV) (30mg/m2/IV)
q week, Mitomycin-C (Mito C) (10 mg/m2) IV (not to exceed 15 mg total per
dose) q six weeks and Dipyridamole (D) (75 rag) orally q.i.d. There were 11
women and 20 men whose median age was 66 years (range 39 to 80 years).
Median Karnofsky performance was 80% (range 60% to 100%).. There were 20
patients with Stage II or III disease and 11 patients with Stage IV. ,Thirty
patients were evaluable for response with 31 patients evaluable for toxicity and
survival. There were 3 complete responses (10%) and 10 partial responses
(33%), for an overall response rate of 43 %. The median survival for patients
with locally advanced disease (Stages II and III) was 11 months (range 4 to 29
months) and 12 months (range 6 to 89+ months) for patients wide metastatic
disease (Stage IV). The dose limiting toxicity was stomatifis observed'in 17
patients (55%) requiring a reducfiori in the 5FU dose. No treatment related
fatalities resulted from therapy.
CONCLUSION: This regimen is safe and well tolerated. It is active therapy
for patients with advanced bile duct cancer. The survival benefit observed from
this four drug program in patients with both locally advanced and metastatic
disease is superior to that which is seen with standard 5FU based therapy or
radiation therapy.
INTERNAL VS EXTERNAL BILIARY DRAINAGE FOR
LONG-TERM OBSTRUCTIVE JAUNDICE: IN REFERENCE
TO THE HEPATIC TISSUE BLOOD FLOW AND THE
HEPATIC ENERGY METABOLISM IN OBSTRUCTIVE
JAUNDICED RATS.
,T. Takada, H. Yasuda, T. Uchida, N: Toyota
1st Department ofSurgery, Teikyo University, Tokyo, Japan
[Object When biliary decompression was applied in early
stages of obstructive jaundice, hepatic energy metabolism
shortly improved. However, in long-term obstructive jaundice,
hepatic energy metabolism was very late to improved after
biliary drainage. We evaluated the effects of biliary drainage
on hepatic energy nietabolism between internal and external
drainage for long-term obstructive jaundiced rats. [Method]
Long-term (4 weeks) obstructive jaundiced rats, Male Specific
pathogen-free Sprague-Dawley rats, received either one of these
two kinds of biliary decompression method; Group 1: internal
drainage by choledochoduodenal fistula, Group 2: external
drainage by Choledochobladderal fistula. Arterial ketone body
ratio (AKBR) was measured as an index of hepatic energy
metabolism. Hepatic tissue blood flow was determined by laser
Dopper velocimetry. [Result] Total bilirubin levels increased
after obstructive jaundice but promptly decreased after both
biliary drainage groups. In the course ofproducing obstructive
jaundice, AKBR become lower by the fourth week of jaundice
and hepatic tissue blood flow decreased early after obstructive
jaundice. After internal drainage, decreased AKBR recovered
to normal after 6 weeks and decreased hepatic tissue blood flow
improved to normal after 10 weeks. On the other hand, after
external drainage decreased AKBR recovered to normal after 10
weeks and no recovery ofhepatic tissue blood flow was observed
after external drainage until 10 weeks. Conclusion
Internal biliary drainage was superior significantly (P<0.01) to
external biliary drainage in recovering decreased hepatic energy
metabolism and hepatic tissue blood flow.
P368 P369
PROLIFERATING CELL NUCLEAR ANTIGEN (PCNA)
EXPRESSION IN THE GALLBLADDER WITH
PANCREATICOBILIARY MAI_JUNCTION
H. Isozaki, K. Okajima, H, Hara, S. Sako, Y. Takeda, K. Fujii
Department of Surgery, Osaka Medical College, Osaka, Japan
Patients with pancreaticobiliary maljunction develops more frequently cancer
of the biliary tract. In the present study, we examined PCNA expression in
the gallbladder with pancreatobiliary maljtmction comparing to that without
maljunction.
(PATIENTS AND METHODS)
A total of 22 patients with pancreaticobiliary maljunction [8 patients
accompanied, with cancer of the gallbladder (MCA group) and 14 patients
without cancer (M group)] were studied. In addition, 23 gallbladder cancer
patients without pancreaticobiliary maljtmction (CA group) and normal
gallbladders of the gastric cancer patients (N group) were also examined as
controls. The PCNA expression of the cancer and rnucosa of the gallbladder
were examined by immunohistocbemical staining method using anti-PCNA
monoclonal,antibody (PC 10). Thenumber of positive cells in every 500 cell
were calculated and expressed as the PCNA-positive rate (%).
(RESULTS)
1) The PCNA positive rate in cancer lesions of MCA group (33.0%) did not
differ from that of CA group (31.6%). 2) The PCNA-positive rates of non
cancerous mucosa of the gallbladder in patients with pancreatieobiliary
rnaljunction (14.2% in MCA group and 11.6% in M group) were
significantly higher than those without maljunction (3.9% in CA. group and
1.5% in N group).
(CONCLUSIONS) The proliferating cell activity of mucosa of the
gallbladder was higher in patients with pancreaticobiliary maljtmction than in
patients without maljunction. This may be one of the explanation of high
incidence of gallbladder cancer in patients with pancreaticobiliary
maljunction.
AGENESIS OF THE GALLBLADDER WITHOUT BILIARY ATRESIA:
REPORT OF 2 CASES
A. Iuppa, G. Bellipanni, C. Ravagli, G.A. Petralia, G. Catania
Department of Surgery, Division of General and Oncological Surgery,
University of Catania, Cataia, Italy
Agenesis of the gallbladderwithout biliary atresia is very rare: the reported
incidencerateis0,01-0,04%,only181 findingsinaseriesof1.352.000autopsies.
In about 50% of the cases it is associated with cardio-vascular, scheletrical,
genito-urinary or gastrointestinal malformations, the Authors report two
cases of atresia of the gallbladder in which US examination showed lithiasis
of the gallbladder. One case presented dilatation of the biliary tract and
underwent CT-scan examination that showed agenesis of the gallbladder;
diagnosticlaparoscopywasperformed.The othercasewasanintraoperative
surprise. None of them presented lithiasis of the biliary tract.
Itis interestingtonotice thatUSexamination, thatusuallyhas a specificityof
95-98%inthediagnosisoflithiasisofthegallbladder,showesalowsensibility
incase ofatresiaofthe gallbladder.Colangiographycan onlyshowexclusion
ofthegallbladder. Inmostofthecaseonlythepresenceofotheranomalies or
the familiarity for this malformation can elicit the suspect of atresia of the
gallbladder and justify a deeper diagnosic study.
164
P370 P371
COMMON BILE DUCT (CBD) LESIONS FOLLOWING LAPAROSCOPIC
SURGERY. PERSONAL EXPERIENCE
A. IuvDa, M. Migliore, G. Petralia, A. Sciuto, G. Catania
Department of Surgery, Division of General and Oncological Surgery, University
of Catania, Catania, Italy
The Authors report theirexperience ofthree lesions (0,46%) ofthe CBD occurred in
a series of 650 laparoscopic cholecystecomies performed from October 1991 to
November 1995 in their department. One was due to an incorrect dissection of the
Calot Trinagle, the other two occurred because of an anomal insertion ofthe cystic
duct. According to 1995 Strasberg's classification one lesion was type E2 and two
were type D (one ofthe common hepatic duct and one ofthe left hepatic duct). All
three,lesions were recognized intraoperatively.
Two have been repaired by laparotomy: one (type E2) by Roux-en-Y
hepaticjejunostomy, the other (type D) by suture ofthe lesion on aT tube stent. The
second case deserves a particular mention because it was due to a very rare instance
ofinsertion ofthe cystic duct in the lefthepatic duct, as no isolated lesions ofthe left
hepatic duct has been reported in the literature due to this exceptional anatomical
malformation.
The third lesion (type D on the commonbile duct) couldbe repairedlaparoscopically
by direct suture and intracorporeal ligation.
We had no mortality. The follow-up is 48 months for the type E2 lesion, 26 months
forthe type D and 12 months fotthe typeD ofthecommon bile duct. No clinical sign
of secondary stenosis of the CBD is evident.
OPEN CHOLESTECTOMY -ARE EFFICIENCY AND COST-BENEFIT
IMPROVEMENTS POSSIBLE FIVE YEARS' COMPARATIVE STUDY
Jeki6 IM, Milovaovi6 A, Zuvela M, Bilanovi6 D, Jovanovi6 M, Milicevic MN
The First Surgical Clinic, Clinical Center of Serbia, Belgrade, Yugoslavia
INTROD1JDTIOI The aim of the study was to analyze efficiency and cost-
benefit of open surgery in the management of cholelythiasis and to point out
possible improvements and prospectives.
MATERIAl.AND METHODS Two groups of patients were compared. Both
groups consisted of 200 consecutively operated patients, from an institution
where over 1000 open cholecystectomies are performed annually. Patients
from both groups were admitted and operated in a three month period, from
October to December, group A in 1989. and group B in 1994. There were 60-
78 (30-39%) men and 122-140 (61-70%) women Mean age was 55,5-A and
48,4-B, age ranging from 23 to 78. Mean hospital stay was 14,2 days -A and
10,6 days oB, ranging from 7 to 26 days. Average preoperative stay in group
A was 4,8 clays, while in group B was 3,2 days. More than one third of the
patients 35 %), from group A had preoperative stay longer than3 days,
while in group B that occurred in only 8 %. Average postoperative stay
exceed 9,4 days in group A, and 7,4 days in group B A repeated US was
performed in 82 pts. (41%) from group A, and in 58 pts. (29%) from group B.
Average use of infusion therapy was: for group A -saline solutions 3,5 and
glucose solutions 3,2 and for group B 2,3 and 2,0 respectively.
Thromboembolic prophylaxis was needed for an average 2,1 days in group
A, and 1,1 days group B. Most common complication was wound infection
in A 16 (8%) and B 11 (5,5%) patients. No early postoperative deaths were
observed.
DISCUSSION Considerable improvement in all analyzed parameters could
be observed. Preoperative stay is still too long due to insufficient diagnostics,
great number of repeated US, over 30%, and lack of organized admission
policy. Standardized and reduced medical therapy should also .be
performed. Antibiotics, when used indiscriminately boost hospital costs.
Improvements in the efficiency and cost benefit effects in group B resulted
from better scheduling and controlled use of hospital resources.
CONCLUSION Slandardized and accurate preoperative diagnostics, efficient
admission polic, clear concepts in prophylactic and therapeutic approach
and rational adjuant therapy should be observed to ensure improvement in
treatment.efficieftcy and positive cost/benefit effects.
P372 P373
SURGICAL TREATMENT OF BILIO-THORAC!C COMMUNICATION:
OUR EXPERIENCE
A. Kakouds, D. Kostarelos, D. Koufoudakis, N. Papalexandds
1st Surgical Clinic, Asklipieion General Hospital & Hellenic Red Cross
Hospital
Between 1964 and 1980, we operated upon 7 cases of biliothoracic
fistulas. Out of these, 6 cases were due to hydatid disease and was
caused by a common hepatic al:)cess. Five of the seven cases were
acute, while the other two progressed chronically. One was a bilio-
pleural fistula, whereas the remaining six were bilio-bronchial fistulas.
One of the ruptures occured in the left lung. Finally, one case was a
reccurence of a bilio-bronchial fistula, following a pulmonary Iobectomy.
Our technique consists of two stages. Dudng the first we perform a
decompression of the billiary tree, by draining the bile duct. After 9-12
days we .perform the second stage. This consists of a small
thoracotomy, which allows trans<llaphragmatic, drainage of the sub-
diaphragmatic space of the hepatic abcess. In our matedal we had one
postoperative death due to a pre-existing cardiac failure. In the
remaining 6 cases a cure was achieved. The hospital stay ranged from
55 to 155 days. Eventhough, this is a rare condition, the decline in
hydatid disease makes it all the less frequent, the problem still exists.
The major problem is that the more radical approaches such as
concurrent hepatectomy and pulmonary resection, have a high mortality
rate and do not eradicate the possibility of complications, such as
permanent fistulae. We believe that decompression and drainage of the
biliary tree is necessary because the basic pretense for formation ot
bilio-thoracic communication is an elevated intrabillary pressure and the
presence of inflammation. We believe that today, both stages can be
prformed in the same setting. The second stage can be subtituted by
closed drainage, with the help of thoracoscopy or CT assisted scanning.
HEPATIC RESECTION FOR BILIARY CARCINOMA IN THE ELDERLY
M. K, Y. Nimura, N. Hayakawa, J. Kamiya, S. Kondo, M. Nagino,
M. Miyachi, S. Mizuno
The First Department of Surgery, Nagoya University School of Medicine,
Nagoya Japan
To determine the significance of extensive surgery for the elderly, we
retrospectively evaluated the surgical results of hepatic resection for 41 patients
(age >70 years; the elderly group) with biliary carcinoma and compared their
outcomes with those for 158 younger patients (age <70 year; the younger
group).
Since 1979, hepatic reseCtionhas been performed in 199 patientg with advanced
carcinoma of the gallbladder (93 cases) or the bile duct (106 cases). Of 69
elderly patients with biliary carcinoma, surgical resection was possible in 46
(67%) and 41 (59%) of them underwent hepatic resection. These rates were
similar to those for the younger group (73%, 63%).
Major hepatic resection was done in 22 elderly patients (right tdsegmentectomy
4, extended right lobeetomy 13, right lobectomy 4, left trisegmentectomy 1).
The remaining 19 patients underwent smaller hepatectomy than right lobectomy
(extended left lobectomy 5, left lobectomy 3, central bisegmentectomy 1, right
anterior segmentectomy 1, right posterior segmentectomy 1, sub
segmentectomy 6, independent caudate lobectomy 2). Caudate lobectomy,
pancreatoduodenectomy and/or portal vein resection were concomitantly carried
out in 34, 10 and 5 patients, respectively.
Hyperbilirubinemia above 10 mg/dl and pulmonary complications occurred in
55% and 41% of elderly patients undergoing major hepatic resection,
respectively. These rates were significantly higher than those for younger
patients (p<0.05). Hospital mortality rate after hepatectomy was higher in the
elderly group (27% vs. 11%; p<0.05). In the elderly group, the hospital
mortality rate after smaller hepatectomy than right iobectomy was significantly
lower than that after major hepatectomy (5% vs. 46%). The l-year, 3-year, and
5-year survival rates after hepatectomy for 30 elderly patients excluding the
hospital mortality were similar to those for the younger group (72%, 36%, 16%
vs. 69%, 33%, 23%).
Morbidity and hospital mortality rates for elderly patients after hepatectomy were
significantly higher than those for younger patients. Mortality after smaller
hepatectomy than right lobectomy was acceptable even in elderly patients. Long
term survival after hepatectomy for biliary carcinoma in the elderly group was
satisfactory when comparedwith that in the younger group.
165
P374 P375
CONGENITAL MALFORMATIONS OF THE GALLBLADDER,
THE CYSTIC DUCT AND THE CYSTIC ARTERY
D.Katsikas, E.Misiakos, J.Kakisis, M.Safioleas, G.Karatzas
2nd Department of Propedeutic Surgery, Athens University
Medical School, Laiko Hospital, Athens, Greece
Congenital malformations of the gallbladder, the cystic duct
and the cystic artery are relatively common and related to
position, form or number of these structures. A retro-
spective study was carried out on 57 cases during the last
25 years in our Department, in order to delineate the several
types of these anomalies, the diagnostic evaluation and their
surgical management. Our material is consisted mainly of 4
cases of bilobar gallbladder, one case of double gallbladder,
9 cases of intrahepatic gallbladder, 3 cases of gallbladder
aplasia, 3 cases of double cystic duct, 2 cases of long
cystic duct, 2 cases of short cystic duct, 2 cases with ano-
malous cystic artery and 26 other cases with various ano-
malies. There was concomitant cholelithiasis in 34 cases
and choledocholithiasis in 9 cases. Clinical symptomatology
included upper abdominal discomfort, jaundice, nausea and
vomiting, hepatic colic or cholangitis. Diagnosis was esta-
blished preoperatively with ultrasonography and intravenous
cholangiography or was revealed incidentally at, operation.
Surgical management included simple cholecystectomy in
45 cases, T-tube insertion in 3 cases and cholecystectomy
with T-tube insertion in 6 cases. No major complications
occured intra- and postoperatively. Congenital malforma-
tions of the gallbladder, cystic duct and cystic artery should
be carefully diagnosed and explored surgically in order to
avoid serious complications during surgery of the extrahePa-
tic biliary tree.
DUODENOGASTRIC BILIARY
CHOLECYSTECTOMY 24-hr
MONITORING BILITEC 2000 ).
REFLUX FOLLOWING
SPECTROPHOTOMETRY
Kawiorski W., Herman R.M., Watega P.,
Ist Dept. of General and GI Surgery, Collegium Medicum
Jagiellonian Univesity, Cracow, Poland
Several clinical studies showed that Duodenogastric
Biliary Reflux (DGR) increased following cholecystectomy. The
role of this alkaline reflux has been controversial because of
the problems with its measurement. Now the new 24-hr fiber-
optic spectrophotometry monitoring system BILITEC 2000
has become available to assesse bile concentration.
The aim of present study was to evaluate the incidence
of gastric biliary reflux following cholecystectomy.
40 Patients with cholelithiasis were studied before and 6-8
weeks following ChC. Gastroduodenoscopy and 24-hour
spectrophotometric bile monitoring using Bilitec 2000 system
were performed in each patient. The results were computer
analyzed using Oesophogram, Synectics Medical software.
Results: Biliary reflux was observed during gastroscopy
in 50% of patients undergoing ChC. The incidence of DGR
episodes increased significantly in 31 76% of 40 patients
during 24-hour Bilitec monitoring. Total bilirubin absorbance
increased from 12.1% before ChC to 34.3% following surgery.
Conclusion" The incidence of DGR and total exposure of
gastric mucosa to biliary contents bilirubin significantly
increased following cholecystectomy. The ambulatory 24-hour
bilirubin absorption measurement system BILITEC 2000
seems to be an easy and recommended method of DGR
analysis.
P376 P377
THE TREATMENT STRATEGY OF THE GALLBLADDER AFTER
ENDOSCOPIC SPHINCTEROTOMY.
YCA Keuleman*, EAJ Rauws#, K Huibregtse#, DJ Gouma*. Academic Medical
Centre Amsterdam, *Department of Surgery, #Department ofGastroenterology.
Endoscopic sphincterotomy (ES) is increasingly used for patients with common
bile duct stones (CBD) with a gallbladder in situ. There is controversy however
about the different treatment strategies ofthe gallbladder: early elective (laparo-
scopic) cholecystectomy or a "wait and see" policy (gallbladder removal on
indication). It has been suggested that treatment is adjusted according to patients
characteristics. In young fit patients cholecystectomy is generally performed and
for older frail patients the "wait and see" policy has been accepted.
The aim ofthis study was to analyse iftherapeutic strategy ofthe gallbladder after
ES is indeed influenced by patient characteristics as age and co-morbidity or by
other factors as referral pattern (surgeon or gastroenterologist).
During Jan. Sept. 1995, 67 patients (mostly referrals) with CBD stones and a
gallbladder in situ underwent successfull ES and stone clearance in the AMC.
There were 29 men and 38 women and the mean age was 57 years (range 23-79
years). Three patients developed acute cholecystitis within one week after ES and
underwent cholecystectomy. From the remaining 64 patients, 37 patients (group
A) were planned for elective cholecystectomy and for 27 patients (group B) a
"wait and see"policy was selected by the referring physician. There was no diffe-
rence in age between group A and B respectively 57 years (range 23-78 y) and 60
years (range 29-79 y). Four patients in group B had severe contra-indications for
surgery. For the remaining patients in both groups co-morbidity expressed as the
mean ASA-score was not different respectively 1.5 versus 1.8.
During follow up 32/37 patients from group A underwent cholecystectomy after a
mean interval of7 weeks (range 1-18 weeks). Three patients were operated acute-
ly (cholecystitis) without complications and 29 electively with complications
(10%). The remaining patients are waiting for surgery (mean 25 weeks, range
10-40).
From group B 5/27 underwent cholecystectomy (19%) on indication without post-
operative complications. The remaining patients are without symptoms. Surgeons
advised cholecystectomy in 81% and gastroenterologist in 53% (p< 0.05).
Conclusion: Patients characteristics as age and comorbidity did not appear to
have much influence on treatment strategy ofthe gallbladder after ES. The choice
oftreatment seems to be more influenced by the referring doctor. A prospective
evaluation ofboth treatment strategies seems to be indicated.
A CASE OF BILIARY MUCINOUS CYSTADENOCARCINOMA
Hyung Chul Kim.M.D, Seun Young Kim, M.D, Chang Ho Kim, M.D
Chan Sup Sire, M. D" Young Sik Song, M. D
Department of General sugery, Internal medicine', College of
:licine, soon Chun Byang University, Chonan, Korea
Bi iary cystadenocarcinoma and cyst adenocarcinoma are
tumors which have a good prognosis after complete surgical
removal, Correct pre-operative diagnosis depends on the imaging
charicteristics of the tumors, Computed I;omography,
Ul trasonography, angiography and cholangiogram are useful
diagnostic procedure in bi iary cystic tumor but definite
diagnosis cannot be made without histologic diagnosis. Cytologic
examination of tissue obtained brushing the suspected tumor at the
time of PTC or ERCP helps to differentiate other disease from
carcinoma.The cysts are lined by mucin-producing columnar cells
with occation goblet cel Is, and are smooth, grayish-tan in
color, with relatively few loculations.The fluid within the cysts
is thick, mucoid, and viscous, often cloudy brown or hemorrhagic, and
may contain necrotic material.
Before the surgery, cholangioscopy is necessary for deciding
operation field.The prognosis of biliary cystic tumor seems to be
much better than that of other solid gepatic tumors. If there is no
evidence of metastasis,complete resection of these tumors is
therefore, necessary for these possibly curable disease.
Resently, we experienced a 60-year-old woman complained of
Jaundice and generalized itching sensation, which was diagnosis as
bi liary mucinous cystadenocarcincma. We decided operation field by
cholangioscopy.and performed left hepatic lobectomy and T-tube
choledochoJejunostomy.So, we report this case with a review of
relevant terature.
166
P378 P379
THE ANALYSIS OF RESIDUAL STONI APTER IPATIC DUCT
[IHDI OPERATION
J.H. Klm.. B.S. Kwak., J.B. Park., J.S. Park.
Dept. of Surgery, Christian Hospital, Kwnaglu, KOREA
This study ptuqmses to classify the patterns of IHD stones
Involvements In relation wlth hepatic resection and to evaluate the
availability of hepatic resection for the IHD stones treatment
through studying the characteristics of residual stones. 116
patients of our hospital received operations for IHD stones from
Jan., 1988 to Dec., 1994 and we kept the record of their residual
stones. IHD stones are classified into two types: localized type
and diffuse type. The former is defined by the occurrences of
stones in one or two segments In one or each lobe; the latter is
defined by the stones extensively scatteredin one or both lobes.
The localized type ts again bisected Into simple type and
complicated type. The simple type has unimpacted stones without
ductal stenosis In the st branch, while the complicated one has
impacted stones with ductal stenosis In the 2nd or later branch.
85 (73.2%1 cases had locallzed stones and 31 (26.8/6) cases had
diffuse stones (2.7:1 ratio). Among the localized stones cases, 25
(29.4%) were of simple type and 60 (70.5%) were of complicated
type. The choledocholithotomy was performed on simple localized
type, while the hepatic resection was performed on complicated
localized and diffuse types. The hepatic resection of 31 diffuse
cases was accompanied by drainage procedures. Post-operation
examinations observed occes of residual stones In 3 (9.6/61
cases of localized type In simple type and 2 In complicated
type) and 8 (25.8/6) In diffuse .type. After the hepatic resection,
residual stones were mostly localized Into one segment, and
common sites of residual stones were as follows: 3 (27.2/6) cases
In B4, 2 (18.1%) In B5 and another 2 (18.1%1 in B5 and B6.
The causes of the residual stones were as follows: missed stones
In 3 of simple localized cases and 2 (25/6) complicated type and
diffuse type cases, difficult approach In 4 (50%) eases and
Inadequete resection due to misdlagnosis In 2 1250/o) cases. The
conclusion Is that simple localized type of IHD stones can be
managed successfully by accurate resection of the hepatic
segnaent, whereas residual stones In cases of complicated localized
and difftme IHD stones can be mlnimld by an adequete
resection with drainage procedures under accurate preopative
diagnosis.
THE EFFECT OF PREOPERATIVE BILIARY DRAINAGE BY
ENDOPROSTHESIS ON THE INFLAMMATORY CASCADE IN
OBSTRUCTIVE JAUNDICE. A.N.Kimmings1'2' S.J.H van Deventer2,
E.A.J.Rauws3, I-I.Obertop1, K.I-Iuibregtse3, D.J.Gouma Department of Surgery
Inflammation Research and Depalent of Gastroenterology,Academic
Medical Center, Amsterdam, the Netherlands.
Postoperative septic complications are more frequent in patients with obstructive
jaundice (OJ) than in non-jaundiced patients. These complications are thought to
be due to an increased susceptibility to endotoxin, leading to the induction of
cytokines and the inflammatory cascade. Both the lack ofbile in the gut and the
.obstruction are thought to play a role. Internal biliary drainage in experimental
animal studies showed a clear reduction ofthe inflammatory response, with
significant reductions in"endotoxemia and, therefore, reduction ofcytokine
induction and restoration ofcellular immunity. On the other hand, endoscopic
drainage itselfcan result in a number ofprocedure related complications, such as
a cholangitis. The beneficial effects on the inflammatory cascade have not been
studied in the clinical setting.
The aim ofthis study is to determine the effect ofpreoperative biliary drainage by
endoprosthesis on the components ofthe inflammatory cascade as risk factors for
postoperative morbidity in OJ.
Patients (n=l5) were included with obstructivejaundice due to a 'resectable'
distal obstruction for which endoprosthesis placement by ERCP was possible.
Parameters (mean + SEM) were measured before stent placement (t=0), when the
patients werejaundiced, and after weeks biliary drainage (t=3). Drainage vas
adequate as measured by bilirubin levels at t=0 and t=3 weeks ofrespectively 244
tmol/l (34) and 50 (18) (p=0.002). Systemic endotoxin levels of4.3 pg/ml (0.5)
were not reduced after weeks and were 4.8 pg/ml (0.5) (NS), nor were most
cytokines, t=0 versus t=3: TNF 23.8 pg/ml (3.0) and20.6 (4.3); TNF receptor p75
7.4 ng/ml (1.1) and 6.2 (1.4); p55 3.1 ng/ml (0.5) and 3.0 (0.6); IL-6 3.7 pg/ml
(1.0) and 6.8 (3.0); IL-10 3.7 pg/ml (1.5) and 2.3 (0.9) (all NS). Only IL-8 was
significantly reduced from 118 pg/ml (19.3) to 24.5 (7.2) (p=0.002) Bacterial
cultures ofbile became positive in all samples after weeks stenting (p=0.001).
Conclusions: Preoperative biliary drainage by endoprosthesis, although adequate
to reduce bilirubin levels and other routine obstruction related parameters, did not
reduce endotoxin or most cytokine levels. This could be due to the fact that the
infected bile (cholangitis) after endoprosthesis placement itself leads to the
induction ofan inflammatory reaction.
P380 P381
CLINICOPATHOLOGICAL CLASSIFICATION OF PERIPHERAL
CHOLANGIOCARCINOMA COMPARISON WITH ARGYROPHILIC
NUCLEOLAR ORGANIZER REGIONS
Y. Kin, A. Yamaguchi, M. lsogai, A. Hori, C. Ando*
Department of Surgery and Pathology*, Ogaki municipal hospital, Ogaki,
Japan
We studied the clinicopathological features of nineteen consecutive patients
with peripheral cholangiocarcinoma (CC.) and classified four types according to
the micro-and/or macroscopic appearance. Type (n=3) indicated intraductal
papillary growth with superficial spreading, and was characterized by massive
mucin production and a comparatively better prognosis. Type 2 (n=4), the
periductal infiltrating type, showed a limited stricture of the intrahepatic bile
duct resembled extrahepatic bile duct carcinoma. Type 3 (n=10), the mass
forming type with ductal infiltration, was the most common type of the
peripheral CC and demonstrated the worst prognosis because of extensive tumor
growth and distant metastases. Type 4 (n=2), the mass forming type without
ductal infiltration, was characterized by intrahepatic metastases and abnormal
serum alpha-fetoprotein level similar to that in hepatocellular carcinoma. To
determine the degree of malignancy from the cellular activity of each type, we
used the argyrophilic nucleolar organizer region(AgNOR) technique to examine
various specimens. The mean numbers of AgNORs in CC were, type 1; 3.37
+0.03(mean_SD), type 2; 4.00___0.67, type 3; 4.30+0.52, type 4; 3.91_
0.06, respectively, while that in normal bile duct (control) was 2.11 +__0.25.
Although there were no significant differences among these types, the number
of AgNORs correlated to the histological malignant potential and patients with
specimens showing a high AgNOR count (>3.70) demonstrated a significantly
worse survival rate than those with a low count (p=0.032)., Furthermore, the
AgNOR count was a significant prognostic factor by Cox's multivariate
regression analysis. In conclusion, the differences in these four types of
peripheral CC could predict the extent of tumor invasion and curability of
surgical procedures, and the number of AgNORs would be a useful prognostic
predictor for peripheral CC.
SURGICAL MANAGEMENT OF BENIGN BILIARY
STRICTURES
V. Kopchak
Institute of Clinical & Experimental Surgery, Kiev, Ukraine
Over a period of 20 years, 587 patients underwent elective
surgical treatment for benign biliary strictures (BBS).
According to Bismuth's classification, type I-II of BBS occured
in 35%, type III-V in 65% of patients. Clinical presentations
included obstructive jaundice, pain, fever. Bacterial
contamination of the bile was universal. Endoscopic retrograde
cholangiography and transhepatic cholangiography proved to
be most effective among the diagnostic imaging techniques.
(Presence of BBS and obstructive jaundice considered to be
absolute indications for surgical treatment.) In patients with
severe suppurative cholangitis and hepatic failure (3,2%) the
treatment consisted of two stages: external biliary drainage and
subsequent reconstruction. Bile ducts were repaired in 25,6% of
cases, the rest of procedures were reconstructive. The insertion
of transhepatic stent tubes (ITST) was used in 53% of
operations. To prevent the development of stenosis and reduce
the duration of ITST anastomoses were dilated through
transhepatic route. Mean time of ITST with dilatation was 7
months versus 16 months in patients without dilatation. In 28
cases the reconstruction was performed using metallic net
prostheses. On the whole, mortality rate was 4, .2%. During the
follow-up, 6,4% of patients developed recurrence of BBS and
required reoperation. Thus, satisfactory results can be achieved
in more than 90% ofpatients with BBS.
167
P382 P383
SURGICAL TREATMENT OF PATIENTS WITH
BILE DUCTS CYSTS.
V.Koubishkin, V.Vishnevsky, D.Ionkin,A,Tchjao,
M.Muhammad, A.Vukolov.
A.V.Vishnevsky Institute of Surgery,Moscow,Russia.
The results of surgical treatment of 22 patients with
bile duct cysts are presented.Age range was 16 to 62
yrs. According to Todani's classification 16 pts. had
type cysts, pt.-. type IYA, and 5 pts.- type Y cysts.
All but two patierits had signs of the desease from
childhood. Six pts. had previous nonradical
operations for their cysts. Radical resection of cysts
with subsequent hepaticojejunostomy was perfomed in
16 pts., hepatic resections in 5 pts., and explorative
laparotomy in pt. One patient died postoperatively,
and 4 had anastomotic leaks. Morphological
examination showed malignant transformation of
cysts in 5 pts./22,7%/. Early diagnosis and
radical treatment of bile duct cysts are very important
in view of the high rate of malignant transformation of
biliary cysts.
RISK FACTORS IN SURGERY FOR CALCULOUS
CHOLECYSTITIS
N.A.Kouznetsov, N.M.Kusin
N.N.Burdenko Elective Surg.Clin. I.M.Sechenov Med.Acad.,
Moscow, Russia.
The abstract analyses experience of the N.N.Burdenko Elective
Surg.Clin. in surgical treatment of cholelithiasis. A total of 5017
operations for planned order; 14,3% of patients who underwent
operation 65 years of age and-older.
Cholecystectomy was expanded to choledocholithotomy in 2.7%
papillosphincterotomy in 1.7% separation of biliodigestive fistules in
0.6% of cases. Various combined operation were carried out on 558
patients, tntraoperative complications developed in 0.96% of cases;
damage to the hepaticocholedochus (0.14%) and to the hepatic artery
proper (0.2%) and its right branch (0.2%). Relaparotomy was
performed in 0.86% of cases; for bile leakage (054%) and for bleeding
(0.15%). Suppuration occurred in 3.9% of patients who were
operation on. Total mortality was 0.25% (0.09% after planned and
5.7% after emergency operations). Fatal complications were
encountered in 0.1% of patients under 65 years o.f age and in 1.18% of
older patients. Fatal outcomes occured in 1.1% of 558 combined
operations, one of which was cholecystectomy; in none of the cases
could the fatal complication be connected with expansion of the
intervention. As it can be seen from the above-discussed material,
there are definite prospects for improving the results of
cholecystectomy: an obligatory condition is conduction of the
operation in a planned order and under 65 years of age.
Careful assessment of the operative risk factors for each patients on
the basis of modern mathematical methods will help in solving the
problem of the possibility of surgical treatment. Such objective
assessment of the risk factors will make it possible to reduce the
indices of fatal complications to minimal and to bring operative
mortality to zero.
P384 P385
DURATION OF THE EXTERNAL COMMON BILE DUCT
DRAINAGE
V.Krasauskas G.Tikus, T.Perkauskas
First Clinic of Surgery, Kaunas Medical Academy, Kaunas,
Lithuania
In recent decades the development of endoscopical methods of
investigation and treatment of the bile ducts has decreased the role
of the external common bile duct drainage(ECBDD), but in some
cases it is inevitable. Mostly it is biliary obstruction and cholangitis
caused by choledocholithiasis. The duration of maintenance of the
ECBDD varies from to 4 weeks. We analyzed two consecutive
groups of patients from two departments of general surgery. In the
first group there were 52 patients, in 37(71,2%) of them T tube was
used for ECBDD and in 15(28,8%) cases a simple drain was
inserted through choledochotomy or cystic duct. The second group
consisted of 54 patients, 32(59,3%) of them with T tube and
22(4,7%) with a simple drainage tube. In all cases subhepatic drain
was inserted during the operation, but in the first group it was
taken out on the 2-4 th day after the operation and in the second it
was extracted later than drain from the common bile duct. The
ECBDD was maintained for 20,5 + 2,1 days in the first group and
15,9 + 2,3 in the second. Cholangiograms were performed in all
cases before the removal of the ECBDD and the regression of
jaundice was achieved. After the removal of the ECBDD we
observed complication(biliary peritonitis) in the first group
(1,92%) and 10(18,52%) in the second (3 biliary peritonitis and
pain, fever, leakage of bile through subhepatic drain in 7 cases). We
suppose that the lower rate of complications in the first group
belongs upon the maximum deposition of collagen during the
process of healing in 21 day what correlates with the strength of the
tissues surrounding the drainage tube. In conclusion, if
cholangiography reveals no pathology, we propose 3 week period
as the minimal duration for the ECBDD, after which the lowest
complication rate can be expected.
CHOLANGIO-DUODENAL INTERPOSITION OF AN ISOLATED
JEJUNAL SEGMENT (CDI) AFTER CENTRAL BILE-DUCT
RESECTION
B. Kremer, F. Reibe, I. Vogel, H. Grimm, D. Henne-Bruns
Department of Surgery, University ofKid, Kiel, Germany
After central bile duct resection bilio-intestinal drainage is routinely
perfommd by a Roux-en-Y-Ioop. Cholangio-duodenal interposition of an
isolated jejunal segment offers the benefit of a potential endoscopic
control and intervention of the bilio-intestinal anastomosis during the
patient's follw-up.
Between 1989-95 in 33 cases (central bile duct carcinoma n=22, benign
strictures or choledodal cysts n=l 1) the cholangio-duodenal drainage
was reconstructed after central bile duct resection by interposition of a
15-25 cm jejunal segment.
In 18 cases the first endoscopic control was performed before being
discharged, h 8 ot these patients the bilio-intestinal anastomosis could be
investigated, in 10 cases endoscopy was incomplete because of a kinking
ofjejunal segments.
During the follow up 11 patients died due to the extrahepatic tumor
recurrence. 3 patients with reconstruction of severe iatrogenic bile duct
injury developed anastomotic strictures. 2 patients were treated by
endoscopic piKmil drainage, one percutaneously.
Conclusions: Due to the publication of Shamberger et al. cholangio-
duodenal reconstruction byjejunal segment interposition may be affected
by a higher rate of cholangitis compared to the Roux-en-Y-technique.
Only shorter segments (up to 15cm) provide the benefit of endoscopic
follow-up. Subsequently we conclude that: 1. jejunal segment
interposition as bilio-intestinal reconstruction should only be used in
patients with high risk ofanastomotic restenosis due to tumor recurrence
(Klatskin-carcmoma) or risk of cicatricial stenosis; 2. only short
segments should be used and 3. repeated laboratory investigations are
necessary in order to detect biliary obstruction early.
168
P386 P387
GASLESS LAPAROSCOPIC CHOLECYSTECTOMY USING A SIMPLE
ABDOMINAL WALL LIFTING DEVICE
N. Kurauchi, Y. Ito, T. Sato, I. Onodera, R. Ishizuka
Department of Surgery, National Nishisapporo Hospital, Sapporo, Japan
Recent advantages of gasless laparoscopic surgery have been
provided by expensive or intricate instruments. A retrospective study
was carried out on our initial experience of 32 gasless laparoscopic
cholecystectomies using a simple abdominal wall lifting device to
ascertain the feasibility of complete surgery. The simple device is an
400-mm long and 10-mm wide flat bar acutely angled at the centre.
The abdominal wall distention for laparoscopic procedures was
obtained by positioning the device into the abdominal cavity through
a 15-mm periumbilical incision and retracting the device to lift the right
lower ribs. The operating field was easily created by lifting the
abdominal wall with the bar and the ribs. In all cases from September
1994, the operating field was smaller than that under
pneumoperitoneum especially in the obese body, but enough wide to
perform cholecystectomy and bile duct exploration in 2 cases.
Intracperative convertion to open surgery was neededin 2 cases,
because of severe adhesion in one case and disorientation of biliary
tract in another case. No complication related with this procedure was
observed. We. conduded that the simple abdominal wall lifting device
would contribute to gasless laparoscopic cholecystectomy except for
seveobese patients.
EFFECT OF CHOLESTYRAMINE ON THE FORMATION OF PIGMENT GALLSTONE IN
HIGH CARBOHYDRATE DIET-FED HAMSTERS
Y.C. Lee, D.K. SonE, 3.S. Ki C.S. Choi
Department of SurEery, Hallym University, Seoul, Korea
HiEh carbohydrate diet(hiEh CHO diet) is known to increase the
piEment Eallstone formation in hamsters, but the mechanisms has
not clarified yet.. It is postulated that hiEh CHO diet is poor
cholecystokinin stimulator, therefore it causes relative bile
stasis and increases the piEment Eallstone formatior Bile salts
exerts neEative feedback action on cholecystokinin, and
cholestyramine, bile salt sequestrant, results in enhancement of
the release of cholecystokinin and pancreatic protein secretion
with administration of amino acids. This study was desiEned to
investiEate the effect of cholestyramine on the formation of
piEment Eallstone and whether that effect occurred throuEh
cholecystokinin action. Forty seven hamsters were divided into
three Eroups: Eroup I(n=16} was fed on normal rodent cow(43
carbohydrate), Eroup II(n=14) was fed on hiEh CHO diet(6S
carbohydrate), Eroup III(n=17) was fed on hiEh CHO diet containing
426 cholestyramine. Animals were sacrificed after 6 weeks and
stones were observed Erossly. Gallstones developed in 0 of group
I, 4a.9 of group II and S.9 of Eroup III(P<0.0S, group II vs III).
To evaluate the chronic status of cholecystokinin level, the wet
weight of pancreas and the average area of pancreatic acinar in
microscopic high power field were measured, but there was no
siEnificant difference between group II and group III in
pancreatic weight and average area of pancreatic acinar(P>0.0S). In
gallbladder bile analysis, there was also no siEnificant
difference between group II and roup III in cholesterol,
phospholipid, total calciu total bilirubin and bile acid level.
In conclusion cholestyramine decreases the frequency of pigment
gallstone formation in high CHO diet-fed hamsters, but it is not
clear whether the mechanism, of cholestyrsmine decreasing the
Eallstone formation is through the action of cholecystokinin.
P388 P389
EVALUATION OF THE SO-CALLED "MINI-LAP"CHOLECYSTECTOMY
IN THE TREATMENT OF GALLSTONE DISEASE
Anatol Lemieszewski Marian Pardela
2nd Department of General & Vascular Surgery, Silesian Academy ofMedicine,
Zabrze, Poland
The introduction oflaparoseopic choleeysteetomy with minimal access surgery
had a tremendous impact on surgeons' way of thinking. "Mini-lap"
eholecysteetomy has been promoted as an exellent alternative procedure to
conventional, and mainly laparoseopic choleeystectomy, because it offers
minimal invasion without the disadvantage ofvery expensive equipment and the
need ofa special training.
Since 1985 we have performed 1061 choleeysteetomies for gallstone disease.
Until September 1992 the gallbladder had been removed using conventional
technique. Then, the "mini-lap"method was introduced as an alternative to
laparoscopic surgery for elective, uncomplicated eholelithiasis. The group of 41
patients undergoing this procedure, included 32 women and 9 men, whose age
ranged from 19 to 73. Patients with symptomatic cholelithiasis were operated on
through this access after the diagnosis had been confirmed by ultrasound
imaging. "Mini-lap" was not performed in the eases hydrops, empyema or
eholecystitis were suspected. Five eentimetres long skin incision was performed
along right upper transreetal line.
Cystic duet and cystic artery was ligated with surgical hemostatic clips
(Surgiclip), and the gallbladder was removed retrograde with the exa6t
hemostasis ofthe gallbladder bed by means ofargon-beam coagulator.
Extrahepatic biliary duets were manually controlled, the tube was placed into the
foramen Winslowi for adequate drainage, and then abdominal wound was closed
using automatic stapler device (Signet-12 or PPW55). The average length of
hospitalization was 2,5 days. In the laboratory investigations we confirmed no
deviations, and we noticed no early and late complications.
1. Small skin incision greatly improves the final cosmetic effect, comparable to
laparoseopie eholecysteetomy, and allows manual control of extralmpatie biliary
tract.
2. Use of surgical hemostatic clips, argon-beam coagulator, and automatic skin
stapler, significantly facilitates and shortens removal ofthe gallbladder.
PRIMARY SCLEROSING CHOLANGITIS, ENDOSCOPIC
DIAGNOSIS AND TREATMENT
S. Linder, C. Sfderlund.
Department of Surgery, Stockholm S6der Hospital, Stockholm,
Sweden
Primary selerosing ch.olangitis (PSG) is characterized by multifocal
strictures creating a 'beaded"appearance of the biliary tree. The
disease is located intrahepatically but the extrahepatie biliary tract
is often affected. Diagnosis may be settled by endoscopic
retrograde eholangiography (ERC). In PSC the incidence of
eholangioeareinoma is increased and the differential diagnosis may
be difficult. ERC also offers therapeutic possibilities.
Methods. Seventeen patients, 13 men and 4 women underwent
diagnostic and/or therapeutic ERC. Endoscopic treatment was
attempted when dominant strictures in the hilum of the liver and/or
the common bile duct were encountered. Co-axial dilatators,
balloons and endoprosthesis were used.
Results. Twelve patients had ulcerative colitis. PSC was already
diagnosed in 8. All patients had abnormal liver function tests and 9
had clinical symptoms. Endoscopic therapy was considered
possible in 11. Co-axial dilatators were always used, in 7 also
ballons. Endoprosthesis were inserted in 6. Bile duct width
improved in 8, liver values in 3 and'clinical improvment was noted
in 4 patients. Cholangitis occurred in 4 patients and 2 had
progressive jaundice. Cholangioeareinoma was diagnosed in 6 (1
cancer in situ) and colon cancer in 2.
Conclusions. ERC is the diagnostic method of choice in PSC.
Endoscopic therapy may be used in selected eases. Some
immediate beneficial effects may be acb.ioved but the results in the
long run are uncertain. With a high incidence of cancer in PSC
efforts must be focused on diagnosing these patients and to identify
patients "at risk".
169
P390 P391
STAGING OF KLATSKIN TUMOR BY US, DOPPLER-US
AND CT: COMPARISON WITH OPERATIVE FINDINGS.
F Loria, S Cantoni*, T Centomno, C De Renzis, G Loria, G
Ascenti, A Blandino, P Frosina, E Scribano, C Frola*.
Ist. Radiologia Univ. Messina-*IV Div. Radiologia Osp. S. Martino,
Geneva
The aim of radiologic.al investigations is the preoperative assessment
of the resecability of the tumor.
35 patients with pathologically proved Klatskin tumor underwent US,
Doppler-US and CT. PTC and/or ERCP were performed in all
patients, celiac and mesenteric angiography in 10/35. Imaging
findings were compared with operative findings.
The level of intrahepatic biliary obstruction was correctly determined
in 100% of patients with US and CT. Portal occlusion and wall
infiltration were correctly diagnosed by US and Doppler-US in 4/4
(100%) and 15/18 patients (83%) respctively, by. CT in 4/4 (100%)
and 13/18 (72%) patients respectively. Hepatic artery involvement
was detected by US and Doppler-US in 3/7 cases (43%), by CT in
4/7 cases (57%). Nodal, hepatic and peritoneal metastases were
revealed by US and CT in 3/8, 2/3 and 1/3 patients, respectively.
US and CT compared with operative findings and PTC,
demonstrated a sensitiviy of 100% of biliary involvement. Portal
occlusion and wall infiltration were diagnosed by US and Doppler-
US in 100% and 839(, of patients, by CT in 100% and 72% of
patients. Resecability was correctly diagnosed in 28/28 cases. US and
CT may be valuable in the preoperative staging of Klats""fifin tumor,
specially in predicting ductal and portal involvement and contribute to
avoid laparatomies in patients who can benefite from biliary drainage.
PERIOPERATIVE SURGICAL COMPLICATIONS IN 150
VIDEOLAPAROSCOPIC CHOLECYSTECTOMIES
].af,axelli_ Dallatana R, Maltempi P, Confalonieri MA and
Calzoni D.
Surgical Department, S.Carlo Borromeo Hospital, Milan.
Videolaparoscopic (VLS) cholecystectomy is fast becoming the
procedure of choice for the treatment of symptomatic gallstones
and gallbladder benign neoplasm.
We report our experience during the last three years about 150
VLS cholecystectomies performed in 100 women and 50 men
aged from 18 to 78 years (mean 46.8), affected by cholelithyasis
(146 cases), gallbladder polyposes (3) and adenomioma (1). 28
of the 146 cholelithyasis were cholecystitis; 7 patients were
submitted to Endoscopic Sphincterectomy (ERCP-S) to remove
choledochal calcolouses before surgery. Pathologic analysis
revealed in a cholecystitis the presence ofa little carcinoid tumor.
We analysed the pedoperative complications and their
treatment. We observed 7 cases of bleeding, 4 discovered
intraoperatively and 3 immediately after surgery. 6 ofthem were
undergone to immediate laparotomy and a gallbladder fossa
hematoma was treated conservatively. One patient had jaundice
due to Odditis and was treated with ERCP-S. Two cases had
ombelical wound infection. Conversion to open cholecystectomy
was required totally in 14 cases. Results are similar to those
reported in litterature. Adeguate surgical training, expertise and
respect to the safety ofthe patient are mandatory. In our opinion,
hemorragic complications are to be treated with laparotomy.
P392 P393
ACUTE CHOLSTITIS: IMPORTANCE OF OPEN SIEY IN THE
LAPARPIC ERA
P. I, J. , M. , A. , F.
-
G, F. I
D OF , II OF IC, I-
, AIN
Yeais ago, the urgent surgical indication of Acute
Cholecystitis (AC) was in deep controversy with the
deleted indication. Today, we observe the same dilemma
between the advantages of using the laparoscopic via
opposite to open surgery.
We introduce the results obtained from an urgent sur-
gical attitude in AC using the laparotomic via; this
based on a prospective analysis obtained from patient
fine records. From a total of 154 diagnoeed patients
in a chronological period of 4 years, 136 had a sur-
gical treatment and 18 a medical one. The stoical
exclusion criteria was anesthesic. The average age for
both sex was 70,8 years old. Average post-operative
stay was 7,72 days. No kind of major post-operational
complications were observed. Average age of not opera-
ted was 76,55. Total average stay was 10,3 days. 2 ca-
ses of death between not operated. The results show
that in spite of being AC a pathology of elderly
patients and with a serious and important diseases,
an urgent surgical attitude and the use of open sur-
gery do not increase the morbi-mortality. Further com-
parative studies regarding age of patient and degree
of evolution of operated AC, will prove the advanta-
ges, if any, of one via over the other.
170
LAPAROSCOPIC CHOLECYSTECTOMY.
DOES IT EFFECT BILE REFLUX?
G.I. Madderno P.S. Baxter
Department of Surgery, The Queen Elizabeth Hospital, Adelaide,
South Australia
This study evaluated the effect of laparoscopic cholecystectomy for
cholelithiasis on bile reflux. Sixty six patients (43 females) fasted for
6 hours prior to intravenous injection of 80 100 MBq of the radio
pharmaceutical agent 99Tcm-DIDA. After 30 minutes, scans were
carried out using a 'mobile 300 rrrn diameter digital gamma camera
with an cn board computer. Patients were seated upright and a
minute control view of the abdominal field was obtained. After 300 mls
of full cream milk, minute serial abdominal views were taken over
the next hour. Areas of bile reflux were seen as a dense focus of
accumulation of activity in the left upper quadrant, distinct from liver
and bowel and were mapped at the time of maximal definition and
expressed as a percentage of the total abdominal activity recorded by
the control scan.
Patients underwent cholecystectomy after a median of 28.5 days (range
8-588 days) following the study and those who had an uncomplicated
post-operative course were re-investigated with a milk-DIDA scan a
a median time of 50 days (range 18-370 days) post-operatively. Fifty
seven patients underwent laparoscopic cholecystectomy and 9 open
cholecystectomies were performed.
Sixteen patients had bile reflux before cholecystectomy and 25 after.
Thirty five patients had no bile reflux both pre and post
cholecystectomy and of those who had no reflux before cholecystectomy
13 had positive reflux after cholecystectomy with a 4.3% median
increase of reflux (range 1.2%-10.6%). Twelve patients recorded reflux
on both occasions with 7 increasing post cholecystectomy (median 1.4%,
range 0.4-8.7%) and 5 decreasing (median 1%, range 0.2-4.2%).
Laparoscopic cholecystectomy did not have any significant effect cn
the anunt of bile refluxed post operatively nor was there any
significant difference with patients who had open cholecystectomy.
We conclude that cholecystectomy has no effect on bile reflux.
P394 P395
GALLSTONE ILEUS: ANALYSIS CF 11 PATIENTS AND
LITERATURE REVIEW.
R.MadJov,P.Chervenkov,P.Arnaudov,K.Georgiev
Dept. Surgery,Med. Unlversity,Varna,Bugaria
Gallstone ileus is rare but serious complica-
tion of Cholelithiasls. Out of a total 2643 bi-
liary operations we had performed I| operations
for gallstone obstruction of the intestine
(0,42%).It is most common a geriatric surglcal
emergency 8 of all the 11 patients were over
65 years of age.
Preoperative diagnosis was exact only in four
patients. All the patients had clinical, labo-
ratory and radiologic findings for acute intes-
tinal obstruction and required emergency surge-
ry. Cholecystoduodenal fistula was the most fre-
quent type (found in 7 pts). Obstructing stones
were located most common in the terminal ileum.
In 8 patients enterotomy,stone removal and re-
lieve of intestinal obstruction was performed.
In 3 patients one stage operation was performed
holecystectomy, removal of the mpcted sto-
ne and flstula repair.
Enterolithotomy alone remains the mainstay of
urgent operative treatment for gallstone ileus,
but the additional performance of one sage
cholecystectomy and reapir of internal billary
fistula is desirable if local and surglcal con-
ditions allow it.
Gallstone ileus, like the other complications
of Cholellthiasis, remains a strong argument
for early choleCTStectomy in patients with
symptomatic gallstones.
Immunomorpholog/cal studies in gallbladder carcinomas
P.Mjewski. R.Marcinlak, M.Drews, J.Szmeja,M.Blczysko,
M.Rewiska, K.Matysiak
Department of Clinical Pathomorphology, HI Department
ofSurgery Pozna, Poland
We investigated 55 specimens of gallbladder
carcinomas, 46 female and 7 male cases, and performed
routine histopathological and immunomorhological
examinations localizing CEA,p53, PCNA, Ki-67 and lectins
PNA, UEA in tumor specimens.Age of the patients ranged
between 47 and 86 years old. We stated adenocarcinoma in 46
cases, mucus producing adenocarcinoma in 6 cases, carcinoma
mucocellulare in case and 2 cases of planoepithelial
carcinoma. We also evaluated tumor cell grading for
adenocarcinoma and stated 18 cases of O-1, 25 cases of G-2
and 9 cases of G-3. CEA, PNA, UEA expression on cell
membranes was characteristic for mature G-1 cancers, and
their cytoplasmatic localization was characteristic for
immature G-3 neoplasms (p<0,05). P-53 oncoprotein
cytoplasmatic expression found in 12 cases was characteristic
for immature G-3 adenocarcinomas(p<0,05).We did not found
correlation n nuclear expression of p-3 oncoprotein
and type oftissue localization ofPCNA and Ki-67 proliferation
markers in relation to tissue maturity of gallbladder cancer.
Conclusion: CEA,p53 and lectins PNA, UEA could be
immunomorphological maturity markers in gallbladder
carcinoma.
P396 P397
RESULTS AFFER OPEN COMMON BILE DUCT
EXPLORATION FOR CHOLEDOCHOLITHIASIS
A. Maleckas I. Toker, S. Butrimaviius, K. Kavaliauskas,
R. Jankovskij
st. Surgical Clinic of Kaunas Medical Academy, Kaunas,
Lithuania
A retrospective analysis of 96 patients for whom open common
file duct (CBD) exploration was performed during the period
1991 1994, in the Urgent Abdominal Surgery department of the
st. Surgical Clinic of Kaunas Medical Academy. There were 28
male and 68 female. I'he age ranged between 23 and 90 years,
mean age 63.8 years. The indications for the exploration of CBD
were jaundice, cholangitis, acute pancreatitis, diagnosed stones
that were not eliminated during endoscopic sphincterotomy (ES)
and dilated CBD found during the operation. In 79 cases the
stones in CBD were found and eliminated, and in 17 cases there
were no stones. After the exploration of CBD operations were
terminated performing in 2 cases (2% primary CBD suture, in 13
cases (13.5%) primary CBD suture with drainage through cystic
duct, in 46 cases (48%) CBD drainage through choledochotomy,
in 19 cases (20%) CBD drainage through cystic duct, in 15 cases
(15.5%) choledochoduodenostomy, in case (1%)
chbledochojejunostomy Postoperative mortality rate 10.4%.
Postoperative complications rate 25%. In 8 cases (8.3%) of all
performed operations were found retained CBD stones, in 8 cases
(8.3%) wound complications, in 3 cases (3.5%) septic
intraabdominal complications and in 5 cases (5.5%) pulmonary
complications.
Conclusions: There was rather high postoperative mortality rate
10.4%. Most of the patients were elderly ones and the CBD was
not cleared during ES. In these cases more conservative approach
must be applied trying to insert endoscopically CBD stents and
this can help to reduce the mortality rate.
CHOLELITHIASIS AFTER TOTAL GASTRECTOMY
B. Manasiievic, M. Stosic
Department of Surgery, Health Centre, Vranje, Yugoslavia
The purpose of this study was to evaluate whether total
gastrectomy (performed for gastric cancer) leads to an
increased risk of cholelithiasis. Also, should be consider a
prophylactic cholecystectomy, after total gastrectomy with
Roux-Y reconstruction. The exact mechanism by which
gastric resection can cause cholelithiasis is not yet clear,
although various patholehetic hypotheses have been
suggested. The most emphasized is gallbladder stasis
and an alteration in the enterohormonal mechanisms,
resulting from vagal denervation and the exclusion of the
duodenum from the alimentary transit.
A total of 14 patients who had undergone total
gastrectomy with Roux-Y reconstrucion (for gastric
cancer), between 1989 and 1994 were studied. One
(7,1%) of 14 patients had gallstones before surgery. TO
evaluate the prevalence of cholelithiasis, among other
examinations, we did an abdominal ultrasound every 4
months. Cholelithiasis have developed in 6 (42,8%)
patients followed after total gastrectomy. Four patients
had expressive clinical simptoms, one patient had
obstructive icterus and urgent operative treatment. The
median time to the devolopment of gallstones was 11
months (range: 4 to 54).
In conclusion, after total gastrectomy, there is an
increased risk of gallstones. That risk could be a reason
for preserving (when possible), the duodenal passage,
with jejunal interposition, after total gastrectomy. However,
when a Roux-Y reconstruction is performed, a
prophylactic cholecystectomy should be considered.
171
P398 P399
COMBINED APPROACH TO BILE DUCT STONES. RESULTS
AFTER ERCP FOLLOWED BY LAPAROSCOPIC
CHOLECYSTECTOMY.
C. Marco, E. Veloso, R. Almenara, E. Cugat, C: Hoyuela, P.
Collera, J. Espin6s.
Departments of Surgery and Gastroenterology. Hospital Mutua de
Terrassa, University of Barcelona. Barcelona, Spain.
AIM: To assess the efficiency, morbidity, and mortality of a
combined approach, endoscopic and laparoscopic, to treat suspected
common bile duct stones.
MATERIAL AND METHODS: Between April 91 and November
95, 1050 laparoscopic cholecystectomies were performed at our
institution. Of these, 133 patients presented clinical, ultrasonic or
biochemical anomalies suggesting choledocholithiasis and therefore
ERCP was performed preoperatively.
RESULTS: Common bile duct (CBD) was considered normal in 59
patients.. Stones were found in 64 cases, CBD was dilated but
without calculi in 10 patients and in 2 patients cannulation of
ampulla was not possible. Of 62 patient with confirmed CBD
stones complete extraction was possible in 58. In 4 patients
clearance was not possilble and were operated via laparotomy.
Endoscopists performed papilotomy in 23 patients without calculi
due to personnal criteria dilatation of the CBD, inflammatory
ampulla). Morbidity included mild pancreatitis (7), cholangitis (1),
choleperitoneum (1) and CBD perforation (1). There was no
mortality. In 121 patients ulterior laparoscopic cholecystectomywas
performed.
CONCLUSIONS: 1. Using classical criteria (clinical, biochemical,
ultrasonographic) accurate diagnosis of CBD stones was only
possible in 47% of patients.
2. ERCP, in our experience cleared effectively CBD stones in
93.5% of patients with low morbidity and without mortality.
PERCUTANEOUS CHOLECYSTOSTOMY FOR TREATMENT OF
ACUTE DISEASES OF THE BIUARY TRACT.
L. Mc Cormack, E. de Santibarms, J. Sivod, A. Domenech, J. Pekoli.
HPB Surgery Section, General SuRlery Service, Hospital Italiano, Buenos
Aires, ARGENTINA.
Between june 1989 and december 1995 were performed 61
percutaneous cholecystostomy (PC), 55 of them for treatment of acute
diseases of the biliary tract (ADBT) in elderly or critically ill patients as an
alternative to operative therapy. Twenty seven were men and twenty
eight women. The average age was 64 years old (r 19-93 years).
Indications were 46 acute cholecystitis (24 calculous (ACC) and 22
acalculous (AAC)) and 9 cholangitis. The PC was performed with
transhepatic access guided in 53 cases by US and by CT in 2, SeldingeCs
technique was used in 47 patients and trocar technique in 8. In 7 cases
the clinical course didnl improve after PC (2 gallbladder gangrene, 4
MOFS and cholangitis). The morbidity was 11% including 3
bacteremias one of them with hemodinamic and respiratow
compromise), hemobilia, vagal reaction and catheter dislodgment
without bile leaks requiring cholecystectomy 48 hours after PC. The
global mortality was 18% (10 patients) meaning 27% for AAC (6 patients),
12,5% for ACC 3 patients) and 11% for cholangitis (1 patient). There
was no mortality related to the method. In ten patients with ACC the
gallstones were treated by .percutaneous cholecystolithotomy, 6 were
operated (5 electively), 3 died and 5 didn't have definitive treatment.
Fourteen patients with AAC were cured without ,6 died and 2
required SURlery for gallbladdergangrene. In the group of cholangitis one
patient require complementary percutaneous blliary drainage and 4 died;
definitive treatment include 2 percutaneous choiedocholithotomy,
percutaneous drainage of obstructive parcreatic abscese and 2 elective
These results indicate that PC is safe and effective temporary method in
elderly and critically ill patients with ADBT, allowing to performed elective
surgery or bringing a percutaneous access for treatment of the causing
disease. In AAC the PC can be used as an inmediate and definitive
therapy and cholecystectomy can be avoid.
P400 P401
Choledochal Cysts, Study ofVariable Presentations,
And Value ofDifferent Diagnostic Methods
Twelve patients with different types ofcholedoehal cysts 4 males
and 8 females were included, age ranged between 7 and 40 years
(mean age 25.8 years). Allpatientswere presented with cholestatic
jaundice and recurrent attacks of cholangitis, 5 were
cholecysteetomized before being referred because of persistent
symptoms andjaundice. Allpatientswere evaluated clinically. Also
routine laboratory tests as well as liver profile were done
.Ultrasound and CT scan were decisive in one patient only.
Endoscopic retrograde eholangiopancreatography was decisive in
all patients, type choledochal cyst was found in 6 patients, type
II in 2, type IVA in 2, type IVB in one, and type V Caroli disease
in another one.
Associated diseases were gall stones in 5 cystolithiasis in 9 and
liver cirrhosis in 3.
Chronic pancreatitis was diagnosed in one patient with Caroli
disease had very bad general condition died in liver failure 6
patients were submitted to choledococysto
Jejunostomy (Roux-en-Y anastomosis), they did well. Another 3
patientswere submitted to cystectomy and hepaticojejunostomy.
Endoscopic retrograde cholangiopancreatography was the
diagnostic procedure ofchoice.
SELECTION OF OPERATIVE PROCEDURES FOR
ADVANCED CANCER OF PROXIMAL BILE DUCT
H. Miyake, S. Tashiro, S. Yogita, D. Wada, M. Harada, T.
Matsumura, Y. Fukuda, M. Ishikawa, K. Yagi, K. Mise, T.
Ohnishi
The First Dept. of Surgery, The University of Tokushima, School
of Medicine, Tokushima 770, Japan
We investigated the operative procedures lkr cancer of the
proximal bile duct. Eights patients (6 men and 2 women), ranging
in age fiom 45 to 73 years (mean, 64-1-11.3 year) underwent
operations in last 18 months. Patients with main tumor located at
theright hepatic duct or common hepatic duct (4 patients)
underwent extended right hepatic lobectomy and with main tumor
located at the left hepatic duct or common hepatic duct (4 patients)
underwent extended left hepatic lobectomy. All procedures
included resection of both the caudal lobe and the extrahepatic bile
duct. Vascular invasion of cancer vas evident in 2 patients. One
patient had invasion to the right hepatic artery and another patient
had portal invasion. These 2 patients undcrwent vascular
resection, too. Concerning metastasis to regional lymph nodes,
nodal involvement was evident in 5 patients out of 8 patients.
Three of 5 patients had nodal involvements of the parahepatic
artery and remaining patient had paraaortic lymph node
metastasis. In case of extended right hepatic lobectomy, we
preserve the upper part of the left medial segment and perform
right portal vein embolization (RPE) before operation to prevent
postoperative hepatic failure due to shortage of the remnant liver.
Out of 4 patients vho underwent extended right hepatic
lobectomy, RPE was performed on 3 patients and all patients vere
in good condition after operation but the remaining patients
vithout RPE died after operation due to hepatic lhilure.
In conclusion, hepatic lobectomy combined vith caudal
lobectomy and resection of the extrahepatic bile duct was
necessary lkr advanced cancer of .the proximal bile duct.
Furthermore, extended'lymphadenectomy had to be carried out.
172
P402 P403
UTILITY OF BIOC//gCAL TSTS AND NON'/NVP.glVE
A. naco, M. Vao, G. nce11,
Pallante, D.zzucco and R. Siani
Department of Surgery, Gaveno and Rvol Hospitals;
Service of Digestive Endoscopy, Rivoli H., Turin, Italy
The use of RRCP has facilitated accurate dlagnoss and
treatment in patients wth biliary tract dsease (.
Surg.1993;59:525-8)(.J;Surg.1993;165:474-8). The am
is to define, n biochemical tests and US data scans, a
factor able to predict a most judicious use of ERCP and
better selection of patients (pts) to this purpose. In
the past six months (may-october 1995), 79 consecutive
pts, 47 females and 32 males, ranging in age from 40-90
(mean 64.9) wth b[liary tract disease were successfully
studed by ERCP. In 49 (62) cases an operative ERCP
wth sphincterotomy (ES) was performed: 35 (71.4) stone
extractions" II (22.4) phlogistc papllary strctures"
2 (4) choledocal tumours and carcinoma of the
pancreas. Complication rates were: 5 cases of haemorrage
and perforation. The sphncterotomy was matched wth
clinical-presentation, US data scan (coon ble duct
dlatation), and various combinations of biochemical
abnormaltes (ser billrubin, transaminases and
amylase).The data were analyzed by Mantel-Haenszel test..
According to literature, no patient with simple
hyperamylasemia was studied by ERCP. No difference was
noticed at the admission between 36 pts with increased
serum transaminases and bilirubin undergoing ES and 19
pts undergoing diagnostic ERCP (respectively P=0.2;
P=0.4), just as it wasn't in the case of 22 pts wth
ncreased ser transaminases and bilirubin at the
moment of ES compared to II pts undergoing ERCP without
ES (P= 0.6). Not sgnifcant were the relationships
between simple increased ser transaminasemia and
blirubn and ES at the admission and at the moment of
simple ERCP (respectively P=O.98/P=O.41/P=O.86/P=0.29)
as well. The only. significant difference was noted
between 30 pts with CBD dlatation undergoin ES and
those with CBD dilatation undergoing ERCP without ES
(P=O.O00). In conclusion, US scan will detect CBD
dilatation as a factor of hepato-pancreatic disease
suggesting the use of operative ERCP, with ES.
SURGERY FOR ANOMALOUS ARRANGEMENT OF THE
PANCREATICOBILIARY DUCTAL SYSTEM WITHOUT DILATATION
OF THE BILIARY TRACT
Y, Murakami, T. Yokoyama, T. Kodama, Y. Takesue, Y. Matsuura
First Department of Surgery, Hiroshima University School of Medicine,
Hiroshima, .Japan
Pancreaticobiliary ductal diversion with excision of the gallbladder and dilated
biliary duct has recently been accepted as the operative method of choice for
anomalous arrangement of the pancreaticobiliary ductal system with dilatation
.of the biliary tract (AAPBDS with DBT), due to the risk of this disorder
developing into carcinoma of the biliary tract as a result of the regurgitation
of pancreatic juice into the biliary tract. However, for AAPBDS without
DBT, the maximum diameter of the extrahepatic biliary duct being less than
10 mm, it remains undetermined whether pancreaticobiliary ductai diversion or
cholecystectomy alone should be performed. We experienced 15 cases of
AAPBDS and 6 cases of them were AAPBDS without DBT. A clinico-
pathological study was carried out on 6 cases of AAPBDS without DBT to
determine the preferred operative method for AAPBDS without DBT. The
patients' ages ranged from 26 to 56 years and sex ratio of men to women was
1:5. All patients were diagnosed as having AAPBDS by ERCP or PTCD and
the maximum diameter of the extrahepatic biliary duct ranged from 5 mm to
mm. A Roux-en-Y hepaticojejunostomy with excision of the gallbladder and
biliary duct was performed for three patients. A gastrojejunostomy and
cholecystectomy with liver resection were performed for each patient with
advanced carcinoma Of the gallbladder. A gastrojejunostomy was performed for
patients with advanced carcinoma of the gallbladder. Subsequent
pathological findings showed hyperplasia of cases and adenocarcinoma of 4
cases in the gallbladder, and hyperplasia of 2 eases and dysplasia of case.
Considering the dysplastic changes of the extrahepatic biliary duct,
conclude that pancreaticobiliary ductal diversion with excision of the
gallbladder and extrahepatic biliary duct should also be performed for
AAPBDS without DBT.
P404 P405
ERCP IN DIAGNOSIS OF CARCINOMA OF THE AMPULLA OF VATER
A.Na9orni, J.MilanoviL D.Mitrovic, V.Katic, V.Pejic, 5.Petrovic-
Na9orni, T.Tasi4, M.Jeremi4, V.ivkovi, I.StQmenkovic, S.Tadi4.
Clinic for 9ostroentefo/ogff and/epotolo?ff, Clinic for sufgefif,
Clinic forpotholog/, Focult/ ofmedicine Nil, Nil, Yugos/ovio
Carcinoma of the ampulla of Vater is much less frequent than
carcinoma of the pancreas, gallbladder and extrahepatic bile ducts. It
is high curable carcinoma, but diagnosis is often made late. The aim
of this study was to analyse ERCP and histolosic findin6s in our
patients with ampullary carcinoma, preoperatively proved. There
were 21 patients, 12 men and g women. The ages ranged from 36 to
7'6 years (mean, 57.2 years). According to Tasaka three macroscopic
forms were observed: intramural protruding in 5 patients (23.8%),
exposed protruding in 8 patients (38.1%) and ulcerating type in 8
patients (38. 1%). Selective cannulation of the common bile duct was
performed in 20 patients and bile duct dilatation was demonstrated
in 16 patients (80%). This finding was associated with defect in
the ampulla in 6 patients. The normal bile duct was found in 4
patients. The cannulation of the main pancreatic duct was obtained
in 12 patients. Dilatation of the main pancreatic duct and side
branches were observed in 8 patients (66.7%). A stenosis of the
main pancreatic duct was detected in only one case. Forceps biopsy
were performed in 16 patients (exposed protruding and ulcerating
type) without the aid of sphincterotomy. There were 13 well
differentiated, 2 moderately and one poorly differentiated
adenocarcinoma. Conclusion: ERCP is the main diagnostic procedure
for car:inoma of the ampulla of Vater because it identifies the
location of lesions endoscopically, opacities the bilio-pancreatic
ducts and allows confirmation by biopsy.
LAPAROSCOPIC BYPASS OF PERI-DUODENAL
TUMOURS
LK Nathanson, IS Bailey, M Rhodes, NA O'Rourke, GA
Fielding.
Royal Brisbane Hospital and Wesley Hospital, Brisbane,
Australia
The majority of patients with irresectable peri-duodenal
tumours are treated endoscopically. Laparoscopic
surgical techniques now allow surgical bypass of peri-
duodenal tumours. Twenty-nine patients with:- ERCP or
stent failure (10), irresectable tumour at laparoscopy (9),
gastric outlet obstruction (7) or preferred surgery (3)
had:- 13 cholecyst-jejunostomies (CCJ), 8
gastroenterostomies (GE), 2 choledocho-jejunostomies
(CDJ) and 5 CCJ and GE. Laparoscopic cholangiography
confirmed cm + clearance of cystic duct, common duct
junction from tumour in 19/21 patients, 2/21 had CDJ (1
open and laparoscopic). Three patients required early
re-operation leak from CJJ 1, leak from GE and
twisted GE 1. Two patients died within 30 days of
surgery. Three of eight GE only patients developed
jaundice requiring treatment ERCPstent,
percutaneous stent and.1 laparoscopic CCJ. No biliary
bypass patients required re-intervention for recurrent
jaundice. Twenty-one of 29 patients have died during
follow-up survival, 75 525 days. Laparoscopic bypass
is a useful technique for palliating some patients with
peri-duodenal tumours.
173
P406 P407
LAPAROSCOPIC CHOLECYSTECTOMY VERSUS MINI-
CHOLECYSTECTOMY AND CLASSIC CHOLECYSTECTOMY
D.Nedelkovski,A.Jovchevski, K.Sekulovski
Department of Digestive Surgery, Military
Hospital, Skopje, Macedonia
Laporoscopic cholecystectomy is a currently
worldwide applied method of treating biliary
calculosis. In our hospital it was introduced
in clinical practice at the beginning of 1994.
The goal of our study is to show the relation-
ship of aparoscopic cholecystomy(LC) versus
mini-laparotomic cholecystectomy (MLC) with
incision of 5-7cm, and classic cholecystomy
(CC) with incision of 12-15cm.
From the beginning of 1994 up to July 1995,
during the period of 18 months at our Depart-
ment 591 chOlecystectomies were carried out,
of which 60 LC, 56 MLC and 475 CC.
The treated patients with calculosis in the
gallbladder were:455 with chronic inflammati-
on, 134 with acute inflammation of which:44
phlegmonous, 45 gangrenous, 28 empyema and 14
hydropic. The ratio female versus male was
4:1 respectively.
Analysed results show significant differences
in length of incision, operating time, opera-
tive difficulties (scale i-i0), hospital stay,
return to regular activities,wound cosmeses,
incidence of wound infections, possibility of
incisional herniae and visceroparietal adhe-
sions.
We can conclude that LC is an improved method
of choic.n treating non-complicated chole-
cystolithiasis, MLC has a better cosmeses
effect, while CC is appropriatewith infla-
mmations with pericholecystic changes.
COMPARISON OF BILIARY STENTING BY PERCUTANEOUS
TECHNIQUE VS COMBINED-ENDOSCOPIC APPROACH
P Ng, KP Wong, N Young, S. Williams
Department of Radiology, Westmead Hospital, Sydney, Australia
The purpose of this study was to compare biliary stenting by
percutaneous transhepatic placement (PTP) with combined
percutaneous and endoscopic technique (CP). A retrospective
analysis of 48 patients with PTP and 65 with CP between 1991
and 1994. Most patients had insertion of plastic endoprosthesis.
Results showed 85% of PTP and 94% of CP patients had
successful stent positioning across strictures. 76% of PTP and
58% of CP patients had a > 20% reduction in bilirubin within 5
days. 59% of PTP and 38% of CP patients required narcotic
analgesia post-procedure. 27%.of PTP and 33% of CP patients
suffered significant sepsis. 27% of PTP patients and 34% of CP
patients suffered stent blockage, at mean of 14 and 10 weeks
respectively. 12% of PTP and 33% of CP patients with hilar
strictures had stent blockage. Conclusions reached were that
PTP and CP have similar overall stent blockage rates, but CP
performs less well for hilar lesions. PTP has a higher drainage
success rate, but causes more pain than CP.
P408 P409
LAPAROSCOPIC AND ENDOSCOPIC MANAGEMENT
OF COMMON BILE DUCT STONES AND
OBSTRUCTIVE JAUNDICE
M. Nichitaylo
Institute ofAdvanced Medical Training, Kiev, Ukraine
Laparoscopic cholecystectomy (LC) has proved to be effective
method of treatment of cholelithiasis. In a period of 1993-
1995 LC was performed in 1570 patients, and among them
were 94 with common bile duct stones (CBDS) and
obstructive jaundice. Diagnosis of CBDS was established
preoperatively by means of ultrasound examination and
endoscopic retrograde cholangiopancreatography (ERCP) in
75 (80%) cases, in others ERCP was not performed (17) or
failed (2). When the presence of CBDS was confirmed by
ERCP, endoscopic papillosphincterotomy with subsequent
lithiasis extraction or mechanical lithotripsy and nasobiliary
drainage followed. LC was carried out 2-5 days later. Externil
biliary drainage via cystic duct was used in only 5 cases. One
patient developed cystic duct stump leakage due to clips and
required reoperation. In 6 patients CBDS were detected
during LC and trans-cystic CBD exploration and removed by
laparoscopic (4) or open (2) choledocholithotomy. No
complications were observed. In 13 patients CBDS were
indentified within 6 months following LC. Endoscopic
papillosphincterotomy was performed successfully in all cases
exept one, which required .open choledocholithotomy. The
mean hospitalization time was 4,9 days. There was no
mortality. In conclusion, results of LC and ERCP in patients
with CBDS and obstructive jaundice compare favorably with
those of conventional procedures with respect to mortality,
complications and length of hospital stay.
BILE DUCT INJURY DURING CHOLECYSTECTOMY IN THE
ERA OF LAPAROSCOPY
J. Nicolet, A. Sauvanet, J. Belghiti.
Department of Digestive Surgery, Hospital Beaujon, University Paris
VII, Clichy, France.
The development of laparoscopic cholecystectomy (LC) has been
associated with a rise in the incidence of bile duct injury (BDI). The
aim of this study was to assess the changes in the presenting features
and management of BDI in the era of LC.
Methods: All BDI cafes (primary or secondary referai) treatcd at our
center since january 1979 have been included for analysis.
Results: Between 1979 and 1989, 31 patients (average 2.8 per year)
were treated for BDI after cholecystectomy vs 35 patients from 1990
to mid-1995 (average 6.4 per year). Of thcse most rccent 35
patients, 16 (46%) have had an open cholecystectomy (OC) and 19
(54%) havc had a LC. In 9 (26%) patients, the BDI was discovercd
during the operation: 6 (38%) during OC and 3 (16%) during LC.
The presenting features in the 26 patients in whom BDI was not
discovered at the time of surgery were as follows
n Septic Biliary Biliary Emergency
s,ndrom fistula ascites rcopcration
of 10 (10%) 3 (30%) (10%) 3 (30%)
LC 16 7 (44%) 3 (19%) 7 (44%) 9 (5('5%)
Of the 16 (46%) patients undergoing an angiography, 9 (56%) had
lesion of the hepatic artery or of the portal vein; 4 (38%) after OC
vs 5 (71%) after LC. The type of biliary lesion according to
Bismuth's classification was identical, in the both groups. Endoscopic
treatment was not attempted in any case after OC but was attempted
in 5 (26%) cases after LC. Roux-en-Y hepaticojejunostomies was
performed in 11 patients (69%) after OC and12 (63%) aftcr LC.
Conclusion: this study confirms an increase in the incidence of BDI
in patients undergoing laparoscopic cholecystectomy although 50%
are still related to open cholecystectomy. Bile duct injury after
laparoscopic cholecystectomy are diagnosed later, notably through
septic and peritoneal complications, and are more frequently
accompanied by vascular lesions.
174
P410 P411
BILIARY MDTILITY DI_RDER AND FOOD ALLERGY
A. l No'aller, A.S. Lun]akov, A/A. Nizov
Medical university nane.d I. Pax lov,Ryazan, Russia
Dyskinesia of 'allbladder, chronical hepatobi-
liary and 'astrointestinal diseases are often
connected_ with aller'y to food p-,r.,dur'..to It. was
found in 203 of 380 observed pat.ients with food
allers"y. Prilry food allerp2y, especialy in chi-
ldren's a'e enables developnent of chro-nical
di'estive dise,es in future. Secondary food
alier'y w observed in 202 patients.
The perneability of hepate-intestinal barrier.
to protein-ovalbumin w inv@sti'ated by r,adio
immune nethod. Before and in 4 hours after the
load (3 dnp ets's) it w studied the concent-
ration of ovalbumin, I'G-immune complex and
t ibodies to ovalbumine in blood serum. The per-
n<eability of barrier and s-d.
p. of absorpti
of anti'en in food z,-. w increed.
Chronic uncalcuZous cholecystitis w observed
in 697 patients, calculous cholecystitis 2.,
biliary dyskinesia-in 18. 7t, chronic hepatitis
-in 1!. 3Z of ces. We used next thods
treat,nt: dietetic correction (elimination and
hyDosensibile diets, sDecial DPoducts without
ai!er'ens), antihistanine ndica,nts,
licat natrium, physical terapy <acu-, ler-and
electroDuncture, hyperbaric oxyenation, laser,
blood radiation, Dlasmapheresis, enterosor,bt
and ,thrs_,_ The ]iorobic
and health resor,ts theraDy were effective
etients with combination 0f' chronic cholecys
titis and food ailer'y. Aller%'ic echanis
cholecystitis and c,t.ilic disorders calls for
at.tention during" the treatment oF these
w. ent.s.
Laparoscopic treatment of mine bile duct stones.
Faillace; A.. Piazzini Albani; G. Peucchini; L. Campana.
D g
-S GO)
ep, e me i m oly, e
1 to v d gelic
ep F 1992 d e 1995,1041 ve lic o1
p p fdto have o1o1 d 82 p
g e -vve te. 39 pm mt ERCP d lic Umt
mfiy. Two pm 1 e c e 41 p e
lic oly. Two pm gve .Five
pm lic lm, vto mg.
lic oly r mto ee
w md c j. m, lm e
f ff a md lm W e lic 1 ff
e cm ff e lm, T pla md e ely cl
agtm. CP- prvely, e
cl pg Tbe.
of e 41 pm 21 mt i oloy e clue ff e
olc m, e 18 p de p T. e lm had
prov ep w ffe e ff e blab. o pm gve
m p
e 21 pm e age rov e vm y md
ye. epmo muto K's clue, eo1 age
rov 13 em.
e elly pmt & y f lg bluelY. pmt p
lic lmf be lage d opm p pveCP f
licUmt be eb m ofo1o1
e pmt r to n e f ys e m. ic
oly revole wld of g d e ffm
gbe atto revolphmm to off y mto
epmt: emt o1 tegp of "re" d e
hto be y amtmd lict b y
P412 P413
LAPAROSCOPIC CHOLEDOCHOTOMY SHOULD WE
AVOID T-TUBES AND CLOSE THE DUCT PRIMARILY?
NA O'Rourke, GA Fielding, IS Bailey, M Rhodes, T Nano,
LK Nathanson.
Royal Brisbane Hospital and Wesley Hospital, Brisbane,
Australia.
Prior to the advent of laparoscopic cholecystectomy
there was debate about the merits of a post
choledochotomy T-tube. As experience with
laparoscopic bile duct exploration has increased most
surgeons advocate T-tube drainage of laparoscopic
choledochotomies. During experience of 210
laparoscopic bile duct explorations we have questioned
this concept. Seventy-one patients had a completed
laparoscopic procedure with choledochotomy. A T-tube
was placed in 46 patients, the duct closed primarily in
18 patients, choledocho-duodenostomy (CDD) fashioned
in 4 patients and the duct closed over a double pigtail
stent in 3 patients. Eight of 46 (15%) of T-Tube
patients had significant T-tube related morbidity with 3
requiring further laparoscopy. One of 19 primary closure
patients required laparoscopy and a further suture on
the second post-operative day for a bile leak and 1/18
leaked bile for 4 days. No stented or CDD patient
suffered morbidity. Post-operative stay for primary
closure was 2 (1-4) days compared with 4 (1-21) days
for T-tube closure (p< 0.05, Mann -u- Whitney).
Primary closure of the choledochotomy with or without
a stent is now our preferred treatment.
CHANGES IN LIVER CIRCULATION FOLLOWING
THE REVERSE TRENDELENBURG POSITION
DURING COa PNEUMOPERITONEUM
M. Ohtake, T. Maruta, T. Sakaguchi*, T. Aono,
K. Tsukada, Y. Tamiya, K. Hatakeyama
Departments of Surgery and Physiology*,
Nfiata. University, Niigata, Japan
The effects of the reverse trendelenburg
poit.ion (rT) on liver circulation were
studied under CO pneumoperitoneum. Six
female .pigs were anesthetized with halothane,
and the portal venous flow and hepatic
arterial flow were measured by ultrasonic
volume flow meter after laparotomy. Abdominal
ws11 was then closed and the intraabdominal
pressure was increased step by step.
Pneumoperitoneum reduced portal venous flow
and hepatic arterial flow and increased
systemic arterial pressure, rT associated
with pneumoperitoneum further decreased the.
portal venous flow, but increased systemic
arterial pressure and hepatic arterial flow.
These responses are augmented in proportion
to the slope of rTo When the magnitude of rT
was fixed, increasing intraabdominal pr@ssure
did not affect hepatic blood flow. On the
other hand, rT with open laparotomy decreased
both po,al venous flow and hepatic arterial
flow, and it was found that reduction in the
flows by rT with open laparotomy was evident
than in rT with closed laparotomy. These
findings suggest that rT, which provides an
improved operative view, can be safely used in
the case of laparoscopic cholecystectomy
during pneumoperitoneum.
175
P414 P415
IMPROVEMENT OF EXTRACELLULAR WATER DEPLETION MARKERS
AFTER ENDOSCOPIC DRAINAGE IN OBSTRUCTIVE JAUNDICE.
FJ. Padillo, J.M Gallardo Valverde, M Rodriguez, A. Naranjo, P. Montilla, F.
Infante, A. Martin-Maid, M. Canis, G. Mifio, C. Pera, A. Sitges-Serra*
Departamento de Cirugia, Hospital Reina Sofia, Ctrdoba, and *Hospital del
Mar, Barcelona, Spain.
Experimental obstructive jaundice (OJ) has been associated with extracellular
water (ECW) depletion and paradoxical increase in atrial natriuretic peptide
(ANP) plasma concentratigns. ECW depletion predisposes to acute renal failure.
To investigate whether non-surgical biliary drainage results in reduction of
plasma ANP and repletion of the ECW, endocrine markers of volume depletion
were determined in 19 patients with obstructive jaundice before and after
endoscopic biliary drainage. There were 10 women and 9 men with a mean age
of 66 yrs (range 38-86). Six had benign conditions and 13 had periampullary
tumors. Patients were kept on a fixed i.v. solution regimen and enteral nutrition
intake. Plasma ANP, Aldosterone (Aid) and Renin (Ren) concentrations were
measured by radioimmunoassay before and on the 3rd to 7th day after
endoscopic biliary drainage. ECW was determined at the same time using
tetrapolar bioimpedance. Serum bilirubin and fractional sodium excretion were
also measured. Before biliary drainage, 94% of the patients had an elevated
ANP(>60 pg/ml) and mean Ren and Aid concentrations in the upper limit of
normal. After biliary drainage, serum bilirubin decreased (15+6 vs. 5+3 mg/dl,
P<0.01) and this was associated with decreases in ANP (114+45 vs. 79+45
pg/ml, P<0.01), Ren (54+77 vs. 20+20 tu/ml, P=0.13), Aid (170+93 vs. 98+67
pg/ml, <0.01) and fractional sodium excretion (0.59+0.29 vs. 0.84_+0.66, P=0.1).
ECW increased from 20+3% body weight to 22+_4% body weight (P<0.05). The
present study reports for the first time that plasma ANP is increased in patients
with OJ. After biliary drainage there is an increase in ECW and a parallel
decrease of the endocrine markers of volume depletion. These findings are
relevant to the perioperative management of patients with obstructive jaundice.
This work is supported by grant FISS 95/1369.
ROUTINE DYNAMIC INTRAOPERATIVE CHOLANGIOGRAM
(RIOC) AND INTRAOPERATIVE ULTRASONOGRAM (R1US)
DURING LAPAROSCOPIC SURGERY FOR COLELITHIASIS
A. Paganini, *F. Carlei, *D. Lomanto, F. Feliciotti, M. Guerrieri,
*M. Nardovino *M. Sottili, A. Tamburini, E. Lezoche
Istituto di .Scienze Chirurgiche, Universita di Ancona. *I.N.I.
Canistro, L'Aquila. Italy
Aim ofthis study was to prospectively investigate the role of RIOC
and RIUS during laparoscopic cholecystectomy (LC). From
December 1990 to November 1995 1348 unselected consecutive
patients underwent LC with RIOC. In 87 more cases ductal stones
were suspected or proven. Factors that were critical to the safety and
efficacy ofRIOC were: midclavicular trocar perpendicular and close
to the cystic duct, complete dissection of Calot's triangle prior to
RIOC, use of cholangiogram clamp and contrast solution at 125
mg/ml Iodine, dynamic study with filling of the entire biliary tree, no
more dissection after IOC. In 90 cases laparoscopic RIUS was also
performed with a 7.5 MHz rigid probe from the umbilical trocar. In
48 patients IOC was not performed because the fluoroscope was not
available. Of the remaining 1300 patients, in 39 (3%) IOC was
unsuccessful due to failed or improper cannulation (28), no passage
of dye (9), low cystic duct-CBDjunction. IOC was feasible in 1261
cases (97%) in an average time of 5 minutes 20 seconds (range 3-15
minutes). No CBD lesions were observed. Metal clips were too
close to the bile ducts and were removed in 15 cases. Anomalies of
surgical importance were identified in 29 cases (2.3%) and
unsuspected CBD stones in 68 (5.3%). Of the 90 patients who
underwent IOC and IUS, suspected CBD stones were identified in 2
and silent stones in 6 (by both exams in 4, by I.US only in 2). IUS
was limited by adhesions from previous surgery in 3 cases. Hepatic
angyomas were discovered in 2. Median time for IUS only was 5
min. RIOC after complete blunt dissection in the area of Calot's
triangle is a safe and effective, method to reduce the incidence of
major ductal lesions and identifies silent CBD stones. IUS is more
readily available, provides parenchymal informations and may be
complementary to IOC to correctly interpret radiologic artifacts, but
in our experience is not a substitute for IOC.
P416 P417
LONG-TERM RESULTS AFTER SINGLE STAGE
LAPAROSCOPIC TREATMENT OF GALLSTONES AND
COMMON BILEDUCT (CBD) STONES
/k. Paganini, F. Feliciotti, *F. Carlei, *D. Lomanto, M. Guerrieri,
*M. Nardovino *M. Sottili, A. Tamburini, E. Lezoche
Istituto di Scienze Chirurgiche, Universita di Ancona. *I.N.I.
Canistro, L'Aquila. Italy
From April 1991 to November 1995 151 patients underwent single
stage laparoscopic treatment of gallstones and CBD stones (females
95, males 56, mean age 55.1 years, age range 12-94 years, 40
patients over 70 years of age), in 102 through the cystic duct (no
biliary drainage in 75, trans-cystic drainage in 27) and in 49 after
choledochotomy (T-tube in 48). Operative mortality occurred in
patient (0.6%). Two patients died for unrelated causes
(thrombocytopenia and uncompensated cirrhosis, 2 years and
month after discharge, respectively). Of the remaining 148 patients,
37 (25%) are presently lost to follow-up, leaving 113 patients
available (75%), with a mean follow-up of 19.8 months (range 1-55
months), which included physical examination, laboratory exams
and ultrasound. Follow-up of more than 12 months is available in 81
cases. Residual CBD stones were observed in 10 cases (6.7%)
which were discovered at completion cholangiogram in 2, at pre-
dismissal cholangiogram in 5 and at follow-up in 3 cases. Residual
stones were removed 4-5 weeks postoperatively by ES in case and
by endo/fluoroscopic techniques through the biliary drainage sinus
tract in 6 (with ESWL in 1). Residual (recurrent?) stones were
removed by ES in 3 cases after 4-8 months, respectively. In case,
with prior hemigastrectomy, an asymptomatic 5 mm residual CBD
stone was discovered 18 months after laparoscopy and has been left
untreated. Vague abdominal pain with dyspepsia but no altered liver
tests has been observed in 30 cases (26.5%); 81 patients (71.6%)
are presently completely symptom-free with normal liver tests. A
single, self-resolving episode of biliary colic was reported by one
patient, with normal liver tests. Stipsis was reported in 5 cases.
Laparoscopic single stage treatment ofgallstones and CBD stones is
a safe and effective procedure that provides definitive cure ofbiliary
lithiasis with no long-term morbidity.
CO PNEUMOPERITONEUM VS GASLESS LAPAROSCOPIC
CHOLECYSTECrOMY
C. PALANIVELU MS Mch.. P S Rajan, S V Sivakumar,
K Sendhilkumar, R Parthasarathi, Dept. of Surgical
Gastroenterology, Coimbatore Medical College and Hospital,
INDIA.
Gasless laparoscopy can be performed safely without side effects ofCO
pneumoperitoneum particularly in elderly and preexisting conditions
such as cardiopulmonary dysfunctions. A prospective study was con-
ducted to evaluate the effectiveness of this technique. 30 consecutive
symptomatic cholelithiasis were treated by gasless laparoscopy and 30
with CO pneumoperitoneum. There was no significant difference in the
incidence ofsex, age andnumber ofacutecholecystitisin each group.This
study was conducted by single surgeon with experience of over 1500
Laparoscopic cholecystectomies.
Plannerliftsystemwasusedin all thecases. Periumbilical 1-2cmsincision
was made and through this laparofan was inserted and abdomen was lifted
by motorised laparolift. Valveless trocar sheaths were used. The same
periumbilical entry was used for laparoscope. Totally four ports were
made as describedin standardLaparoscopiccholecystectomy. Ports were
needed to be placed at low level compared to CO pneumoperitoneum.
Problems: 1) Less intraperitoneal laparoscopic space. 2) Movements of
intraperitoneal structures with varying respiratory phase. 3) Additional
ports are frequently needed for effective exposure. 4) Possibility ofhigh
incidence of thermal injury.
Advantages ofgasless laparoscopy 1) Use of conventional instruments
2) Suction and irrigation do not affect the laparoscopic space. 3) Extrac-
tion ofgall bladder is easy through epigastric port. 4) No change in cardio
respiratory status.
Conclusion Gasless laparoscopic cholecystectomy can be safely
performed and so is the preferred procedure of choice in high risk
patients..Due to technical difficulties this may be considered as an
alternative to pneumoperitoneum for others.
176
P418 P419
PANCREATICODUODENECTOMY FOR NEUROGENIC
TUMOUR OF COMMON BILE DUCT RARE TUMOUR OF
BILE DUCT.
C. PALANIVELU MS Mch.,. P S Rajan, S V Sivakumar,
K Sendhilkumar, R Parthasarathi, Dept. of Surgical
Gastroenterology, Coimbatore Medical College and Hospital,
INDIA.
Tumour of neurogenic origin is very rare in the bile ducts. A case of
schwannoma in the distal common bile duct is reported.
In March 1994, 23 year aged female had obstructivejaundice found
to have dilated common duct of4 cms in diameter with mass lesion in
the distal duct proximal to ampulla evaluated by ultrasound and
ERCP. Pre-operative histology could not be established.
US,CTscan, MRIrevealednoevidenceofspread. Pylorus preserving
pancreatico duodenectomy was carried out, Fleshy growth was seen
arising fromonesideofthecommonductjustproximal totheampulla.
No satellite nodules. No evidence of lymphnodes. Histology proved
to be schwannoma.
Schwannoma, a rare tumour of bile duct origin has been reported.
LAPAROSCOPIC REMOVAL OF IMPACTED E R C P
BASKET FIRST CASE REPORT
C. PALANIVELU MS Mch., P S .Rajan, S V Sivakumar,
K Sendhilkumar, R Parthasarathi, Dept. of Surgical
Gastroenterology, Coimbatore Medical College and Hospital,
INDIA.
Endoscopic sphincterotomy and clearance of stones is the preferred
procedure for common duct stones. Impaction ofERCP basket is one
of the rare complication and laparotomy is being done to remove the
basket. In April 1994, a case of impacted ERCP.basket was success-
fully removed first time by Laparoscopic method.
With CO pneumoperitoneum, common duct is exposed and
Laparoscopic choledochotomy was performed using microscissor.
ERCPbasketwas disimpactedanddelivered into theperitoneal cavity
using right angled forceps along with two stones and delivered out
through the trocar. After completion ofcholecystectomy, T-tube was
placed in the common duct and choledochotomy was narrowed with
vicryl stitches.
Impaction of ERCP basket is a rare complication and first case of
successful laparoscopic removal of impacted ERcPbasket has been
performed.
P420 P421
REVIEW OF BILIARY-ENTERIC ANASTOMOSES
RW Parks, GW Johnston, BJ Rowlands
Professorial Surgical Unit, Royal Victoria Hospital, Belfast,
Northern Ireland
Procedures involving biliary-entede anastomoses for benign and
malignant disease were retrospectively reviewed over a 9 year
period. A total of 133 patients were treated, ofwhom 44 (33%)
were tertiary referrals. Benign disease accounted for 61 patients
{eholelitliasis (n=25), chronic panereatitis (n=10), selerosing
eholangitis (n=8), other (n=l8)} whereas 72 had malignant disease
{pancreatic eareinorna (n=43), eholangioeareinorna (n 13),
periarnpullary eareinorna (n=9), other (n=7)}. The mean age of
those with benign disease was 58.2 years, and ofthose with
malignant disease 62.5 years. Operative procedures performed
were eholedoehoduodenostorny (n=47), eholeeystjejunostorny
(n=25), eholedoehojejunostorny (n=22), hepatieodoeho-
jejunostorny (n=25), hepatieodoehoduodenostorny (n=2),
Whipple's (n=10), and local excision (n=2). There was no
significant difference in hospit.al mortality between those with
benign o malignant disease (1.6% vs 8.3%). Early morbidity
occurred in 14.8% ofthose with benign disease, compared to
20.8% ofthose with a malignant condition. Late morbidity
occurred in 19.7% ofthose with benign disease, compared to
22.2% ofthose with a malignant condition. There was no
significant difference in mean postoperative stay between those
with benign or malignant disease (12.2 days vs 15.6 days). The
median survival for those with malignant disease was 8 months
(range 1-60 months). Biliary bypass procedures are suitable for
benign and malignant disease. There was no significant difference
in mortality, morbidity or length ofpostoperative stay between
these two groups ofpatients.
SURGICAL COMPLICATIONS AFTER ENDOSCOPIC PAPILLOTOMY-EPT
V. Peqan, M. Omejc, J. Vrako, V. Mlinari
University Medical Centre Ljublana, Departmenf of
Gastroenterologic Surgery,. Zaloka 7, LJUBLJANA, SLO
In a retrospective study we have analysed 30 patients
having surgical complications after EPT in the period
from 1982 t{11 the end 1995. The mean age of patients
was 61 year and there were two thirds female patients.
In a 4 years period we have surgicaly treated 0 cases
of choledochoduodena! perforations, 12 cases of conser-
vatively uncontrolable bleeding and 8 cases ith incar-
cerated or ,broken Dormia basket. In the postoperative
period 'we have lost 3 patients due to retroperitoneal
sepsis after choledochoduodenal perforation. All patients
with incisional haemorrhage survived after surgical
haemostasis. The incarcerated or broken Dormia baskets
were all succesfully removed and all patients recovered
uneventfuly. The diagnosis of perforation was based
either on discovered extravasation of the contrast media
or on the presence of gas bubbles in the surroundings of
the duodenum.
Conclusions: EPT is a relatively safe procedure if
perforned by an experienced endoscopist. Surgical
complications are rare and only in exceptional
circumstances (age, delayed diagnosis).fata1.
Conservative treatment ofcholedochoduodenal
perforation is very risky and the treatment of
choice should be an early operative revision
and reconstrucction.
177
P422 P423
RESIDUAL CALCULOSIS OF BK,E DUCTS
V. Pci6, M. Jeremi6, M. Stoiljkovi6, A. Nasomi, M. Stojanovi6
G. Stojanovi6, S. Jeremi6
Surgical and Gastroenterology Clint'eClinieal center of NiT
Despite the great progress in preoperative and postoperative biliari
diagnostics, residual ealeulosis of bile duets remains important problem in
In surgical clinic in Ni, period from 1985 to 1994 resistered 63
patients with residual calculosis (RC) of hepaticocholedochus filCH),
aler operations of the 8allblnder mad bile ducts. ARer cholecystectomy
(3325). registered 42 eases or 1.2% mad fter operations on HCH (240)
registered 21 eases or 8.75% Re.
The autors analyzed causes, methods of treatment and outcomes of RC.
They emphasized value of the preventive procedures (preOperative
diagnosis, intraopemtive cholarosraphy, cholangioscopy, and
ultrasonography, choise of operative procedure, surseons expirienec) for
decrease of incidence of this unagreable complication.
Residual-caleulosis treated by reoperations in 42 cases (66%) and
endoscopic papillotomy in 21 cases (33%). Reinterventions were in form
of choledochotomy, extraction of calculi and T drainage, biliodisestive
anastomosis or sphyncteroplasty. Endoscopic pappilotomy performed in
lfigh-risque patients, calculosis of distal parts of HCH and evidcnc of
papillary stenosis.
Specific complications were: leakage of biliodisestive .anastomosis,
t cholartis after biliodisestive anastomosis in one case and
tzmcreatitis after one sphynctemplasty. It was not mortality. RC remains
important problem in biliary sursery because of many problems in
prevency and treatment. Reotxations are difficult and require great
expirience in biliary surgery. Endoscopic papillotomy is metod of choise
tn selected cases.
H Petrovi, V. ArlRo, V, (adovi0 H Hllidevi and
I KostfC"
Ir, oP Digestive Di& CES, Sca'xl o FL, Beograd-YU
aim o tl study is tl assessrnt o tl
gallbl () ctile m 8 cls (Cs),
6 wi htc/atc (), 6 tis
wi l%is cosis (), eily
s% %is: 0 ely () iO
late () t tol gt II
II I (i) ti (BI).
st wi R c
I ct (SIO) ion I
i s, ctio (oP ay
i otivity ion .
y t () X:. +/- i.,), ejti
cti () X:7.TZ +/- 7.9) ejtion te ()
X=.X+/-O.& c, m y
ill i till stu.
cisto m Io
(7. +/- .5, p<0. Oi), I (X:7.Z +/- 6.9, p
O. Oi) (X=I. IZ/+/., p <O. Oi)
eoPs. In (X:aT. +/- 0.9)
le (X= aB. TX +/-
I0. 9) (X:L IX+/. 5) (p< O. 0).
, m (x=Bg. +/-aO.O), (X: 7.Z
+/-.) (X=i. BZ+/-O. 6) 't f (p>
0.). , m BI, (+/-& 5),
cli t tol t wi ot
clul it, t
tility ely o ioloical
ctile ti late t otion t
att to esli
(). tits wi ial
t, t tion
t, tility st
P424 P425
EFFECT OF COMBINATION THERAPY WITH IMMUNOSUPPRESSORS
AND UDCA IN THE TREATMENT OF PRIMARY BILIARY CIRRHOSIS.
A. Pezzoli, P. Fusaroli, G. Mazel!a, C. Fabbd, A. Cipolla, E Roda.
Cattedra di Gastroenterologia University of Bologna Bologna Italy.
Primary biliary cirrhosis (PBC) is a chronic cholestatic liver disease in which
intra-hepatic bile ducts are progressively destroyed. The disease is
associated with profound, but yet not fully characterized, immunological
disturbances. Presently, the most widespread drug used to treat this disease
is ursodeoxycholic acid (UDCA) which has been shown to ameliorate
symptoms and improve serum liver enzymes by increasing the
hydrophilic/hydrophobic ratio of the total bile acid pool. It has also been
suggested that immunosuppressive therapy may be useful in treatment of
this disease. Aim of this study was to evaluate the efficacy of joint therapy in
the treatment PBC using UDCA in combination with immunosuppressive
drugs.
Nine patients (8F:IM) with diagnosis of PBC (stage I-III) made according to
the usual biochemical, serological, clinical and histological criteria were
enrolled for the study. Each patient was treated randomly with 3 different
therapy combinations: 1) UDCA 900mg 2) Azathioprine (AZA) 50mg +
UDCA 900mg, 3) Methylprendisolone (MP) 8mg + UDCA 900mg. Each
treatment combination was administered for 4 weeks with a wash-out period
of 4 weeks in-between. Before and after each treatment period serum ALT,
AIPh, 7GT, bilirubin and IgM were tested. Statistical analysis was performed
using Fdedman's nonparametric test.
The combination UDCA with immunosuppressors was able to improve, with
respect to UDCA alone, serum markers of cholestasis and transaminase
levels as well as reducing IgM levels.
....m..............s.. ...v.. .s....r.. !.. ..r..
Basal 83 70 872 267
UDCA 55 48 717 165
AZA + UDCA 51 38* 535* 125
MP + UDCA 43 21" 436* 95
;.p<0.01 vs basal 0.01 0.01 0.01 0.01
p<0.05 vs UDCA;
In conclusion this study indicates a possible role for the use of
immunosuppressive therapy in the treatment of PBC in association with
UDCA.
BILIOENTEROSTOMY IN BENIGN BILE
DISTURBANCIF_,S
S. PoWc, M. Kozelj M. Horvat I Kuder F Grandovec
DepartementforAbdonnalSurgery,
TeachingHosptaIMARIBOR SLOVENIA
OUTFLOW
In a reuospecfive stady (Jan 1992 to Oct 1995) we =nelysed the outcome
of52 petienls (1)( 16 m, 36 f;avmq age 74 yeaea, raage 37-90 years
who underwent bilioenterostomy for a benign bile outflow problems. In
42 pts choledochoduodenmtomy and in 10 pts choledojejunostomy was
done. In 39 pts cholecystectomy was done as well, 13 pts have had
cholecystectomy done before. The median ASA value for the entire grup
was 3. The indication for smgery were: bile duct stones (28
obsmzcfion umsed by dmmic pmmreafitis (15 pts), stmmsis ofthe papilla
of Vater (5 pts) ill divelticule (2 pts) %vstic" bile duct
dilalation (1 pts), iatrosenic lesion ofbile duct(l pts). Jmmdiced were 39
pts and 13 were not. EPT was tried in 7 pts without success. Total
0) leve (mmo), eete phoOm=e (AP) end sem St
(GT) (nkat/l) levels were measured before the operation(l), on 7-10 th
poetopmive day (2) end oe in the ollow up(3). The avemse levels
were: TB =118 (14-668), AP 1=5.6 (0.7-18), GT 1=5.1 (O.4-26), TB
2=38 (7.110), AP 2=2.4 (0.8-6),GT 2=2.1 (0.2-9), TB 3=12 (4-22), AP
3=1.6 (1.1-2.6), GT 3=0.8 (0.2-1.7). Janndice diseppeared 5-18 days
after operatil"ne avmehospital ay was 10 days (8-20). The were
5 minor mmplications (7.3 %) end no one died. No pts have become
icteric or had cholmgitis in the follow up (2 months to 3.8 yeers).
We consider the surgical derivation of bile in above mmtioned
indications in old pts as easy, short, safe and efficimt method, with
acceptablerate ofmmbidity and mortality.
178
P426 P427
IATROGENIC LESIONS OF THE BILIARY TRACT: DIAGNOSTIC AND
THERAPEUTICAL OPTIONS
A. Prineipe, M.L. Lugaresi, I. Biechierri, M.C. Gallo, B. Nardo, G. Fuga, A.
Mazziotti, A. Cavallari
2 Department of Surgery-Policlinico S.Orsola-University ofBologna-Italy
Iatrogenie biliary lesions after Laparoscopie Choleeystectomy (LC) has an
increased incidence varying from 0.3 to 0.7% versus the low incidence of 0.1 to
0.2% seen with Open Choleeysteetomy (OC). The prognosis is strictly correlated
with the moment of discovery of the lesion which rarely reveals itself during the
operation. To prevent or reduce the incidence ofthe injuries we favour, in ease of
doubt, the use of a selective perioperative cholangiography or an ERCP ater the
operation. Aim of this study is to analyse the incidence and risk factors of these
bile duct injuries and to evaluate the management ofthese injuries.
Material and Methodes- Since 1982, 66 pts, divided in two groups, were
observed. Ofthe 17 pts comprising the first group (25%) the lesion was revealed
immediately or early, with the planed diagnostic work up; in 7 cases after
choleeystectomy, in 3 after hepatic resection and in 2 after gastric resection. The
treatment was: a biliary duct plasty in cases, an HepaticoJejunoStomy (I-IJS) at
the biliary confluence in 7, a CholedochoDuodenoStomy in 1, and in the last one
with a Smith derivation. In 49 pts (second group: 75%) the lesion was detected
later. Site, extension, and grade of the lesion were established with PTC and/or
ERCP.
Results- Three pts (17.6%) in the first group dead postoperatively due to
pulmonary embolism, myocardial infarct, and hepatic insufficiency, respectively.
The FU (1-14 yrs) showed good results in 12 pts (85.7%) and the death of the
Smith procedure after 2 years for secondary biliary cirrhosis. In the second group,
4 pts (8.1%) needed a new anastomosis; 2 because of abiliary fistula and 2 for a
stenosis occurred after year and 4 years from the first repair. The clinical FU
revealed a good outcome in 45 pts (91.8%). Two pts (4%) with biliary cirrhosis
died after 7 and years. Three pts present recurrent angiocholitis.
Conclusions- Selective perioperative cholangiography or postoperative ERCP are
fundamental to prevent or reduce iatrogenic injuries. Only an expert surgeon can
offer a good prognosis, which is most favorable in cases of immediate repair. HIS
must be performed at the biliary confluence and extended to the left hepatic duct,
so to avoid reinterventions and therefore a poor outcome ofthe patients.
CARCINOMA OF THE MIDDLE AND LOWER BILIARY TRACT: STAGING
AND INDICATIONS FOR CURATIVE RESECTIONS
A. Principe, M.L. Lugaresi, M.C. Gallo, I. Biechierri, E. Polito, R. Lords, G. Serra,
A. Cavallari
2 Department of Surgery- Polielinieo S. Orsola- University ofBologna- ITALY
Primitive carcinoma of the Middle and Lower biliary tract is a rare tumor
characterized by; late clinical presentation, very extensive diffusion and a severe
prognosis. Early diagnosis and radical excision with curative intent greatly
influence survival. The purpose of this study is to demonstrate that only surgical
exploration and perioperative studies are ables to provide a correct assessment of
reseetability. Material and Methods- From 1982, 21 pts with primitive carcinoma
ofthe Middle and Lower third ofthe Common Bile Duct (M=6 pts; L=15 lOtS) were
observed. The preoperative work up, carried out in all cases through US, CT and
PTC or ERCP, established the site of the tumor, staging and reseetability. One pt,
with tumor in the Lower third, due to severe cardiac compromise (ASA IV), was
not operated. At operation, exploration, intraoperative US (which provides greater
sensitivity than angiography), and frozen section of the resected margins modified
the preoperative staging in 10 pts (50%), 2 in the Middle third and 8 in the Lower
third. Reseetability was confirmed in 16 cases (80%: R.I. M 83.3%, R.I.
L=78.6%). Five pts with tumor of the Middle third (3 St.I, 2 St. II) were treated
with simple excision of the tumor. The only pt with diffuse metastasis (St. IVb)
was drained with a trans-tumoral T-tube. Eleven pts with tumor in the distal third
(4 St.II, St.III, St.IVa,1 St.IVb) were considered resectable and therefore
underwent Panereato-Duodenectomy(PD). The remaining 3 pts, who were
considered inoperables (2 for diffuse metastasis, and because of advanced age)
were treated with a palliative Bilio-Digestive Anastomosis. Results- Middle third:
the only pt with palliative surgery died in 2 days. Three pts (2 St.I, St. II) in spite
of favorable staging died from relapse at 13, 14, and 19 months (m.s. 15 mo); the
others 2 pts (1 St.I, St.II) are alive at 73 and 120 months (m.s.96 mo).-Lowr
third_" no operative mortality. Average srvival of the 3 pts treated with palliation
was 6 months; pts treated with PD died (m.s. 20 mo) while 3 pts (2 St.II,
1St.IVa) are alive at 29, 48, 156 months (m.s.77 mo). The only pt not operated
(ASA IV) survived months with an external biliary drain. Conclusions-
Definitive staging and correct indications for curative resection are only possible
using intraoperative studies following the preoperative diagnostic work up. To
evaluate the outcome only the definitive histologic examination is able to recognize
a tumor of the pancreatic head. Results suggest that with the increased surgical
aggressivity survival may be prolonged with good quality oflife, and that survival
obtained with surgical and non surgical palliative measures are comparable.
P428 P429
PRESENTATION AND DIAGNOSIS OF
DUODENAL DIVERTICULAE
Rashed MYT, ZEl-Sefi T
Alexandria University, HPB Unit, Medical Dept.
ZMenoufeiya Univ.,Liver Institute, Surg. Dept.
Most duodenal diverticulae(DD) are discovered
accidently at endoscopy. This study evaluates
the relationship between the site of the DD
and associated diseases and it's impact on
management. Out of 540 endoscopic examinations
DD were found in 26 patients. M to F ratio was
1:2.25 with a mean age of 56 years. Five had
anterior bulb diverticulae(BD)and 21 had
second part diverticulae(SPD).In 4 patients
with BD, chronic active ulcers were found. Out
of 21 with SPD 4 had only moderate biliary
dilatation and papillary dysfunction, ll had
choledocholithiasis(8 cholecystectomised)with
small choledocho-duodenal fistula in 2. The
remaining 6 patients had GB, and CBD stones
(3 presented with acute biliary pancreatitis)
The Vater's papilla with rudimentary intramu-
ral CBD(ICBD) was at the edge of the divert-
iculum in 6,at the floor with long dilated
ICBD in 7, in between two diverticulae with
bulging ICBD in 4, and paradiverticular in 4
patients. The management included endoscopic
sphinctrotomy with/without stone extraction
and choledochoduodenostomy. This study demon-
strates the association of DD with bulb ulcers,
biliary lithiasis,papillary dysfunction and
biliary pancreatitis.
CHOLEDOCHAL CYSTS IN ADULTS AND CHILDREN:
PRESENTATION, DIAGNOSIS AND MANAGEMENT
MYT Rashed, aT. Ei-Sefi, zI. Marwan, ZA.Helmy
Medical Dept.,HPB Unit,Alexandria University
ZSurgical Dept.,Liver Institute, Menoufeiya
University, Egypt.
Eighteen patients(4 children, 14 adults) with
choledochal cysts were studied. Fourteen were
F and 4 M, with a median age of 26 years(range:
.4 months-40 years). The clinical presentation
in 17 patients included abdominal pain and
recurrent attacks of cholangitis. The remaining
one(14 months) presented with cyst rupture.
Five patients had previous cholecystectomy. The
diagnosis was made in all patients by endosco-
pic retrograde pancreatography. According to
Todani classification, i0 (56%) patients had
type I cysts, 2 had typeII,2 had type III,2 2
had a type IVa cyst, one had type IVb, and one
had type V cyst. Associated diseases included
cystolithiasis in 16, cholelithiasis in 3,1iver
cirrhosis in 3, and chronic pancreatitis in one
The management in the children group included
cystectomy with Roux-en-Y hepaticojejunostomy
(HJ) except one who died of liver failure. In
adults:of the 7 type I cyst, cystectomy with HJ
was feasible in 4,while partial excision and
choldochocystojejunostomy was done in the
remaining 3.For type II,excision of the cyst
with closure of the choledochous over a T-tube
was done. For type III,endoscopic deroofing of
the choledochocoele was perfformed. For types
IVa, b, cystectomy and HJ was done. All patients
had resolution of their symptoms. Two patients
had late cholangitls.
179
P430 P431
Analysis of Iatrogenic Injury Biliary
Duct: Review of The China Literature
Zhang Ren
Deprtment of Surgery, The 2nd Central
Hospital, Tianjin, P. R. China
Ten patients were led iatrogenic injury
of extrahepatic bile duct between 1980 to
1995 at our hospital. We collected 617
cases of the China literature combine with
10 cases following analysis.
Morbidity: the sequence of original opera-
tion. (Number indicating patient No.)
cholecystectomy 463, gastrectomy 45 common
bile duct (exploration, lay up T
" tube
not proper, puncture 81, liver trauma
12, choledoch-cyst 5, abdominal tumor 4,
ERCP 3, gallbladder duet trauma 3, chemi-
cal lesion I.
Injury .Position and Type
common bile duct 184, common hepatic duct
145, combining site 146, right hepatic
duct 27, right and left hepatic ducts 8,
accessory hepatic 4, not clear 3.
Operation:Emergency 189, Elective 426.
Type: Transection and part trauma, Suture
ligation.
Cause Calot' s triangle I. Anatomic Vari-
ation. 2. Inflammtory adhesion ambiguousness
3. Bluring management of fundamental conce-
ption.
Operator: I. Blind stop bleeding and liga-
tion. 2. Despise eholecysteetomy and sober.
3. Pursue operative velocity. 4. Operation
opportunity selecting unsuitable.
5. Operation method selecting unsuitable.
CHOLEDOCHAL CYST DIAGNOSED WITH ENDOSCOPIC
RETROGRADE CHOLANGIOPANCREATOGRAPHY
II.IUBINI A.DEPOLO, M.PEII, M.URAVI(
Clinic for Internal medicine and Surgery, Institute for
pathology, Clinical Hospital center Reka, Croatia
The choledochal cyst is e rare development anomaly,
in the maor/ of cases congenita/, of the bJliary
tract. Hence this review of diagnosing this rare
disease in our material of ten years is made, to point
out the importance of endoscopic retrograde
cholangiopancreatography. The method is very
effective scanning the size, form, position and content
of the cyst, a fact of great Importance when planning
surgical treatment as the only possible cure. An
analysis is presented out of the resu/ts Of 2672
ERCP.s carried out during the past ten years. Only
three choledochal cysts, or 0,1 pro mille of the
exam/ned patients, has been diagnosed. Ai patients
were female of various age groups (8,19 and 36
years).
P432 P433
COMPLICATIONS OF LAPAROSCOPIC CHOLECYSTECTOMY WITH
SPECIFICATION OF DIAGNOSIS AND REPAIR OF IATROGENIC
-BILIARY DUCTS INJURY
S.Rudzki, J.Jesipowicz, J.Pilat, M.Jesipowicz
The First Department of General Surgery
Medical Academy in Lublin, Poland
Performing of laparoscoplc cholecystectc (l.ch.) was accompanied
by the inczeased nr of intraoperational ccmplicatlons, especia-
lly extrahepatic billary ducts injures. The aim of this study is to
present four year experience of the authors in performing l.ch. and
to point out the cclications which occured. In the years 1992
1995 there were 1200 l.ch. performS. Patients with acute inflanmm-
t.[on of gallblader and inflanstory changed surroundings as well as
patients who had earlier undergone an operation on their epigas
were not qualified to be operated on using l.ch.. In 4 cases there
were postoperative complications which required another operation:
in one case there was a classical iatrogenic injury of.the
bile duct, diagnosed on the third day after the l.ch., repaired by
hepatic-jejunoanastcmlosis,
in one case injury at one point of the omTmon bile duct wall took
place undoubtedly performed by electrocautery,
in two cases 3 months after l.ch. inflanTatory infiltration
proved to be probably the result of burning with diathermy.
In one case transection of the conmlon bile duct occured diagnosed
intraoperatively. It required conversion and repair operation. End
to end anastomosis splinted by T tube was performed. In one patient
during the operation right pnemlothorax took place, and in one case
puncture of the aorta with Veress needle occured (with no conse-
quences). L.ch. requires frc, a surgeon both exellent knowledge
of anatc and its variations of hepatoduodenal ligament as well as
being especially critical while operating. In our opinion l.ch. is
the method of choice in the treatment of gallbleder stones and it
does not have to be acccxnpanied by iatrogenic bile ducts injury
hich constituted 0,1o in our Clinic.
BREATH-HOLD MR CHOLANGIOIANCREATOGRAPHY
(MRCP) WITH FAST ADVANCED SPIN ECHO TECHNIQUE
(FASE) IN THE DIAGNOSIS OF MALIGNANT
OBSTRUCTION OF THE LOWER BILIARY TRACT
Jinkan Sai. Joe Ariyama, Masafumi Suyama, Kazuhiro Sato, Yoshihiro
Kubokawa', Masahiro Irimoto, Hitoshi Katayama
Department of Gastroenterology and Radiology, untendo University,
Tokyo, apan
OBJECTIVE To determine whether MRCP can provide useful
images in distal bile duct obstruction.
SUBJECTS AND METHODS MRCP was performed in 55 patients
with suspected pancreatobiliary diseases. In 14 patients lower biliary
tract obstruction was confirmed by direct cholangiography. These
included 8 pancreatic carcinomas, 2 bile duct carcinomas, carcinoma
of the papilla of Vater and 3 chronic pancreatitis. MRCP was performed
with Toshiba 1.5T System VISART using QD Spine Coil. The sequence
parameters were TR/TE=o/250ms, one shot, echo train length of 202,
slice thickness=40mm, matrix=384X 384, FOV=35 x 35cm. Frequency
selected fat saturation pulses were employed. Acquisition time was 3
seconds.
R ES U LT Excellent images of pancreatobiliary tract were obtained in all
patients by MRCP. Pancreatic carcinomas appeared as main pancreatic and
common bile duct strictures. In bile duct carcinomas localization and
patterns of bile duct obstruction were clearly visualized. In carcinoma of
papilla of Vater filling defect was observed both in the distal common bile
duct and main pancreatic duct. In chronic pancreatits smooth tapering
stenosis of the common bile duct was depicted. In patients with
unresectable malignancy, endoscopic biliary dranage was performed and
MRCP was useful to evaluate patency of the stent.
CONCLUSION MRCP is a noninvasive technique with excellent
accuracy in the diagnosis of bile duct obstruction and its causes.
180
P434 P435
BILIARY IMAGING BY SPIRAL CT CHOLANGIOGRAPHY
Z SAJAD, J OXTOBY, D.J WEST, M.DEAKIN
Departments of Radiology and Surgery, North Staffordshire
Hospital, Stoke on Trent, UK
Following intravenous injection of biliary contrast, Spiral
Acquisition Computerised Tomography allows the acquisition of
volumetric data enabling three dimensional reconstruction
and multi-angle projections of the biliary tree. Scans were
acquired using a Picker PQ2000. Oral biliary contrast (Sodium
Ipodate 3g) was given 14 hours and 3 hours before scanning to
fill the gallbladder. 150mls of Meglumine Iotroxate (50mg/ml)
[Biliscopin, Schering AG, Germany] was infused intravenously
over 60 minutes before scanning followed by 20mg of
Buscopan. An initial spiral scan was performed through the
upper abdomen to Iocalise the biliary tree using a collimated
slice thickness of 10mm with a pitch of 1, allowing a coverage
of 21cms. A second scan was then performed with a
collimated slice thickness 2-3 mm with a pitch of tol.5 during
a single breath hold. The axial images were indexed at lmm.
The data collected were used to reconstruct the biliary tree
using surface shading (3D) and multi-planer reconstruction
methods.
Scans were performed in 30 patients, 7 of whom had previously
undergone failed ERCP. Satisfactory scans showing complete
biliary anatomy including cystic duct insertion and anatomical
variations of hepatic ducts were obtained in 29 patients (97%).
Pathology was demonstrated in 4 patients common bile duct
stones (2), benign common bile duct stricture (1) and papilliary
stenosis in a patient with scleroderma. (1). All patients with both
abnormal and normal scans have had the findings confirmed by
alternative means or by uneventful clinical follow-up. All
patients tolerated the procedure well. SCTC is an attractive
non-invasive method of biliary imaging producing high definition
pictures of the biliary tree.
P436
LONG TERM EFFECTS OF LOST INTRAPERITONEAL
GALLSTONES
SARA A.M CiNG| A.- AKTAN A.O-YE(EN C.- YALIN R.
Marmara Uhv. School ofMedicine Dept. ofGeneral Surgery
istanbul -TURKEY
As laparoscopic cholecystectomy LC has gained wide
spread popularity, different types of complications are
encountered. One of them is the spillage of gallstones into the
abdominal cavity during surgery.
An animal model has been used to investigate the long and
the short term effects ofthis complication. Gallstones taken from
20 LC patients were placed {nto the abdominal cavity of 20 rats.
The stones were analyzed for biochemical nature and cultures
were obtained. The rats were kept alive in two groups for 3
and 6 month periods. Nine patients (45%) had pure cholesterol
stones and 4 patiens (20%) had bilirubin stones and 7 had (35%)
mixed stones. Cultures obtained resulted negative. At the end of
the study during laparotomies it was observed that the stones
were sealed with omentum. Any gross pathology, abscess
formation was not seen except in a rat, in the group 6 month,
which had a necrotic omentum and abdominal wall sinus
formation. Pathological studies revealed different degrees of
lymphocytic response between the groups. Inflammatory cell
response was seen in lesser degree in the cholesterol groups and
mesothelial cell proliferation was seen as a late response As a
result, lost intraperitoneal gallstones did not cause harmfull
sequela in the long term period and there was no gross
histopathological difference between the 3 and 6 months groups.
Due to these results it is concluded that agressive manipulations
for the spilled gallstones should be avoided during LC.
181
CHOLELITHIASIS FOLLOWING GASTRECTOMY OR
COLEL-TOMY FOR CANCER
N. SANDO, Y. SETSU
Department of Surgery, Mitosaiseikai General Hospital, Mito,
Japan.
PURPOSE: The aim of this study was to clarify clinical f'mdings
of cholelithiasis following gastrectomy or colectomy for cancer
of the stomach or the colon. METHODS: We examined 235
cases with gastrectomy and 151 cases with colectomy for cancer.
Gastrectomized cases were composed of 160 cases of subtotal
gastrectomy (SG), 66 cases of total gastrectomy (TG), 9 cases
of other types of gastrectomy (OG). Colectomized cases were
composed of 26 cases of right hemicolect0my (RH), 57 cases of
transverse colectomy or left hemicolectomy or sigmoidectomy
(TLS), 42 cases of proctosigmoidectomy (PS) and 26 cases of
abdominoperineal excision of rectum (AP). Cholelithiasis was
.diagnosed when the findings of stones or sludge were detected
in the gallbladder or bile ducts on ultra sonograms or
computerized tomograms. RESULTS: Postoperative
gallstones or sludge were detected in 25 cases (10.7%) of
gastrectomized cases, and in 12 cases (7.9%) of colectomized
cases. The incidence of cholelithiasis was significantly
(P<0.01) higher in cases of TG than in cases of SG, and
significantly (P<0.01) higher in cases of Roux-en -Y
anastomosis than in cases of Billroth-I anastomosis. The
incidence of cholelithiasis in cases of AP was higher than in
cases of other colectomies, but the difference not statistically
significant (P>0.05). Biliary surgery were performed in 6
gastrectomized cases with cholelithiasis and in no colectomized
case with it. CONCLUSIONS: Cholelithiasis occurs in
about 10% of cases with gastrectomy and in about 8% of cases
with colectomy for cancer, and appears to be the major
complication after TG and Roux-en -Y anastomosis. However
cholelithiasis following colectomy may be not the major
complication.
P437
BILOMA & BILIARY FISTULA POST-HEPATORRAPHY
FOR LIVER TRAUMA
MD Shahrudin & SM Noori
Department of Surgery, Faculty of Medicine
University of Malaya, 59100 Kuala Lumpur,MAL.
During 1986-1994, 6250 patients were admitted
to the A&E Unit of the University Hospital KL0
with 175 patients requiring hepatorraphy, ii
patients developed either a biloma, biliary
fistula or both.. Patients' ages ranged from
15-40 years with a mean ISS of 23. 7 patients
suffered penetrating injury & 4 were victims
of blunt trauma. The right lobe was injured in
i0 patients, with 1 patient sustaining left
lobe injury. All liver injuries were either
grade 3(7 patients) or grade 4(4 patients). No
patient sustained extrahepatic biliary tract
injury. Bilomas & fistulas were diagnosed 14-
30 days post-injury(mean 24 days) by CT and
HIDA scans. All were managed by CT-guided per-
cutaneous drainage. 1 patient also required
percutaneous transhepatic cholangiography with
biliary stent placement due to bile-stained
ascites. Fistulas persisted from 5-120 days
(mean 44 days). No patient required further
operative intervention and all fistulas closed
spontaneously without complication.
P438 P439
SELECTIVE ERCP AND DOSCOPIC PAPILLOTOHY (EPT) IN
ACUTE LITHIASIC PANCREATITIS (ALP)
.A.Secch, W.Sanchi, E. Tagllaferri, S.M.Krupik and G.
Ralmundo. Surgical Division. Hospital Itallano and Ho
pltal provincial. Rosario. Argentina.
ALP may be accompanied by papillary obstruction (PO) in
most cases (67g), and it is spontaneusly resolved within
the first 48 hs.(Br.J.Surg.79:Suppl S120,1992). Persis-
tent PO (PPO)is present in a minimum percentage (5-10g).
There are the only ones that should be treated endoscop
tally or surgically in a relatively early way (48-72hs).
METHODS: From 1987 to 1995, patients -197) having ALP
have been studied and treated prospectively. At admison
patients were divided into 2 groups: Group j:mild and
moderate cases (n=179) and Group 2: severe cases (n=18).
Group 1: patients underwent medical treatment for 6+I
days and then a complete and definite billary surgery
was performed (89Z). EPT prior to surgery was done only
when evidence of PPO (n=13), and as the only treatment in
3 cases. Group 2: in patients admitted at Intensive Care
Unit, slstemlc and local complications were treated. EFT
was performed in cases of PPO (n=5) and as the only
treatment in case. Definite billary surgery performed
in 15 patients (84Z) with a mean of 9.9+5.1 days after
admission. RESULTS: Group I: the mean hospitalization
time was 8+_2 days. Morbility was 8.9 and mortality was
null. Group 2: the mean hospitalization time was 24+15
days. Morbillty was 38Z and mortallty was 16g. There was
no direct correlation between PO and mortality. We think
that performing an early EPT, without a justified PO
does no modify the course of ALP, but may complicate the
illness. Selective ERCP and EPT were appropriated for
the treatment of ALP.
INTERACTION OF OCTAPEPTIDE Or: CHOLECYSTOKININ, VASOACTIVE INTESTINAL
PEPTIDE AND SUBSTANCE P ON DYNAMICS OF BILIARY SYSTEM AND
CARDIOVASCULAR SYSTEM
ZOo Shengquan. Oiu Fazu. Zhang Jlsnfeng.
Depaimnt of surgery, TongLJl Hospital, TongJi Medical University,
Wuhan. Chino 430030.
AIM:
This study designed to determine the effects of the octapeptide, of Cholecystoklnln
[CCK-OF.vasonctive Intestinal peptide rTIPj and Substancs P [SPJ dynamics of biliory
system .and cardiovascular system.
GROUPS:
(1) CCK-OP Infused at 10 no/kg/min |n:13)o (2| CCK-OP infused at 20 ng/klgmin
|n=15|, |3J VIP Infused at 50 ng/ko/min (n:14J, (4] VIP at 50 ng/kg/mln csnJlonlng with
CCK-OP at 20 ng/kgnln infused |n=11)o [5) SP infused at 100 ng/kg/mln |n=15), (6)
SP infused at 200 ng/kgmin (n:12).
METHODS:
A pressure-monitored perfusion cather Inserted into left ventricle Left ventricle
stroke pressure (LVSI and dl)/dt obtained. A catheter passed into the
bile duct and the Sphincter of Oddl |OS) through duodenum, motility of OS and
pressure in the bile duct recorded during 30 minutes perfuslon.
RESULI$:
Both CCK-OP snd SP slgnlficsntiy atlmulsted dyamlcs of blllary system Incresslng
pressure In bile duct (p(O.05),lnceaslng phasic frequency and motility
Index of O$ (p(0.05), CCK-OP and SP significantly Inhibited dynamics of cardiovascular
eystem0lecreaslng LVSP and dp/dt Lo(0.05)o with CCK-OP being potent. VIP
alone :viewed little effect dee,'easing pressure In bile duct and
significant effect 05 and cardiovascular system. In conjunction with CCK-OP, VIP
produced Inhltlon the effect of CCK-OP blllory system,but not cardiovascular system.
CONCLJSION:
These studies indicate 1hat may be Important ntersction between dynamics of
blllary system and cardiovascular system. We conclude that gastrointestinal peptides
may play great roles in the Interaction between blllary system and cardiovascular system.
P440 P441
SURGICAL MANAGEMENT OF CARCINOMAS OF THE BILE DUCTS
(PERIAMPULLARY TUMORS INCLUDED)
Kanac Shinbara '), Takashi Kodama'), Yoshio Takcsuc', Hiroaki Tsumura
Naokuni Tatsumoto ', Takas Yokoyama 2), Yuicdrou Matsuura ').
First Department of Surgery and General Me&tint2, Hiroshima University
School of Medicine Hirosma Japan
Rcsectability rates and long-term survival rates of patients with carcinomas
of the bile ducts remain low because of the difficulty of early diagnosis and
the proximity of these tumors to vital structures. Until recently palliative
pcrcutancous, endoscopic, or surgical biliary drainage was considered to be
acceptable treatment in terms of operative death and long-term survival when
compared with more aggressive methods. Then we retrospectively analyzed
the outcome of 51 patients who underwent surgical treatments for primary
carcinoma of the bile ducts (periampullary tumors included) in our institute
between 1981 and 1995. The diagnosis was confirmed by reexamination of
preserved,tissue specimens. The 11 female and 40 male patients (mean age,
65 years) underwent follow-up for a maximum of 14 years (mean, 2.7 years).
Resectability rates were 68% for extrahepatic bile duct and 79% for
periampulla, respectively. The 5-year mortality rate in resectable cases was
significantly higher compared with in palliative cases (55% vs 0%
p<0.05).The curability rate in cases of periampullary, lower- and middle-third
tumors was higher than that of the upper-third tumors (79% or 73% vs 46%).
According to the TMN classification, the curability rate in cases of stage
was higher than others. The judgment of resectability of tumors by
preoperative evaluation was so difficult that we decided it at operation in
many cases. Thus we usually performe operations except for the cases with
definite invasion to large vessels. Recently, in resectable cases of the upper-
third cholangioma, we try to performe cholangioplasty using stents, and/or
post operative intraluminal radiation to residual cancer for QOL of patients.
In conclusion, resection of tumors makes worthwhile long survival in cases
of the bile duct cancers.
MANAGEMENT OF ASSOCIATED BIIJ DUCT STONES IN
THE ERA OF LC. O.Sutis,_M._D., A.Bubnys, M.D. Clinic of
Abdominal Surgery, Vilnius University Santarisldu Hospital,
Lithuania.
The advent of laparoscopic cholecystectomy (LC) has challenged the
management of associated gallbladder (GB) and common bile duet
(CBD) stones used before. A combined endoscopie-laparoscopic
(two-stage) approach was approved. The aim of this report is to
evaluate our experience with this approach.
We prospectively collected data on 1473 patients (pts) who
underwent cholecystectomy from December 1992 to June 1995.
Selective endoscopic retrograde cholangiographies (ERCH) were
employed with a 91% success rate in 136 pts with "high" risk for
CBD stones preoperatively and seventy.five pts were found to have
CBD stones. A significant correlation was observed between
suspected stones by ultrasound and stones found by ERCH
(p<0,01). Endoscopic choledocholithotomy was successful in 60 pts.
Laparoscopic or conventional intraoperative cholangiographies were
performed for 12 pts in which ERCH was unsuccessful. Open
surgery was considered as the second option for 15 pts in which
ERCH clearance failed. In 76% of pts with simultaneous
eholeeystolithiasis and choledoeholithiasis two-stage approach was
used successfully, with a complication rate 6,6% and a mean hospital
stay of 8,2 days.
Long-term follow ups (mean follow-up 12,4 months) were performed
in all pts managed by two-stage approach. No recurrent biliary
disease was observed. In group with qo risk for CBD stones
retained CBD stones were revealed during LC and postoperatively
in one and 4 pts respectively. Stones were successfully removed with
ERCH.
The results obtained suggest that a two-stage approach is clinically
effective for pts with simultaneous cholecystolithiasis and
choledocholithiasis although it requires adequate selection ofpts but
not requires additional expenses.
182
P442 P443
BILIARY BIOPSY USING A PULLBACK ATHERECTOMY
CATHETER
J SINGANAYAGAM, J W OXTOBY, J MCCAIG, P TIWARI,
M DEAKIN, D J WEST
Departments of Radiology and Surgery, North Staffordshire
Hospital, Stoke on Trent, UK
Transluminal or percutaneous cytology from biliary strictures
can be obtained although false negative rates are high. Guided
core biopsies can be difficult to obtain but are helpful not only in
the diagnosis of malignancy but also for obtaining specific
histology in the case of benign strictures.
The Pullback Atherectomy Catheter is a wire guided device
designed for the removal of arteriosclerotic plaques in
peripheral vascular disease. We have used.this catheter for
obtaining biopsies in the biliary tree in patients with biliary
strictures. Eight patients with obstructive jaundice underwent
percutaneous trans-hepatic cholangiography following failure to
cross strictures from below at ERCP. After passage of a guide-
wire across the stricture, biopsies at the strictured site were
obtained. Satisfactory cores of up to lcm x 2mm were obtained
in all patients. Seven patients underwent stenting. His(ological
diagnoses were adenocarcinoma (4), malignancy of uncertain
cell type (1), inflammation (1) fibrosclerosis (1) and normal
epithelium (1). In the patient where the biopsy yielded normal
epithelium subsequent trucut biopsy at laparoscopy proved a
Clinical diagnosis of pancreatic carcinoma. Biopsies taken the
pullback atherectomy catheter gave a definitive diagnosis in 7
patients with a sensitivity of 87%. The pullback atherectomy
catheter is a useful device for obtaining biopsies of the biliary
tree when conventional techniques have failed or where core
tissue of strictures is required.
EXTRAHEPATIC BiLE DUCT AND
GALLBLADDER CARCINOMA
E.M. S6ziier, N. Akyiirek, M. Akplnar, Z. Ydmaz
Department of Surgery, University ofErciyes, Kayseri, Tiirkiye
Primary adenocarcinoma of the extrahepatic bile duct (EHBD) is an un-
common malignant tumor leading to progressive biliary obstruction, sep-
sis, secondary biliary cirrhosis and hepatic failure. The incidance of gall-
bladder carcinoma (GC) varies in different parts of the world. Early diag-
nosis.of gallbladder and EHBD carcinoma are rarcly achieved because of
a lack of spesific signs and symptoms.
The data concerning 27 patients operatedon for EHBD carcinomas and
31 for GC were collected retrospectively. This study did not include pati-
ents with carcinoma of the ampulla vatcr or intrahepatic cholangiocarci-
noma 42 patients were men (72%) and 16 women (28%) mean age
60.1 years.
EHBD carcinomas were classified an upper -middle or lower-third tu-
mors according to the American Joint Committee on cancer. Distrubition
of EHBD carcinomas: 12 patients had. upper-third, 4 middle-third, 3 lo-
wer-third and 8 diffuse. Also GC were divided according to Nevin classi-
ficaUon There were 13 patients in stage 5, 8 in stage 4, 8 in stage 3,1
in stage 2 and in stage 1.
In the diagnosis ofEHBD carcinomas, US were performed in 27, PTC in
19 and CT in 6 patients. The most common diagnostic procedures were
US and CT in the diagnosis ofthe GC.
The curative resectability rate was 0% and palliative resectability rate
was 75 % in the patients with EHBD carcinoma. Curative resections were
done 22.5% ofthe patients with GC. The mean survey was approximately
9 monthsin the GC and 6 months in EHBD carcinoma.
As a result of this study; the prognosis is poor in EHBD carcinoma and
GC. The most effective factors on the prognosis are early diagnosis and
extended curative resections.
P444 P445
LAPAROSCOPIC CHOLECYSTECTOMY ANALYSIS OF
INTRA AND POSTOPERATIVE COMPLICATIONS
V.Spasov, V.Dimov, T.Josifovski, V.Avramovski
Clinic for Digestive Surgery, Medical Faculty, University "St. Ciril and
Metodi", Skopje, Republic ofMacedonia
Laparoscop.ic cholecystectomy (LH) has been performed in 286
selected patients with symptomatic gall bladder calculosis. This
represent one fifth of all cholecystectomies operated in our clinic from
October 92. In 33(11,5%) cases LH was performed in complicated
cholecystitis. More frequently intraoperative complications in
36(12,5%) cases were bile leak due to accidental perforation ofwall of
gall bladder. Spillage of stones occurred in 9(3,1%) cases, and in one
case had been reason for conversion. Bleeding from cystic artery
happened in 6(2%) cases. Enlargement of umbilical incision had been
necessary in 17(6,3%) cases because the difficult extraction of gall
bladder. One case (0,34%) of transection of common bile duct was
recognized on operation and "end to end" suture over T tube was
created after conversion. A case with suture of small bowel to the
umbilical incision was noticed on the end of operation. Only one
patient had to be reoperated after LH due to biliary peritonitis from
incompletely occluded cystic duct. Two cases of postoperative bile
collection were recognized (confirmed by HIDA) and successfully
treated with punctions and drainage. Two retained common bile
stones (0.74%) were evacuated endoscopiealy after papillotomy. Five
(1,8%) infections of umbilical wound after operation needed
management early removing of skin clamps. Two postoperative
hernias (0.74%) occurred in late postoperative period. Conversion rate
was 19(6,6%).No mortality occurred in our serie. Usually we
discharge patients after successfully LH on second postoperative day.
Our modest experience with LH confirm .advantages of this operation,
but in the same time warn for many pitfalls in the path of successful
intra and postoperative management ofthe patients.
ESTROGEN RECEPTORS IN GALLBLADDER CARCINOMA
I.Stamenkovid,V.Kati6,A.Nagorni,M.Jeremi6
Medical FacUlty,University of Ni,Yugoslavia
Malignant tumors of the Gallbladder are in
increasing. The percentage of signet ring cell
carcinoma is not frequent,but it has the spe-
cial features which are not known well.Becau-
se thatwe study them:
Cancer tissues of 3 patients with signet ring
cell carcinoma were fixed with 10% neutral
formalin. The paraffin sections were .stained
with HE,PAS,HID-AB and PAP. (using the primary
antibody of estrogen receptor-ER).
The patients are only women,between 30 and 40
-year old,with predominantely sialomucins
with diffuse infiltration and with poorer out-
comes.The most important finding is the demo-
nstration of R in the nucleus of the cancer
cells.
The authors discuss estrogen depender prolife
ration in cases with signet ring cell carcino
ma as well as the possible endocrine therapy
of these patients in the near future.
183
P446 P447
COMPLICATIONS OH LAPAROSCOPIC CHOLECYSTECTOMY
E.Stanowski,A.aczyrski,T.Koziarski
I Surgical Clinic of Central Military Hospital
Warsaw Poland.
From 1991 to 1995, 2?O0patients have been qali-
fled to laparoscopic cholecystectomy/LCH/.
1712 female and 988 male from 12 to85 years
of age.Among 2700 patients have been found 1080
cases of acute cholecystitis,and 1610 chronic
cholecystitis,10 cases carcinoma of gallbladder,
Among 1080 cases of acute cholecystitis have
been found 425 cases of empyema gallbladder and
657 cases of hydroma gallbladder.15 of our
patients required creationof C09pneumoperito-
neum so"open"way due to peritonel adhesions
having got different abdominal operations pre-
viously. In the rest of our patients the CO
pneumoperitoneum have been typicaly with Vres
needle.8 of our patients CO2 pneumoperitoneum
have been done to the greater omentum,68 ofLCH
have been changed on classical cholecystectomy
due to anatomical changes and identyfication
difficulties and /or great inflamation changes
well of gallbladder or in the duodenohepatic
ligament.7 patients common bile duct have been
injured,55 patients of gallbladder wll have
been injured with bile discharge and calculi
have been lost.Those patients required washing
out and sucking out the bile and the calculi
wos pickingup to the special container.YOour
patients required draining of peritoneal cavity.
We have had 45 cases of bleeding frrm abdominal
wII,55 cases of bleeding from place gallbladder
resected.2 cases of dudenum lesion.Open chole-
cystectomy have been performed 14 time in those
cases.Bleeding after abdominal troacar puncture
wos provide maitres suturae made under camera
control or Foley catheter hemostatic compresion
IMMUNOHISTOCHEMICAL DETECTION OF MIB-I IN
CARCINOMA OF THE EXTRAHEPATIC BILE DUCT
T.SUto, H.Nitta, M.Murakami, Y.Hayakawa,
H.Kawamura, Y.Shimada, R.Sasaki S.Kanno* and
K.Saito
First Department of Surgery, Iwate Medical
University, Morioka and Department of
Surgery, Iwate prefectural Kuji Hospital*,
Kuji, Japan
We investigated the value of
immunohistochemical detection of MIB-1 as a
prognostic marker in carcinoma of the
extrahepatic bile duct (EHBD).
MATERIALS AND METHODS: Surgical specimens
from 47 patients with EHBD cancer were
immunostained with MIB-1 (IMMUNOTEH S.A.)
against Ki-67 nuclear antigen, using the
avidin-biotin peroxidase complex technique.
RESULTS: 1)Survival rate of the patients
with low MIB-1 labeling index (LI<32%) has
been better than the patients with high MIB-
1LI (LI>32) (p<0.05). 2)MIB-I LI was
significantly higher when lymph, node
metastasis was positive (43.7+/-23.9%) than
negative (25.1+/-16.8). 3)No significant
differences were observed according to the
TNM staging, histologic type, lymphatic
invasion or venous invasion.
CONCLUSION: The results indicate that MIB-1
might be a prognostic indicator in patients
with EHBD carcinomas.
P448 P449
SURGICAL TREATMENT OF CANCERS OF THE EXTRAHEPATIC
BILIARY TRACT IN THE ELDERLY
T. Takahashi, N. Morita, Y. Kobayashi, D. Hayashi, T.Inokuchi, K.
Okamura, H. Shinagawa, T. Enoki, S. Noshima, K.Esato
First Deprtment of Surgery, Yamaguchi Univ., Ube, Japan
Cancers of the extrahepatic biliary tract are rare, but they pose great
problems from the diagnostic and therapeutic points of view. Surgical
resection offers the only prospect of cure for patients with this type of
cancer. The purpose of this study is to clear the optimal surgical treatment
of cancers of the extrahepatic biliary tract in the elderly. From January
1975 to December 1993, 32 surgical resections out of 48 patients were
performed. 16 patients were excluded for surgery. We review a series of 7
surgical resection of cancers of the extrahepatic biliary tract performed in
patients 75 or older than 75 years of age (aged group). A retrospective
comparison was conducted with a group of 25 surgical resection of those
performed in patients younger than 75 years of age (young group) during
the same time period. The elderly patients were ranged from 75 to 86-year-
old (mean age, 79.0 years). In the aged group there were 5 tumours in the
upper common bile duct and 2 tumours in the lower, and 13 in the upper
and 12 in the lower in the young group. 5 resections of bile duct
only(including hepatic duct) and 2 pancreatoduodenectomy were
undergone in the aged group, whereas 12 resections of only bile duct, 2
resections of bile duct with partial hepatectomy and 11 pancreato-
duodenectomy were undergone in the young group. Perioperative factors
affecting survival (operation time, blood loss in operation and hospital stay)
were compared between two groups, but there were no significant
differences. The overall operative mortality was none in this series. The
operative morbidity was 14% (1/7) in the aged group and 52% (13/25) in
the young group. In the aged group, the operative morbidity was
significantly lower than that in the young group (P < 0.05). Three and five
year survival rates were 51.4, 51.4% in the aged group and 26.1, 11.2 % in
the young group, without significant differences. Itwas concluded that age
is not a contraindication to surgical resection of cancers of the extrahepatic
biliary tract which offers the only hope for long-term survival.
ENDOSCOPIC BILIARY ENDOPROSTHESIS FOR
UNEXTRACTABLE COMMON BILE DUCT STONES.
L. Topa, Z. Berger, A. Pap; 2nd Dept. Med., St. hnre Hospital,
Budapest.
Endoscopic sphincterotomy fot removal of stones from the
common bile duct is an established procedure. Large stones,
however, can be unavailable for basket trapping and/or
extraction in some cases. In this patients which are at high risk
of surgery, endoscopic insertion of biliary endoprosthesis seems
to be an alternative approach to dissolution therapy or ESWL.
During the last 5 year, among 2143 ERCP-s 505 examinations
demonstrated common bile duct CBD/ stones in our
institution. In 51 of these cases, an endoprosthesis was inserted
into the CBD after extended .endoscopic sphincterotomy
because of failure of extraction of the large stones. Also
ursodeoxycholic-acid treatment was initiated thereafter. Mean
age of patient was 76 yrs range 49-92 yrs/, 33 females and 18
males. Acute complications after procedure were mild bleeding
not requiring transfusion, anfl perforation treated surgically
some days after prosthesis placement. Late complications until
now included: peritonitis in case, and recurrent jaundice due to
drain clogging in 6 patient. These patient were treated with
replacement of endoprosthesis. Remaining patient are well since
the procedure and in 11 cases controlled, about 6 months after
endoprosthesis placement endoscopy verified significantly
smaller or no stones in the common bile duct and in 3 cases even
the endoprosthesis has disappeared. Conclusion: endoscopic
insertion of a biliary endoprosthesis is a safe and effective
treatment for the huge CBD stones in high risk patients in whom
endoscopic sphincterotomy and attempts to remove the stones
are not succesful. In more than 20 % of cases dissolution of
stones with ursodeoxycholic acid may be expected.
184
P450 P451
THE DILEMMA OF HOW TO DEAL WITH A GALLBLADDER CARCINOMA
1N LAPAROSCOPIC SURGERY
A. Tuchmann, K. Pinnisch, P. Razek, R. Schmiederer
Dept. of Surgery, Hospital Floridsdorf, Vienna/Austria
INTRODUCTION: If a gallbladder carcinoma (g.c.) is re-
moved by laparoscopy, there could be the danger of car-
cinoma oell spread and metatasizing. The present retro-
spective study ought to be of help in decision-making
if the diagnosis of a g.c. is made after a laparoscopic
cholecystectomy (.l.che.).
PATIENTS AND METHODS: Between 1992 and 1995 six pat.
suffering from g.c. were operated by laparoscopy. The
tumor diagnosis was not made prior to the intervention.
During the same period 1288 1.che's were performed.
The incidence of g.c. was 0.47% (6/1288)..The tumor
stages were pTI n=5, pT2 n=1, pT5 n=1, pT# n=1. In four
patients the operation was terminated by laparoscopy.
In two cases we had to revert to open che.!The post-
operative development was uneventful in all cases.
RESULTS: %wo cases no treatment both alive and no
recurrence. Two cases required an additional resection
and lymphadenectomy of the hepatoduodenal ligament
both alive and no recurrence. One case no treatment,
metastases at the port sites after six months, death
at eight months. One case was liver metastases, chemo-
therapy, alive two months after surgery.
DISCUSSION AND CONCLUSION: The diagnosis of a g.c.
should be verified before surgery so as to I. avoid
tumor trauma by laparoscopy, 2. subject the patient to
open surgery. If che. is carried out by laparoscopy
despite the incidence of a g.c., a second surgical
intervention ought to be performed which should in-
clude an additional liver tissue resection as well as
a hepatoduodenal lymphadenectomy. Resection of the
port sites is also required.
ACUTE CHOLECYSTIrIS: DZRECT DI3SOLU?ICS C?
GALLSTONES WITH ORGANIC DISSOLVENTS AS AN
ALTERNATIVE THERAPY.
J.L Velo, R. Alvarez, M. Trinchet, J. Robles,
E. Gell, E. Cos.
H. G. U. Gregorio Marafion, Department of
Gastroenterology, Madrid, Spain.
Surgical treatment is the most widely
therapeutic aproach used in acute
cholecystitis; however on high risk patients
it should be interesting to performance
alternative therapies to improve the
prognosis.
METHODS: Ten patients aged between 70 and 80
years old with acute cholecystitis were
estudied. In all patients, a percutaneous
transhepatic drainage during 5-8 days was
used. After resolution of acute inflamation,
direct contact disolution with methyl tert-
butyl ether (MTBE) was performed. Results and
complications were compared with 20 patients
with non complicated gallbladder stones
treated with MTBE.
RESULTS: I.- All ten patients were
asymtomatic in a period of 5 to 8 days after
cholecystostomy. 2.- Gallbladder stones were
dissoluted in 100% of the cases. 3.-
Complications of the procedure were similar
in both groups.
CONCLUSIONS: Cholecystostomy and direct
dissolution with organic disolvents is an
excellent therapy in patients with acute
cholecystitis and with increased surgical
risk.
P452 P453
LAPAROSCOPIC CHOLECYSTECTOMY IN ACUTE
CHOLECYSTITIS
A.Viiklepp, J.Troost, H.Poola
Department of Surgery, Estonian Seamen's Hospital,
Tallinn, Estonia
From 01.Jan.1994 to 15.Nov.1995 138 patients (118 female
and 20 male) with a mean age of58 years suff'ering from
acute choleeystitis underwent gallbladder operation. In 60
cases (43 % )conventional Cholecystectomy and in 78 cases
(57 %) Laparoscopic Choleeystectomy was performed, but in
6 ofthem (4,3 %) the operative approach has to be changed
to open Choleeystectomy in case ofsevere inflammatorie
adhaesions and technical difficulties during preparation of
Calot's triangle. Intraoperative technical difficulties occured
in 24 other Laparoscopic Choleeystectomy patients, but they
needed no conversion to open procedure. Mean operating
time in laparoscopic Cholecystectomy was 96 min. It is 30
rain longer ifcompared to Laparoscopie Cholecystectomy
uncomplicated gallstone disease cases (66 rain).
postoperative complications occured in 14 cases(10,1%)
(Wound infection in 6, develop ofcardiac complaints in 4,
postoperative hernia in 2, icterus in 2 cases).
There were no patients who needed later laparatomy because of
complications and no per-or postoperative cases of death.
30 patients (21,7 %) had significant concured pathology
(M.ischaemieus, M.Hypertonicus, Diabetes mellitus, Rheuma-
toidarthritis, Bronchial asthma), and they stayed in hospital 10,4
days. Other acute eholeeystitis patients, who were operated on
laparoseopieally, spent in hospital 5.2 days.
These results show the possibility ofLaparoscopic Cholecystec-
tomy as a secure operative approach to acute cholecystitis with
nearly the same postoperative advantage than laparascopic ope-
ration for uncomplicated gallstone disease.
INTRAHEPATIC LITHIASIS SECONDARY TO
IATROGENIC BILE DUCT INJURY. A PLEA FOR THE
PERMEABLE CHOLANGIO-DIGESTIVE ANASTOMOSIS.
L.Vlad, N.Hajjar, S.Xhepa, C.Mitre
Clinic of Surgery No.3, University ofMedicine Cluj, Cluj, Romania
Our work proceed from the case of a 58 years old female patient
who underwent an open cholecistectomy for lithiasis with an
iatrogenic injury of the main bile duct during the operation.The
billiary repair was performed at once by cholangio-jejujnostomy with
Roux en Y loop. After one year, the patient became icteric, jaundice
due to biliary obstruction, that lasts permanently. 3 years after first
operation was admitted in our clinic for cholangitis and persistent
infection with bacillus Proteus. We found intraoperatively the
stenosis of cholangio-digestive anastomosis and disseminated
intrahepatic lithiasis. The type of billiary stenosis was Type III
according Bismuth's classification. The operation consisted in
abolishing of the previous anastomosis, an extended intrahepatic
desobstruction followed by cholangio-jejunostomy (with Roux en Y
loop) using the left hepatic duct according Hepp Couinaud's
technique. The disappearance of jaundice became complete only
after 3 month. The reported case is a plea for the permeable
cholangio-digestive anastomosis. Two main factors are thought to be
responsible for the formation of intrahepatic stones bile stasis and
bacterial infection. An inadequate billiary drainage due to the first
repair was responsible for the occurring of the bile stasis which aids
growth of intrahepatic calculi and aggravates bacterial infection in
situ. The prognosis of these cases depends on the level an extent of
the stenosis, inefficient previous tentative of the biliary repair and
not at the least on the experience of the operating surgeon. The
permeable anastomosis.and the adequate biliary drainage are the key
ofthe success in the repair of iatrogenic lesions ofthe bile ducts.
185
P454 P455
ENDOSCOPIC ULTRASOUND IN THE DIAGNOSIS
AND STAGING OF GALLBLADDER CARCINOMA
K.Wakabayashi, J.Ariyama, M.Suyama and K.Sato
Department of Gastroenterology, Juntendo University. Tokyo 113, Japan
Objective To evaluate the accuracy of endoscopic ultrasound in
assessing the diagnosis and staging of carcinomas of the gallbladder.
Subjects and methods During a period of'3 years,endoscopic
ultrasound (EUS) has been performed in 469 patients suspected of
having carcinoma of the gallbladder. Examination has performed using
Olympus UM-20 with 12 MHz radial transducer. In 29 patients carcinoma of
the gallbladder was histologically verified by operation. Eleven patients
were men and 18 were women,age ranged 42 to 85 years with average of
62 years.EUS images and pathological findings were compared,and
diagnostic accuracy of EUS was evaluated.
Results In 28 of 29 patients EUS accurately demonstrated carcinomaof
the gallbladder.A superficial carcinoma was depicted by EUS. Five tumors
were limited to the mlcosa,3 to the propria muscle layer,16 to the
subserosal layer and 5 invaded, beyond the subserosal layer. Depth
invasion was correctly diagnosed 14 of 29 (48%) by EUS. Depth invasion
was overestimated in 6, and underestimated in 9. In those patients EUS
failed to demonstrate 3 layer structures of the gallbladder wall.
Conclusion EUS is highly accurate in the detection of carcinomas of
the gallbladder.Also EUS provides useful information on depth invasion of
carcinomas.
CHOLANGIOCARCINOMA IN PATIENTS WITH
OPISTHORCHIASIS
P. Watanana
Department of Surgery, Faculty of Medicine Siriraj Hospital,
Mahidoi University, Bangkok 10700, Thailand
Cholangiocarcinoma is very common in endemic area of the liver fluke
Opisthorchis viverrini. Survival after surgical treatment of this cancer in
association with opisthorchiasis was prospectively studied in thirty
patients, all resided in endemic of the fluke. The median age was 52
years (range $2-69 years) and twenty five patients male. Seven
patients had their tumours removed, four with concomitant Hver
resection. Twenty two patients underwent pamative biliary bypass
procedures using the segmental duct. Laparotomy and biopsy of
metastatic lesion was done in one patient. Patients followed up to 2
years until death. The 1-YSR after tumour resection was 85.7%, and
2-YSR 42.9%. Following pamative proecedure, 1-YSR 26.1%,
all patients died within two years. The median survival was 8 months.
Survival after surgical treatment of cholangiocarcinoma in patients with
opisthorchiasis is rather similar to that reported among those without
associated liver fluke infection.
P457
THE EFFECT OF REVERSING OBSTRUCTIVE JAUNDICE
ON BOWEL-WALL PERMEABILITY IN THE RAT
C.J.Whalan,P.A. Drew, P.G.Devitt,A.R. Dennison
B.Wei and G.J. Maddern
Department of Surgery, University of Adelaide,
Adelaide, Australia
Introduction
Patients who undergo operations to relieve obstructive
jaundice (OJ) have an unusually high incidence of septic
post-operative complications, such as multisystem organ
failure. A possible trigger may be the development of
a defect in the gut barrier, allowing bacteria or
bacterial toxins to enter the tissues. We hypothesised
that the bile produced after a period of OJ may damage
the bowel wall and alter bowel permeability.
Methods
Experiment 1. A Thiry-Vella loop (TVL) was created,
and a silastic tube inserted into the bile duct, the
distal end of which was inserted into duodenum. The
tube was blocked for week to allow OJ to develop. It
was then unblocked and the TVL instilled with labelld
Escherichia coli. Twenty-four hours later bacteria in
the distant organs (liver, spleen and mesenteric lymph
nodes) were counted.
Experiment 2. A length of silastic tubing was inter-
posed between the bile duct and the duodenum.. It was
blocked for 7 days and then released, and 24 hours
later the permeability of the gut to radio-labelled
endotoxin and EDTA was measured.
Results and Conclusions
No evidence was found for increased bacterial trans-
location or bowel permeability following return of
bile to the bowel lumen.
ACALCULOUS CHOLESTEROSIS OF THE GALLBLADDER
N. Xeropotarnos, D. Karabetsos, N. Baltoyiannis, D. Cassioumis
Department of Surgery, University of Ioannina, Ioannina, Greece
The pathophysiological and clinical significance of hyperplastic
cholecystoses has not been completely understood. Cholesterosis, the
commonest of hyperplastic cholocystoses, may occasionaly give rise to
symptoms by itself.
Between February 1978 and Decerr:ber 1995, at the Surgical Department of
Ioannina Medical School, 3382 cholecystectomies had been performed.
Cholesterosis was found in 399 cases (11.8%) and among them there were
37 cases without stones (acalculous) in the gallbladder or in the choledochal
duct, a frequency of 9.3%. The material comprises 294 women and 105 men
(men to women ratio of 1:2.8) and the average age was 56 years (range 17
93 years). The diagnosis was, mainly made by ultrasonography.
The clinical symptomatology for the 37 cases without calculi in the
gallbladder was as: pain 33 (89%), dyspeptic symptom 27 (73%), jaundice
14 (38%) and fever 4 (11%). Cholecystectomy was performed in all cases
and in 4 of them choledocholithotomy was also performed.
Cholesterosis was found both macroscopically (strawberry gallbladder) and
microscopically in all cases. Cholesterosis polyps were noticed in 15 cases.
In 2 gallbladders there were also found adenomyomatosis.
While the finding of cholesterosis is not itself an indication for
cholecystectomy, in many cases may give rise to symptoms and serious
complications such as jaundice which are indistinguishable from those of
biliary lithiasis. Cholecystectomy is the only therapeutic approach.
186
P458 P459
MANAGEMENT OF THE KLATSKIN TUMORS
O.Ya0mur, H.Sonmez, A.Alparslan, FC.Ozkan, H.Ezici
Department of Surgery, University of (ukurova, Adana,
TURKEY
Carcinoma of the hepatic duct and hepatic duct
bifurcation(Klatskin tumor) are being seen with
increasing frequency and present unique problems in
management. These tumors tend to be very slow
growing, and death occurs as a result of biliary
obstruction rather than metastatic disease. Therefore,
palliative procedures had been advised. Between march
1989- June 1995 period, 13 patients with carcinoma of
the bifurcation of the hepatic ducts were treated. There
were 8(61%) male and 5(39%) female, mean age was
54.3 year. Jaundice was the most presenting symptom
among patients, followed by abdominal pain, weight loss
and pruritis. Serum liver function tests were slightly
elevated only in 3(23%). According to Child-Pugh Score,
6(46%) patients were in grade B and others in grade C.
The site and extent of the obstructive lesion was best
obtained by transhepatic cholangiography(100%) and
abdominal CT(46%). The types of procedures were
performed three categories: potentially curative resection
in 1(8%), palliative surgical bypass in 4(31%) and
operative intubation and drainage of ductal obstruction
by U-tube or internal stent insertion in 8(61%). Seven
(53.8%) patients were lost in early postoperative period.
All patients except one were unsuitable for curative
resection. Resection of the tumor is the only hope of
cure; but palliative procedures are compatible with
prolonged survival time.
P460
BILIARY INTESTINAL DRAINAGE PROCEDURE IN PRIMARY
HEPATOLITHIASIS
Takahiro Yasakal) Mitsuji Ohtsubo2) Satoshi Shirahama3) Masato
Furukawal)
Department of Surgery) Nagasaki Chuo National Hospital, Nagasaki
Japan
Department of Surgeryz) Kamigoto Hospital
Department of Internal Medicinea) Kamigoto Hospital, Nagasaki, Japan
Primary Hepatolithiasis (PHL) is the most refractory condition to
treat surgically among the various benign biliary tract diseases. Some
cases have the postoperative complications such as recurrent stones
and cholangitis. In this study, in order to examine the effect of the
biliary intestinal drainage procedure (BIDP) on the postoperative
complication, and outcome, sixty nine patients with PHL treated
surgically in our hospital from 1986 to 1995 were reviewed. The
patients were divided into a BIDP group (n=24) and non-BIDP group
(n=45). In a BIDP group, choledochojejunostomy performed in 16,
choledochoduodenostomy in 5 and papilloplasW in 3 In a non-
BIDP group, hepatectomy were performed in 31, operative extraction
of the stones in 10, percutaneous transhepatic cholangioscopic
lithotomy (PTCSL) in 4cases.
Postooerative biliarv comolications in PHL
hepatic residual and/or
cholangitis
liver
resection
cases
recurrent stones abscess
BIDP (+) 12 8 (66.7) 4 (33.3) 4*(33.3)
(n=24) (-) 12 6 (50.0) 5 (41.7) 3"(25.0)
non-BIDP (+) 31 4 (12.9) 0 0
(n=45) 14 (14.3) 0 0
Total 69 20 (29.0) 9 (13.0) 7 (10.1)
*with cholangitis
Ascending cholangitis were occurred in 9 (37.5%) and liver abscess
in ? (29.2%) of 24 cases, respectively, in BIDP group. However, no
complications were occurred in non-BIDP group regardless of
presence of hepatic resection.
It is suggested that the biliary intestinal drainage procedure is
unreasonable procedure in PHL, especially, with residual stones or
stenosis of the intrahepatic bile duct.
187
THE ROLE OF CT WITH INJECTION OF CONTRAST MATERIAL INTO
BILIARY TRACT FOR EVALUATION OF ANATOMICAL CORRELATION
B BILIARY TREES AND THE PORTAL VEIN
Yamamoto H, Watanabe K, Yamada S, Jingu K, Tsururnachi T, Fujita Y,
Honda I, Watanabe S, Satomi D, Ryu M.
Division of Gastroenterological Surgery, Chiba Cancer Center Hospital.
Chiba City, Japan.
Department of Surgery, National Cancer Center Hospital East.
CT with injection of contrast material into the biliary tract provides
informations of the anatomical correlation between biliary trees and the portal
vein. In this study, we evaluated the efficacy of this method in assessing surgical
procedure for bile duct carcinoma. Among 5 patients with hilar or diffuse bile
duct carcinoma who had undergone percutaneous tanshepatic biliary drainage
(PTBD) due to obstructive jaundice, right or left lobectomy of the liver with or
without pancreatoduodenectomy was designed in 4 cases and pancreato-
duodenectomy with resection of extrahepatic bile duct in one case by
conventional cholangiography. CT scan of the entire liver was performed after
injection of contrast material into the biliary tract through the PTBD tube
(CTPTB). In the images of CTPTB, the biliary tracts were demonstrated as high
density ducts, and the portal vein was displayed as low density structure,
compared with the liver parenchyma. Therefore, it was easy to assess the
anatomical correlation between bile ducts and the portal vein by CTPTB. In these
patients, the transection line of the bile duct and the number of bilioenteric
anastomosis was predicted, based on the location of the portal vein branches.
According to the preoperative prediction, surgical procedure was excuted. Became
bile ducts within the Glissonian sheath are embedded in connective tissue,
separation of bile ducts alone in the hepatic hilum is difficult. Therefore, during
surgery of the bile duct carcinoma in the hepatic hilum, there is difficulty in
confirming position of segmental branches of bile duct in the hepatic hilum. The
transection line of the bile duct including surrounding the connective tissue may
be determined, based on the location of the portal vein branches after
skeletonization. CTPTB provides preoperative information on anatomy of biliary
trees, based on location of branches of the portal vein. Thus, it is useful to
predict the consequence of dissection of hilar bile duct for determining the
surgical procedure for bile duct carcinoma.
P461
PROBLEMS RELATED TO T-TUBE INTUBATION OF THE
CBD FOR CHOLEDOCHOLITHIASIS
E.Yettimis,P.Vachliotis,H.Tsipras,E.Georgiopoulou,T.Togia,
M.Paulou,S.Spyrou,Th.Polymeropoulos
1st Surgical Dep,Athen's General HospitaI,Athen's,Greece
Intubation of the CBD with T-tube is the most common
procedure after choledochotomy for choledocholithiasis.ln this
work we present our 15-year experience in treating 71 pts
who underwent choledochotomy(M:45%,F:55%).The main
indications for CBD exploration were: dilated CBD
(26,5%),multiple small stones in gallbladder (24,5Vo),known
0
choledocholithiasis(19%) or obstructive jaundice (18 %).T-tube
was placed in 408 pts (57,4%). Choledochoduodenostomy
was also performed (33%) or primary closure of the CBD
(5%).During the immediate postoperative period we had
75(10,5%) complications related to the kind of operation.Of
them 24 were T-tube related: accidental removal (6),
obstruction (5), bile leakage (6), bile peritonitis on T-tube
removal (6).Other complications included: retained stones
(25),small bile leakage(13),mild postoperative pancreatitis (9),
subhepatic collection(4). Minor complications not related to
the kind of operation were noted in 126 cases (17,7%)
Reoperation was performed in 16 pts:retained stones(6),T-
tube obstruction(1),bile peritonitis (5), wound dehiscence(4).
Retained stones were also treated with ERCP(12p).Total
mortality was 2,1%.1t is concluded that (a)T-tube intubation
of the CBD is accompanied by serious complications which
need special attention and treatment.(b)ERCP is a very useful
method for pre or postoperarive extraction of CBD stones
eliminating the need for cho|edochotomy and T-tube
intubation. (c)Bile duct operations need special attention and
fine surgical technic to avoid serious postoperative
complications.
P462 P463
HU YIZE, HUANG CHUNGCHU, HU KONG
Research Section of HPB Surgery, Second Affiliated Hospital, Guangzhou
Medical College, Guangzhou, China
SKELETONIZATION RESECTION COMBINING CENTRAL HEPATIC
RESECTION AND CLINICOPATHOLOGIC STUDY FOR HILAR
CHOLANGIOCARCINOMA
There were 98 cases of extrahepatic bile duct cancer in our hospital between
October 1984 and April 1995 Among them, there were 56 cases of hilar
cholangiocarcinoma, accounting for 57 % (56/98). Since 1990,
skeletonization resection combined with central hepatic resection was
performed in 16 cases of hilar cholangiocarcinoma, in which age was from
55 to 78 years vith mean of 59 years. Resectable rate 28.5% (16/56).
After resection, the ana.'stomoses between hepatic duct stomata and Roux-en-
Y jejunal loop was performed with transanastomotic silicon stent. There was
operative mortahty.
Tumor specimens of 16 cases were available for histoloic study and
immunohstochemistry of carcinoembryonic antigen (CEA). llile of patients
were available for CEA test and clonorchiasis examination.
The results of 16 cases of clinicopathologic study and clinical follow-up
showed poorly differentiated adenocarcinoma in 7 cases (45%), well
differentiation in 8 cases (50%), malignantly adenomatoid hyperplasia in
case (5%), Colonorchiasis eggs in bile and specimens in 5 cases (31%).
CEA of bile was clear elevation in 16 cases, with mean of 81+10.6 ng/ml
(normal < 15 ng/ml). Immunohistochemistry of CEA in 100% positive
demonstration. Lymph node metastasis was in (19%). PeHneural
invasion was in 4 cases (25%). Postoparative follow-up was made in 16
cases with average survival of 20.3 months. The longest survival was 42
months in 2 cases, who are alive up to now.
In conclusion, the character of hilar cholangioccarcinoma in Guangzhou area
is that tumors have relatively large with lxor differentiation (45%).
Carcinogenic cause should be related to clonorchiasis infestation of the liver.
Poor &fferentiation and diffuse demonstration in CEA staining are
significant effects of influencing the prognosis. Skeletonization resection
combining central hepatic resection is good choice. Resection with
transanastomotie silicon stent can be to pro-long survival of patients. CEA_
test in bile preoperatively is quite useful in the diagnosis of
cholangiocarcinoma.
P464
LOWER THIRD BILE DUCT CANCER: EXPERIENCE
ON 28 CASES
A. Zrbi, G. Balzano, P. Veronesi, V. Di Carlo.
Dep.of Surgery, S. Raffaele Hospital, University of Milan, Italy.
Currently distal bile duct cancers are jointly reported with the
others tumours of the periampullary region. In order to focuse
on the own characteristics of this neoplasm, we separately
analyzed 28 cases ofresected lower third bile duct cancer
In the 1989-1995 period we performed 28
pancreatoduodenectomy (PD) for distal bile duct cancer, out of
182 PD for malignancy (15.4%). Male/female ratio was 2.6;
mean age was 62.5 yr Obstructive jaundice was the first
clinical presentation in all patients. Diagnostic investigations
included ultrasounds and CT scan in all cases, ERCP or PTC in
50% of cases. In 16 patients PD was performed with pylorus
preservation; in 10 cases intraoperative radiation therapy
(IORT) was added; 7 patients received postoperative
chemotherapy (CT). Final diagnosis was based on both
macroscopi.c and microscopic appearance and was reviewed in
all cases. Survival curves were calculated by the Kaplan-Meier
method.
In 54% of patients a mass in the pancreatic head was shown by
US and/or CT scan (mean size 3.1 cm). Final diagnosis was
preoperatively suspected in. 43% of cases.Operative mortality
was 3.4% (1 case of sepsis). Major complications were 13.7%,
minor complications 31%. Median survival was 22 months (m.),
with a 13% 5yr survival rate. Median survival was 30 m. in node
negative patients and 17 m. in node positive (p=0.08). No
difference was observed between patients undergoing pylorus
preservation or classical Whipple procedure, nor between
patients undergoing IORT and/or CT or without adjuvant
treatments.
In conclusion lower third bile duct cancer is difficult to
preoperatively differentiate from pancreatic head cancer; it
shows a severe prognosis, not significantly different from
pancreatic cancer.
188
ANALYSIS OF GENE ALTERATIONS IN HUMAN GALLBLADDER
CARCINOMA
T.Yoshida, T. Sugai, W. Habano, S. Kanno, S. Nakamura
Division of Pathology, Central Clinical Laboratory, School of Medicine, lwate
Medical University, Morioka, Japan
Considerable evidence has shown that gene alteration plays an important role in
the development and progression of a variety of tumors. However, genetic change
in gallbladder carcinoma has not yet been clarified. In this study, we analyzed
alterations of p53, APC, DCC, RB 'and K-ras genes and mierosatellite instabilities
(MSI) in 25 gallbladder carcinomas to investigate the role of genetic alterations in
their carcinogenesis, and to study correlations with elinieopathologieal factors.
Tissue samples were taken from 25 patients with gallbladder carcinomas (9
males :16 females, mean age 65 years, range: 38-79 years). Genomie DNAs were
prepared from freshly frozen surgical materials. We examined loss of
heterozygosity (LOH) at the p53, APC, DCC and RB gene regions by a
polymerase- chain-reaction (PCR)-based analysis. In addition, the p53 and APC
gene regions for allele loss were assessed using an automated fluorescent DNA
sequencer. Five mierosatellite markers were used for the determination of MSI by
PCR. Screening of sequence variations of the p53 and K-ras genes was perfpnned
by PCR-single strand conformation polymorphism (SSCP) analysis in 18 of the
25 cases. Allele loss was detected at the p53 (55%, 6/11; LOll/informative ease),
DCC (43%, 6/14), APC (31%, 5/16), andRB (13%, 1/8) loci. Two eases (8%)
showed MSI. P53 gene alterations (exon 5-8) and the K-ras gene (exonl) detected
by PCR-SSCP analysis, were found in 47% 7 /18) and 0% 0/18 ),
respectively. A higher frequency of LOH at the DCC locus was seen in the
cases with lymph node metastasis than in those without lymph node metastasis.
Gene alterations of p53, DCC and APC, rather than RB, K-ras gene alterations
and microsatellite instabilities, probably play an important role in carcinogenesis
of gallbladder carcinoma. These findings suggested that LOH at the DCC locus
gene may be related to the degree of lymph node metastasis.
P465
THE TREND OF THE GALLSTONE DISEASE IN CHINA OVER THE
PAST DECADE
Xl. Zhu, SD. Zhang, ZQ. Huang
Department of Surgery, Beijing ledical University
Beijing, China
This is the second national survey on 3911 surgically
treated cases with gallstones collected from 33
hospitals of 5 provinces and 2 main cities of China
and was aimed at reevaluating the trend of the chole-
lithiasis in China and comparing with the first
survey completed 10 years ago.
The results showed that cholelithiasis remained one
of the most common surgical disease and accounted for
11.5g of overall hospitalized patients in general
surgery at the same period of time and it occurred
mostly in females over 50 years of age with female
male ratio of 2.57. BO of the patients with single
positiohed gallstones had their stones in gallbladder
(GBS) and was mainly cholesterol.stone (CS) in nature
while pigmented(PS) bile duct stones (BDS) were found
only in 10 of the cases which made the GBS/BDS ratio
and the CS/PS ratio a significant diferonce as 7.36:1
and 3.4:1 respectively when compak.ed with the results
of 1.5:1 and 3.4:1, 10 years ago. A significant
decreased incidence of biliary ascariasis (2.41 Vs
16.1g) and biliary bacterial infection rate (54.6g Vs
71.4) as well as the dietary changes from low
protein, fat, high vegetable to the opposite were
found directly relevant to the above alterations.

				

